# Pan-cancer whole-genome analyses of metastatic solid tumours

**DOI:** 10.1038/s41586-019-1689-y

**Published:** 2019-10-23

**Authors:** Peter Priestley, Jonathan Baber, Martijn P. Lolkema, Neeltje Steeghs, Ewart de Bruijn, Charles Shale, Korneel Duyvesteyn, Susan Haidari, Arne van Hoeck, Wendy Onstenk, Paul Roepman, Mircea Voda, Haiko J. Bloemendal, Vivianne C. G. Tjan-Heijnen, Carla M. L. van Herpen, Mariette Labots, Petronella O. Witteveen, Egbert F. Smit, Stefan Sleijfer, Emile E. Voest, Edwin Cuppen

**Affiliations:** 1 Hartwig Medical Foundation, Amsterdam, The Netherlands; 2 Hartwig Medical Foundation Australia, Sydney, New South Wales Australia; 3 Center for Personalized Cancer Treatment, Rotterdam, The Netherlands; 4 Erasmus MC Cancer Institute, Rotterdam, The Netherlands; 5 Netherlands Cancer Institute/Antoni van Leeuwenhoekhuis, Amsterdam, The Netherlands; 6 Center for Molecular Medicine and Oncode Institute, University Medical Center Utrecht, Utrecht, The Netherlands; 7 Meander Medisch Centrum, Amersfoort, The Netherlands; 8 Radboud University Medical Center, Nijmegen, The Netherlands; 9 Maastricht University Medical Center, Maastricht, The Netherlands; 10 VU Medical Center, Amsterdam, The Netherlands; 11 Cancer Center, University Medical Center Utrecht, Utrecht, The Netherlands

**Keywords:** Cancer genomics, Cancer genomics

## Abstract

Metastatic cancer is a major cause of death and is associated with poor treatment efficacy. A better understanding of the characteristics of late-stage cancer is required to help adapt personalized treatments, reduce overtreatment and improve outcomes. Here we describe the largest, to our knowledge, pan-cancer study of metastatic solid tumour genomes, including whole-genome sequencing data for 2,520 pairs of tumour and normal tissue, analysed at median depths of 106× and 38×, respectively, and surveying more than 70 million somatic variants. The characteristic mutations of metastatic lesions varied widely, with mutations that reflect those of the primary tumour types, and with high rates of whole-genome duplication events (56%). Individual metastatic lesions were relatively homogeneous, with the vast majority (96%) of driver mutations being clonal and up to 80% of tumour-suppressor genes being inactivated bi-allelically by different mutational mechanisms. Although metastatic tumour genomes showed similar mutational landscape and driver genes to primary tumours, we find characteristics that could contribute to responsiveness to therapy or resistance in individual patients. We implement an approach for the review of clinically relevant associations and their potential for actionability. For 62% of patients, we identify genetic variants that may be used to stratify patients towards therapies that either have been approved or are in clinical trials. This demonstrates the importance of comprehensive genomic tumour profiling for precision medicine in cancer.

## Abstract (web summary)

The mutational landscape of metastatic cancer genomes is analysed in a large-scale, pan-cancer study of metastatic solid tumours that includes whole-genome sequencing of 2,520 tumour–normal tissue pairs.

## Main

In recent years, several large-scale whole-genome sequencing (WGS) analysis efforts have yielded valuable insights into the diversity of the molecular processes that drive different types of adult^1,2^ and paediatric^3,4^ cancer and have fuelled the promises of genome-driven oncology care^5^. However, most analyses were done on primary tumour material, whereas metastatic cancers—which cause the bulk of the disease burden and 90% of all cancer deaths—have been less comprehensively studied at the whole-genome level, with previous efforts focusing on tumour-specific cohorts^6–8^ or at a targeted gene panel^9^ or exome level^10^. As cancer genomes evolve over time, both in the highly heterogeneous primary tumour mass and as disseminated metastatic cells^11,12^, a better understanding of metastatic cancer genomes will be highly valuable to improve on adapting treatments for late-stage cancers.

**Fig. 1:**
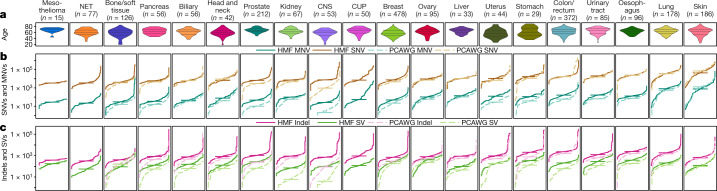
Mutational load of metastatic cancer. **a**, Violin plot showing age distribution of each tumour type, with twenty-fifth, fiftieth and seventy-fifth percentiles marked. **b**, **c**, Cumulative distribution function plot (individual samples were ranked independently for each variant type) of mutational load for each tumour type for SNVs and MNVs (**b**) and indels and SVs (**c**). The median for each tumour type is indicated by a horizontal bar. Dotted lines indicate the mutational loads in primary cancers from the PCAWG cohort^14^. Only tumour types with more than ten samples are shown (*n* = 2,350 independent patients), and are ranked from the lowest to the highest overall SNV mutation burden (TMB). CUP, cancer of unknown primary.

**Extended Data Fig. 1:**
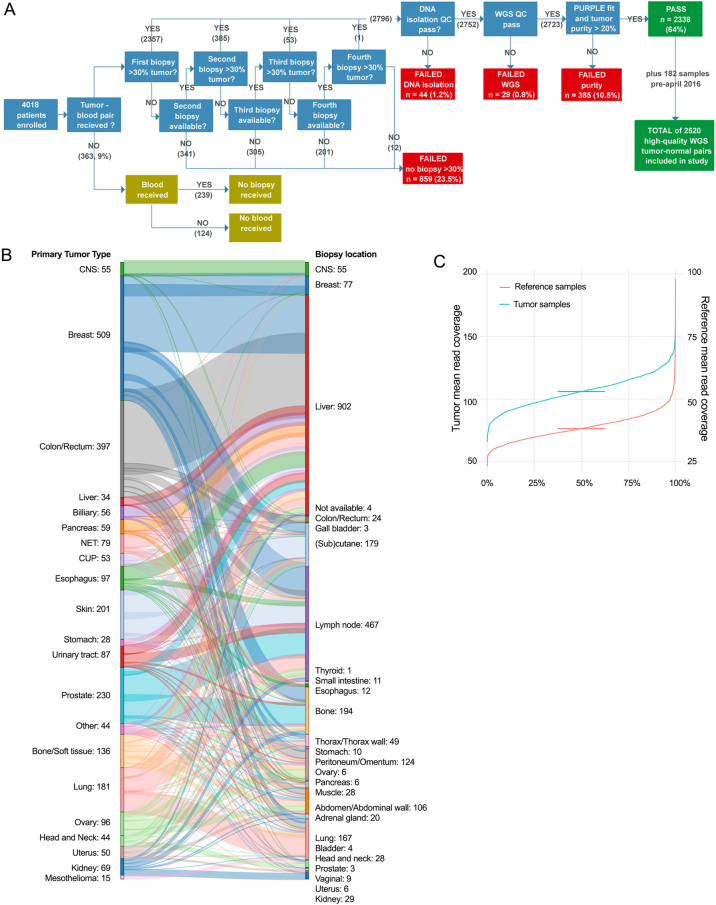
Hartwig sample workflow, biopsy locations and sequence coverage. **a**, Sample workflow from patient to high-quality WGS data. A total of 4,018 patients were enrolled in the study between April 2016 and April 2018. For 9% of patients, no blood and/or biopsy material was obtained, mostly because conditions of patients prohibited further study participation. Up to four fresh-frozen biopsies were obtained per patient, and were sequentially analysed to identify a biopsy with more than 30% tumour cellularity as determined by routine histology assessment. For 859 patients, no suitable biopsy was obtained, and 2,796 patients were further processed for WGS analysis. In total, 44 and 29 samples failed in either DNA isolation or library preparation and raw WGS data quality control tests, respectively. For a further 385 samples, the WGS data were of good quality, but the determination of tumour purity based on WGS data (PURity & PLoidy Estimator; PURPLE) was less than 20%, making reliable and comprehensive somatic variant calling impossible and were therefore excluded. Eventually, 2,338 pairs of tumour and normal tissue samples with high-quality WGS data were obtained, which were supplemented with 182 pairs from pre-April 2016, adding up to 2,520 pairs of tumour and normal samples that were included in this study. **b**, Breakdown of cohort by biopsy location. Tumour biopsies were taken from a broad range of locations. Primary tumour type is shown on the left, and the biopsy location on the right. **c**, Distribution of sample sequencing depth for tumour and blood reference samples (*n* = 2,520 independent samples for each category). The median for each is indicated by a horizontal bar.

Here we describe the pan-cancer whole-genome landscape of metastatic cancers based on 2,520 paired tumour (106× average depth) and normal (blood, 38×) genomes from 2,399 patients (Supplementary Tables 1 and 2, Extended Data Fig. 1). The sample distribution over age and primary tumour types broadly reflects the incidence of solid cancers in the Western world, including rare cancers (Fig. 1a). Sequencing data were analysed using an optimized bioinformatic pipeline based on open source tools (Methods, Supplementary Information) and identified a total of 59,472,629 single nucleotide variants (SNVs), 839,126 multiple nucleotide variants (MNVs), 9,598,205 insertions and deletions (indels) and 653,452 structural variants (SVs) (Supplementary Table 2).

## Mutational landscape of metastatic cancer

We analysed the mutational burden of each class of variant per cancer type based on the tissue of origin (Fig. 1, Supplementary Table 2). In line with previous studies on primary cancers^13,14^, we found extensive variation in the mutational load of up to three orders of magnitude both within and across cancer types.

**Extended Data Fig. 2:**
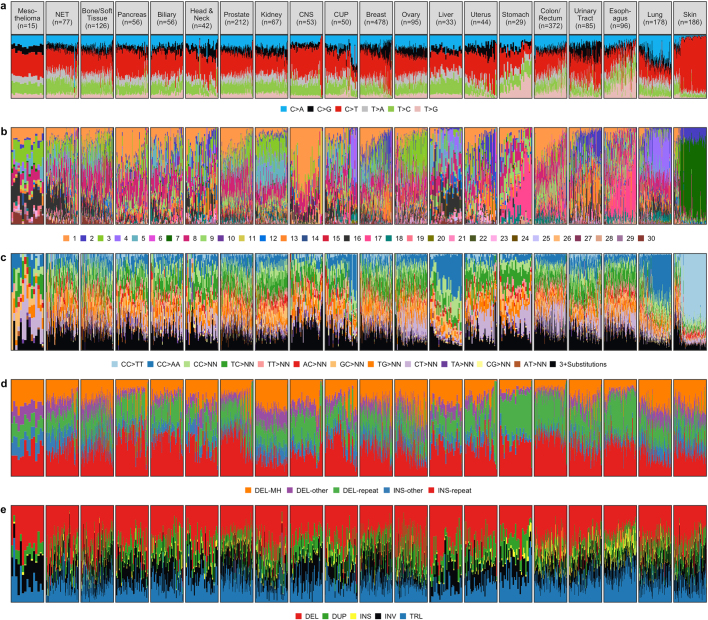
Mutational context distribution per tumour type. **a**–**e**, Variant subtype, mutational context or signature per individual sample for each SNV (**a**), SNV by COSMIC signature (**b**), MNV (**c**), indel (**d**) or SV (**e**). Each column chart is ranked within tumour type by mutational load from low to high in that variant class. MNVs are classified by the dinucleotide substitution, with ‘NN’ referring to any dinucleotide combination. SVs are classified by type. DEL, deletion (with microhomology (MH), in repeats and other); DUP, tandem duplication; INV, inversion; TRL, translocation; INS, insertion. Highly characteristic known patterns can be discerned, for example the high rates of C>T SNVs, CC>TT MNVs and COSMIC S18 for skin tumours, and high rates of C>A SNVs and COSMIC S4 for lung tumours.

**Extended Data Fig. 3:**
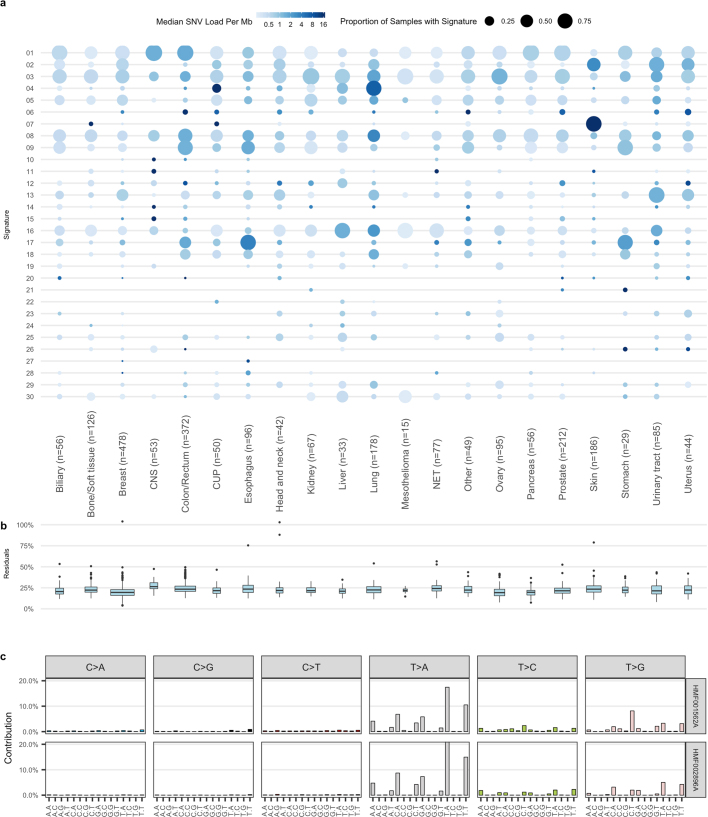
SNV mutational signatures. **a**, Prevalence and median mutational load of fitted COSMIC SNV mutational signature per cancer type (the number of patients per category is provided). The observed distribution largely reflects the patterns observed from primary cancers^13^. **b**, Box plots of relative residuals in fits per cancer type (sum of absolute difference between the fitted and actual divided by total mutational load). Boxes represent the twenty-fifth to seventy-fifth percentiles, and whiskers extend to the highest and lowest values within 1.5× the upper/lower quartile distance, with outliers shown as dots. **c**, Proportion of variants by 96 trinucleotide mutational context for two selected samples with high residuals and high mutational load. Top and bottom panels represent the highest outliers for breast (HMF002896) and oesophagus (HMF001562) cancers, respectively, from **b**. Both of these samples were previously treated with the experimental drug SYD985—a duocarmycin-based HER2-targeting antibody–drug conjugate^68^.

The median SNV counts per sample were highest in skin, predominantly consisting of melanoma (44,000) and lung (36,000) tumours, with tenfold higher SNV counts than sarcomas (4,100), neuroendocrine tumours (NETs) (3,500) and mesotheliomas (3,400). SNVs were mapped to COSMIC mutational signatures and were found to broadly match the patterns described in previous cancer cohorts per cancer type^13^ (Extended Data Figs. 2, 3). However, several broad spectrum signatures such as S3, S8, S9 and S16 as well as some more specific signature (for example, S17 in specific tumour types) appear to be overrepresented in our cohort. These observations may indicate enrichment of tumours that are deficient in specific DNA repair processes (S3), increased hypermutation processes (S9) among advanced cancers, or reflect the mutagenic effects of previous treatments^15^.

The variation for MNVs was even greater, with lung (median of 821) and skin (median of 764) tumours having five times the median MNV counts of any other tumour type. This can be explained by the well-known mutational effect of UV radiation (CC>TT) and smoking (CC>AA) mutational signatures, respectively (Extended Data Fig. 2). Although only dinucleotide substitutions are typically reported as MNVs, 10.7% of the MNVs involve three nucleotides and 0.6% had four or more nucleotides affected.

**Extended Data Fig. 4:**
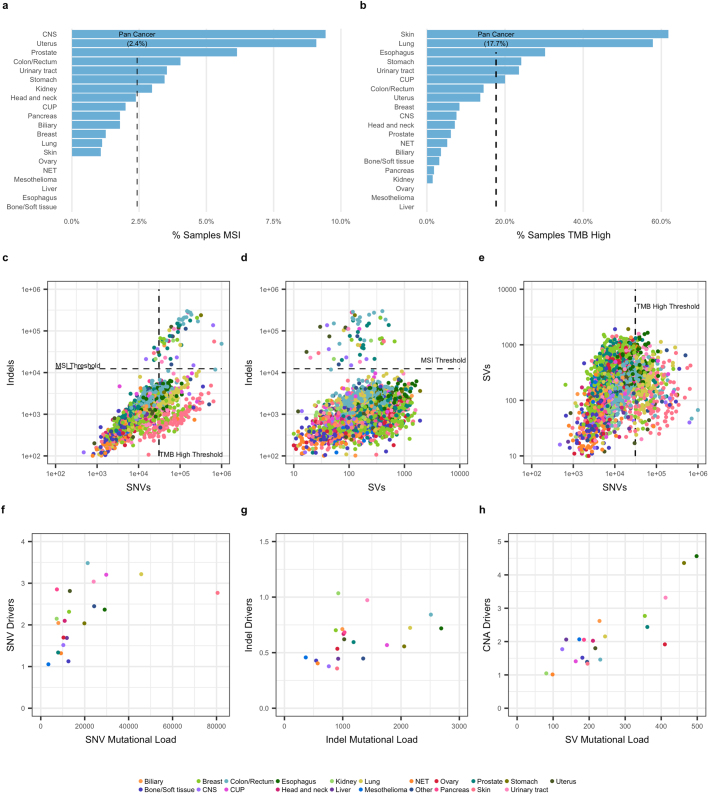
Mutational load, genome-wide analyses and drivers. **a**, Proportion of samples by cancer type classified as microsatellite instable (MSIseq score > 4). **b**, Proportion of samples with a high mutational burden (TMB > 10 SNVs per Mb). **c**–**e**, Scatter plots of mutational load per sample for indels versus SNVs (**c**), indels versus SVs (**d**), and SVs versus SNVs (**e**). MSI (MSIseq score > 4) and high TMB (>10 SNVs per Mb) thresholds are indicated. **f**–**h**, Mean mutational load versus driver rate for SNVs (**f**), indels (**g**) and SVs (**h**), grouped by cancer type. MSI samples were excluded.

Indel counts were typically tenfold lower than SNVs, with a lower relative rate for skin and lung cancers (Fig. 1c). Genome-wide analysis of indels at microsatellite loci identified 60 samples with microsatellite instability (MSI) (Supplementary Table 2), which represents 2.5% of all tumours (Extended Data Fig. 4). Notably, 67% of all indels in the entire cohort were found in the 60 MSI samples, and 85% of all indels in the cohort were found in microsatellites or short tandem repeats. The highest rates of MSI were observed in central nervous system (CNS) (9.4%), uterine (9.1%) and prostate (6.1%) tumours. For metastatic colorectal cancer lesions, we found an MSI frequency of only 4.0%, which is lower than that reported for primary colorectal cancer, and in line with better prognosis for patients with localized MSI colorectal cancer, which metastasizes less often^16^.

The median rate of SVs across the cohort was 193 per tumour, with the highest median counts observed in ovarian (412) and oesophageal (372) tumours, and the lowest in kidney tumours (71) and NETs (56). Simple deletions were the most commonly observed subtype of SV (33% of all SVs), and were the most prevalent in every cancer type except stomach and oesophageal tumours, which were highly enriched in translocations (Extended Data Fig. 2).

To gain insight into the overall genomic differences between primary and metastatic cancer, we compared the mutational burden in the Hartwig Medical Foundation (HMF) metastatic cohort with the Pancancer Analysis of Whole Genomes (PCAWG) dataset^14^, which, to our knowledge, is the largest comparable whole-genome sequenced tumour cohort (*n* = 2,583) available so far, and which has 95% of biopsies taken from treatment-naive primary tumours. In general, the SNV mutational load does not seem to be indicative for disease progression as it is not significantly different in this study compared with the PCAWG for most cancer types (Fig. 1b). Prostate and breast cancer are clear exceptions with structurally higher mutational loads (*q* < 1 × 10^−10^, Mann–Whitney test), which potentially reflects relevant tumour biology and is, for prostate cancer, consistent with other reports^8,17^. CNS tumours also have a higher mutational load that is explained by the different age distributions of the cohorts.

**Extended Data Fig. 5:**
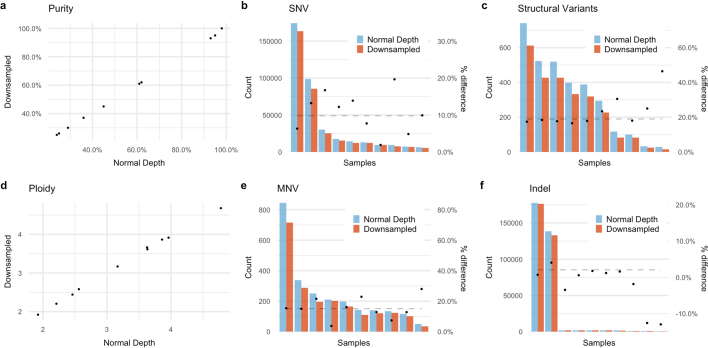
Effect of sequencing depth on variant calling. **a**–**f**, Comparison of variant calling of ten randomly selected samples at normal depth and 50% downsampled (approximately 50 times, similar to the mean coverage for the PCAWG project^14^) for purity (**a**), SNV counts (**b**), SV counts (**c**), ploidy (**d**), MNV counts (**e**) and indel counts (**f**). Decreasing coverage results in an average decrease in sensitivity of 10% for SNVs, 2% for indels, 15% for MNVs and 19% for SVs.

**Extended Data Fig. 6:**
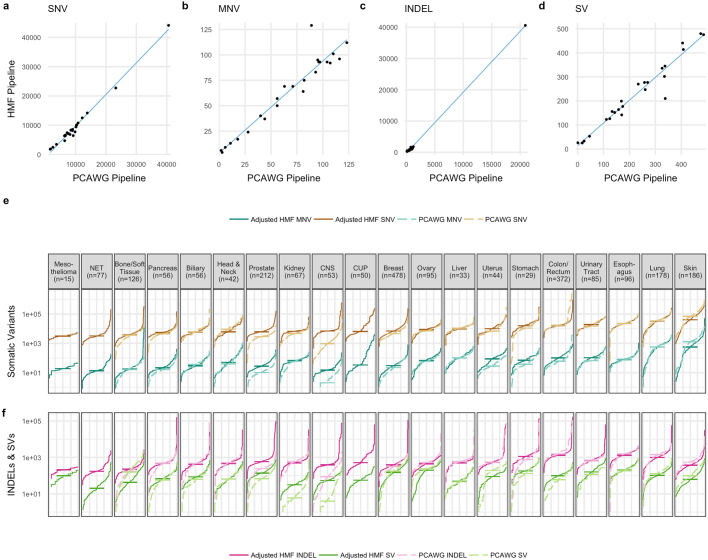
Effect of bioinformatic analysis pipeline on variant calling. **a**–**d**, Comparison of observed mutational count per sample for SNVs (**a**), MNVs (**b**), indels (**c**) and SVs (**d**) on 24 patient samples analysed by the PCAWG and HMF pipelines. The PCAWG pipeline was found to have a 43% lower sensitivity for indels (which is based on a consensus calling), 18% lower for SVs (based on a different algorithm) and 6% lower for MNVs (only includes MNVs involving two nucleotides), with nearly the same sensitivity for SNVs. **e**, **f**, Cumulative distribution function plot for each tumour type (the number of independent patients per category is provided) of coverage and pipeline-adjusted mutational load for SNVs and MNVs (**e**) and indels and SVs (**f**). Mutational loads as shown in Fig. 1 were adjusted for the sensitivity effects caused by differences in sequencing depth coverage (Extended Data Fig. 4) and analysis pipeline differences (**a**–**d**). After this correction, the TMB between primary and metastatic cohorts across all variant types are much more comparable (**e**, **f**), which indicates that technical differences do contribute to the reported mutational load differences between primary and metastatic tumours. Prostate cancer is the most notable exception, with approximately twice the TMB in all variant classes, although more subtle differences, potentially driven by biology, can also be observed for other tumour and mutation types. For cancer types that are comparable with the PCAWG cohort, the equivalent PCAWG numbers are shown by dotted lines. The median for each cohort is shown by a horizontal line.

By contrast, the mutational loads of indels, MNVs and SVs are significantly higher across nearly all cancer types analysed (Fig. 1c). This is most notable for prostate cancer, in which we observe a more than fourfold increased rate of MNVs, indels and SVs. Although these observations may represent the advancement of disease and the higher rate of certain mutational processes in metastatic cancers, they are also partially due to differences in sequencing depth and bioinformatic analysis pipelines (Extended Data Figs. 5, 6, Supplementary Information).

## Copy number alteration landscape

**Fig. 2:**
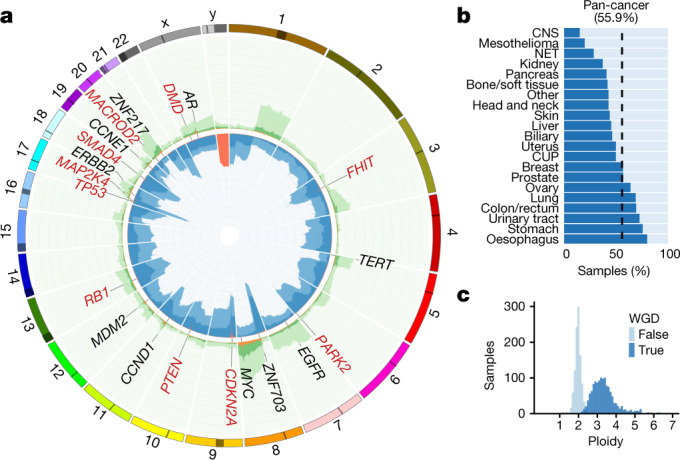
Copy number landscape of metastatic cancer. **a**, Proportion of samples with amplification and deletion events by genomic position pan-cancer. The inner ring shows the percentage of tumours with homozygous deletion (orange), LOH and significant loss (copy number < 0.6× sample ploidy; dark blue) and near copy neutral LOH (light blue). Outer ring shows percentage of tumours with high level amplification (>3× sample ploidy; orange), moderate amplification (>2× sample ploidy; dark green) and low level amplification (>1.4× amplification; light green). The scale on both rings is 0–100% and inverted for the inner ring. The most frequently observed high-level gene amplifications (black text) and homozygous deletions (red text) are shown. **b**, Proportion of tumours with a WGD event (dark blue), grouped by tumour type. **c**, Sample ploidy distribution over the complete cohort for samples with and without WGD.

Pan-cancer, the most highly amplified regions in our metastatic cancer cohort contain established oncogenes such as *EGFR*, *CCNE1*, *CCND1* and *MDM2* (Fig. 2). The chromosomal arms 1q, 5p, 8q and 20q are also highly enriched in moderate amplification across the cohort, with each affecting more than 20% of all samples. For amplifications of 5p and 8q, this is probably related to the common amplification targets of *TERT* and *MYC*, respectively. However, the targets of amplifications on 1q, which are predominantly found in breast cancers (more than 50% of samples), and amplifications on 20q, which are predominantly found in colorectal cancers (more than 65% of samples), are less clear.

Overall, an average of 23% of the autosomal DNA per tumour has loss of heterozygosity (LOH). Unsurprisingly, *TP53* has the highest LOH recurrence at 67% of samples, and many of the other LOH peaks are also explained by well-known tumour-suppressor genes (TSGs). However, several clear LOH peaks are observed that cannot easily be explained by known TSG selection, such as one on 8p (57% of samples). LOH at 8p has previously been linked to lipid metabolism and drug responses^18^, although the involvement of individual genes has not been established.

There are remarkable differences in the LOH between cancer types (Supplementary Fig. 1). For instance, we observed LOH events on the 3p arm in 90% of kidney samples^19^ and LOH of the complete chromosome 10 in 72% of CNS tumours (predominantly glioblastoma multiforme^20^). Furthermore, the mechanism for LOH in *TP53* is highly specific to tumour type, with ovarian cancers exhibiting LOH of the full chromosome 17 in 75% of samples, whereas in prostate cancer (also 70% LOH for *TP53*) this is nearly always caused by highly focal deletions.

**Extended Data Fig. 7:**
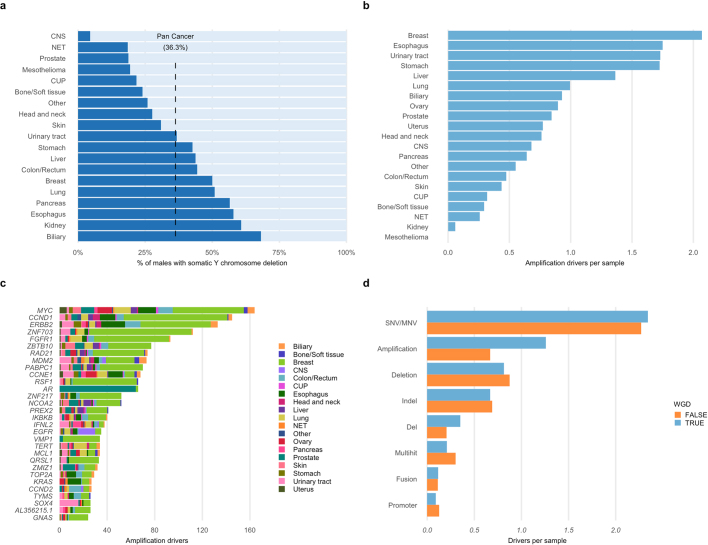
Somatic Y chromosome loss and driver amplifications. **a**, Proportion of male tumours with somatic loss of more than 50% of Y chromosome (dark blue) grouped by tumour type. **b**, Mean rate of amplification drivers per cancer type. **c**, Breakdown of the number of amplification drivers per gene by cancer type. **d**, Mean rate of drivers per variant type for samples with and without WGD.

Unlike LOH events, homozygous deletions are nearly always restricted to small chromosomal regions. Not a single example was found in which a complete autosomal arm was homozygously deleted. Homozygous deletions of genes are also surprisingly rare: we found only a mean of 2.0 instances per tumour in which one or several consecutive genes are fully or partially homozygously deleted. In 46% of these events, a putative TSG was deleted. Loss of chromosome Y is a special case and is deleted in 36% of all male tumour genomes but varies strongly between tumour types, from 5% deleted in CNS tumours to 68% deleted in biliary tumours (Extended Data Fig. 7).

An extreme form of copy number change can be caused by whole-genome duplication (WGD). We found WGD events in 56% of all samples ranging from 15% in CNS to 80% in oesophageal tumours (Fig. 2). This is much higher than previously reported for primary tumours (25–37%)^21,22^ and from panel-based sequencing analyses of advanced tumours (30%)^23^.

## Significantly mutated genes

**Extended Data Fig. 8:**
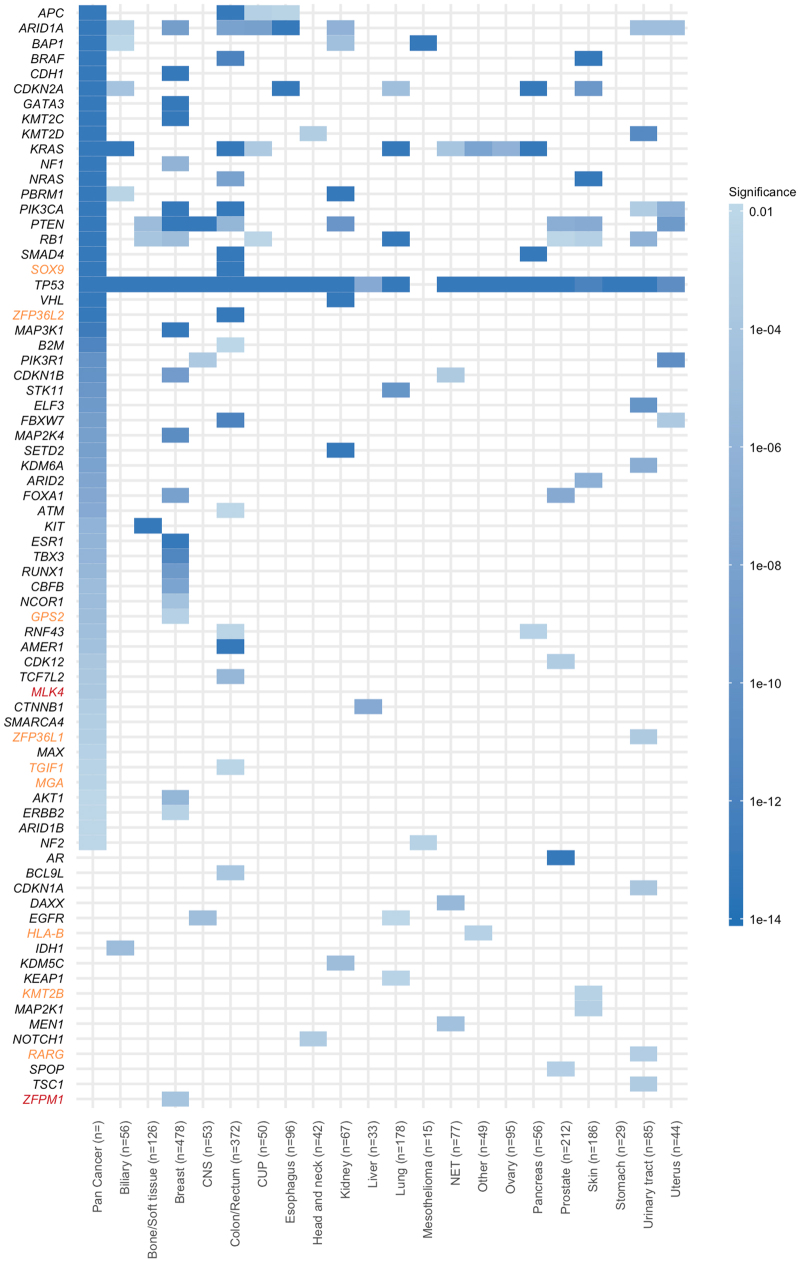
Significantly mutated genes. Tile chart showing genes found to be significantly mutated per cancer type (the number of independent patients per category is provided) and pan-cancer using dNdScv. Gene names marked in orange are also significant in a previous study^24^, but not found in the COSMIC gene census or curated gene databases. Gene names marked in red are novel in this study. Significance (Poisson with Benjamini–Hochberg false discovery rate correction) is indicated by the intensity of shading.

Analyses for significantly mutated genes using strict significance cut-off values (*q* < 0.01) reproduced previous results on cancer drivers^24^, and identified a few novel genes that are potentially related to metastatic cancer (Extended Data Fig. 8, Supplementary Table 3). In the pan-cancer analyses, we identified *MLK4* (also known as *MAP3K21*; *q* = 2 × 10^−4^)—a mixed lineage kinase that regulates the JNK, P38 and ERK signalling pathways and has been reported to inhibit tumorigenesis in colorectal cancer^25^. In addition, in our tumour type-specific analyses, we identified a metastatic breast cancer-specific significantly mutated gene—*ZFPM1* (also known as *FOG1*; *q* = 8 × 10^−5^), a zinc-finger transcription factor protein without clear links to cancer. Our cohort also lends support to previous findings for significantly mutated genes that are currently not included in the COSMIC Cancer Gene Census^26^. In particular, eight significantly mutated putative TSGs found previously in an independent dataset^24^ were also found in our analyses, including *GPS2* (pan-cancer, breast), *SOX9* (pan-cancer, colorectal), *TGIF1* (pan-cancer, colorectal), *ZFP36L1* (pan-cancer, urinary tract) and *ZFP36L2* (pan-cancer, colorectal), *HLA-B* (lymphoid), *MGA* (pan-cancer), *KMT2B* (skin) and *RARG* (urinary tract).

We also searched for genes that were significantly amplified or deleted (Supplementary Table 4). *CDKN2A* and *PTEN* were the most significantly deleted genes overall, but many of the top genes involved common fragile sites, particularly *FHIT* and *DMD*, which were deleted in 5% and 4% of samples, respectively. The role of common fragile sites in tumorigenesis is unclear and aberrations that affect these genes are frequently treated as passenger mutations that reflect localized genomic instability^27^. In *CTNNB1*, we identified a recurrent in-frame deletion of the complete exon 3 in 12 samples, 9 of which are colorectal cancers. Notably, these deletions were homozygous but thought to be activating as *CTNNB1* normally acts as an oncogene in the WNT and β-catenin pathway and none of these nine colorectal samples had any *APC* driver mutations. We also identified several significantly deleted genes not previously reported, including *MLLT4* (*n* = 13) and *PARD3* (*n* = 9).

Unlike homozygous deletions, amplification peaks tend to be broad and often encompass large numbers of genes, making identification of the amplification target challenging. However, *SOX4* (6p22.3) stands out as a significantly amplified single gene peak (26 amplifications) and is highly enriched in urinary tract cancers (19% of samples highly amplified). *SOX4* is known to be overexpressed in prostate, hepatocellular, lung, bladder and medulloblastoma cancers with poor prognostic features and advanced disease status and is a modulator of the PI3K and Akt signalling pathway^28^.

Also notable was a broad amplification peak of 10 genes around *ZMIZ1* at 10q22.3 (*n* = 32), which has not previously been reported. *ZMIZ1* is a transcriptional coactivator of the protein inhibitor of activated STAT (PIAS)-like family and is a direct and selective cofactor of NOTCH1 in the development of T cells and leukaemia^29^. *CDX2*, previously identified as an amplified lineage-survival oncogene in colorectal cancer^30^, is also highly amplified in our cohort with 20 out of 22 amplified samples found in colorectal cancer, representing 5.4% of all colorectal samples.

## Driver mutation catalogue

We created a comprehensive catalogue of mutations in known (COSMIC curated genes^31^) and newly discovered (ref. ^24^ and this study) cancer genes across all samples and variant classes, similar to that previously described for primary tumours^32^ (N. Lopez, personal communication). We used a prioritization scheme to give a likelihood score for each mutation being a potential driver event. By taking into account the proportion of SNVs and indels estimated to be passengers using the dNdScv R package, we found 13,384 somatic candidate driver events among the 20,071 identified mutations in the combined gene panel (Supplementary Table 5), together with 189 germline predisposition variants (Supplementary Table 6). The somatic candidate drivers include 7,400 coding mutations, 615 non-coding point-mutation drivers, 2,700 homozygous deletions (25% of which are in common fragile sites), 2,392 focal amplifications and 276 fusion events. For non-coding variants, only essential splice sites and promoter mutations in *TERT* were included in the study owing to the current lack of robust evidence for other recurrent oncogenic non-coding mutations^33^. A total of 257 variants were found at 5 known recurrent variant hotspots^9^ and included in the candidate driver catalogue.

For the cohort as a whole, 55% of point mutations in the gene panel candidate driver catalogue were predicted to be genuine driver events, using our prioritization scheme (Methods). To facilitate the analysis of variants of unknown significance at a per-patient level, we calculated a sample-specific likelihood score for each point mutation being a driver event by taking into account the mutational burden of the sample, the biallelic inactivation status for TSGs, and hotspot positions for oncogenes. Predictions of pathogenic variant overlap with known biology—for example, clustering of benign missense variants in the 3′ half of the *APC* gene (Supplementary Fig. 2)—fits with the absence of FAP-causing germline variants in this part of the gene^34^.

**Fig. 3:**
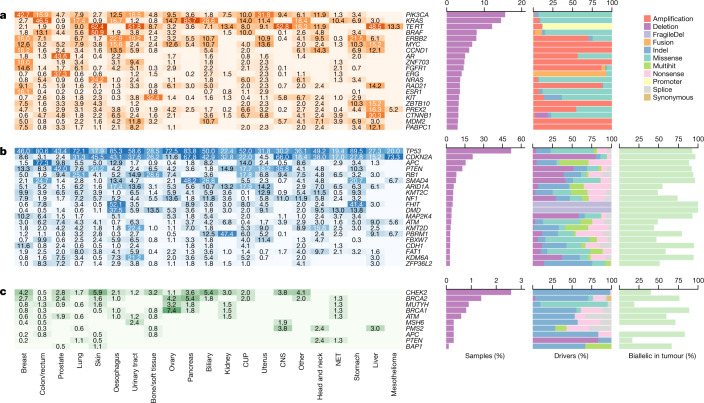
The most prevalent driver genes in metastatic cancer. **a**–**c**, The most prevalent somatically mutated oncogenes (**a**), TSGs (**b**) and germline predisposition variants (**c**). From left to right, the heat map shows the percentage of samples in each cancer type that are found to have each gene mutated; absolute bar chart shows the pan-cancer percentage of samples with the given gene mutated; relative bar chart shows the breakdown by type of alteration. For TSGs (**b**), the final bar chart shows the percentage of samples with a driver in which the gene is biallelically inactivated, and for germline predisposition variants (**c**), the final bar chart shows the percentage of samples with loss of wild type in the tumour.

Overall, the catalogue is similar to previous inventories of cancer drivers, with *TP53* (52% of samples), *CDKN2A* (21%), *PIK3CA* (16%), *APC* (15%), *KRAS* (15%), *PTEN* (13%) and *TERT* (12%) identified as the most commonly mutated genes, which together make up 26% of all the candidate driver mutations in the catalogue (Fig. 3). However, all of the ten most frequently mutated genes in our catalogue were reported at a higher rate than for primary cancers^35^, which may reflect the more advanced disease state. *AR* and *ESR1* in particular are more prevalent, with putative driver mutations in 44% of prostate and 16% of breast cancers, respectively. Both genes are linked to resistance to hormonal therapy, a common treatment for these tumour types, and have been previously reported as enriched in advanced metastatic cancer^9^ but are identified at higher rates in this study.

**Fig. 4:**
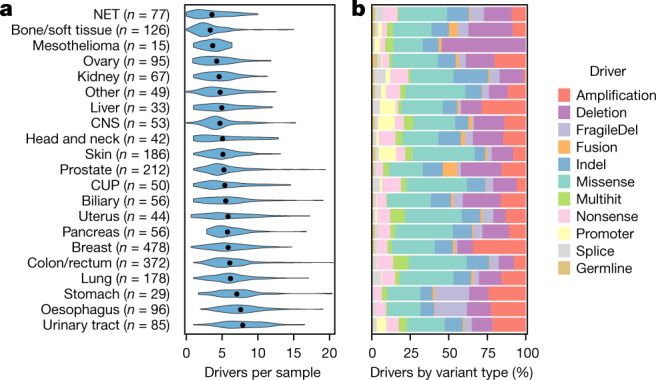
Number of drivers and types of mutation per sample by tumour type. **a**, Violin plot showing the distribution of the number of drivers per sample grouped by tumour type (number of patients per tumour type is provided). Black dots indicate the mean values for each tumour type. **b**, Relative bar chart showing the breakdown per cancer type of the type of alteration.

At the per-patient level, the mean number of total candidate driver events per patient was 5.7, with the highest rate in urinary tract tumours (mean value of 8.0) and the lowest in NETs (mean of 2.8) (Fig. 4). Oesophageal and stomach tumours also had increased driver counts, largely owing to a much higher rate of deletions in common fragile site genes (mean of 1.6 for both stomach and oesophageal tumours) compared with other cancer types (pan-cancer mean of 0.3). Fragile sites aside, the differential rates of drivers between cancer types in each variant class do correlate with the relative mutational load (Extended Data Fig. 4), with the exception of skin cancers, which have a lower than expected number of SNV drivers.

In 98.6% of all samples, at least one somatic candidate driver mutation or germline predisposition variant was found. Of the 34 samples with no identified driver, 18 were NETs of the small intestine (representing 49% of all patients of this subtype). This probably indicates that small intestine NETs have a distinct set of yet drivers that are not captured in any of the cancer gene resources used and are also not prevalent enough in our relatively small NET cohort to be detected as significant. Alternatively, NETs could be mainly driven by epigenetic mechanisms that are not detected by WGS^36^.

The number of amplified driver genes varied significantly between cancer types (Extended Data Fig. 7), with highly increased rates per sample in breast cancer (mean of 2.1), oesophageal cancer (mean of 1.8), urinary tract and stomach cancers (both mean of 1.7), nearly no amplification drivers in kidney cancer (mean of 0.1), and none in the mesothelioma cohort. In tumour types with high rates of amplifications, these amplifications are generally found across a broad spectrum of oncogenes, which suggests that there are mutagenic processes active in these tissues that favour amplifications, rather than tissue-specific selection of individual driver genes. *AR* and *EGFR* are notable exceptions, with highly selective amplifications in prostate cancer, and in CNS and lung cancers, respectively, in line with previous reports^20,37,38^. Notably, we also found twofold more amplification drivers in samples with WGD events despite amplifications being defined as relative to the average genome ploidy.

The 189 germline variants identified in 29 cancer predisposition genes (present in 7.9% of the cohort) consisted of 8 deletions and 181 point mutations (Fig. 3c, Supplementary Table 6). The top five affected genes (containing nearly 80% of variants) were the well-known germline drivers *CHEK2*, *BRCA2*, *MUTYH*, *BRCA1* and *ATM*. The corresponding wild-type alleles were found to be lost in the tumour sample in more than half of the cases, either by LOH or somatic point mutation, indicating a high penetrance for these variants, particularly in *BRCA1* (89% of cases), *APC* (83%) and *BRCA2* (79%).

The 276 fusions consisted of 168 in-frame coding fusions, 90 *cis*-activating fusions that involve repositioning of regulatory elements in 5′ genic regions, and 18 in-frame intragenic deletions in which one or more exons was deleted (Supplementary Table 7). *ERG* (*n* = 88), *BRAF* (*n* = 17), *ERBB4* (*n* = 16), *ALK* (*n* = 12), *NRG1* (9 samples) and *ETV4* (*n* = 7) were the most commonly observed 3′ partners, which together make up more than half of the fusions. In total, 76 out of the 89 *ERG* fusions were *TMPRSS2*–*ERG* and affected 36% of all prostate cancer samples in the cohort. There were 146 fusion pairs not previously recorded in CGI, OncoKb, COSMIC or CIViC databases^31,39–41^.

**Extended Data Fig. 9:**
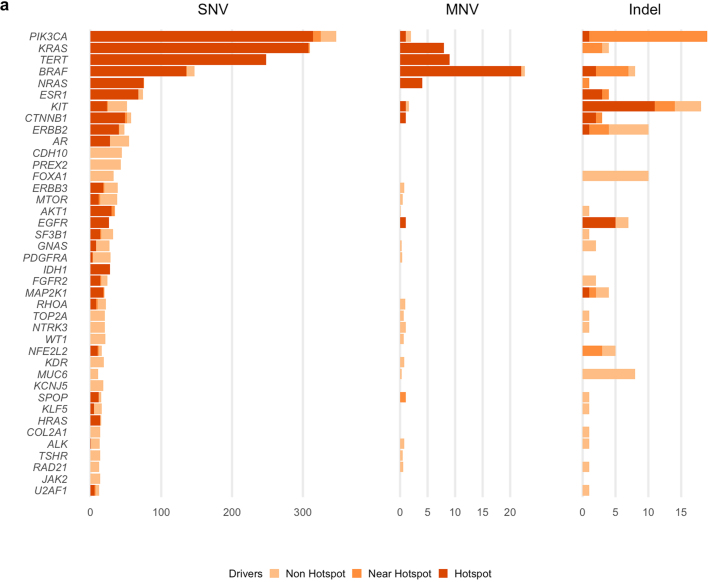
Oncogenic hotspots. Count of driver point mutations by variant type. Known pathogenic mutations curated from external databases are categorized as hotspot mutations. Mutations within five bases of a known pathogenic mutation are shown as near hotspot, and all other mutations are shown as non-hotspot.

We found that 71% of somatic driver point mutations in oncogenes occur at or within five nucleotides already known to pathogenic mutational hotspots. In the six most prevalent oncogenes (*KRAS*, *PIK3CA*, *BRAF*, *NRAS*, *TERT* and *ESR1*), the rate was 97% (Extended Data Fig. 9). Furthermore, in many of the key oncogenes, we document several likely activating but non-canonical variants near known mutational hotspots, particularly in-frame indels. Despite in-frame indels being exceptionally rare overall (mean of 1.7 per tumour), we found an excess in known oncogenes including *PIK3CA* (*n* = 18), *KIT* (*n* = 17), *ERBB2* (*n* = 10) and *BRAF* (*n* = 8) frequently occurring at or near known hotspots (Extended Data Fig. 9). In *FOXA1*, we identified ten in-frame indels that are highly enriched in prostate cancer (seven out of ten cases) and clustered at two locations that were not previously associated with pathogenic mutations^42^.

For TSGs, our results strongly support the Knudson two-hit hypothesis^43^, with 80% of all TSG drivers found to have biallelic inactivation by genetic alterations (Fig. 3), homozygous deletion (32%), multiple somatic point mutations (7%), or a point mutation in combination with LOH (41%). This rate is, to our knowledge, the highest observed in any large-scale WGS cancer study. For many key TSGs, the biallelic inactivation rate is almost 100%—*TP53* (93%), *CDKN2A* (97%), *RB1* (94%), *PTEN* (92%) and *SMAD4* (96%)—which suggests that biallelic genetic inactivation of these genes is a strong requirement for metastatic cancer. Other prominent TSGs, however, have lower biallelic inactivation rates, including *ARID1A* (55%), *KMT2C* (49%) and *ATM* (49%). For these cases, the other allele may also be inactivated by non-mutational epigenetic mechanisms, or tumorigenesis may be driven via a haploinsufficiency mechanism.

**Extended Data Fig. 10:**
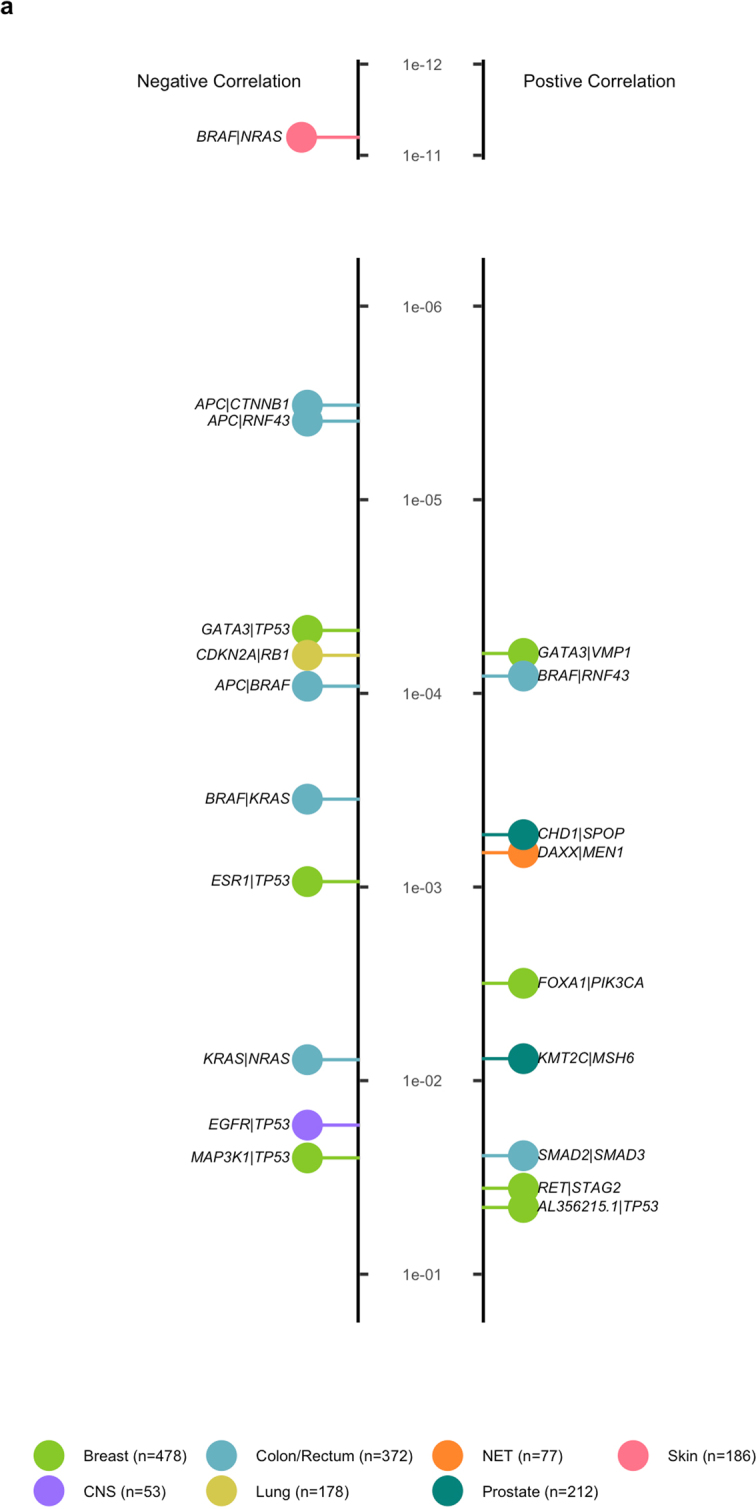
Driver co-occurrence. **a**, Mutated driver gene pairs that are significantly positively (right) or negatively (left) correlated in individual tumour types (number of independent samples per tumour type is indicated in Fig. 1) sorted by *q* value (Fisher exact test adjusted for false discovery rate). Pairs of genes on the same chromosome that are frequently co-amplified or co-deleted by chance are excluded from positively correlated results. The 20 significant findings include previously reported co-occurrence of mutated *DAX*–*MEN1* in pancreatic NET (*q* = 7 × 10^−4^), and *CDH1*–*SPOP* in prostate tumours (*q* = 5 × 10^−4^), as well as negative associations of mutated genes within the same signal transduction pathway such as *KRAS*–*BRAF* (*q* = 4 × 10^−4^) and *KRAS*–*NRAS* (*q* = 0.008) in colorectal cancer, *BRAF*–*NRAS* in skin cancer (*q* = 6 × 10^−12^), *CDKN2A*–*RB1* in lung cancer (*q* = 8 × 10^−5^) and *APC*–*CTNNB1* in colorectal cancer (*q* = 3 × 10^−6^). *APC* is also strongly negatively correlated with both *BRAF* (*q* = 9 × 10^−5^) and *RNF43* (*q* = 4 × 10^−6^), which together are characteristic of the serrated molecular subtype of colorectal cancers^69^. *SMAD2*–*SMAD3* are highly positively correlated in colorectal cancer (*q* = 0.02), which supports a previous report in a large cohort of colorectal cancers^70^. In breast cancer, we found several novel relationships, including a positive relationship for *GATA3*–*VMP1*(q = 6 × 10^−5^) and *FOXA1*–*PIK3CA* (*q* = 3 × 10^−3^), and a negative relationship for *ESR1*–*TP53* (*q* = 9 × 10^−4^) and *GATA3*–*TP53* (*q* = 5 × 10^−5^).

We examined the pairwise co-occurrence of driver gene mutations per cancer type and found ten combinations of genes that were significantly mutually exclusively mutated, and ten combinations of genes that were significantly concurrently mutated (Extended Data Fig. 10). Although most of these relationships are well established, in breast cancer, we found new positive relationship for *GATA3*–*VMP1* (*q* = 6 × 10^−5^) and *FOXA1*–*PIK3CA* (*q* = 3 × 10^−3^), and negative relationships for *ESR1–TP53* (*q* = 9 × 10^−4^) and *GATA3*–*TP53* (*q* = 5 × 10^−5^). These findings will need further validation and experimental follow-up to understand the underlying biology.

## Clonality of variants

**Extended Data Fig. 11:**
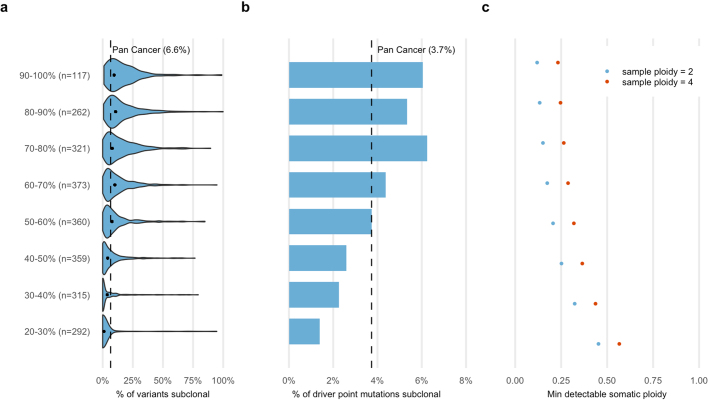
Subclonality of somatic variants. **a**, Violin plot showing the percentage of point mutations per tumour purity bucket (the number of independent samples per category is indicated) that are subclonal in each purity bucket per sample. Black dots indicate the mean for each bucket. **b**, Percentage of driver point mutations that are subclonal in each purity bucket. **c**, Approximate somatic ploidy detection cut-off of the HMF pipeline at median 106× depth coverage for each purity bucket and for sample ploidy 2 and 4. Subclonal variants with cellular fraction less than this cut-off are unlikely to be detected by our pipeline analyses.

To obtain insight into ongoing tumour evolution dynamics, we examined the clonality of all variants. Notably, only 6.6% of all SNVs, MNVs and indels across the cohort and just 3.7% of the point-mutation drivers were found to be subclonal (Extended Data Fig. 11). The low proportion of samples with subclonal variants could be partially due to the detection limits of the sequencing approach (sequencing depth, bioinformatic analysis settings), particularly for low purity samples. However, even for samples with more than 80% purity, the total proportion of subclonal variants only reaches 10.6% (Extended Data Fig. 11). Furthermore, sensitized detection of variants at hotspot positions in cancer genes showed that our analysis pipeline detected over 96% of variants with allele frequencies above 3%. Although the cohort contains some samples with high fractions of subclonal variants, overall the metastatic tumour samples are relatively homogeneous without the presence of multiple diverged major subclones. Low intratumour heterogeneity may be in part attributed to the fact that nearly all biopsies were obtained by a core needle biopsy, which results in highly localized sampling, but is nevertheless much lower than previous observations in primary cancers^12^.

In the 117 patients with independently collected repeat biopsies from the same patient (Supplementary Table 8), we found 11% of all SNVs to be subclonal. Although 71% of clonal variants were shared between biopsies, only 29% of the subclonal variants were shared. We cannot exclude the presence of larger amounts of lower frequency subclonal variants, and our results suggest a model in which individual metastatic lesions are dominated by a single clone at any one point in time and that more limited tumour evolution and subclonal selection takes places after distant metastatic seeding. This contrasts with observations in primary tumours, in which larger degrees of subclonality and several major subclones are more frequently observed^12,44^, but supports other recent studies that demonstrate minimal driver gene heterogeneity in metastases^6,45^.

## Clinical associations

**Fig. 5:**
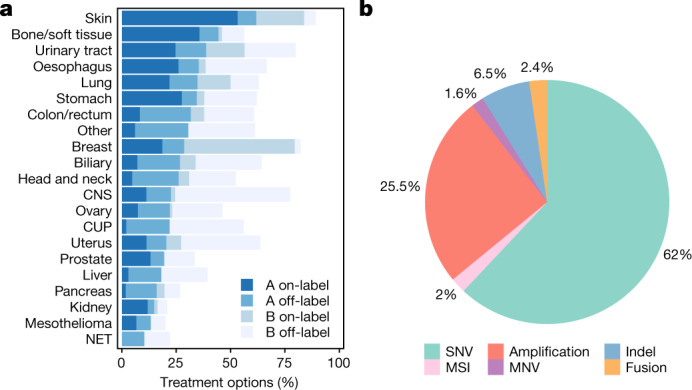
Clinical associations and actionability. **a**, Percentage of samples in each cancer type with a putative candidate actionable mutation based on data in the CGI, CIViC and OncoKB databases. Level A represents presence of biomarkers with either an approved therapy or guidelines, and level B represents biomarkers with strong biological evidence or clinical trials that indicate that they are actionable. On-label indicates treatment registered by federal authorities for that tumour type, whereas off-label indicates a registration for other tumour types. **b**, Break down of the actionable variants by variant type.

We analysed opportunities for biomarker-based treatment for all patients by mapping driver events to clinical annotation databases (CGI^41^, CIViC^39^ and OncoKB^40^). In 1,480 patients (62%), at least one predicted candidate ‘actionable’ event was identified (as defined in the Methods, Supplementary Table 9), in line with results from primary tumours^32^. Half of the patients with a predicted candidate actionable event (31% of total) contained a biomarker with a predicted sensitivity to a drug at level A (approved anti-cancer drugs) and lacked any known resistance biomarkers for the same drug (Fig. 5a). In 18% of patients, the suggested therapy was a registered indication, whereas in 13% of cases it was outside the labelled indication. In a related pilot study with implementation in 215 treated patients, we showed that such treatment with anticancer drugs outside of their approved label can result in overall clinical benefits^46^. In a further 31% of patients, a level B (experimental therapy) biomarker was identified. The predicted actionable events spanned all variant classes including 1,815 SNVs, 48 MNVs, 190 indels, 745 copy number alterations, 69 fusion genes and 60 patients with microsatellite instability (Fig. 5b).

Tumour mutation burden (TMB) is an important emerging biomarker for responses to immune checkpoint inhibitor therapy as it is a proxy for the amount of neo-antigens in the tumour cells. In two large phase 3 trials of patients with non-small-cell lung cancer, both progression-free survival and overall survival are significantly improved with first line immunotherapy as compared with chemotherapy for patients whose tumours have a TMB of greater than 10 mutations per megabase^47,48^.

Although various clinical studies based on this parameter are currently emerging, TMB was not yet included in the above actionability analysis. However, when applying this cut-off to all samples in our cohort, 18% of patients would qualify, varying from 0% for patients with mesothelioma, liver and ovarian cancers to more than 50% for patients with lung and skin cancers (Extended Data Fig. 4b).

## Data availability and resource access

The Hartwig Medical cohort described here is, to our knowledge, the largest metastatic whole-genome cancer resource, and based on a broad patient consent was specifically developed as a community resource for international academic cancer research. Somatic variants and basic clinical data (tumour type, gender, age) are publicly available and can be explored at the patient, cohort and gene level through a graphical interface (database.hartwigmedicalfoundation.nl) originally developed by the International Cancer Genome Consortium^49^. Patient-level genome-wide germline and somatic data (raw BAM files and annotated variant call data) are considered privacy sensitive and available through an access-controlled mechanism (see www.hartwigmedicalfoundation.nl/en for details).

The cohort is still expanding, with data from 4,000 patients already available, and includes data that go beyond the basic clinical and genomic data analysed in this paper such as post-biopsy treatments and responses, and previous treatment information.

## Discussion

Genomic testing of tumours faces numerous challenges in meeting clinical needs, including the interpretation of variants of unknown significance, the steadily expanding universe of actionable genes—often with an increasingly small fraction of patients affected—and the development of advanced genome-derived biomarkers such as tumour mutational load, DNA repair status and mutational signatures. Our results demonstrate that WGS analyses of metastatic cancer can provide novel and relevant insights and are instrumental in addressing some of the key challenges of precision medicine in cancer.

First, our systematic and large-scale pan-cancer analyses on metastatic cancer tissue allowed for the identification of several cancer drivers and mutation hotspots. Second, the driver catalogue analyses can be used to mitigate the problem of variants of unknown significance interpretation^32^ both by leveraging previously identified pathogenic mutations (accounting for more than two-thirds of oncogenic point-mutation drivers) and by careful analysis of the biallelic inactivation of putative TSGs that accounts for over 80% of TSG drivers in metastatic cancer. Third, we demonstrate the importance of accounting for all types of variant, including large-scale genomic rearrangements (via fusions and copy number alteration events), which account for more than half of all drivers, but also activating MNVs and indels that we have shown are commonly found in many key oncogenes. Fourth, we have shown that using WGS, even with very strict variant calling criteria, we could find candidate driver variants in more than 98% of all metastatic tumours, including predicted putatively actionable events in a clinical and experimental setting for up to 62% of patients.

Although we did not find metastatic tumour genomes to be fundamentally different from primary tumours in terms of the mutational landscape or genes that drive advanced tumorigenesis, we described characteristics that could contribute to responsiveness to therapy or resistance in individual patients. In particular, we showed that WGD events are a more pervasive element of tumorigenesis than previously understood, affecting over half of all metastatic cancers. We also found metastatic lesions to be less heterogeneous than reported for primary tumours, although the limited sequencing depth does not allow conclusions to be made about low-frequency subclonal variants.

The cohort described here provides a valuable complementary resource to whole-sequence-based data of primary tumours such as the PCAWG project in advancing fundamental and translational cancer research. Although it was established as a pan-cancer resource, several of the tumour type-specific cohorts are very large in their own rights. Already two of these cohorts (prostate^50^ and breast^51^) have been analysed in more detail, providing enhanced cancer subtype stratification and revealing characteristic genomic differences between primary and metastatic tumours. As the Hartwig Medical cohort includes a mix of treatment-naive metastatic patients and patients who have undergone (extensive) previous systemic treatments, it provides unique opportunities to study responses and resistance to treatments and discover predictive biomarkers, as these data are available for discovery and validation studies.

## Methods

A detailed description of methods and validations is available as Supplementary Information. No statistical methods were used to predetermine sample size. The experiments were not randomized, and investigators were not blinded to allocation during experiments and outcome assessment.

### Sample collection

Patients with advanced cancer not curable by local treatment options and being candidates for any type of systemic treatment and any line of treatment were included as part of the CPCT-02 (NCT01855477) and DRUP (NCT02925234) clinical studies, which were approved by the medical ethical committees (METC) of the University Medical Center Utrecht and the Netherlands Cancer Institute, respectively. A total of 41 academic, teaching and general hospitals across The Netherlands participated in these studies and collected material and clinical data by standardized protocols^52^. Patients have given explicit consent for whole-genome sequencing and data sharing for cancer research purposes. Core needle biopsies were sampled from the metastatic lesion, or when considered not feasible or not safe, from the primary tumour site and frozen in liquid nitrogen. A single 6-μm section was collected for haematoxylin and eosin (H&E) staining and estimation of tumour cellularity by an experienced pathologist and 25 sections of 20-μm were collected in a tube for DNA isolation. In parallel, a tube of blood was collected. Leftover material (biopsy, DNA) was stored in biobanks associated with the studies at the University Medical Center Utrecht and the Netherlands Cancer Institute.

### Whole-genome sequencing and variant calling

DNA was isolated from biopsies (>30% tumour cellularity) and blood according to the supplier’s protocols (Qiagen) using the DSP DNA Midi kit for blood and QIAsymphony DSP DNA Mini kit for tissue. A total of 50–200 ng of DNA (sheared to average fragment length of 450nt) was used as input for TruSeq Nano LT library preparation (Illumina). Barcoded libraries were sequenced as pools on HiSeqX generating 2 × 150 read pairs using standard settings (Illumina). BCL output was converted using bcl2fastq tool (Illumina, v.2.17 to v.2.20) using default parameters. Reads were mapped to the reference genome GRCH37 using BWA-mem v.0.7.5a^53^, duplicates were marked for filtering and INDELs were realigned using GATK v.3.4.46 IndelRealigner^54^. GATK HaplotypeCaller v.3.4.46^55^ was run to call germline variants in the reference sample. For somatic SNV and indel variant calling, GATK BQSR^56^ was applied to recalibrate base qualities. SNV and indel somatic variants were called using Strelka v.1.0.14^57^ with optimized settings and post-calling filtering. Structural Variants were called using Manta (v.1.0.3)^58^ with default parameters followed by additional filtering to improve precision using an internally built tool (Breakpoint-Inspector v.1.5). To assess the effect of sequencing depth on variant calling sensitivity, we downsampled the BAMS of 10 samples at random by 50% and reran the identical somatic variant calling pipeline.

### Purity, ploidy and copy number calling

Copy number calling and determination of sample purity were performed using PURPLE (PURity & PLoidy Estimator), which combines B-allele frequency, read depth and structural variants to estimate the purity of a tumour sample and determine the copy number and minor allele ploidy for every base in the genome. The purity and ploidy estimates and copy number profile obtained from PURPLE were validated on in silico simulated tumour purities, by DNA fluorescence in situ hybridization (FISH) and by comparison with an alternative tool (ASCAT^59^). ASCAT was run on GC-corrected data using default parameters except for gamma, which was set to 1 (which is recommended for massively parallel sequencing data). We implement a simple heuristic that determines if a WGD event has occurred: major allele ploidy > 1.5 on at least 50% of at least 11 autosomes as the number of duplicated autosomes per sample (that is, the number of autosomes which satisfy the above rule) follows a bimodal distribution with 95% of samples have either ≤6 or ≥15 autosomes duplicated.

### Sample selection for downstream analyses

Following copy number calling, samples were filtered out based on absence of somatic variants, purity <20%, and GC biases, yielding a high-quality dataset of 2,520 samples. Where multiple biopsies exist for a single patient, the highest purity sample was used for downstream analyses (resulting in 2,399 samples).

### Mutational signature analysis

Mutational signatures were determined by fitting SNV counts per 96 tri-nucleotide context to the 30 COSMIC signatures^26^ using the mutationalPatterns package^60^. Residuals were calculated as the sum of the absolute difference between observed and fitted across the 96 buckets. Signatures with <5% overall contribution to a sample or absolute fitted mutational load <300 variants were excluded from the summary plot.

### Germline predisposition variant calling

We searched for pathogenic germline variants (SNVs, indels and copy number alterations) in a broad list of 152 germline predisposition genes previously curated^61^, using GATK HaplotypeCaller^55^ output from each sample. For each variant identified, we assessed the genotype in the germline (HET or HOM), whether there was a second somatic hit in the tumour, and whether the wild type or the variant itself was lost by a copy number alteration. We observed that for the variants in many of the 152 predisposition genes that a loss of wild type in the tumour via LOH was lower than the average rate of LOH across the cohort and that fewer than 5% of observed variants had a second somatic hit in the same gene. Moreover, in many of these genes, the *ALT* variant was lost via LOH as frequently as the wild type, suggesting that a considerable portion of the 566 variants may be passengers. For our downstream analysis and driver catalogue, we therefore restricted our analysis to a more conservative ‘high confidence’ list including only the 25 cancer related genes in the ACMG secondary findings reporting guidelines (v.2.0)^62^, together with four curated genes (*CDKN2A*, *CHEK2*, *BAP1* and *ATM*), selected because these are the only additional genes from the larger list of 152 genes with a significantly increased proportion of called germline variants with loss of wild type in the tumour sample.

### Clonality and biallelic status of point mutations

The ploidy of each variant is calculated by adjusting the observed VAF by the purity and then multiplying by the local copy number to work out the absolute number of chromatids that contain the variant. We mark a mutation as biallelic (that is, no wild type remaining) if variant ploidy > local copy number − 0.5. For each variant, we also determine a probability that it is subclonal. This is achieved via a two-step process involving fitting the somatic ploidies for each sample into a set of clonal and subclonal peaks and calculating the probability that each individual variant belongs to each peak. Subclonal counts are calculated as the total density of the subclonal peaks for each sample. Subclonal driver counts are calculated as the sum across the driver catalogue of subclonal probability × driver likelihood.

### MSI status determination

To determine the MSI status, we used the method described by the MSIseq tool^63^ and counted the number of indels per million bases occurring in homopolymers of five or more bases or dinucleotide, trinucleotide and tetranucleotide sequences of repeat count four or more. MSIseq score of >4 were considered MSI.

### Significantly mutated driver genes

We used Ensembl^64^ v.89.37 as a basis for gene definitions and have taken the union of Entrez identifiable genes and protein-coding genes as our base panel (25,963 genes of which 20,083 genes are protein coding). Pan-cancer and at an individual cancer level we tested the normalized nonsynonymous (dN) to synonymous substitution (dS) rate (that is, dN/dS) using dNdScv^24^ against a null hypothesis that dN/dS = 1 for each variant subtype. To identify significantly mutated genes in our cohort, we used a strict significance cut-off value of *q* < 0.01.

To search for significantly amplified and deleted genes, we first calculated the minimum exonic copy number per gene. For amplifications, we searched for all the genes with high-level amplifications only (defined as minimum exonic copy number >3 × sample ploidy). For deletions, we searched for all the genes in each sample with either full or partial homozygous gene deletions (defined as minimum exonic copy number < 0.5) excluding the Y chromosome. We then searched separately for amplifications and deletions, on a per-chromosome basis, for the most significant focal peaks, using an iterative GISTIC-like peel off method^65^. Most of the deletion peaks resolve clearly to a single target gene, which reflects the fact that homozygous deletions are highly focal, but for amplifications this is not the case and most of our peaks have ten or more candidates. We therefore annotated the peaks, to choose a single putative target gene using an objective set of automated curation rules. Finally, filtering was applied to yield highly significant deletions and amplifications.

Homozygous deletions were also annotated as common fragile sites based on their genomic characteristics, including a strong enrichment in long genes (>500,000 bases) and a high rate (>30%) of deletions between 20 kb and 1 Mb^27^.

### Somatic driver catalogue construction

We created a catalogue of mutations in known cancer genes in our cohort across all variant types on a per-patient basis. This was done in a similar incremental manner to that previously described^32^ (N. Lopez, personal communication) in which we first calculated the number of genes with putative driver mutations in a broad panel of known and significantly mutated genes across the full cohort, and then assigned the candidate driver mutations for each gene to individual patients by ranking and prioritizing each of the observed variants. Key points of difference in this study were both the prioritization mechanism used and our choice to ascribe each mutation a probability of being a driver rather than a binary cut-off based on absolute ranking.

The four steps to create the catalogue are as follows. (1) Create a panel of candidate genes for point mutations using significantly mutated genes and known cancer genes using the union of Martincorena significantly mutated genes^24^ (filtered to significance of *q* < 0.01), HMF significantly mutated genes (*q* < 0.01) at global level or at cancer type level and COSMIC curated genes^26^ (v.83). (2) Determine TSG or oncogene status of each significantly mutated gene using a logistic regression classification model (trained using COSMIC annotation). (3) Add mutations from all variant classes to the catalogue when meeting any of the following criteria: (i) all missense and in-frame indels for panel oncogenes; (ii) all non-synonymous and essential splice point mutations for TSGs; (iii) all high-level amplifications for significantly amplified target genes and panel oncogenes; (iv) all homozygous deletions for significantly deleted target genes and panel TSGs; (v) all known or promiscuous in-frame gene fusions; and (vi) recurrent *TERT* promoter mutations. (4) Calculate a per-sample likelihood score (between 0 and 1) for each mutation in the catalogue as a potential driver event, to ensure that only likely pathogenic and excess mutations (based on dN/dS) are used to determine the number of drivers. All putative driver mutation counts reported at a per-cancer type or sample level refer to the sum of driver likelihoods for that cancer type or sample.

### Clinical associations and actionability analysis

To determine clinical associations and potential actionability of the variants observed in each sample, we compared all variants with three external clinical annotation databases (OncoKB^40^, CGI^41^ and CIViC^39^) that were mapped to a common data model as defined by https://civicdb.org/help/evidence/evidence-levels. Here, we considered only A and B level variants. This classification of potential actionable events can also be mapped to the recently proposed ESMO Scale for Clinical Actionability of molecular Targets (ESCAT)^66^ as follows: ESCAT I-A+B (for A on-label) and I-C (for A off-label) and ESCAT II-A+B (for B on-label) and III-A (for B off-label). For each candidate actionable mutation, it was also determined to be either on-label (that is, evidence supports treatment in that specific cancer type) or off-label (evidence exists in another cancer type). To do this, we annotated both the patient cancer types and the database cancer types with relevant DOIDs, using the disease ontology database^67^. For each candidate actionable mutation in each sample, we aggregated all the mapped evidence that was available supporting both on-label and off-label treatments at the A or B evidence level. Treatments that also had evidence supporting resistance based on other biomarkers in the sample at the same or higher evidence level were excluded as non-actionable. Samples classified as MSI in our driver catalogue were also mapped as actionable at level A evidence based on clinical annotation in the OncoKB database. For each sample, we reported the highest level of predicted actionability, ranked first by evidence level and then by on-label vs off-label.

### Reporting summary

Further information on research design is available in the Nature Research Reporting Summary linked to this paper.

## Online content

Any methods, additional references, Nature Research reporting summaries, source data, supplementary information, acknowledgements, peer review information; details of author contributions and competing interests; and statements of data and code availability are available at 10.1038/s41586-019-1689-y.

## Supplementary information

Supplementary Information .This file contains a detailed description of methods and validation results

Reporting Summary

Supplementary Figure 1.**Copy Number profile per cancer types**. Circos plots showing the proportion of samples with amplification and deletion events by genomic position per cancer type. The inner ring shows the % of tumours with homozygous deletion (red), LOH and significant loss (copy number < 0.6x sample ploidy - dark blue) and near copy neutral LOH (light blue). The outer ring shows the % of tumours with high level amplification (>3x sample ploidy - orange), moderate amplification (>2x sample ploidy - dark green) and low level amplification (>1.4x amplification - light green). Scales on both rings are 0-100% and inverted for the inner ring. The most frequently observed high level gene amplifications (black text) and homozygous deletions (red text) are labelled

Supplementary Figure 2.**Coding mutation profiles by tumour suppressor driver gene**. Location and driver classification of all coding mutations (SNVs and indels) in tumour suppressor genes (TSG) in the driver catalogue. The lollipops on the chart show the location (coding sequence coordinates) and count of mutations for all candidate drivers. The height of lollipop represents the total count of each individual variant in the cohort (log scale). The height of the solid line represents the sum of driver likelihoods for that variant, i.e. the proportion that are expected to be drivers. (Partially) dotted lines hence indicate variants for which driver role is uncertain. Variants are unshaded if all instances of that variant are monoallelic single hits with no LOH. The right column chart shows the estimated number of drivers (calculated as the sum of driver likelihoods) and passenger variants in each gene by cancer type

Supplementary Figure 3.**Coding mutation profiles by oncogene driver gene**. Location and driver classification of all coding mutations (SNVs and indels) in oncogenes (a) and tumour suppressor genes (TSG) (b) in the driver catalogue. The lollipops on the chart show the location (coding sequence coordinates) and count of mutations for all candidate drivers. The height of lollipop represents the total count of each individual variant in the cohort (log scale). The height of the solid line represents the sum of driver likelihoods for that variant, i.e. the proportion that are expected to be drivers. (Partially) dotted lines hence indicate variants for which driver role is uncertain. The right column chart shows the stimated number of drivers (calculated as the sum of driver likelihoods) and passenger variants in each gene by cancer type

Supplementary Table 1 .Overview of contributing organizations and local principal investigators

Supplementary Table 2 .Overview of cohort and sample characteristics

Supplementary Table 3 .Pan-cancer (n = 2399 independent patients) and cancer typespecific (n per tumor type category is provided in Fig. 1) dNdScv results (see Supplementary Information Detailed Methods for statistical details)

Supplementary Table 4 .Recurring amplifications (a) and deletions (b) and associated target genes

Supplementary Table 5 .Somatic driver catalogue

Supplementary Table 6 .Germline driver catalogue

Supplementary Table 7 .Gene Fusions

Supplementary Table 8 .Overview of patients with multiple biopsies

Supplementary Table 9 .Actionable mutations

## Data Availability

All data described in this study are freely available for academic use from the Hartwig Medical Foundation through standardized procedures and request forms that can be found at https://www.hartwigmedicalfoundation.nl/en/appyling-for-data/. Available data include germline and tumour raw sequencing data (BAM files, including non-mapped reads), annotated somatic and germline variants (VCF files with annotated SNV and indels, and pipeline output files for purity and ploidy status as well as copy number alteration and structural variants) and clinical data. Examples of output files can be found at https://resources.hartwigmedicalfoundation.nl. In brief, a data request can be initiated by filling out the standard form in which intended use of the requested data is motivated. First, an advice on scientific feasibility and validity is obtained from experts in the field that is used as input by an independent data access board who also evaluates if the intended use of the data is compatible with the consent given by the patients and if there would be any applicable legal or ethical constraints. Upon formal approval by the data access board, a standard license agreement that does not have any restrictions regarding intellectual property resulting from the data analysis needs to be signed by an official organization representative before access to the data are granted. After approval, access to data is provided under a license model, with the only main restriction that the data can only be used for the research detailed in the original request. Raw data files will be made available through a dedicated download portal with two-factor authentication. Non-privacy sensitive somatic variants can also be browsed and explored through an open access web-based interface which can be accessed at http://database.hartwigmedicalfoundation.nl/.

## Code availability

All code used is open source and available from third parties or developed by Hartwig Medical Foundation (https://github.com/hartwigmedical/). A full list of tools and versions used including links to the source code is provided in the Supplementary Information.
