# Supplementary material for: Pan-cancer whole-genome analyses of metastatic solid tumours
Source: Nature. 2019 Oct 23;575(7781):210–6. doi: 10.1038/s41586-019-1689-y (PMC6872491; doi:10.1038/s41586-019-1689-y)
Supplement: Supplementary file 2 — Reporting Summary [file 41586_2019_1689_MOESM2_ESM.pdf]

nature research

Corresponding author(s): Priestley and Cuppen

Reporting Summary

Nature Research wishes to improve the reproducibility of the work that we publish. This form provides structure for consistency and transparency in reporting. For further information on Nature Research policies, see Authors & Referees and the Editorial Policy Checklist.

Statistical parameters

When statistical analyses are reported, confirm that the following items are present in the relevant location (e.g. figure legend, table legend, main text, or Methods section).

n/a	Confirmed	
☐	☒	The exact sample size (*n*) for each experimental group/condition, given as a discrete number and unit of measurement
☐	☒	An indication of whether measurements were taken from distinct samples or whether the same sample was measured repeatedly
☐	☒	The statistical test(s) used AND whether they are one- or two-sided<br>*Only common tests should be described solely by name; describe more complex techniques in the Methods section.*
☐	☒	A description of all covariates tested
☐	☒	A description of any assumptions or corrections, such as tests of normality and adjustment for multiple comparisons
☐	☒	A full description of the statistics including central tendency (e.g. means) or other basic estimates (e.g. regression coefficient) AND variation (e.g. standard deviation) or associated estimates of uncertainty (e.g. confidence intervals)
☒	☐	For null hypothesis testing, the test statistic (e.g. *F*, *t*, *r*) with confidence intervals, effect sizes, degrees of freedom and *P* value noted<br>*Give P values as exact values whenever suitable.*
☒	☐	For Bayesian analysis, information on the choice of priors and Markov chain Monte Carlo settings
☒	☐	For hierarchical and complex designs, identification of the appropriate level for tests and full reporting of outcomes
☐	☒	Estimates of effect sizes (e.g. Cohen's *d*, Pearson's *r*), indicating how they were calculated
☐	☒	Clearly defined error bars<br>*State explicitly what error bars represent (e.g. SD, SE, CI)*

Our web collection on statistics for biologists may be useful.

Software and code

Policy information about availability of computer code

Data collection	No software was used for data collection
Data analysis	All analyses are based on open source software, which is available from third parties or developed by Hartwig Medical Foundation and available on GitHub (https://github.com/hartwigmedical/). The table below lists all external and internally developed software/tools, versions used and public links to the source code.<br><br>External software/tools:<br>bcl2fastq 2.17 to 2.20 http://sapac.support.illumina.com/downloads/bcl2fastq-conversion-software-v2-20.html<br>BWA-mem 0.7.5a https://github.com/lh3/bwa<br>Sambamba 0.6.5 https://github.com/biod/sambamba/releases/tag/v0.6.5<br>Picard 1.141 https://broadinstitute.github.io/picard/<br>GATK 3.4.46 https://software.broadinstitute.org/gatk/download/auth?package=GATK-archive&version=3.4-46-gbc02625<br>Strelka 1.0.14 https://github.com/Illumina/strelka<br>mutationalPatterns 1.4.3 https://bioc.ism.ac.jp/packages/3.6/bioc/html/MutationalPatterns.html<br>Manta 1.0.3 https://github.com/Illumina/manta<br>STAR-fusion ?? https://github.com/STAR-Fusion/STAR-Fusion/releases<br>Bioconductor CopyNumber package 1.24.0 http://bioconductor.org/packages/release/bioc/html/copynumber.html<br>ASCAT 2.52 https://github.com/Crick-CancerGenomics/ascat<br>dNdScv 0.1.0 https://github.com/im3sanger/dndscv/releases/tag/0.1.0

Circos 0.69.6 http://circos.ca/distribution/circos-0.69-6.tgz
samtools 1.2 https://github.com/samtools/samtools/releases/tag/1.2
snpeff 4.3s https://sourceforge.net/projects/snpeff/files/snpEff_v4_3s_core.zip/download
vcftools 0.1.14 https://vcftools.github.io/index.html
bcftools 1.9 https://github.com/samtools/bcftools/releases/download/1.9/bcftools-1.9.tar.bz2

HMF internal software/tools:
Strelka_post_process 1.4 https://github.com/hartwigmedical/hmftools/releases/tag/strelka-post-process-v1-4
HMF pipeline v3.0 https://github.com/hartwigmedical/pipeline/releases/tag/v3.0
SAGE 1.1 https://github.com/hartwigmedical/hmftools/releases/tag/sage%E2%80%94v1-1
BPI 1.5 https://github.com/hartwigmedical/hmftools/releases/tag/bpi-v1-5
PURPLE 2.10 https://github.com/hartwigmedical/hmftools/releases/tag/purple-v2-10
Amber 1.5 https://github.com/hartwigmedical/hmftools/releases/tag/amber-v1-5
Cobalt 1.4 https://github.com/hartwigmedical/hmftools/releases/tag/cobalt-v1-4
healthchecker 2.1 https://github.com/hartwigmedical/hmftools/tree/master/health-checker
R analysis suite 1.3 https://github.com/hartwigmedical/scripts/releases/tag/pancancerpaper-v1-3

For manuscripts utilizing custom algorithms or software that are central to the research but not yet described in published literature, software must be made available to editors/reviewers upon request. We strongly encourage code deposition in a community repository (e.g. GitHub). See the Nature Research guidelines for submitting code & software for further information.

Data

Policy information about availability of data

All manuscripts must include a data availability statement. This statement should provide the following information, where applicable:

- Accession codes, unique identifiers, or web links for publicly available datasets
- A list of figures that have associated raw data
- A description of any restrictions on data availability

All data described in this study is freely available for academic use from the Hartwig Medical Foundation through standardized procedures and request forms which can be found at https://www.hartwigmedicalfoundation.nl/en/appyling-for-data/.

Available data includes germline and tumor raw sequencing data (BAM files, including non-mapped reads), annotated somatic and germline variants (VCF files with annotated SNV and indels, and pipeline output files for purity and ploidy status as well as copy number alteration and structural variants) and clinical data. Examples of output files can be found at https://resources.hartwigmedicalfoundation.nl. Briefly, a data request can be initiated by filling out the standard form in which intended use of the requested data is motivated. First, an advice on scientific feasibility and validity is obtained from experts in the field which is used as input by an independent Data Access Board who also evaluates if the intended use of the data is compatible with the consent given by the patients and if there would be any applicable legal or ethical constraints. Upon formal approval by the Data Access Board, a standard license agreement which does not have any restrictions regarding Intellectual Property resulting from the data analysis needs to be signed by an official organisation representative before access to the data is granted. After approval, access to data is provided under a license model, with the only main restriction that the data can only be used for the research detailed in the original request. Raw data files will be made available through a dedicated download portal with two-factor authentication.

Non-privacy sensitive somatic variants can also be browsed and explored through an open access web-based interface which can be accessed at http://database.hartwigmedicalfoundation.nl/.

Field-specific reporting

Please select the best fit for your research. If you are not sure, read the appropriate sections before making your selection.

☒ Life sciences ☐ Behavioural & social sciences ☐ Ecological, evolutionary & environmental sciences

For a reference copy of the document with all sections, see nature.com/authors/policies/ReportingSummary-flat.pdf

Life sciences study design

All studies must disclose on these points even when the disclosure is negative.

Sample size	The metastatic tumor sample cohort described in the paper consists of 2520 independent samples from 2399 patients (including 121 repeat biopsies) collected in 41 hospitals (academic, teaching and general hospitals). No sample size calculations were performed as the main aim of the study was to build up a resource
Data exclusions	Samples that failed predefined QC criteria or with a tumor purity below 20% were excluded from all analyses and not included in the 2520 sample cohort (see Extended Data Fig 1). The tumor purity threshold was defined after bioinformatic tool optimization and simulations with titration series of reference samples and validation experiments on selected cohort samples.
Replication	Independent repeat processing of raw data of the same sample results in the same variant call data
Randomization	Not applicable as the primary goal of this study was to create a resource. The study and analyses do not include any experimental manipulation and only involved the collection of tissue and blood material, the generation of whole genome sequencing data and the collection of clinical data from medical records.

Blinding	Not applicable as the primary goal of this study was to create a resource. The study and analyses do not include any experimental manipulation and only involved the collection of tissue and blood material, the generation of whole genome sequencing data and the collection of clinical data from medical records.

Reporting for specific materials, systems and methods

Materials & experimental systems

n/a	Involved in the study	
☐	☒	Unique biological materials
☒	☐	Antibodies
☒	☐	Eukaryotic cell lines
☒	☐	Palaeontology
☒	☐	Animals and other organisms
☐	☒	Human research participants

Methods

n/a	Involved in the study	
☒	☐	ChIP-seq
☒	☐	Flow cytometry
☒	☐	MRI-based neuroimaging

Unique biological materials

Policy information about availability of materials

Obtaining unique materials	Tumor biopsies are collected as part of two clinical studies and remaining material is deposited in local biobanks as described in the methods. Because of the nature of the material (very small amount), broad accessibility is not possible.

Human research participants

Policy information about studies involving human research participants

Population characteristics	All patient included where diagnosed with metastatic disease and considered fit enough to undergo an invasive core-needle biopsy and planned to start treatment. The median age is 63 years (range 18 - 89). The cohort includes 1221 female and 1178 male subjects. Age and gender information of each patient is included in Supplementary Table 2. All patients were seen in hospitals in the Netherlands, including academic, teaching and general hospitals
Recruitment	Metastatic cancer patients were asked to participate in the studies in any of the 41 participating hospitals. Recruitment involved hundreds of medical specialists and research nurses which minimizes self-selection biases. Recruitment was independent on tumor type. An important requirement for participation was the ability to safely undergo a tumor biopsy. Health conditions and lesion site related risk could therefore have resulted in exclusion of patients.
