# Supplementary material for: Pan-cancer whole-genome analyses of metastatic solid tumours
Source: Nature. 2019 Oct 23;575(7781):210–6. doi: 10.1038/s41586-019-1689-y (PMC6872491; doi:10.1038/s41586-019-1689-y)

Biliary

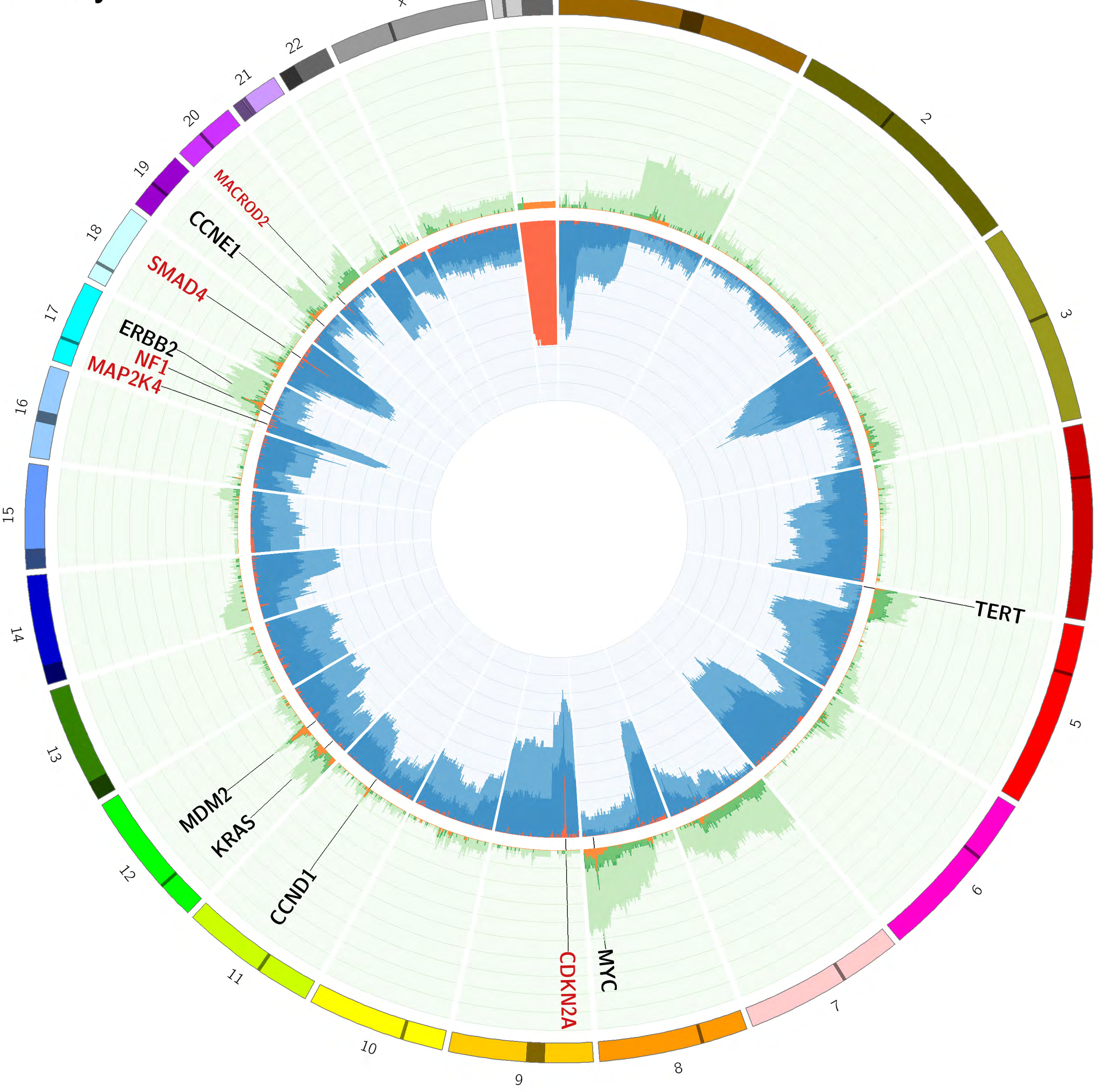

Bone/Soft tissue

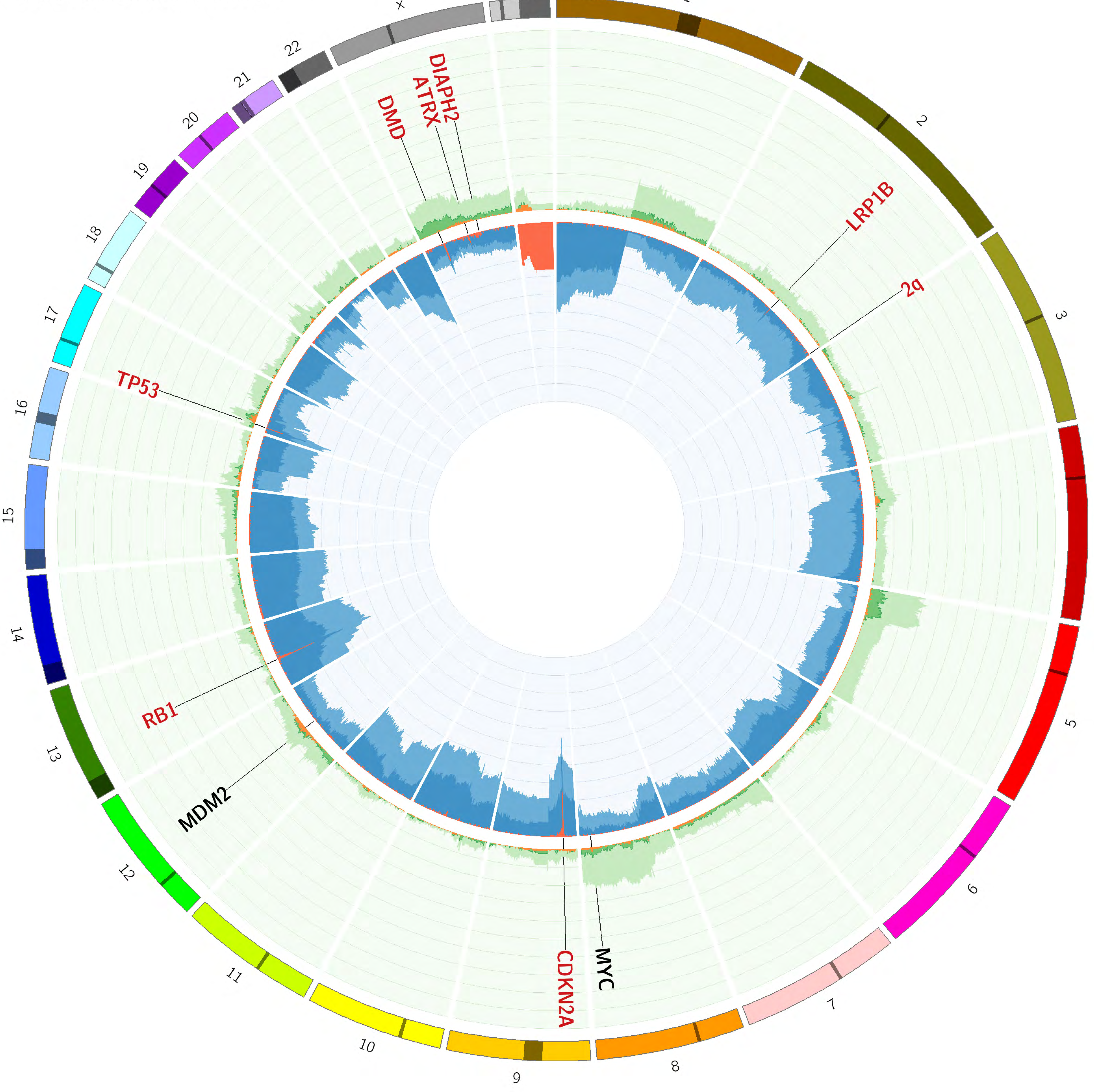

Breast

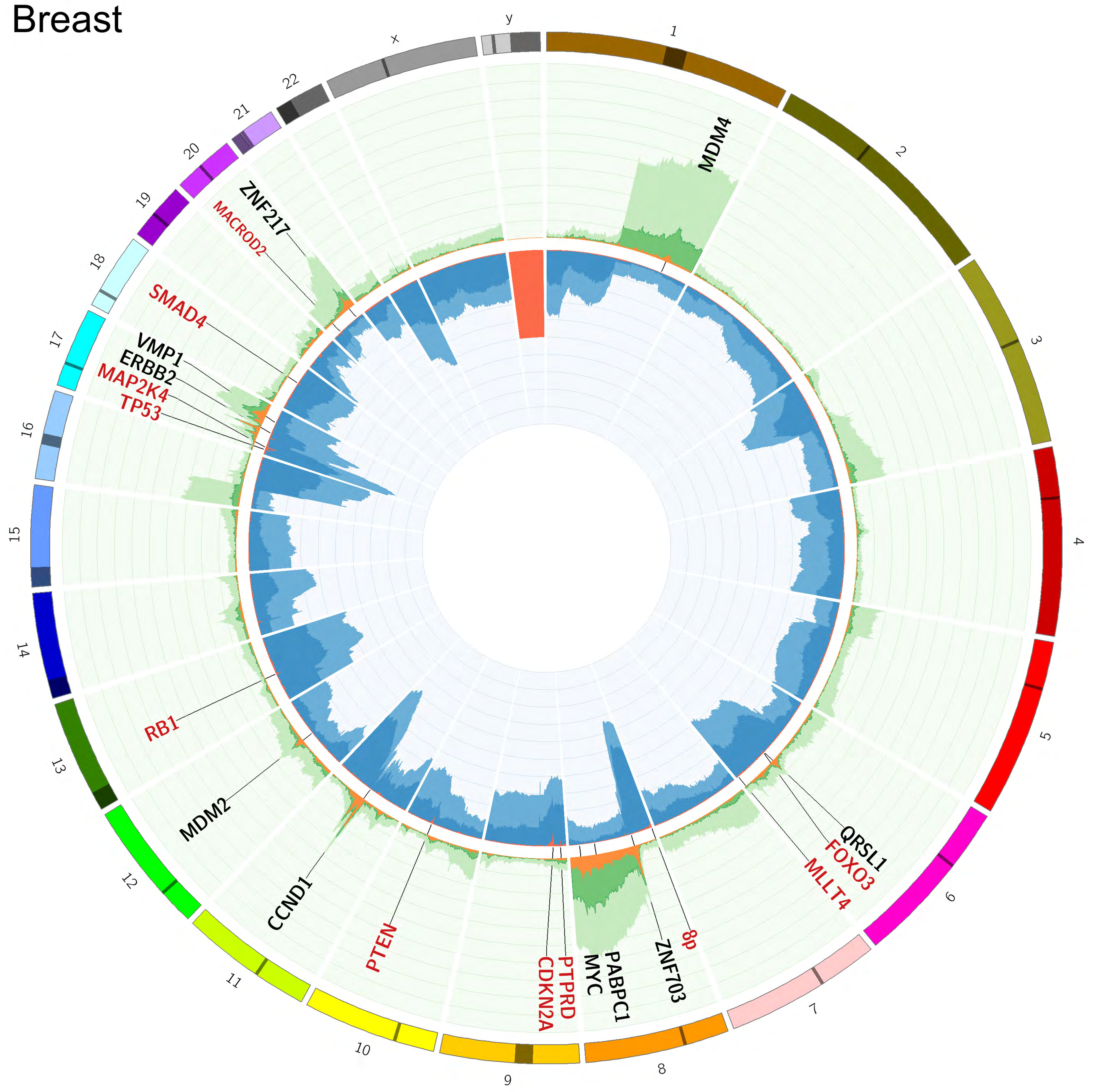

MDM4
ZNF217
MACROD2
SMAD4
VMP1
ERBB2
MAP2K4
TP53
RB1
MDM2
CCND1
PTEN
CDKN2A
PTPRD
MYC
PABPC1
ZNF703
8p
MLLT4
FOXO3
QRSL1

CNS

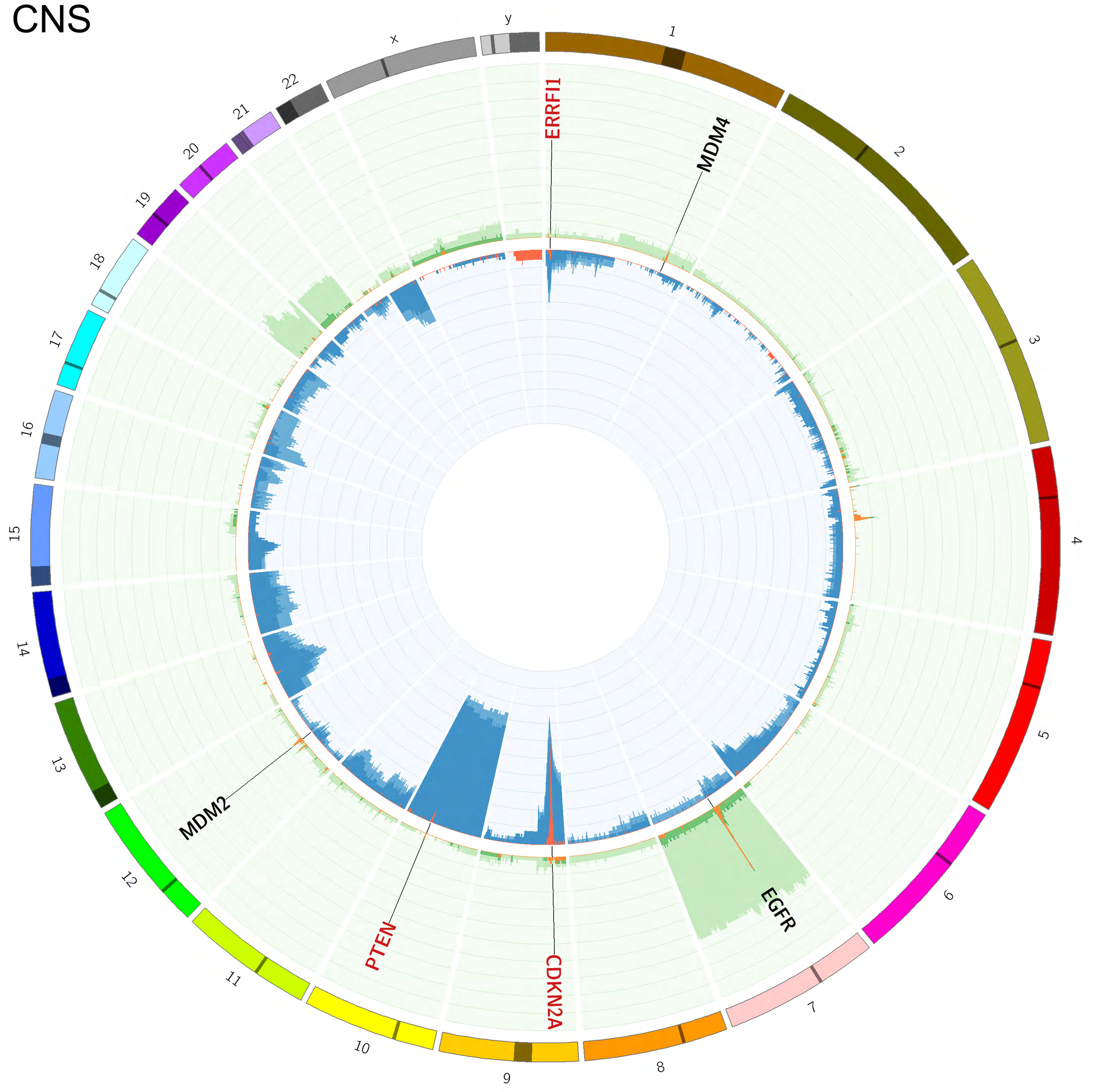

ERRFI1
MDM4
MDM2
PTEN
CDKN2A
EGFR

Colon/Rectum

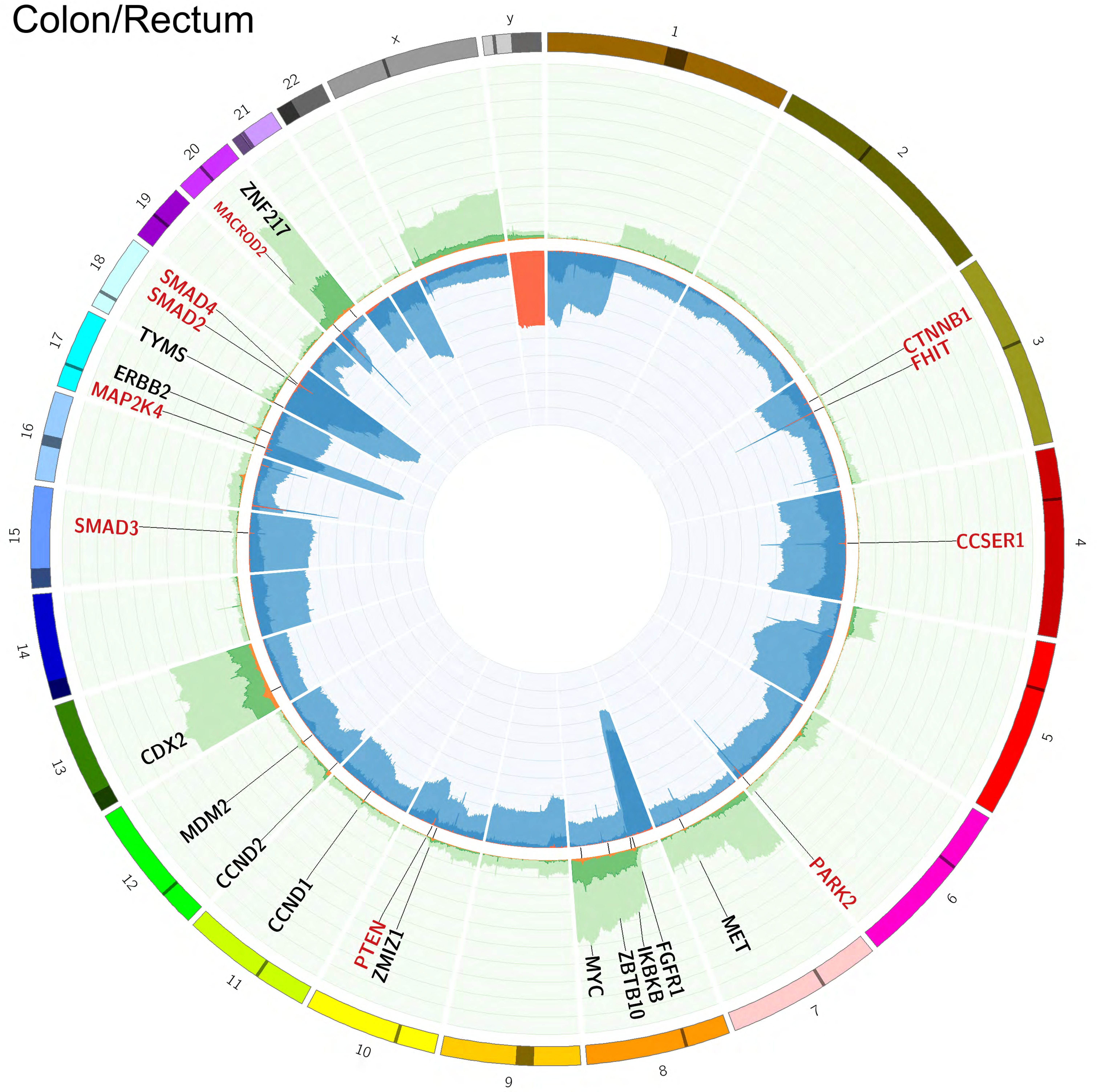

CUP

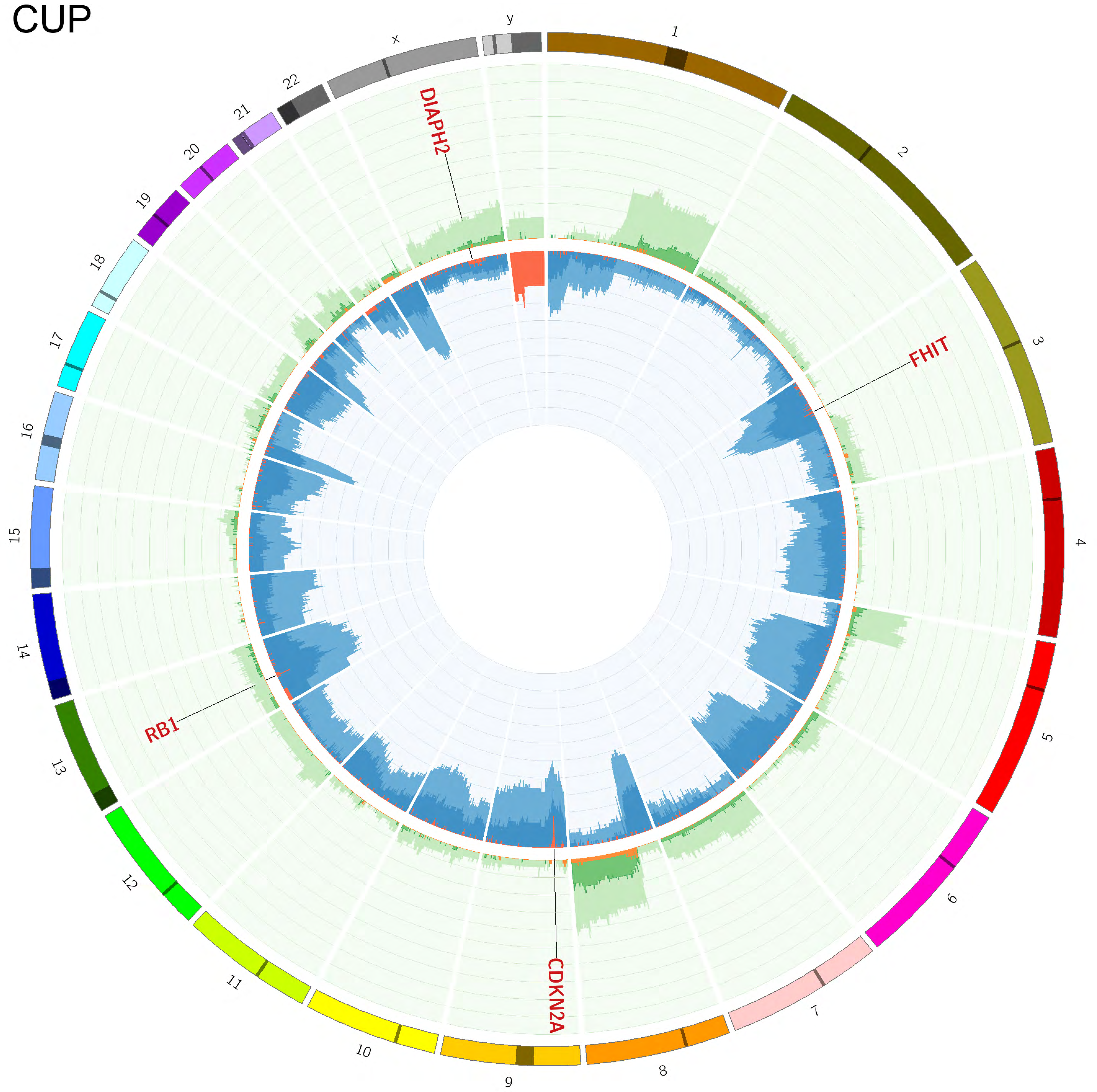

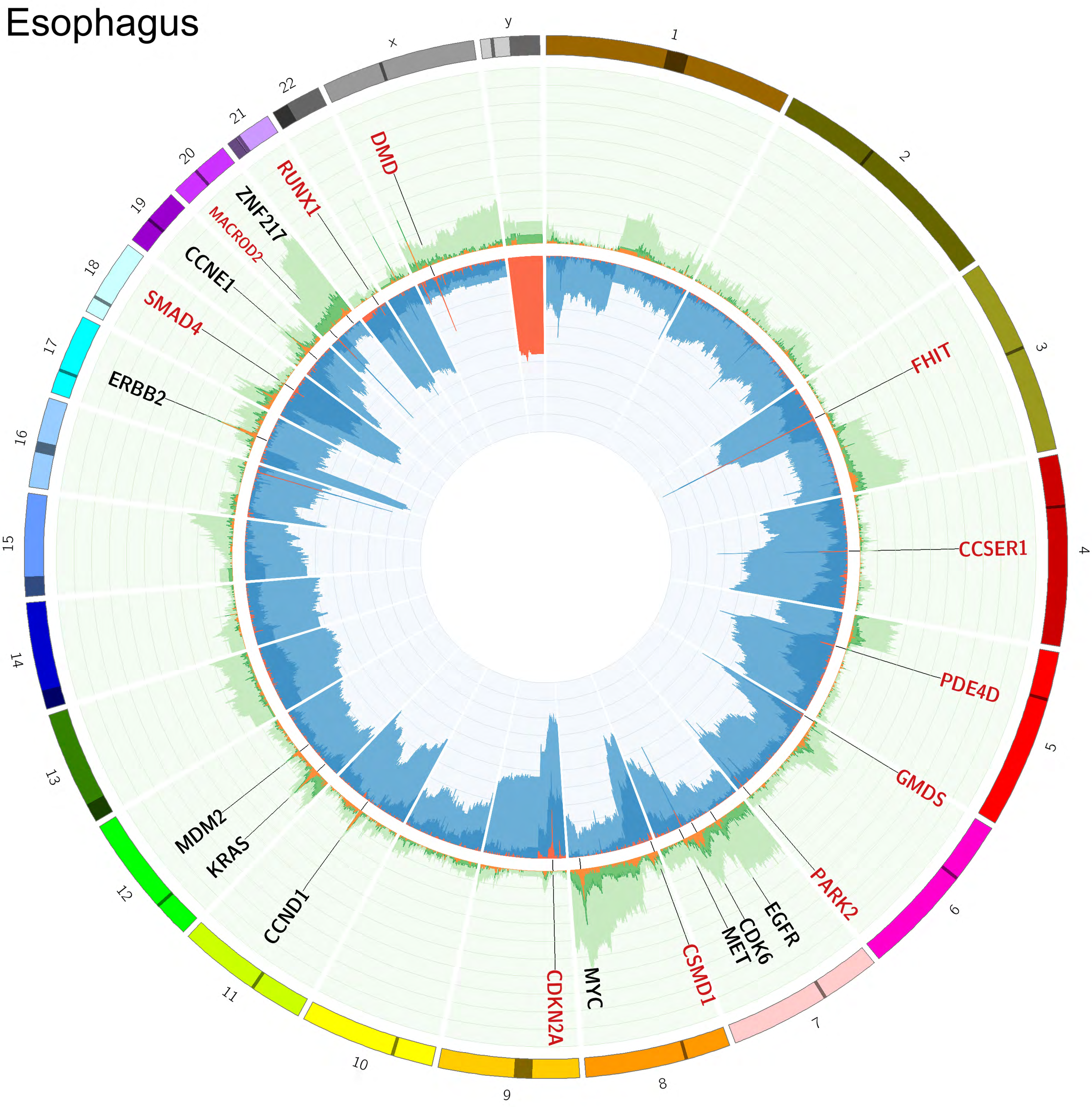

Esophagus
DMD
RUNX1
ZNF217
MACROD2
CCNE1
SMAD4
ERBB2
FHIT
CCSER1
PDE4D
GMDS
PARK2
EGFR
CDK6
MET
CSMD1
MYC
CDKN2A
CCND1
KRAS
MDM2
1
2
3
4
5
6
7
8
9
10
11
12
13
14
15
16
17
18
19
20
21
22
x
y

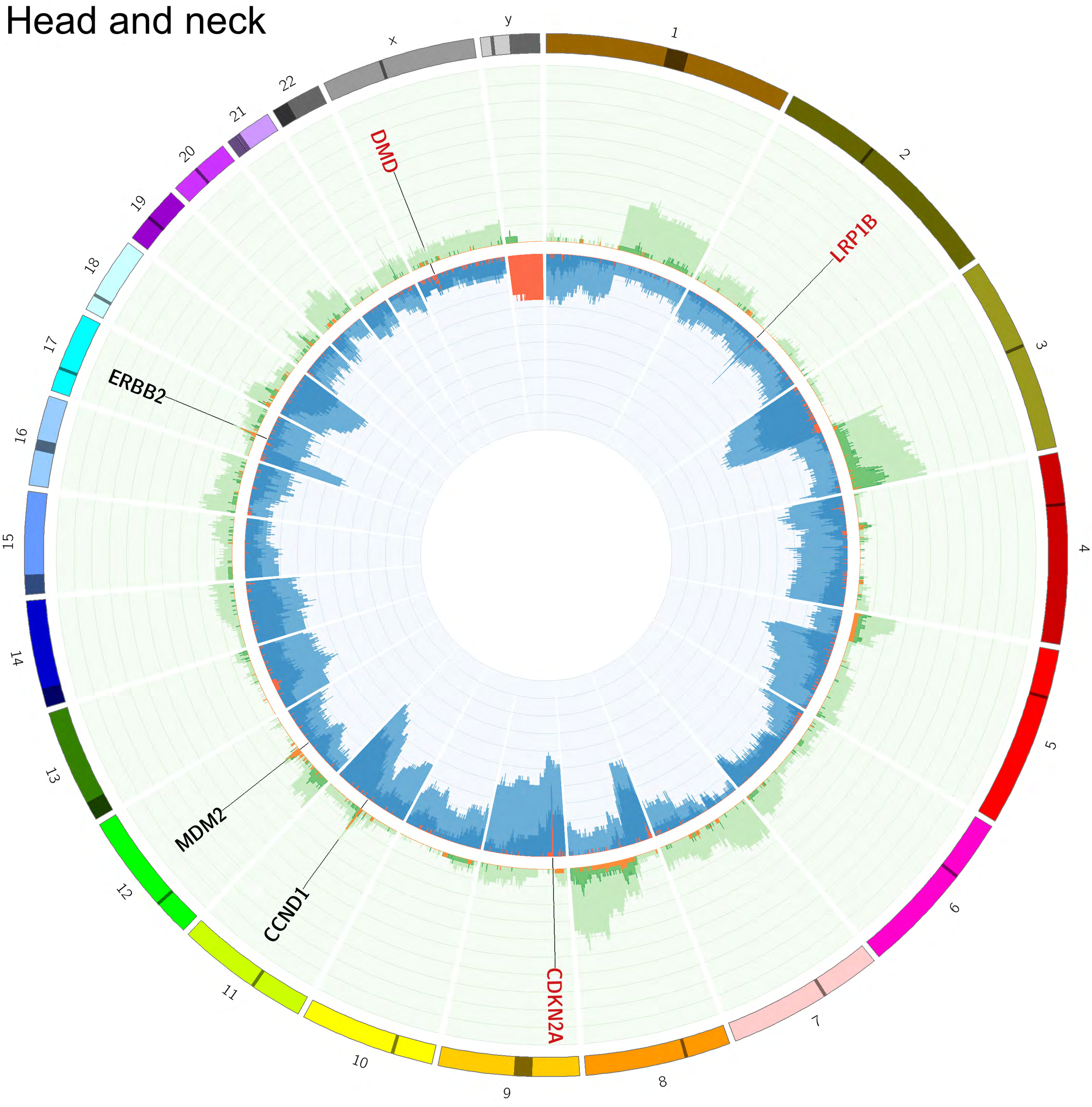

Head and neck
DMD
LRP1B
ERBB2
MDM2
CCND1
CDKN2A
1
2
3
4
5
6
7
8
9
10
11
12
13
14
15
16
17
18
19
20
21
22
x
y

Kidney

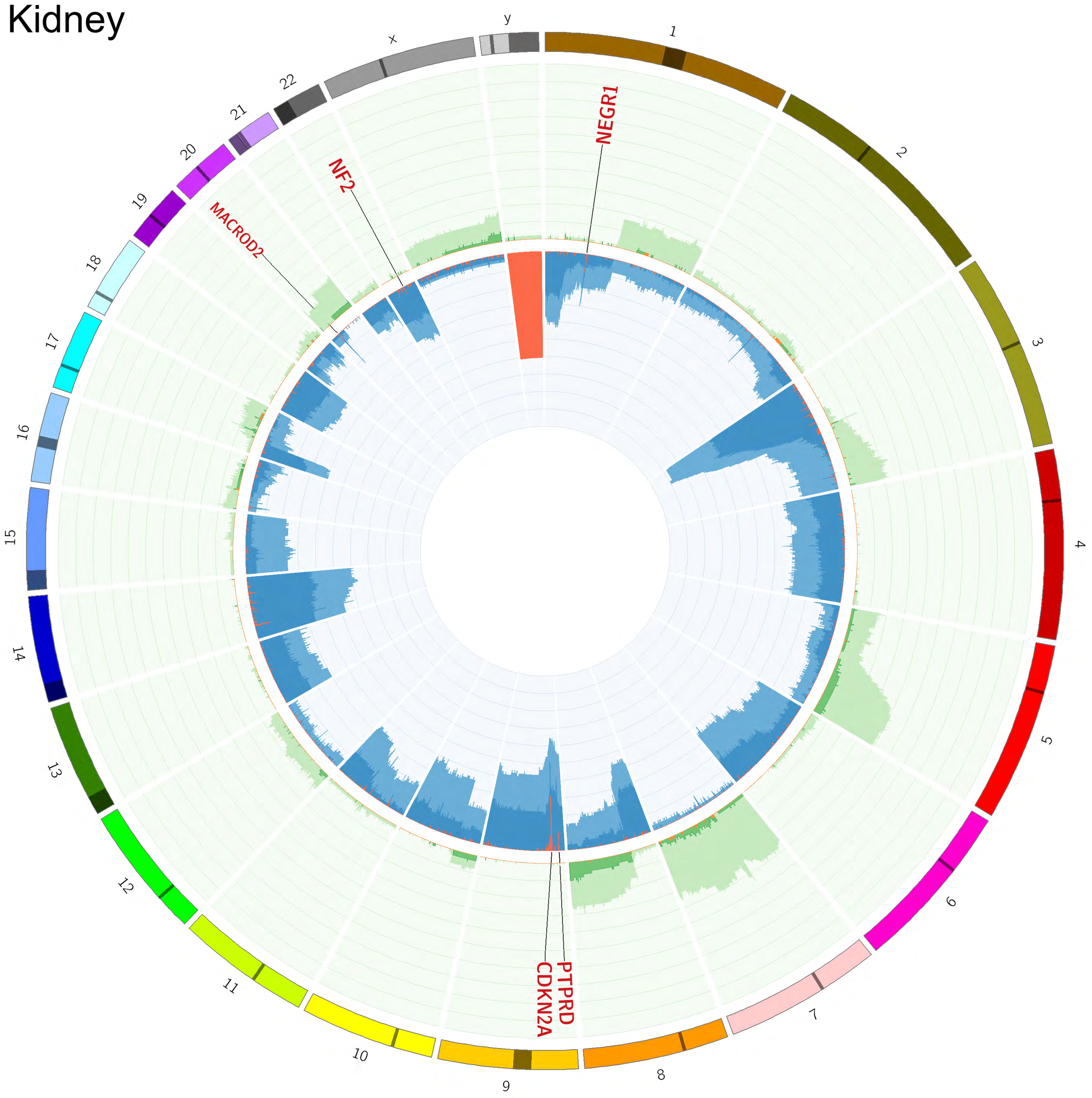

Liver

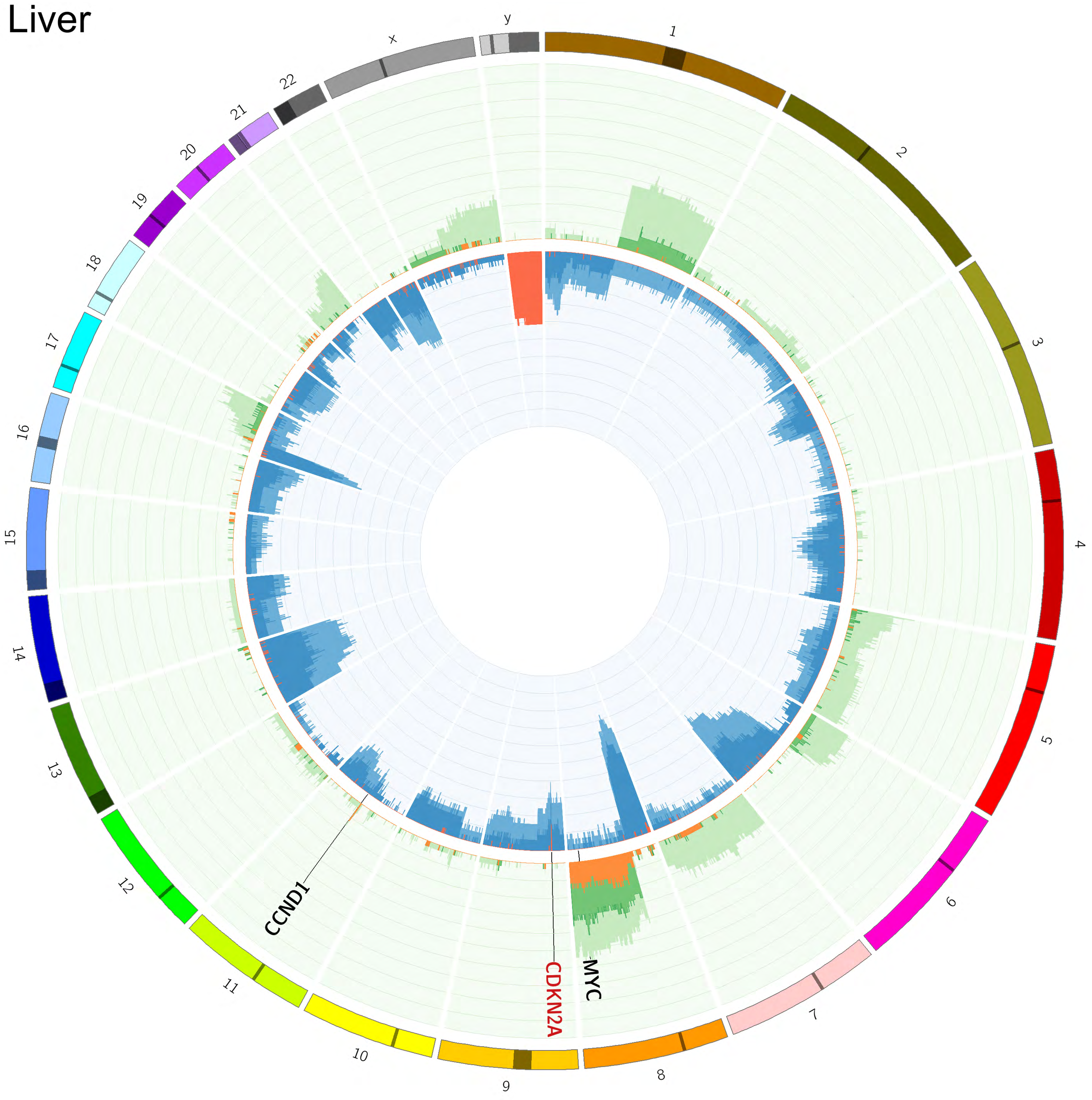

Lung

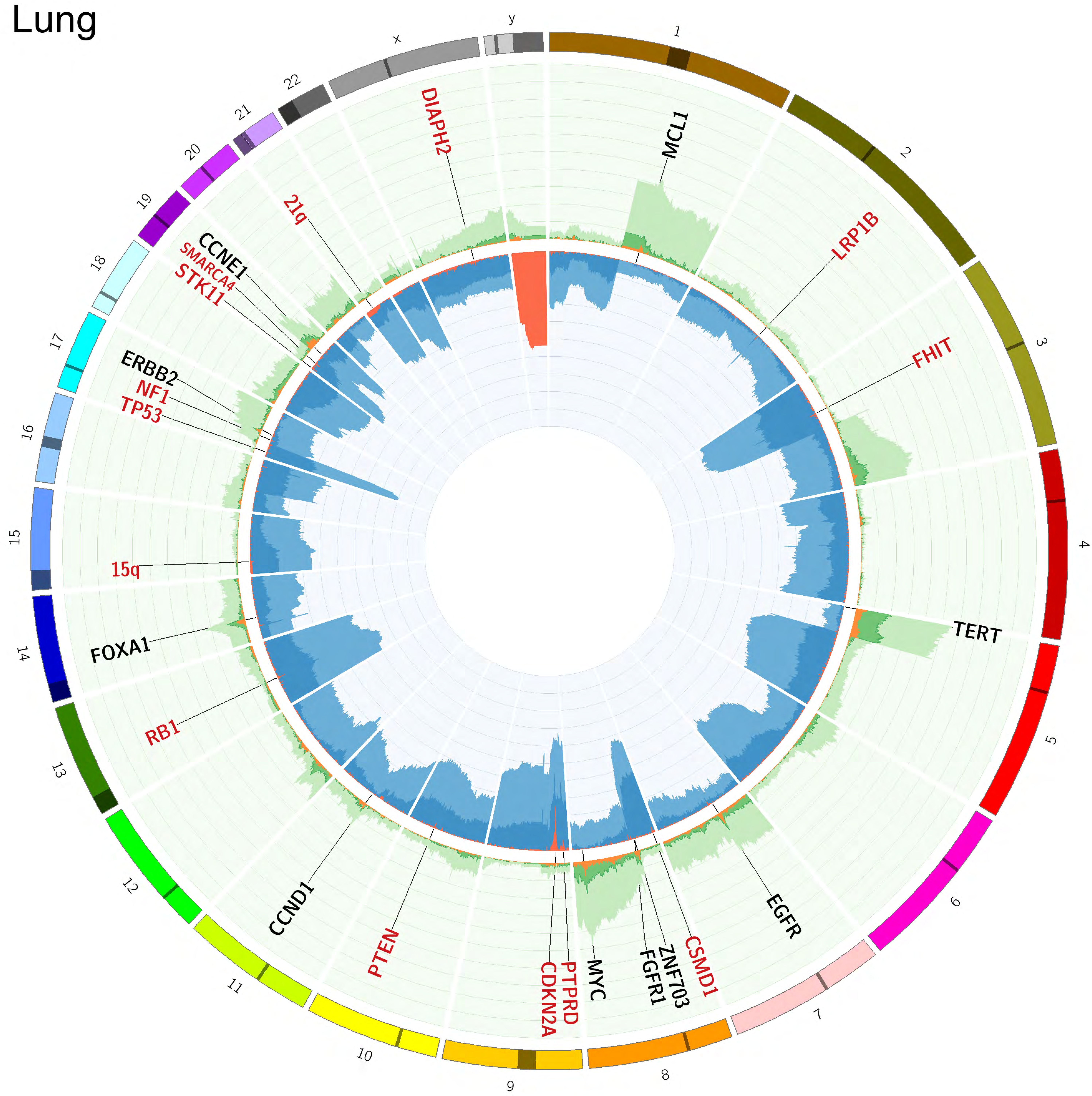

Mesothelioma

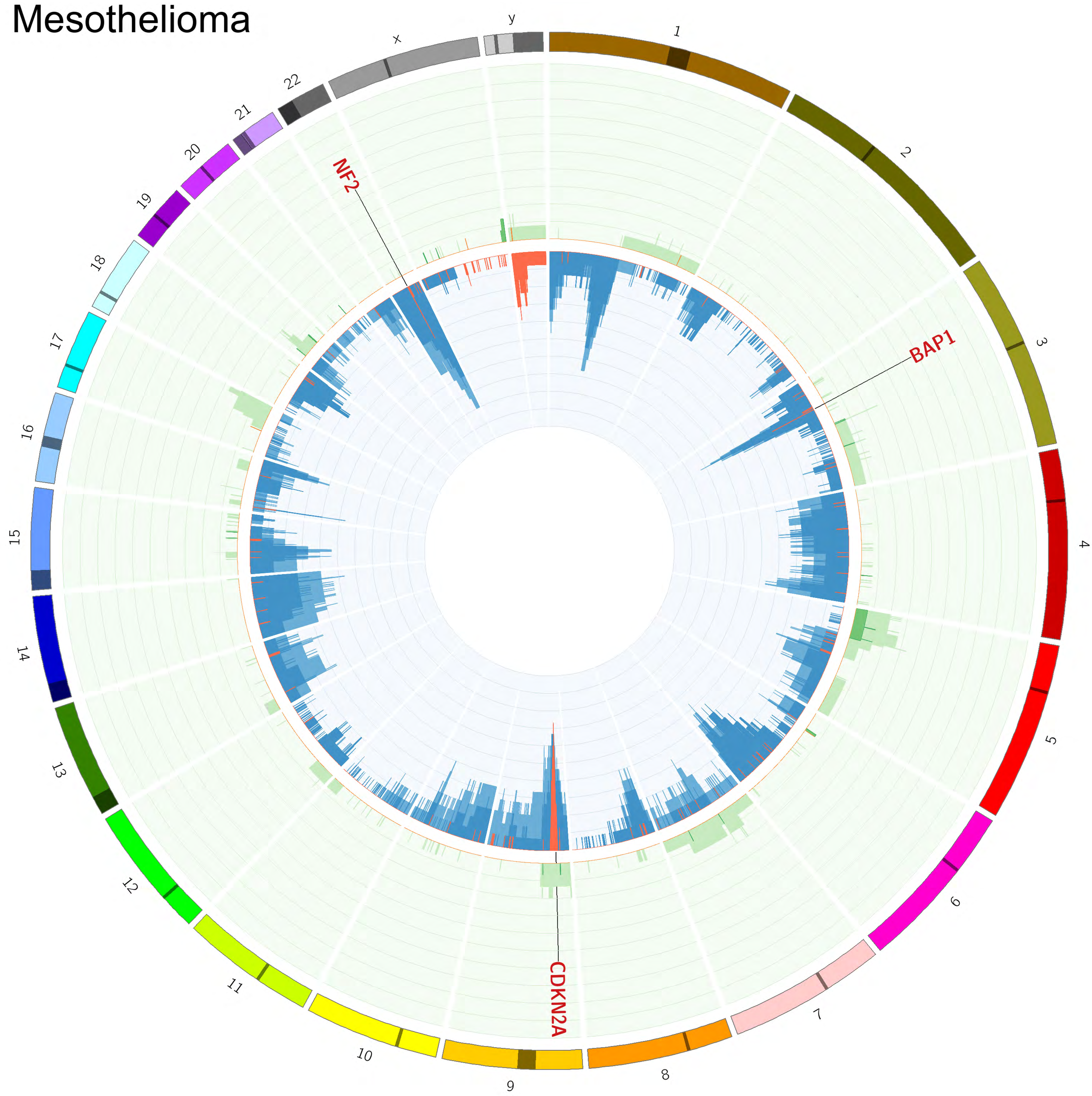

NET

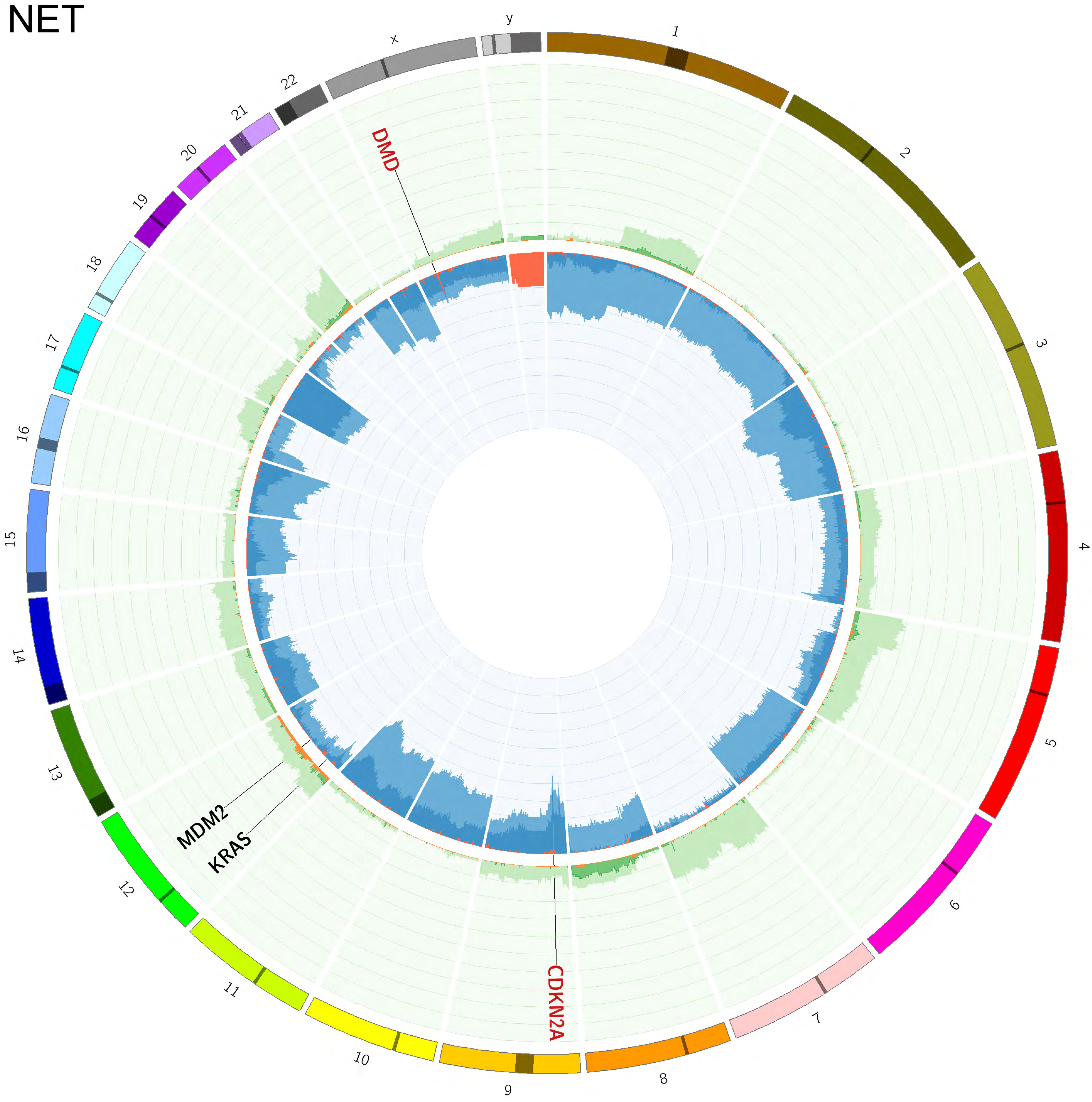
1
2
3
4
5
6
7
8
9
10
11
12
13
14
15
16
17
18
19
20
21
22
x
y
DMD
MDM2
KRAS
CDKN2A

Ovary

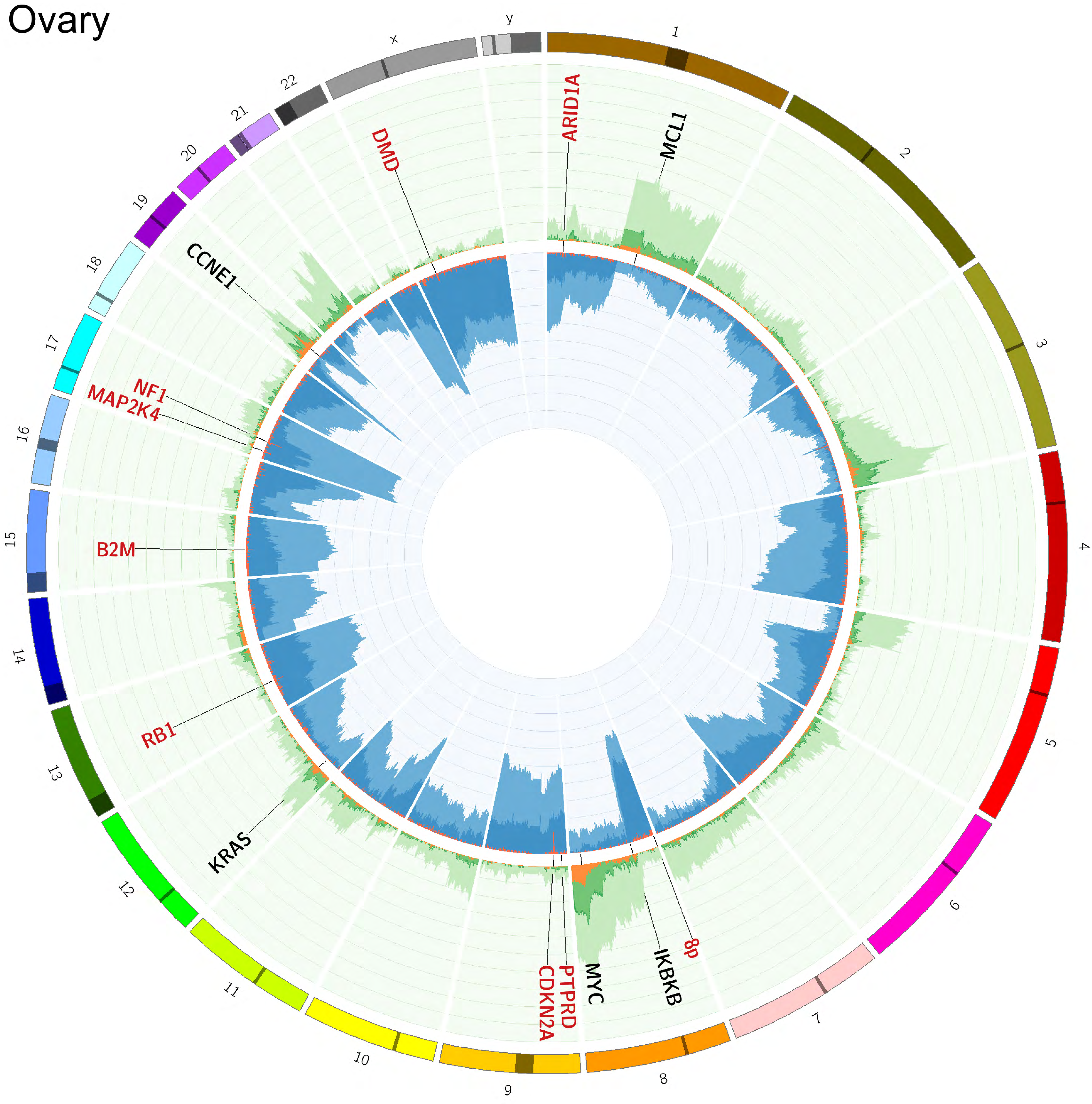
1
2
3
4
5
6
7
8
9
10
11
12
13
14
15
16
17
18
19
20
21
22
x
y
ARID1A
MCL1
DMD
CCNE1
NF1
MAP2K4
B2M
RB1
KRAS
CDKN2A
PTPRD
MYC
IKBKB
8p

Pancreas

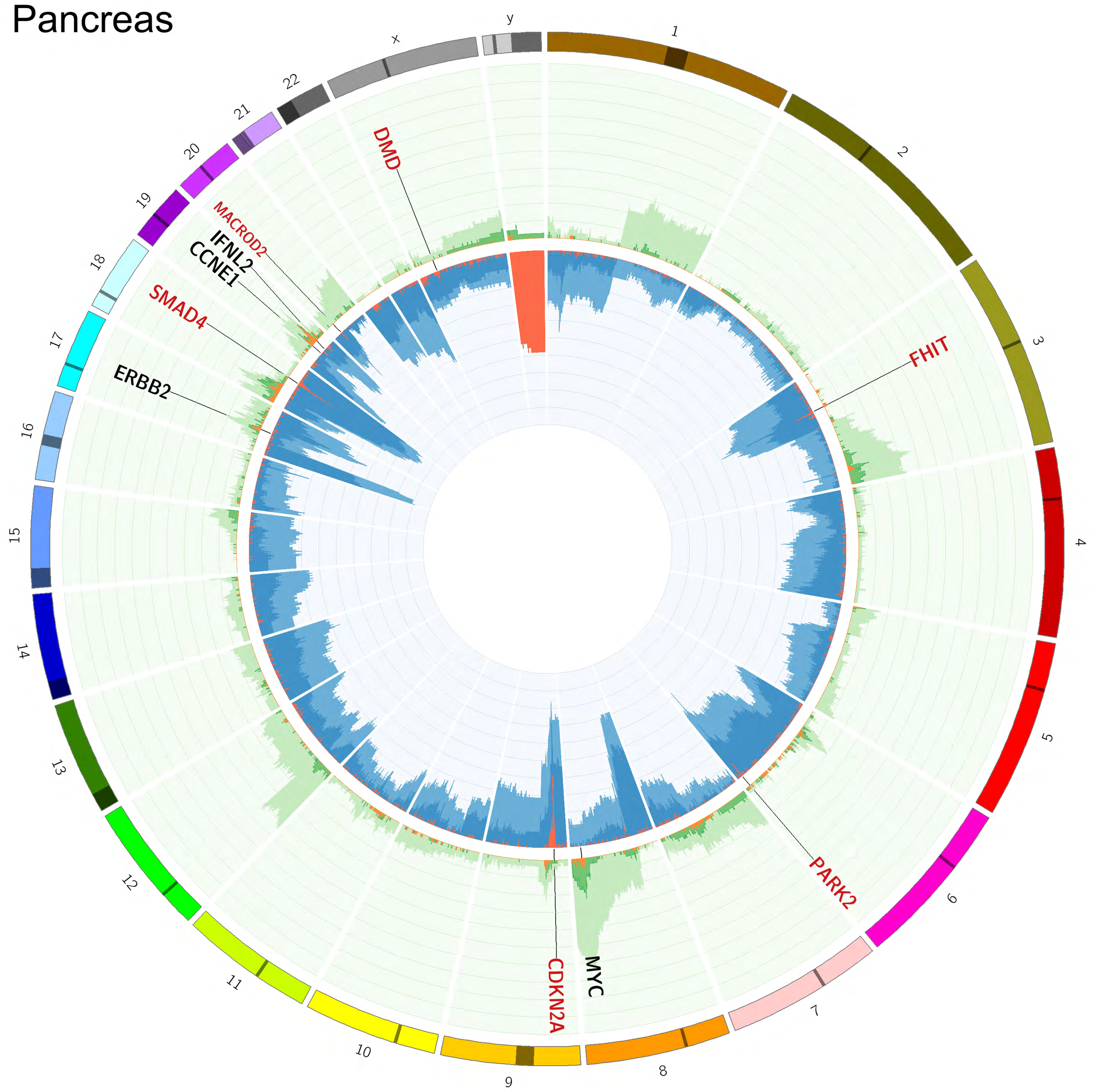

Prostate

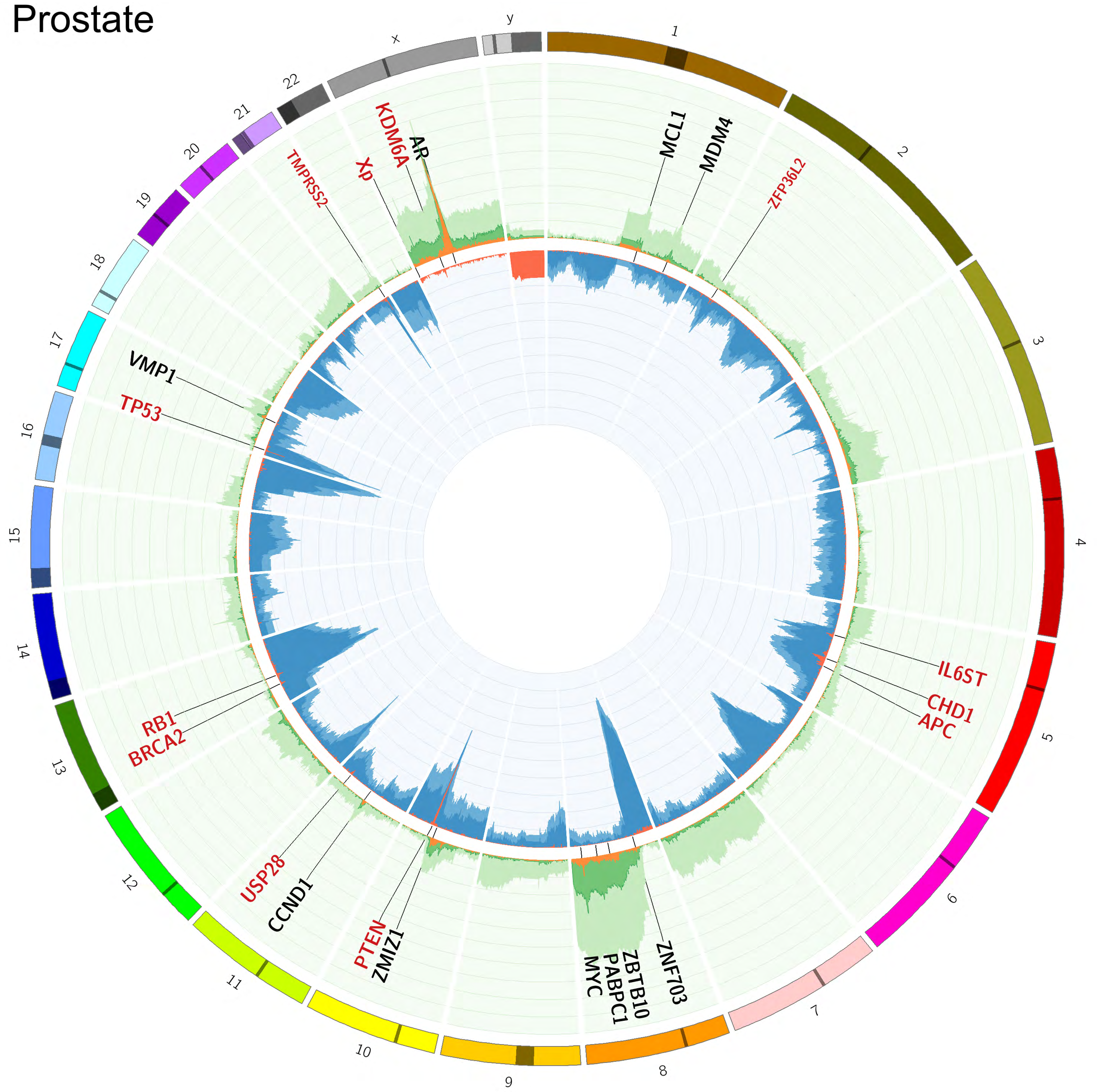

Skin

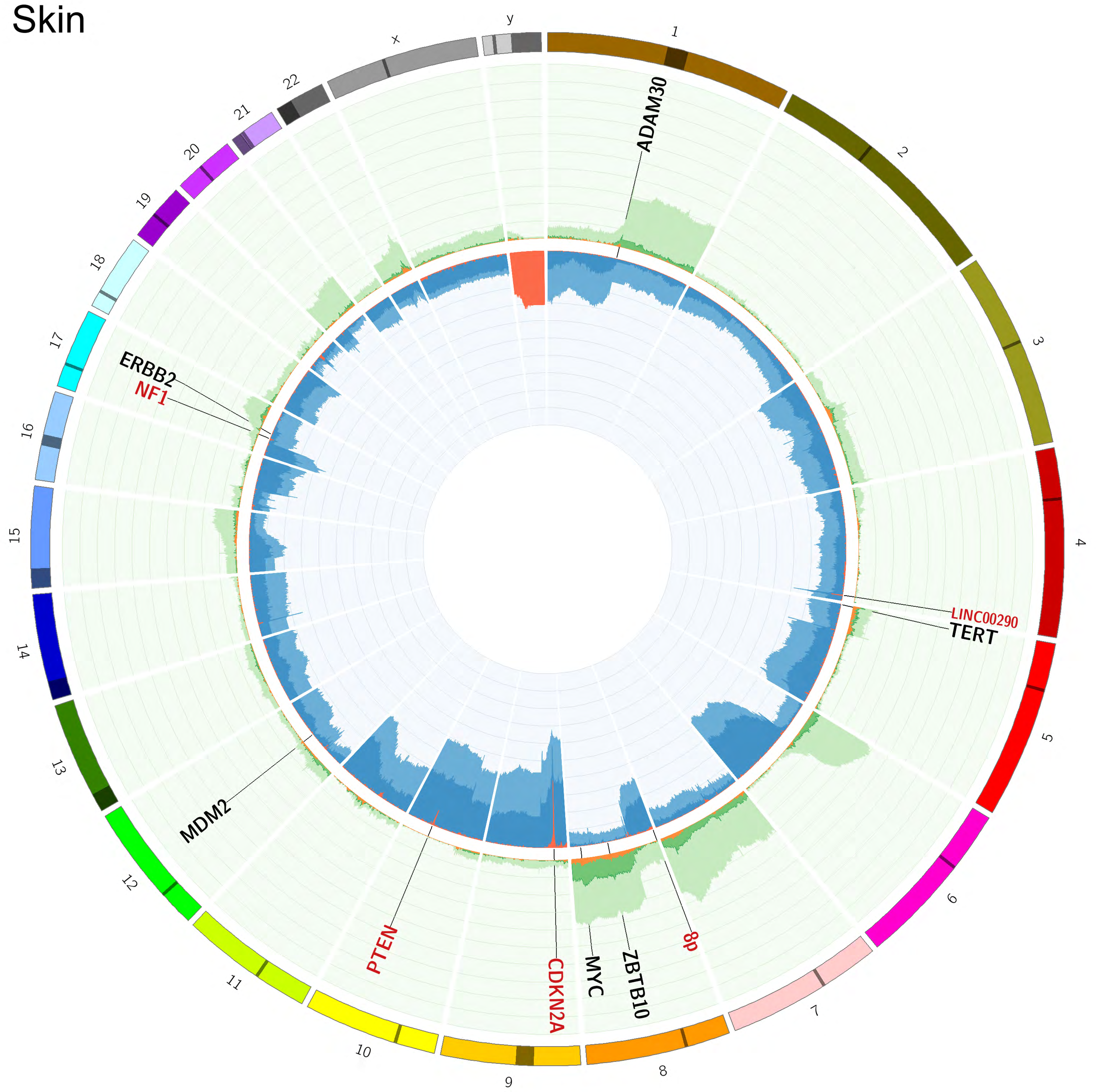

Stomach

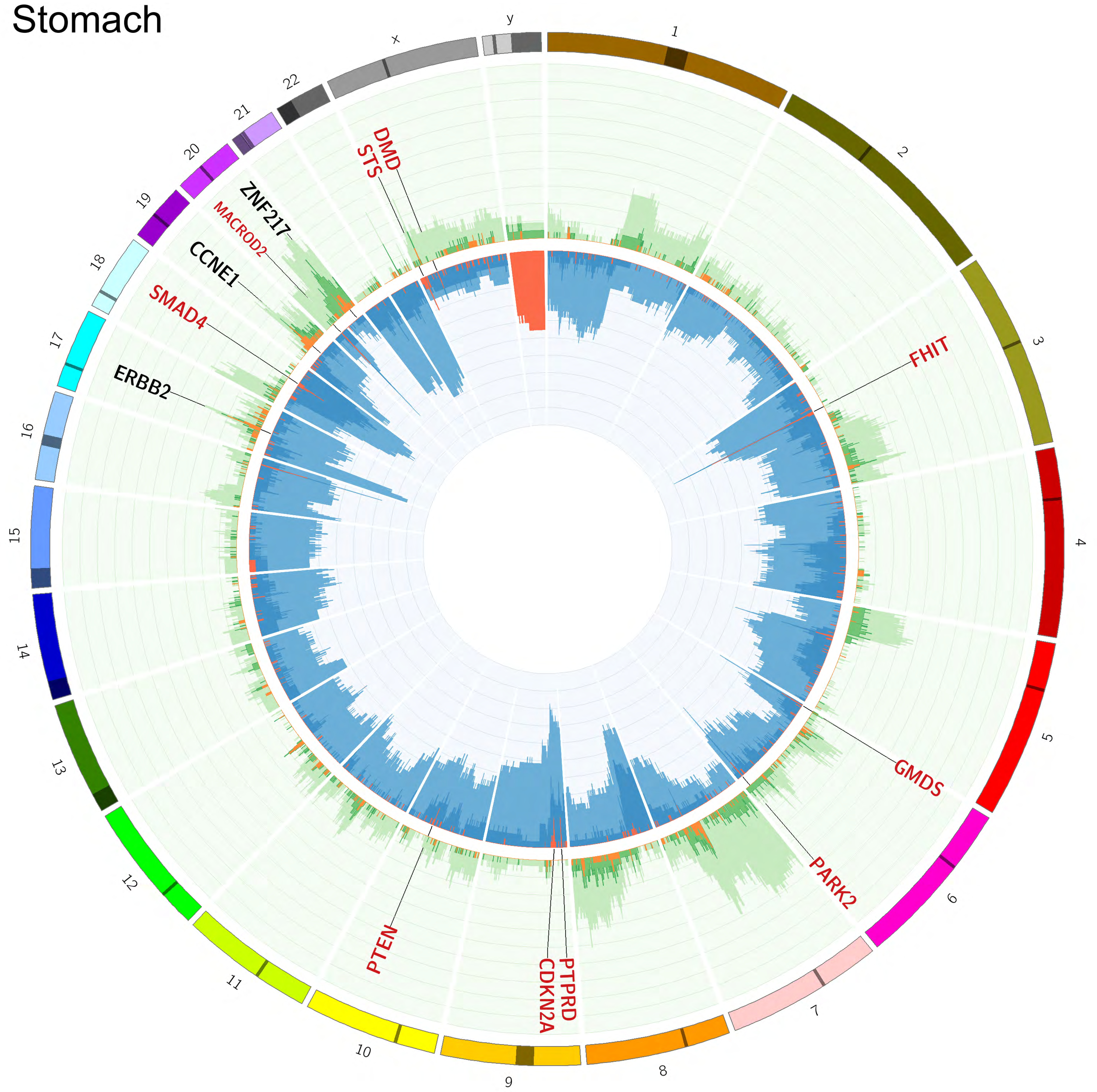

Urinary tract

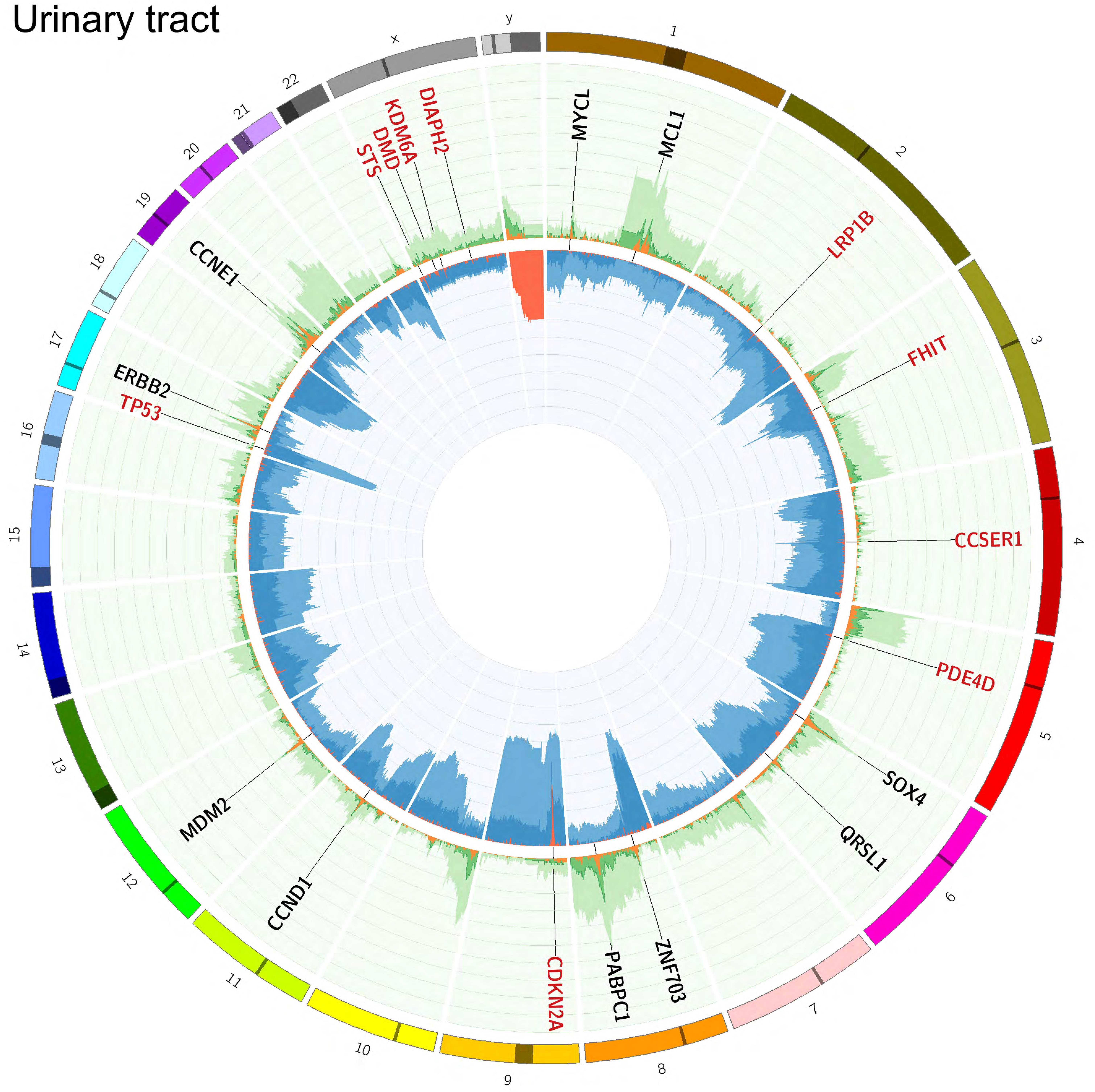

Uterus

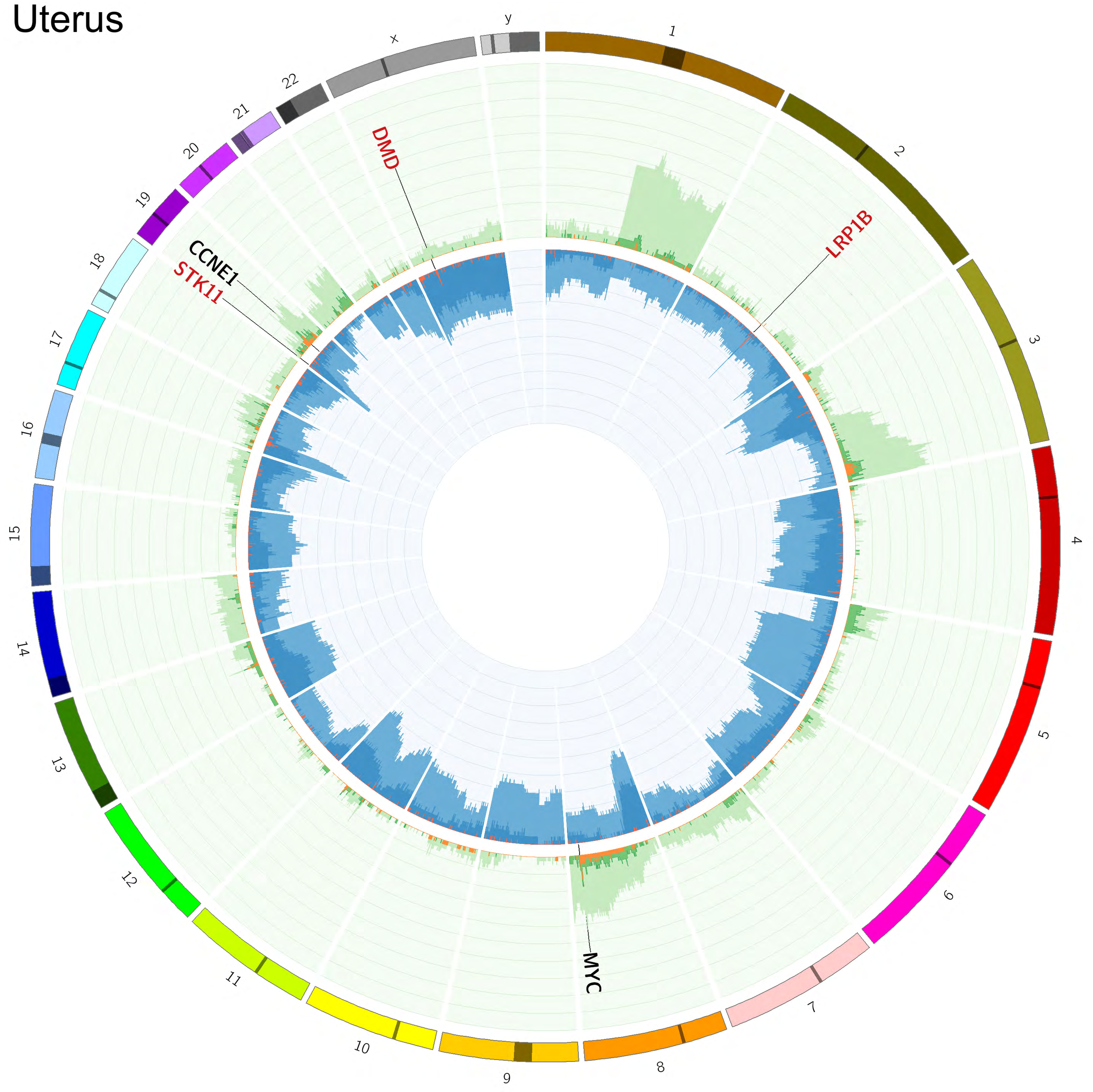

Supplement: Supplementary file 3 — Supplementary Figure 1.Copy Number profile per cancer types. Circos plots showing the proportion of samples with amplification and deletion events by genomic position per cancer type. The inner ring shows the % of tumours with homozygous deletion (red), LOH and significant loss (copy number < 0.6x sample ploidy - dark blue) and near copy neutral LOH (light blue). The outer ring shows the % of tumours with high level amplification (>3x sample ploidy - orange), moderate amplification (>2x sample ploidy - dark green) and low level amplification (>1.4x amplification - light green). Scales on both rings are 0-100% and inverted for the inner ring. The most frequently observed high level gene amplifications (black text) and homozygous deletions (red text) are labelled [file 41586_2019_1689_MOESM3_ESM.pdf]
