# Supplementary material for: Pan-cancer whole-genome analyses of metastatic solid tumours
Source: Nature. 2019 Oct 23;575(7781):210–6. doi: 10.1038/s41586-019-1689-y (PMC6872491; doi:10.1038/s41586-019-1689-y)
Supplement: Supplementary file 4 — Supplementary Figure 2.Coding mutation profiles by tumour suppressor driver gene. Location and driver classification of all coding mutations (SNVs and indels) in tumour suppressor genes (TSG) in the driver catalogue. The lollipops on the chart show the location (coding sequence coordinates) and count of mutations for all candidate drivers. The height of lollipop represents the total count of each individual variant in the cohort (log scale). The height of the solid line represents the sum of driver likelihoods for that variant, i.e. the proportion that are expected to be drivers. (Partially) dotted lines hence indicate variants for which driver role is uncertain. Variants are unshaded if all instances of that variant are monoallelic single hits with no LOH. The right column chart shows the estimated number of drivers (calculated as the sum of driver likelihoods) and passenger variants in each gene by cancer type [file 41586_2019_1689_MOESM4_ESM.pdf]

ACVR1B Variants

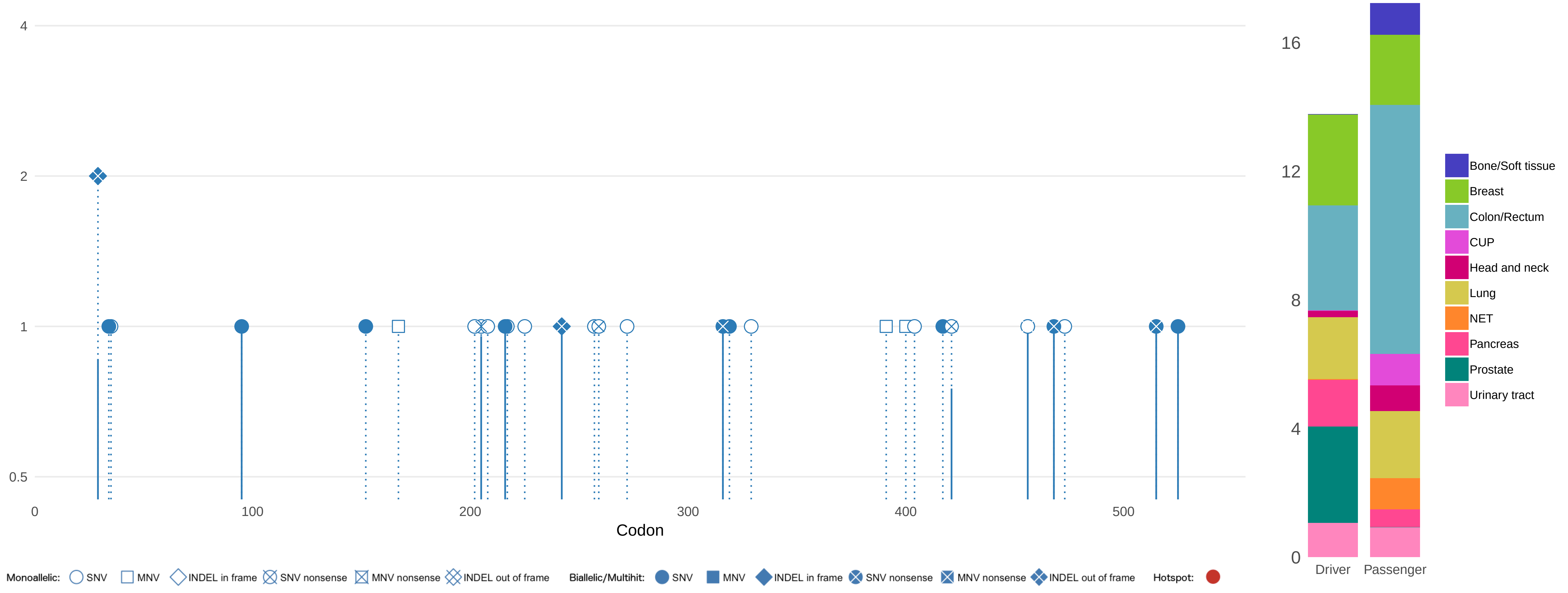

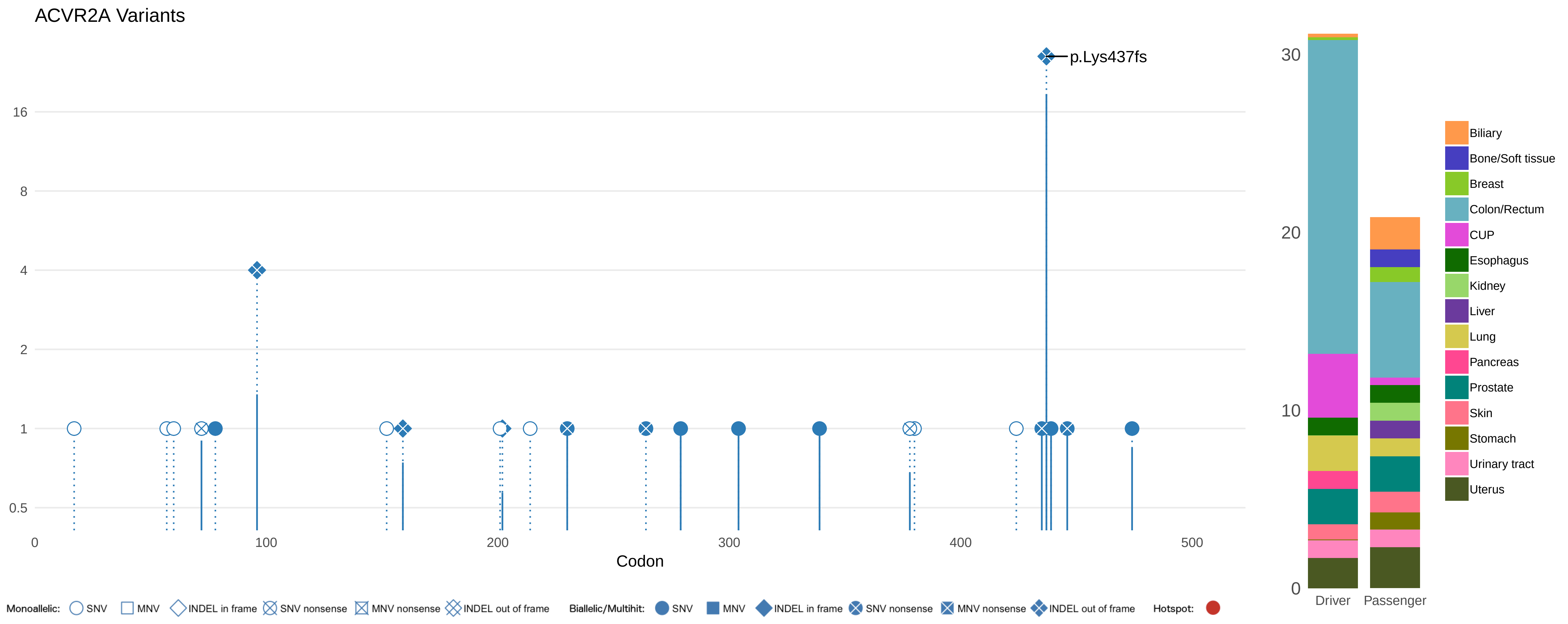

ACVR2A Variants
p.Lys437fs
16
8
4
2
1
0.5
0
100
200
300
400
500
Codon
30
20
10
0
Driver
Passenger
Biliary
Bone/Soft tissue
Breast
Colon/Rectum
CUP
Esophagus
Kidney
Liver
Lung
Pancreas
Prostate
Skin
Stomach
Urinary tract
Uterus
Monoallelic: SNV MNV INDEL in frame SNV nonsense MNV nonsense INDEL out of frame
Biallelic/Multihit: SNV MNV INDEL in frame SNV nonsense MNV nonsense INDEL out of frame
Hotspot:

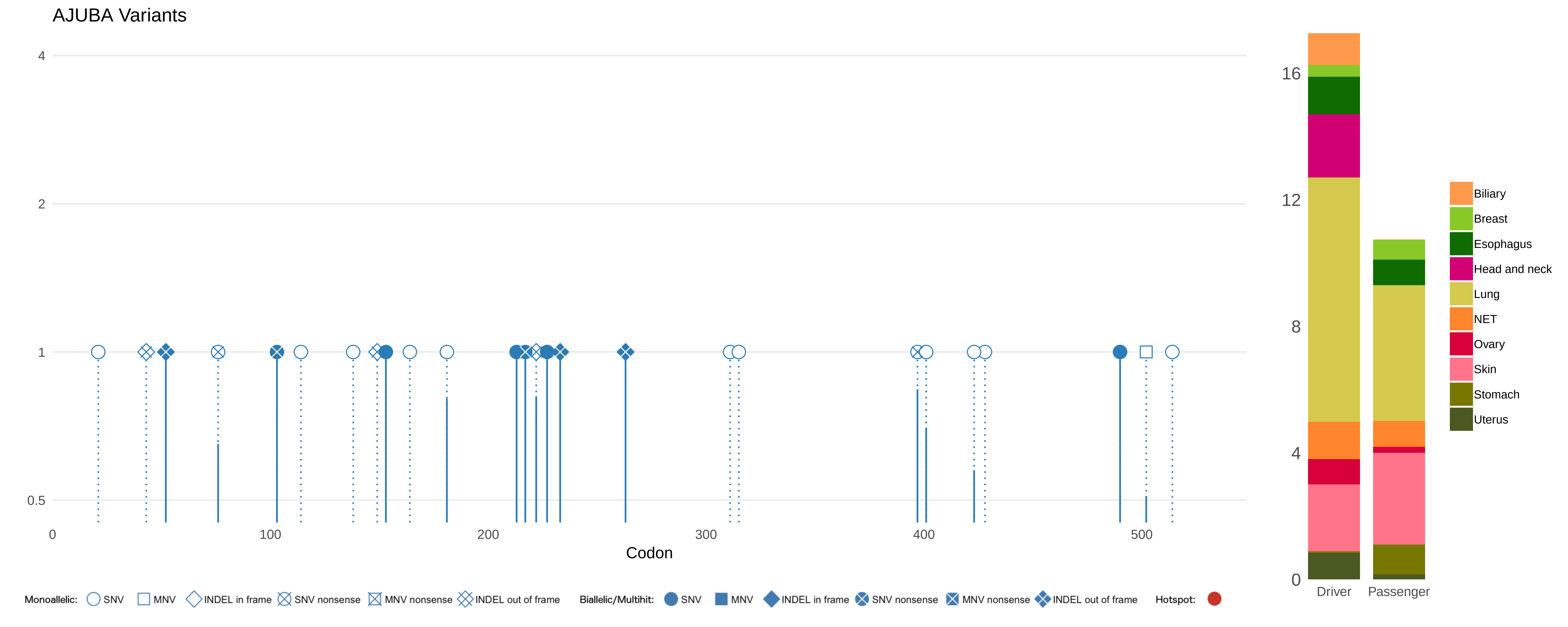
AJUBA Variants
4
2
1
0.5
0
100
200
300
400
500
Codon
16
12
8
4
0
Driver
Passenger
Biliary
Breast
Esophagus
Head and neck
Lung
NET
Ovary
Skin
Stomach
Uterus
Monoallelic: SNV MNV INDEL in frame SNV nonsense MNV nonsense INDEL out of frame
Biallelic/Multihit: SNV MNV INDEL in frame SNV nonsense MNV nonsense INDEL out of frame
Hotspot:

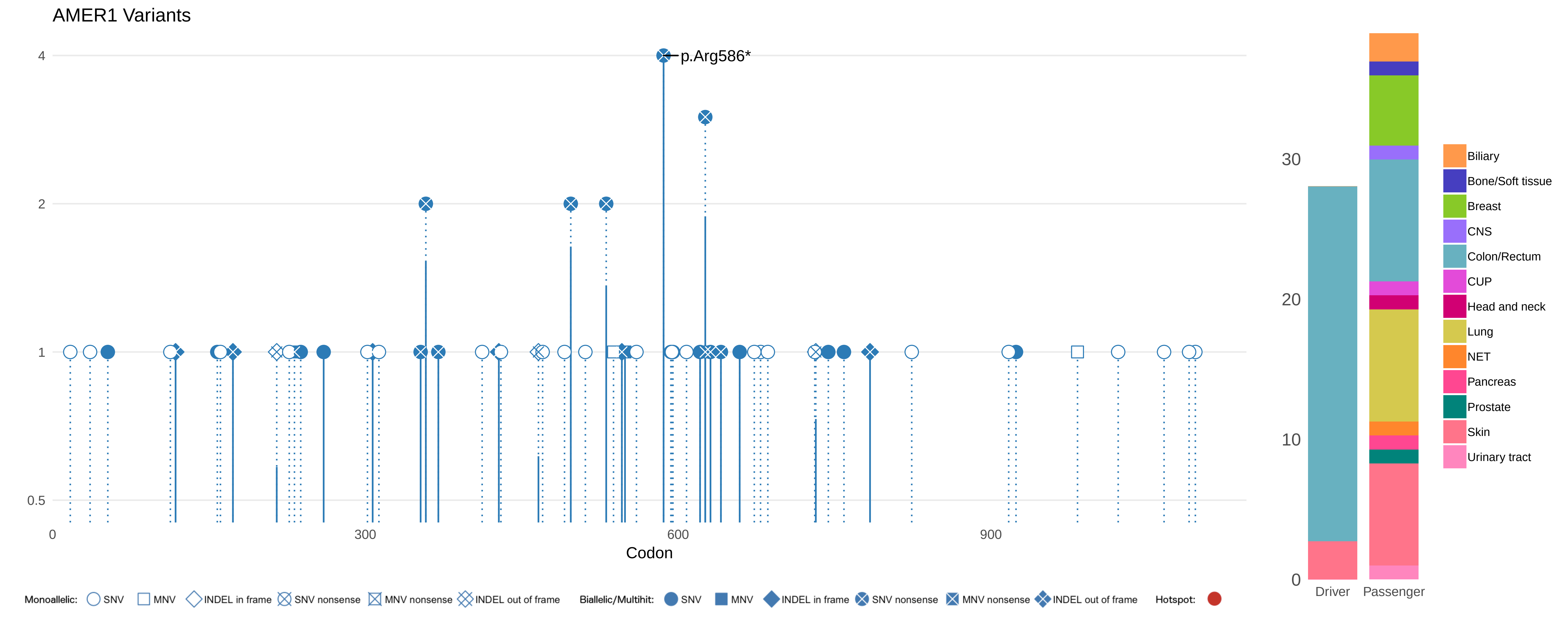

AMER1 Variants
p.Arg586*
4
2
1
0.5
0
300
600
900
Codon
30
20
10
0
Driver
Passenger
Biliary
Bone/Soft tissue
Breast
CNS
Colon/Rectum
CUP
Head and neck
Lung
NET
Pancreas
Prostate
Skin
Urinary tract
Monoallelic: SNV MNV INDEL in frame SNV nonsense MNV nonsense INDEL out of frame
Biallelic/Multihit: SNV MNV INDEL in frame SNV nonsense MNV nonsense INDEL out of frame
Hotspot:

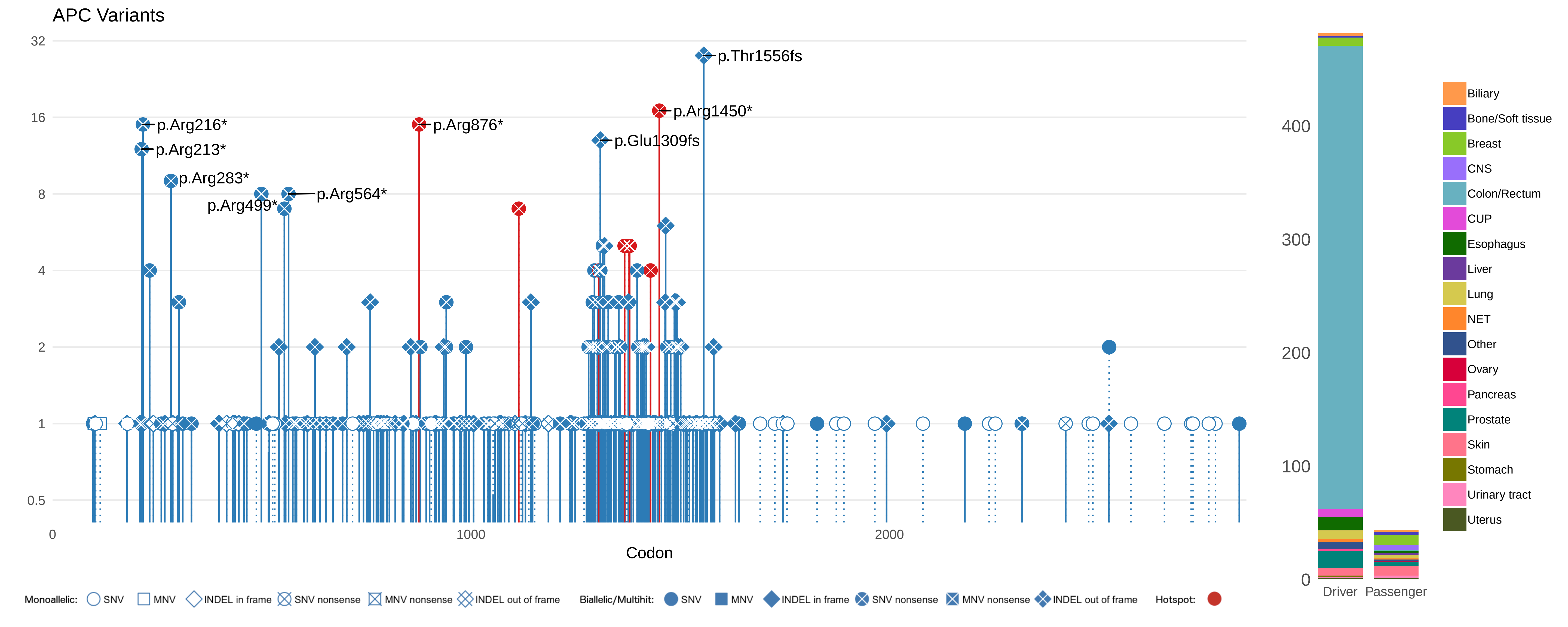

APC Variants
32
16
8
4
2
1
0.5
0
1000
2000
Codon
p.Thr1556fs
p.Arg1450*
p.Arg216*
p.Arg876*
p.Glu1309fs
p.Arg213*
p.Arg283*
p.Arg564*
p.Arg499*
400
300
200
100
0
Driver
Passenger
Biliary
Bone/Soft tissue
Breast
CNS
Colon/Rectum
CUP
Esophagus
Liver
Lung
NET
Other
Ovary
Pancreas
Prostate
Skin
Stomach
Urinary tract
Uterus
Monoallelic: SNV MNV INDEL in frame SNV nonsense MNV nonsense INDEL out of frame
Biallelic/Multihit: SNV MNV INDEL in frame SNV nonsense MNV nonsense INDEL out of frame
Hotspot:

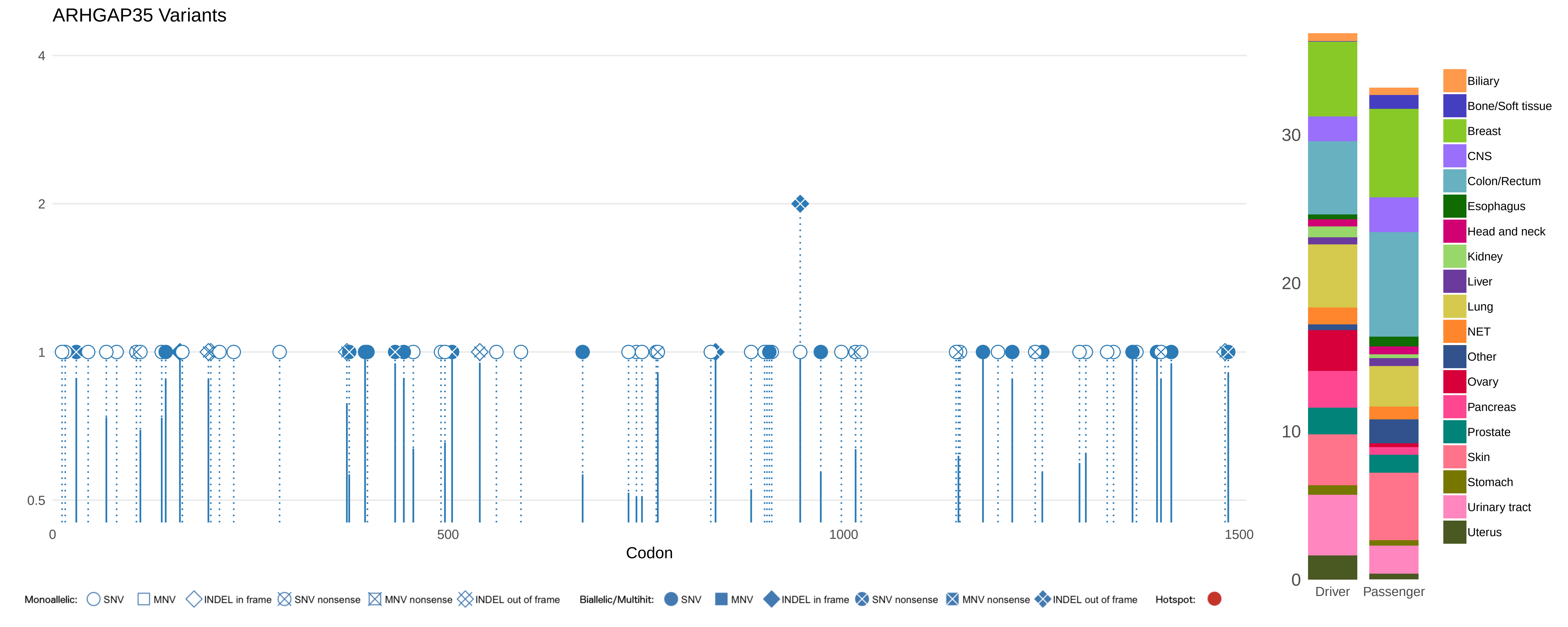
ARHGAP35 Variants
4
2
1
0.5
0
500
1000
1500
Codon
30
20
10
0
Driver
Passenger
Biliary
Bone/Soft tissue
Breast
CNS
Colon/Rectum
Esophagus
Head and neck
Kidney
Liver
Lung
NET
Other
Ovary
Pancreas
Prostate
Skin
Stomach
Urinary tract
Uterus
Monoallelic: SNV MNV INDEL in frame SNV nonsense MNV nonsense INDEL out of frame
Biallelic/Multihit: SNV MNV INDEL in frame SNV nonsense MNV nonsense INDEL out of frame
Hotspot:

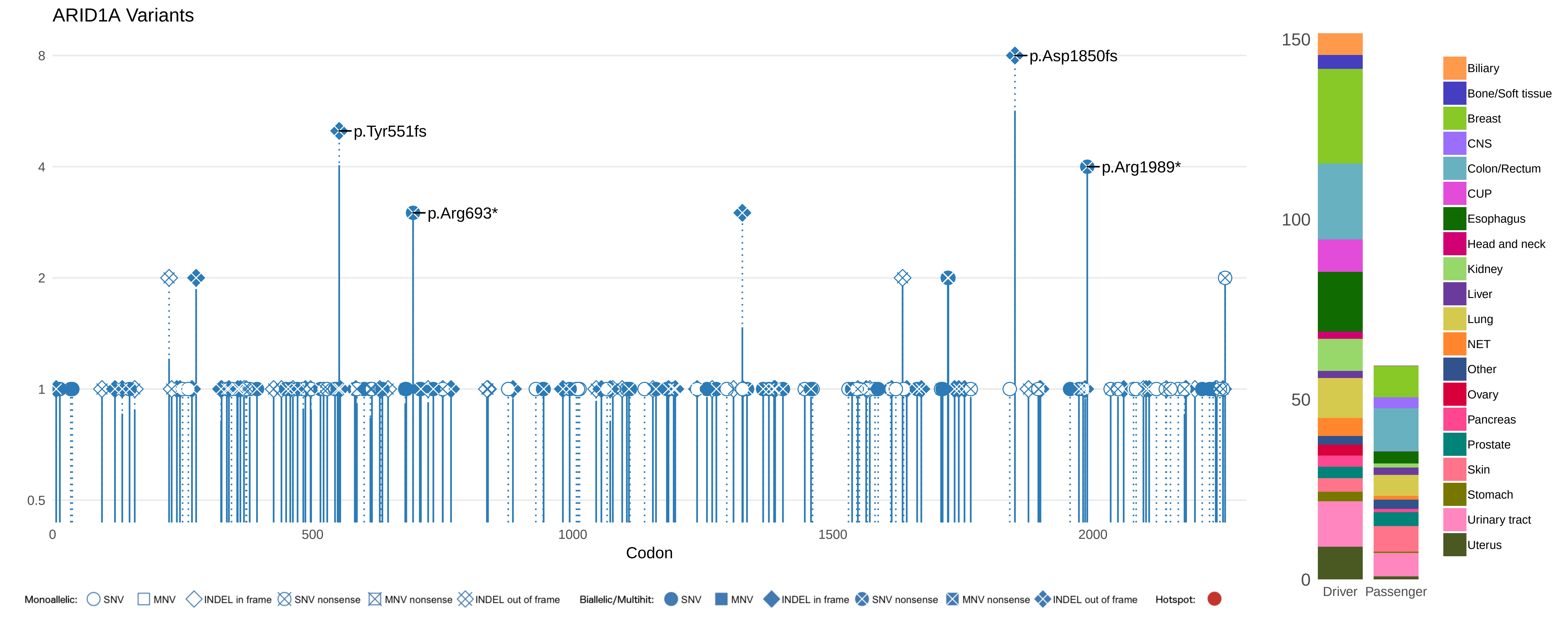

ARID1A Variants
p.Asp1850fs
p.Tyr551fs
p.Arg1989*
p.Arg693*
8
4
2
1
0.5
0
500
1000
1500
2000
Codon
150
100
50
0
Driver
Passenger
Biliary
Bone/Soft tissue
Breast
CNS
Colon/Rectum
CUP
Esophagus
Head and neck
Kidney
Liver
Lung
NET
Other
Ovary
Pancreas
Prostate
Skin
Stomach
Urinary tract
Uterus
Monoallelic: SNV MNV INDEL in frame SNV nonsense MNV nonsense INDEL out of frame
Biallelic/Multihit: SNV MNV INDEL in frame SNV nonsense MNV nonsense INDEL out of frame
Hotspot:

ARID1B Variants

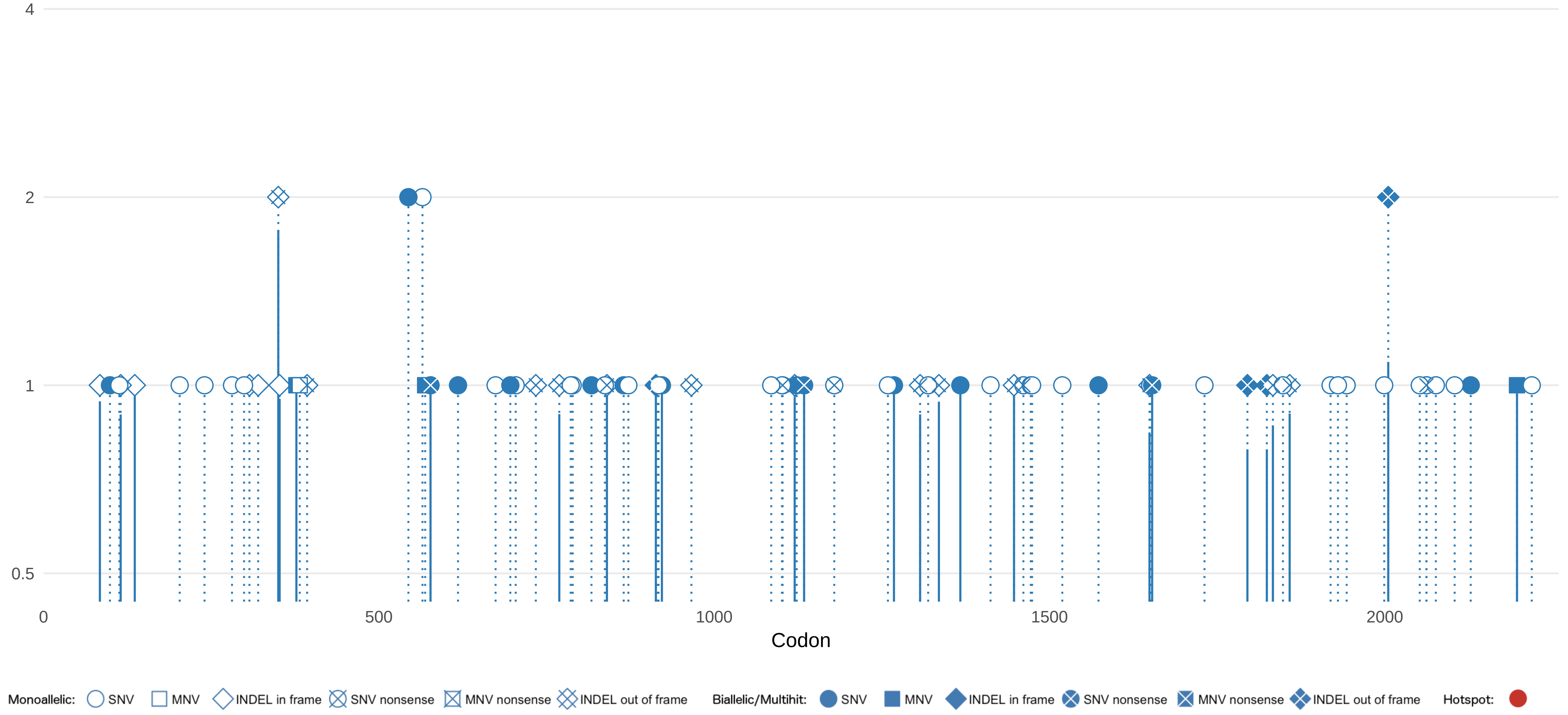

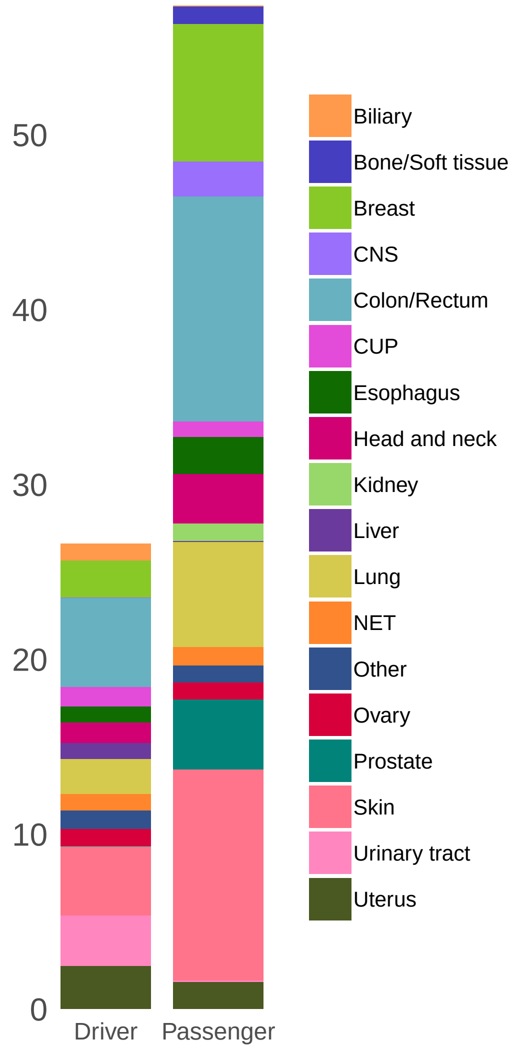

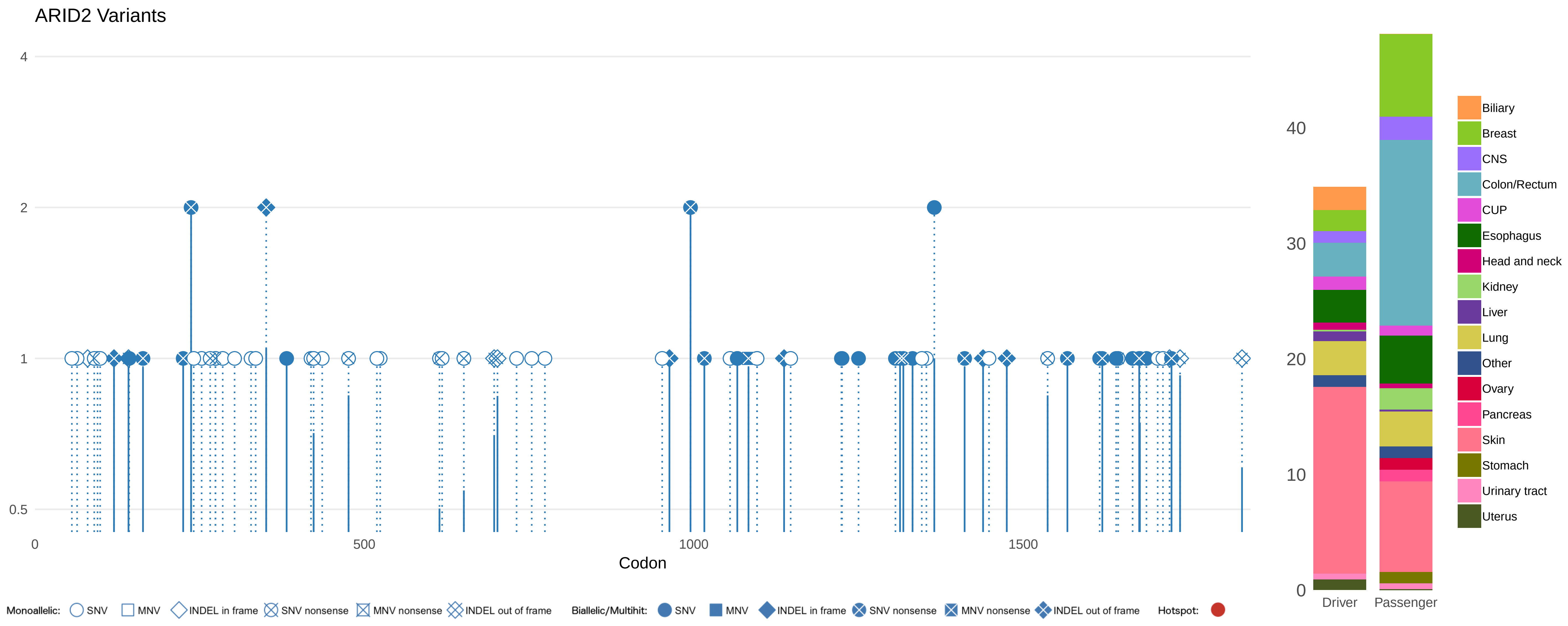
ARID2 Variants
4
2
1
0.5
0
500
1000
1500
Codon
40
30
20
10
0
Driver
Passenger
Biliary
Breast
CNS
Colon/Rectum
CUP
Esophagus
Head and neck
Kidney
Liver
Lung
Other
Ovary
Pancreas
Skin
Stomach
Urinary tract
Uterus
Monoallelic: SNV MNV INDEL in frame SNV nonsense MNV nonsense INDEL out of frame
Biallelic/Multihit: SNV MNV INDEL in frame SNV nonsense MNV nonsense INDEL out of frame
Hotspot:

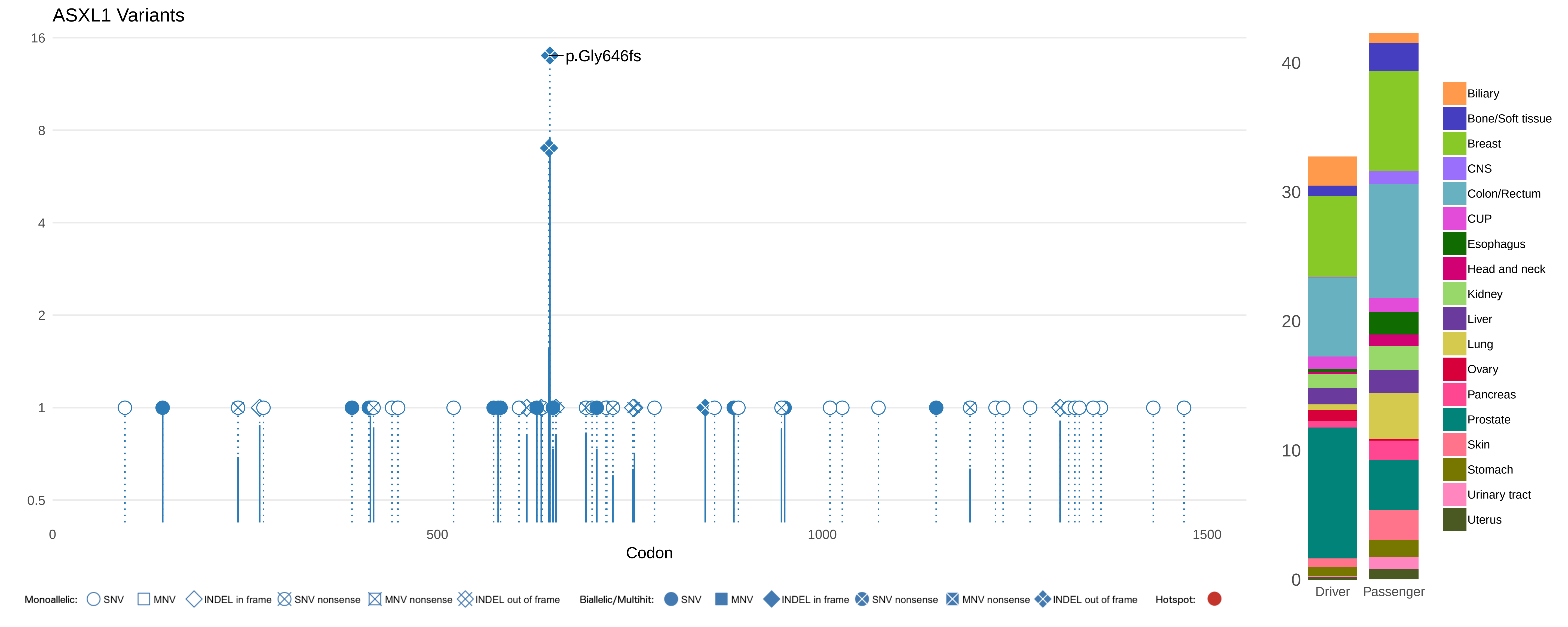
ASXL1 Variants
16
8
4
2
1
0.5
p.Gly646fs
0
500
1000
1500
Codon
40
30
20
10
0
Driver
Passenger
Biliary
Bone/Soft tissue
Breast
CNS
Colon/Rectum
CUP
Esophagus
Head and neck
Kidney
Liver
Lung
Ovary
Pancreas
Prostate
Skin
Stomach
Urinary tract
Uterus
Monoallelic: SNV MNV INDEL in frame SNV nonsense MNV nonsense INDEL out of frame
Biallelic/Multihit: SNV MNV INDEL in frame SNV nonsense MNV nonsense INDEL out of frame
Hotspot:

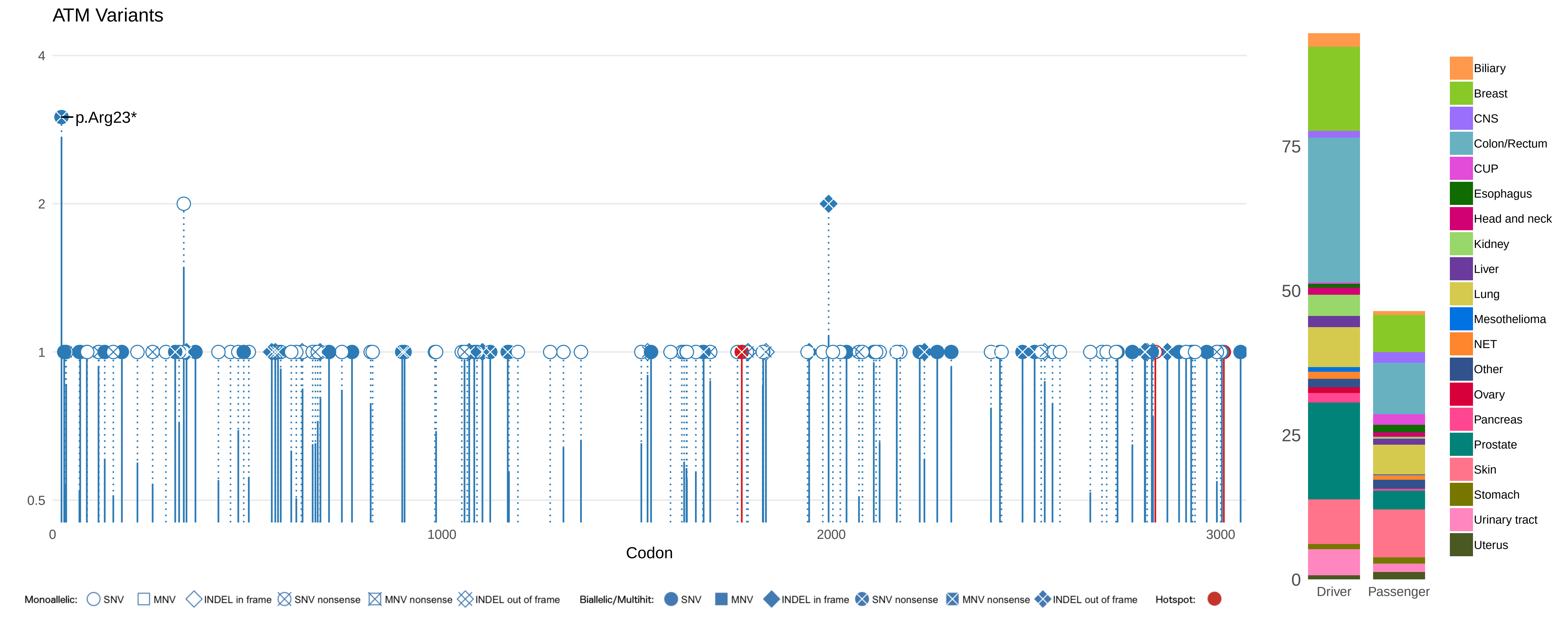

ATM Variants
p.Arg23*
4
2
1
0.5
0
1000
2000
3000
Codon
75
50
25
0
Driver
Passenger
Biliary
Breast
CNS
Colon/Rectum
CUP
Esophagus
Head and neck
Kidney
Liver
Lung
Mesothelioma
NET
Other
Ovary
Pancreas
Prostate
Skin
Stomach
Urinary tract
Uterus
Monoallelic: SNV MNV INDEL in frame SNV nonsense MNV nonsense INDEL out of frame
Biallelic/Multihit: SNV MNV INDEL in frame SNV nonsense MNV nonsense INDEL out of frame
Hotspot:

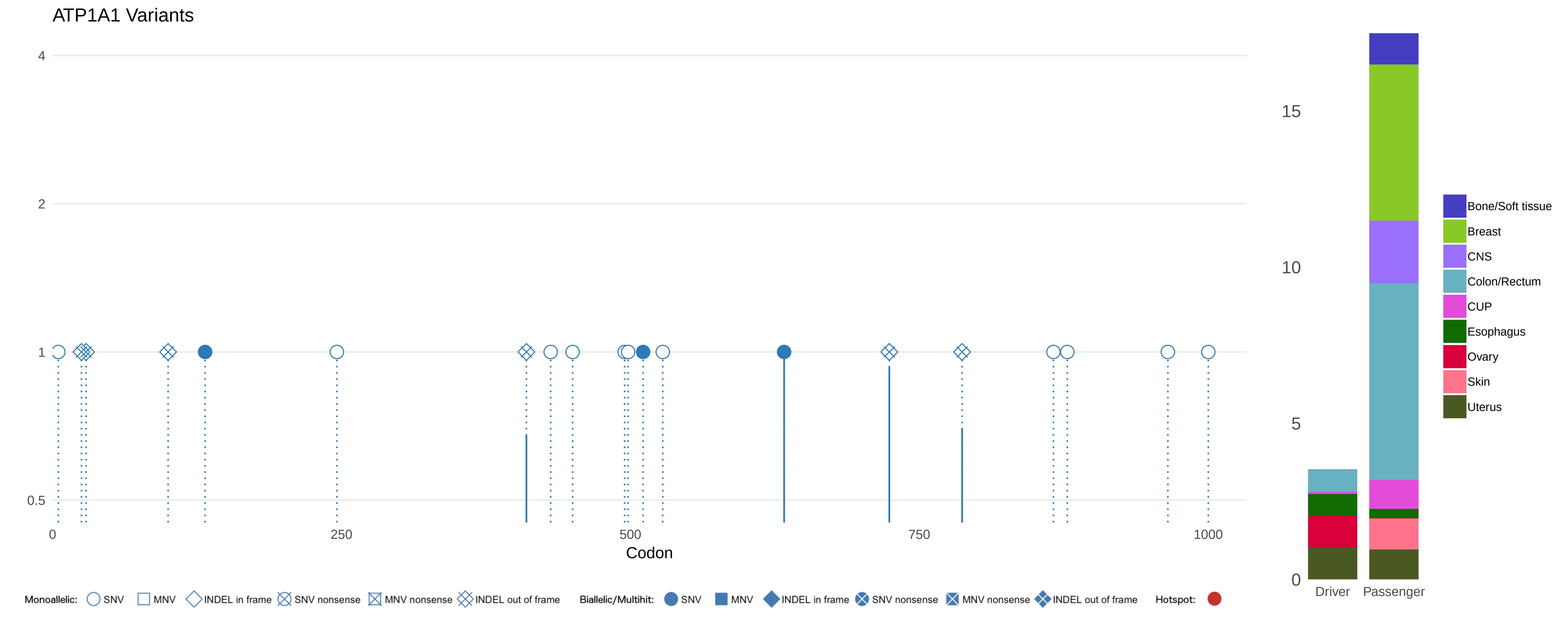

ATP1A1 Variants
4
2
1
0.5
0
250
500
750
1000
Codon
15
10
5
0
Driver
Passenger
Bone/Soft tissue
Breast
CNS
Colon/Rectum
CUP
Esophagus
Ovary
Skin
Uterus
Monoallelic:
SNV
MNV
INDEL in frame
SNV nonsense
MNV nonsense
INDEL out of frame
Biallelic/Multihit:
SNV
MNV
INDEL in frame
SNV nonsense
MNV nonsense
INDEL out of frame
Hotspot:

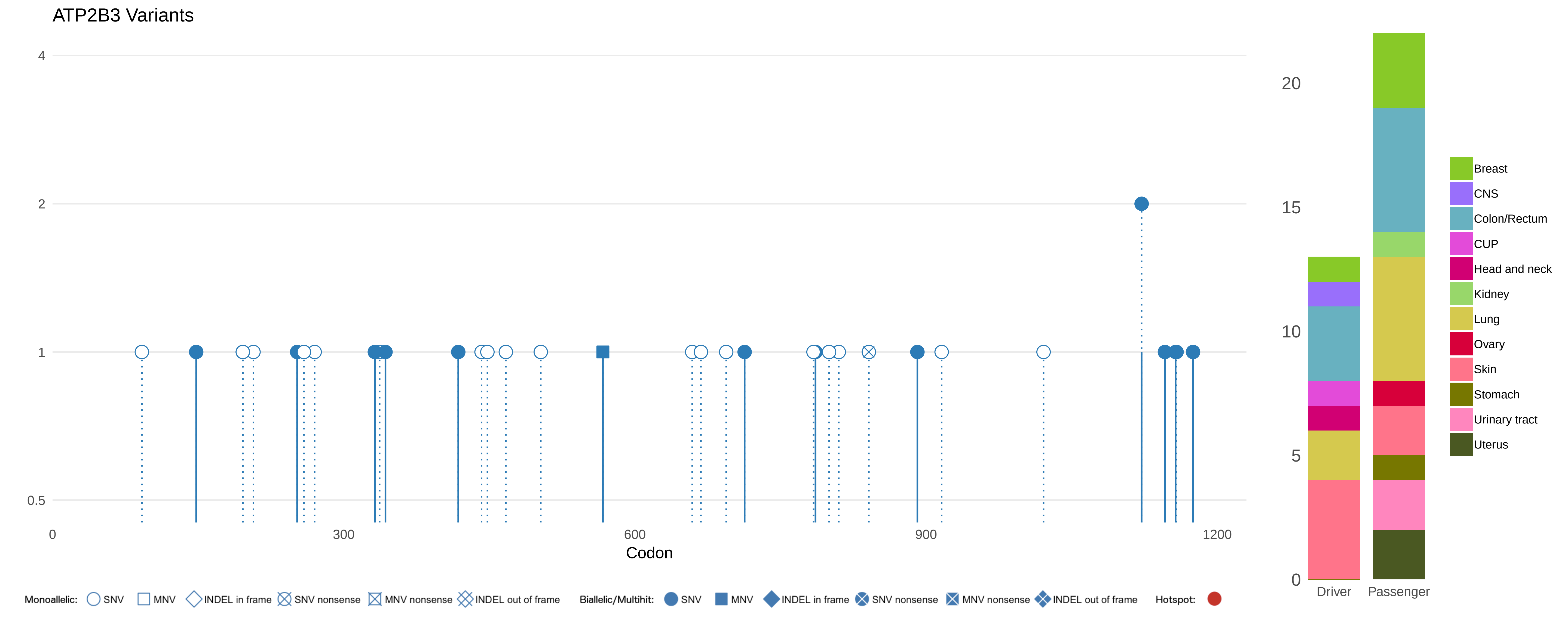

ATP2B3 Variants
4
2
1
0.5
0
300
600
900
1200
Codon
20
15
10
5
0
Driver
Passenger
Breast
CNS
Colon/Rectum
CUP
Head and neck
Kidney
Lung
Ovary
Skin
Stomach
Urinary tract
Uterus
Monoallelic: SNV MNV INDEL in frame SNV nonsense MNV nonsense INDEL out of frame
Biallelic/Multihit: SNV MNV INDEL in frame SNV nonsense MNV nonsense INDEL out of frame
Hotspot:

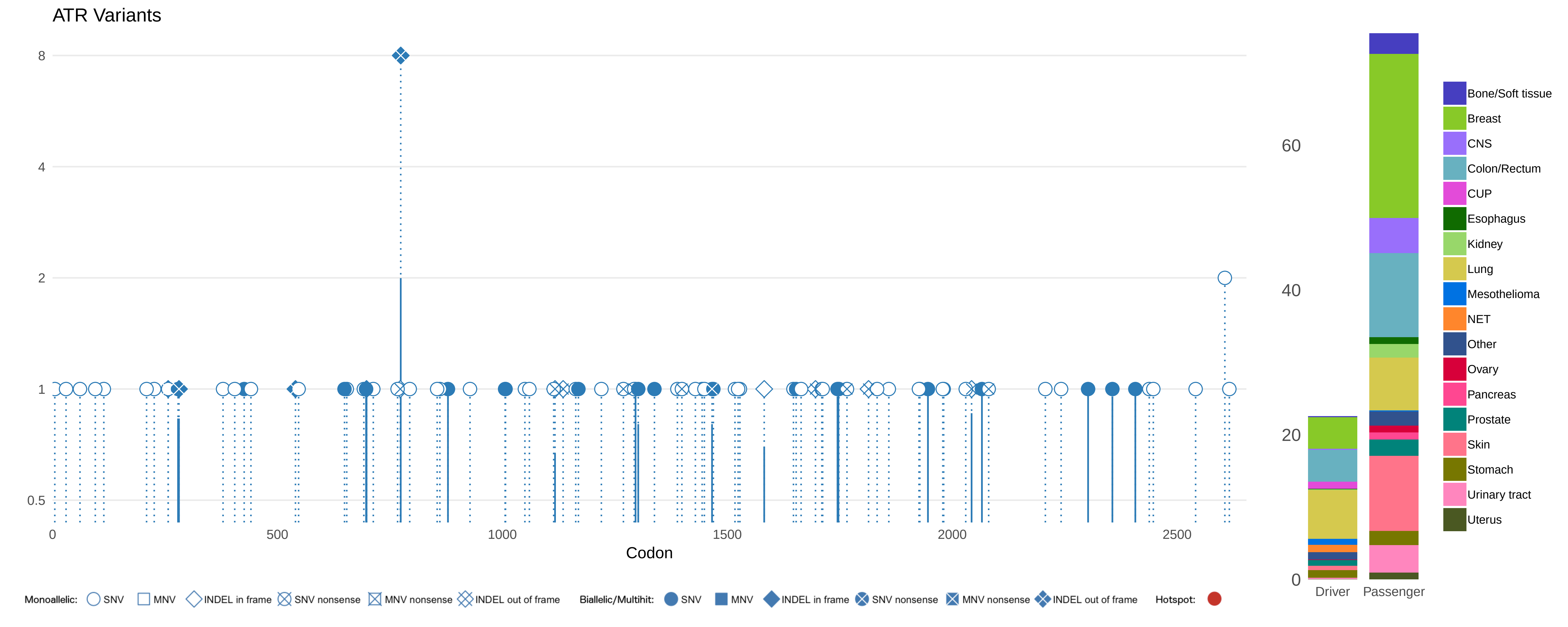
ATR Variants
8
4
2
1
0.5
0
500
1000
1500
2000
2500
Codon
60
40
20
0
Driver
Passenger
Bone/Soft tissue
Breast
CNS
Colon/Rectum
CUP
Esophagus
Kidney
Lung
Mesothelioma
NET
Other
Ovary
Pancreas
Prostate
Skin
Stomach
Urinary tract
Uterus
Monoallelic: SNV MNV INDEL in frame SNV nonsense MNV nonsense INDEL out of frame
Biallelic/Multihit: SNV MNV INDEL in frame SNV nonsense MNV nonsense INDEL out of frame
Hotspot:

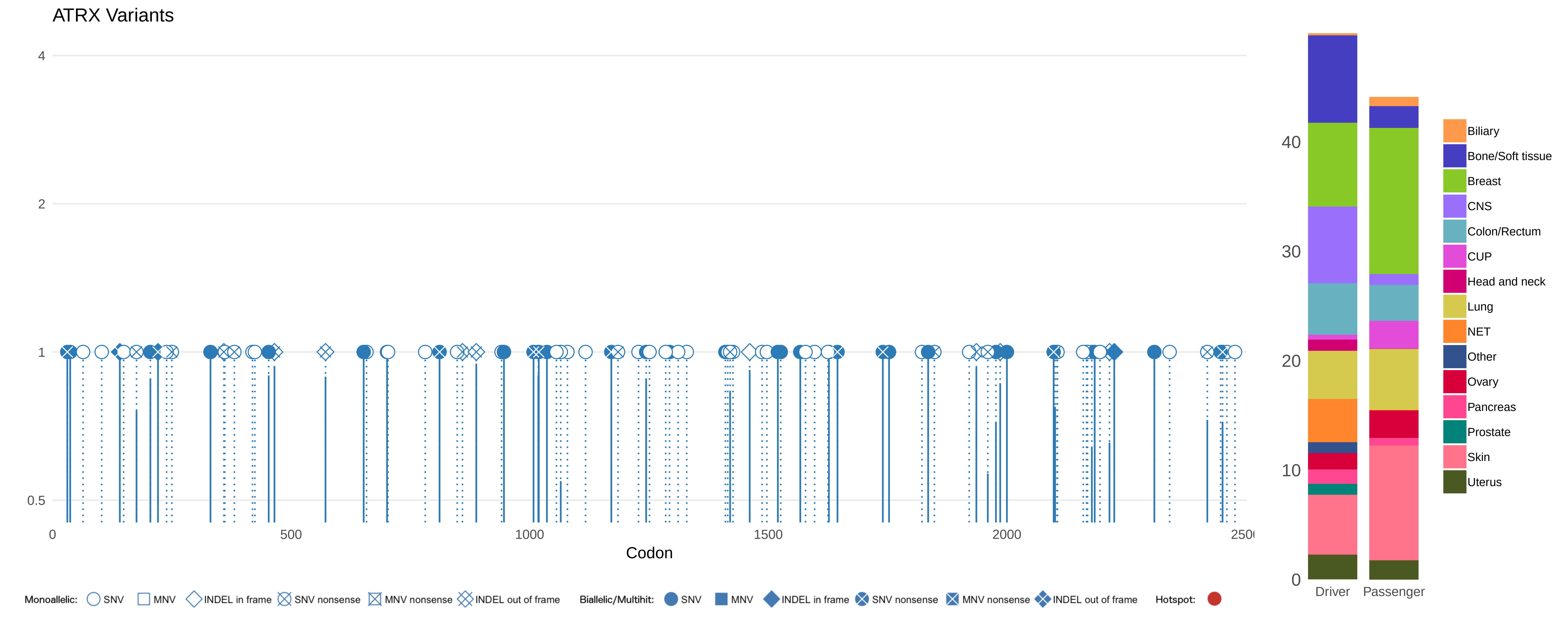
ATRX Variants
4
2
1
0.5
0
500
1000
1500
2000
2500
Codon
Monoallelic: SNV MNV INDEL in frame SNV nonsense MNV nonsense INDEL out of frame
Biallelic/Multihit: SNV MNV INDEL in frame SNV nonsense MNV nonsense INDEL out of frame
Hotspot:
40
30
20
10
0
Driver
Passenger
Biliary
Bone/Soft tissue
Breast
CNS
Colon/Rectum
CUP
Head and neck
Lung
NET
Other
Ovary
Pancreas
Prostate
Skin
Uterus

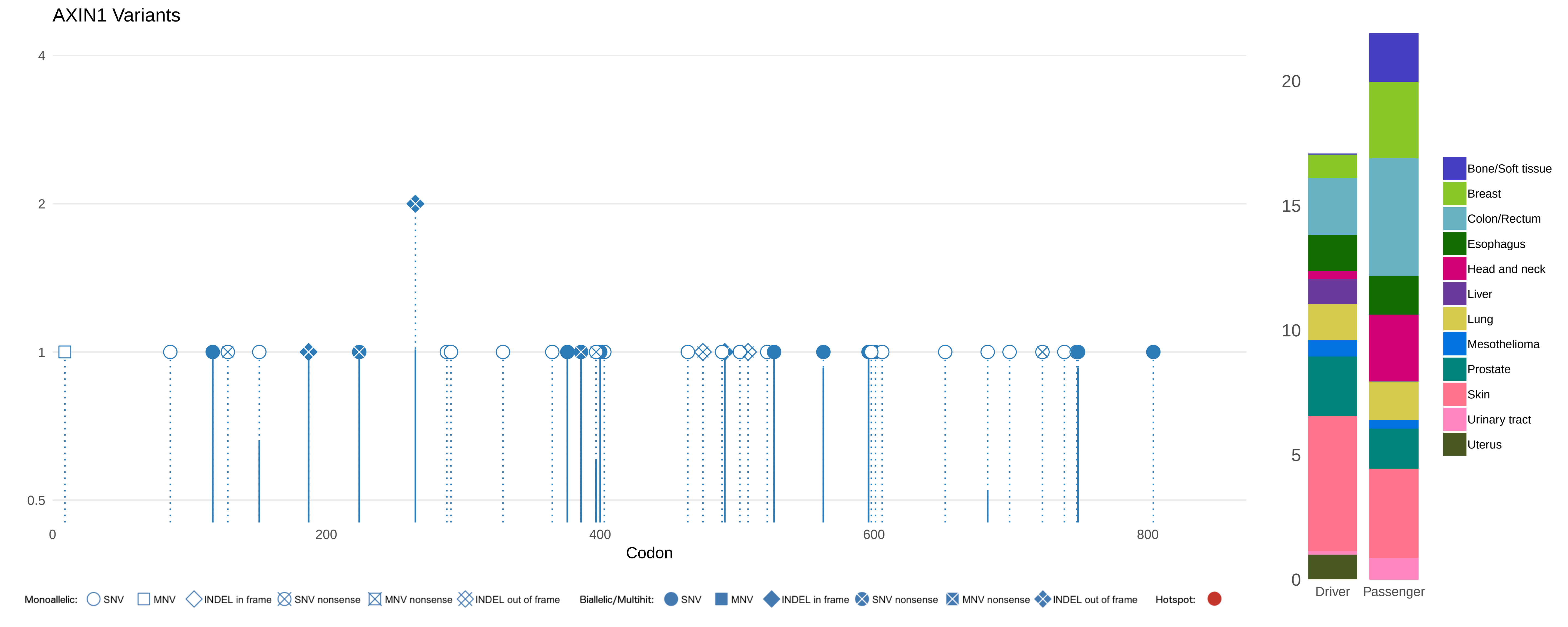

AXIN1 Variants
4
2
1
0.5
0
200
400
600
800
Codon
20
15
10
5
0
Driver
Passenger
Bone/Soft tissue
Breast
Colon/Rectum
Esophagus
Head and neck
Liver
Lung
Mesothelioma
Prostate
Skin
Urinary tract
Uterus
Monoallelic: SNV MNV INDEL in frame SNV nonsense MNV nonsense INDEL out of frame
Biallelic/Multihit: SNV MNV INDEL in frame SNV nonsense MNV nonsense INDEL out of frame
Hotspot:

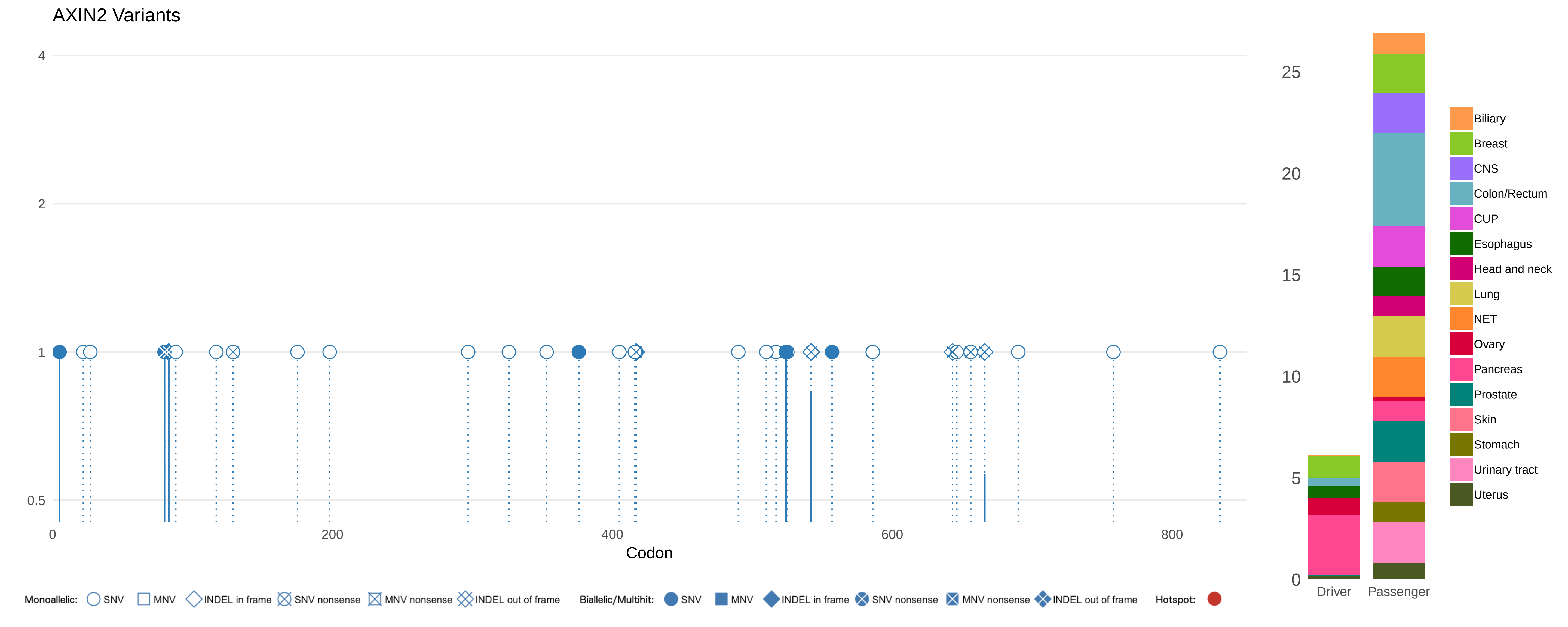
AXIN2 Variants
4
2
1
0.5
0
200
400
600
800
Codon
25
20
15
10
5
0
Driver
Passenger
Biliary
Breast
CNS
Colon/Rectum
CUP
Esophagus
Head and neck
Lung
NET
Ovary
Pancreas
Prostate
Skin
Stomach
Urinary tract
Uterus
Monoallelic: SNV MNV INDEL in frame SNV nonsense MNV nonsense INDEL out of frame
Biallelic/Multihit: SNV MNV INDEL in frame SNV nonsense MNV nonsense INDEL out of frame
Hotspot:

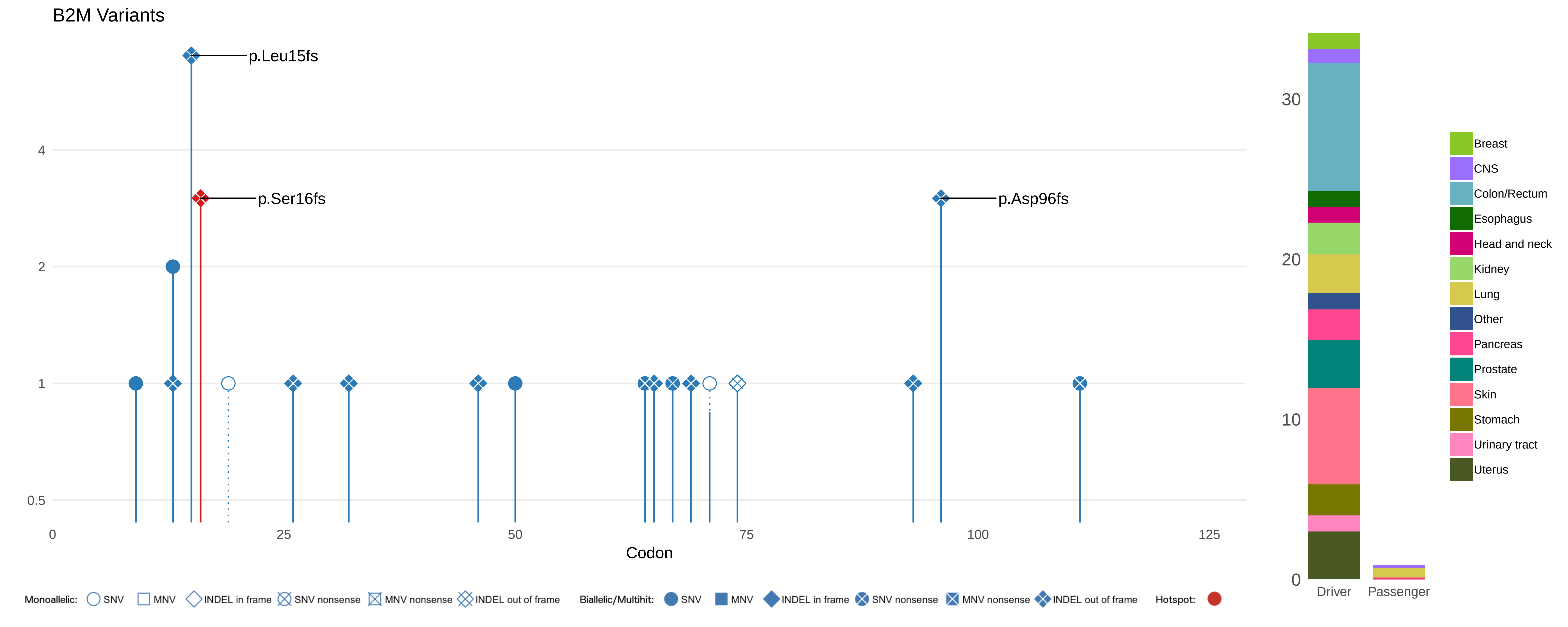

B2M Variants
p.Leu15fs
p.Ser16fs
p.Asp96fs
4
2
1
0.5
0
25
50
75
100
125
Codon
30
20
10
0
Driver
Passenger
Breast
CNS
Colon/Rectum
Esophagus
Head and neck
Kidney
Lung
Other
Pancreas
Prostate
Skin
Stomach
Urinary tract
Uterus
Monoallelic: SNV MNV INDEL in frame SNV nonsense MNV nonsense INDEL out of frame
Biallelic/Multihit: SNV MNV INDEL in frame SNV nonsense MNV nonsense INDEL out of frame
Hotspot:

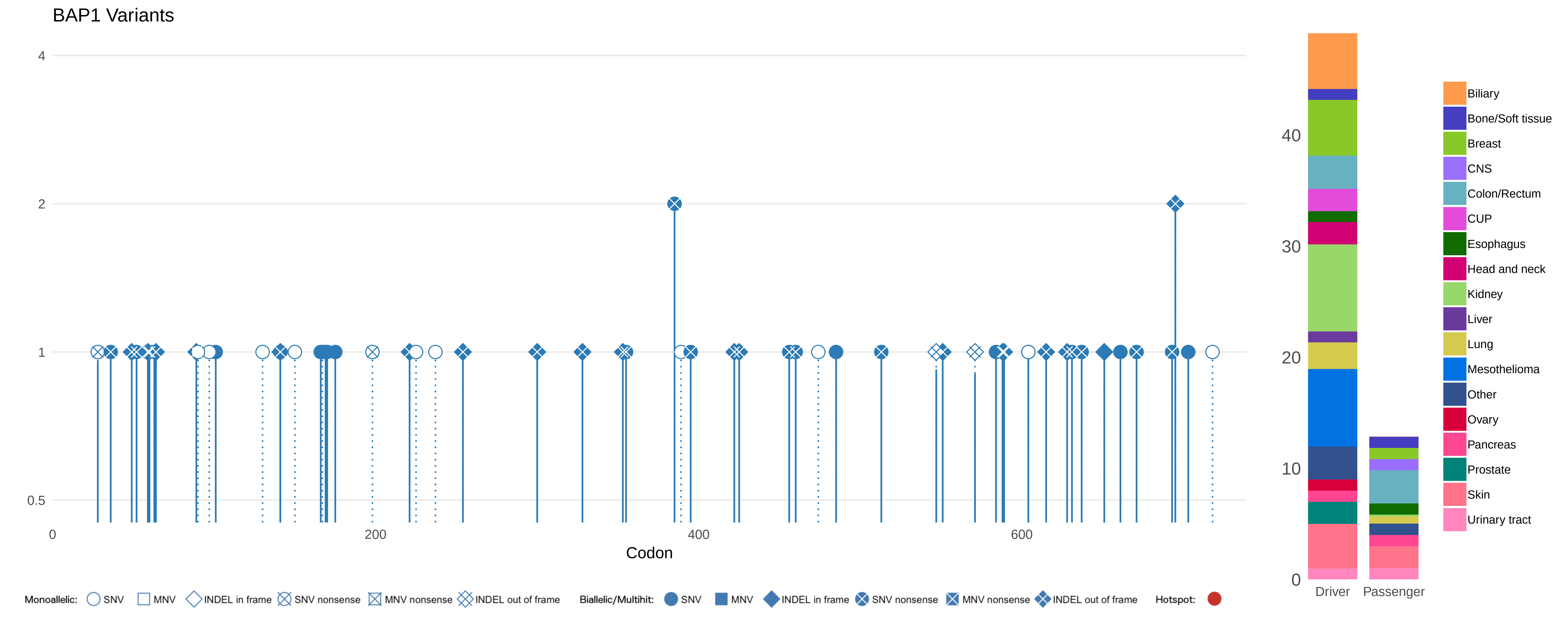

BAP1 Variants
4
2
1
0.5
0
200
400
600
Codon
40
30
20
10
0
Driver
Passenger
Biliary
Bone/Soft tissue
Breast
CNS
Colon/Rectum
CUP
Esophagus
Head and neck
Kidney
Liver
Lung
Mesothelioma
Other
Ovary
Pancreas
Prostate
Skin
Urinary tract
Monoallelic: SNV MNV INDEL in frame SNV nonsense MNV nonsense INDEL out of frame
Biallelic/Multihit: SNV MNV INDEL in frame SNV nonsense MNV nonsense INDEL out of frame
Hotspot:

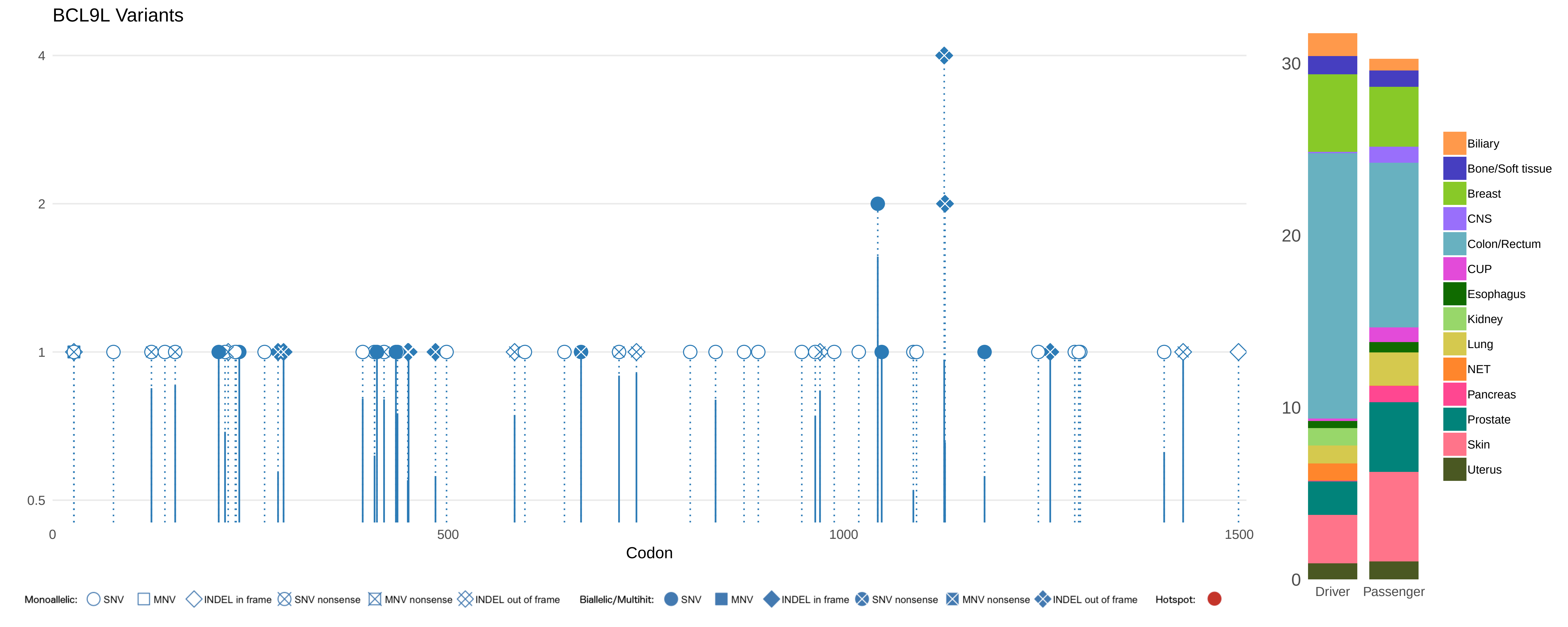
BCL9L Variants
4
2
1
0.5
0
500
1000
1500
Codon
30
20
10
0
Driver
Passenger
Biliary
Bone/Soft tissue
Breast
CNS
Colon/Rectum
CUP
Esophagus
Kidney
Lung
NET
Pancreas
Prostate
Skin
Uterus
Monoallelic: SNV MNV INDEL in frame SNV nonsense MNV nonsense INDEL out of frame
Biallelic/Multihit: SNV MNV INDEL in frame SNV nonsense MNV nonsense INDEL out of frame
Hotspot:

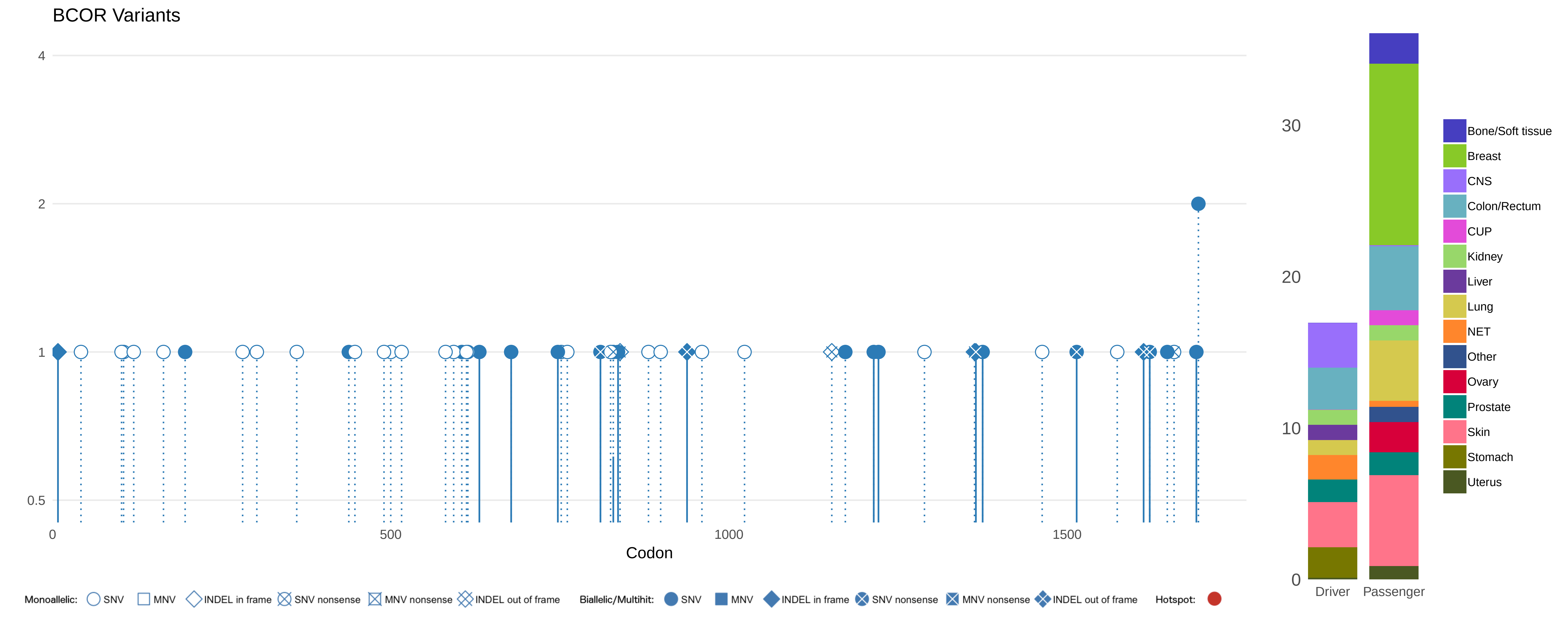
BCOR Variants
4
2
1
0.5
0
500
1000
1500
Codon
Monoallelic: SNV MNV INDEL in frame SNV nonsense MNV nonsense INDEL out of frame
Biallelic/Multihit: SNV MNV INDEL in frame SNV nonsense MNV nonsense INDEL out of frame
Hotspot:
30
20
10
0
Driver
Passenger
Bone/Soft tissue
Breast
CNS
Colon/Rectum
CUP
Kidney
Liver
Lung
NET
Other
Ovary
Prostate
Skin
Stomach
Uterus

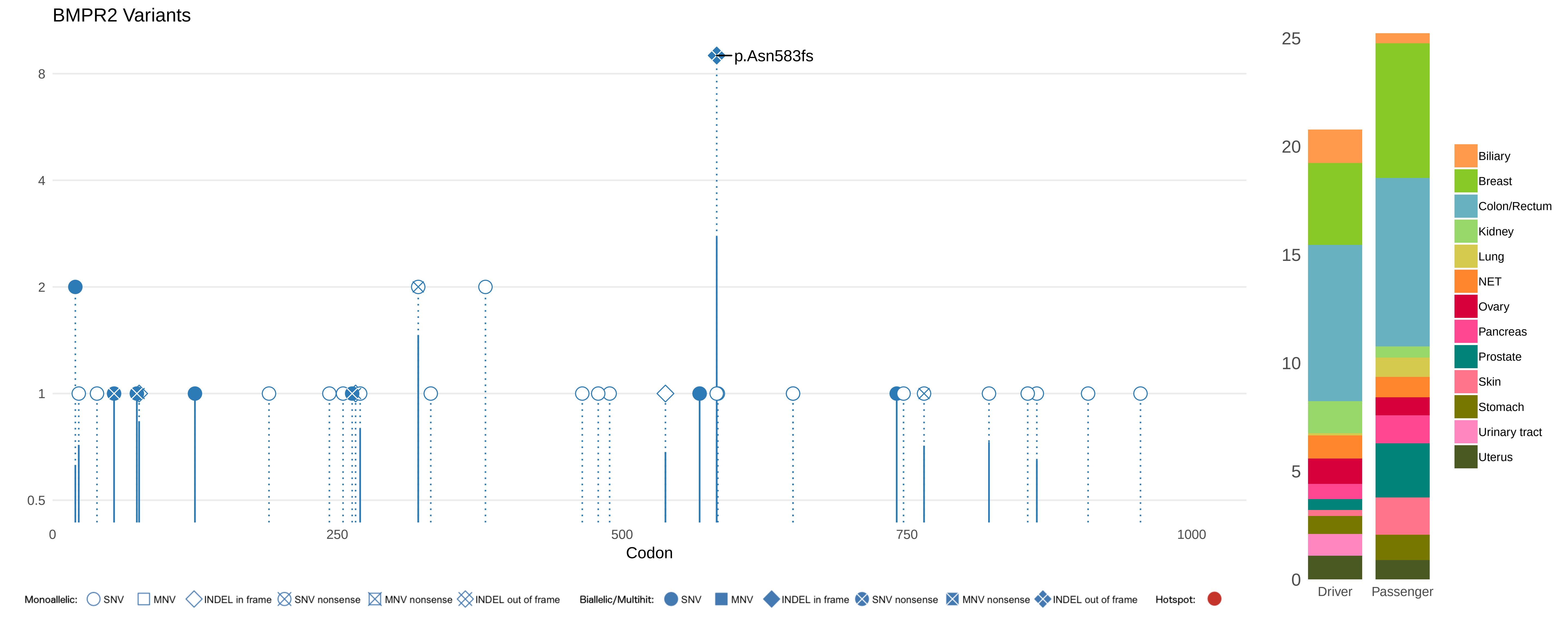

BMPR2 Variants
p.Asn583fs
8
4
2
1
0.5
0
250
500
750
1000
Codon
25
20
15
10
5
0
Driver
Passenger
Biliary
Breast
Colon/Rectum
Kidney
Lung
NET
Ovary
Pancreas
Prostate
Skin
Stomach
Urinary tract
Uterus
Monoallelic: SNV MNV INDEL in frame SNV nonsense MNV nonsense INDEL out of frame
Biallelic/Multihit: SNV MNV INDEL in frame SNV nonsense MNV nonsense INDEL out of frame
Hotspot:

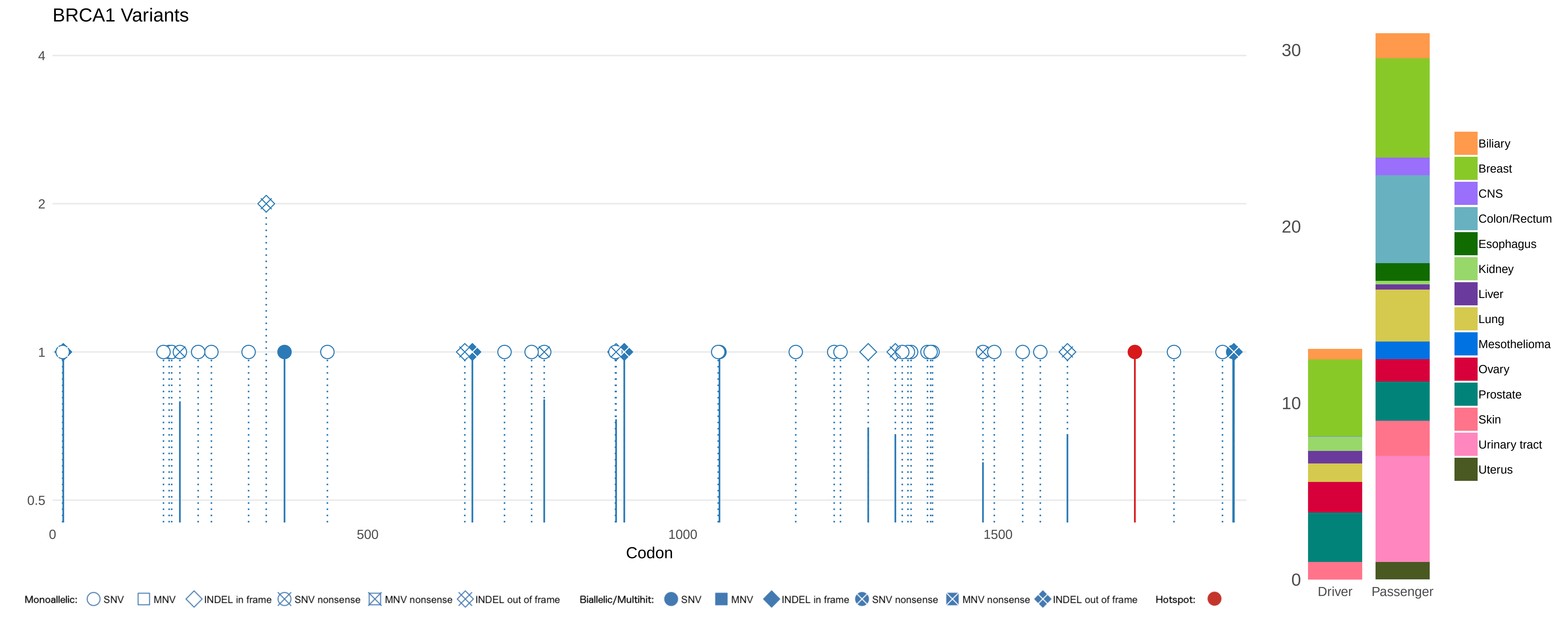
BRCA1 Variants
4
2
1
0.5
0
500
1000
1500
Codon
30
20
10
0
Driver
Passenger
Biliary
Breast
CNS
Colon/Rectum
Esophagus
Kidney
Liver
Lung
Mesothelioma
Ovary
Prostate
Skin
Urinary tract
Uterus
Monoallelic: SNV MNV INDEL in frame SNV nonsense MNV nonsense INDEL out of frame
Biallelic/Multihit: SNV MNV INDEL in frame SNV nonsense MNV nonsense INDEL out of frame
Hotspot:

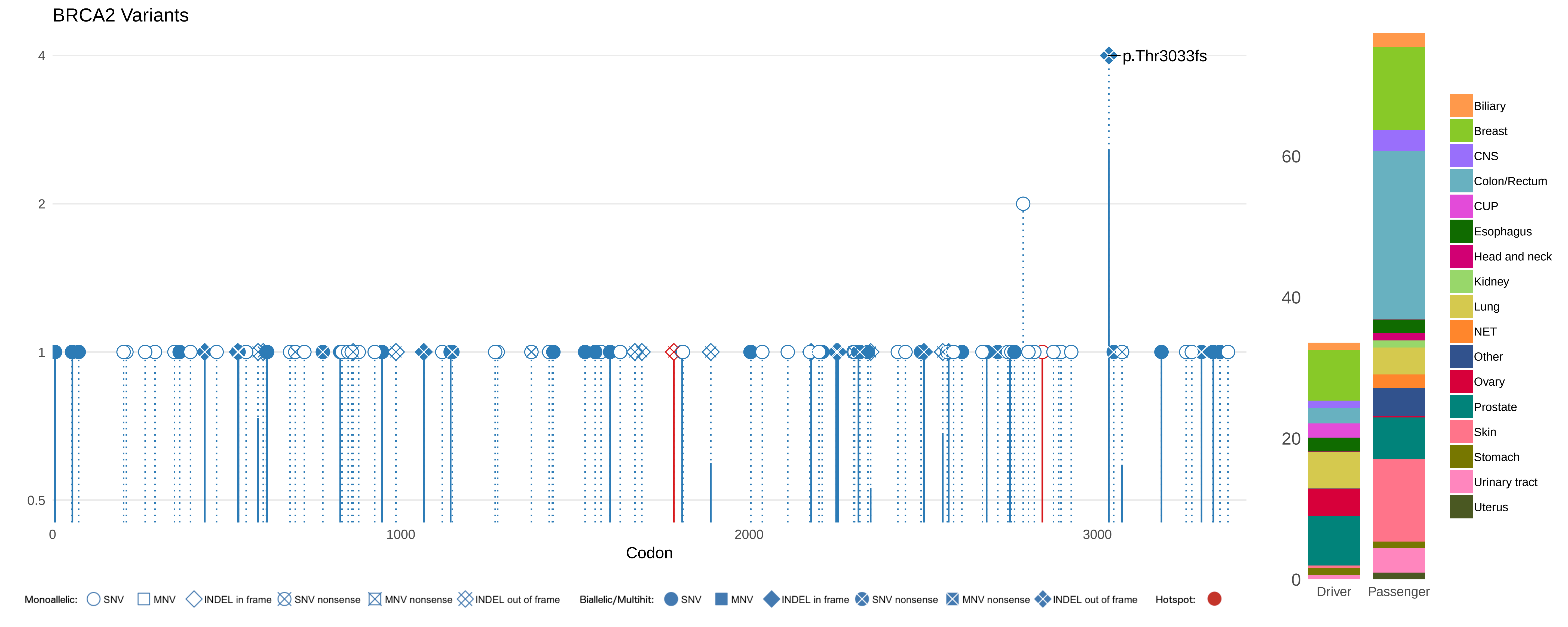

BRCA2 Variants
p.Thr3033fs
4
2
1
0.5
0
1000
2000
3000
Codon
60
40
20
0
Driver
Passenger
Biliary
Breast
CNS
Colon/Rectum
CUP
Esophagus
Head and neck
Kidney
Lung
NET
Other
Ovary
Prostate
Skin
Stomach
Urinary tract
Uterus
Monoallelic: SNV MNV INDEL in frame SNV nonsense MNV nonsense INDEL out of frame
Biallelic/Multihit: SNV MNV INDEL in frame SNV nonsense MNV nonsense INDEL out of frame
Hotspot:

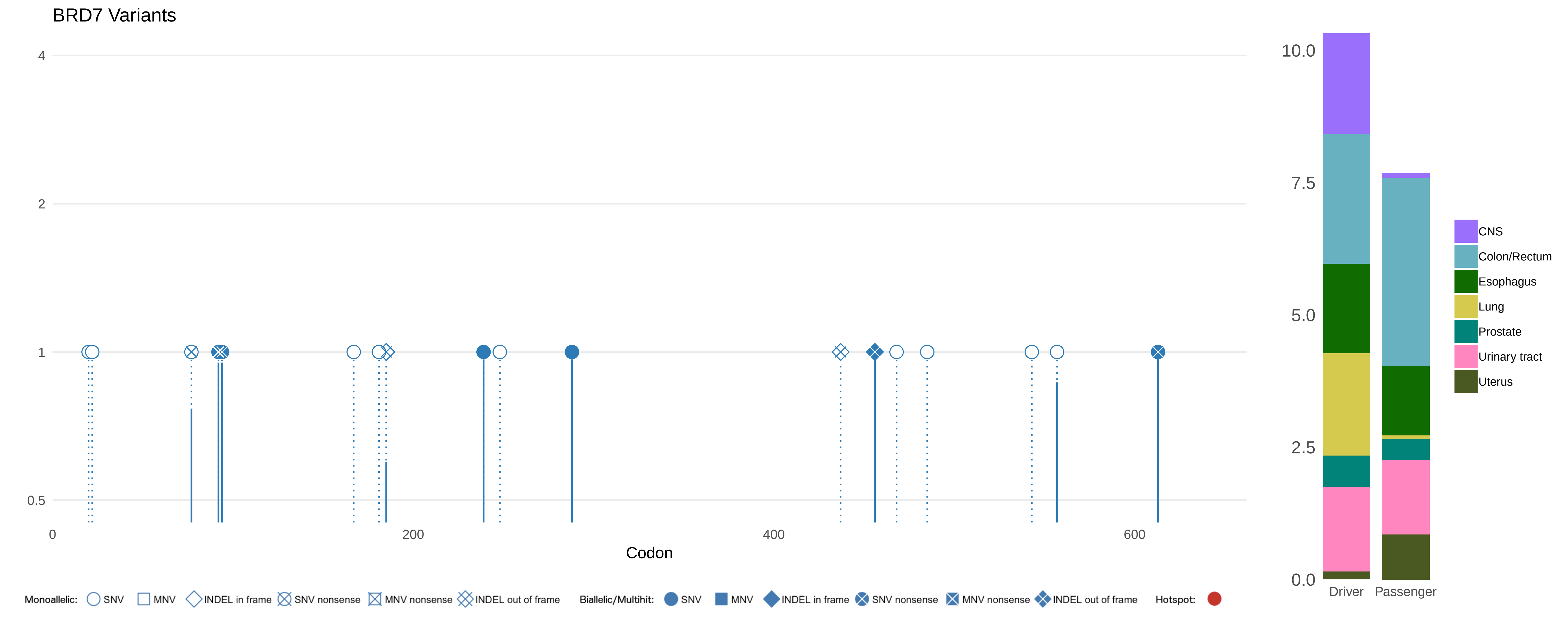

BRD7 Variants
4
2
1
0.5
0
200
400
600
Codon
10.0
7.5
5.0
2.5
0.0
Driver
Passenger
CNS
Colon/Rectum
Esophagus
Lung
Prostate
Urinary tract
Uterus
Monoallelic: SNV MNV INDEL in frame SNV nonsense MNV nonsense INDEL out of frame
Biallelic/Multihit: SNV MNV INDEL in frame SNV nonsense MNV nonsense INDEL out of frame
Hotspot:

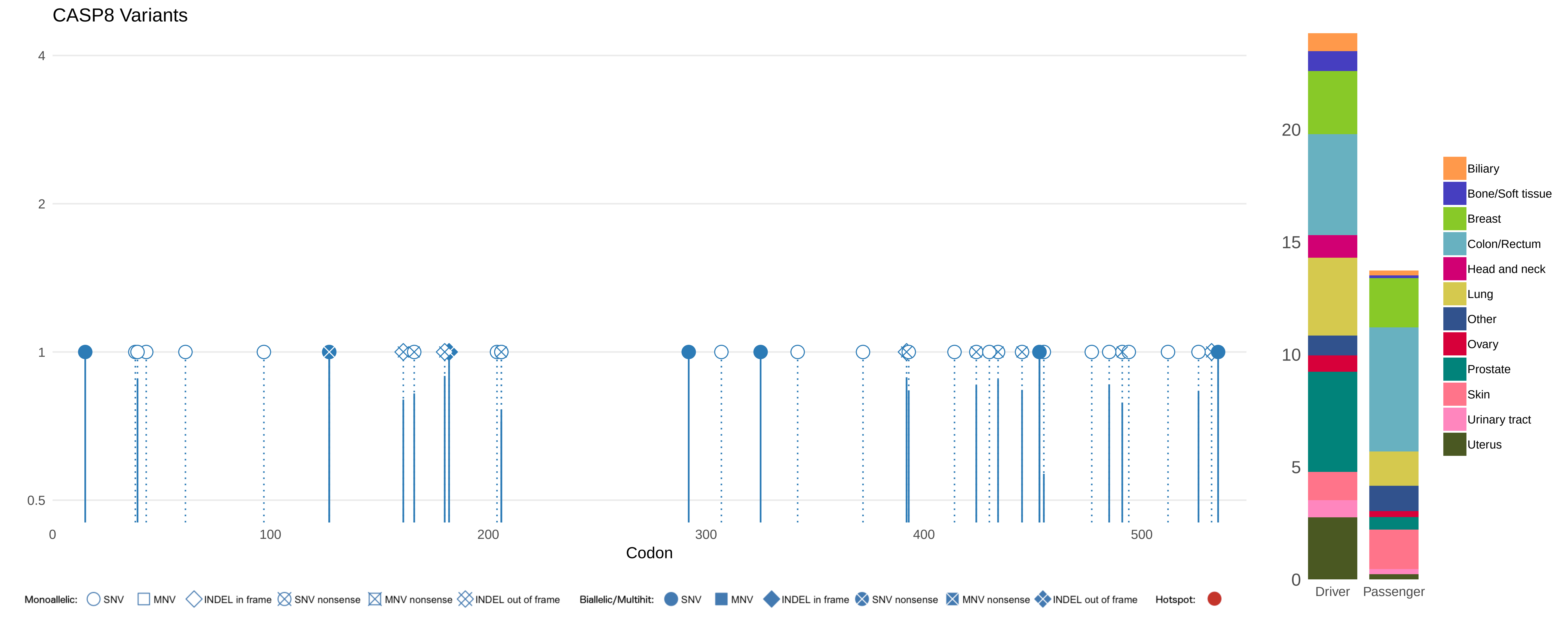
CASP8 Variants
4
2
1
0.5
0
100
200
300
400
500
Codon
20
15
10
5
0
Driver
Passenger
Biliary
Bone/Soft tissue
Breast
Colon/Rectum
Head and neck
Lung
Other
Ovary
Prostate
Skin
Urinary tract
Uterus
Monoallelic: SNV MNV INDEL in frame SNV nonsense MNV nonsense INDEL out of frame
Biallelic/Multihit: SNV MNV INDEL in frame SNV nonsense MNV nonsense INDEL out of frame
Hotspot:

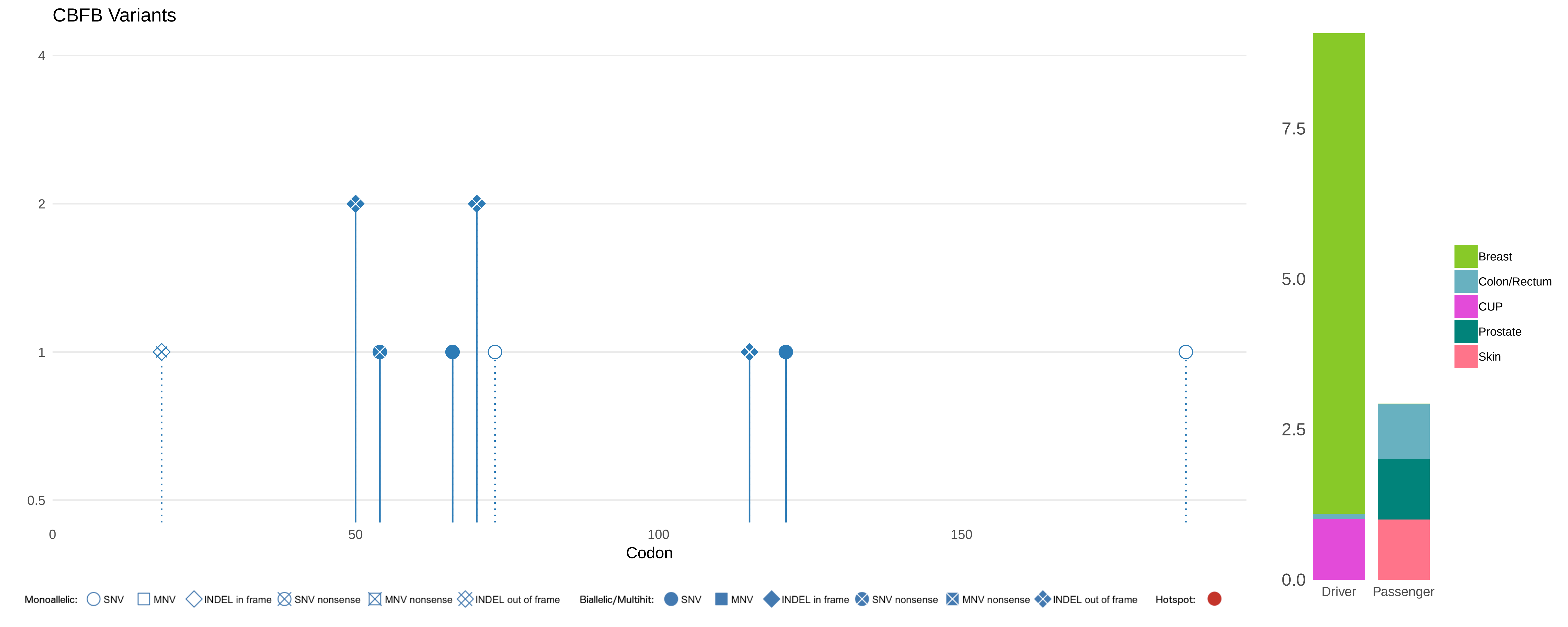
CBFB Variants
4
2
1
0.5
0
50
100
150
Codon
7.5
5.0
2.5
0.0
Driver
Passenger
Breast
Colon/Rectum
CUP
Prostate
Skin
Monoallelic: SNV MNV INDEL in frame SNV nonsense MNV nonsense INDEL out of frame
Biallelic/Multihit: SNV MNV INDEL in frame SNV nonsense MNV nonsense INDEL out of frame
Hotspot:

CBL Variants

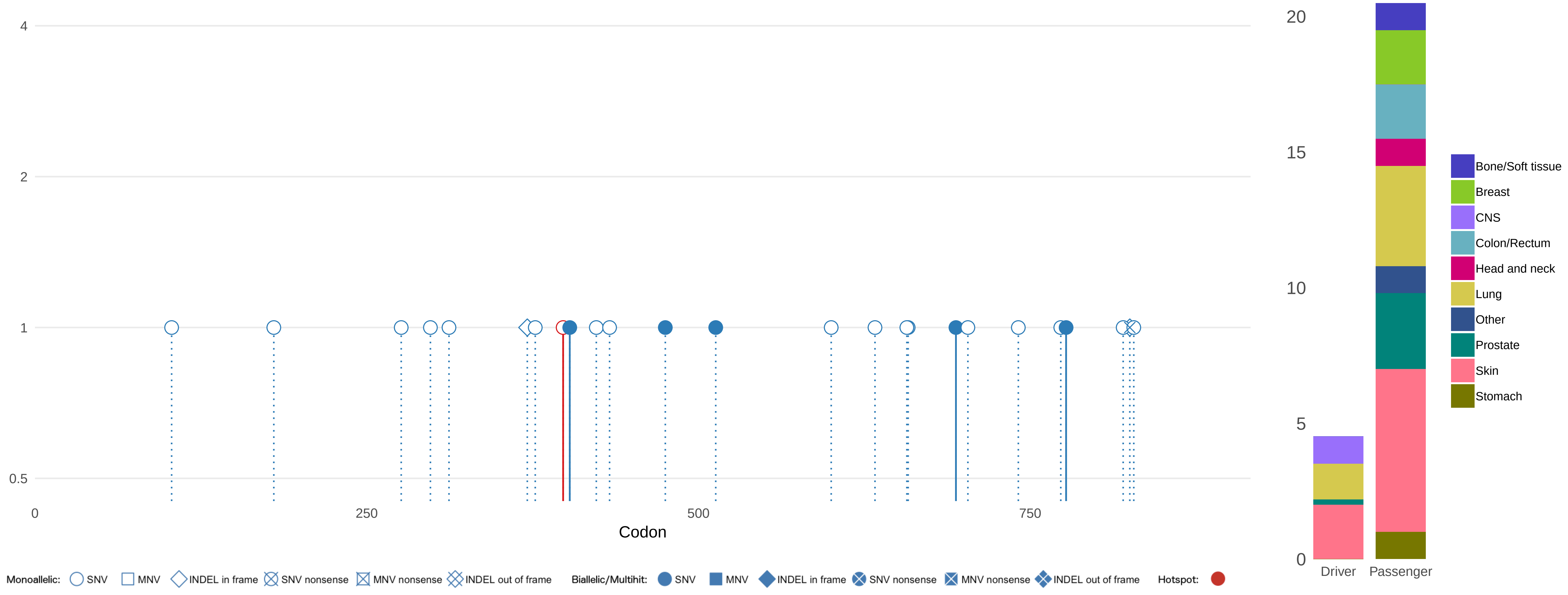

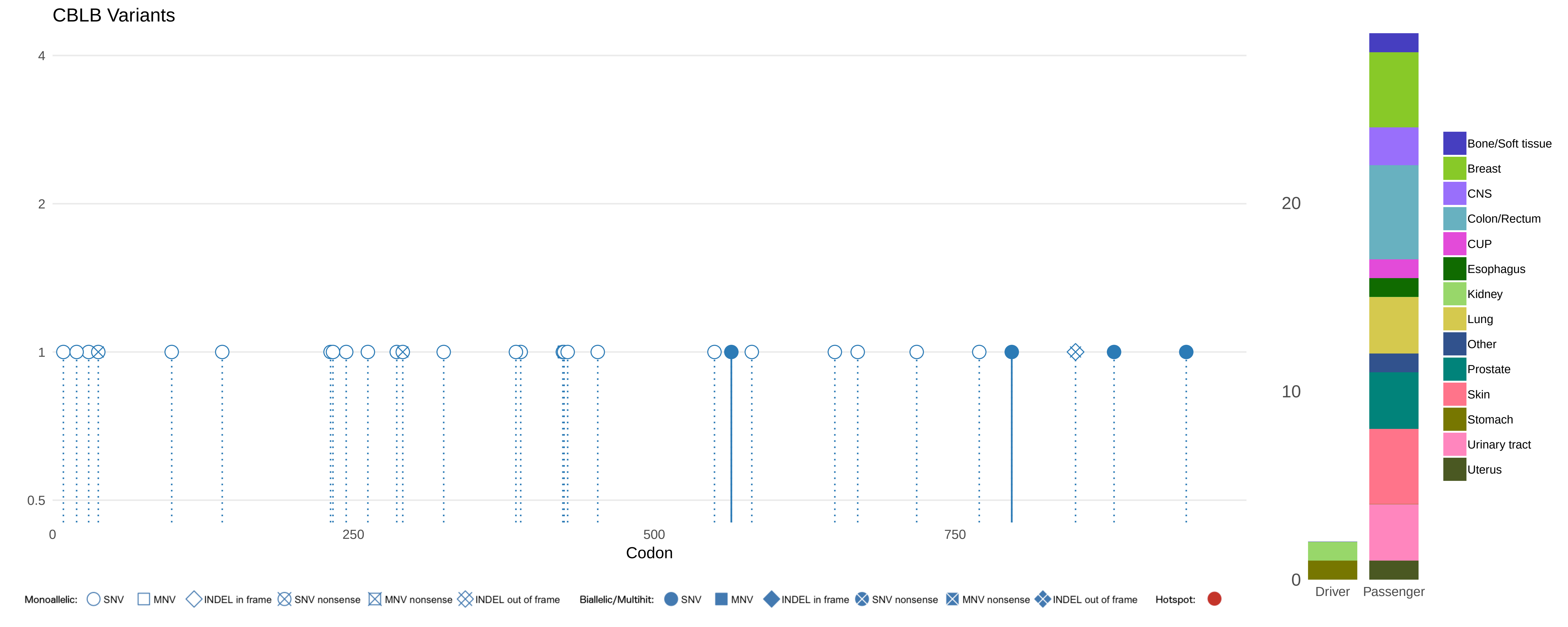
CBLB Variants
4
2
1
0.5
0
250
500
750
Codon
20
10
0
Driver
Passenger
Bone/Soft tissue
Breast
CNS
Colon/Rectum
CUP
Esophagus
Kidney
Lung
Other
Prostate
Skin
Stomach
Urinary tract
Uterus
Monoallelic: SNV MNV INDEL in frame SNV nonsense MNV nonsense INDEL out of frame
Biallelic/Multihit: SNV MNV INDEL in frame SNV nonsense MNV nonsense INDEL out of frame
Hotspot:

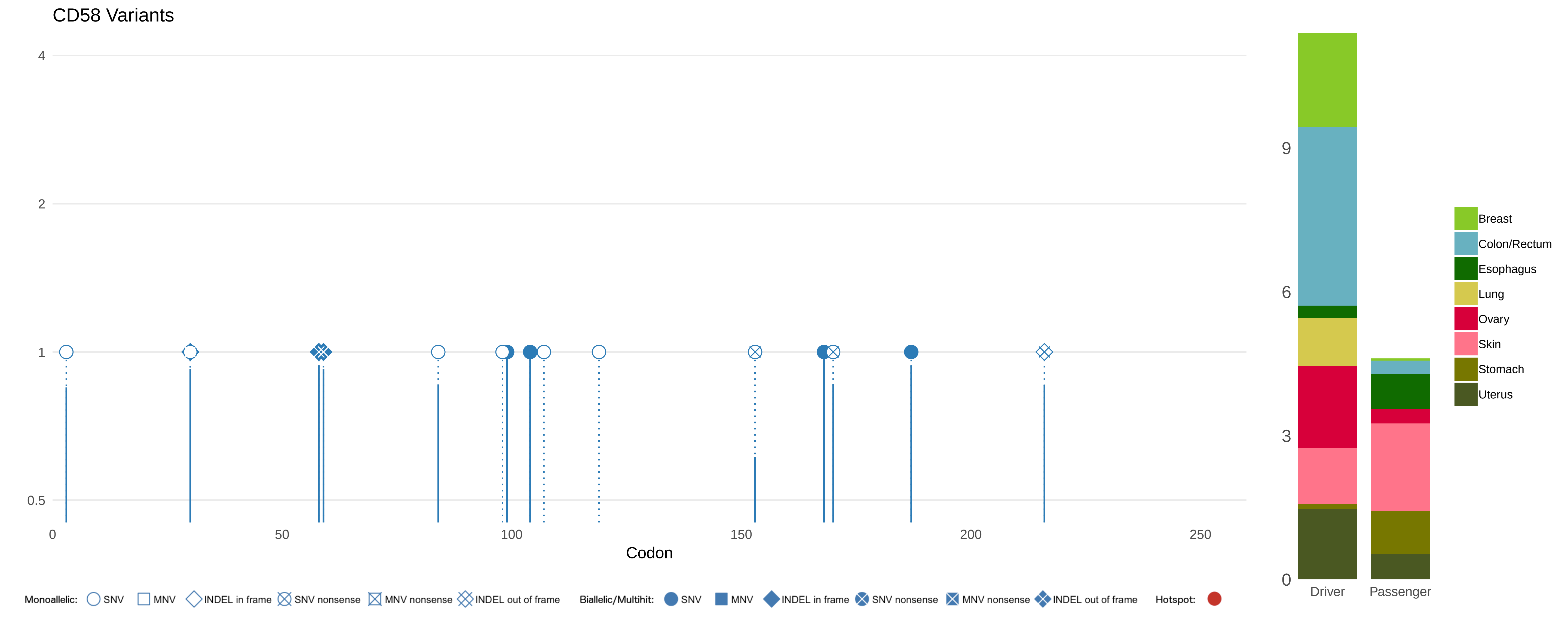

CD58 Variants
4
2
1
0.5
0
50
100
150
200
250
Codon
9
6
3
0
Driver
Passenger
Breast
Colon/Rectum
Esophagus
Lung
Ovary
Skin
Stomach
Uterus
Monoallelic: SNV MNV INDEL in frame SNV nonsense MNV nonsense INDEL out of frame
Biallelic/Multihit: SNV MNV INDEL in frame SNV nonsense MNV nonsense INDEL out of frame
Hotspot:

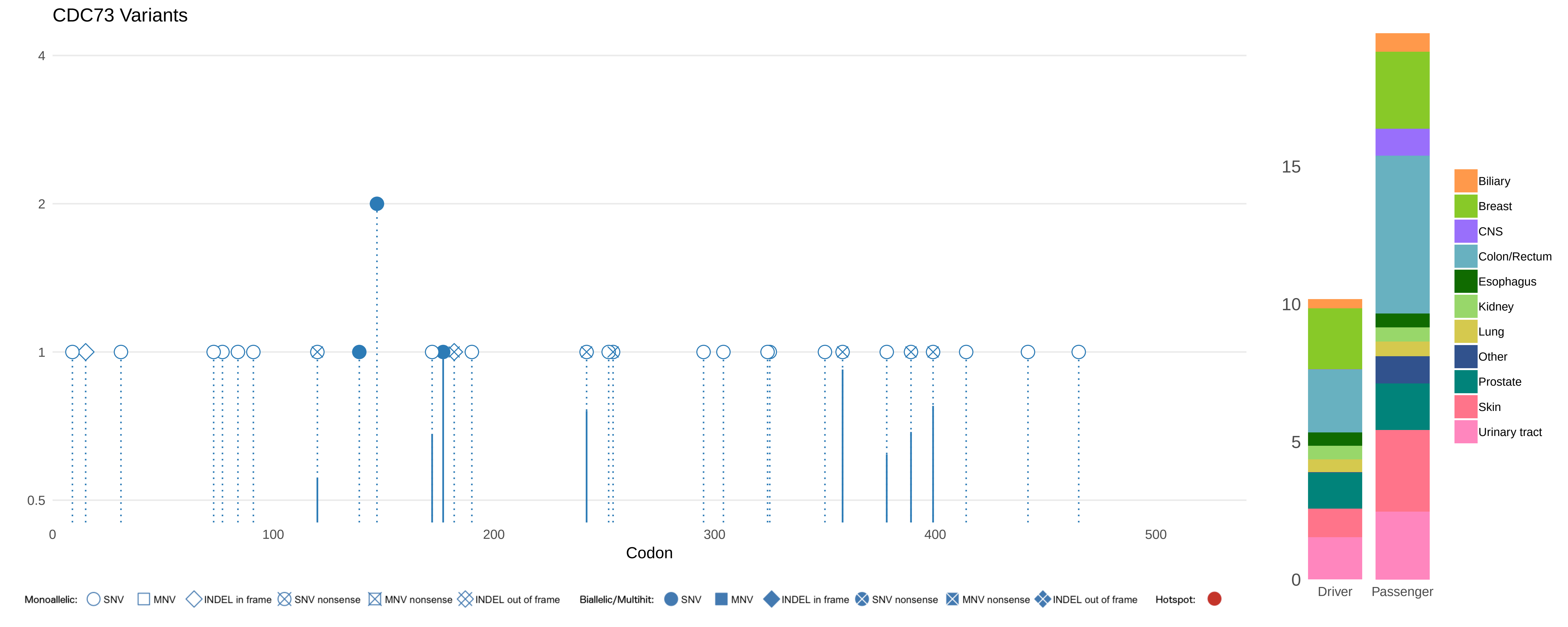
CDC73 Variants
4
2
1
0.5
0
100
200
300
400
500
Codon
Monoallelic: SNV MNV INDEL in frame SNV nonsense MNV nonsense INDEL out of frame
Biallelic/Multihit: SNV MNV INDEL in frame SNV nonsense MNV nonsense INDEL out of frame
Hotspot:
15
10
5
0
Driver
Passenger
Biliary
Breast
CNS
Colon/Rectum
Esophagus
Kidney
Lung
Other
Prostate
Skin
Urinary tract

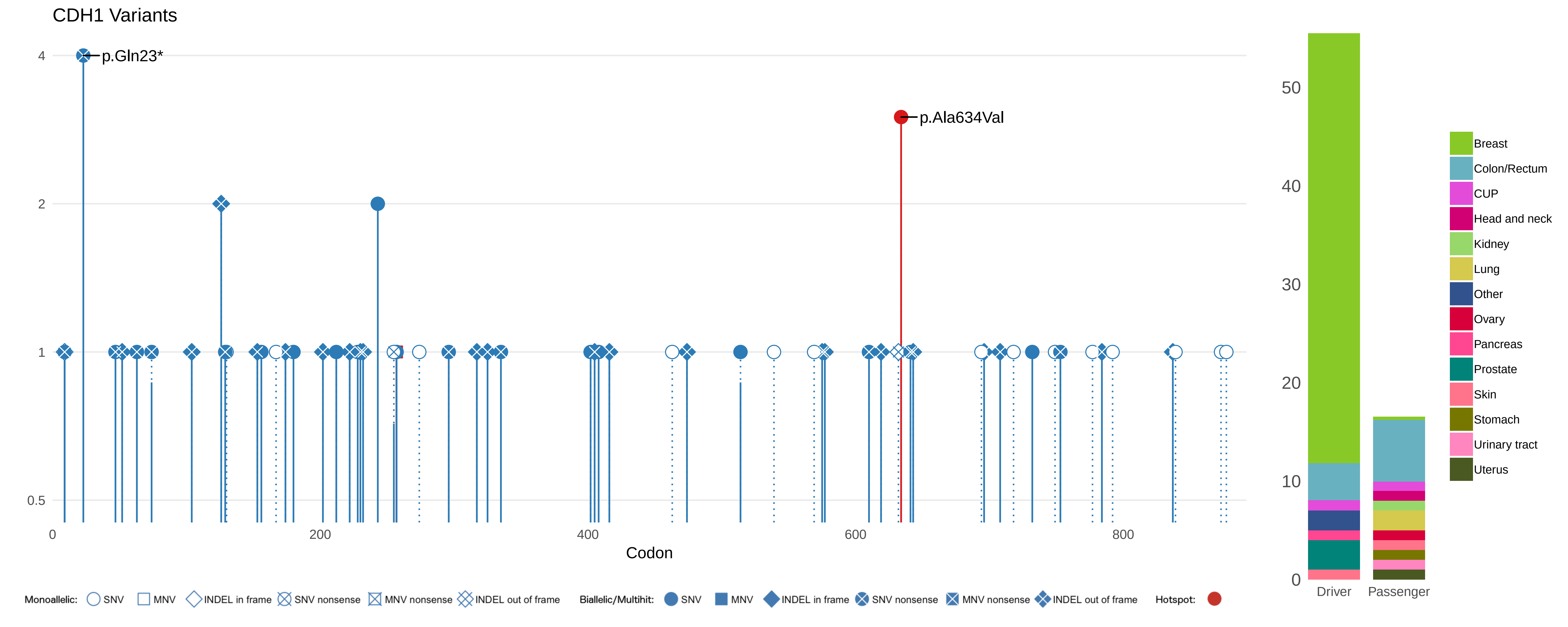
CDH1 Variants
p.Gln23*
p.Ala634Val
4
2
1
0.5
0
200
400
600
800
Codon
Monoallelic: SNV MNV INDEL in frame SNV nonsense MNV nonsense INDEL out of frame
Biallelic/Multihit: SNV MNV INDEL in frame SNV nonsense MNV nonsense INDEL out of frame
Hotspot:
50
40
30
20
10
0
Driver
Passenger
Breast
Colon/Rectum
CUP
Head and neck
Kidney
Lung
Other
Ovary
Pancreas
Prostate
Skin
Stomach
Urinary tract
Uterus

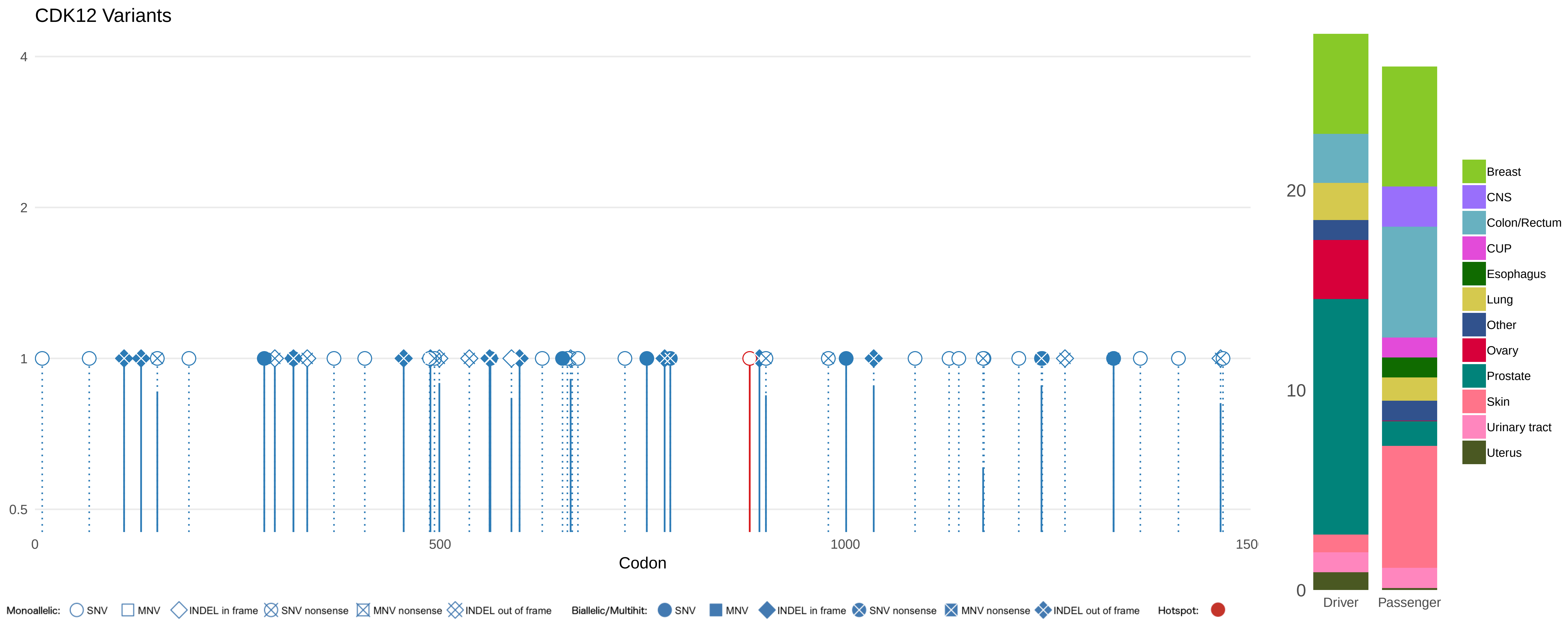
CDK12 Variants
4
2
1
0.5
0
500
1000
150
Codon
20
10
0
Driver
Passenger
Breast
CNS
Colon/Rectum
CUP
Esophagus
Lung
Other
Ovary
Prostate
Skin
Urinary tract
Uterus
Monoallelic: SNV MNV INDEL in frame SNV nonsense MNV nonsense INDEL out of frame
Biallelic/Multihit: SNV MNV INDEL in frame SNV nonsense MNV nonsense INDEL out of frame
Hotspot:

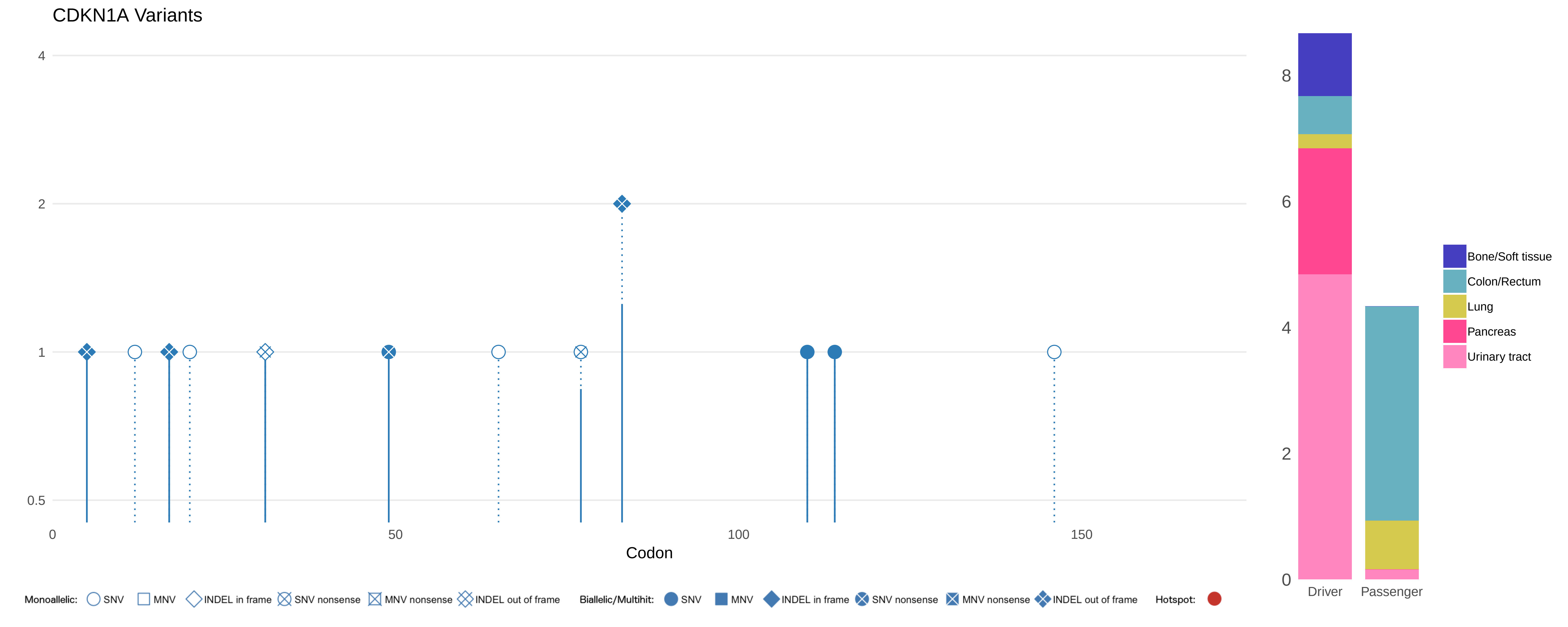
CDKN1A Variants
4
2
1
0.5
0
50
100
150
Codon
Monoallelic: SNV MNV INDEL in frame SNV nonsense MNV nonsense INDEL out of frame
Biallelic/Multihit: SNV MNV INDEL in frame SNV nonsense MNV nonsense INDEL out of frame
Hotspot:
8
6
4
2
0
Driver
Passenger
Bone/Soft tissue
Colon/Rectum
Lung
Pancreas
Urinary tract

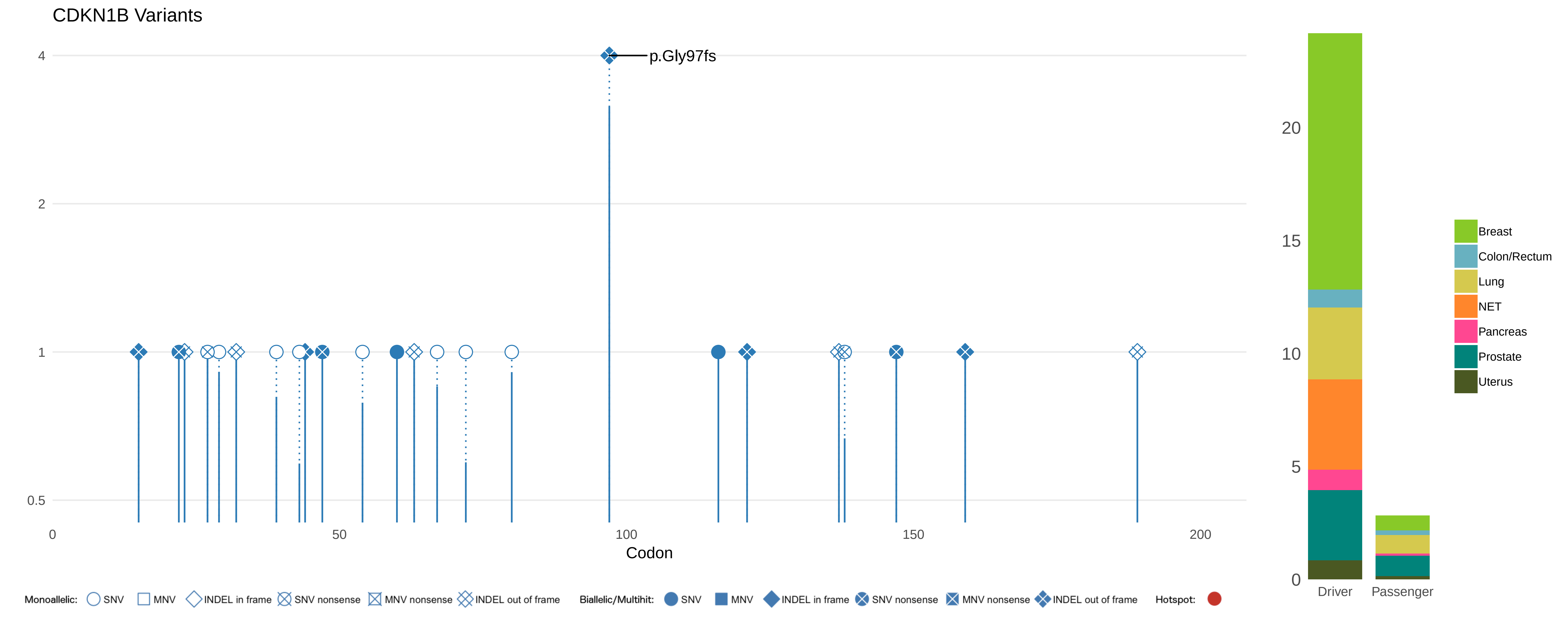
CDKN1B Variants
p.Gly97fs
4
2
1
0.5
0
50
100
150
200
Codon
20
15
10
5
0
Driver
Passenger
Breast
Colon/Rectum
Lung
NET
Pancreas
Prostate
Uterus
Monoallelic: SNV MNV INDEL in frame SNV nonsense MNV nonsense INDEL out of frame
Biallelic/Multihit: SNV MNV INDEL in frame SNV nonsense MNV nonsense INDEL out of frame
Hotspot:

CDKN2A Variants

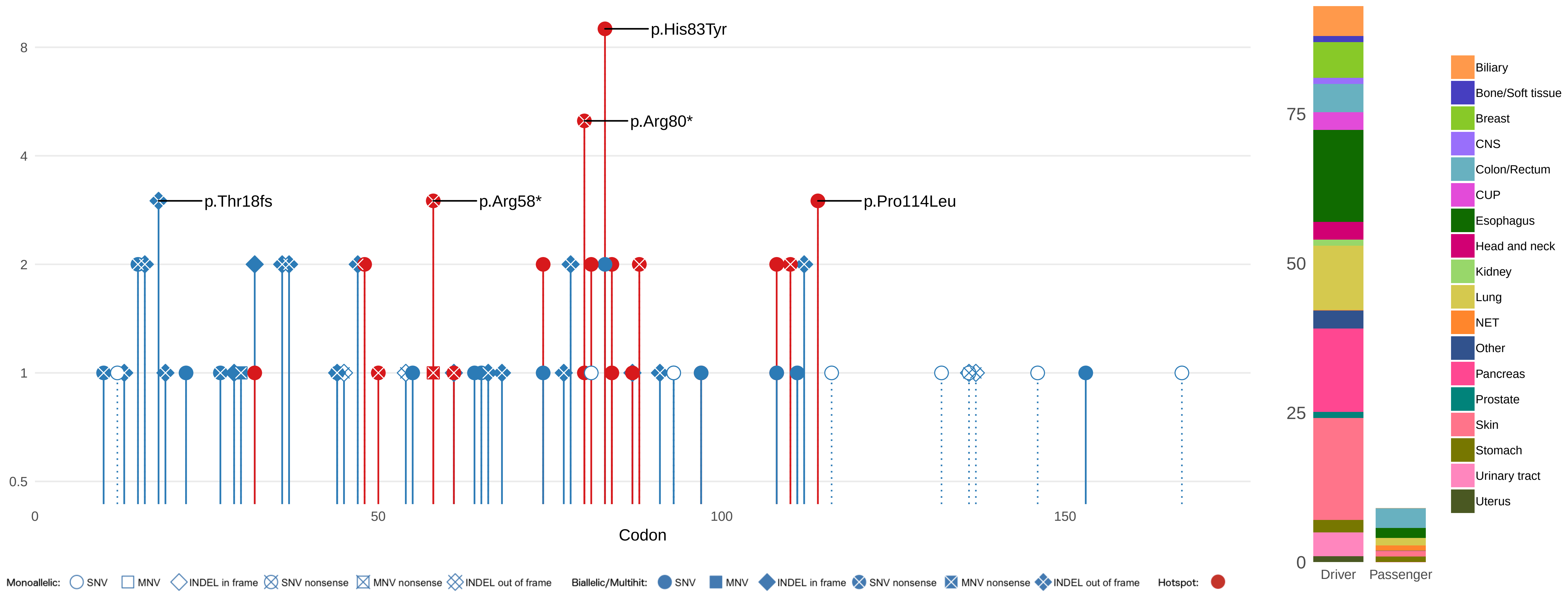

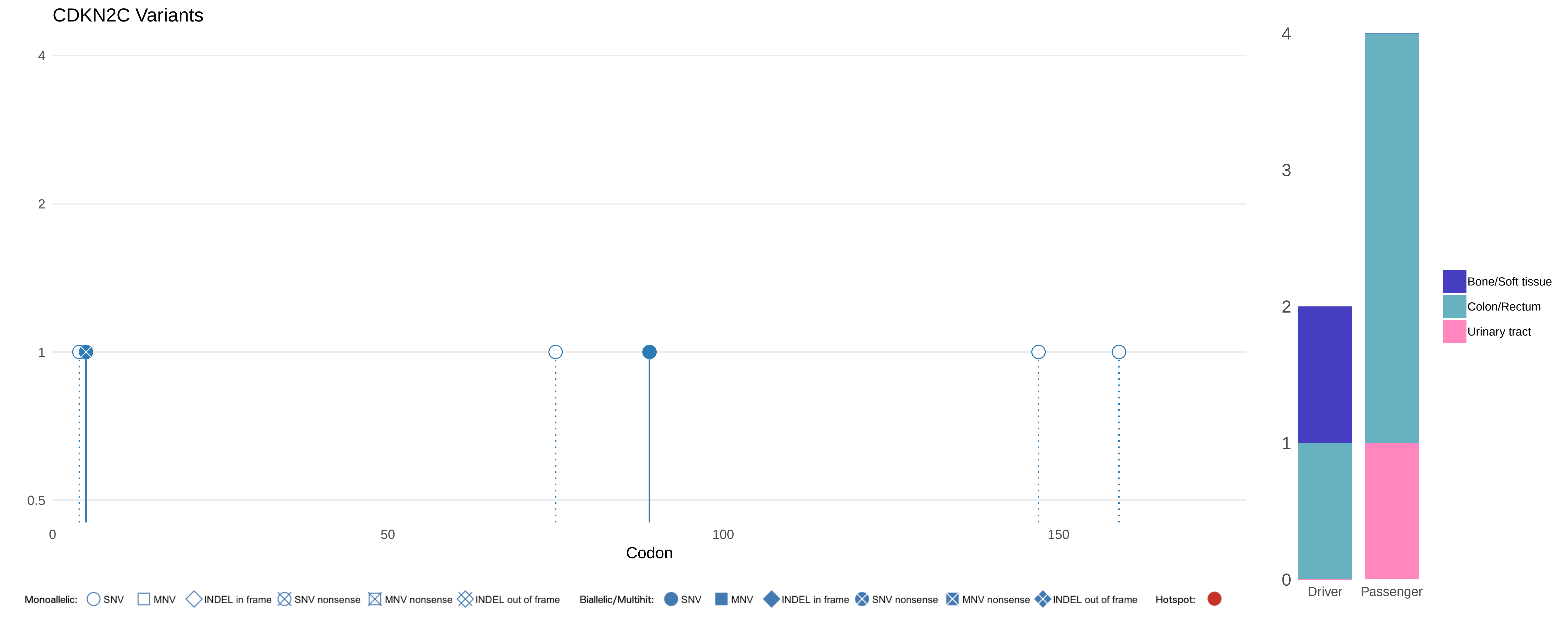
CDKN2C Variants
4
2
1
0.5
0
50
100
150
Codon
4
3
2
1
0
Driver
Passenger
Bone/Soft tissue
Colon/Rectum
Urinary tract
Monoallelic: SNV MNV INDEL in frame SNV nonsense MNV nonsense INDEL out of frame
Biallelic/Multihit: SNV MNV INDEL in frame SNV nonsense MNV nonsense INDEL out of frame
Hotspot:

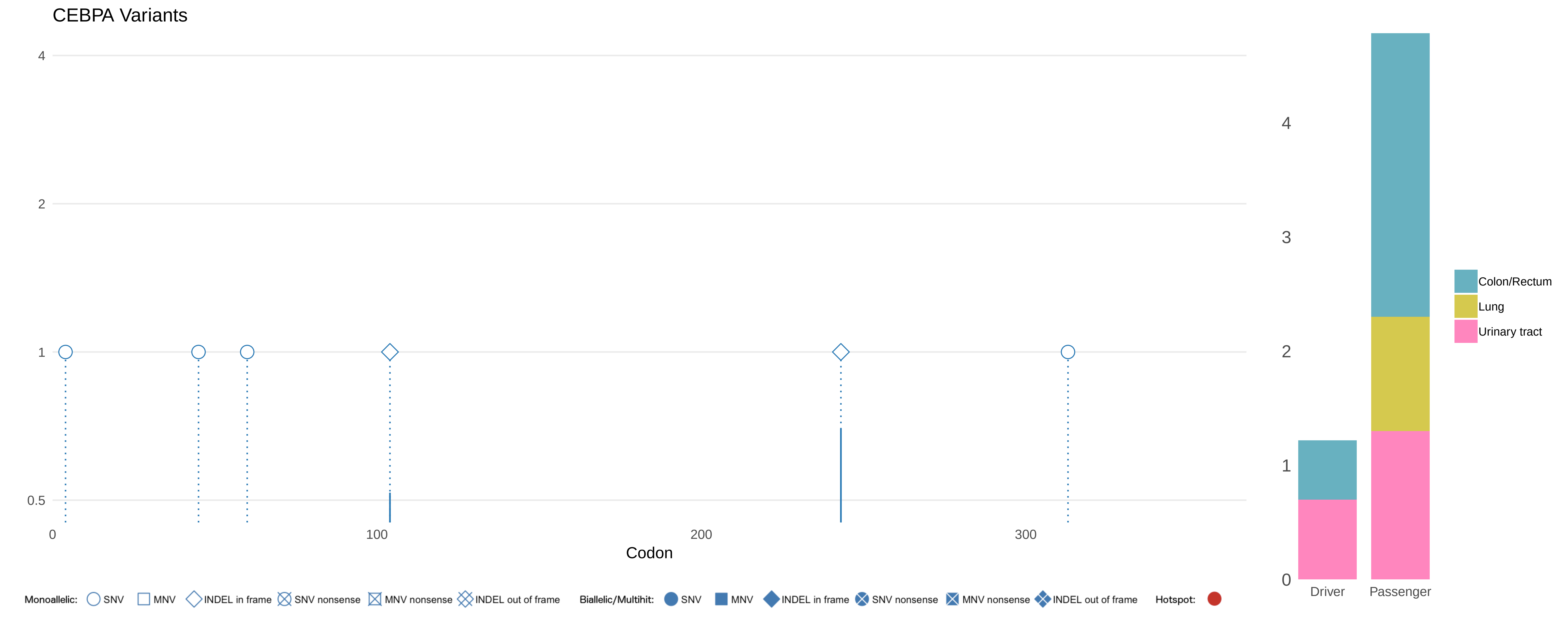

CEBPA Variants
4
2
1
0.5
0
100
200
300
Codon
Monoallelic: SNV MNV INDEL in frame SNV nonsense MNV nonsense INDEL out of frame
Biallelic/Multihit: SNV MNV INDEL in frame SNV nonsense MNV nonsense INDEL out of frame
Hotspot:
4
3
2
1
0
Driver
Passenger
Colon/Rectum
Lung
Urinary tract

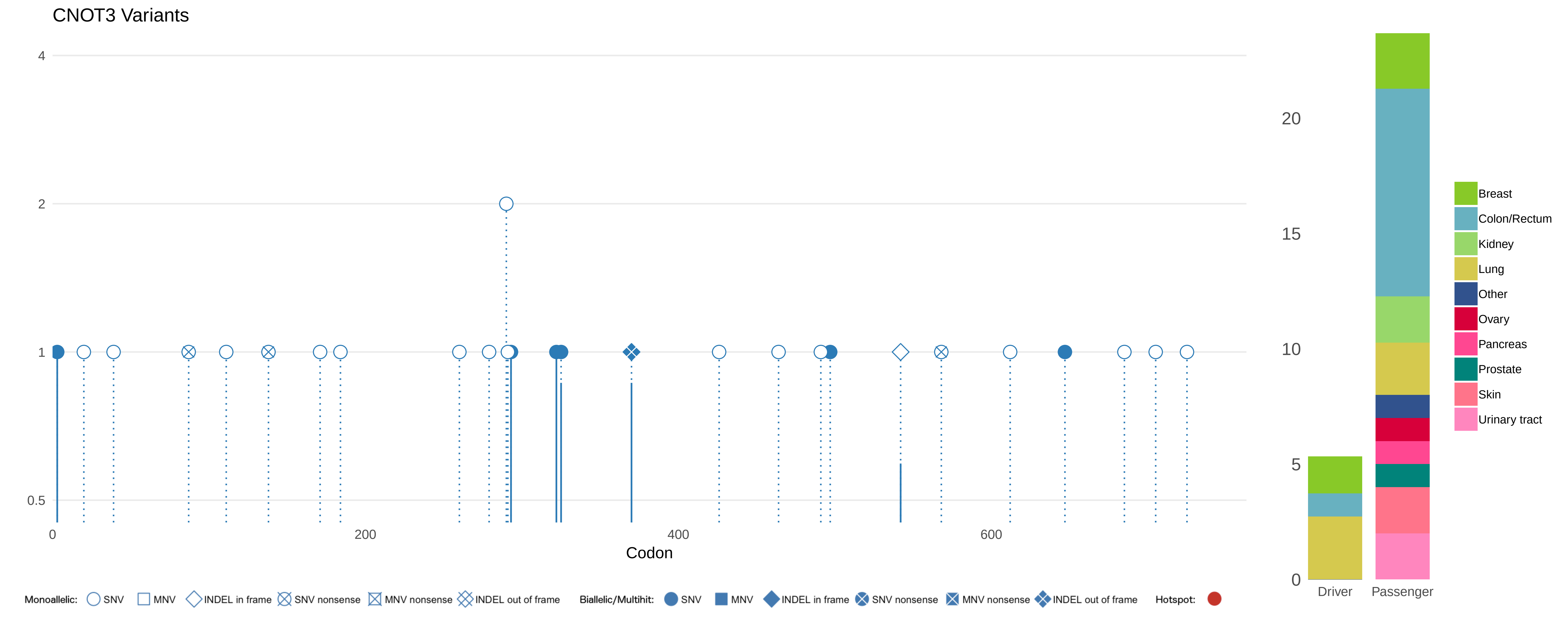

CNOT3 Variants
4
2
1
0.5
0
200
400
600
Codon
20
15
10
5
0
Driver
Passenger
Breast
Colon/Rectum
Kidney
Lung
Other
Ovary
Pancreas
Prostate
Skin
Urinary tract
Monoallelic: SNV MNV INDEL in frame SNV nonsense MNV nonsense INDEL out of frame
Biallelic/Multihit: SNV MNV INDEL in frame SNV nonsense MNV nonsense INDEL out of frame
Hotspot:

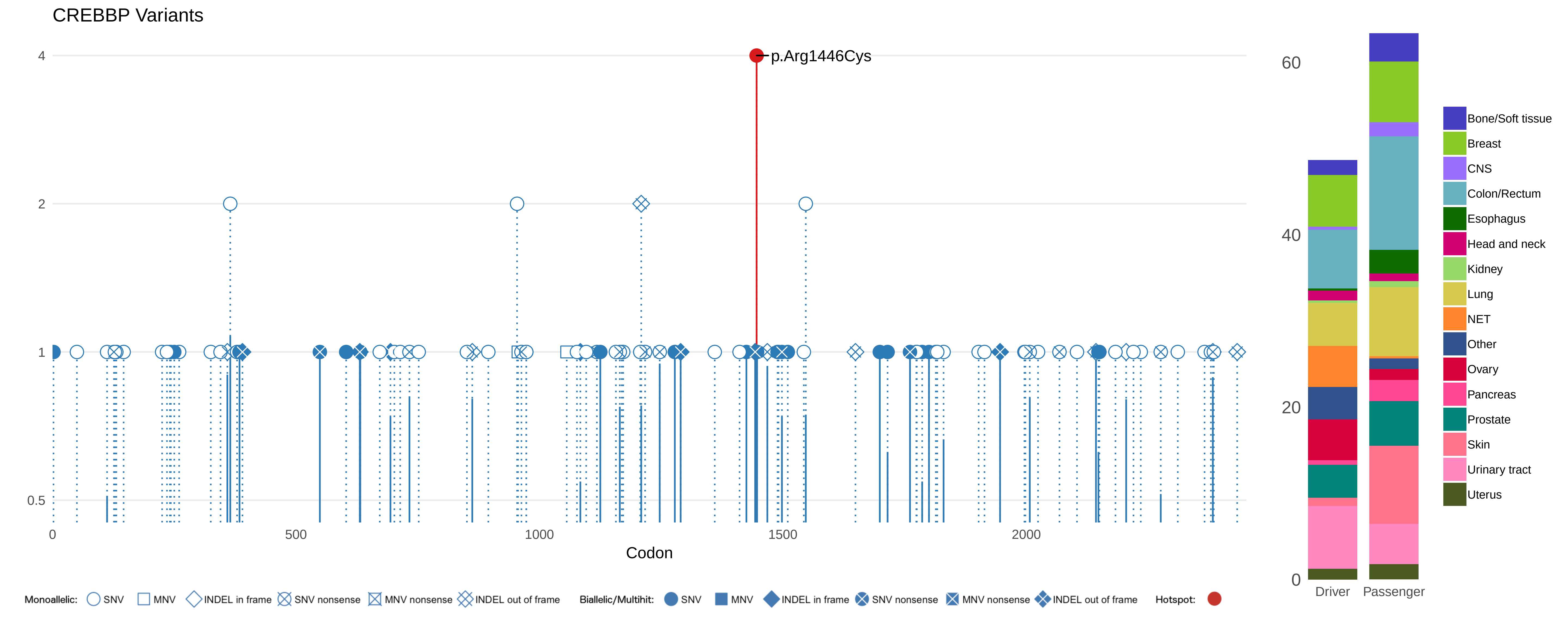
CREBBP Variants
p.Arg1446Cys
4
2
1
0.5
0
500
1000
1500
2000
Codon
Monoallelic: SNV MNV INDEL in frame SNV nonsense MNV nonsense INDEL out of frame
Biallelic/Multihit: SNV MNV INDEL in frame SNV nonsense MNV nonsense INDEL out of frame
Hotspot:
60
40
20
0
Driver
Passenger
Bone/Soft tissue
Breast
CNS
Colon/Rectum
Esophagus
Head and neck
Kidney
Lung
NET
Other
Ovary
Pancreas
Prostate
Skin
Urinary tract
Uterus

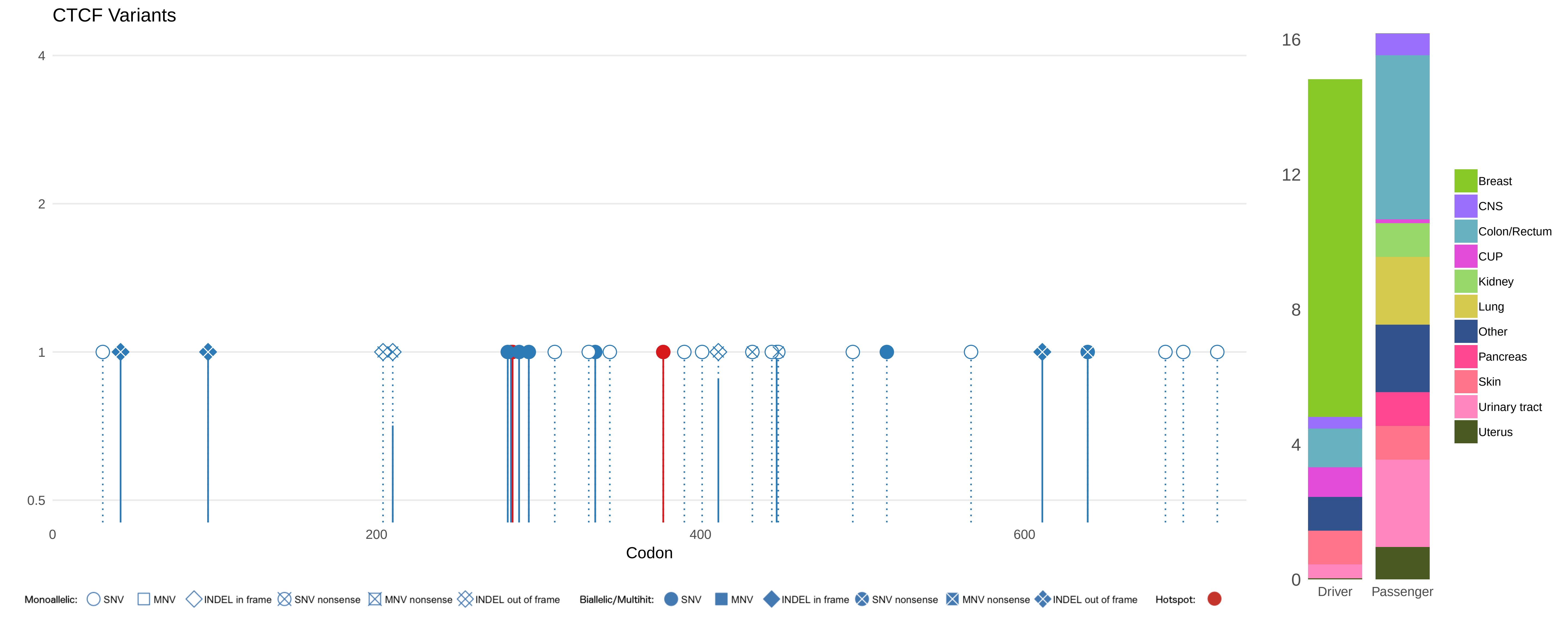

CTCF Variants
4
2
1
0.5
0
200
400
600
Codon
16
12
8
4
0
Driver
Passenger
Breast
CNS
Colon/Rectum
CUP
Kidney
Lung
Other
Pancreas
Skin
Urinary tract
Uterus
Monoallelic: SNV MNV INDEL in frame SNV nonsense MNV nonsense INDEL out of frame
Biallelic/Multihit: SNV MNV INDEL in frame SNV nonsense MNV nonsense INDEL out of frame
Hotspot:

CTNNA1 Variants

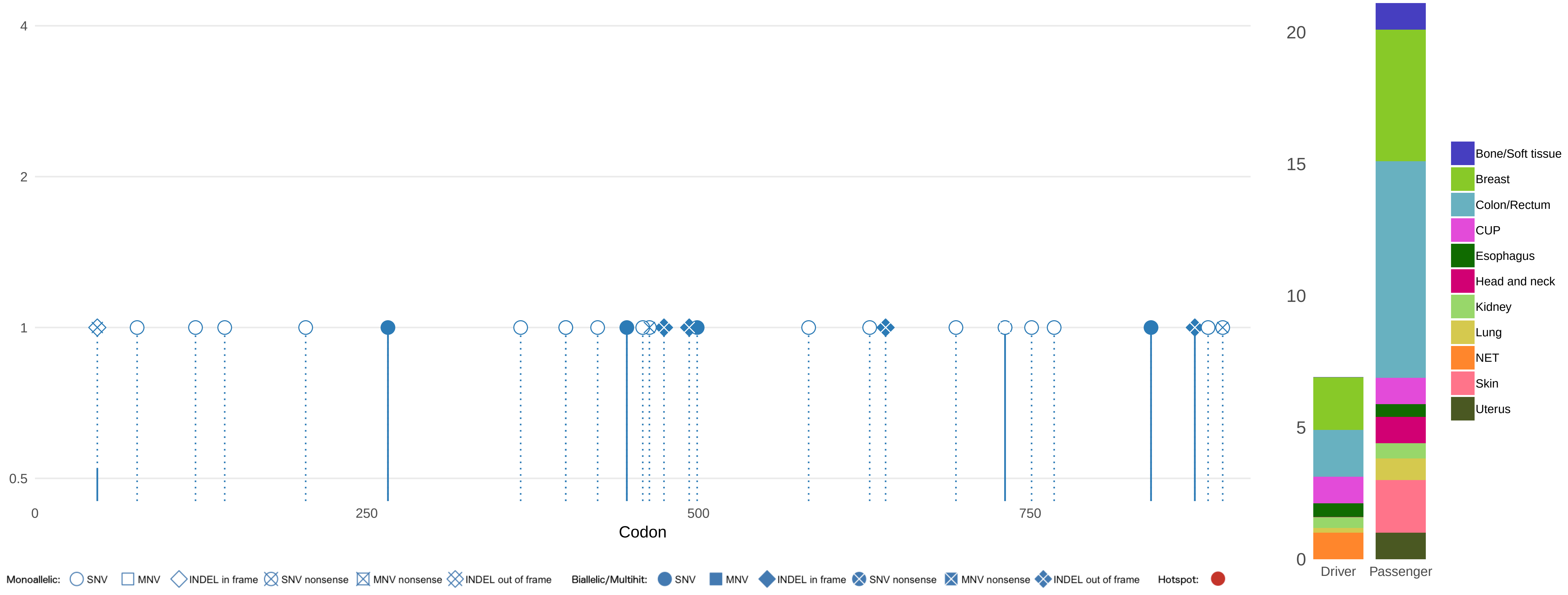

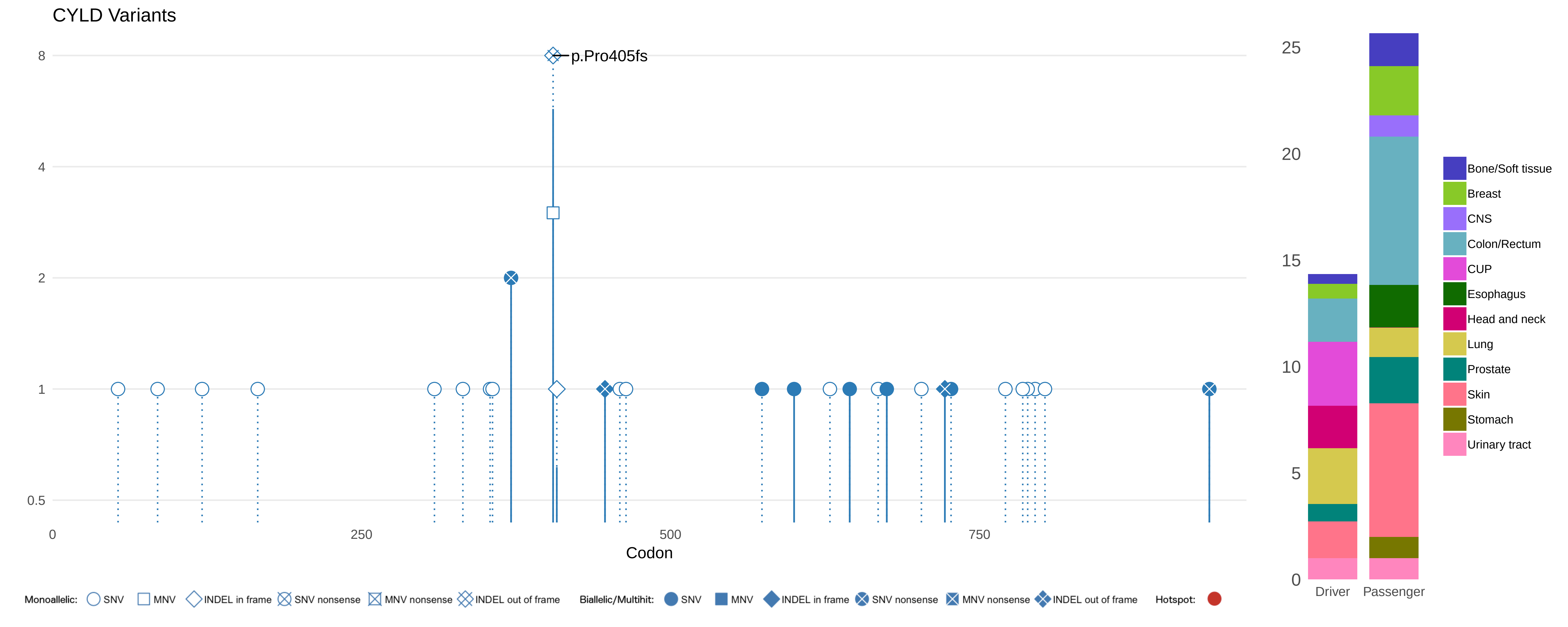

CYLD Variants
p.Pro405fs
8
4
2
1
0.5
0
250
500
750
Codon
25
20
15
10
5
0
Driver
Passenger
Bone/Soft tissue
Breast
CNS
Colon/Rectum
CUP
Esophagus
Head and neck
Lung
Prostate
Skin
Stomach
Urinary tract
Monoallelic:
SNV
MNV
INDEL in frame
SNV nonsense
MNV nonsense
INDEL out of frame
Biallelic/Multihit:
SNV
MNV
INDEL in frame
SNV nonsense
MNV nonsense
INDEL out of frame
Hotspot:

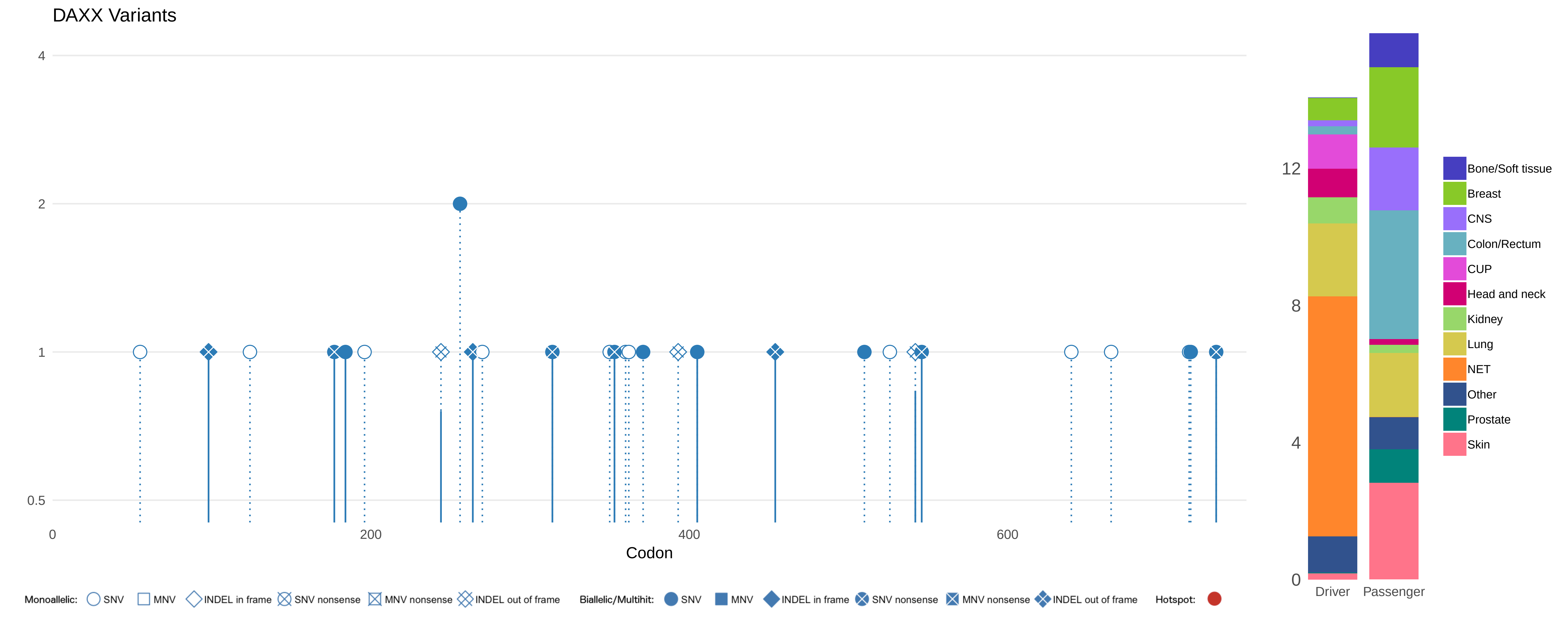
DAXX Variants
4
2
1
0.5
0
200
400
600
Codon
Monoallelic: SNV MNV INDEL in frame SNV nonsense MNV nonsense INDEL out of frame
Biallelic/Multihit: SNV MNV INDEL in frame SNV nonsense MNV nonsense INDEL out of frame
Hotspot:
12
8
4
0
Driver
Passenger
Bone/Soft tissue
Breast
CNS
Colon/Rectum
CUP
Head and neck
Kidney
Lung
NET
Other
Prostate
Skin

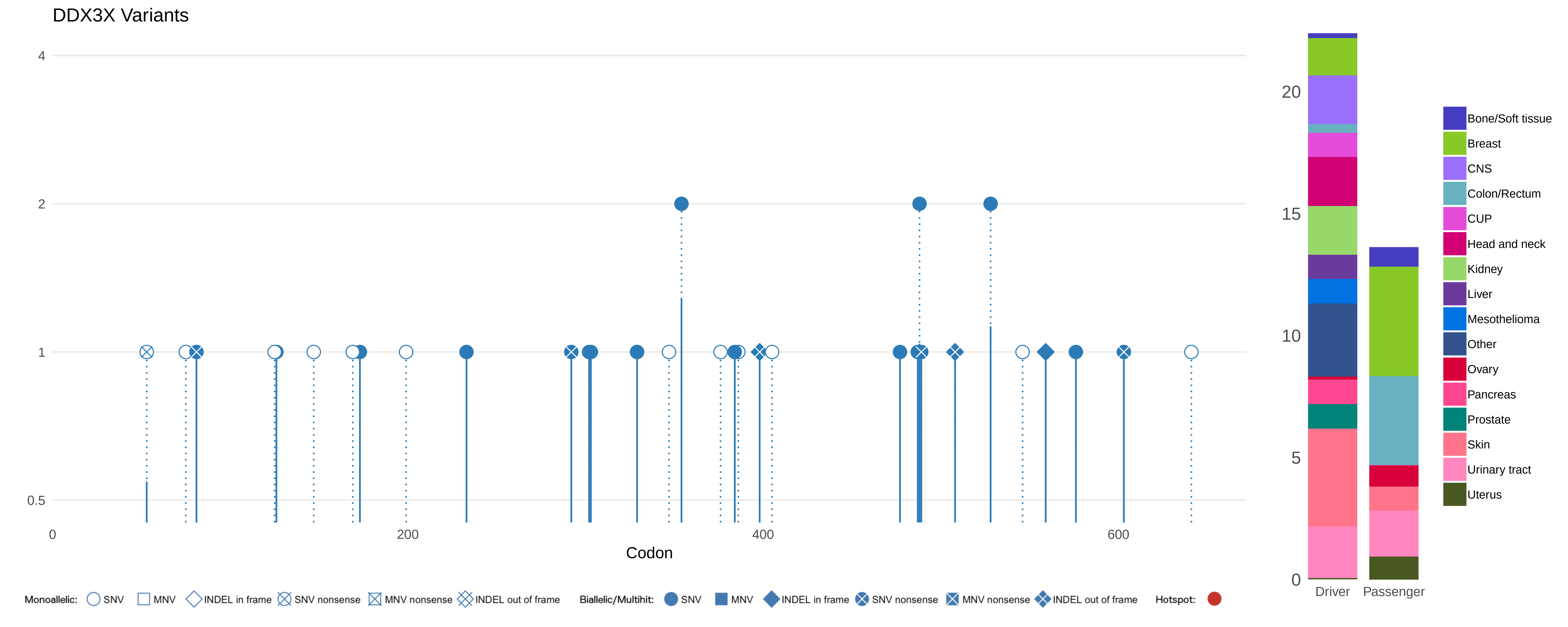
DDX3X Variants
4
2
1
0.5
0
200
400
600
Codon
20
15
10
5
0
Driver
Passenger
Bone/Soft tissue
Breast
CNS
Colon/Rectum
CUP
Head and neck
Kidney
Liver
Mesothelioma
Other
Ovary
Pancreas
Prostate
Skin
Urinary tract
Uterus
Monoallelic: SNV MNV INDEL in frame SNV nonsense MNV nonsense INDEL out of frame
Biallelic/Multihit: SNV MNV INDEL in frame SNV nonsense MNV nonsense INDEL out of frame
Hotspot:

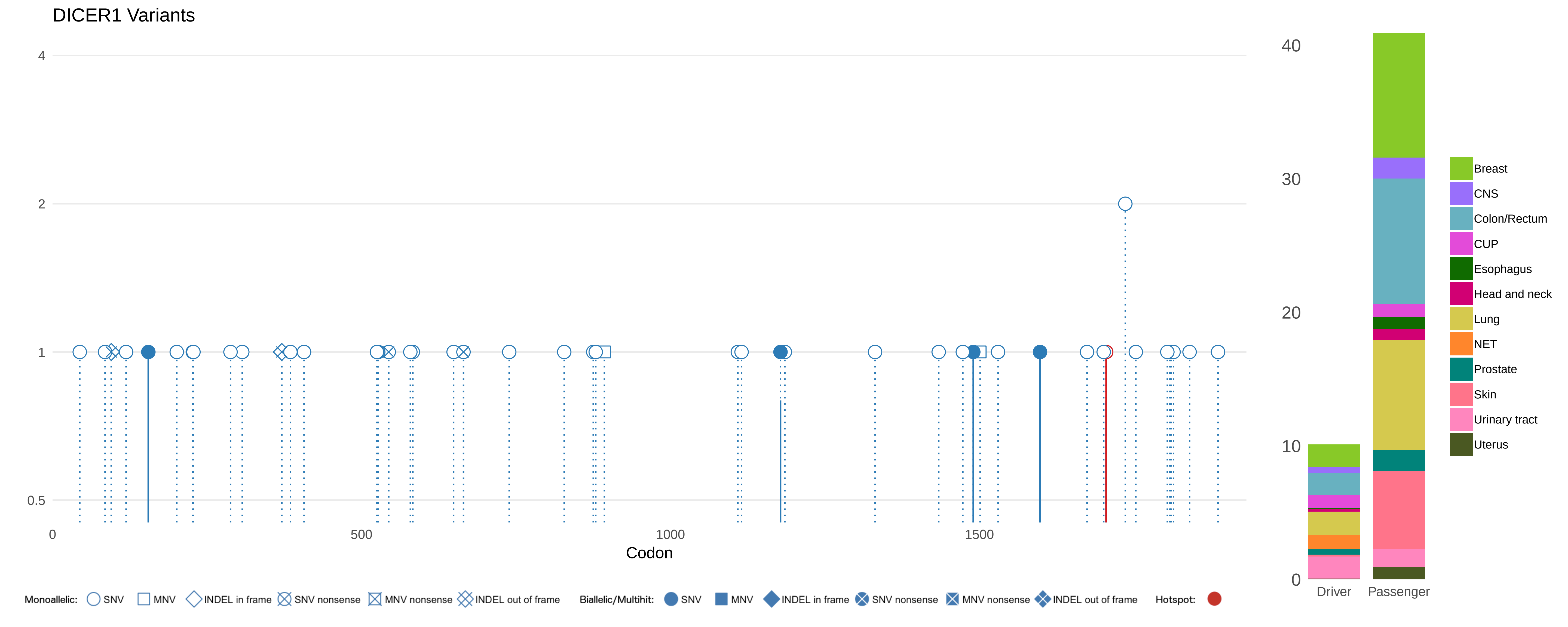

DICER1 Variants
4
2
1
0.5
0
500
1000
1500
Codon
Monoallelic: SNV MNV INDEL in frame SNV nonsense MNV nonsense INDEL out of frame
Biallelic/Multihit: SNV MNV INDEL in frame SNV nonsense MNV nonsense INDEL out of frame
Hotspot:
40
30
20
10
0
Driver
Passenger
Breast
CNS
Colon/Rectum
CUP
Esophagus
Head and neck
Lung
NET
Prostate
Skin
Urinary tract
Uterus

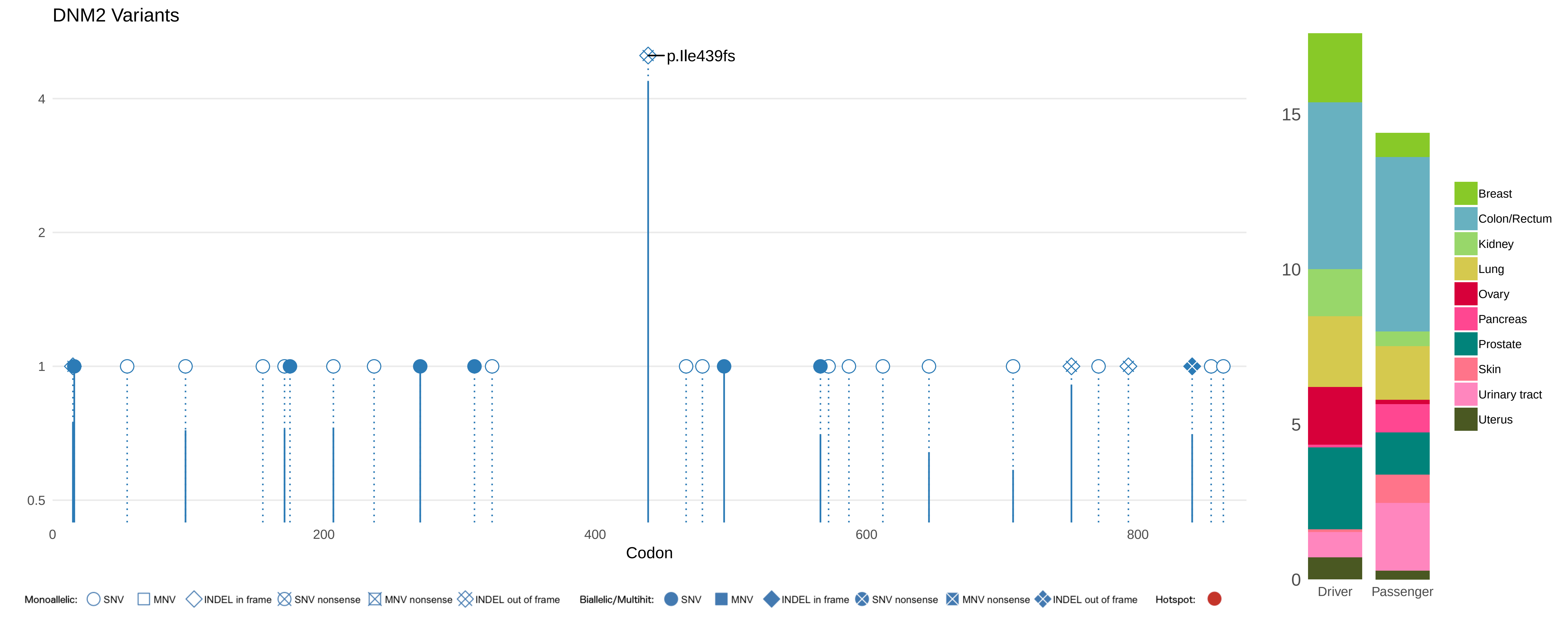
DNM2 Variants
p.Ile439fs
4
2
1
0.5
0
200
400
600
800
Codon
15
10
5
0
Driver
Passenger
Breast
Colon/Rectum
Kidney
Lung
Ovary
Pancreas
Prostate
Skin
Urinary tract
Uterus
Monoallelic: SNV MNV INDEL in frame SNV nonsense MNV nonsense INDEL out of frame
Biallelic/Multihit: SNV MNV INDEL in frame SNV nonsense MNV nonsense INDEL out of frame
Hotspot:

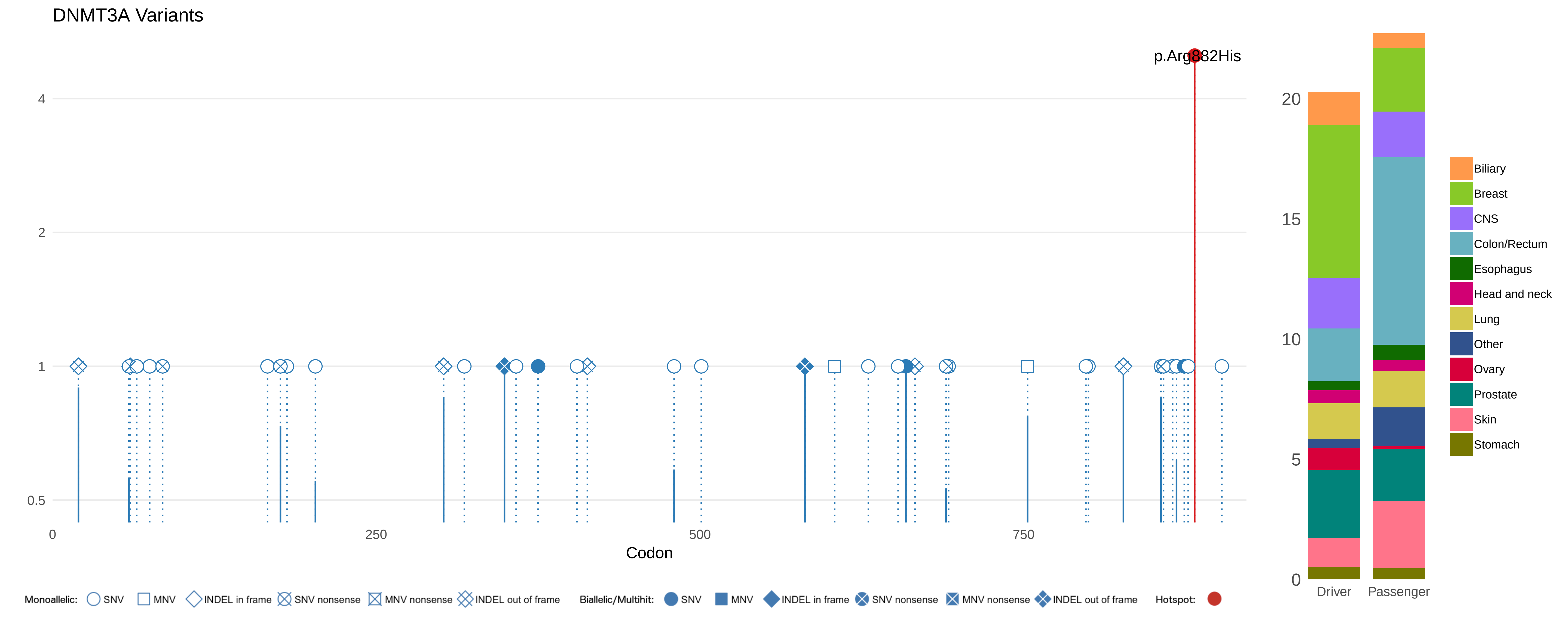

DNMT3A Variants
p.Arg882His
4
2
1
0.5
0
250
500
750
Codon
20
15
10
5
0
Driver
Passenger
Biliary
Breast
CNS
Colon/Rectum
Esophagus
Head and neck
Lung
Other
Ovary
Prostate
Skin
Stomach
Monoallelic: SNV MNV INDEL in frame SNV nonsense MNV nonsense INDEL out of frame
Biallelic/Multihit: SNV MNV INDEL in frame SNV nonsense MNV nonsense INDEL out of frame
Hotspot:

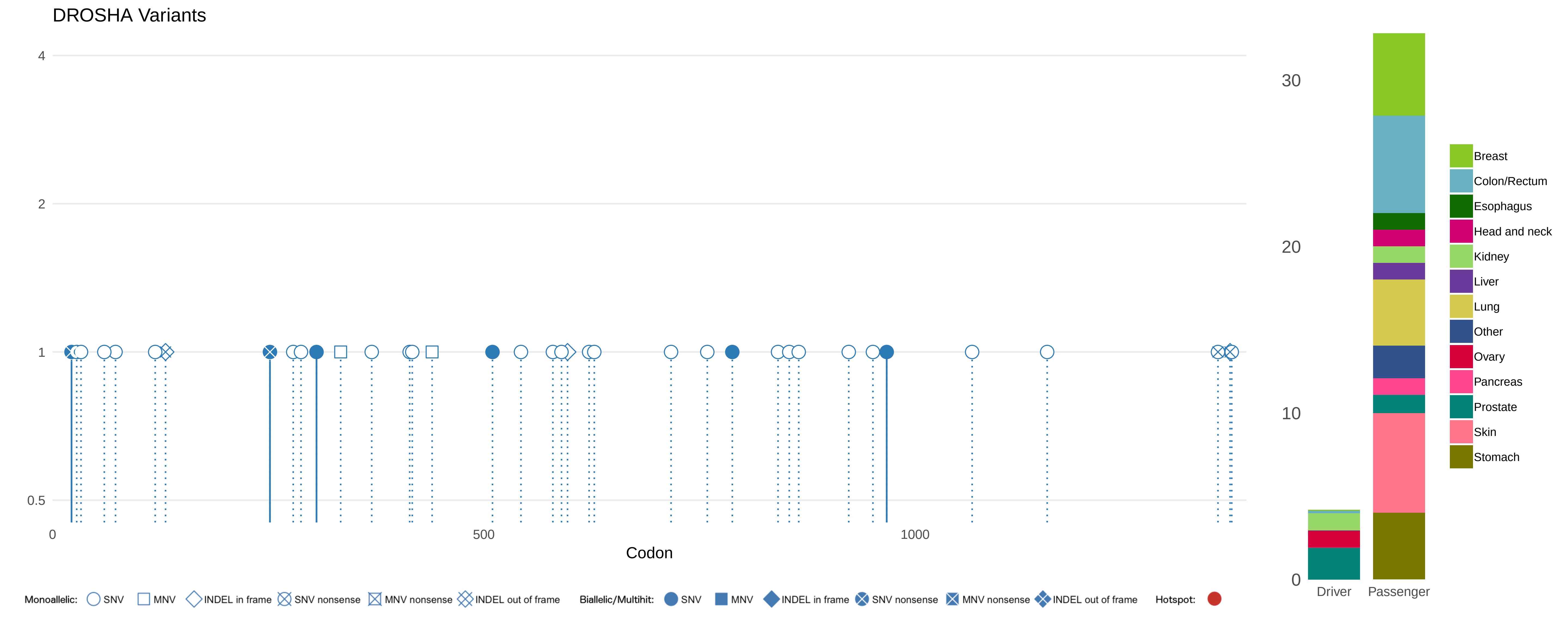

DROSHA Variants
4
2
1
0.5
0
500
1000
Codon
30
20
10
0
Driver
Passenger
Breast
Colon/Rectum
Esophagus
Head and neck
Kidney
Liver
Lung
Other
Ovary
Pancreas
Prostate
Skin
Stomach
Monoallelic: SNV MNV INDEL in frame SNV nonsense MNV nonsense INDEL out of frame
Biallelic/Multihit: SNV MNV INDEL in frame SNV nonsense MNV nonsense INDEL out of frame
Hotspot:

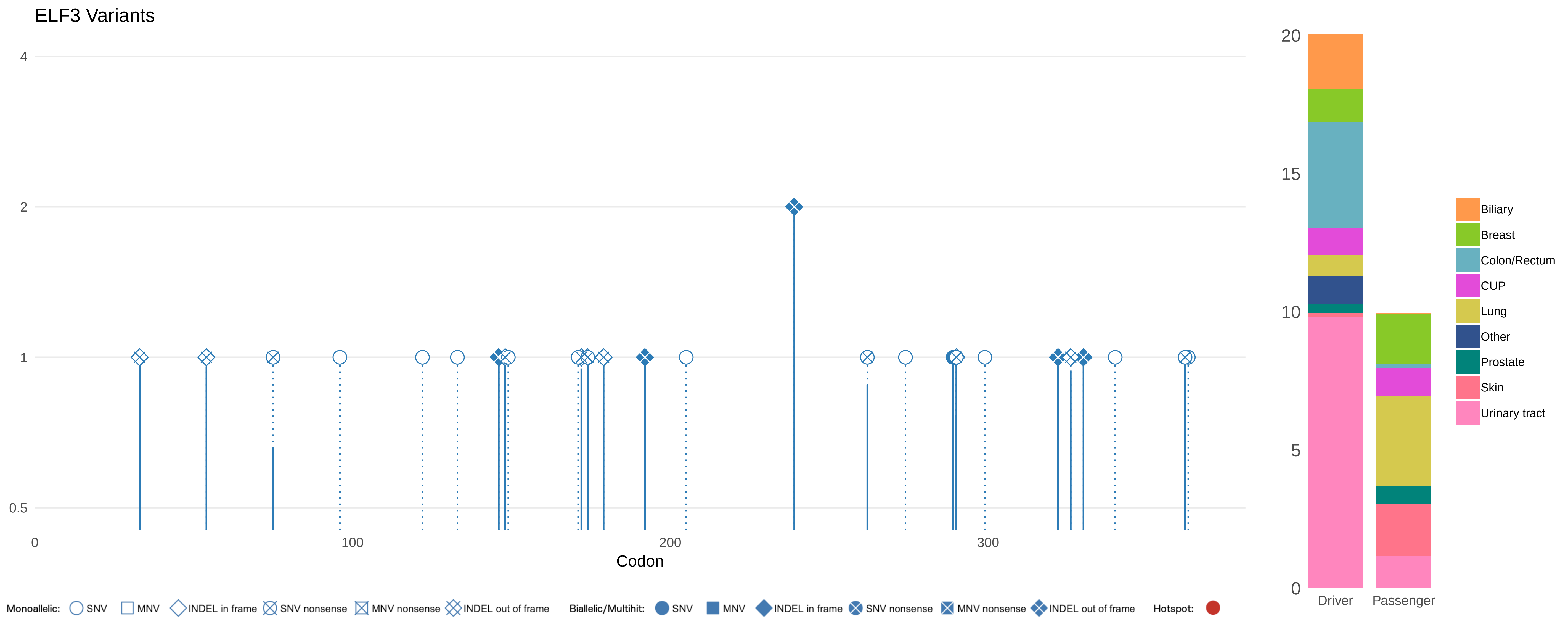
ELF3 Variants
4
2
1
0.5
0
100
200
300
Codon
Monoallelic: SNV MNV INDEL in frame SNV nonsense MNV nonsense INDEL out of frame
Biallelic/Multihit: SNV MNV INDEL in frame SNV nonsense MNV nonsense INDEL out of frame
Hotspot:
20
15
10
5
0
Driver
Passenger
Biliary
Breast
Colon/Rectum
CUP
Lung
Other
Prostate
Skin
Urinary tract

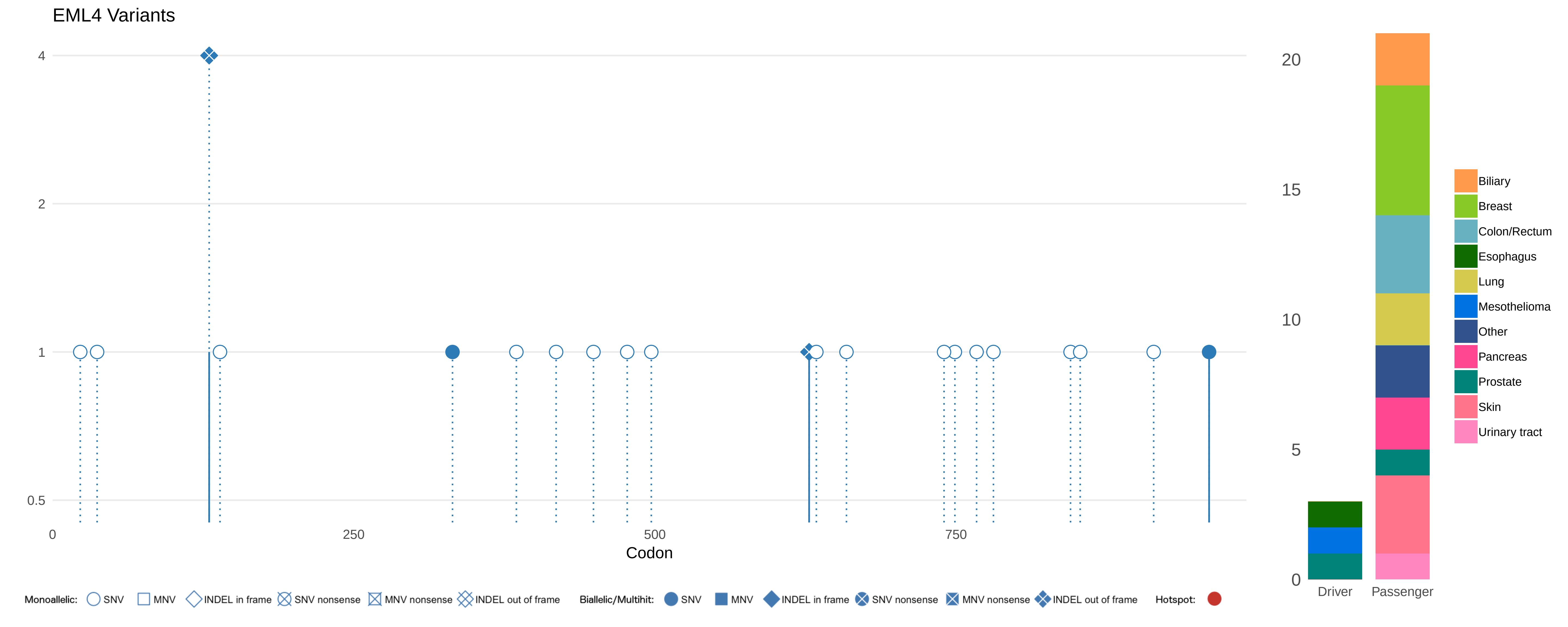
EML4 Variants
4
2
1
0.5
0
250
500
750
Codon
20
15
10
5
0
Driver
Passenger
Biliary
Breast
Colon/Rectum
Esophagus
Lung
Mesothelioma
Other
Pancreas
Prostate
Skin
Urinary tract
Monoallelic: SNV MNV INDEL in frame SNV nonsense MNV nonsense INDEL out of frame
Biallelic/Multihit: SNV MNV INDEL in frame SNV nonsense MNV nonsense INDEL out of frame
Hotspot:

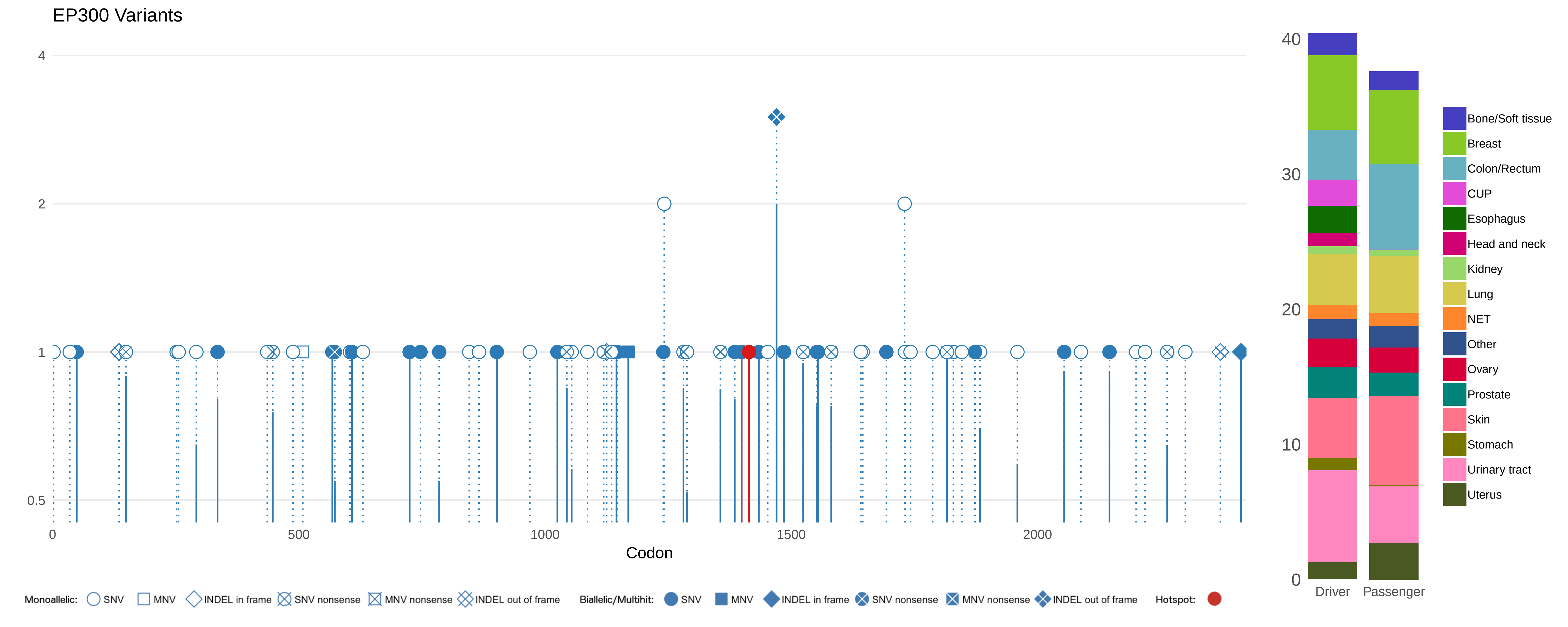
EP300 Variants
4
2
1
0.5
0
500
1000
1500
2000
Codon
Monoallelic: SNV MNV INDEL in frame SNV nonsense MNV nonsense INDEL out of frame
Biallelic/Multihit: SNV MNV INDEL in frame SNV nonsense MNV nonsense INDEL out of frame
Hotspot:
40
30
20
10
0
Driver
Passenger
Bone/Soft tissue
Breast
Colon/Rectum
CUP
Esophagus
Head and neck
Kidney
Lung
NET
Other
Ovary
Prostate
Skin
Stomach
Urinary tract
Uterus

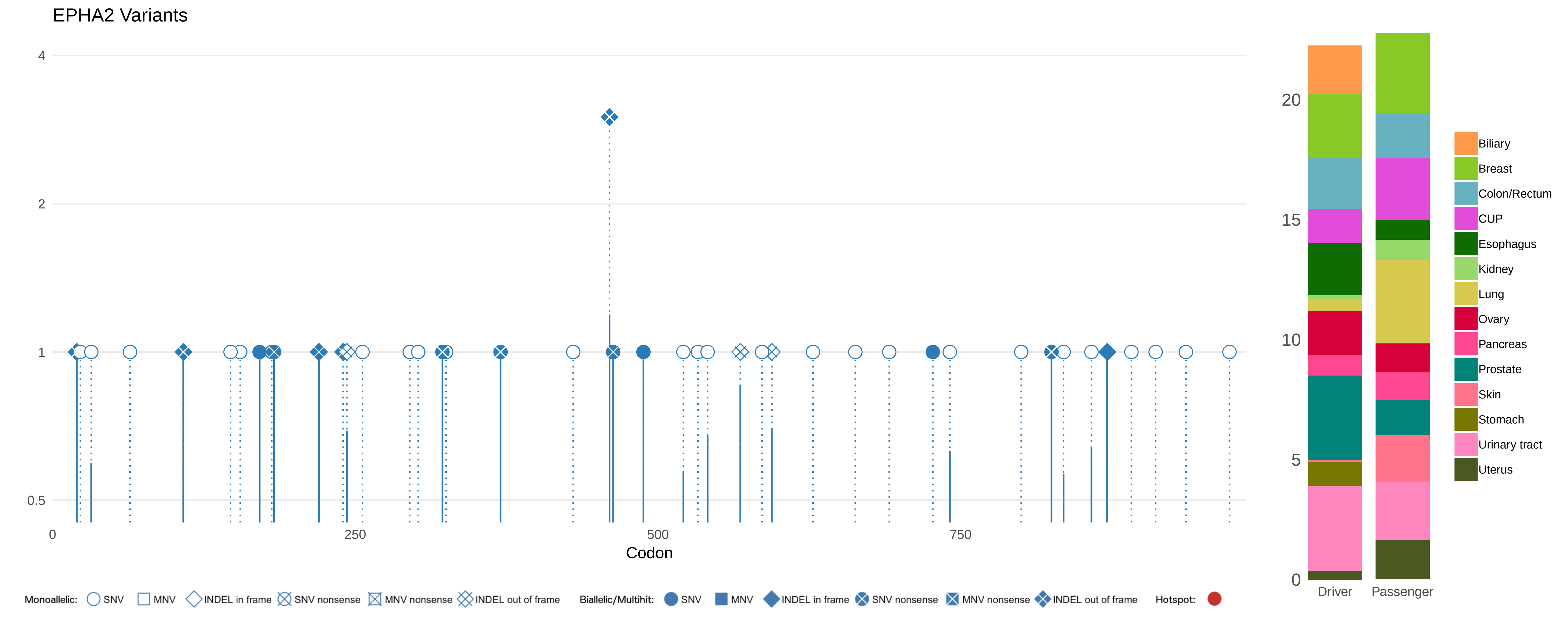
EPHA2 Variants
4
2
1
0.5
0
250
500
750
Codon
20
15
10
5
0
Driver
Passenger
Biliary
Breast
Colon/Rectum
CUP
Esophagus
Kidney
Lung
Ovary
Pancreas
Prostate
Skin
Stomach
Urinary tract
Uterus
Monoallelic: SNV MNV INDEL in frame SNV nonsense MNV nonsense INDEL out of frame
Biallelic/Multihit: SNV MNV INDEL in frame SNV nonsense MNV nonsense INDEL out of frame
Hotspot:

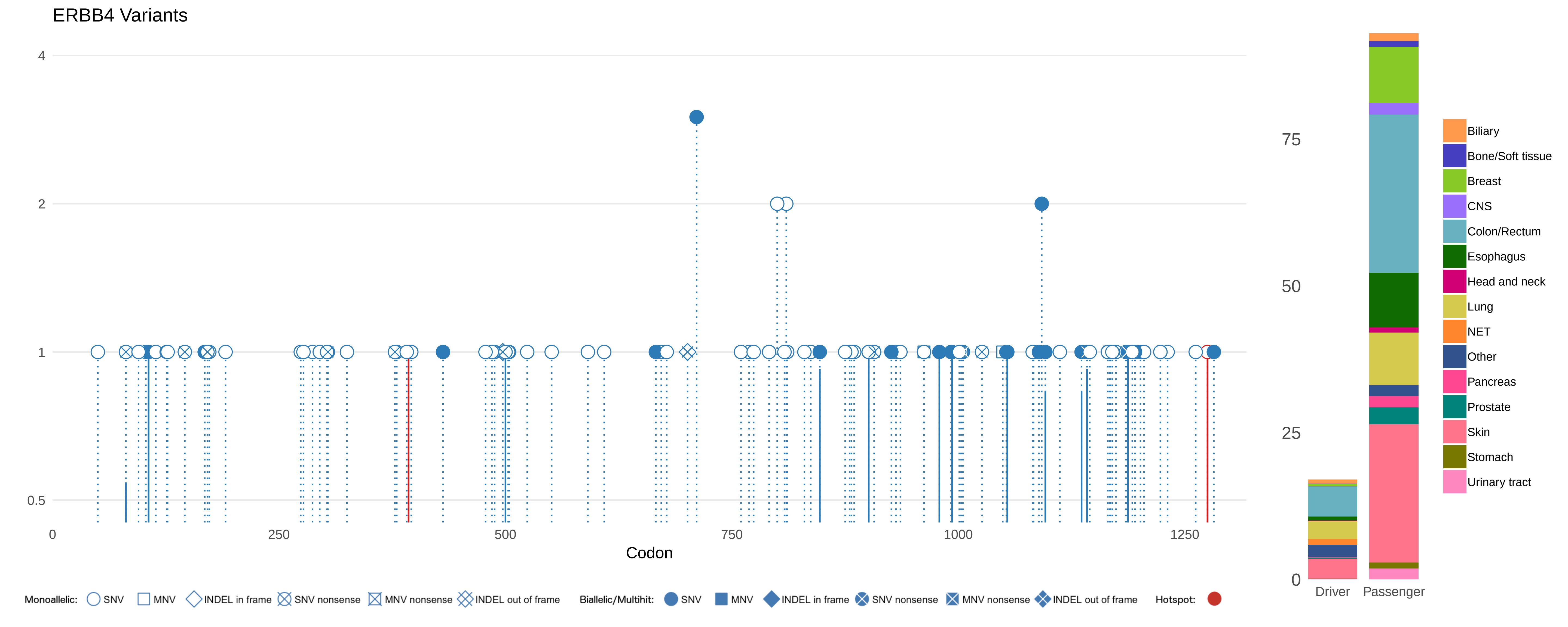
ERBB4 Variants
4
2
1
0.5
0
250
500
750
1000
1250
Codon
Monoallelic: SNV MNV INDEL in frame SNV nonsense MNV nonsense INDEL out of frame
Biallelic/Multihit: SNV MNV INDEL in frame SNV nonsense MNV nonsense INDEL out of frame
Hotspot:
75
50
25
0
Driver
Passenger
Biliary
Bone/Soft tissue
Breast
CNS
Colon/Rectum
Esophagus
Head and neck
Lung
NET
Other
Pancreas
Prostate
Skin
Stomach
Urinary tract

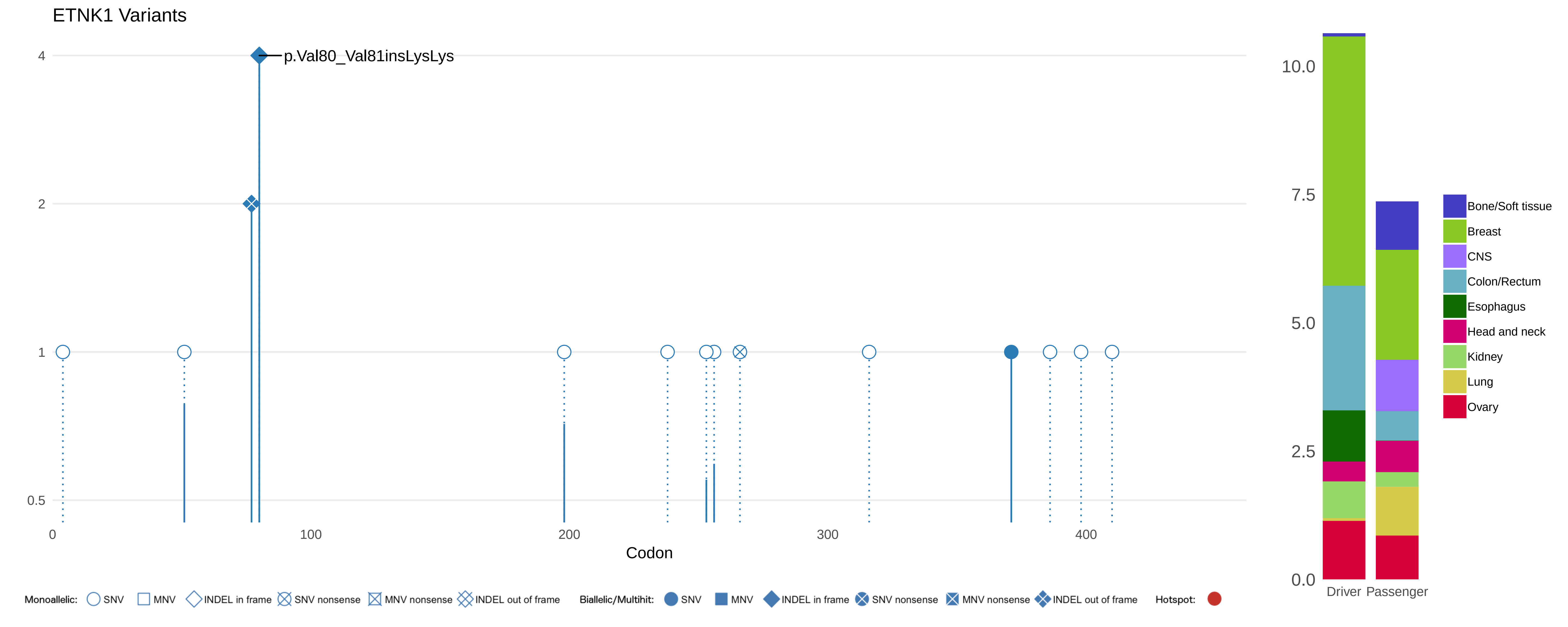

ETNK1 Variants
p.Val80_Val81insLysLys
4
2
1
0.5
0
100
200
300
400
Codon
10.0
7.5
5.0
2.5
0.0
Driver
Passenger
Bone/Soft tissue
Breast
CNS
Colon/Rectum
Esophagus
Head and neck
Kidney
Lung
Ovary
Monoallelic: SNV MNV INDEL in frame SNV nonsense MNV nonsense INDEL out of frame
Biallelic/Multihit: SNV MNV INDEL in frame SNV nonsense MNV nonsense INDEL out of frame
Hotspot:

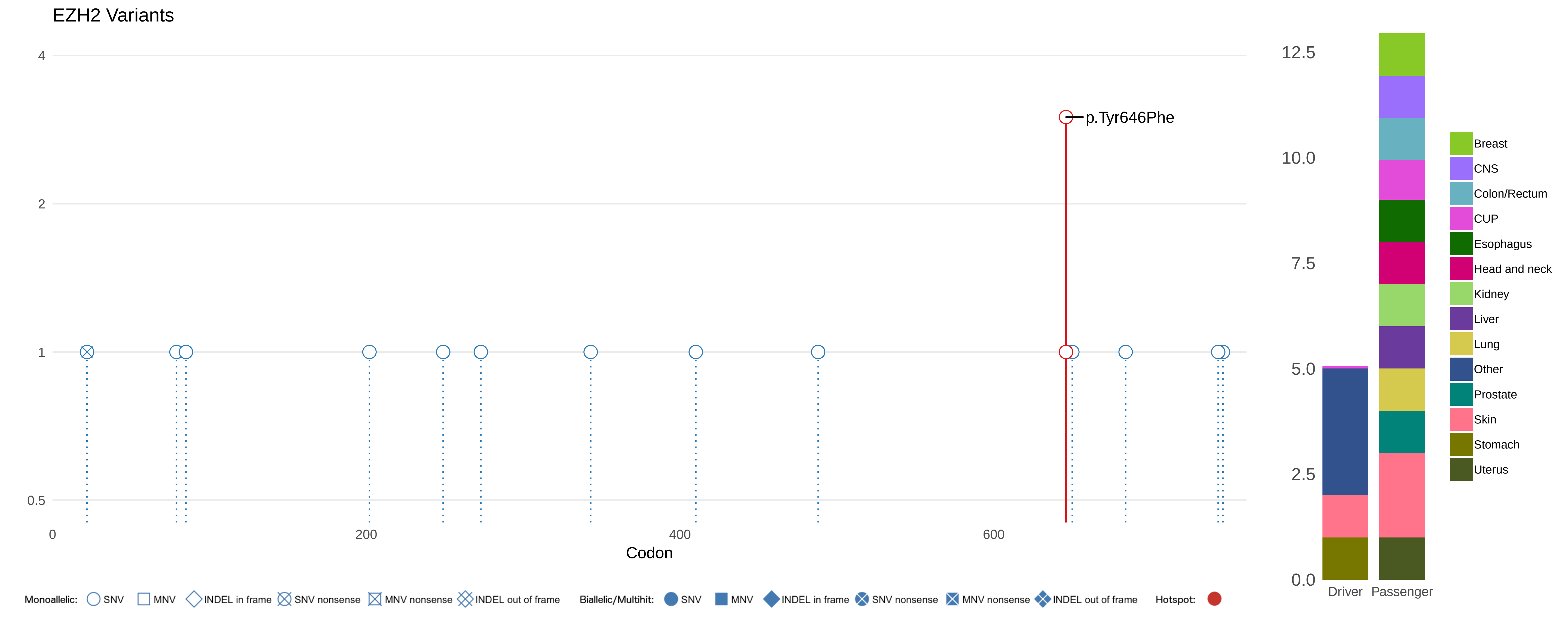
EZH2 Variants
4
2
1
0.5
0
200
400
600
Codon
p.Tyr646Phe
12.5
10.0
7.5
5.0
2.5
0.0
Driver
Passenger
Breast
CNS
Colon/Rectum
CUP
Esophagus
Head and neck
Kidney
Liver
Lung
Other
Prostate
Skin
Stomach
Uterus
Monoallelic: SNV MNV INDEL in frame SNV nonsense MNV nonsense INDEL out of frame
Biallelic/Multihit: SNV MNV INDEL in frame SNV nonsense MNV nonsense INDEL out of frame
Hotspot:

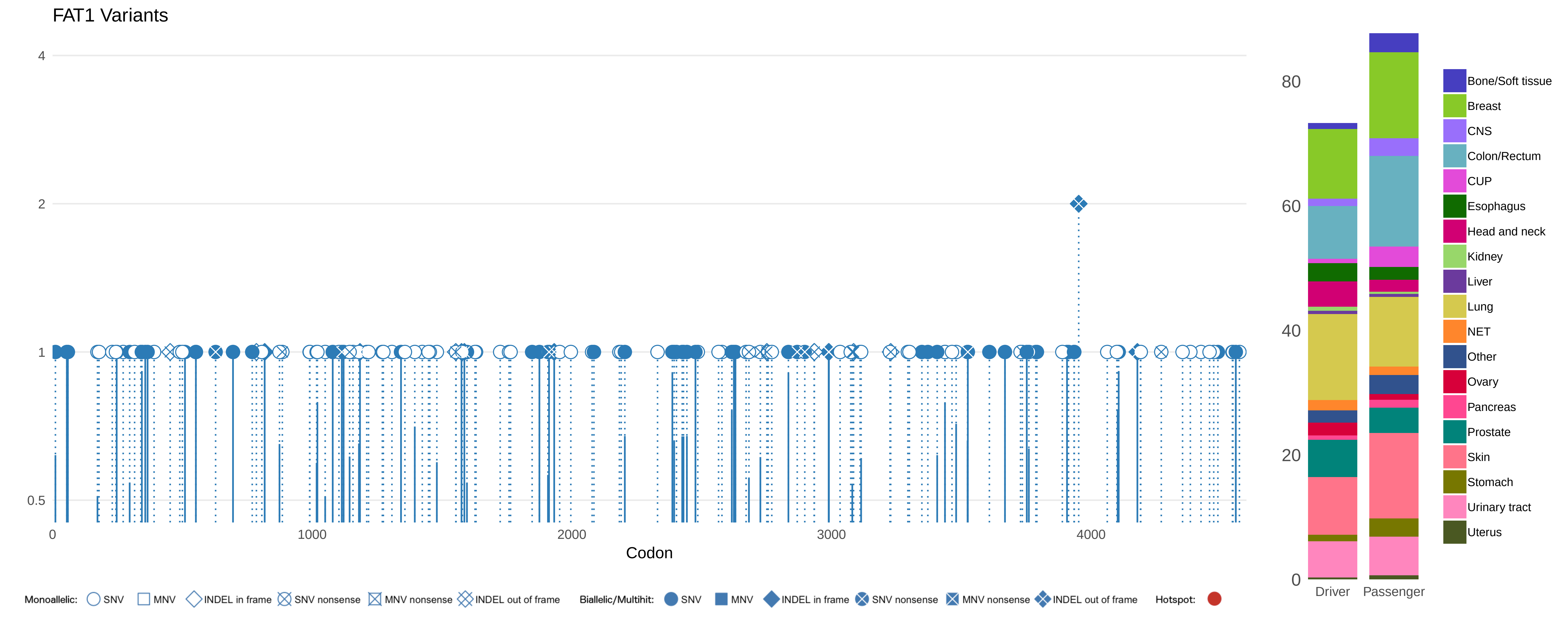
FAT1 Variants
4
2
1
0.5
0
1000
2000
3000
4000
Codon
Monoallelic: SNV MNV INDEL in frame SNV nonsense MNV nonsense INDEL out of frame
Biallelic/Multihit: SNV MNV INDEL in frame SNV nonsense MNV nonsense INDEL out of frame
Hotspot:
80
60
40
20
0
Driver
Passenger
Bone/Soft tissue
Breast
CNS
Colon/Rectum
CUP
Esophagus
Head and neck
Kidney
Liver
Lung
NET
Other
Ovary
Pancreas
Prostate
Skin
Stomach
Urinary tract
Uterus

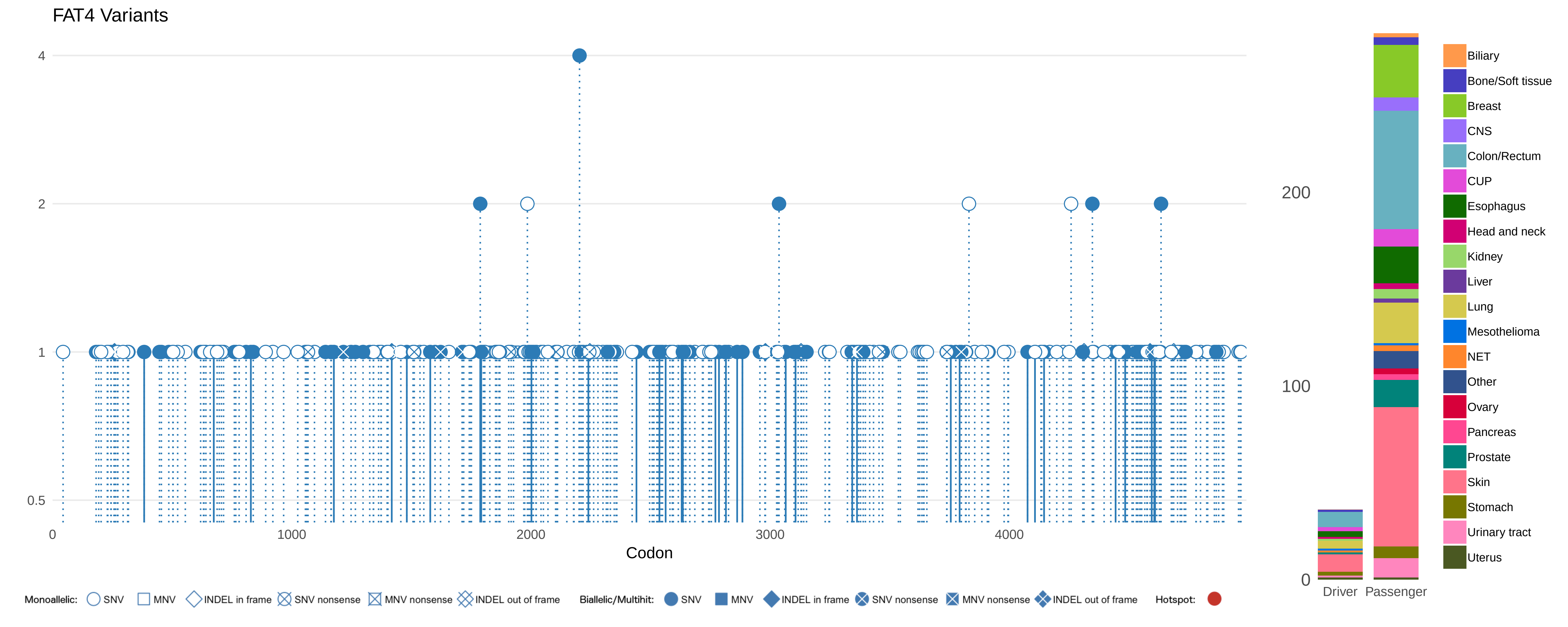
FAT4 Variants
4
2
1
0.5
0
1000
2000
3000
4000
Codon
200
100
0
Driver
Passenger
Biliary
Bone/Soft tissue
Breast
CNS
Colon/Rectum
CUP
Esophagus
Head and neck
Kidney
Liver
Lung
Mesothelioma
NET
Other
Ovary
Pancreas
Prostate
Skin
Stomach
Urinary tract
Uterus
Monoallelic: SNV MNV INDEL in frame SNV nonsense MNV nonsense INDEL out of frame
Biallelic/Multihit: SNV MNV INDEL in frame SNV nonsense MNV nonsense INDEL out of frame
Hotspot:

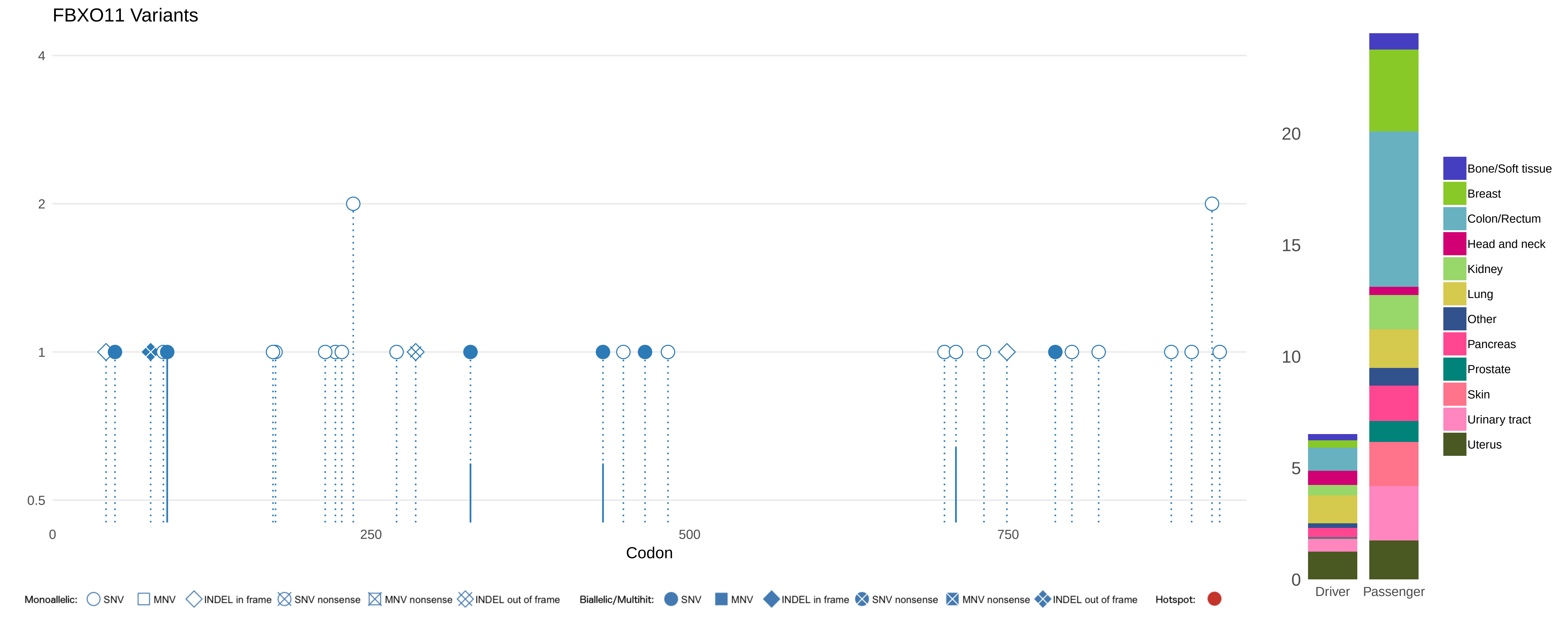

FBXO11 Variants
4
2
1
0.5
0
250
500
750
Codon
Monoallelic: SNV MNV INDEL in frame SNV nonsense MNV nonsense INDEL out of frame
Biallelic/Multihit: SNV MNV INDEL in frame SNV nonsense MNV nonsense INDEL out of frame
Hotspot:
20
15
10
5
0
Driver
Passenger
Bone/Soft tissue
Breast
Colon/Rectum
Head and neck
Kidney
Lung
Other
Pancreas
Prostate
Skin
Urinary tract
Uterus

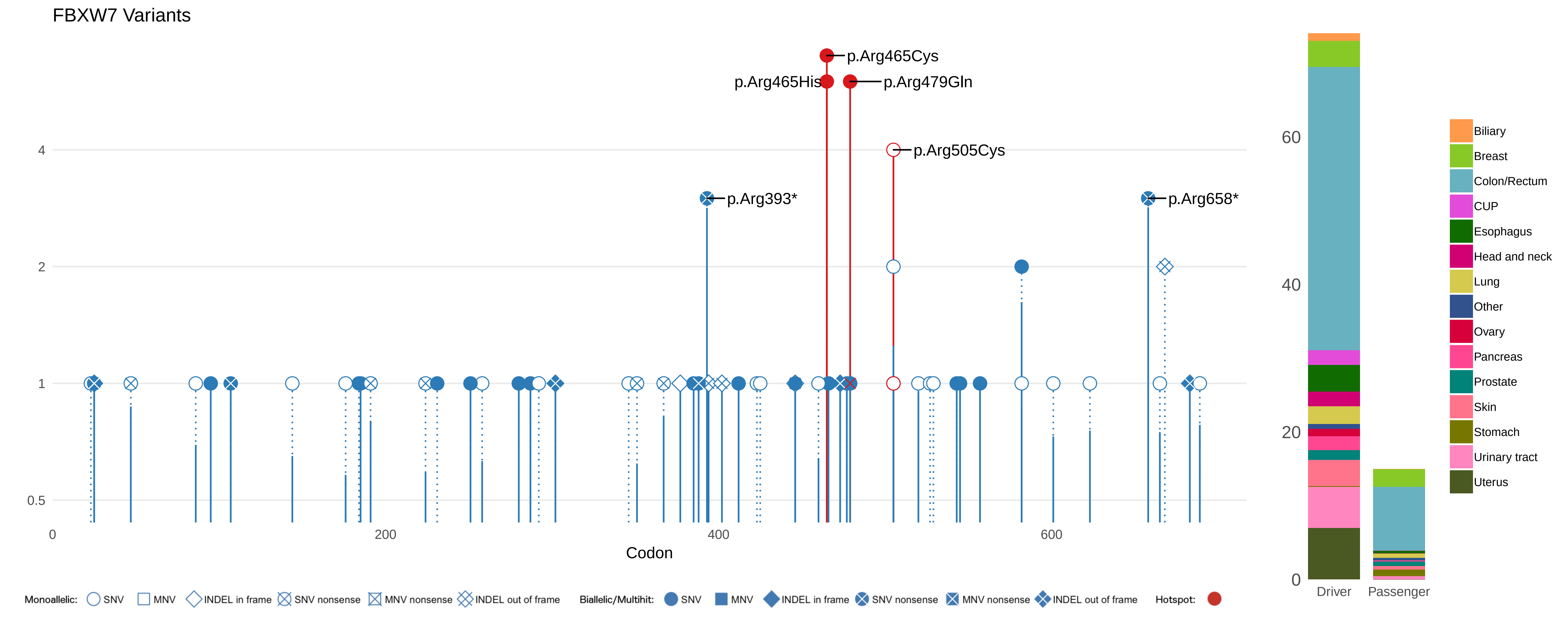
FBXW7 Variants
p.Arg465Cys
p.Arg465His
p.Arg479Gln
p.Arg505Cys
p.Arg393*
p.Arg658*
4
2
1
0.5
0
200
400
600
Codon
Monoallelic: SNV MNV INDEL in frame SNV nonsense MNV nonsense INDEL out of frame
Biallelic/Multihit: SNV MNV INDEL in frame SNV nonsense MNV nonsense INDEL out of frame
Hotspot:
60
40
20
0
Driver
Passenger
Biliary
Breast
Colon/Rectum
CUP
Esophagus
Head and neck
Lung
Other
Ovary
Pancreas
Prostate
Skin
Stomach
Urinary tract
Uterus

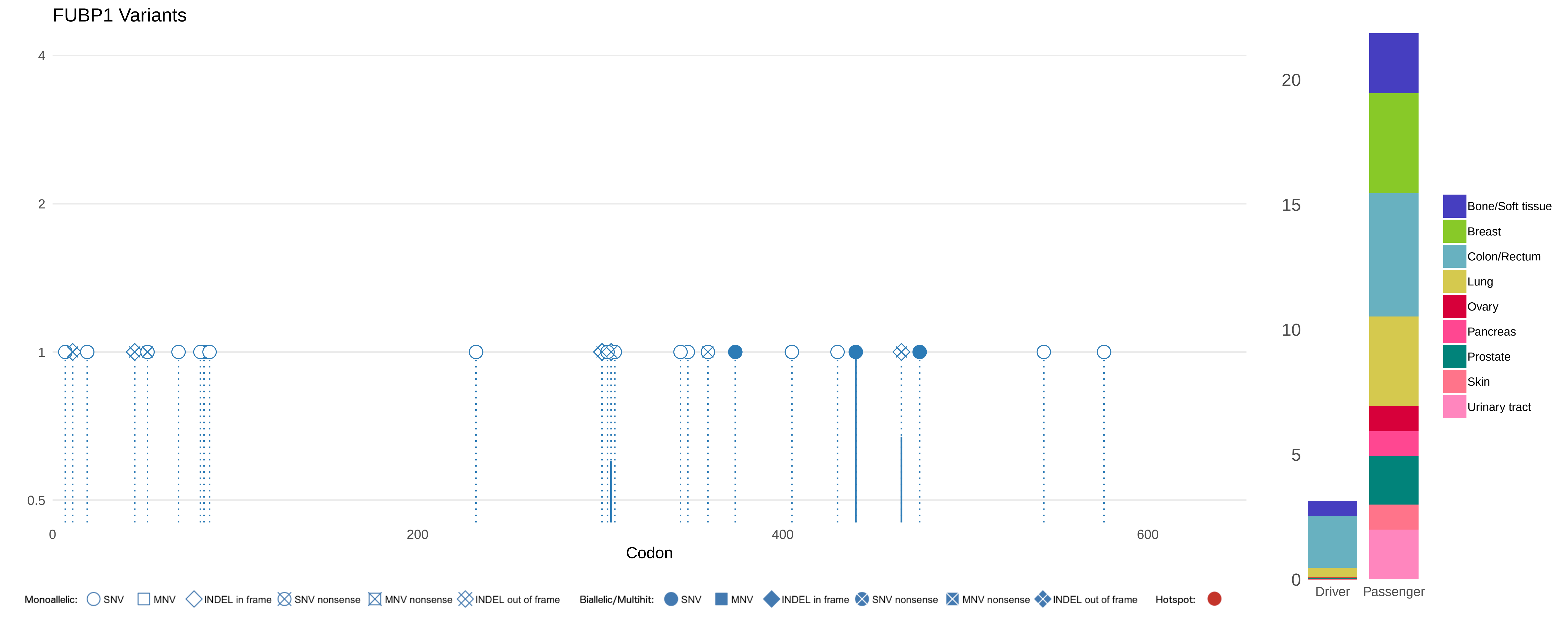

FUBP1 Variants
4
2
1
0.5
0
200
400
600
Codon
Monoallelic: SNV MNV INDEL in frame SNV nonsense MNV nonsense INDEL out of frame
Biallelic/Multihit: SNV MNV INDEL in frame SNV nonsense MNV nonsense INDEL out of frame
Hotspot:
20
15
10
5
0
Driver
Passenger
Bone/Soft tissue
Breast
Colon/Rectum
Lung
Ovary
Pancreas
Prostate
Skin
Urinary tract

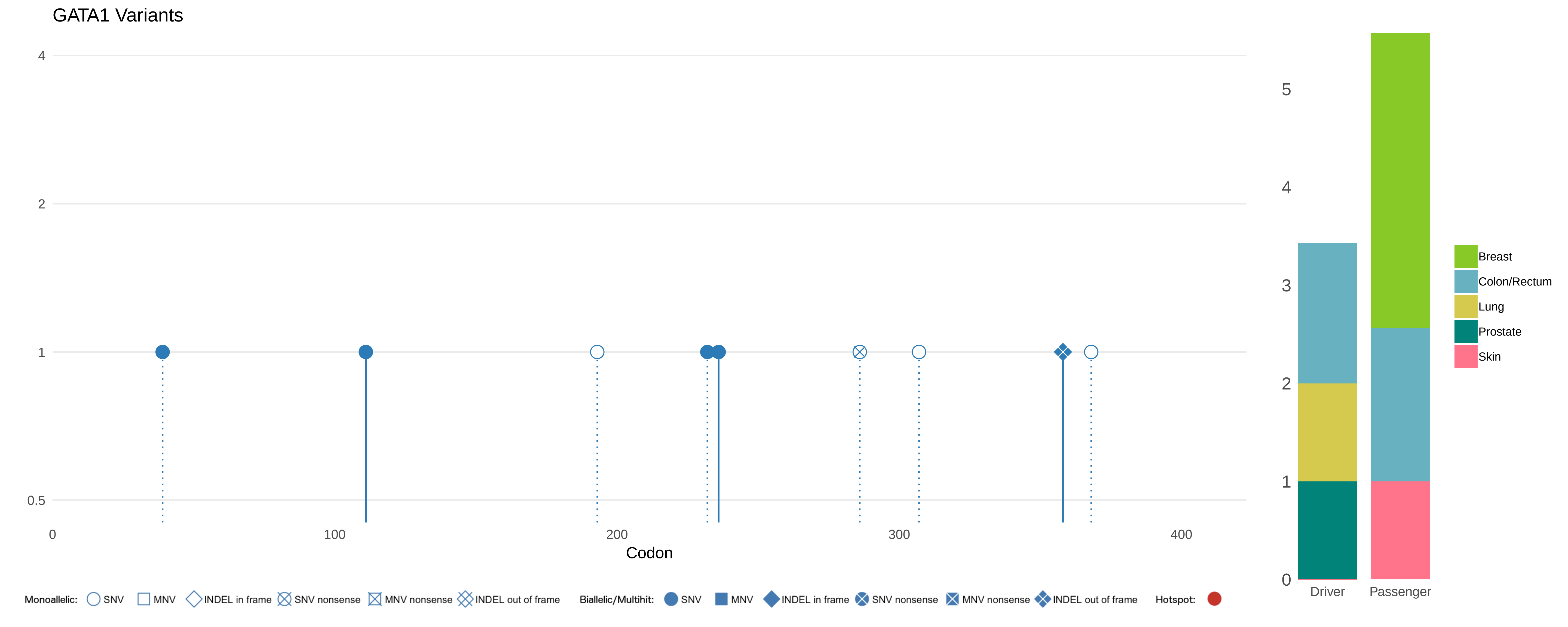
GATA1 Variants
4
2
1
0.5
0
100
200
300
400
Codon
5
4
3
2
1
0
Driver
Passenger
Breast
Colon/Rectum
Lung
Prostate
Skin
Monoallelic: SNV MNV INDEL in frame SNV nonsense MNV nonsense INDEL out of frame
Biallelic/Multihit: SNV MNV INDEL in frame SNV nonsense MNV nonsense INDEL out of frame
Hotspot:

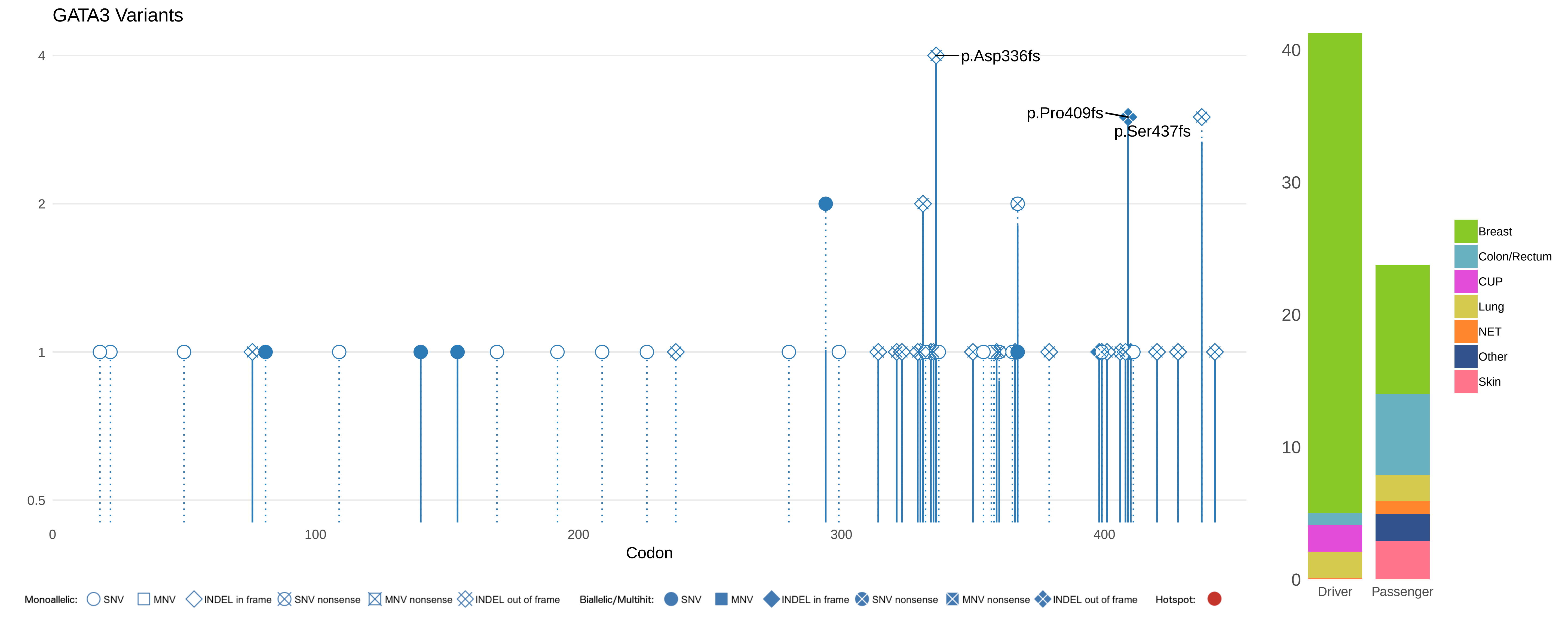

GATA3 Variants
p.Asp336fs
p.Pro409fs
p.Ser437fs
Codon
Monoallelic: SNV MNV INDEL in frame SNV nonsense MNV nonsense INDEL out of frame
Biallelic/Multihit: SNV MNV INDEL in frame SNV nonsense MNV nonsense INDEL out of frame
Hotspot:
Driver
Passenger
Breast
Colon/Rectum
CUP
Lung
NET
Other
Skin

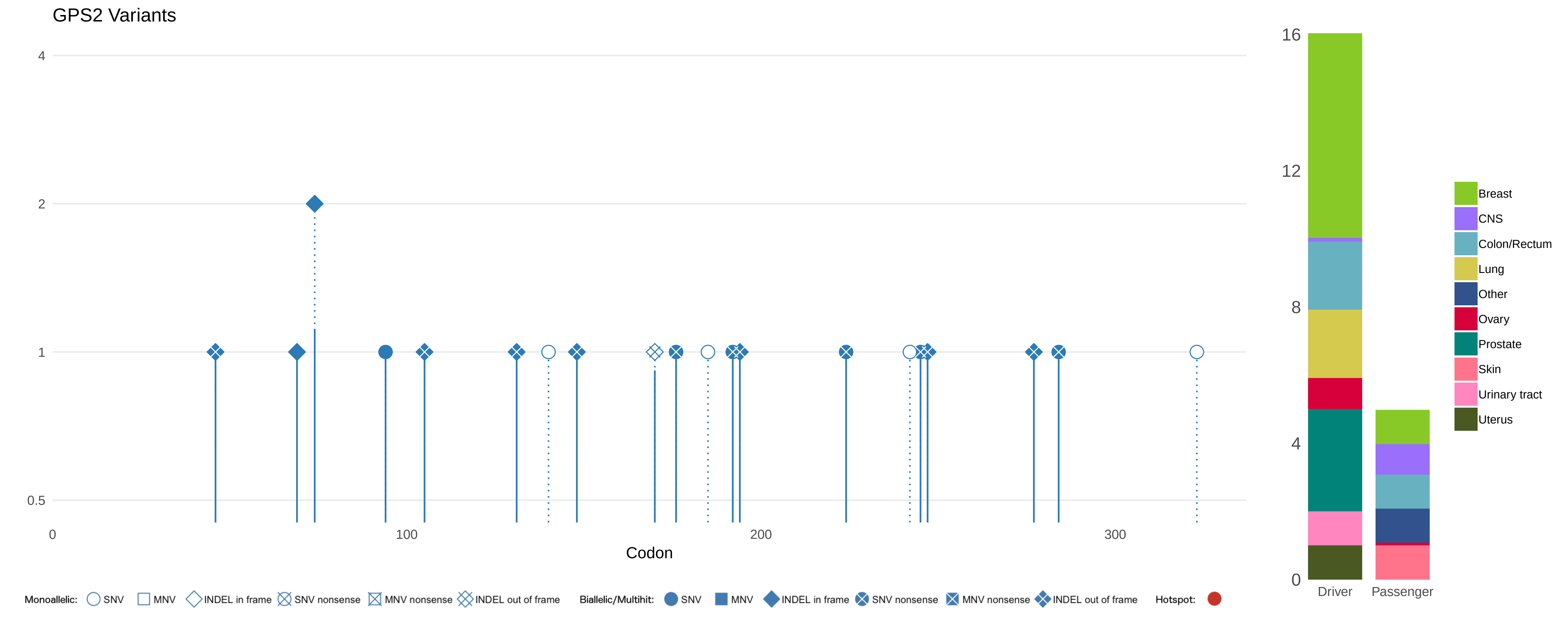
GPS2 Variants
4
2
1
0.5
0
100
200
300
Codon
Monoallelic: SNV MNV INDEL in frame SNV nonsense MNV nonsense INDEL out of frame
Biallelic/Multihit: SNV MNV INDEL in frame SNV nonsense MNV nonsense INDEL out of frame
Hotspot:
16
12
8
4
0
Driver
Passenger
Breast
CNS
Colon/Rectum
Lung
Other
Ovary
Prostate
Skin
Urinary tract
Uterus

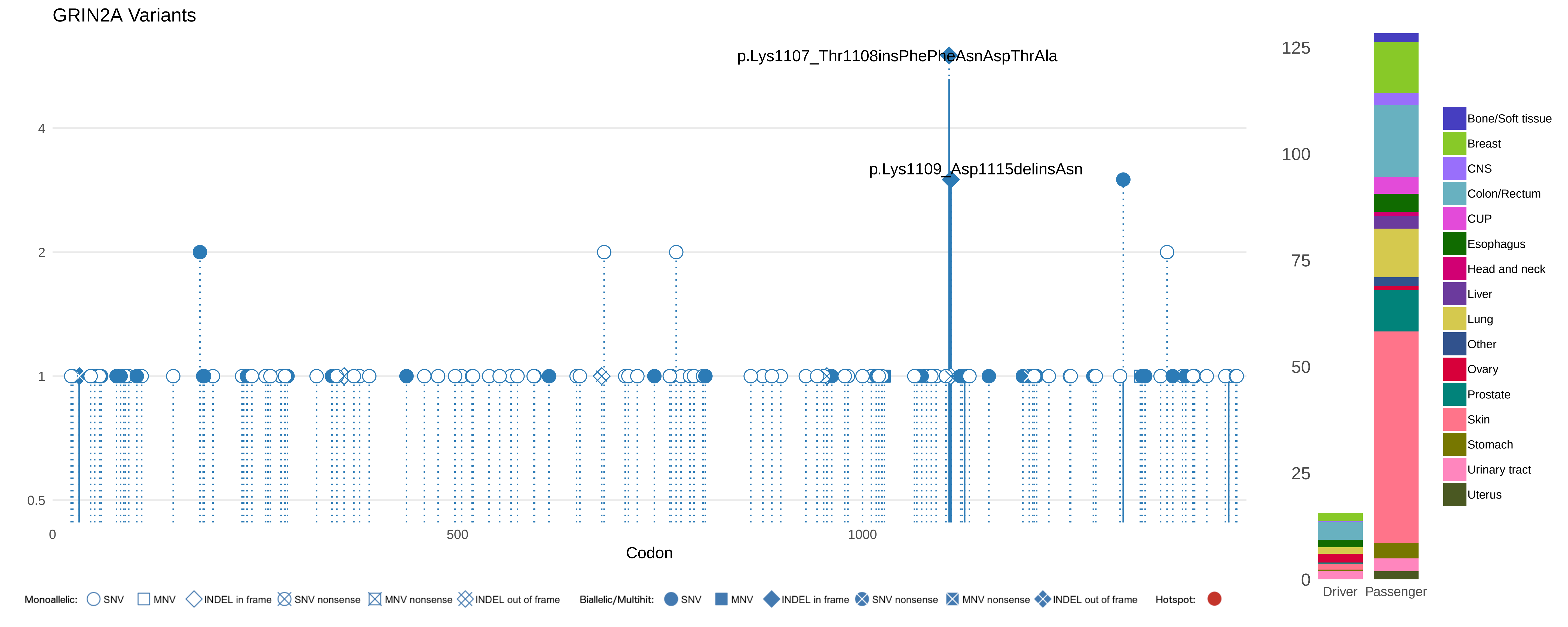

GRIN2A Variants
p.Lys1107_Thr1108insPhePheAsnAspThrAla
p.Lys1109_Asp1115delinsAsn
4
2
1
0.5
0
500
1000
Codon
125
100
75
50
25
0
Driver
Passenger
Bone/Soft tissue
Breast
CNS
Colon/Rectum
CUP
Esophagus
Head and neck
Liver
Lung
Other
Ovary
Prostate
Skin
Stomach
Urinary tract
Uterus
Monoallelic: SNV MNV INDEL in frame SNV nonsense MNV nonsense INDEL out of frame
Biallelic/Multihit: SNV MNV INDEL in frame SNV nonsense MNV nonsense INDEL out of frame
Hotspot:

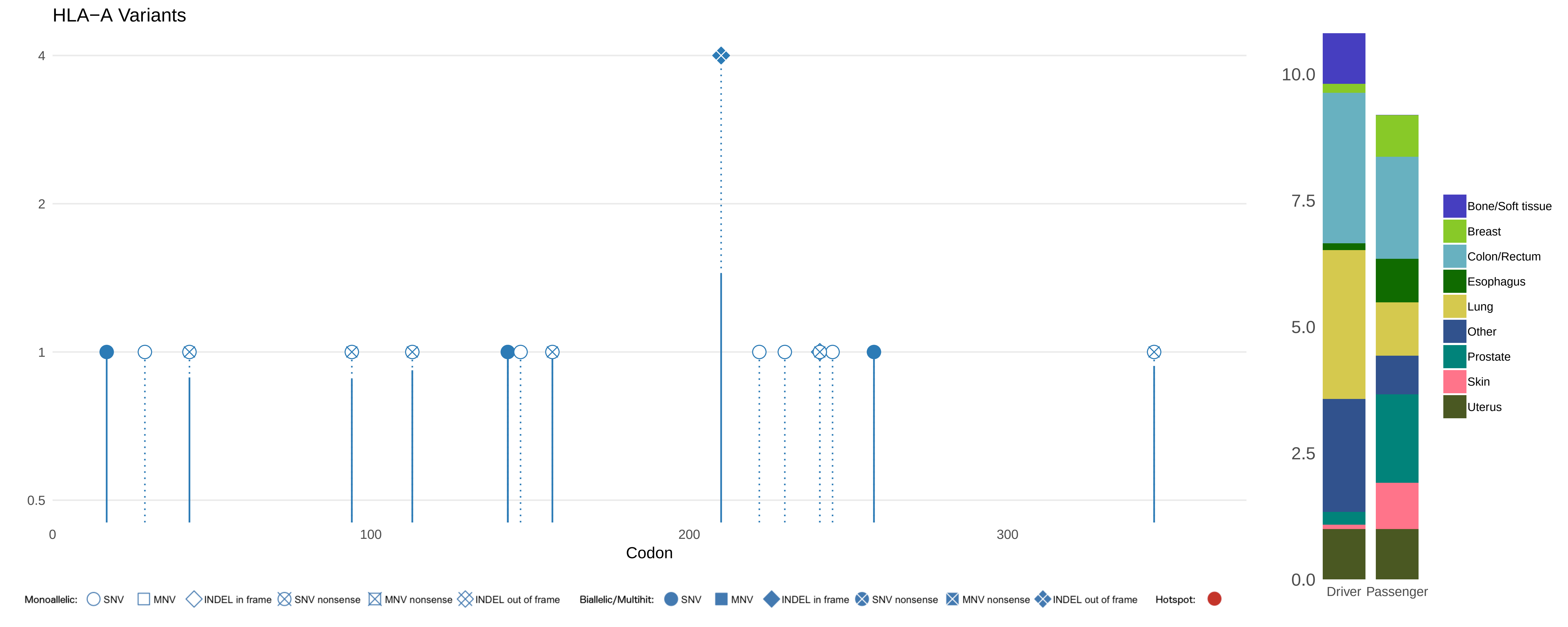
HLA−A Variants
4
2
1
0.5
0
100
200
300
Codon
10.0
7.5
5.0
2.5
0.0
Driver
Passenger
Bone/Soft tissue
Breast
Colon/Rectum
Esophagus
Lung
Other
Prostate
Skin
Uterus
Monoallelic: SNV MNV INDEL in frame SNV nonsense MNV nonsense INDEL out of frame
Biallelic/Multihit: SNV MNV INDEL in frame SNV nonsense MNV nonsense INDEL out of frame
Hotspot:

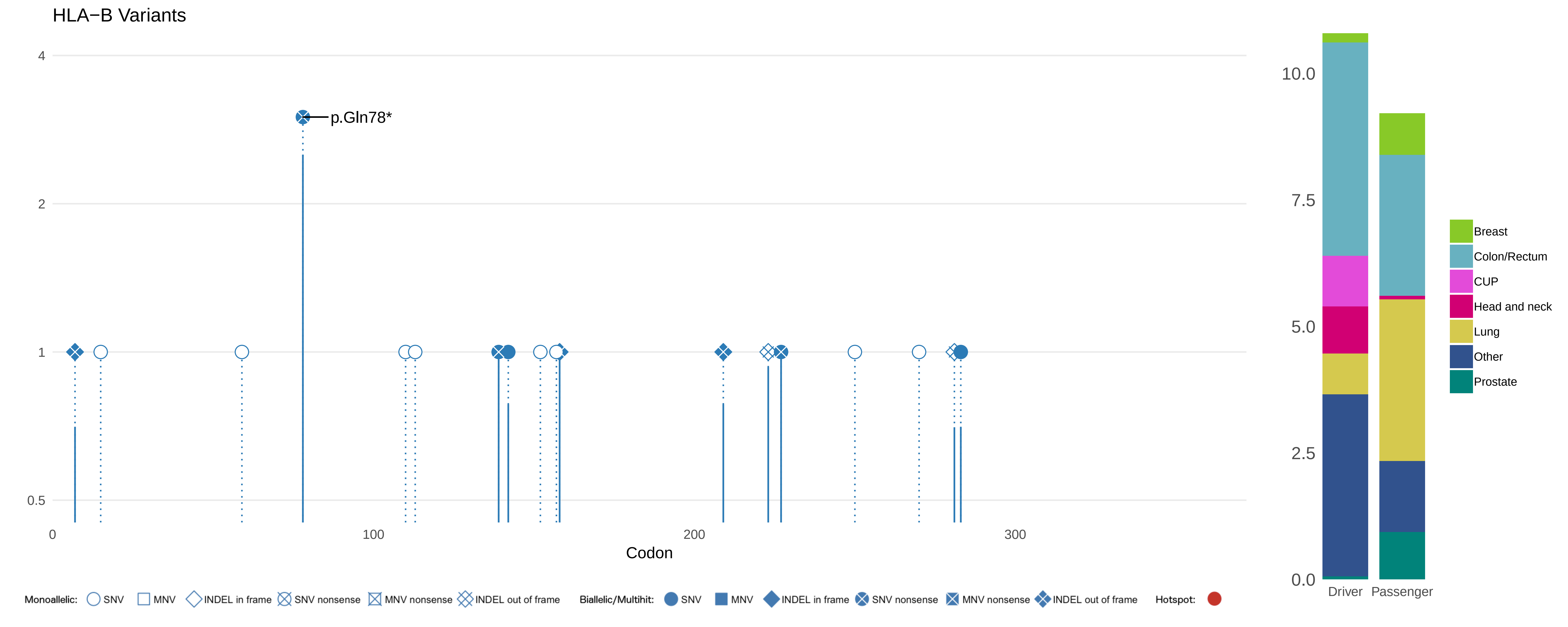
HLA−B Variants
4
2
1
0.5
p.Gln78*
0
100
200
300
Codon
10.0
7.5
5.0
2.5
0.0
Driver
Passenger
Breast
Colon/Rectum
CUP
Head and neck
Lung
Other
Prostate
Monoallelic: SNV MNV INDEL in frame SNV nonsense MNV nonsense INDEL out of frame
Biallelic/Multihit: SNV MNV INDEL in frame SNV nonsense MNV nonsense INDEL out of frame
Hotspot:

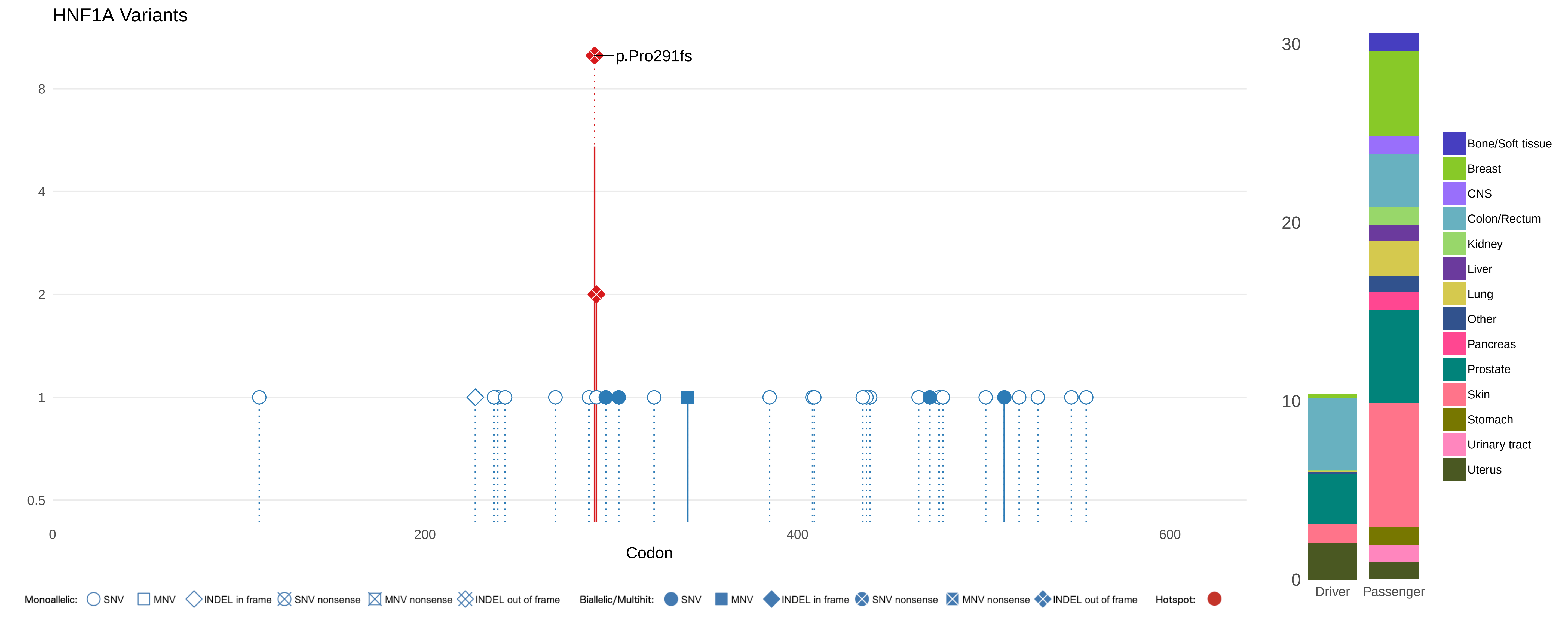
HNF1A Variants
p.Pro291fs
8
4
2
1
0.5
0
200
400
600
Codon
Monoallelic: SNV MNV INDEL in frame SNV nonsense MNV nonsense INDEL out of frame
Biallelic/Multihit: SNV MNV INDEL in frame SNV nonsense MNV nonsense INDEL out of frame
Hotspot:
30
20
10
0
Driver
Passenger
Bone/Soft tissue
Breast
CNS
Colon/Rectum
Kidney
Liver
Lung
Other
Pancreas
Prostate
Skin
Stomach
Urinary tract
Uterus

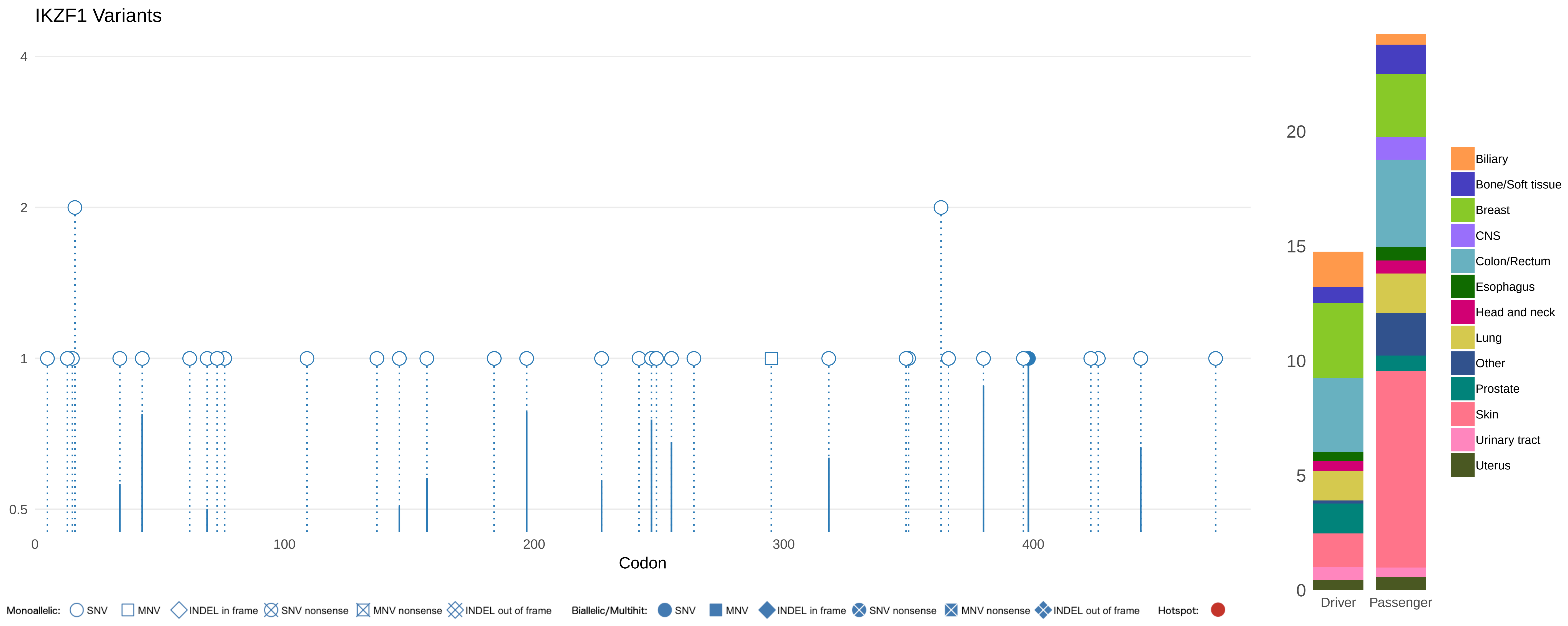
IKZF1 Variants
4
2
1
0.5
0
100
200
300
400
Codon
Monoallelic: SNV MNV INDEL in frame SNV nonsense MNV nonsense INDEL out of frame
Biallelic/Multihit: SNV MNV INDEL in frame SNV nonsense MNV nonsense INDEL out of frame
Hotspot:
20
15
10
5
0
Driver
Passenger
Biliary
Bone/Soft tissue
Breast
CNS
Colon/Rectum
Esophagus
Head and neck
Lung
Other
Prostate
Skin
Urinary tract
Uterus

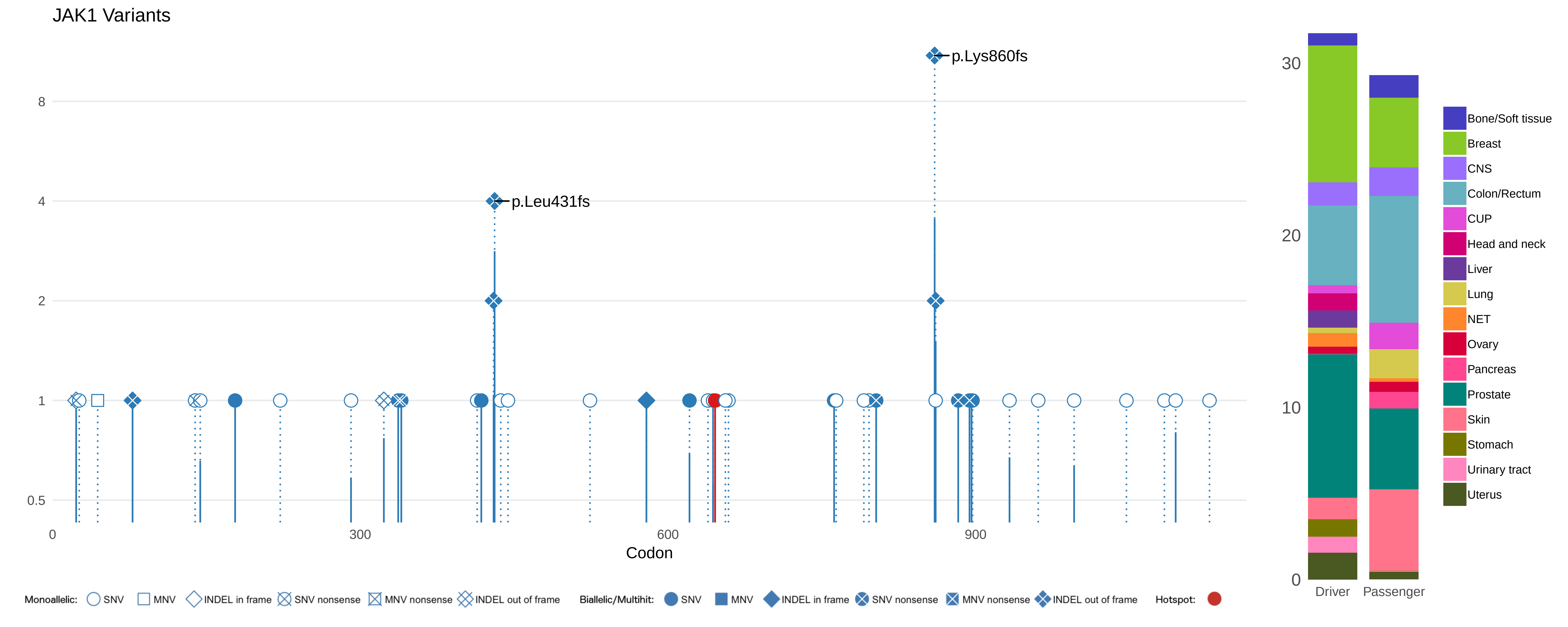

JAK1 Variants
p.Lys860fs
p.Leu431fs
8
4
2
1
0.5
0
300
600
900
Codon
30
20
10
0
Driver
Passenger
Bone/Soft tissue
Breast
CNS
Colon/Rectum
CUP
Head and neck
Liver
Lung
NET
Ovary
Pancreas
Prostate
Skin
Stomach
Urinary tract
Uterus
Monoallelic: SNV MNV INDEL in frame SNV nonsense MNV nonsense INDEL out of frame
Biallelic/Multihit: SNV MNV INDEL in frame SNV nonsense MNV nonsense INDEL out of frame
Hotspot:

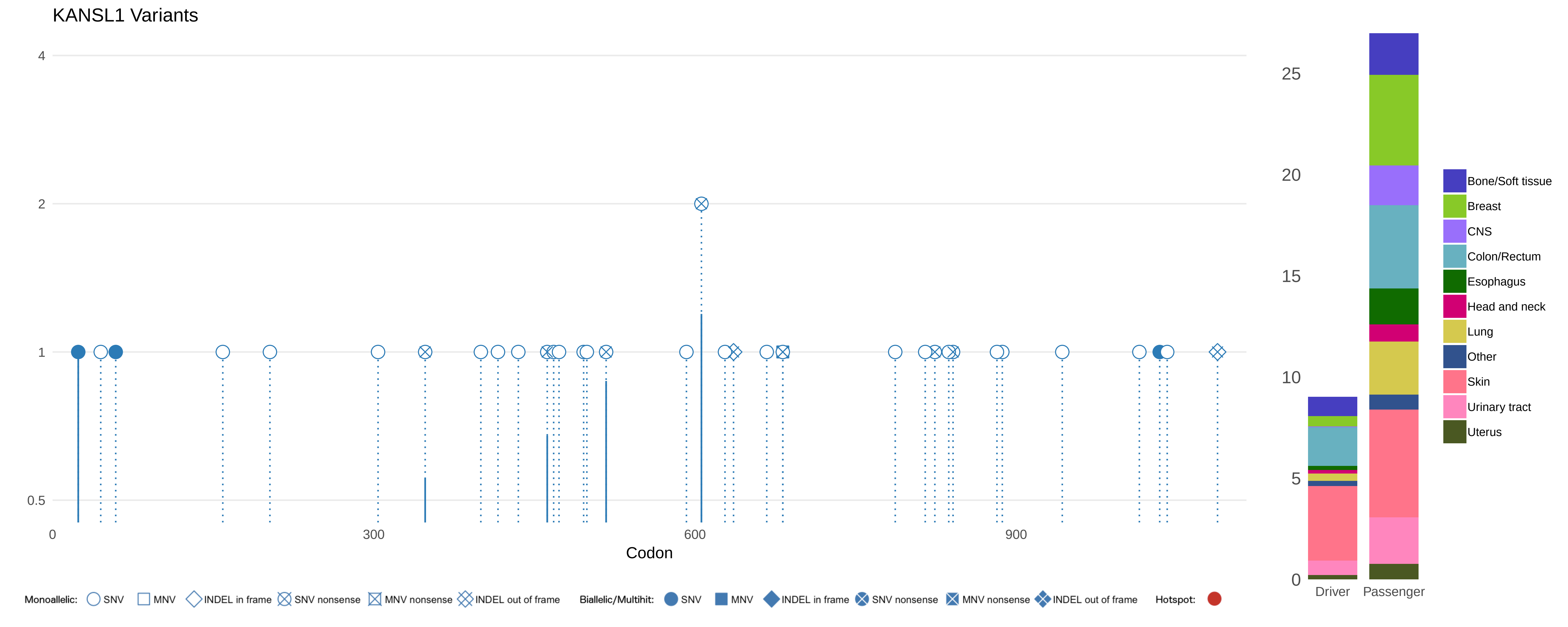
KANSL1 Variants
4
2
1
0.5
0
300
600
900
Codon
25
20
15
10
5
0
Driver
Passenger
Bone/Soft tissue
Breast
CNS
Colon/Rectum
Esophagus
Head and neck
Lung
Other
Skin
Urinary tract
Uterus
Monoallelic:
SNV
MNV
INDEL in frame
SNV nonsense
MNV nonsense
INDEL out of frame
Biallelic/Multihit:
SNV
MNV
INDEL in frame
SNV nonsense
MNV nonsense
INDEL out of frame
Hotspot:

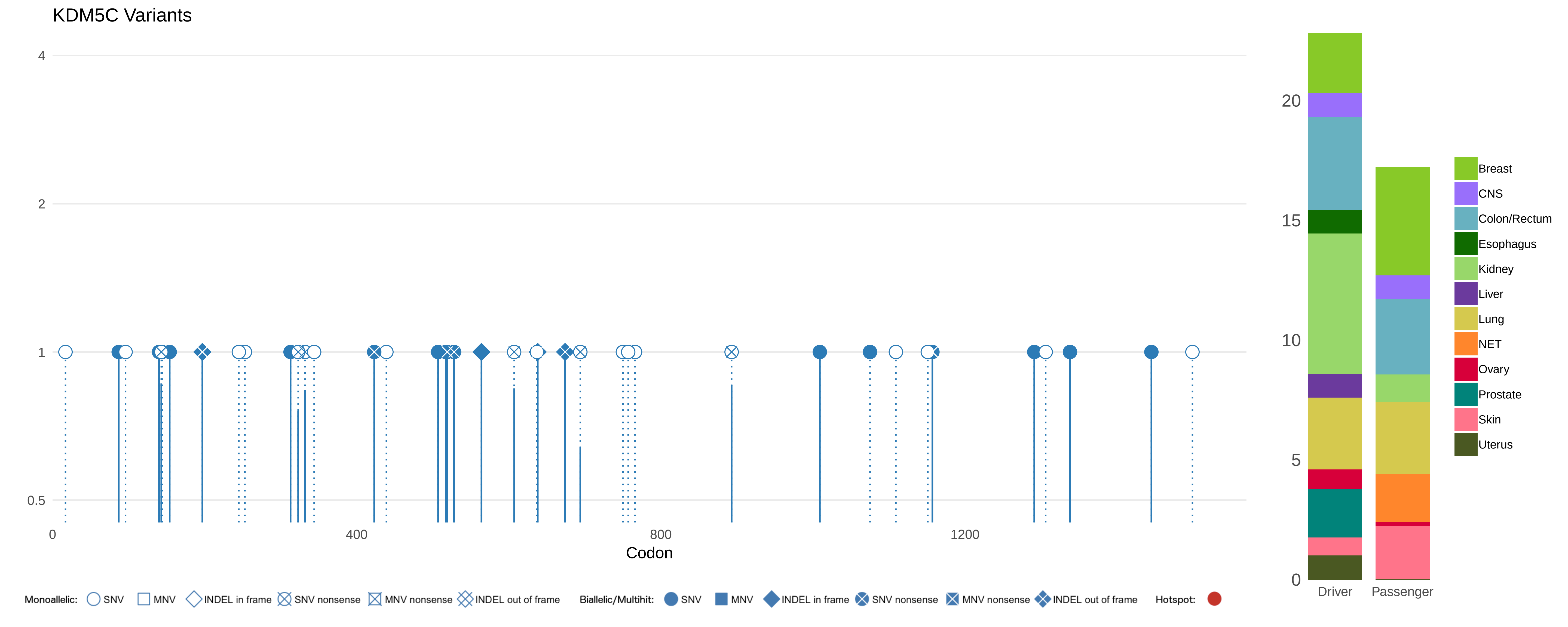

KDM5C Variants
4
2
1
0.5
0
400
800
1200
Codon
20
15
10
5
0
Driver
Passenger
Breast
CNS
Colon/Rectum
Esophagus
Kidney
Liver
Lung
NET
Ovary
Prostate
Skin
Uterus
Monoallelic: SNV MNV INDEL in frame SNV nonsense MNV nonsense INDEL out of frame
Biallelic/Multihit: SNV MNV INDEL in frame SNV nonsense MNV nonsense INDEL out of frame
Hotspot:

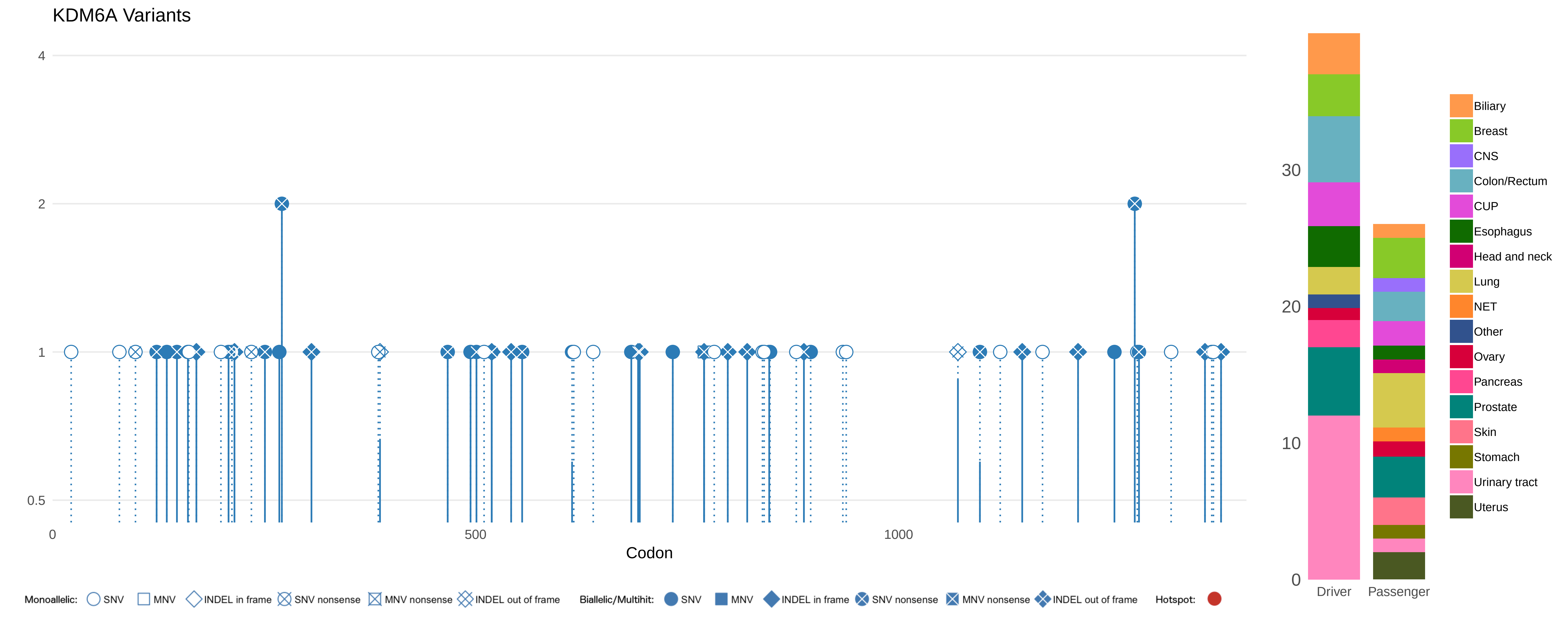
KDM6A Variants
4
2
1
0.5
0
500
1000
Codon
30
20
10
0
Driver
Passenger
Biliary
Breast
CNS
Colon/Rectum
CUP
Esophagus
Head and neck
Lung
NET
Other
Ovary
Pancreas
Prostate
Skin
Stomach
Urinary tract
Uterus
Monoallelic:
SNV
MNV
INDEL in frame
SNV nonsense
MNV nonsense
INDEL out of frame
Biallelic/Multihit:
SNV
MNV
INDEL in frame
SNV nonsense
MNV nonsense
INDEL out of frame
Hotspot:

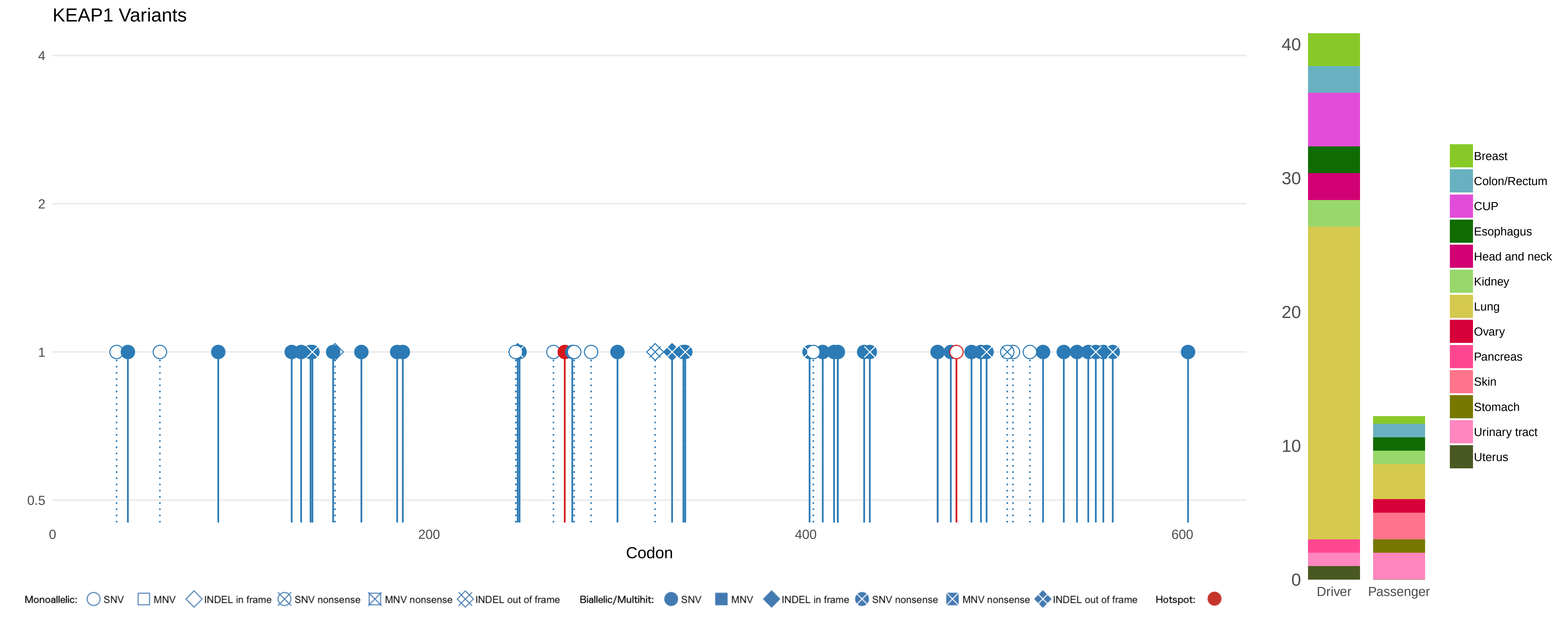

KEAP1 Variants
4
2
1
0.5
0
200
400
600
Codon
40
30
20
10
0
Driver
Passenger
Breast
Colon/Rectum
CUP
Esophagus
Head and neck
Kidney
Lung
Ovary
Pancreas
Skin
Stomach
Urinary tract
Uterus
Monoallelic: SNV MNV INDEL in frame SNV nonsense MNV nonsense INDEL out of frame
Biallelic/Multihit: SNV MNV INDEL in frame SNV nonsense MNV nonsense INDEL out of frame
Hotspot:

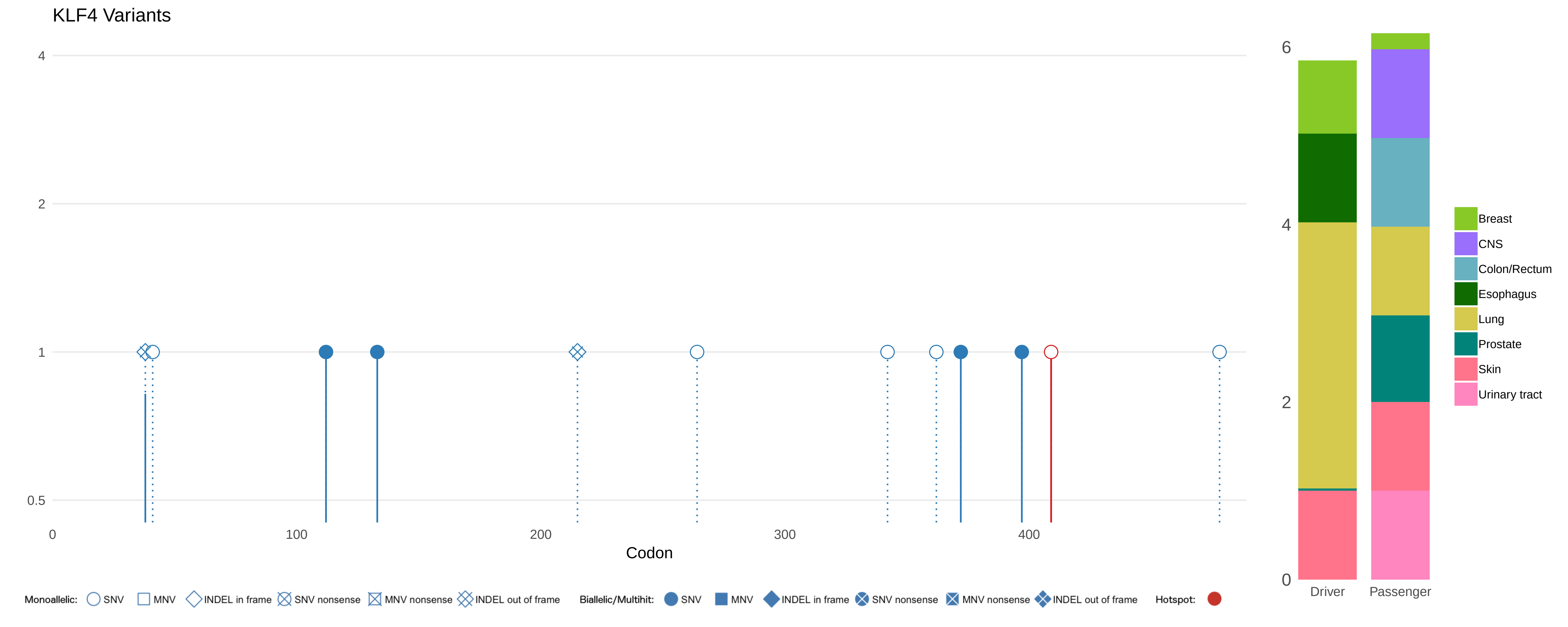

KLF4 Variants
4
2
1
0.5
0
100
200
300
400
Codon
Monoallelic: SNV MNV INDEL in frame SNV nonsense MNV nonsense INDEL out of frame
Biallelic/Multihit: SNV MNV INDEL in frame SNV nonsense MNV nonsense INDEL out of frame
Hotspot:
6
4
2
0
Driver
Passenger
Breast
CNS
Colon/Rectum
Esophagus
Lung
Prostate
Skin
Urinary tract

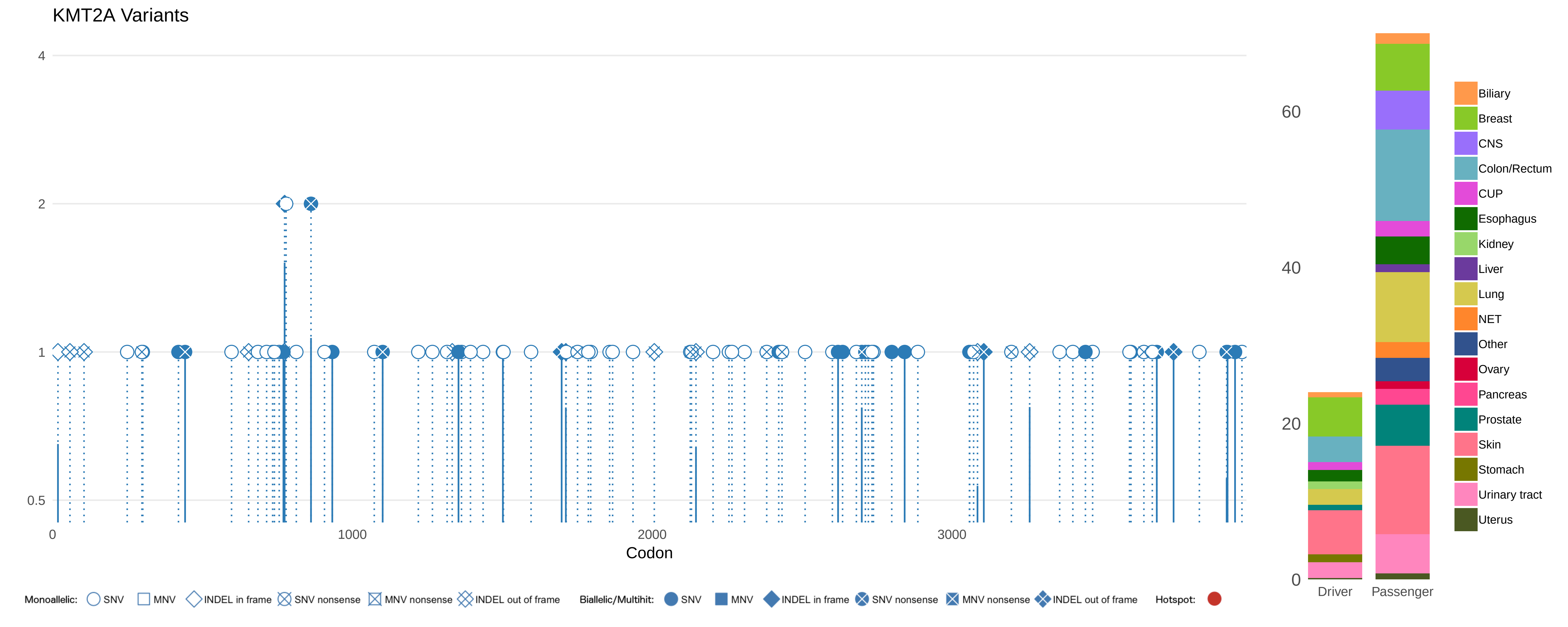

KMT2A Variants
4
2
1
0.5
0
1000
2000
3000
Codon
60
40
20
0
Driver
Passenger
Biliary
Breast
CNS
Colon/Rectum
CUP
Esophagus
Kidney
Liver
Lung
NET
Other
Ovary
Pancreas
Prostate
Skin
Stomach
Urinary tract
Uterus
Monoallelic: SNV MNV INDEL in frame SNV nonsense MNV nonsense INDEL out of frame
Biallelic/Multihit: SNV MNV INDEL in frame SNV nonsense MNV nonsense INDEL out of frame
Hotspot:

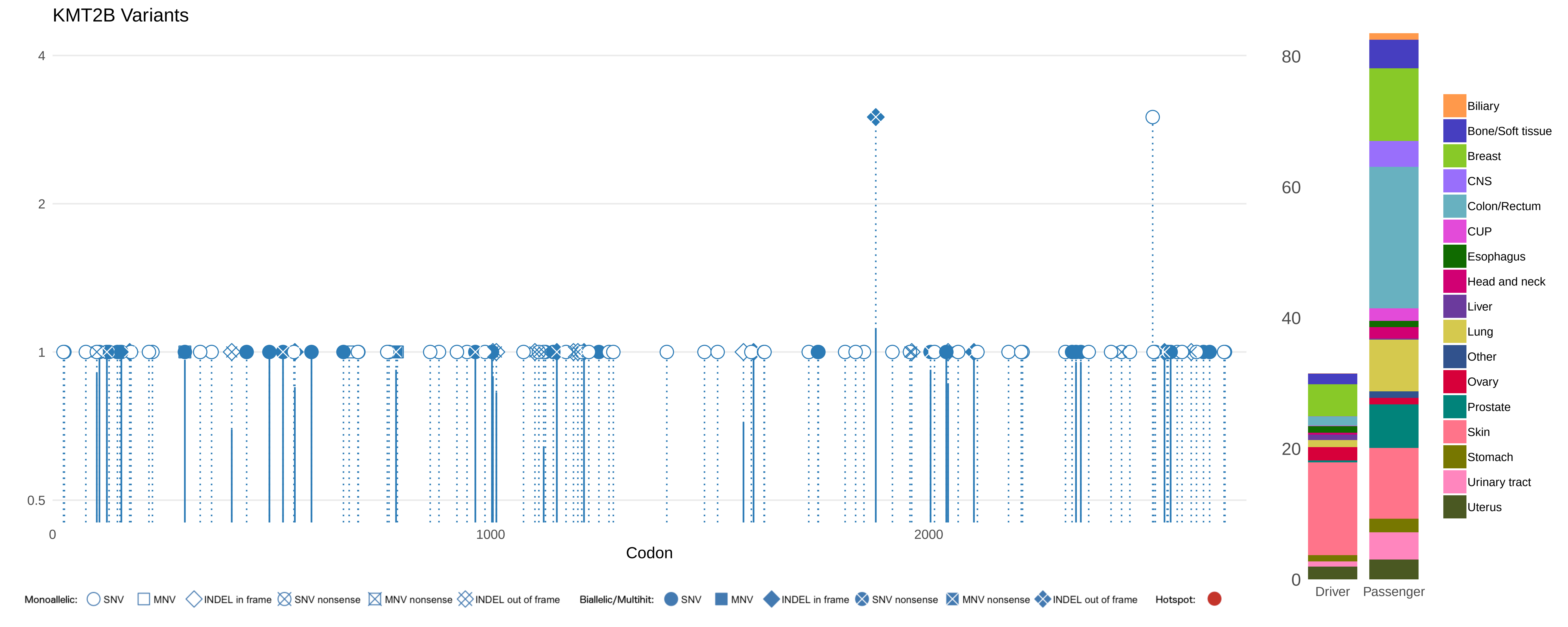
KMT2B Variants
4
2
1
0.5
0
1000
2000
Codon
80
60
40
20
0
Driver
Passenger
Biliary
Bone/Soft tissue
Breast
CNS
Colon/Rectum
CUP
Esophagus
Head and neck
Liver
Lung
Other
Ovary
Prostate
Skin
Stomach
Urinary tract
Uterus
Monoallelic: SNV MNV INDEL in frame SNV nonsense MNV nonsense INDEL out of frame
Biallelic/Multihit: SNV MNV INDEL in frame SNV nonsense MNV nonsense INDEL out of frame
Hotspot:

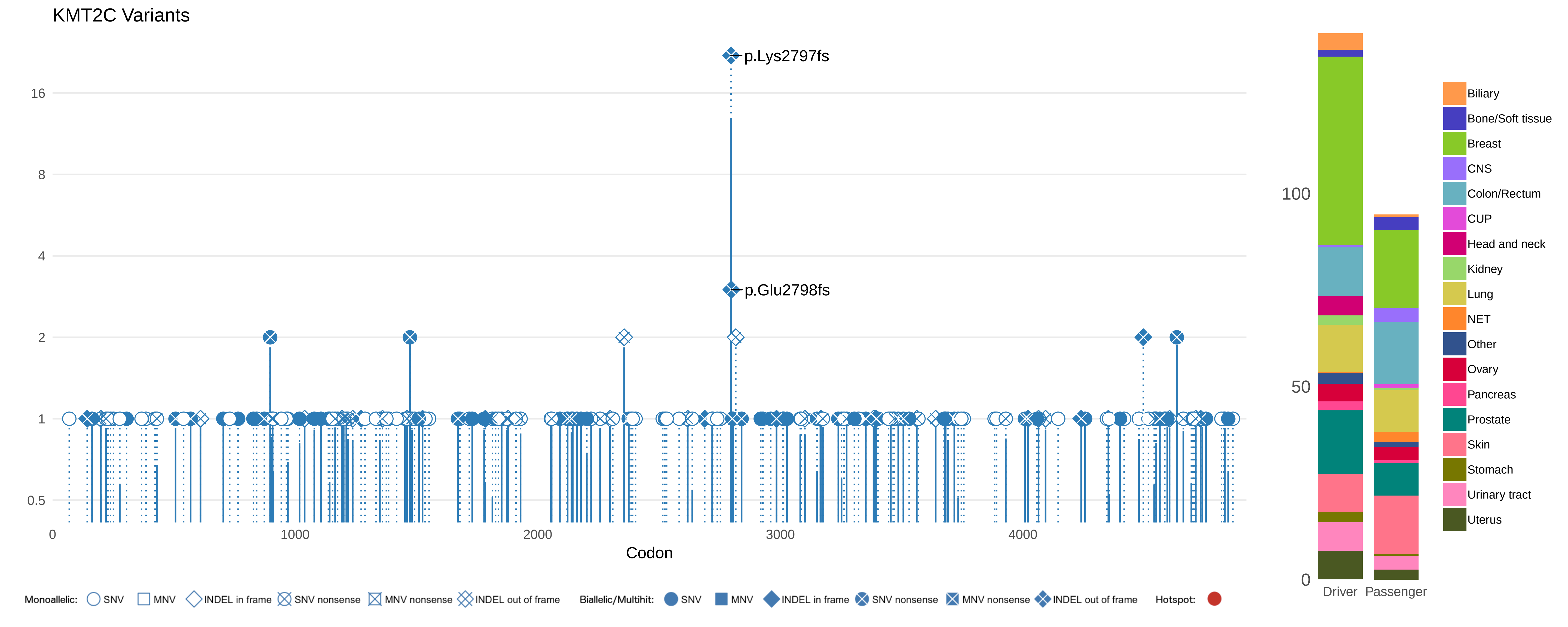

KMT2C Variants
p.Lys2797fs
p.Glu2798fs
16
8
4
2
1
0.5
0
1000
2000
3000
4000
Codon
100
50
0
Driver
Passenger
Biliary
Bone/Soft tissue
Breast
CNS
Colon/Rectum
CUP
Head and neck
Kidney
Lung
NET
Other
Ovary
Pancreas
Prostate
Skin
Stomach
Urinary tract
Uterus
Monoallelic: SNV MNV INDEL in frame SNV nonsense MNV nonsense INDEL out of frame
Biallelic/Multihit: SNV MNV INDEL in frame SNV nonsense MNV nonsense INDEL out of frame
Hotspot:

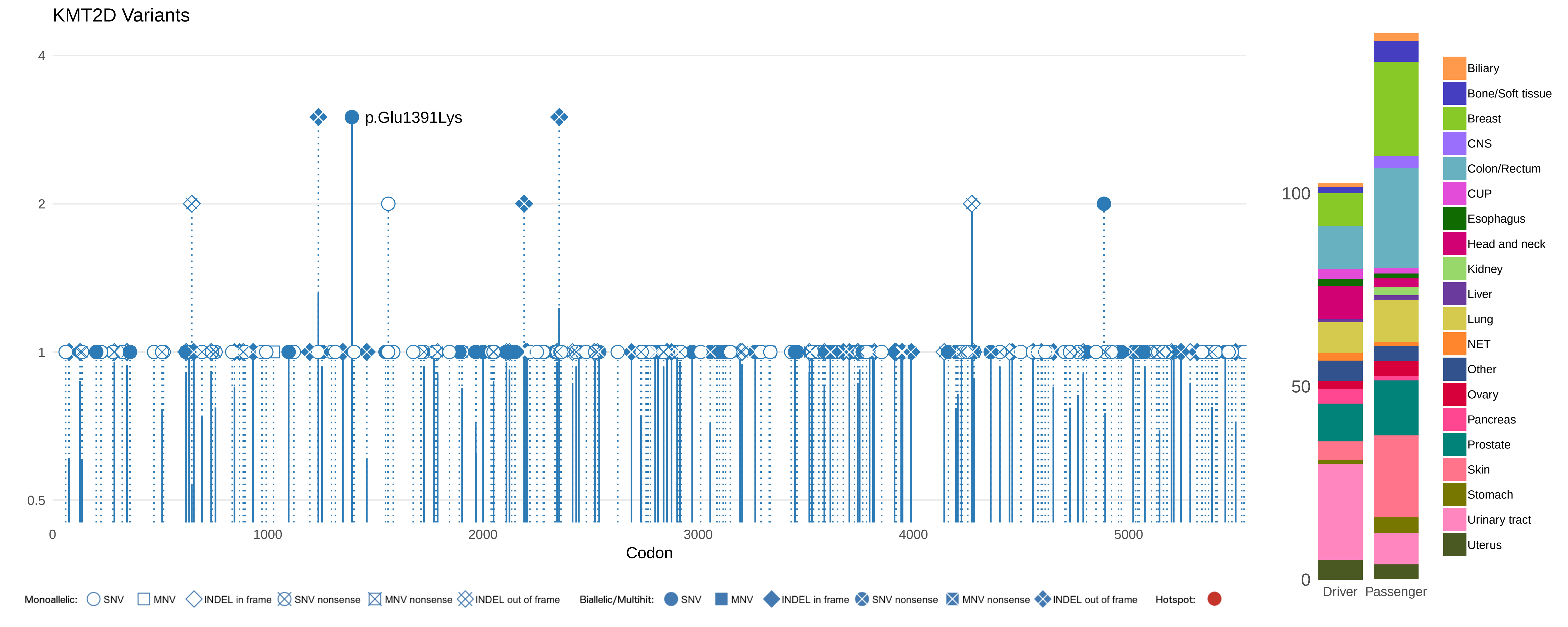
KMT2D Variants
p.Glu1391Lys
4
2
1
0.5
0
1000
2000
3000
4000
5000
Codon
Monoallelic: SNV MNV INDEL in frame SNV nonsense MNV nonsense INDEL out of frame
Biallelic/Multihit: SNV MNV INDEL in frame SNV nonsense MNV nonsense INDEL out of frame
Hotspot:
100
50
0
Driver
Passenger
Biliary
Bone/Soft tissue
Breast
CNS
Colon/Rectum
CUP
Esophagus
Head and neck
Kidney
Liver
Lung
NET
Other
Ovary
Pancreas
Prostate
Skin
Stomach
Urinary tract
Uterus

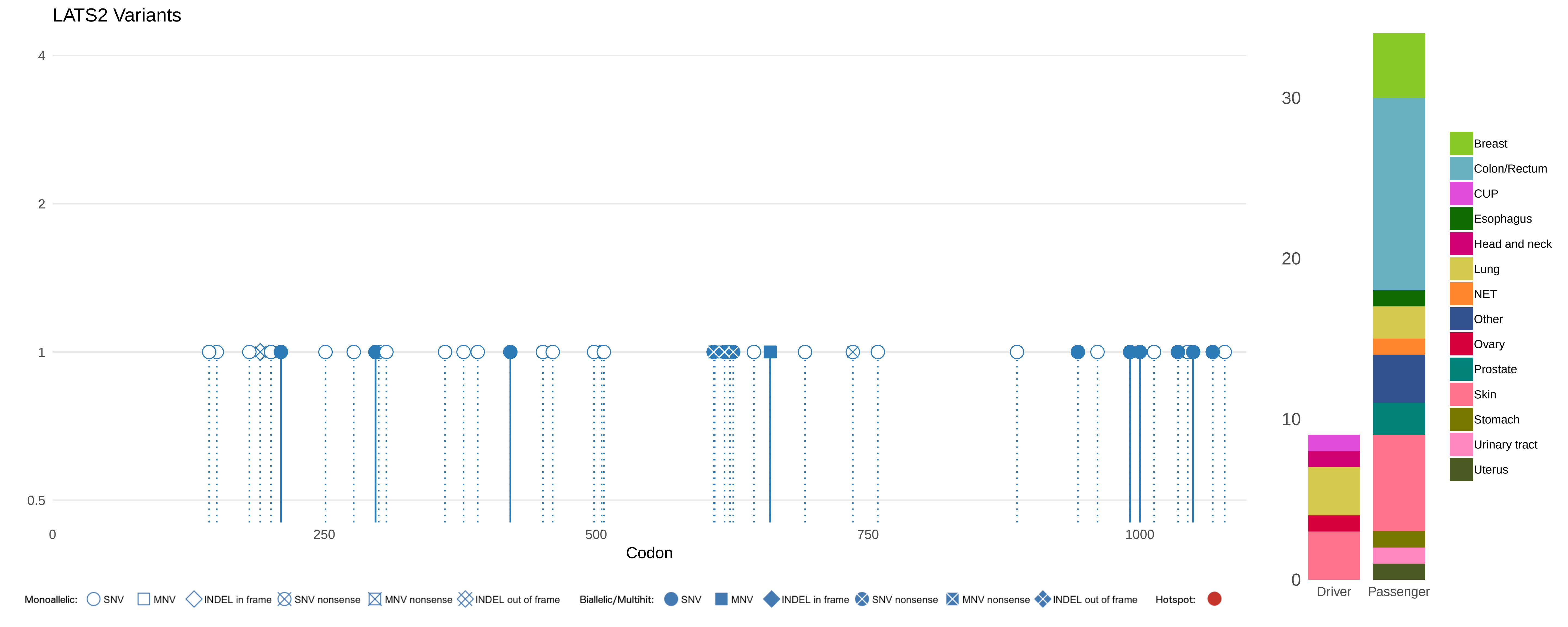

LATS2 Variants
4
2
1
0.5
0
250
500
750
1000
Codon
30
20
10
0
Driver
Passenger
Breast
Colon/Rectum
CUP
Esophagus
Head and neck
Lung
NET
Other
Ovary
Prostate
Skin
Stomach
Urinary tract
Uterus
Monoallelic: SNV MNV INDEL in frame SNV nonsense MNV nonsense INDEL out of frame
Biallelic/Multihit: SNV MNV INDEL in frame SNV nonsense MNV nonsense INDEL out of frame
Hotspot:

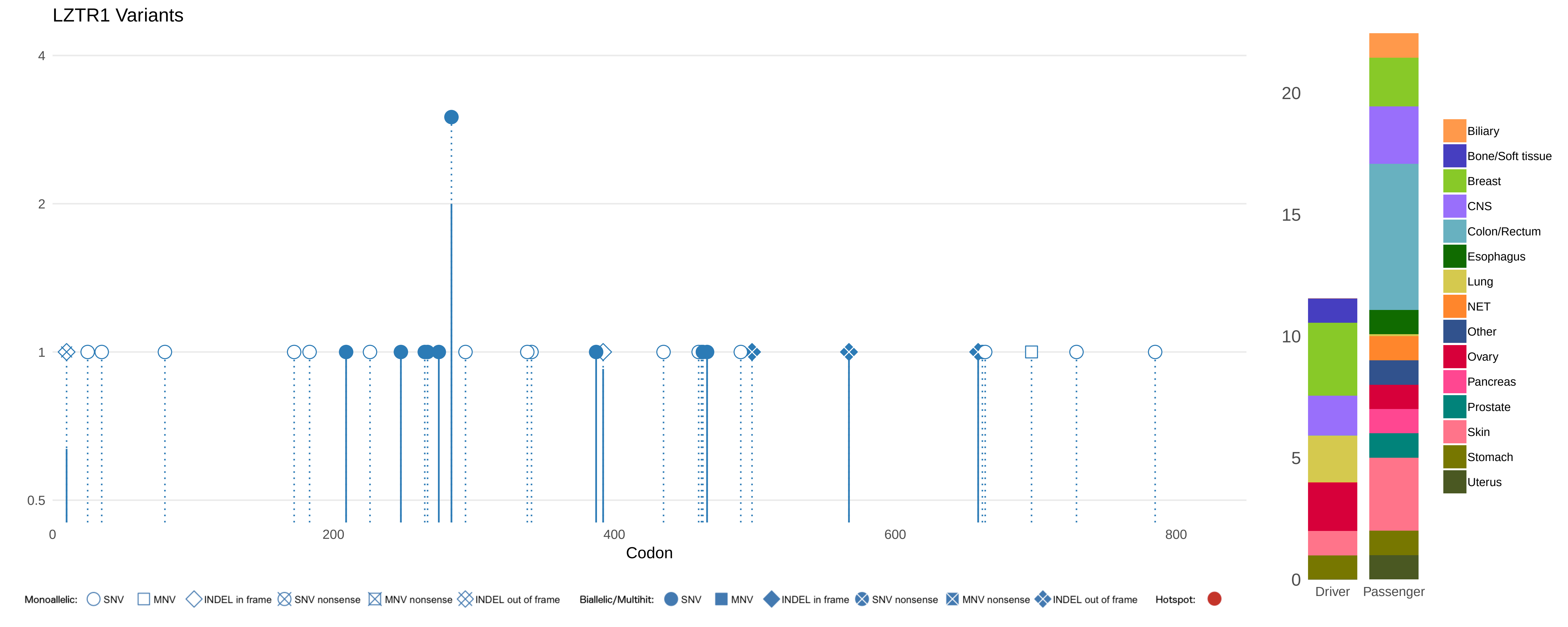

LZTR1 Variants
4
2
1
0.5
0
200
400
600
800
Codon
20
15
10
5
0
Driver
Passenger
Biliary
Bone/Soft tissue
Breast
CNS
Colon/Rectum
Esophagus
Lung
NET
Other
Ovary
Pancreas
Prostate
Skin
Stomach
Uterus
Monoallelic: SNV MNV INDEL in frame SNV nonsense MNV nonsense INDEL out of frame
Biallelic/Multihit: SNV MNV INDEL in frame SNV nonsense MNV nonsense INDEL out of frame
Hotspot:

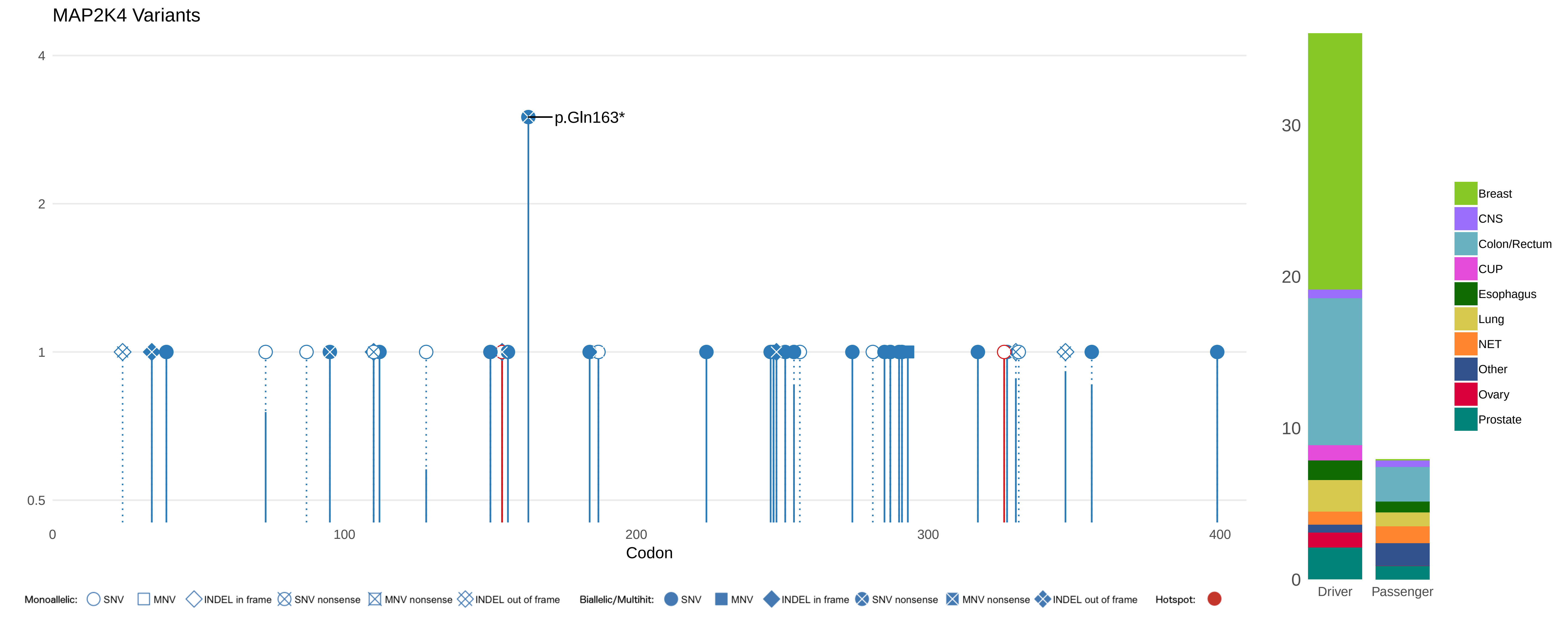
MAP2K4 Variants
p.Gln163*
4
2
1
0.5
0
100
200
300
400
Codon
30
20
10
0
Driver
Passenger
Breast
CNS
Colon/Rectum
CUP
Esophagus
Lung
NET
Other
Ovary
Prostate
Monoallelic: SNV MNV INDEL in frame SNV nonsense MNV nonsense INDEL out of frame
Biallelic/Multihit: SNV MNV INDEL in frame SNV nonsense MNV nonsense INDEL out of frame
Hotspot:

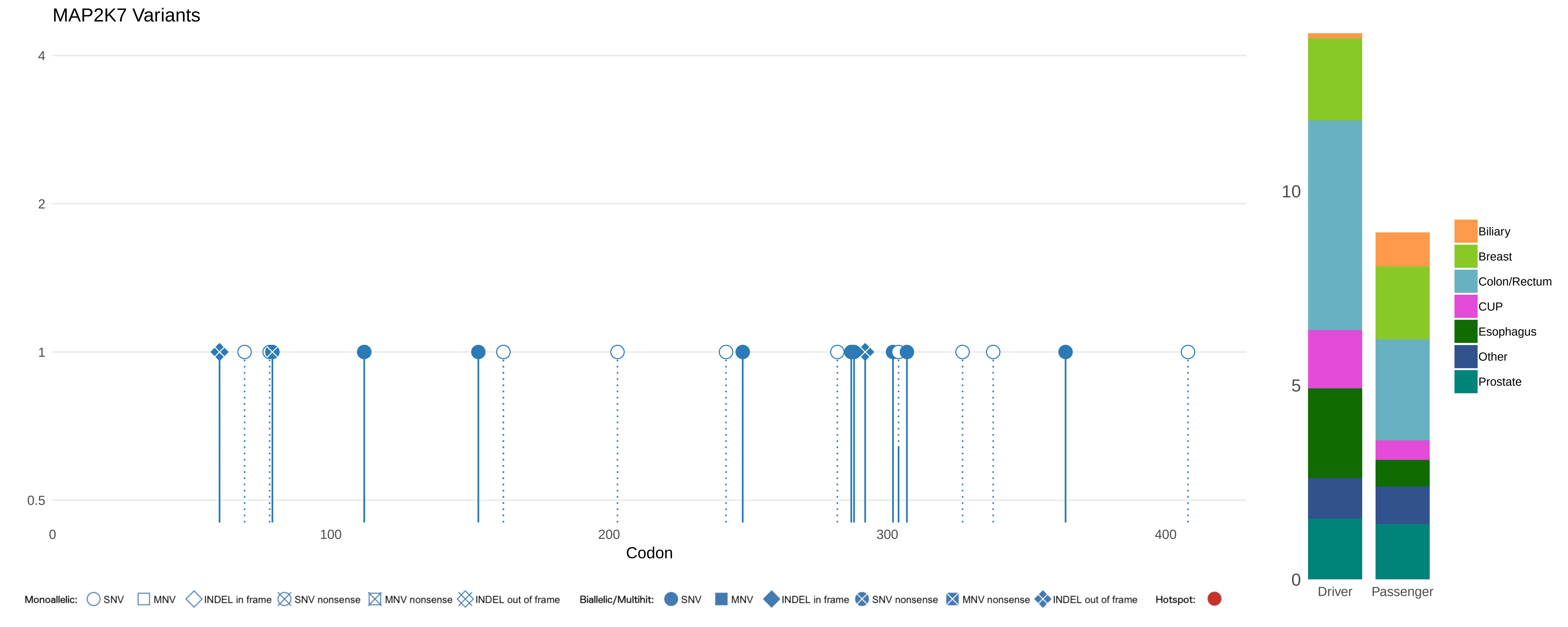

MAP2K7 Variants
4
2
1
0.5
0
100
200
300
400
Codon
Monoallelic: SNV MNV INDEL in frame SNV nonsense MNV nonsense INDEL out of frame
Biallelic/Multihit: SNV MNV INDEL in frame SNV nonsense MNV nonsense INDEL out of frame
Hotspot:
10
5
0
Driver
Passenger
Biliary
Breast
Colon/Rectum
CUP
Esophagus
Other
Prostate

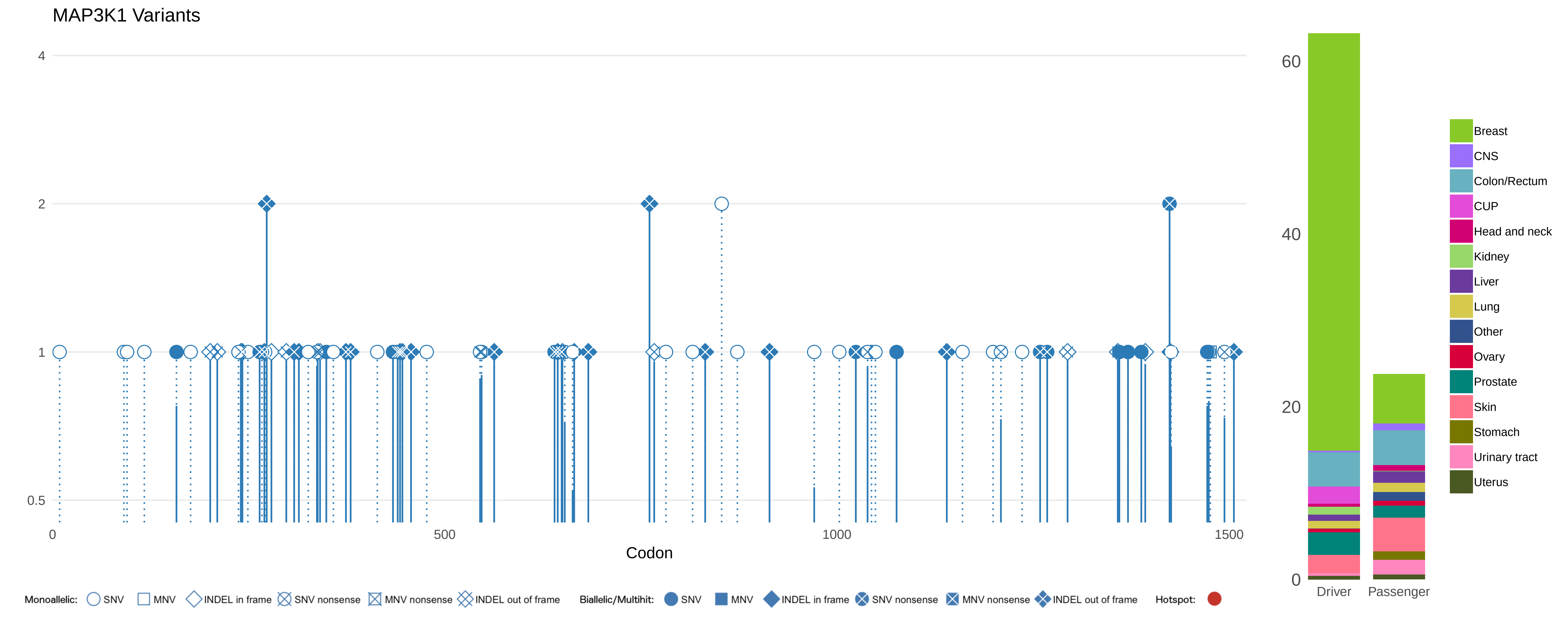
MAP3K1 Variants
4
2
1
0.5
0
500
1000
1500
Codon
60
40
20
0
Driver
Passenger
Breast
CNS
Colon/Rectum
CUP
Head and neck
Kidney
Liver
Lung
Other
Ovary
Prostate
Skin
Stomach
Urinary tract
Uterus
Monoallelic: SNV MNV INDEL in frame SNV nonsense MNV nonsense INDEL out of frame
Biallelic/Multihit: SNV MNV INDEL in frame SNV nonsense MNV nonsense INDEL out of frame
Hotspot:

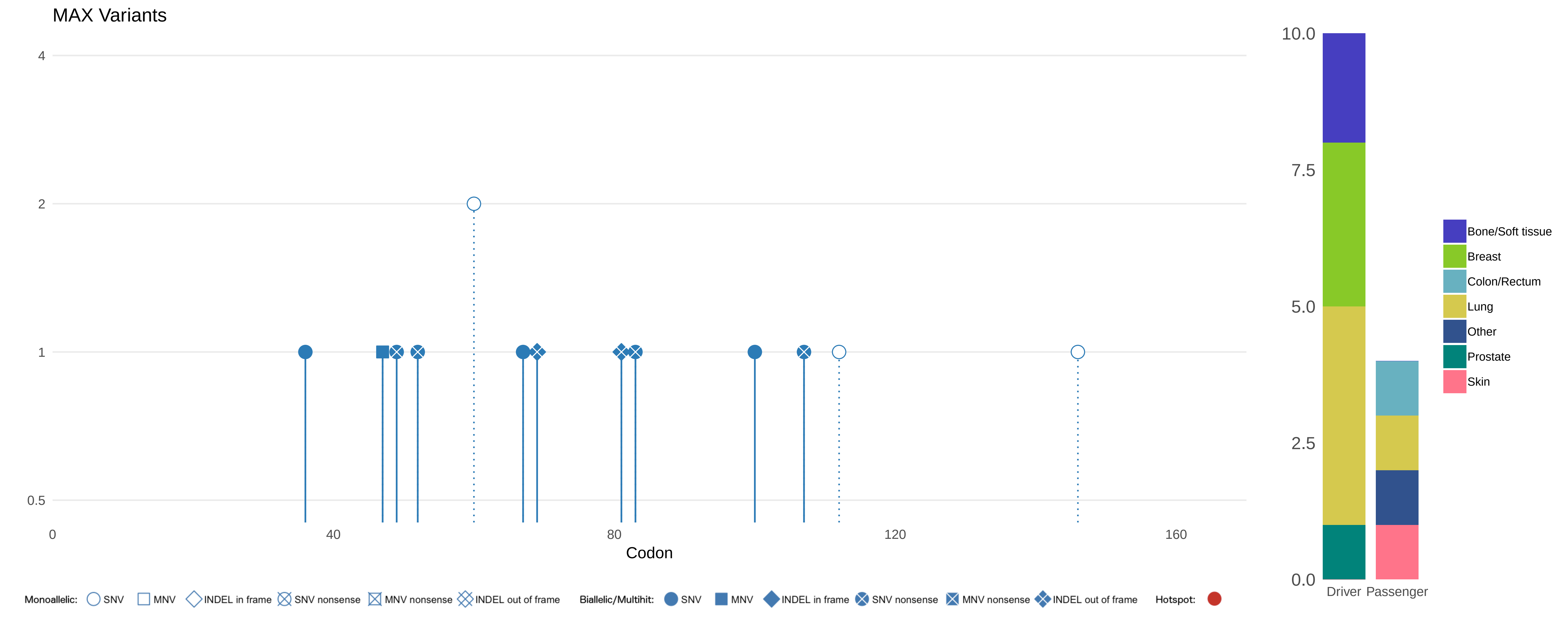

MAX Variants
4
2
1
0.5
0
40
80
120
160
Codon
Monoallelic: SNV MNV INDEL in frame SNV nonsense MNV nonsense INDEL out of frame
Biallelic/Multihit: SNV MNV INDEL in frame SNV nonsense MNV nonsense INDEL out of frame
Hotspot:
10.0
7.5
5.0
2.5
0.0
Driver
Passenger
Bone/Soft tissue
Breast
Colon/Rectum
Lung
Other
Prostate
Skin

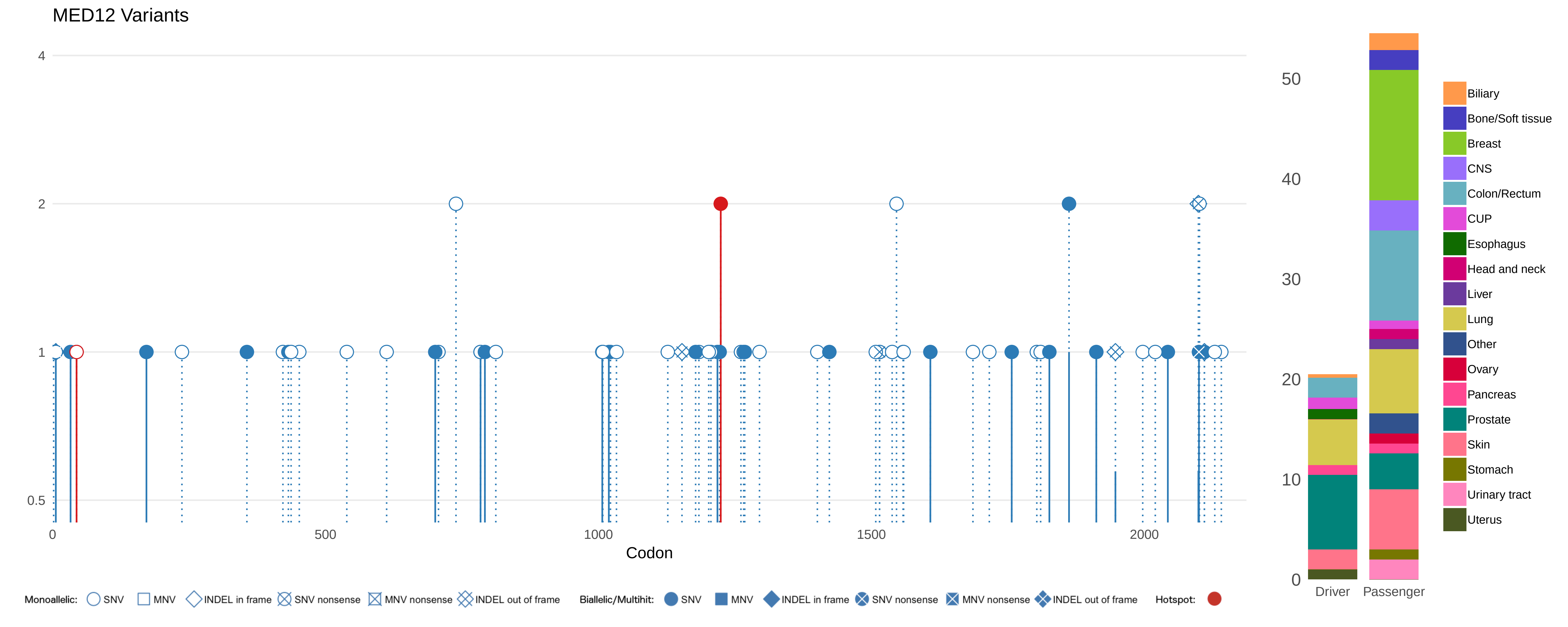
MED12 Variants
4
2
1
0.5
0
500
1000
1500
2000
Codon
Monoallelic: SNV MNV INDEL in frame SNV nonsense MNV nonsense INDEL out of frame
Biallelic/Multihit: SNV MNV INDEL in frame SNV nonsense MNV nonsense INDEL out of frame
Hotspot:
50
40
30
20
10
0
Driver
Passenger
Biliary
Bone/Soft tissue
Breast
CNS
Colon/Rectum
CUP
Esophagus
Head and neck
Liver
Lung
Other
Ovary
Pancreas
Prostate
Skin
Stomach
Urinary tract
Uterus

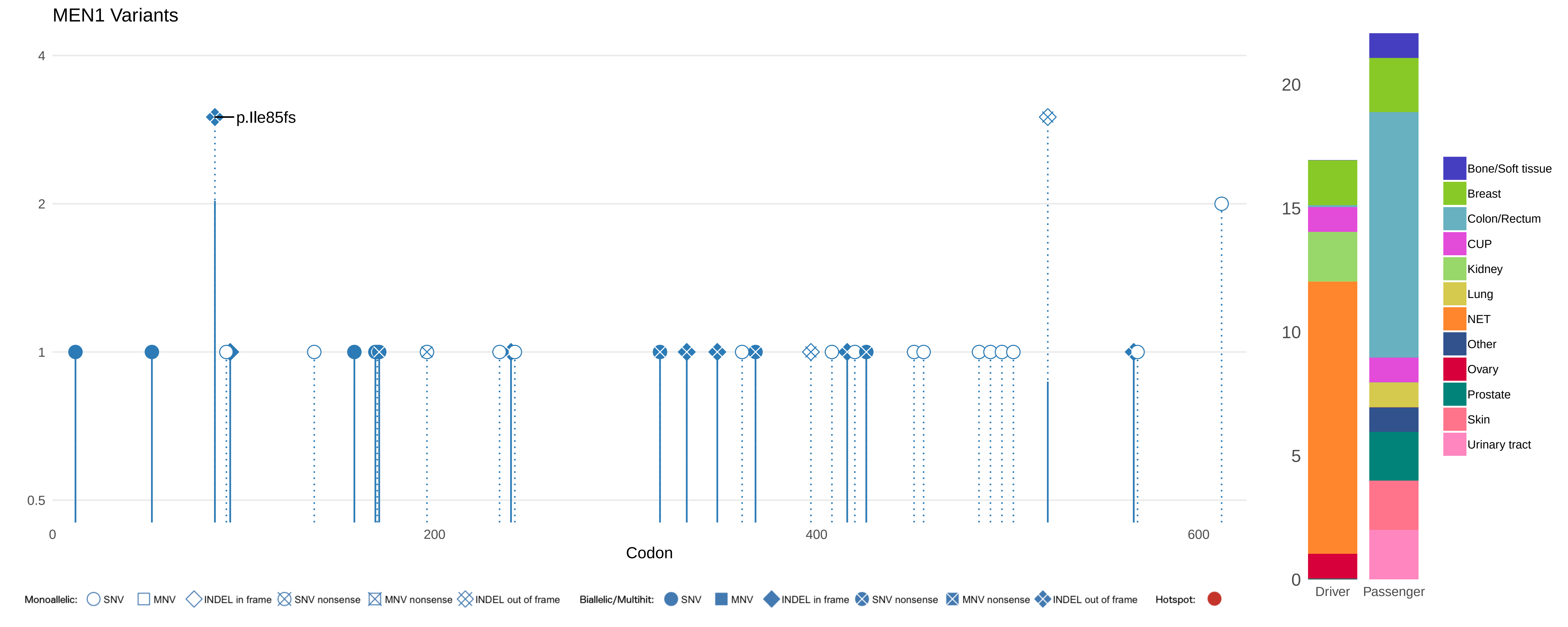

MEN1 Variants
p.Ile85fs
4
2
1
0.5
0
200
400
600
Codon
20
15
10
5
0
Driver
Passenger
Bone/Soft tissue
Breast
Colon/Rectum
CUP
Kidney
Lung
NET
Other
Ovary
Prostate
Skin
Urinary tract
Monoallelic:
SNV
MNV
INDEL in frame
SNV nonsense
MNV nonsense
INDEL out of frame
Biallelic/Multihit:
SNV
MNV
INDEL in frame
SNV nonsense
MNV nonsense
INDEL out of frame
Hotspot:

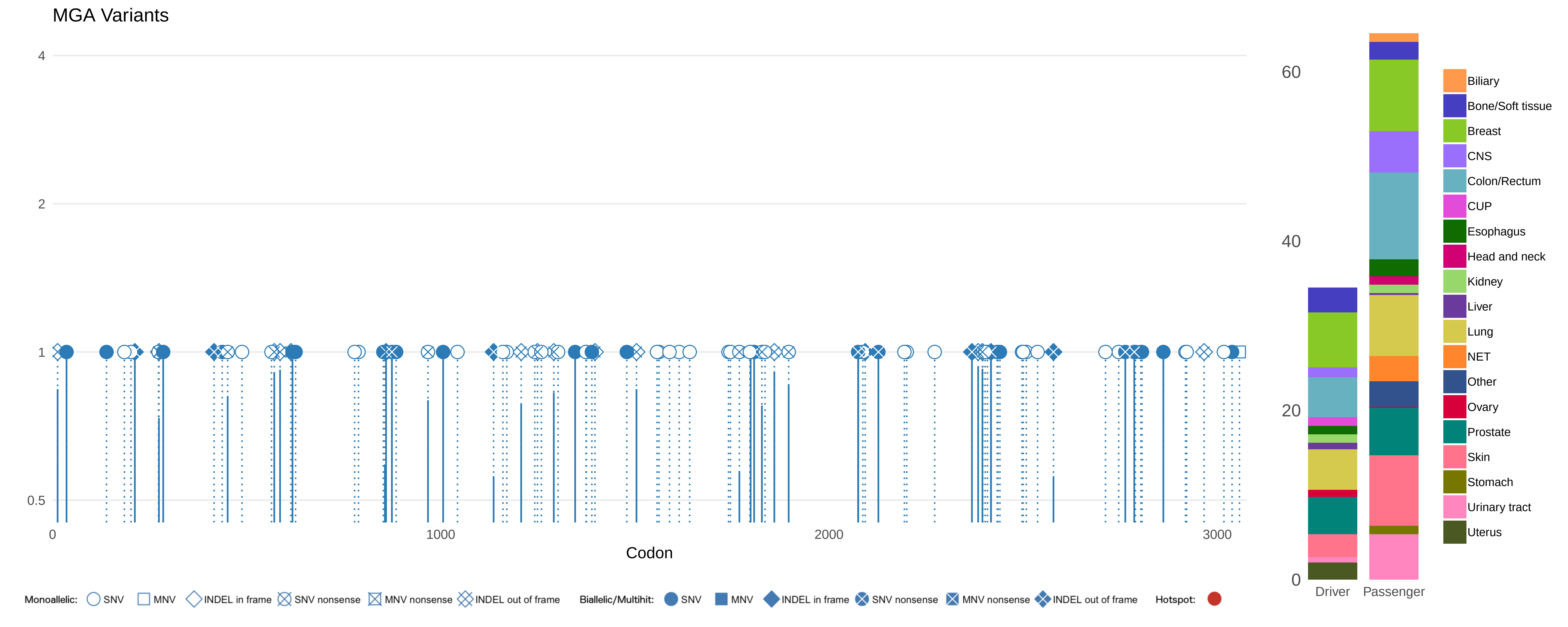

MGA Variants
4
2
1
0.5
0
1000
2000
3000
Codon
Monoallelic: SNV MNV INDEL in frame SNV nonsense MNV nonsense INDEL out of frame
Biallelic/Multihit: SNV MNV INDEL in frame SNV nonsense MNV nonsense INDEL out of frame
Hotspot:
60
40
20
0
Driver
Passenger
Biliary
Bone/Soft tissue
Breast
CNS
Colon/Rectum
CUP
Esophagus
Head and neck
Kidney
Liver
Lung
NET
Other
Ovary
Prostate
Skin
Stomach
Urinary tract
Uterus

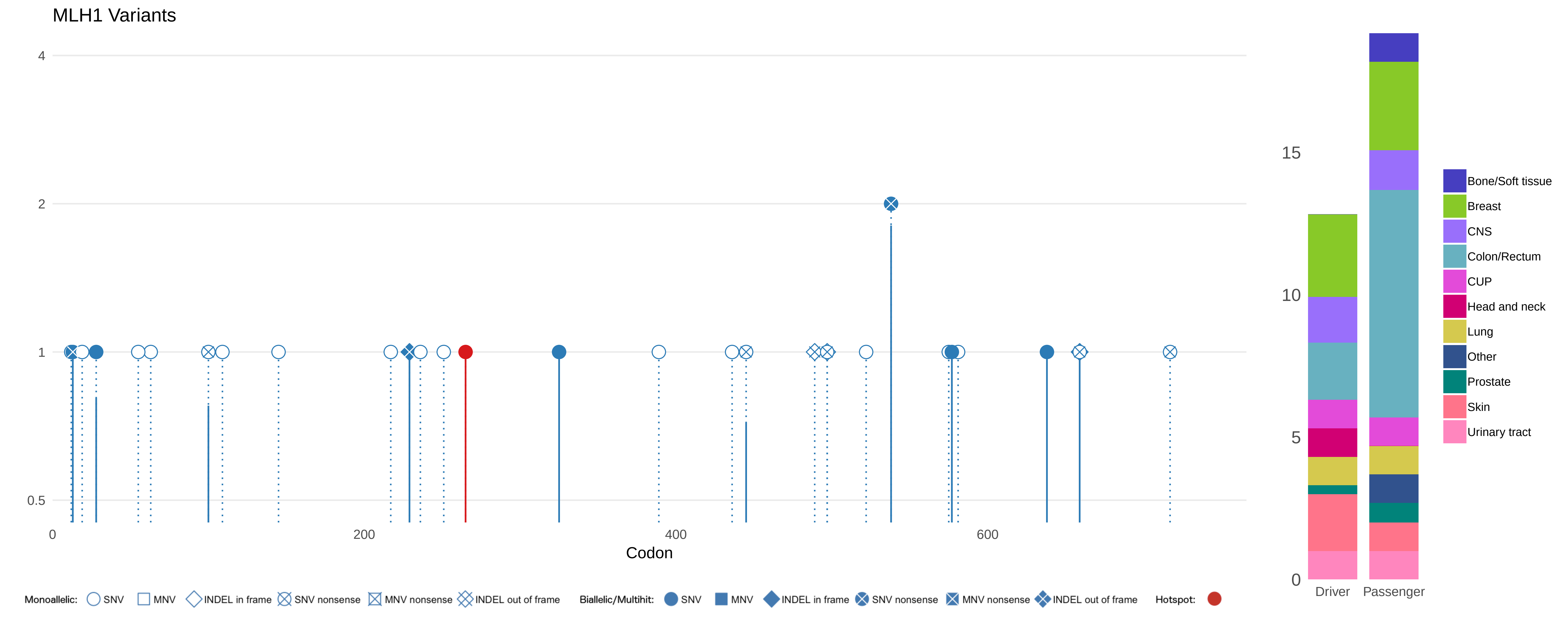

MLH1 Variants
4
2
1
0.5
0
200
400
600
Codon
Monoallelic: SNV MNV INDEL in frame SNV nonsense MNV nonsense INDEL out of frame
Biallelic/Multihit: SNV MNV INDEL in frame SNV nonsense MNV nonsense INDEL out of frame
Hotspot:
15
10
5
0
Driver
Passenger
Bone/Soft tissue
Breast
CNS
Colon/Rectum
CUP
Head and neck
Lung
Other
Prostate
Skin
Urinary tract

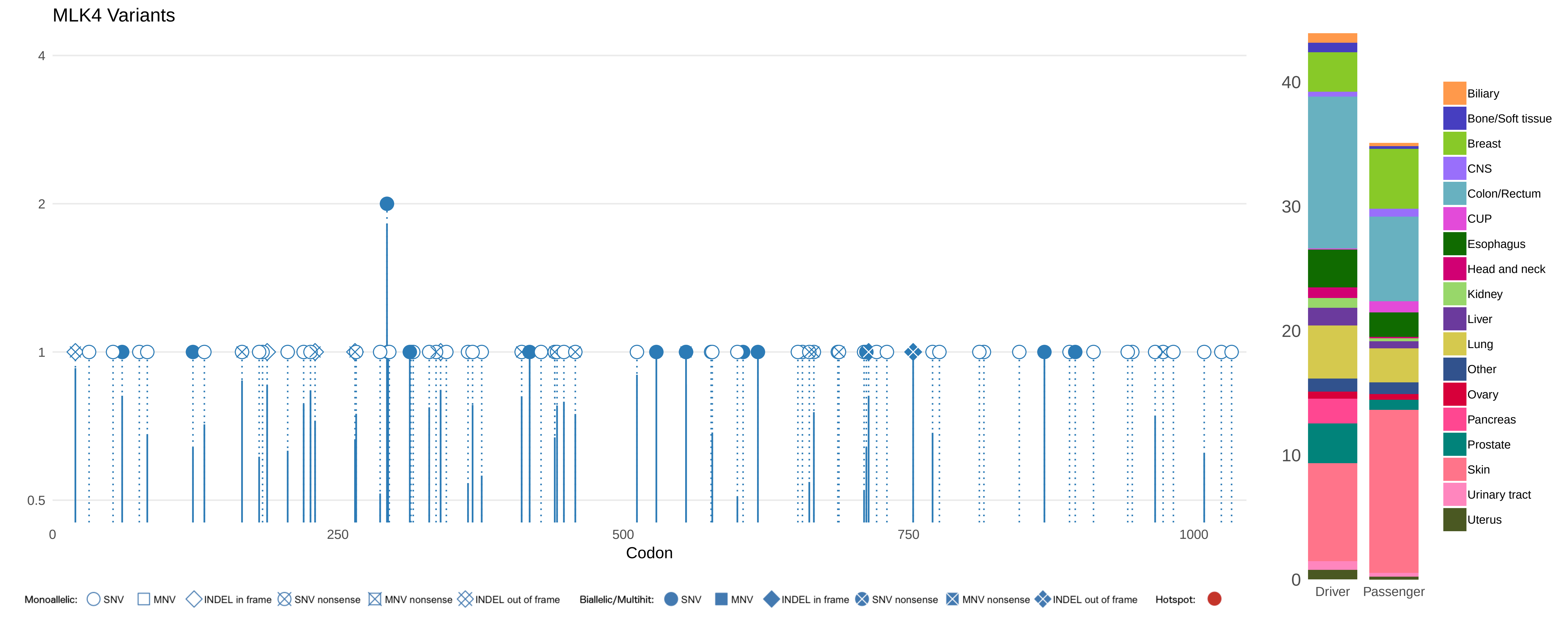

MLK4 Variants
4
2
1
0.5
0
250
500
750
1000
Codon
40
30
20
10
0
Driver
Passenger
Biliary
Bone/Soft tissue
Breast
CNS
Colon/Rectum
CUP
Esophagus
Head and neck
Kidney
Liver
Lung
Other
Ovary
Pancreas
Prostate
Skin
Urinary tract
Uterus
Monoallelic: SNV MNV INDEL in frame SNV nonsense MNV nonsense INDEL out of frame
Biallelic/Multihit: SNV MNV INDEL in frame SNV nonsense MNV nonsense INDEL out of frame
Hotspot:

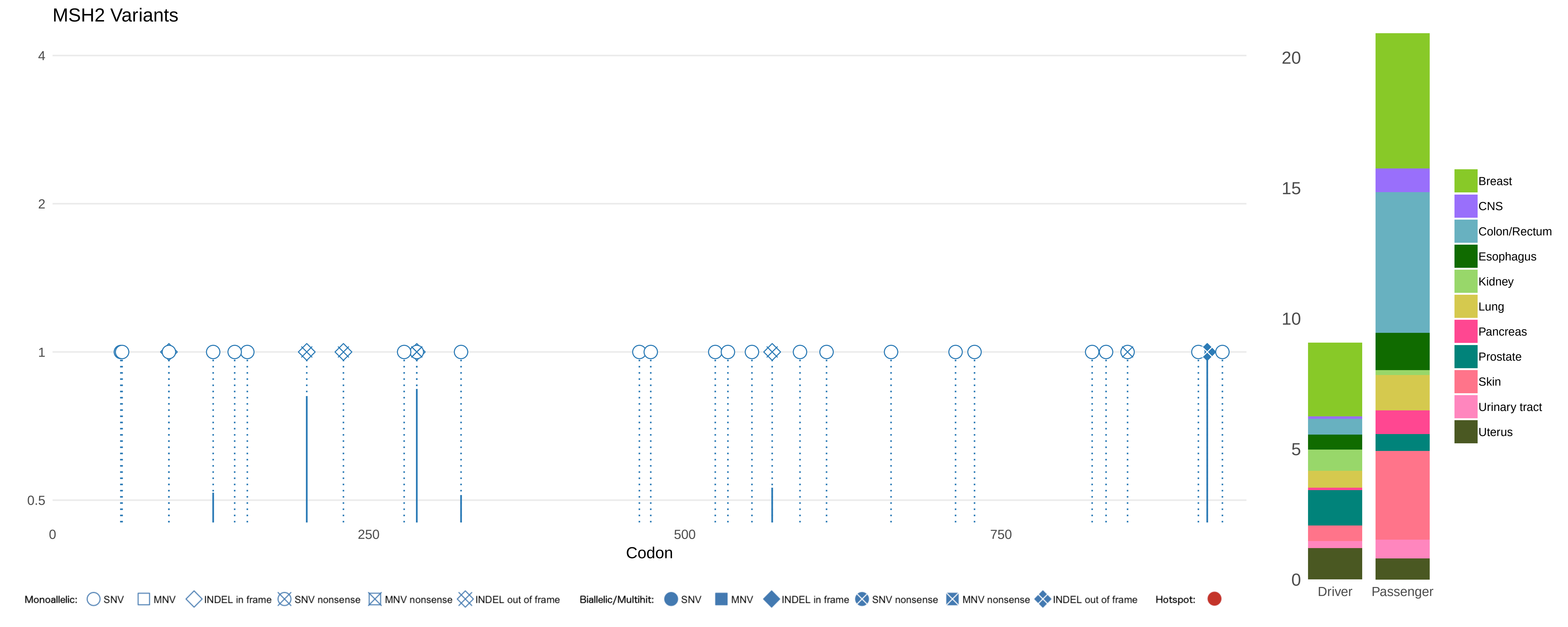
MSH2 Variants
4
2
1
0.5
0
250
500
750
Codon
20
15
10
5
0
Driver
Passenger
Breast
CNS
Colon/Rectum
Esophagus
Kidney
Lung
Pancreas
Prostate
Skin
Urinary tract
Uterus
Monoallelic: SNV MNV INDEL in frame SNV nonsense MNV nonsense INDEL out of frame
Biallelic/Multihit: SNV MNV INDEL in frame SNV nonsense MNV nonsense INDEL out of frame
Hotspot:

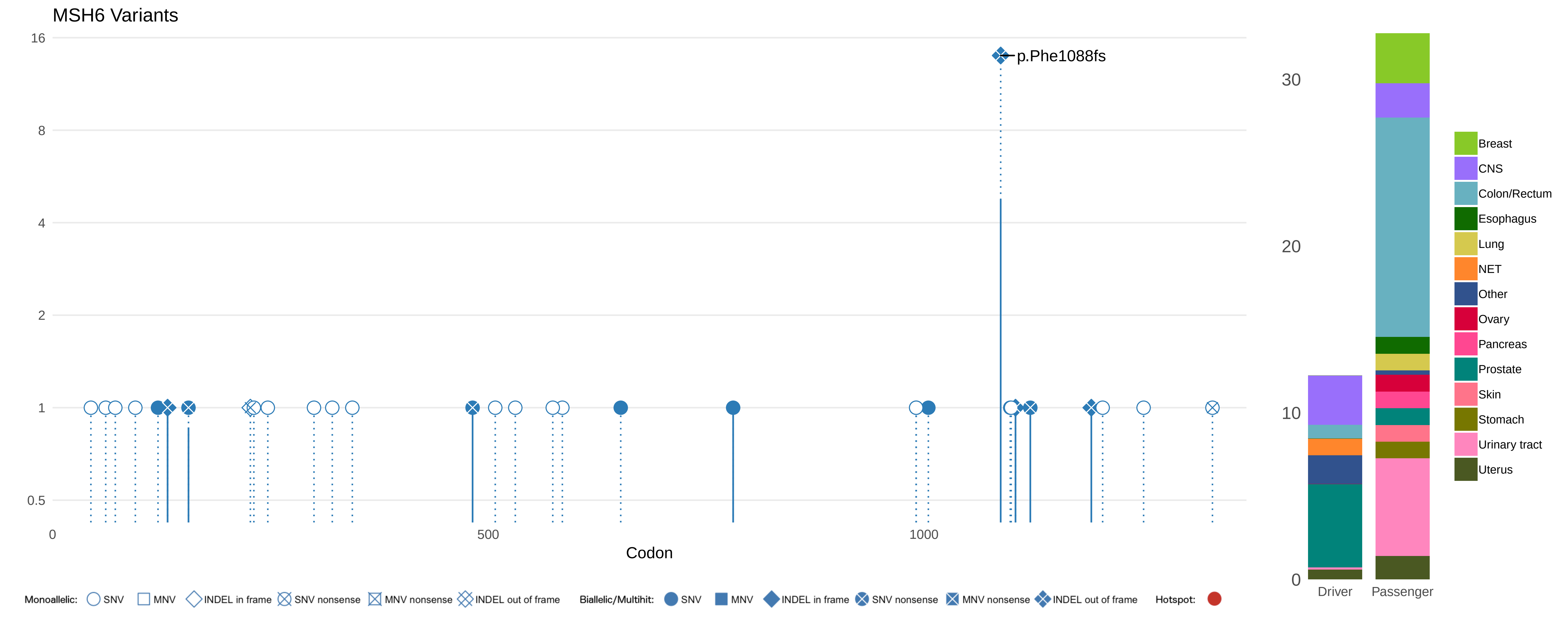

MSH6 Variants
16
8
4
2
1
0.5
0
500
1000
Codon
p.Phe1088fs
30
20
10
0
Driver
Passenger
Breast
CNS
Colon/Rectum
Esophagus
Lung
NET
Other
Ovary
Pancreas
Prostate
Skin
Stomach
Urinary tract
Uterus
Monoallelic: SNV MNV INDEL in frame SNV nonsense MNV nonsense INDEL out of frame
Biallelic/Multihit: SNV MNV INDEL in frame SNV nonsense MNV nonsense INDEL out of frame
Hotspot:

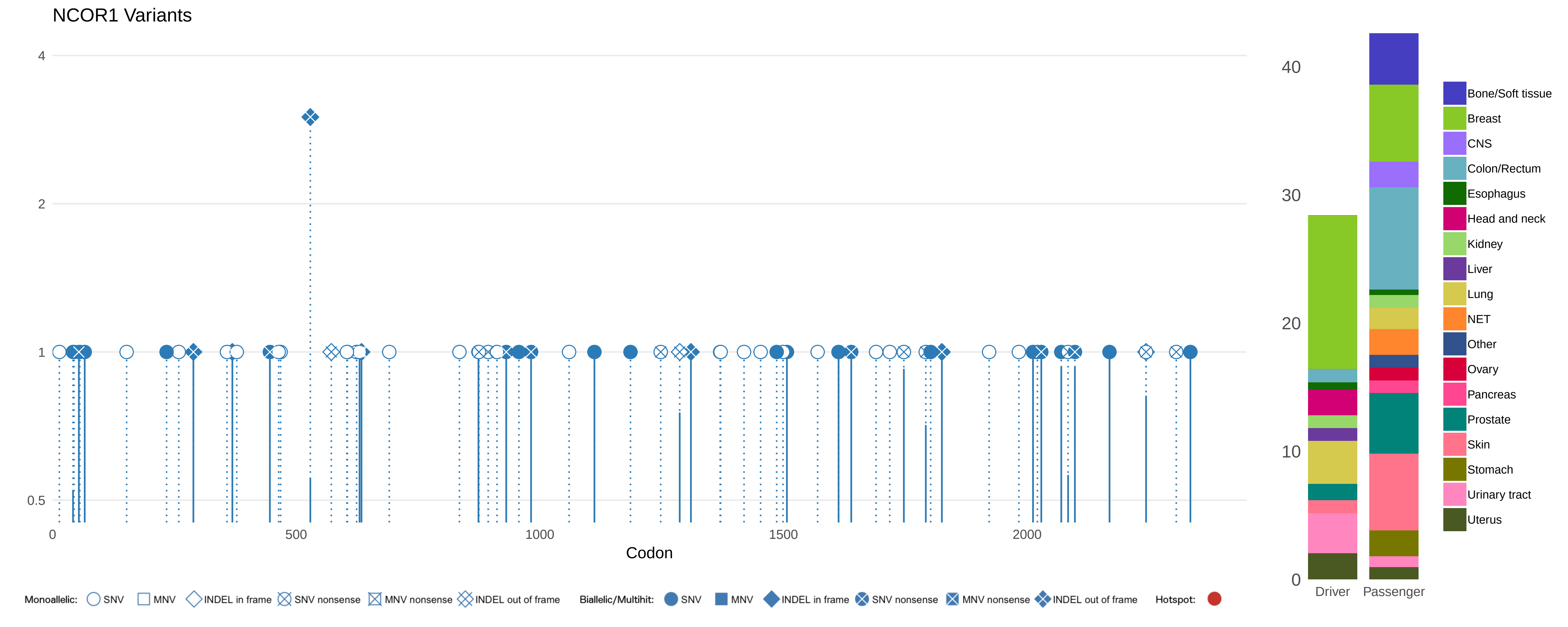

NCOR1 Variants
4
2
1
0.5
0
500
1000
1500
2000
Codon
40
30
20
10
0
Driver
Passenger
Bone/Soft tissue
Breast
CNS
Colon/Rectum
Esophagus
Head and neck
Kidney
Liver
Lung
NET
Other
Ovary
Pancreas
Prostate
Skin
Stomach
Urinary tract
Uterus
Monoallelic: SNV MNV INDEL in frame SNV nonsense MNV nonsense INDEL out of frame
Biallelic/Multihit: SNV MNV INDEL in frame SNV nonsense MNV nonsense INDEL out of frame
Hotspot:

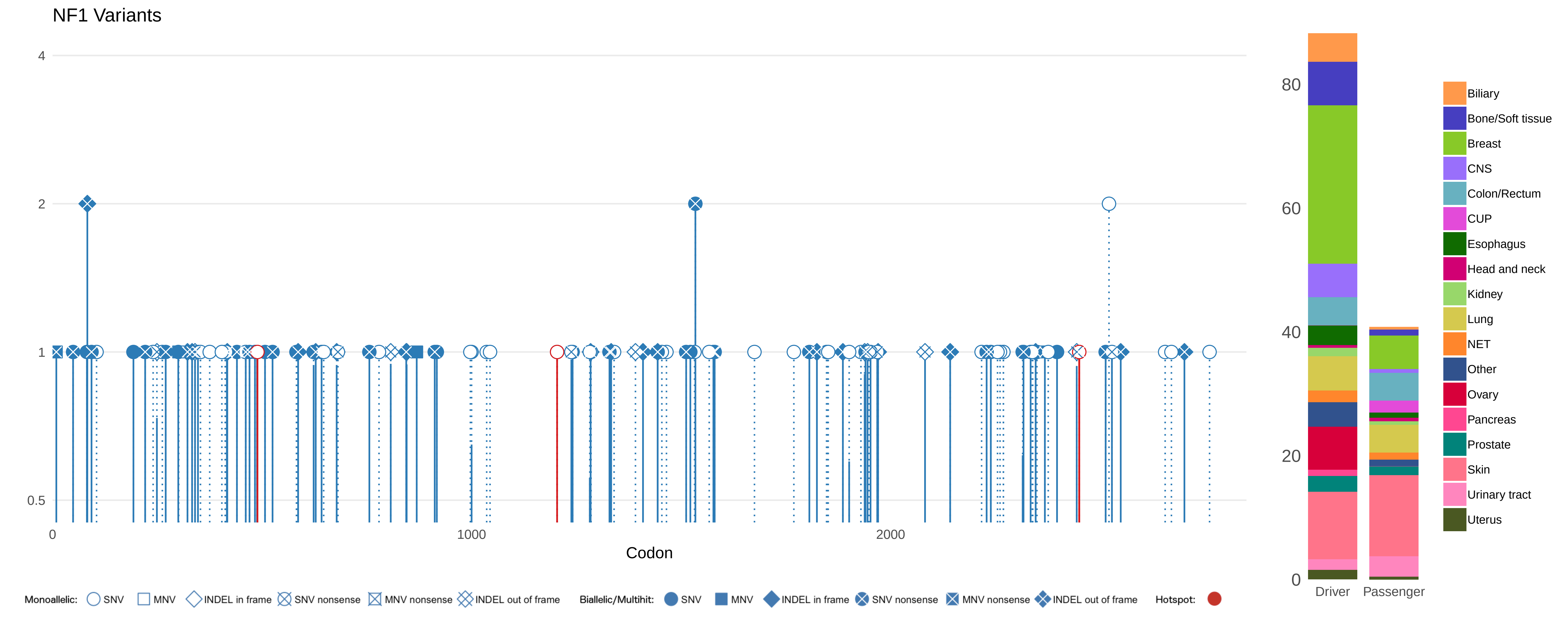
NF1 Variants
4
2
1
0.5
0
1000
2000
Codon
80
60
40
20
0
Driver
Passenger
Biliary
Bone/Soft tissue
Breast
CNS
Colon/Rectum
CUP
Esophagus
Head and neck
Kidney
Lung
NET
Other
Ovary
Pancreas
Prostate
Skin
Urinary tract
Uterus
Monoallelic: SNV MNV INDEL in frame SNV nonsense MNV nonsense INDEL out of frame
Biallelic/Multihit: SNV MNV INDEL in frame SNV nonsense MNV nonsense INDEL out of frame
Hotspot:

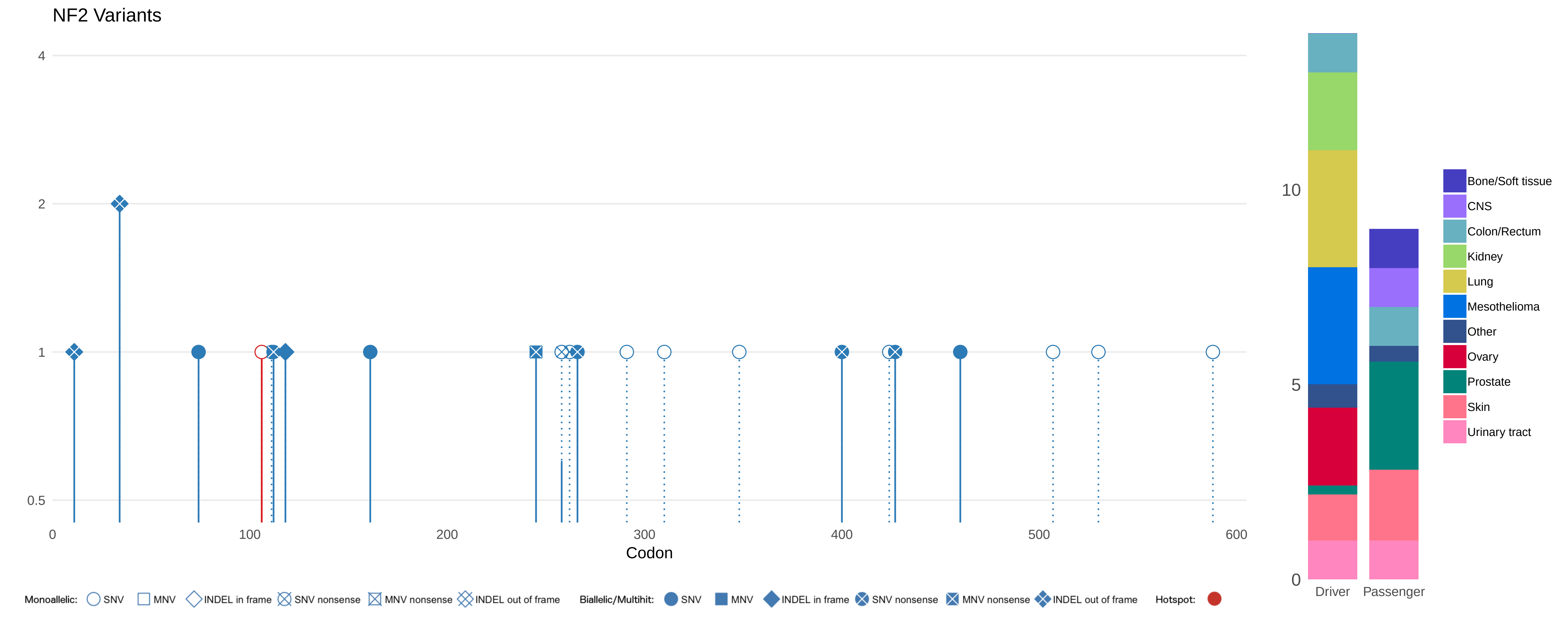
NF2 Variants
4
2
1
0.5
0
100
200
300
400
500
600
Codon
10
5
0
Driver
Passenger
Bone/Soft tissue
CNS
Colon/Rectum
Kidney
Lung
Mesothelioma
Other
Ovary
Prostate
Skin
Urinary tract
Monoallelic: SNV MNV INDEL in frame SNV nonsense MNV nonsense INDEL out of frame
Biallelic/Multihit: SNV MNV INDEL in frame SNV nonsense MNV nonsense INDEL out of frame
Hotspot:

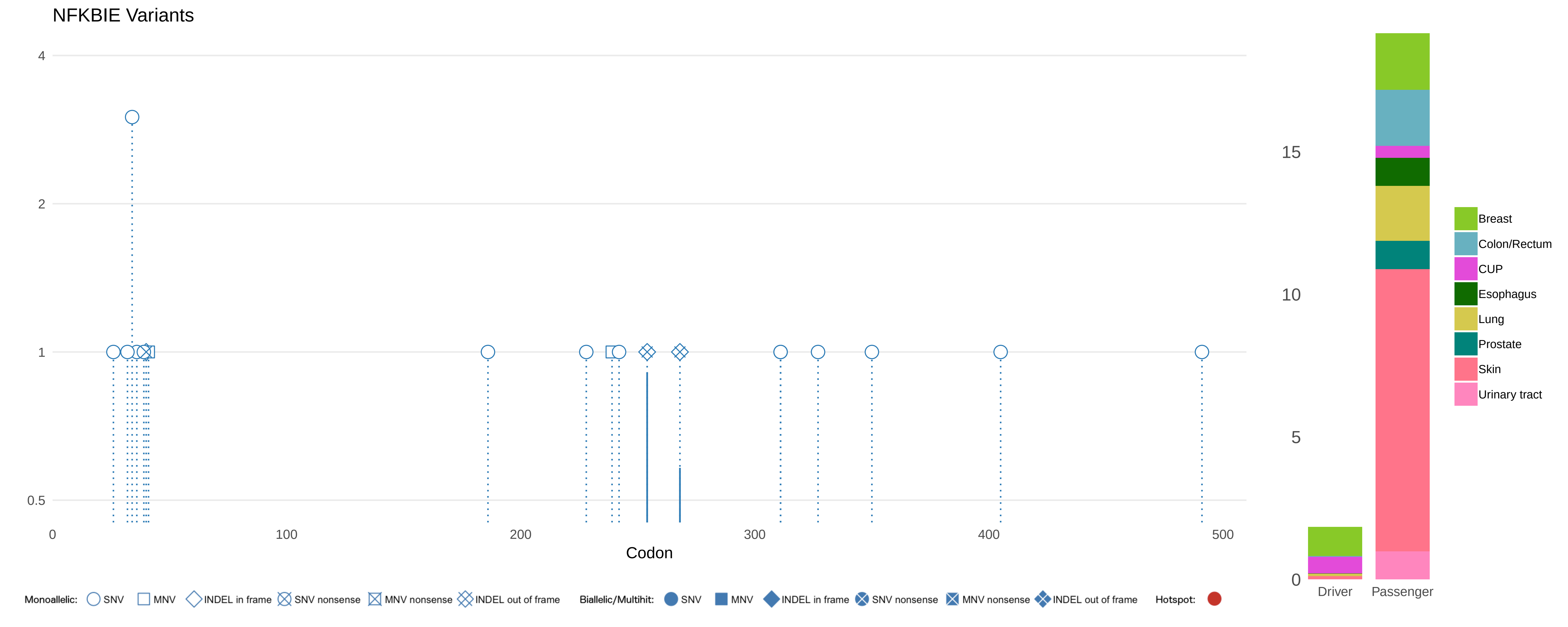
NFKBIE Variants
4
2
1
0.5
0
100
200
300
400
500
Codon
15
10
5
0
Driver
Passenger
Breast
Colon/Rectum
CUP
Esophagus
Lung
Prostate
Skin
Urinary tract
Monoallelic: SNV MNV INDEL in frame SNV nonsense MNV nonsense INDEL out of frame
Biallelic/Multihit: SNV MNV INDEL in frame SNV nonsense MNV nonsense INDEL out of frame
Hotspot:

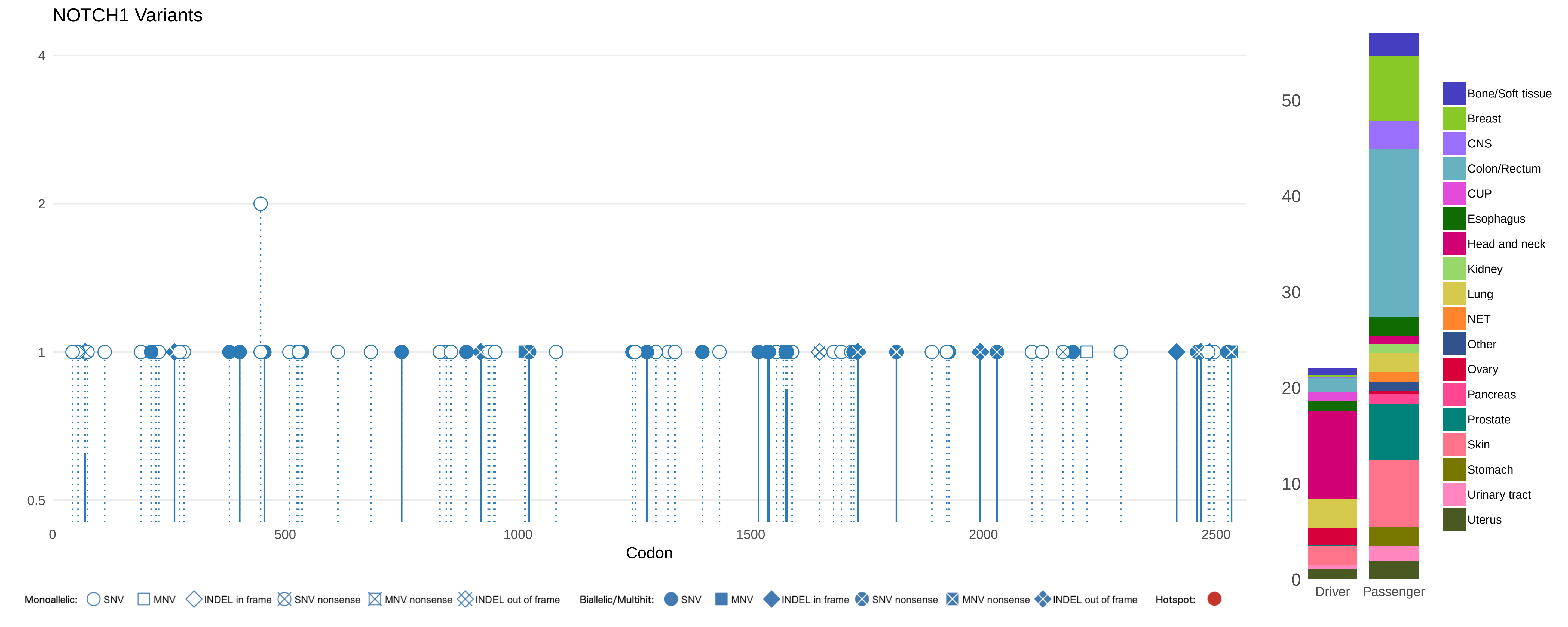

NOTCH1 Variants
4
2
1
0.5
0
500
1000
1500
2000
2500
Codon
50
40
30
20
10
0
Driver
Passenger
Bone/Soft tissue
Breast
CNS
Colon/Rectum
CUP
Esophagus
Head and neck
Kidney
Lung
NET
Other
Ovary
Pancreas
Prostate
Skin
Stomach
Urinary tract
Uterus
Monoallelic: SNV MNV INDEL in frame SNV nonsense MNV nonsense INDEL out of frame
Biallelic/Multihit: SNV MNV INDEL in frame SNV nonsense MNV nonsense INDEL out of frame
Hotspot:

NOTCH2 Variants

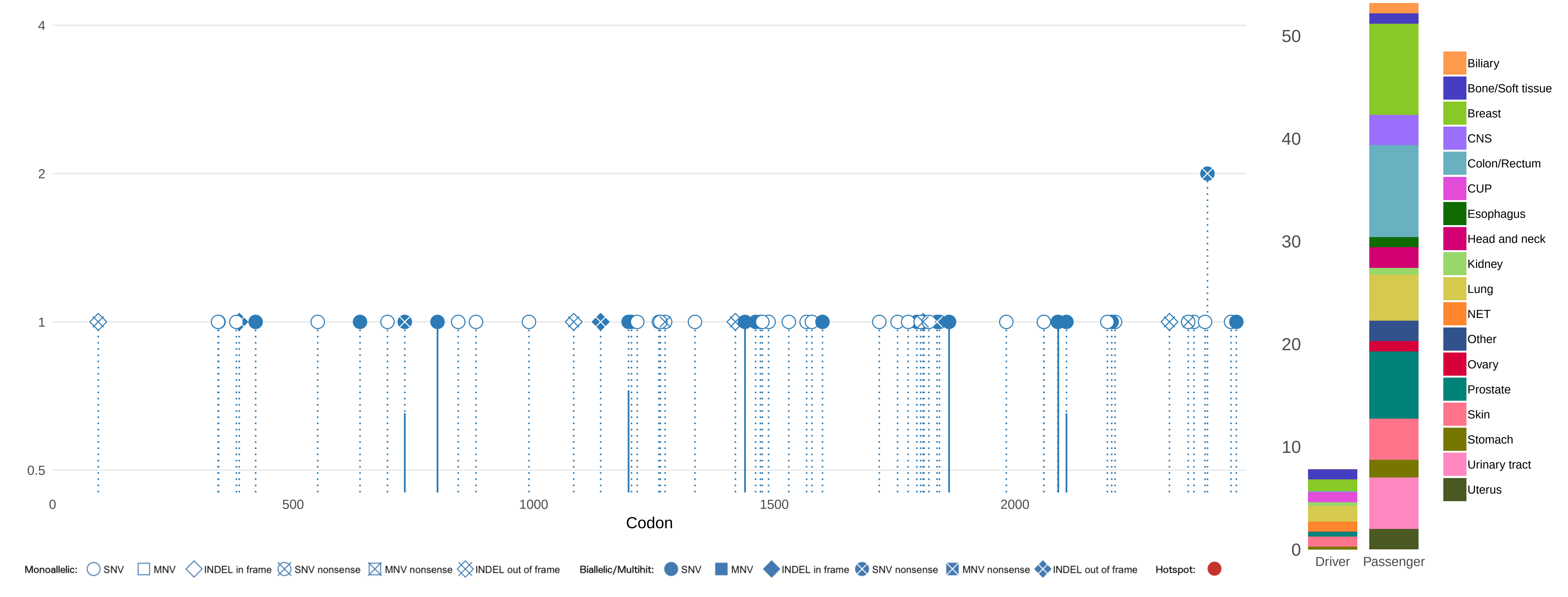

NSD1 Variants

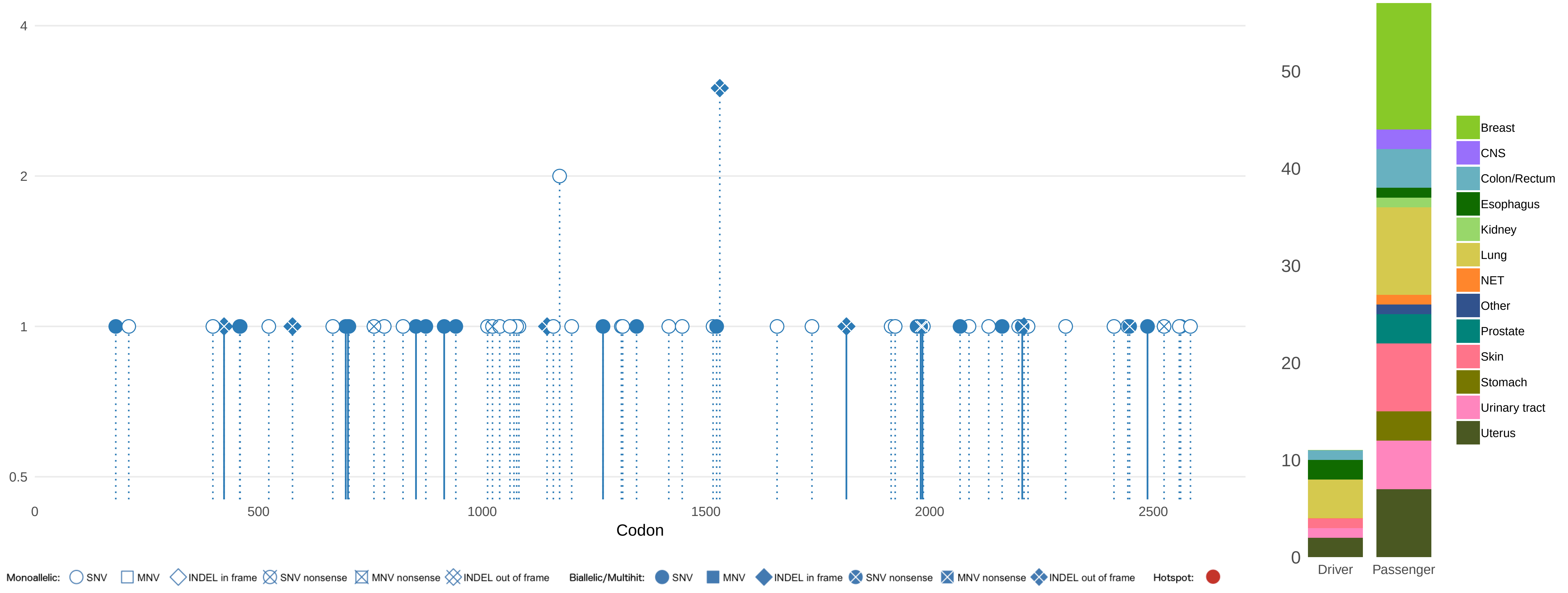

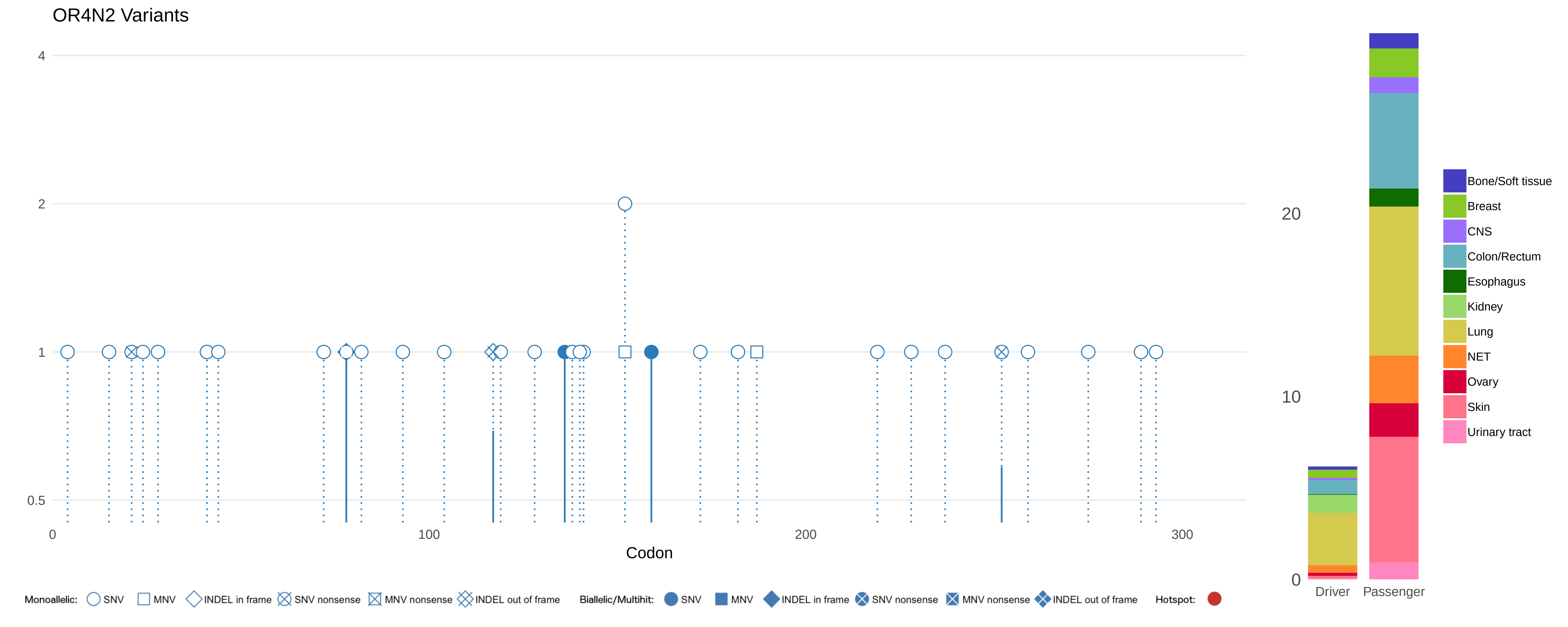
OR4N2 Variants
4
2
1
0.5
0
100
200
300
Codon
Monoallelic: SNV MNV INDEL in frame SNV nonsense MNV nonsense INDEL out of frame
Biallelic/Multihit: SNV MNV INDEL in frame SNV nonsense MNV nonsense INDEL out of frame
Hotspot:
20
10
0
Driver
Passenger
Bone/Soft tissue
Breast
CNS
Colon/Rectum
Esophagus
Kidney
Lung
NET
Ovary
Skin
Urinary tract

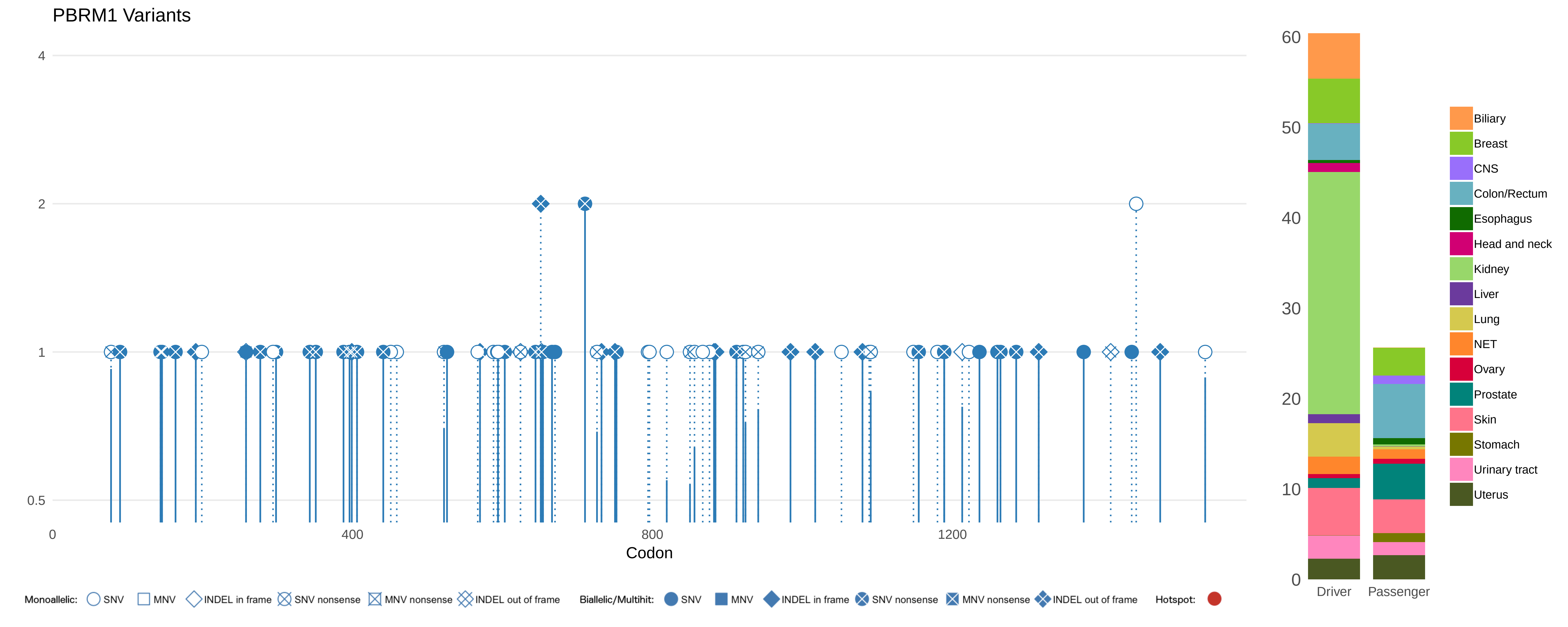

PBRM1 Variants
4
2
1
0.5
0
400
800
1200
Codon
60
50
40
30
20
10
0
Driver
Passenger
Biliary
Breast
CNS
Colon/Rectum
Esophagus
Head and neck
Kidney
Liver
Lung
NET
Ovary
Prostate
Skin
Stomach
Urinary tract
Uterus
Monoallelic: SNV MNV INDEL in frame SNV nonsense MNV nonsense INDEL out of frame
Biallelic/Multihit: SNV MNV INDEL in frame SNV nonsense MNV nonsense INDEL out of frame
Hotspot:

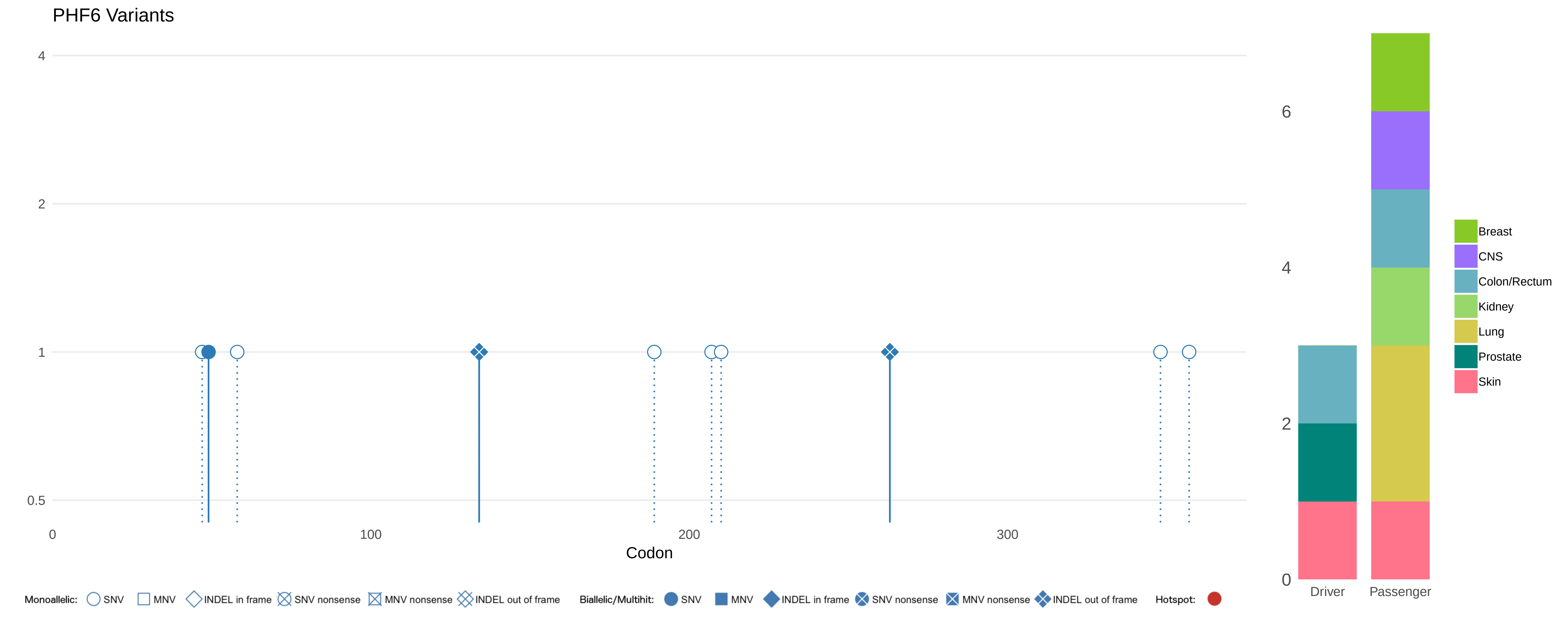
PHF6 Variants
4
2
1
0.5
0
100
200
300
Codon
6
4
2
0
Driver
Passenger
Breast
CNS
Colon/Rectum
Kidney
Lung
Prostate
Skin
Monoallelic: SNV MNV INDEL in frame SNV nonsense MNV nonsense INDEL out of frame
Biallelic/Multihit: SNV MNV INDEL in frame SNV nonsense MNV nonsense INDEL out of frame
Hotspot:

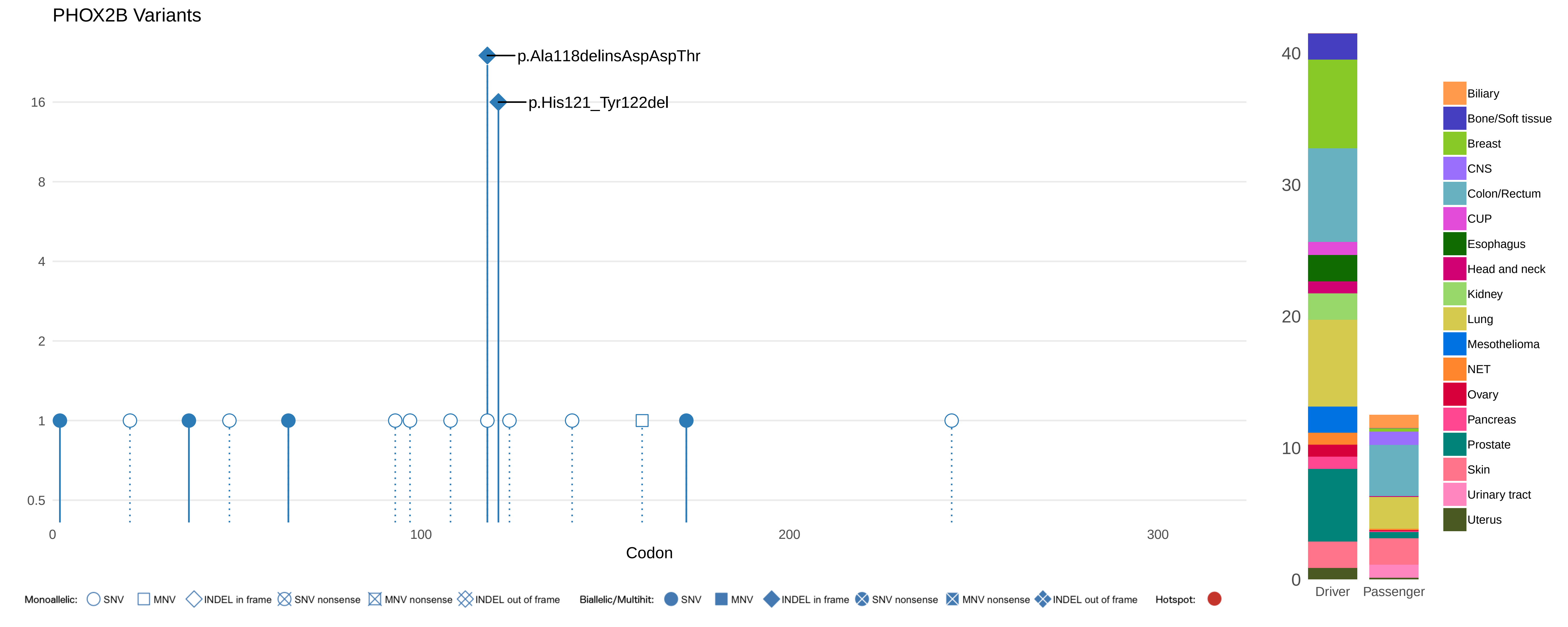
PHOX2B Variants
p.Ala118delinsAspAspThr
p.His121_Tyr122del
16
8
4
2
1
0.5
0
100
200
300
Codon
Monoallelic: SNV MNV INDEL in frame SNV nonsense MNV nonsense INDEL out of frame
Biallelic/Multihit: SNV MNV INDEL in frame SNV nonsense MNV nonsense INDEL out of frame
Hotspot:
40
30
20
10
0
Driver
Passenger
Biliary
Bone/Soft tissue
Breast
CNS
Colon/Rectum
CUP
Esophagus
Head and neck
Kidney
Lung
Mesothelioma
NET
Ovary
Pancreas
Prostate
Skin
Urinary tract
Uterus

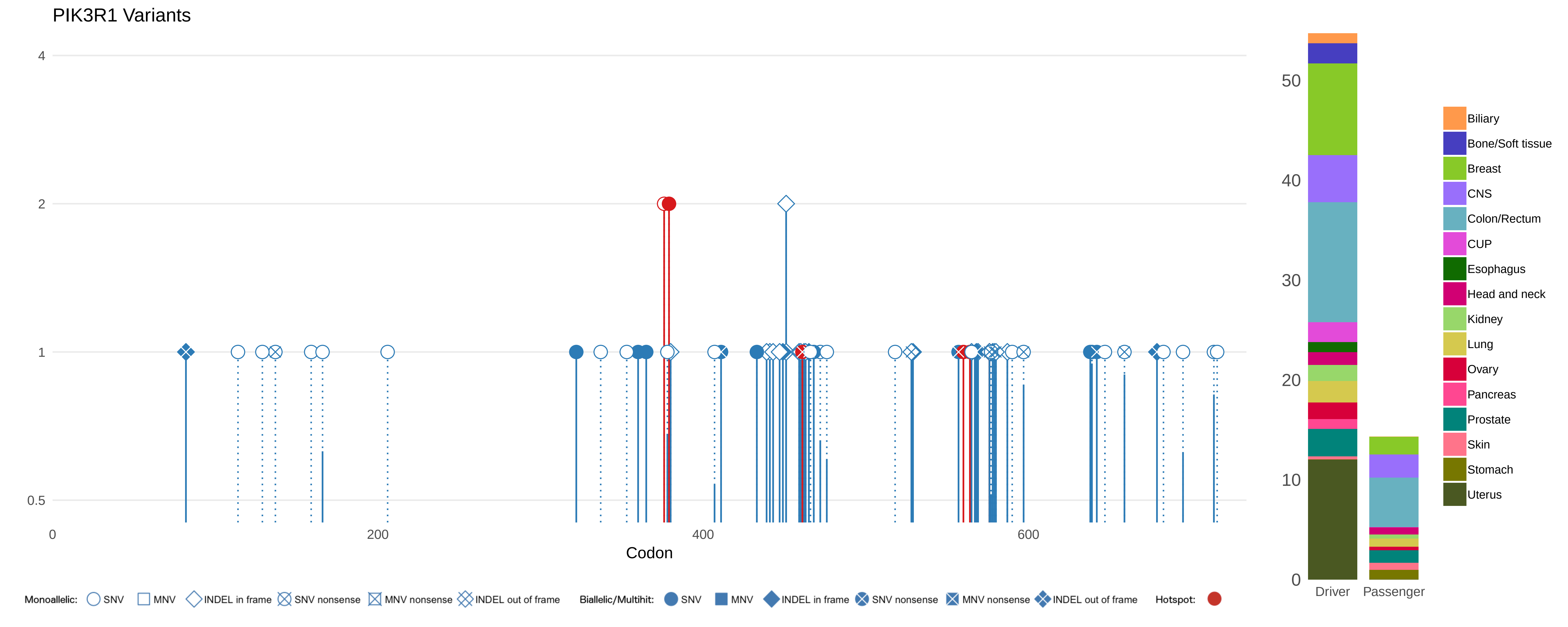
PIK3R1 Variants
4
2
1
0.5
0
200
400
600
Codon
Monoallelic: SNV MNV INDEL in frame SNV nonsense MNV nonsense INDEL out of frame
Biallelic/Multihit: SNV MNV INDEL in frame SNV nonsense MNV nonsense INDEL out of frame
Hotspot:
50
40
30
20
10
0
Driver
Passenger
Biliary
Bone/Soft tissue
Breast
CNS
Colon/Rectum
CUP
Esophagus
Head and neck
Kidney
Lung
Ovary
Pancreas
Prostate
Skin
Stomach
Uterus

POLE Variants

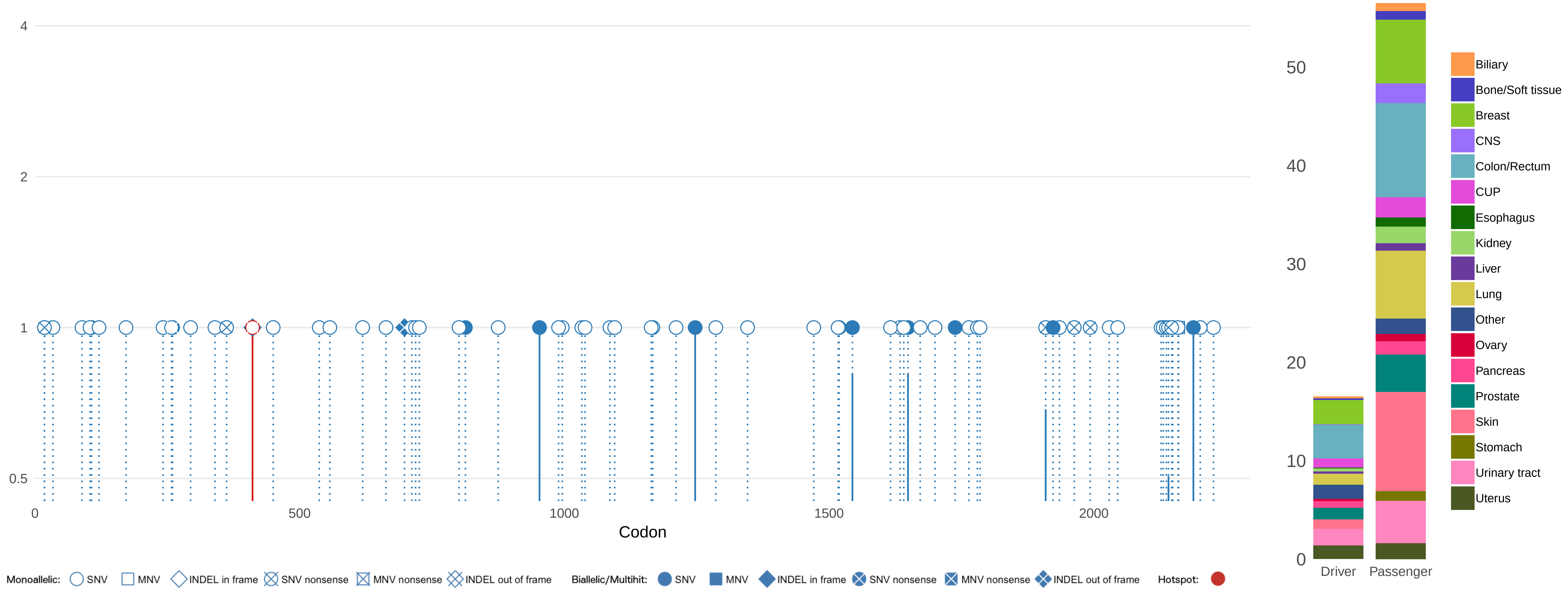

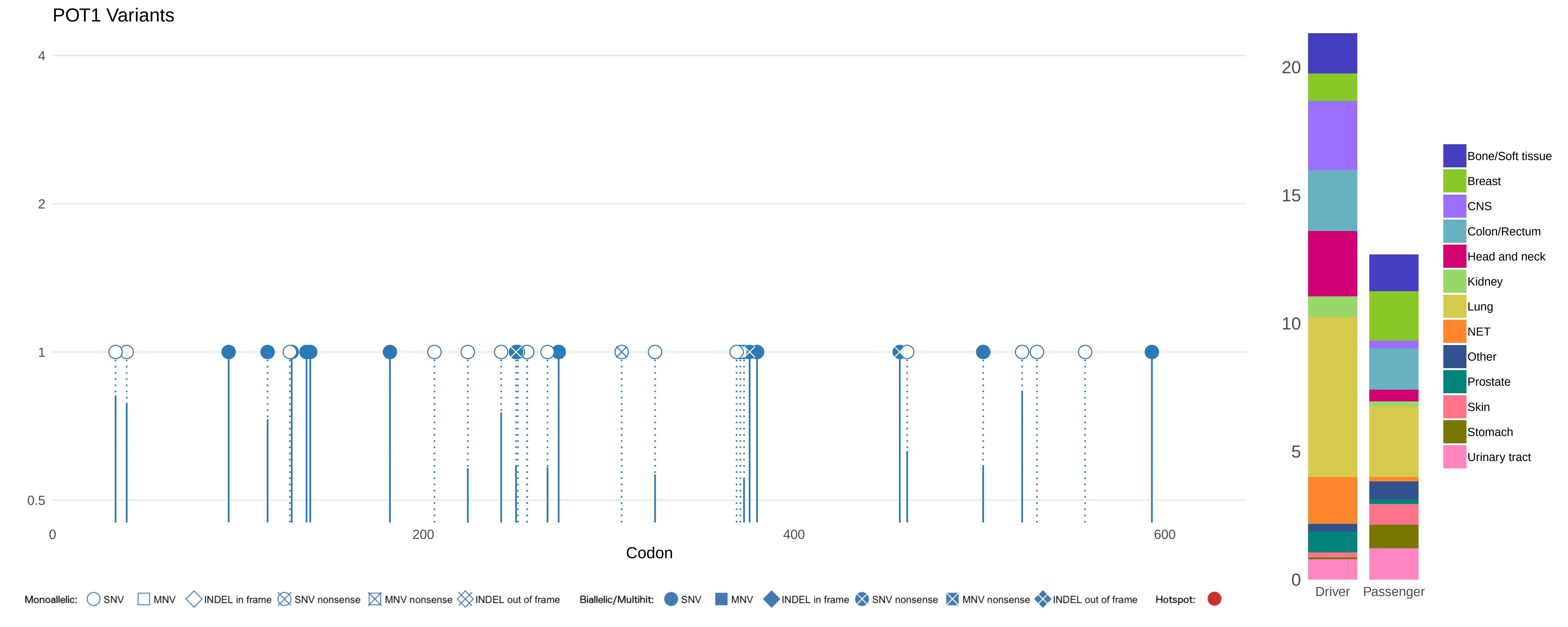

POT1 Variants
4
2
1
0.5
0
200
400
600
Codon
Monoallelic: SNV MNV INDEL in frame SNV nonsense MNV nonsense INDEL out of frame
Biallelic/Multihit: SNV MNV INDEL in frame SNV nonsense MNV nonsense INDEL out of frame
Hotspot:
20
15
10
5
0
Driver
Passenger
Bone/Soft tissue
Breast
CNS
Colon/Rectum
Head and neck
Kidney
Lung
NET
Other
Prostate
Skin
Stomach
Urinary tract

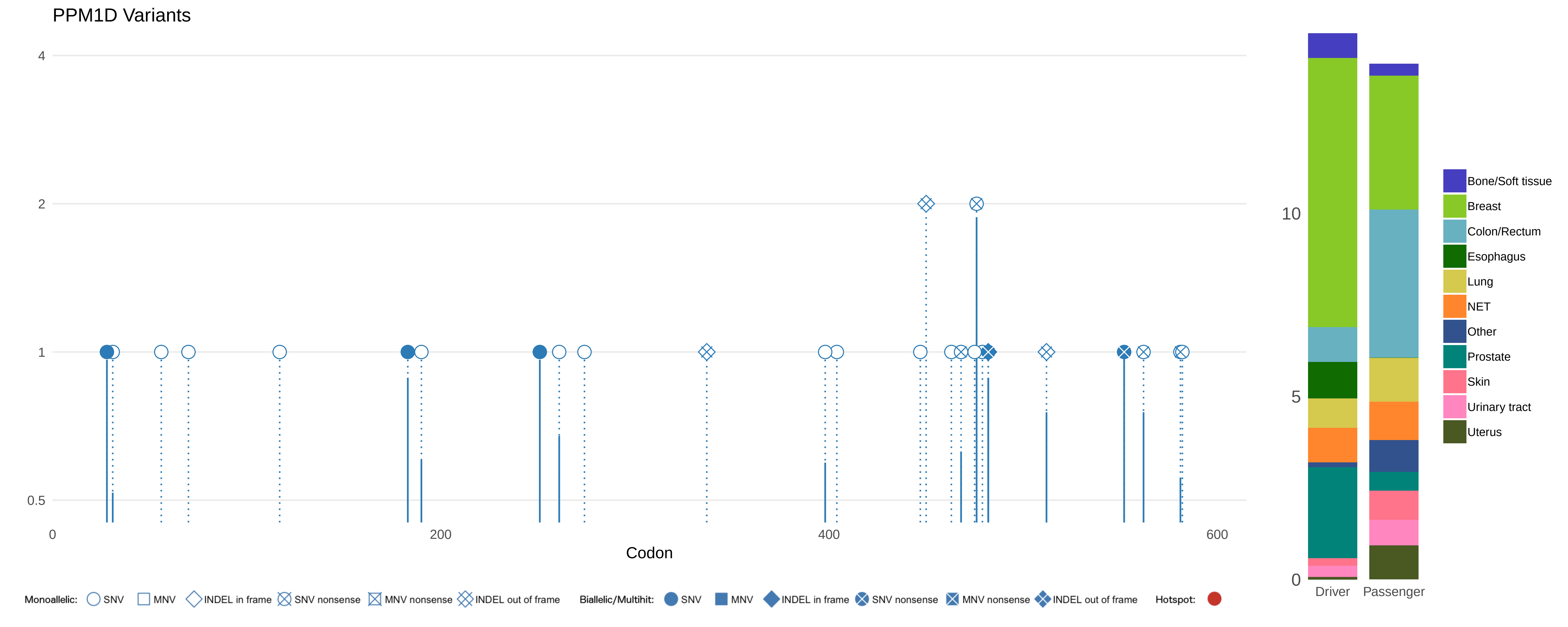

PPM1D Variants
4
2
1
0.5
0
200
400
600
Codon
10
5
0
Driver
Passenger
Bone/Soft tissue
Breast
Colon/Rectum
Esophagus
Lung
NET
Other
Prostate
Skin
Urinary tract
Uterus
Monoallelic: SNV MNV INDEL in frame SNV nonsense MNV nonsense INDEL out of frame
Biallelic/Multihit: SNV MNV INDEL in frame SNV nonsense MNV nonsense INDEL out of frame
Hotspot:

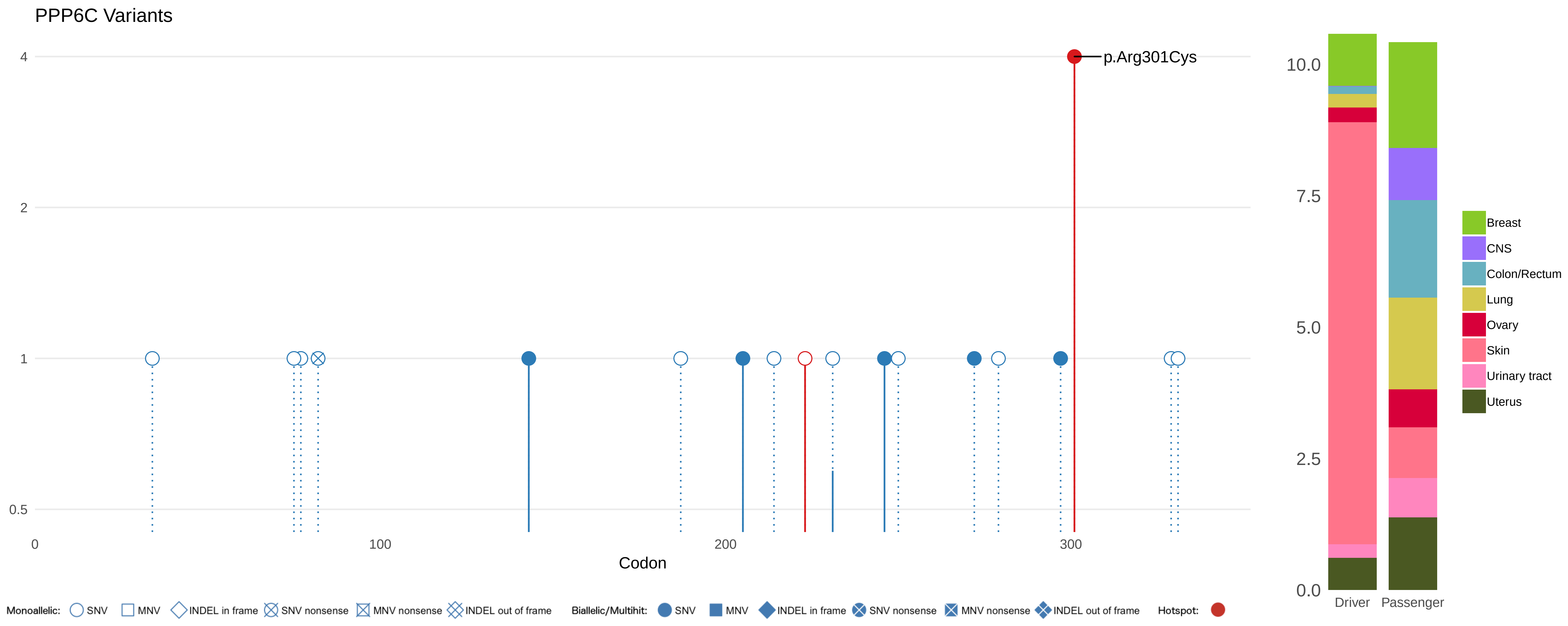
PPP6C Variants
p.Arg301Cys
4
2
1
0.5
0
100
200
300
Codon
10.0
7.5
5.0
2.5
0.0
Driver
Passenger
Breast
CNS
Colon/Rectum
Lung
Ovary
Skin
Urinary tract
Uterus
Monoallelic:
SNV
MNV
INDEL in frame
SNV nonsense
MNV nonsense
INDEL out of frame
Biallelic/Multihit:
SNV
MNV
INDEL in frame
SNV nonsense
MNV nonsense
INDEL out of frame
Hotspot:

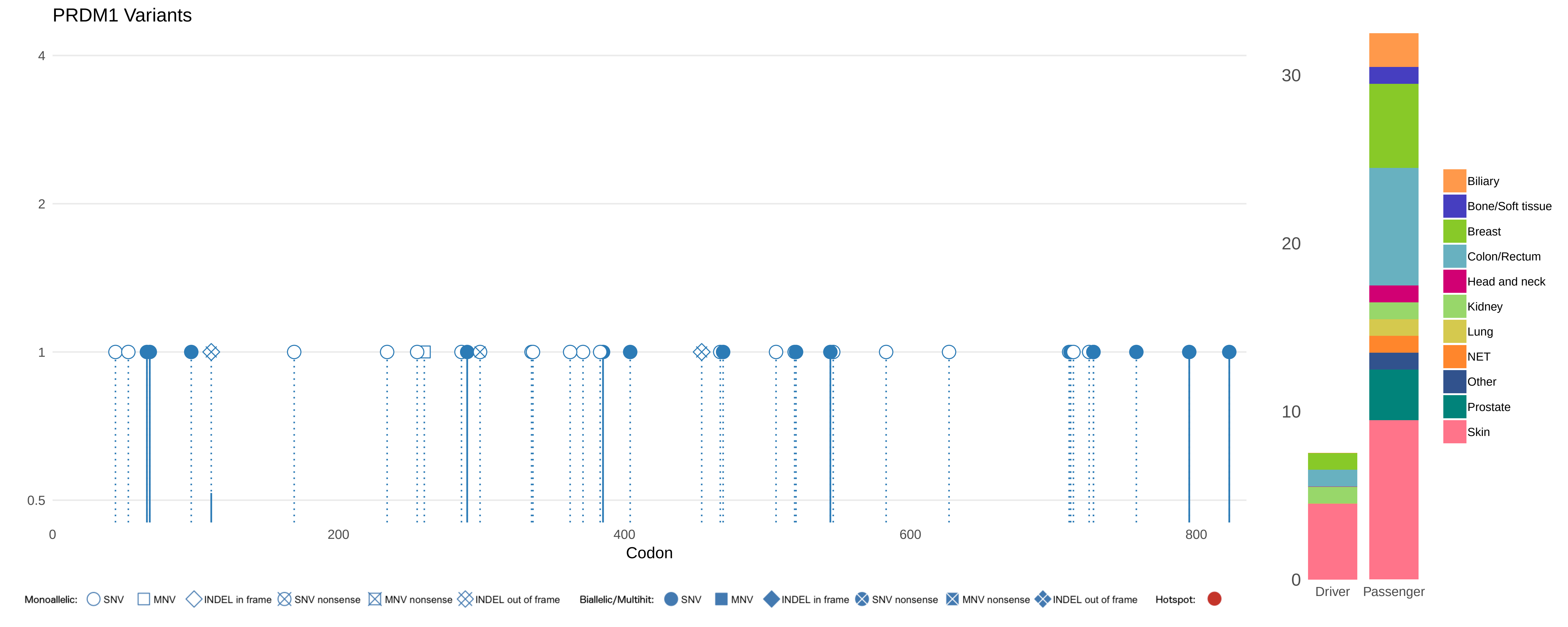
PRDM1 Variants
4
2
1
0.5
0
200
400
600
800
Codon
30
20
10
0
Driver
Passenger
Biliary
Bone/Soft tissue
Breast
Colon/Rectum
Head and neck
Kidney
Lung
NET
Other
Prostate
Skin
Monoallelic: SNV MNV INDEL in frame SNV nonsense MNV nonsense INDEL out of frame
Biallelic/Multihit: SNV MNV INDEL in frame SNV nonsense MNV nonsense INDEL out of frame
Hotspot:

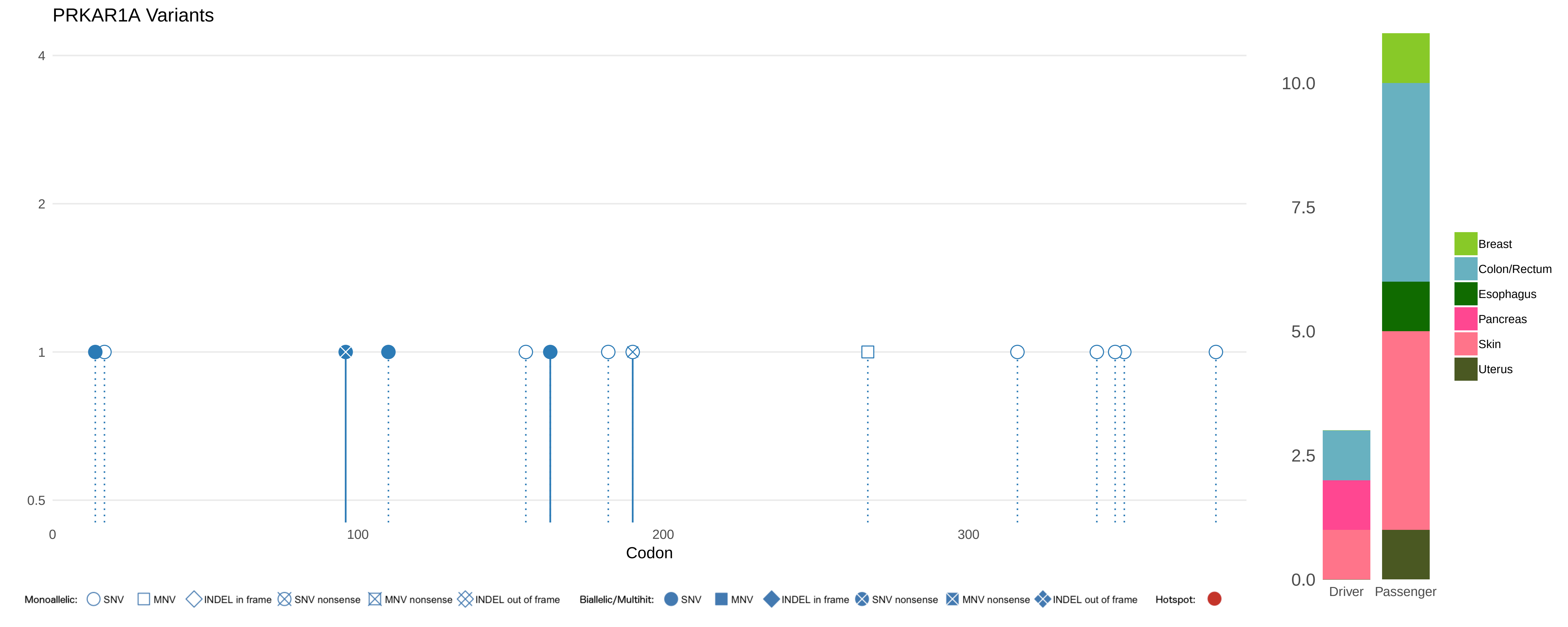
PRKAR1A Variants
4
2
1
0.5
0
100
200
300
Codon
10.0
7.5
5.0
2.5
0.0
Driver
Passenger
Breast
Colon/Rectum
Esophagus
Pancreas
Skin
Uterus
Monoallelic: SNV MNV INDEL in frame SNV nonsense MNV nonsense INDEL out of frame
Biallelic/Multihit: SNV MNV INDEL in frame SNV nonsense MNV nonsense INDEL out of frame
Hotspot:

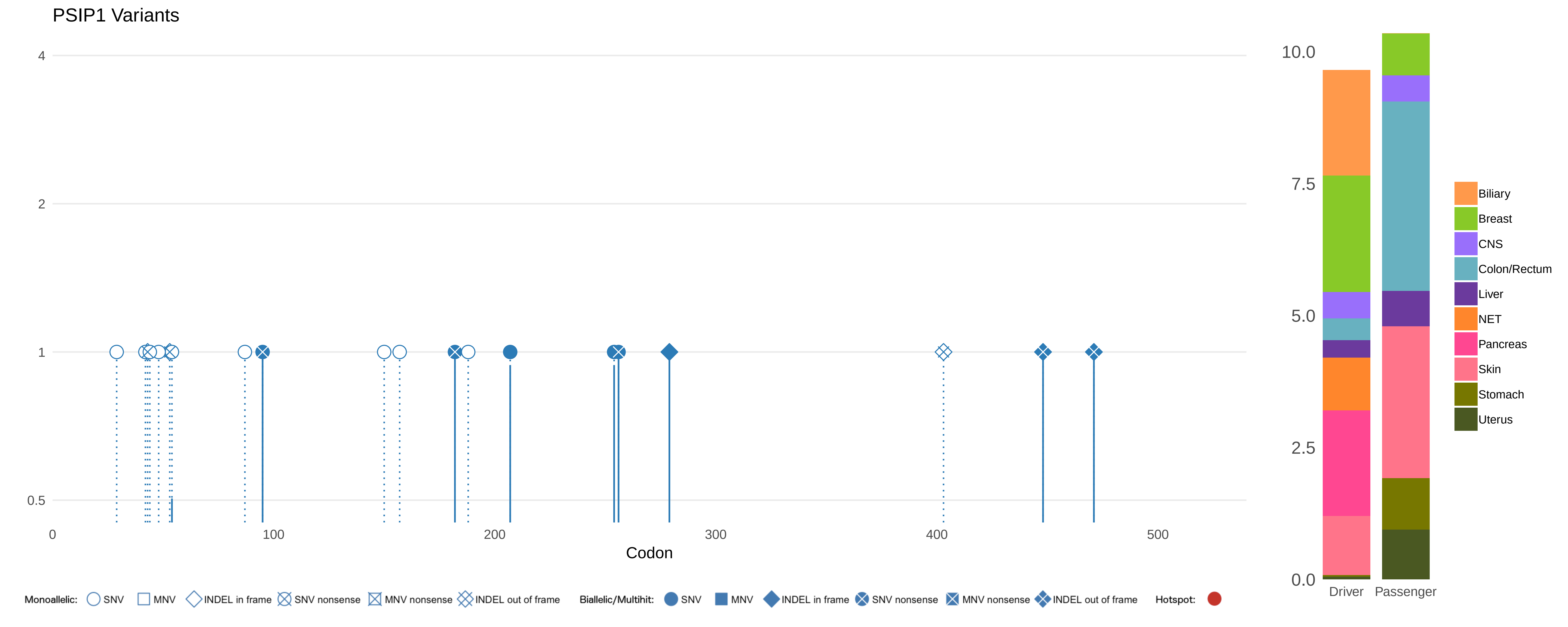

PSIP1 Variants
4
2
1
0.5
0
100
200
300
400
500
Codon
10.0
7.5
5.0
2.5
0.0
Driver
Passenger
Biliary
Breast
CNS
Colon/Rectum
Liver
NET
Pancreas
Skin
Stomach
Uterus
Monoallelic: SNV MNV INDEL in frame SNV nonsense MNV nonsense INDEL out of frame
Biallelic/Multihit: SNV MNV INDEL in frame SNV nonsense MNV nonsense INDEL out of frame
Hotspot:

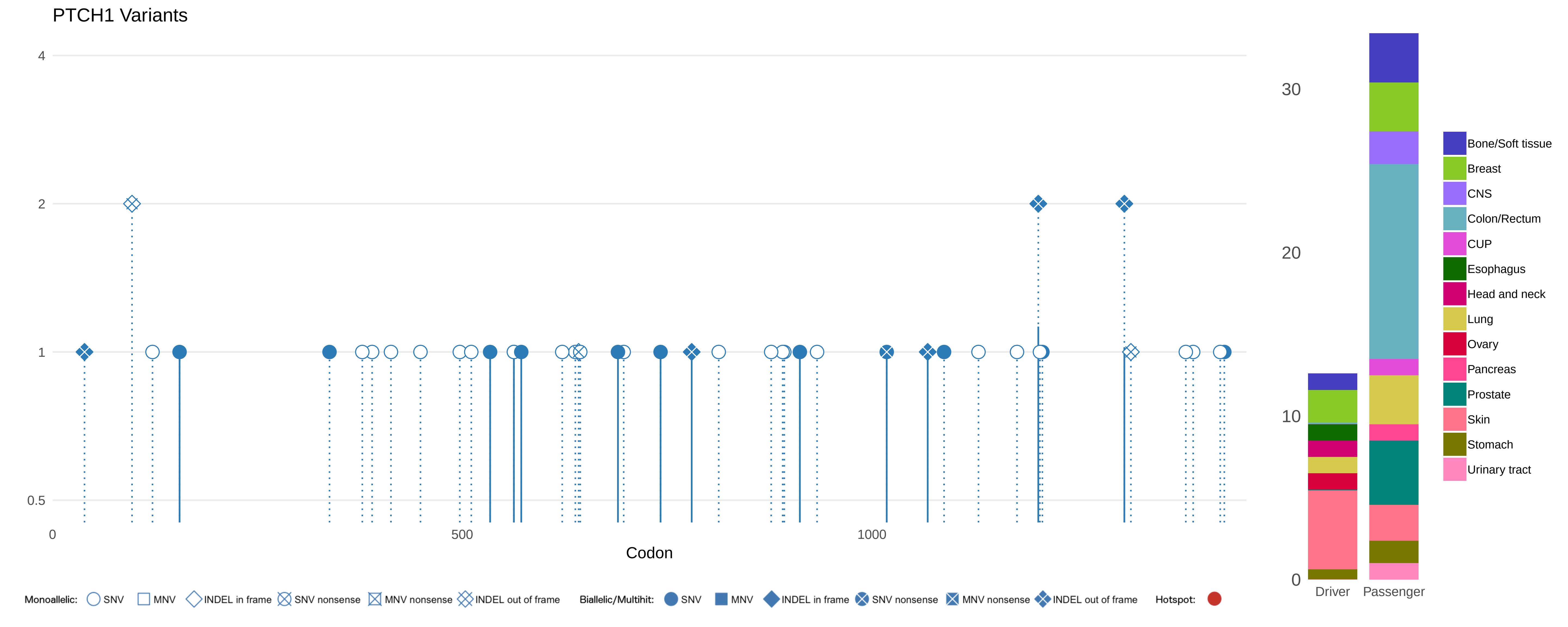
PTCH1 Variants
4
2
1
0.5
0
500
1000
Codon
Monoallelic: SNV MNV INDEL in frame SNV nonsense MNV nonsense INDEL out of frame
Biallelic/Multihit: SNV MNV INDEL in frame SNV nonsense MNV nonsense INDEL out of frame
Hotspot:
30
20
10
0
Driver
Passenger
Bone/Soft tissue
Breast
CNS
Colon/Rectum
CUP
Esophagus
Head and neck
Lung
Ovary
Pancreas
Prostate
Skin
Stomach
Urinary tract

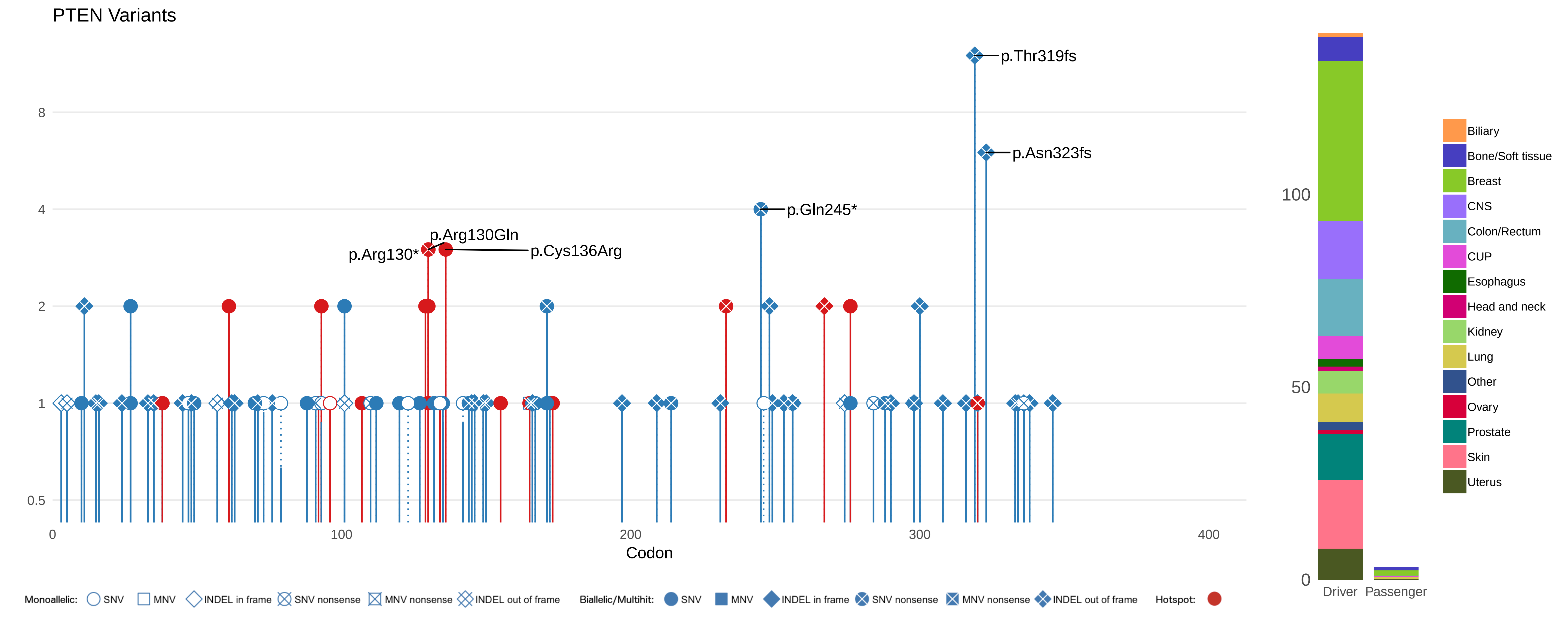

PTEN Variants
p.Thr319fs
p.Asn323fs
p.Gln245*
p.Arg130Gln
p.Arg130*
p.Cys136Arg
8
4
2
1
0.5
0
100
200
300
400
Codon
100
50
0
Driver
Passenger
Biliary
Bone/Soft tissue
Breast
CNS
Colon/Rectum
CUP
Esophagus
Head and neck
Kidney
Lung
Other
Ovary
Prostate
Skin
Uterus
Monoallelic: SNV MNV INDEL in frame SNV nonsense MNV nonsense INDEL out of frame
Biallelic/Multihit: SNV MNV INDEL in frame SNV nonsense MNV nonsense INDEL out of frame
Hotspot:

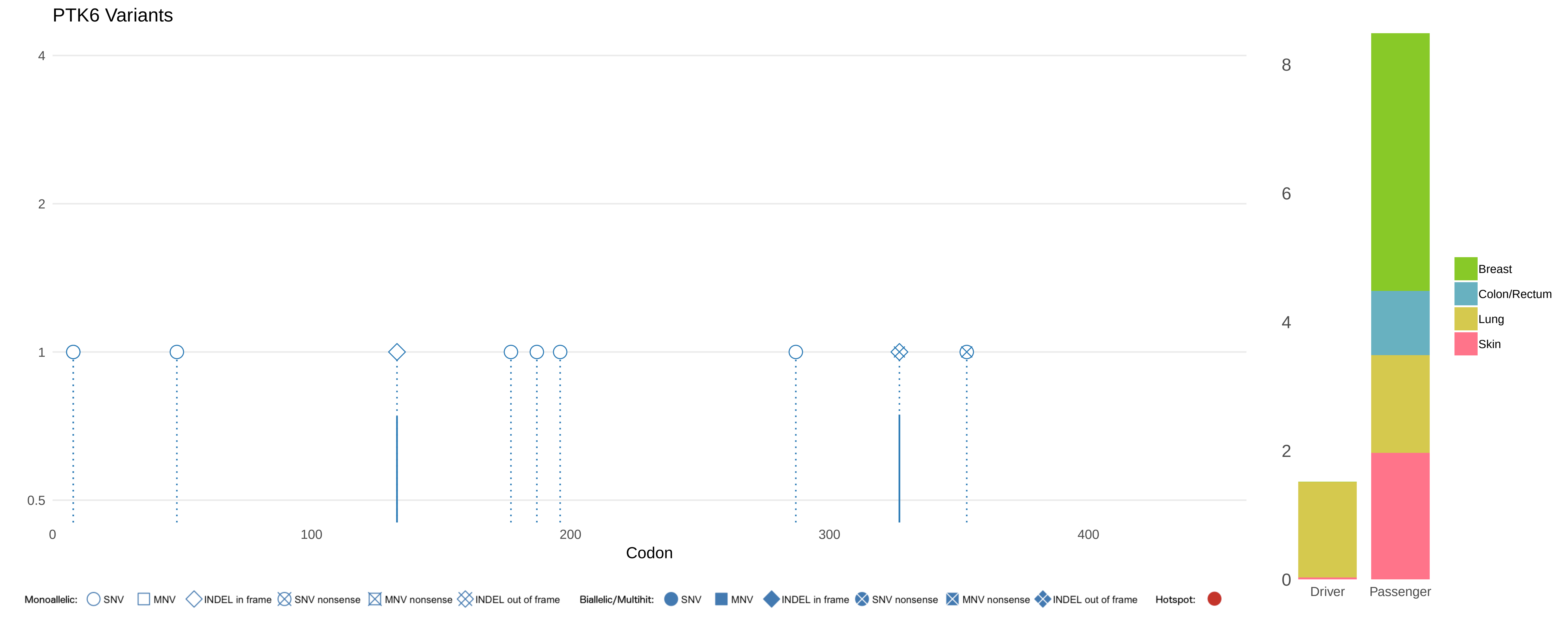
PTK6 Variants
4
2
1
0.5
0
100
200
300
400
Codon
8
6
4
2
0
Driver
Passenger
Breast
Colon/Rectum
Lung
Skin
Monoallelic: SNV MNV INDEL in frame SNV nonsense MNV nonsense INDEL out of frame
Biallelic/Multihit: SNV MNV INDEL in frame SNV nonsense MNV nonsense INDEL out of frame
Hotspot:

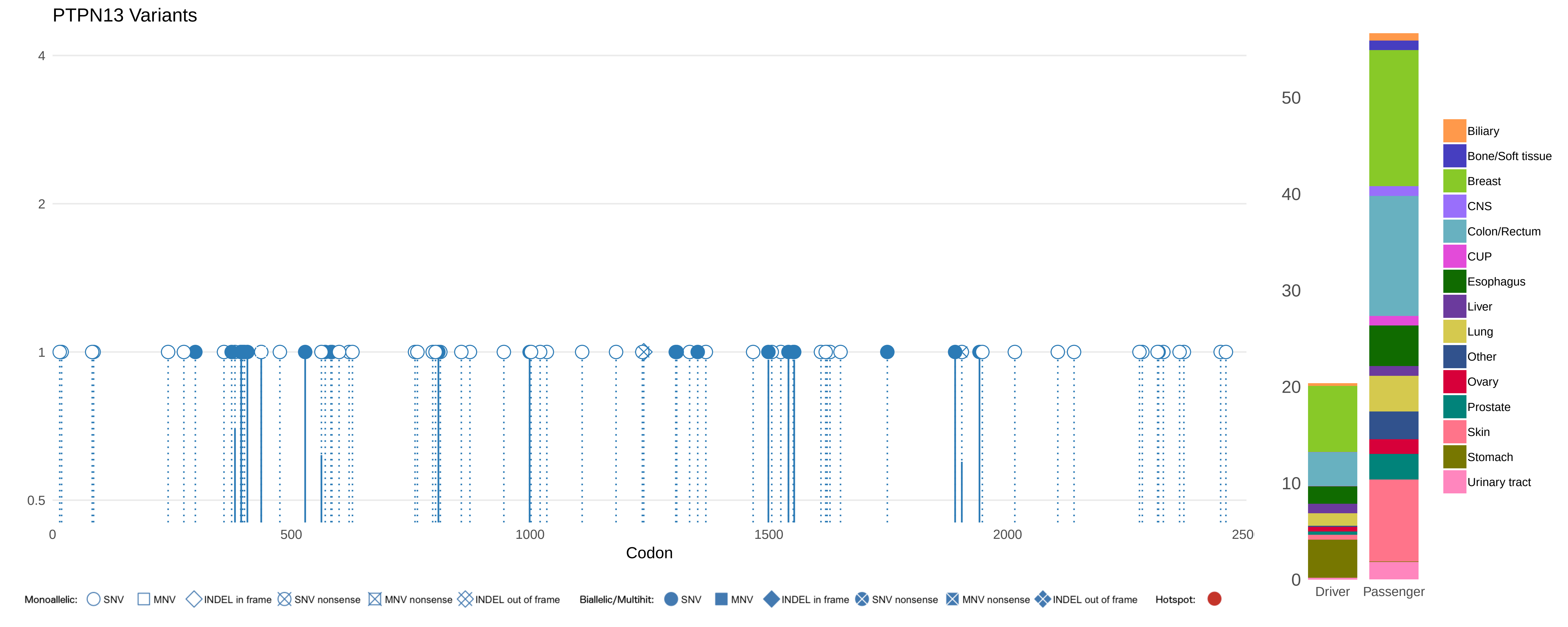
PTPN13 Variants
4
2
1
0.5
0
500
1000
1500
2000
250
Codon
50
40
30
20
10
0
Driver
Passenger
Biliary
Bone/Soft tissue
Breast
CNS
Colon/Rectum
CUP
Esophagus
Liver
Lung
Other
Ovary
Prostate
Skin
Stomach
Urinary tract
Monoallelic: SNV MNV INDEL in frame SNV nonsense MNV nonsense INDEL out of frame
Biallelic/Multihit: SNV MNV INDEL in frame SNV nonsense MNV nonsense INDEL out of frame
Hotspot:

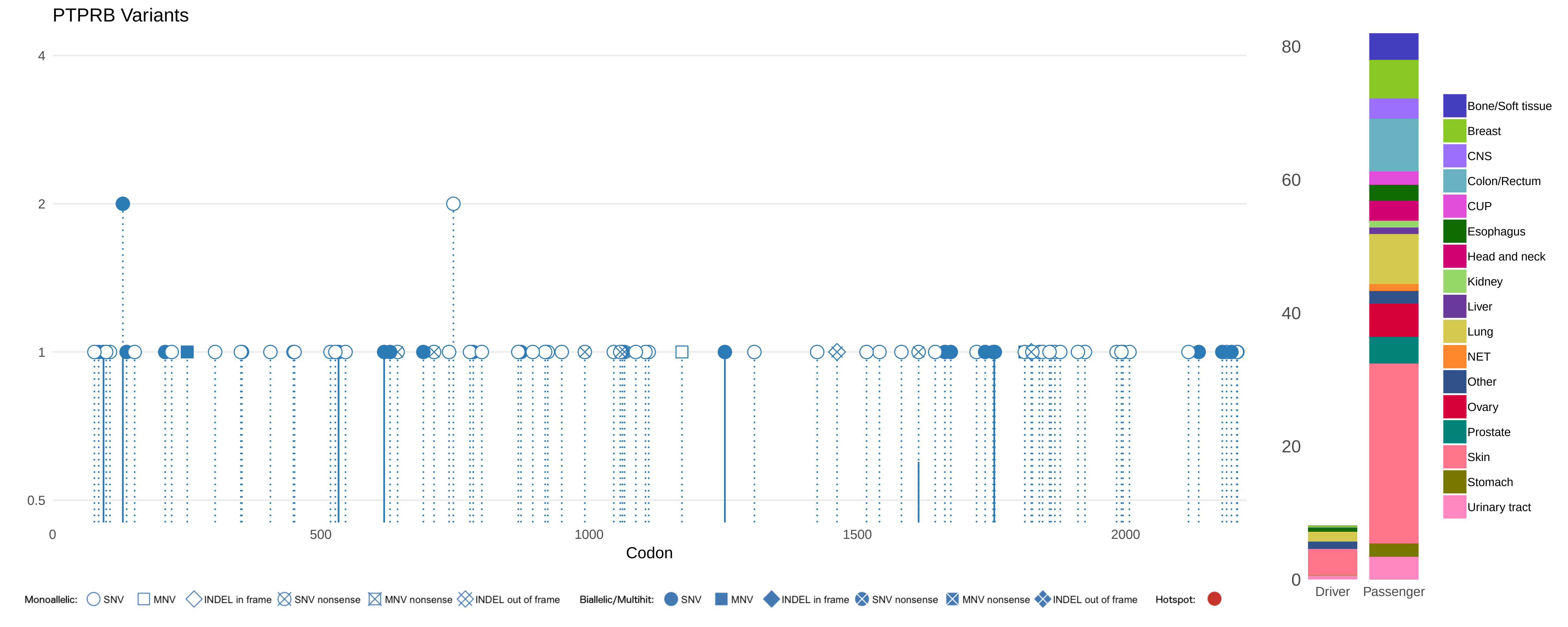
PTPRB Variants
4
2
1
0.5
0
500
1000
1500
2000
Codon
Monoallelic: SNV MNV INDEL in frame SNV nonsense MNV nonsense INDEL out of frame
Biallelic/Multihit: SNV MNV INDEL in frame SNV nonsense MNV nonsense INDEL out of frame
Hotspot:
80
60
40
20
0
Driver
Passenger
Bone/Soft tissue
Breast
CNS
Colon/Rectum
CUP
Esophagus
Head and neck
Kidney
Liver
Lung
NET
Other
Ovary
Prostate
Skin
Stomach
Urinary tract

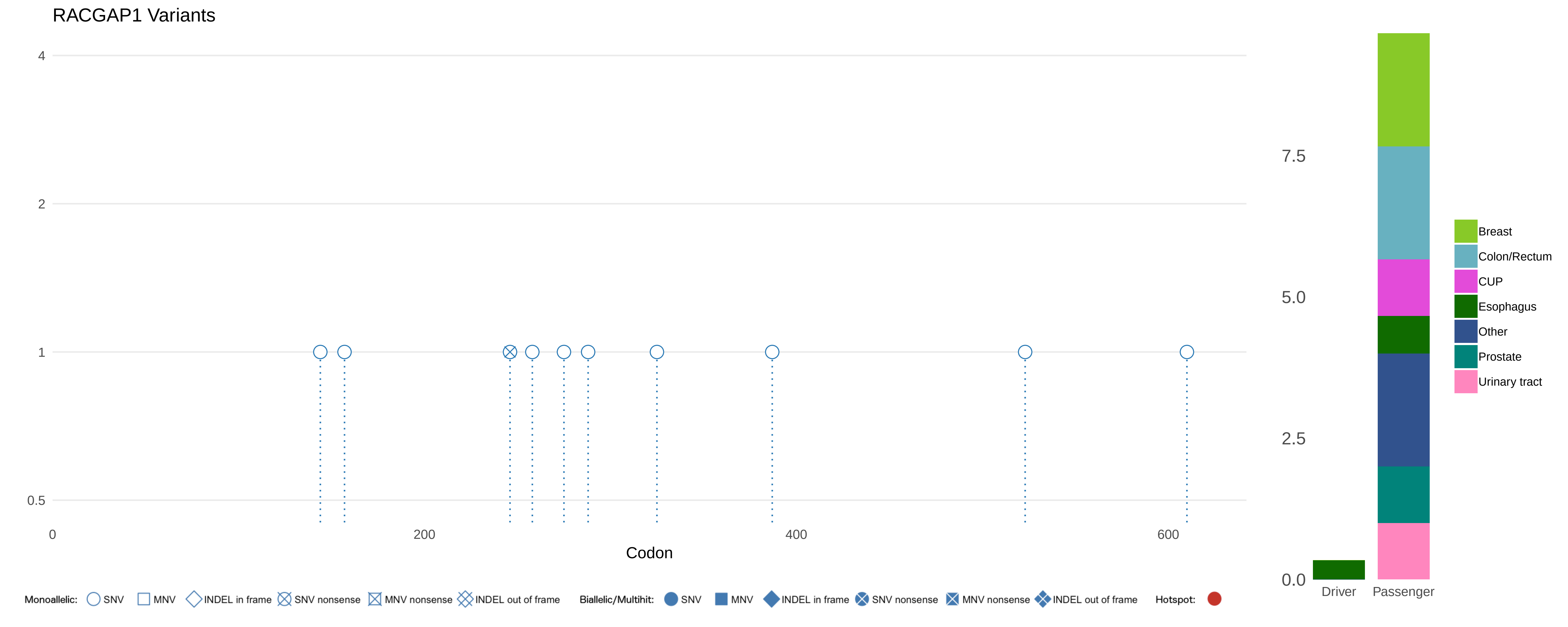
RACGAP1 Variants
4
2
1
0.5
0
200
400
600
Codon
Monoallelic: SNV MNV INDEL in frame SNV nonsense MNV nonsense INDEL out of frame
Biallelic/Multihit: SNV MNV INDEL in frame SNV nonsense MNV nonsense INDEL out of frame
Hotspot:
7.5
5.0
2.5
0.0
Driver
Passenger
Breast
Colon/Rectum
CUP
Esophagus
Other
Prostate
Urinary tract

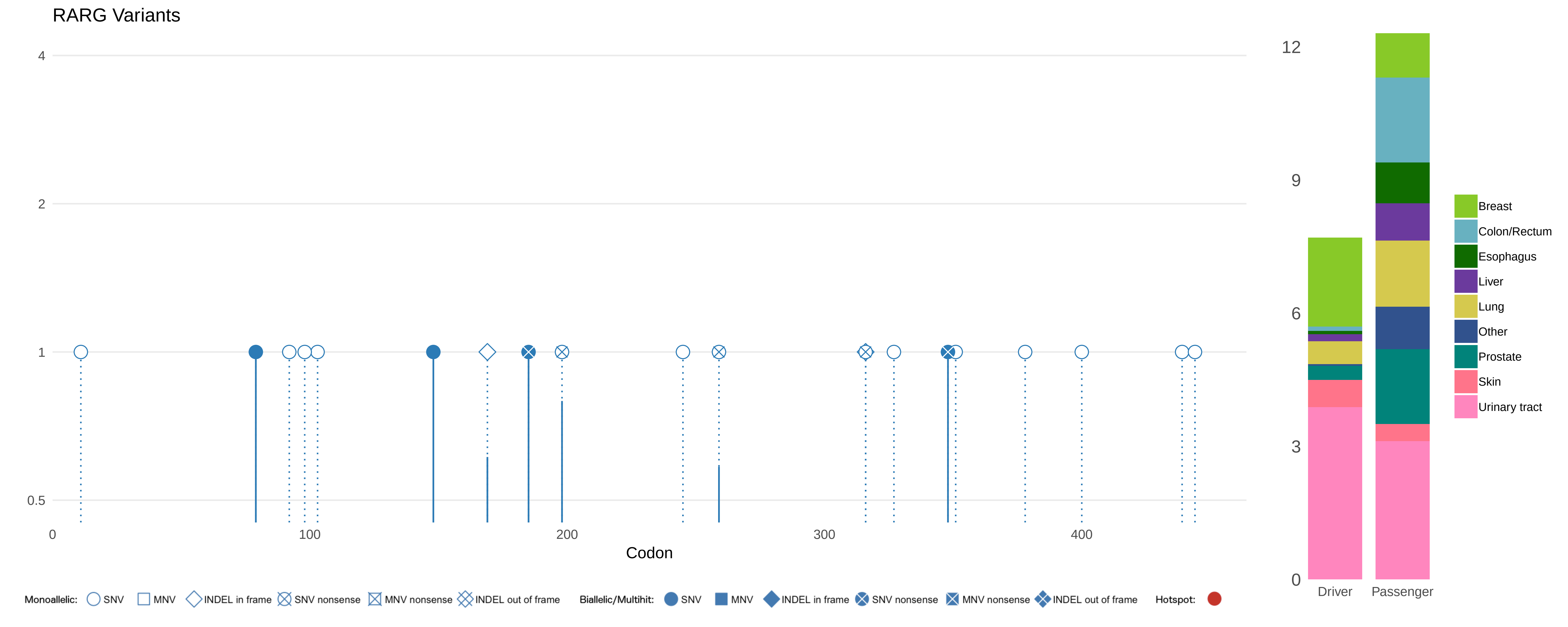
RARG Variants
4
2
1
0.5
0
100
200
300
400
Codon
12
9
6
3
0
Driver
Passenger
Breast
Colon/Rectum
Esophagus
Liver
Lung
Other
Prostate
Skin
Urinary tract
Monoallelic: SNV MNV INDEL in frame SNV nonsense MNV nonsense INDEL out of frame
Biallelic/Multihit: SNV MNV INDEL in frame SNV nonsense MNV nonsense INDEL out of frame
Hotspot:

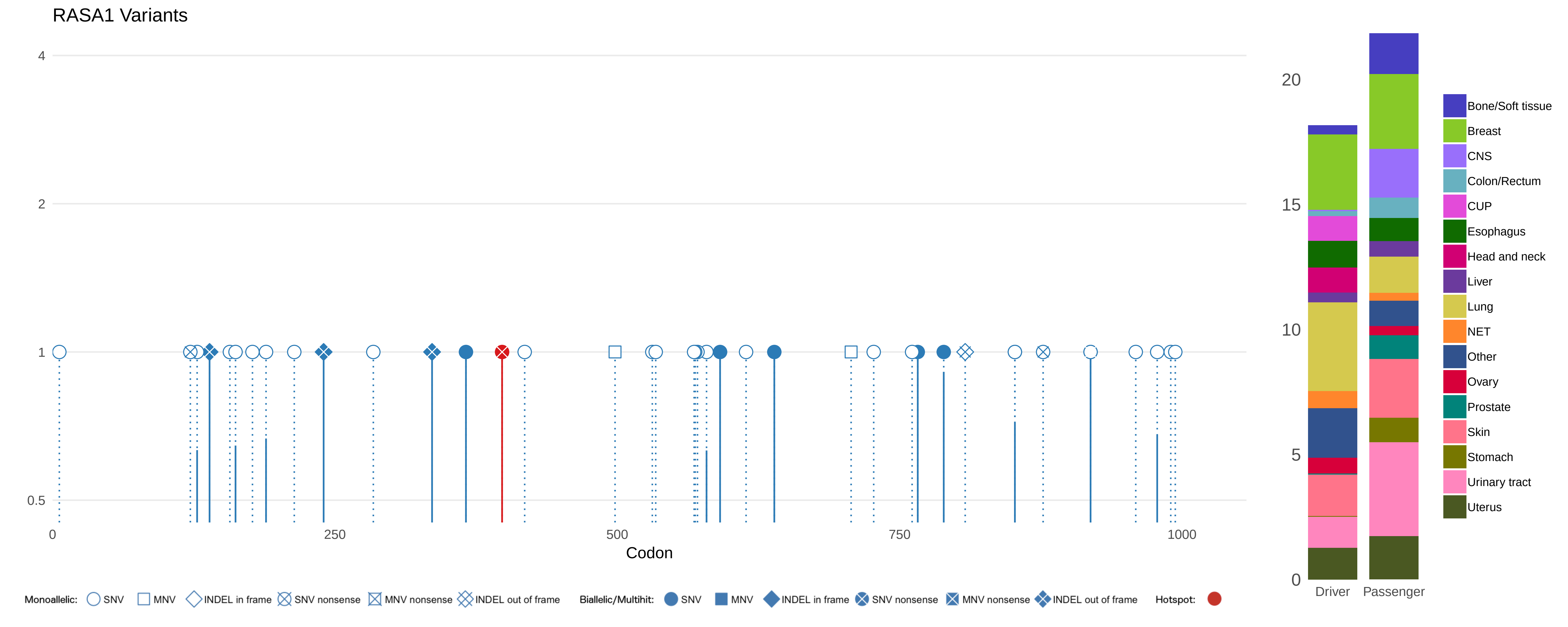

RASA1 Variants
4
2
1
0.5
0
250
500
750
1000
Codon
20
15
10
5
0
Driver
Passenger
Bone/Soft tissue
Breast
CNS
Colon/Rectum
CUP
Esophagus
Head and neck
Liver
Lung
NET
Other
Ovary
Prostate
Skin
Stomach
Urinary tract
Uterus
Monoallelic: SNV MNV INDEL in frame SNV nonsense MNV nonsense INDEL out of frame
Biallelic/Multihit: SNV MNV INDEL in frame SNV nonsense MNV nonsense INDEL out of frame
Hotspot:

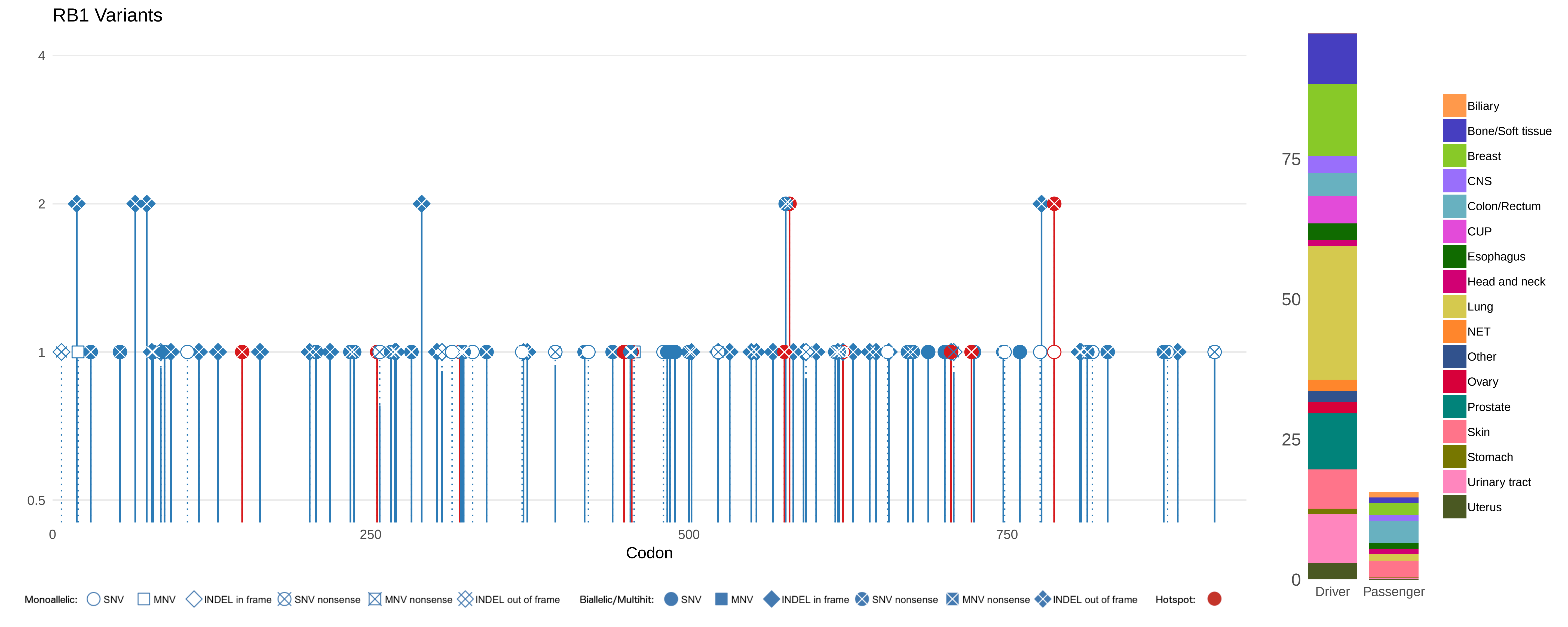

RB1 Variants
4
2
1
0.5
0
250
500
750
Codon
Monoallelic: SNV MNV INDEL in frame SNV nonsense MNV nonsense INDEL out of frame
Biallelic/Multihit: SNV MNV INDEL in frame SNV nonsense MNV nonsense INDEL out of frame
Hotspot:
75
50
25
0
Driver
Passenger
Biliary
Bone/Soft tissue
Breast
CNS
Colon/Rectum
CUP
Esophagus
Head and neck
Lung
NET
Other
Ovary
Prostate
Skin
Stomach
Urinary tract
Uterus

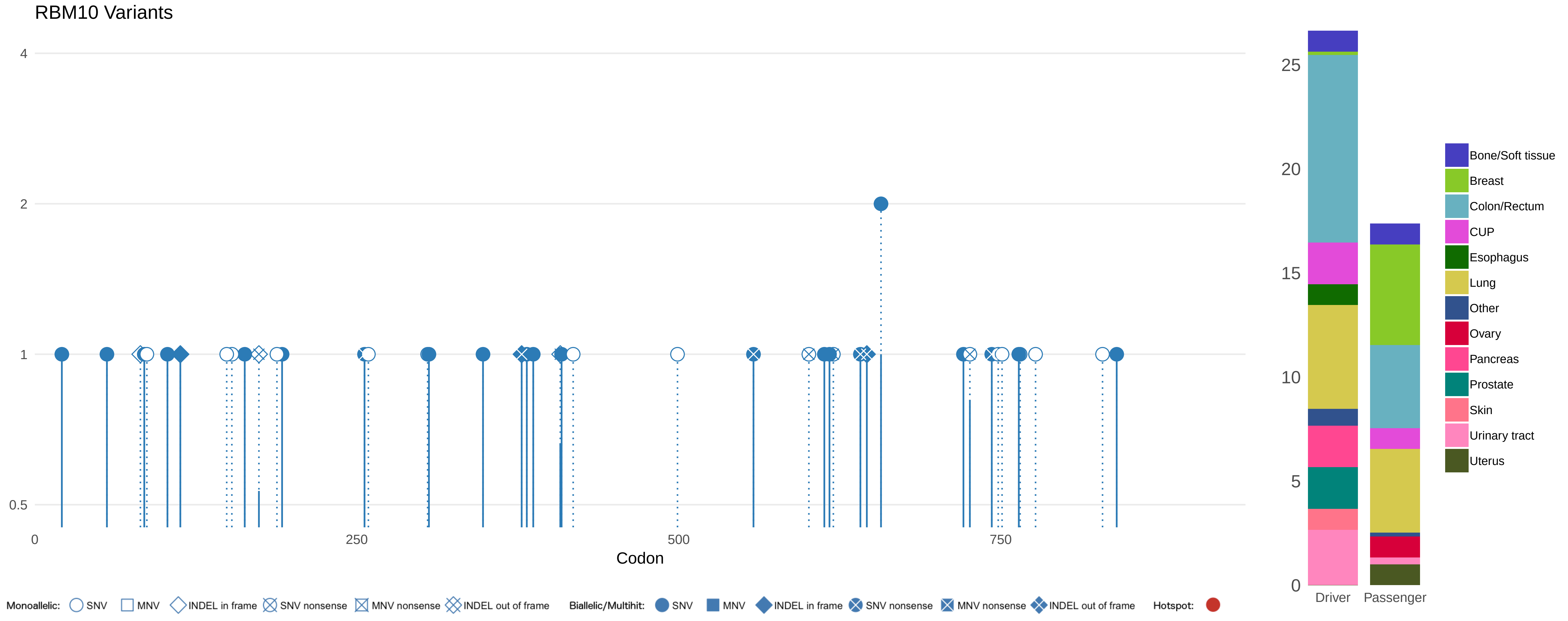

RBM10 Variants
4
2
1
0.5
0
250
500
750
Codon
25
20
15
10
5
0
Driver
Passenger
Bone/Soft tissue
Breast
Colon/Rectum
CUP
Esophagus
Lung
Other
Ovary
Pancreas
Prostate
Skin
Urinary tract
Uterus
Monoallelic: SNV MNV INDEL in frame SNV nonsense MNV nonsense INDEL out of frame
Biallelic/Multihit: SNV MNV INDEL in frame SNV nonsense MNV nonsense INDEL out of frame
Hotspot:

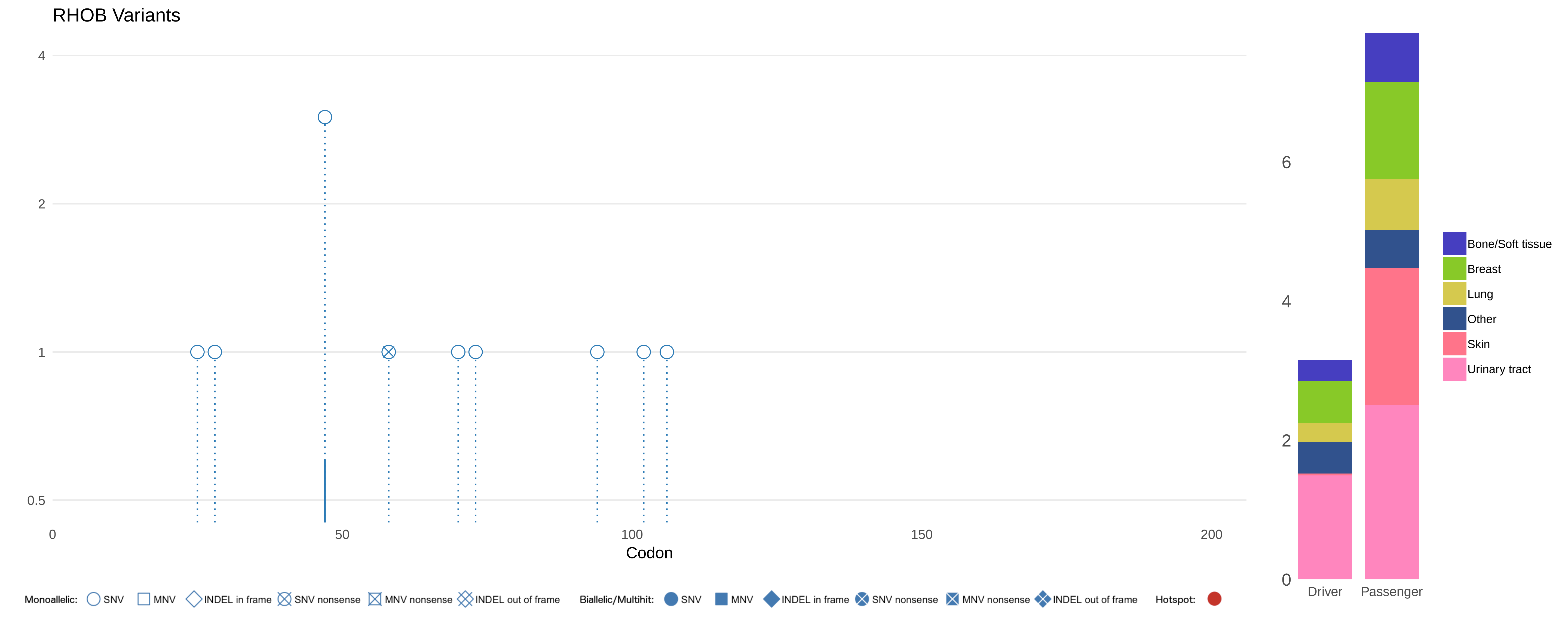

RHOB Variants
4
2
1
0.5
0
50
100
150
200
Codon
Monoallelic: SNV MNV INDEL in frame SNV nonsense MNV nonsense INDEL out of frame
Biallelic/Multihit: SNV MNV INDEL in frame SNV nonsense MNV nonsense INDEL out of frame
Hotspot:
6
4
2
0
Driver
Passenger
Bone/Soft tissue
Breast
Lung
Other
Skin
Urinary tract

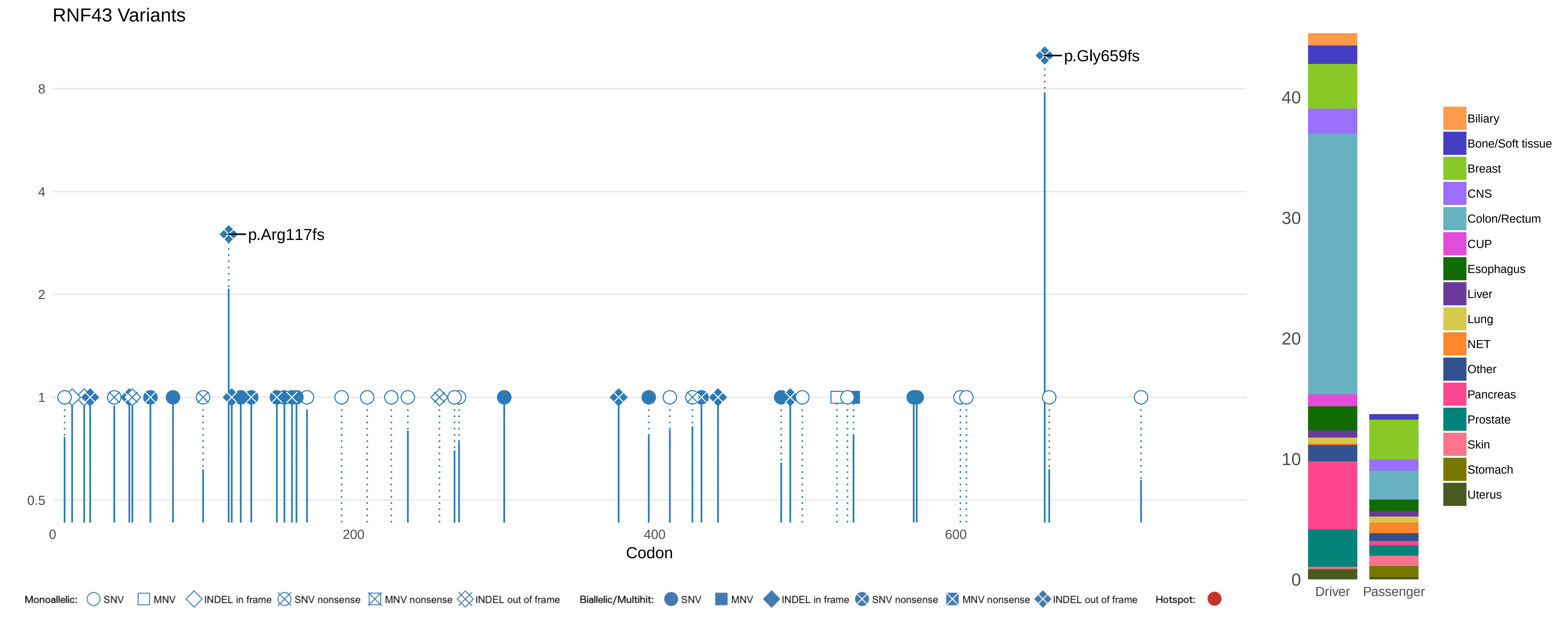

RNF43 Variants
p.Gly659fs
p.Arg117fs
8
4
2
1
0.5
0
200
400
600
Codon
Monoallelic: SNV MNV INDEL in frame SNV nonsense MNV nonsense INDEL out of frame
Biallelic/Multihit: SNV MNV INDEL in frame SNV nonsense MNV nonsense INDEL out of frame
Hotspot:
40
30
20
10
0
Driver
Passenger
Biliary
Bone/Soft tissue
Breast
CNS
Colon/Rectum
CUP
Esophagus
Liver
Lung
NET
Other
Pancreas
Prostate
Skin
Stomach
Uterus

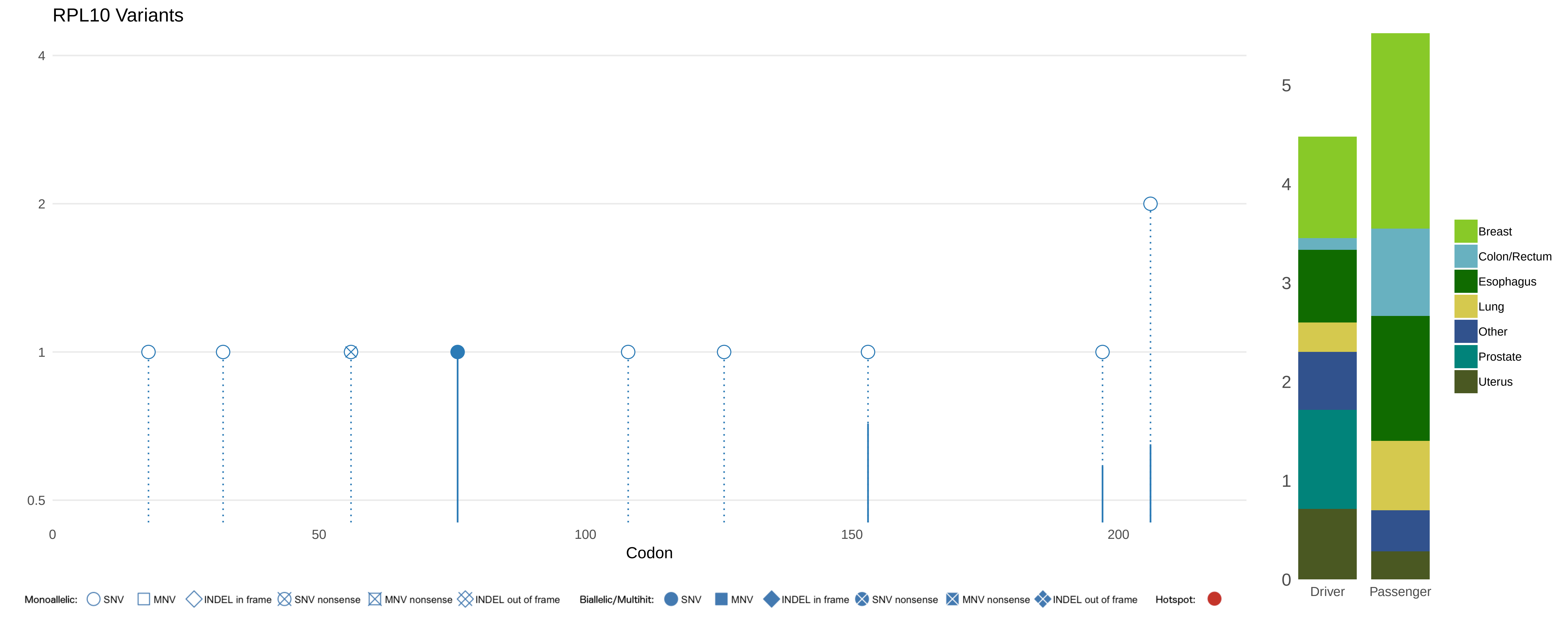

RPL10 Variants
4
2
1
0.5
0
50
100
150
200
Codon
5
4
3
2
1
0
Driver
Passenger
Breast
Colon/Rectum
Esophagus
Lung
Other
Prostate
Uterus
Monoallelic: SNV MNV INDEL in frame SNV nonsense MNV nonsense INDEL out of frame
Biallelic/Multihit: SNV MNV INDEL in frame SNV nonsense MNV nonsense INDEL out of frame
Hotspot:

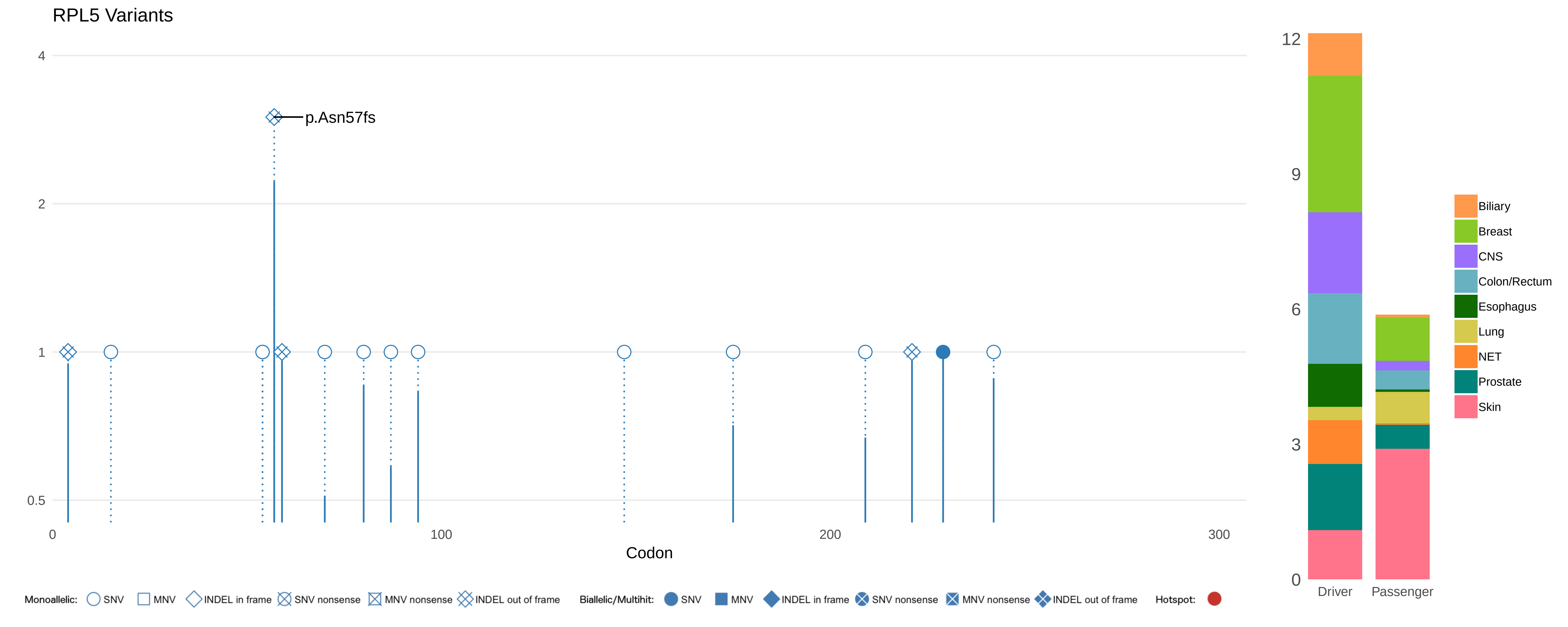
RPL5 Variants
4
2
1
0.5
p.Asn57fs
0
100
200
300
Codon
12
9
6
3
0
Driver
Passenger
Biliary
Breast
CNS
Colon/Rectum
Esophagus
Lung
NET
Prostate
Skin
Monoallelic: SNV MNV INDEL in frame SNV nonsense MNV nonsense INDEL out of frame
Biallelic/Multihit: SNV MNV INDEL in frame SNV nonsense MNV nonsense INDEL out of frame
Hotspot:

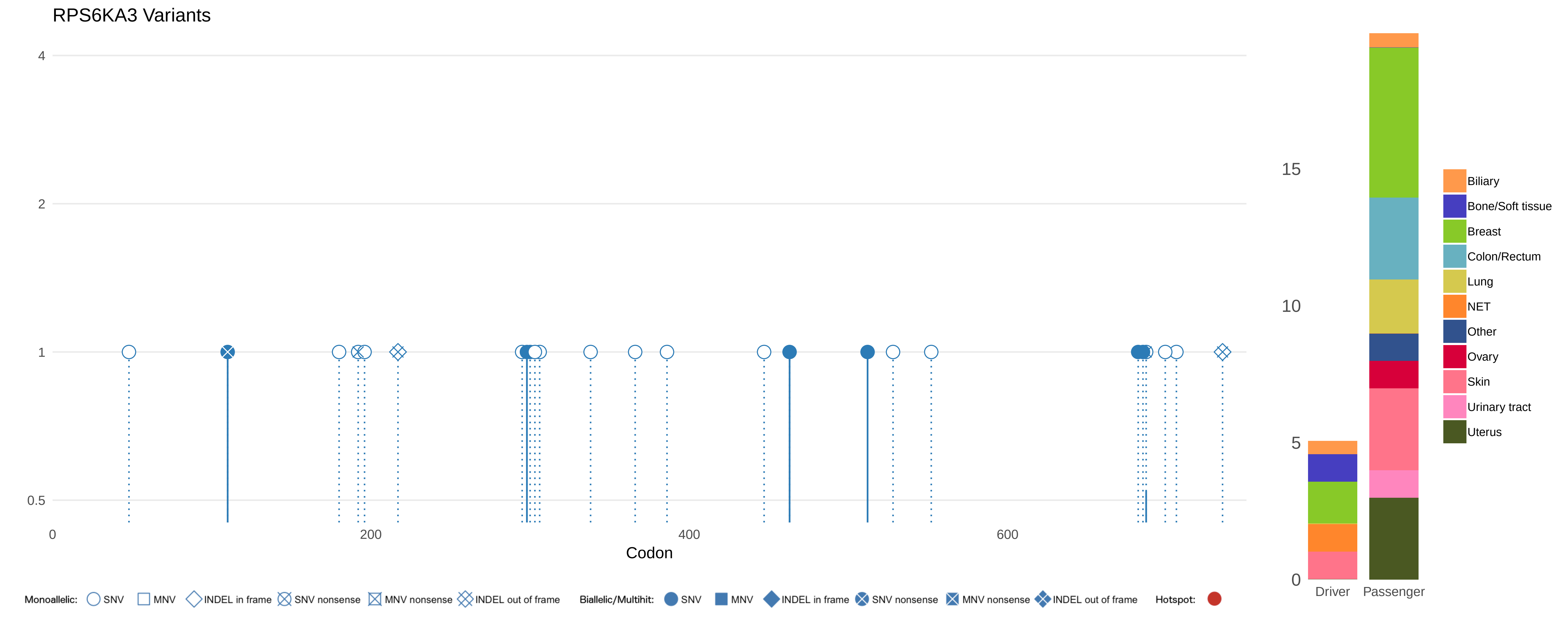
RPS6KA3 Variants
4
2
1
0.5
0
200
400
600
Codon
Monoallelic: SNV MNV INDEL in frame SNV nonsense MNV nonsense INDEL out of frame
Biallelic/Multihit: SNV MNV INDEL in frame SNV nonsense MNV nonsense INDEL out of frame
Hotspot:
15
10
5
0
Driver
Passenger
Biliary
Bone/Soft tissue
Breast
Colon/Rectum
Lung
NET
Other
Ovary
Skin
Urinary tract
Uterus

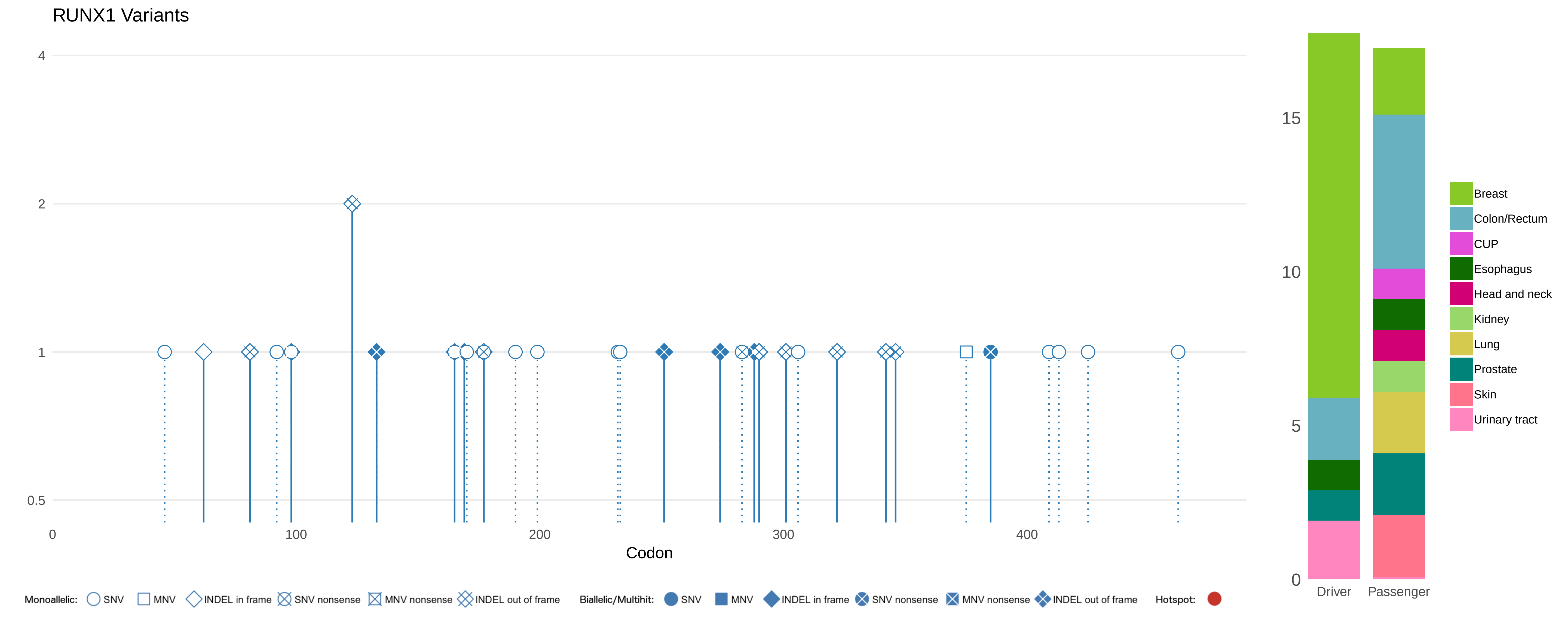

RUNX1 Variants
4
2
1
0.5
0
100
200
300
400
Codon
15
10
5
0
Driver
Passenger
Breast
Colon/Rectum
CUP
Esophagus
Head and neck
Kidney
Lung
Prostate
Skin
Urinary tract
Monoallelic: SNV MNV INDEL in frame SNV nonsense MNV nonsense INDEL out of frame
Biallelic/Multihit: SNV MNV INDEL in frame SNV nonsense MNV nonsense INDEL out of frame
Hotspot:

RXRA Variants

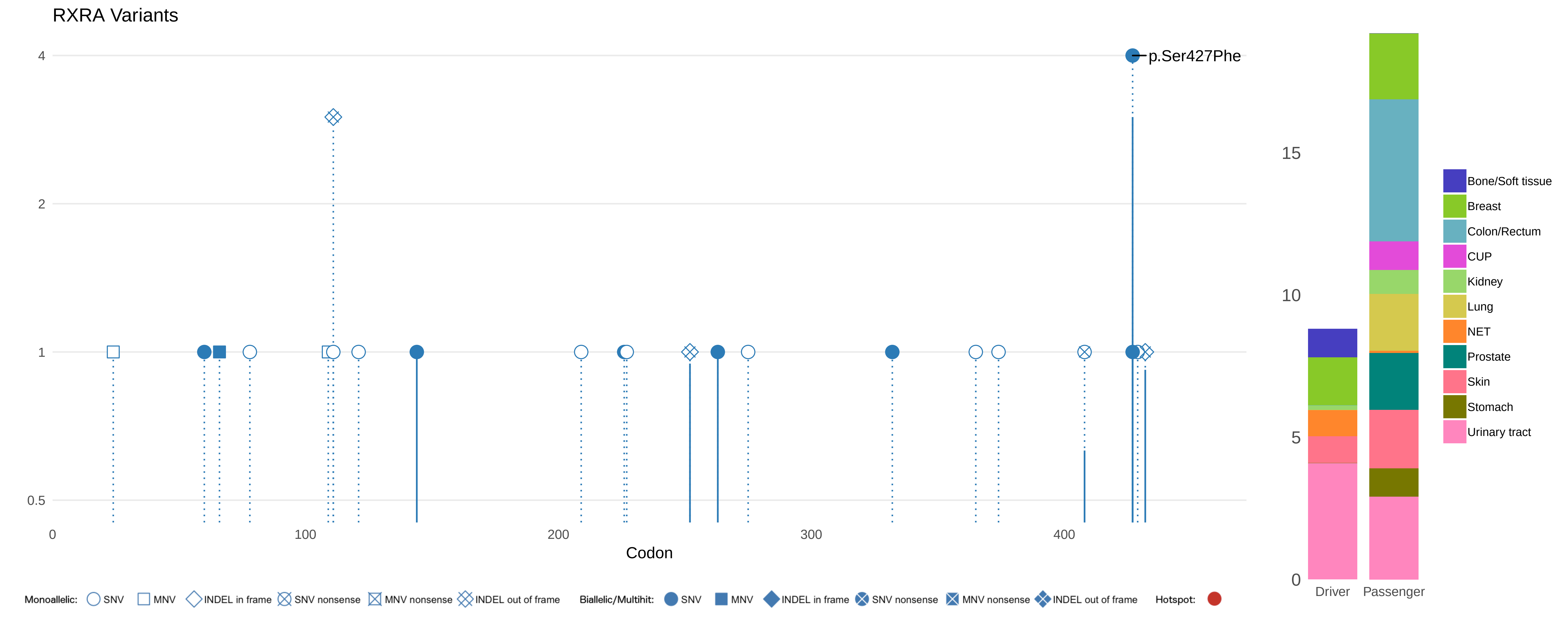

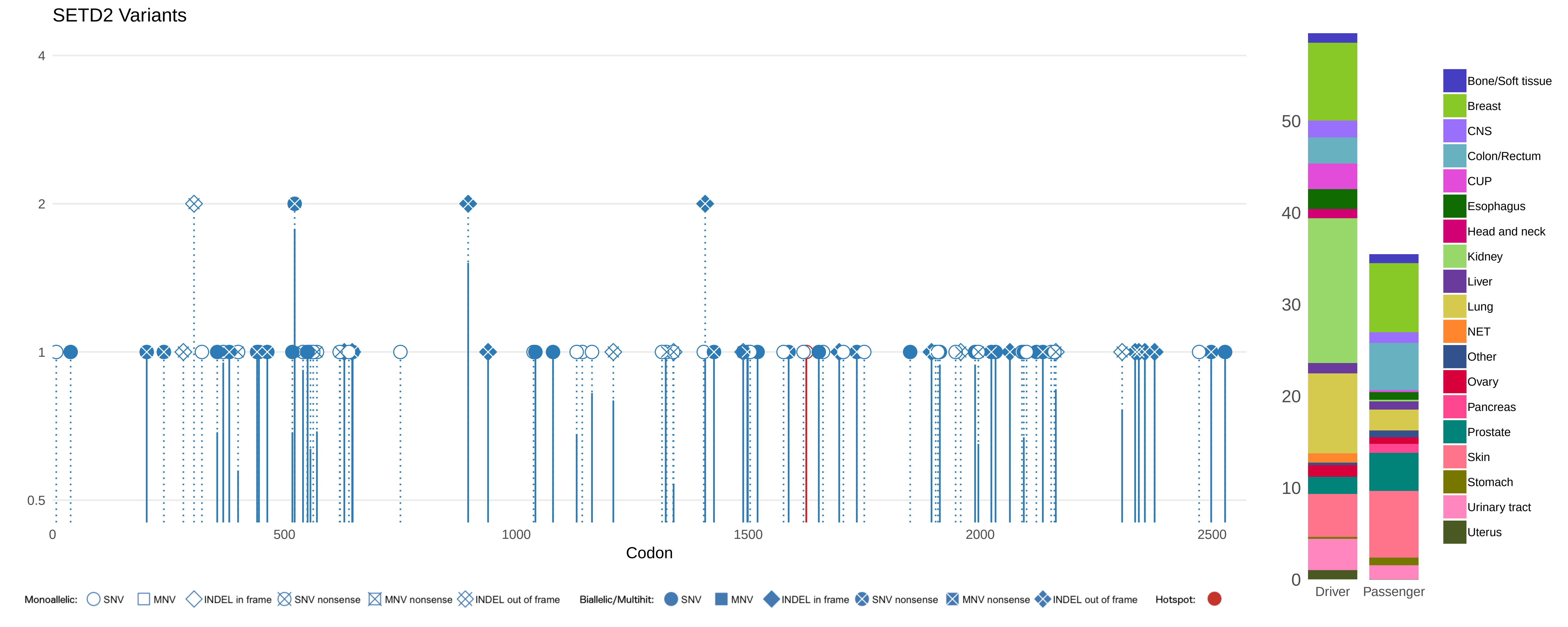
SETD2 Variants
4
2
1
0.5
0
500
1000
1500
2000
2500
Codon
Monoallelic: SNV MNV INDEL in frame SNV nonsense MNV nonsense INDEL out of frame
Biallelic/Multihit: SNV MNV INDEL in frame SNV nonsense MNV nonsense INDEL out of frame
Hotspot:
50
40
30
20
10
0
Driver
Passenger
Bone/Soft tissue
Breast
CNS
Colon/Rectum
CUP
Esophagus
Head and neck
Kidney
Liver
Lung
NET
Other
Ovary
Pancreas
Prostate
Skin
Stomach
Urinary tract
Uterus

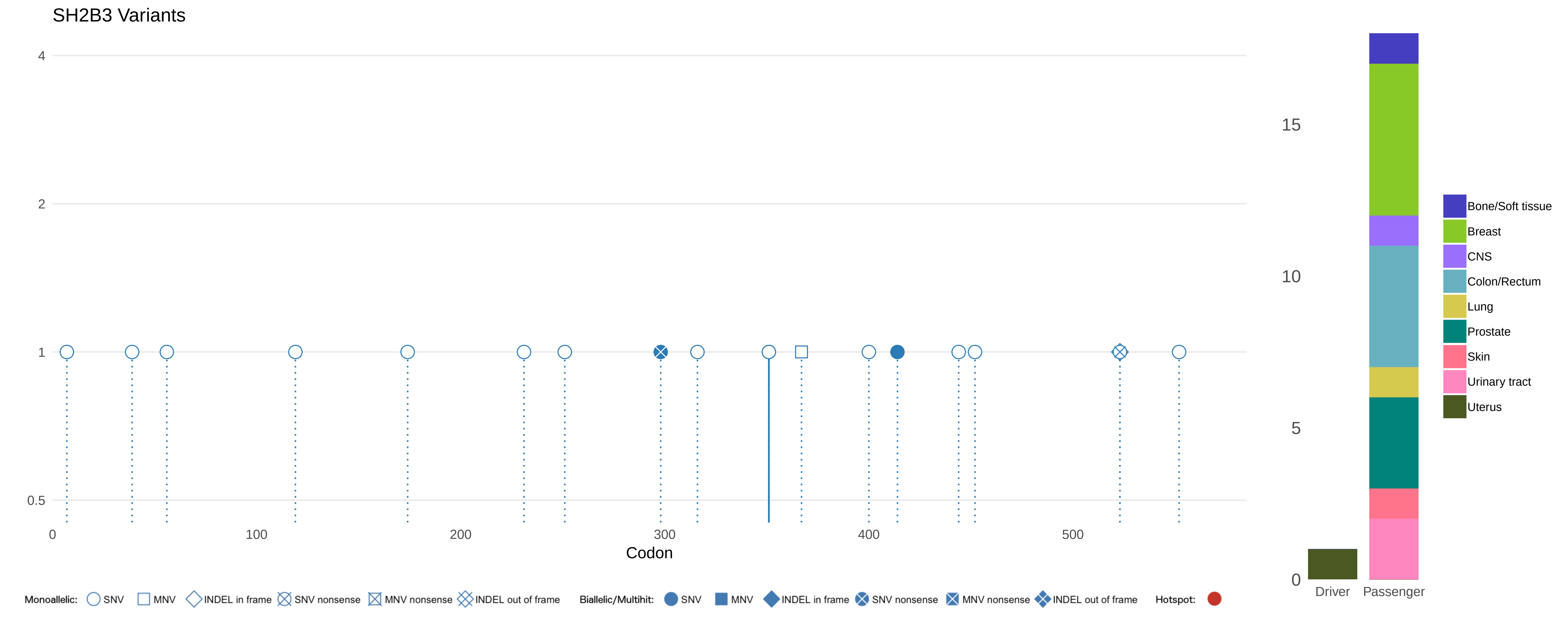

SH2B3 Variants
4
2
1
0.5
0
100
200
300
400
500
Codon
15
10
5
0
Driver
Passenger
Bone/Soft tissue
Breast
CNS
Colon/Rectum
Lung
Prostate
Skin
Urinary tract
Uterus
Monoallelic: SNV MNV INDEL in frame SNV nonsense MNV nonsense INDEL out of frame
Biallelic/Multihit: SNV MNV INDEL in frame SNV nonsense MNV nonsense INDEL out of frame
Hotspot:

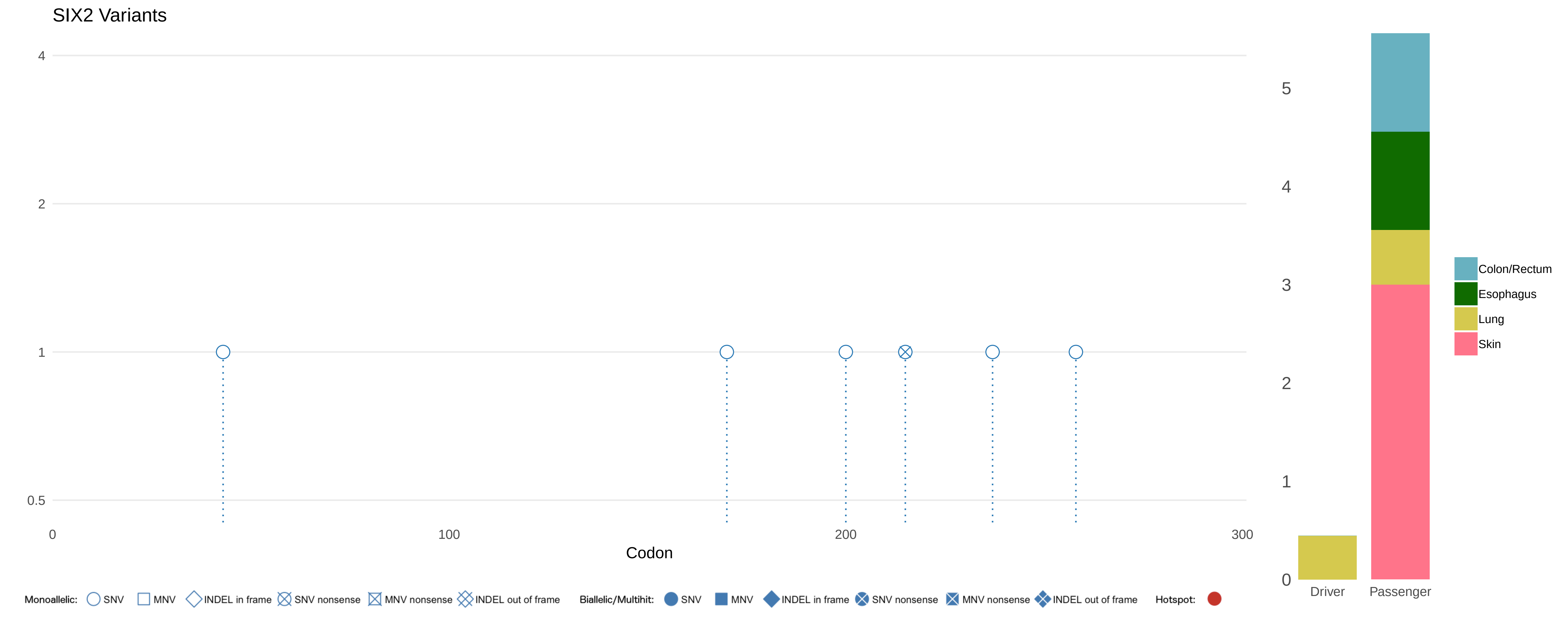
SIX2 Variants
4
2
1
0.5
0
100
200
300
Codon
5
4
3
2
1
0
Driver
Passenger
Colon/Rectum
Esophagus
Lung
Skin
Monoallelic: SNV MNV INDEL in frame SNV nonsense MNV nonsense INDEL out of frame
Biallelic/Multihit: SNV MNV INDEL in frame SNV nonsense MNV nonsense INDEL out of frame
Hotspot:

SMAD2 Variants

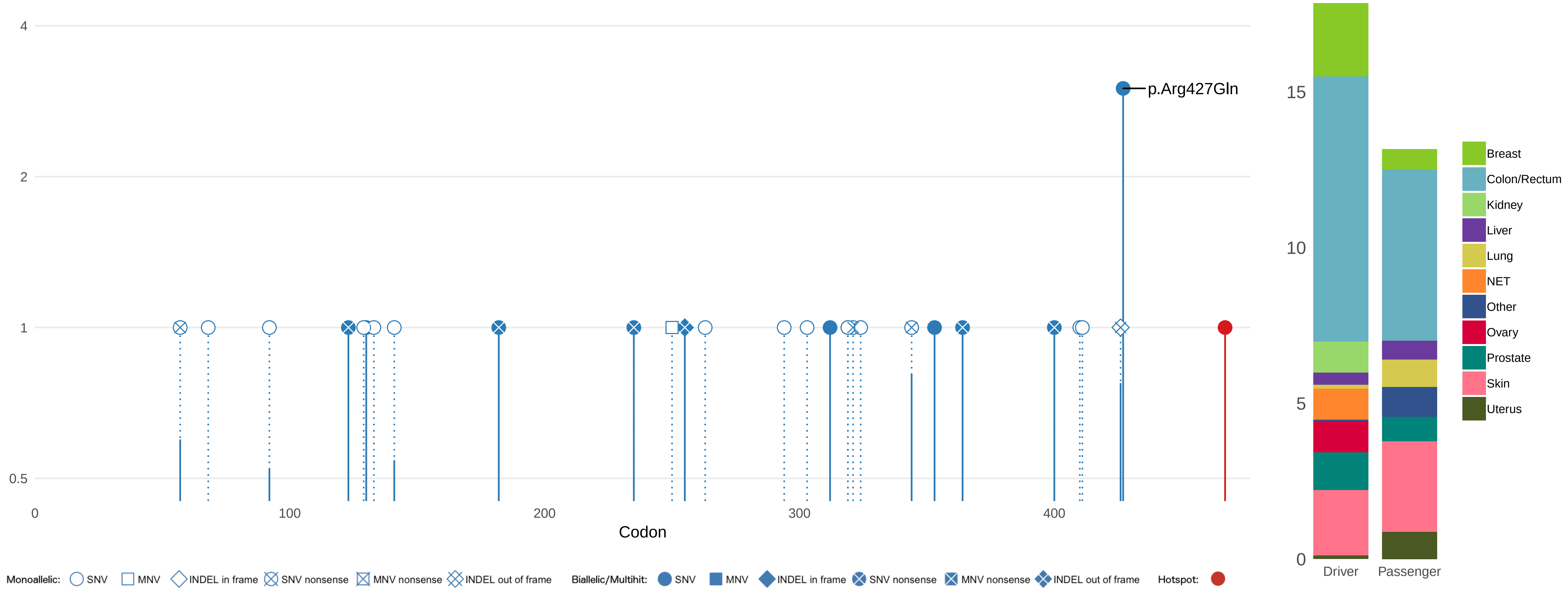

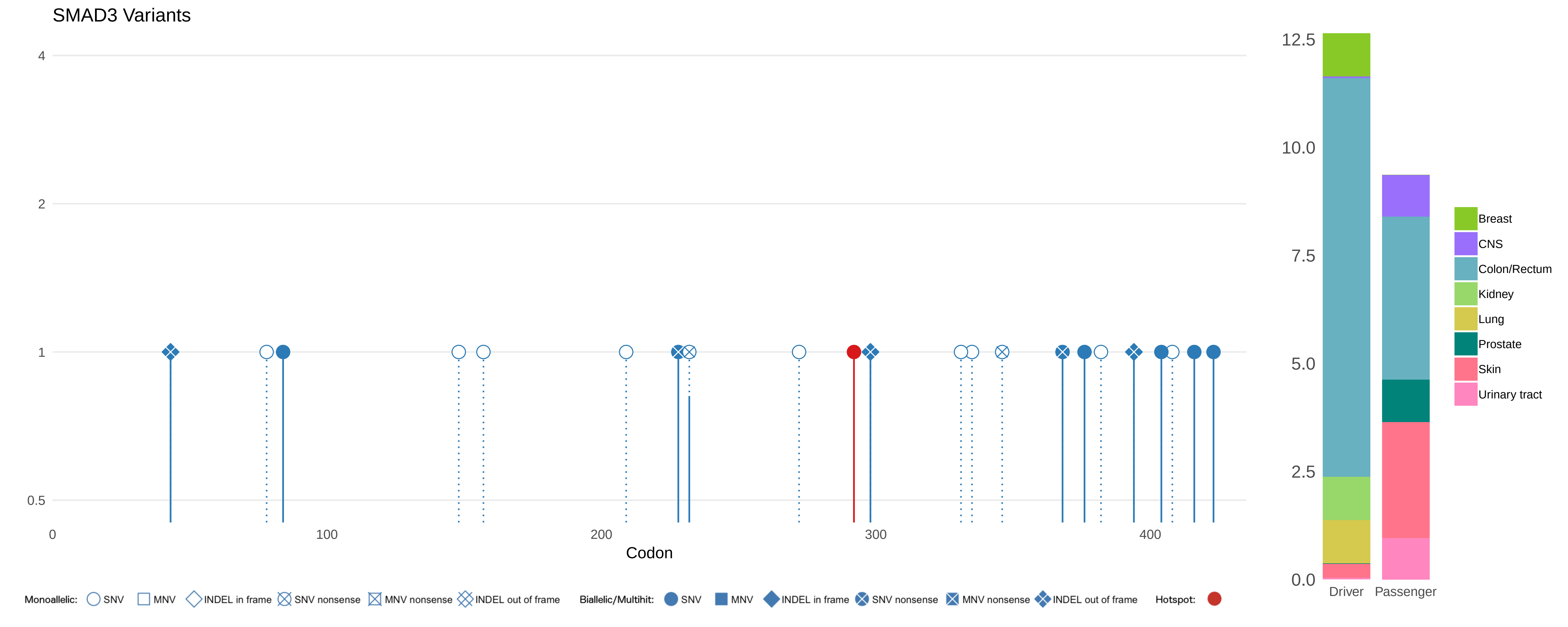
SMAD3 Variants
4
2
1
0.5
0
100
200
300
400
Codon
12.5
10.0
7.5
5.0
2.5
0.0
Driver
Passenger
Breast
CNS
Colon/Rectum
Kidney
Lung
Prostate
Skin
Urinary tract
Monoallelic: SNV MNV INDEL in frame SNV nonsense MNV nonsense INDEL out of frame
Biallelic/Multihit: SNV MNV INDEL in frame SNV nonsense MNV nonsense INDEL out of frame
Hotspot:

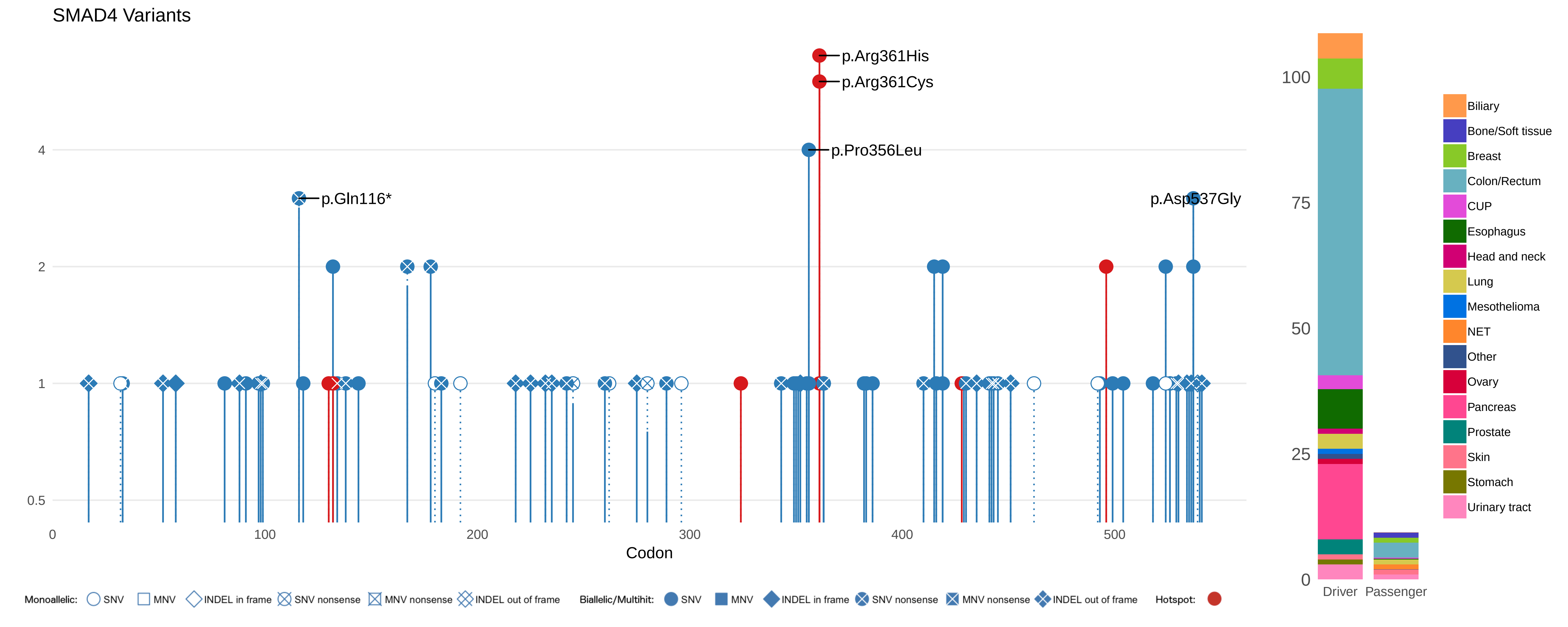

SMAD4 Variants
p.Arg361His
p.Arg361Cys
p.Pro356Leu
p.Gln116*
p.Asp537Gly
4
2
1
0.5
0
100
200
300
400
500
Codon
100
75
50
25
0
Driver
Passenger
Biliary
Bone/Soft tissue
Breast
Colon/Rectum
CUP
Esophagus
Head and neck
Lung
Mesothelioma
NET
Other
Ovary
Pancreas
Prostate
Skin
Stomach
Urinary tract
Monoallelic: SNV MNV INDEL in frame SNV nonsense MNV nonsense INDEL out of frame
Biallelic/Multihit: SNV MNV INDEL in frame SNV nonsense MNV nonsense INDEL out of frame
Hotspot:

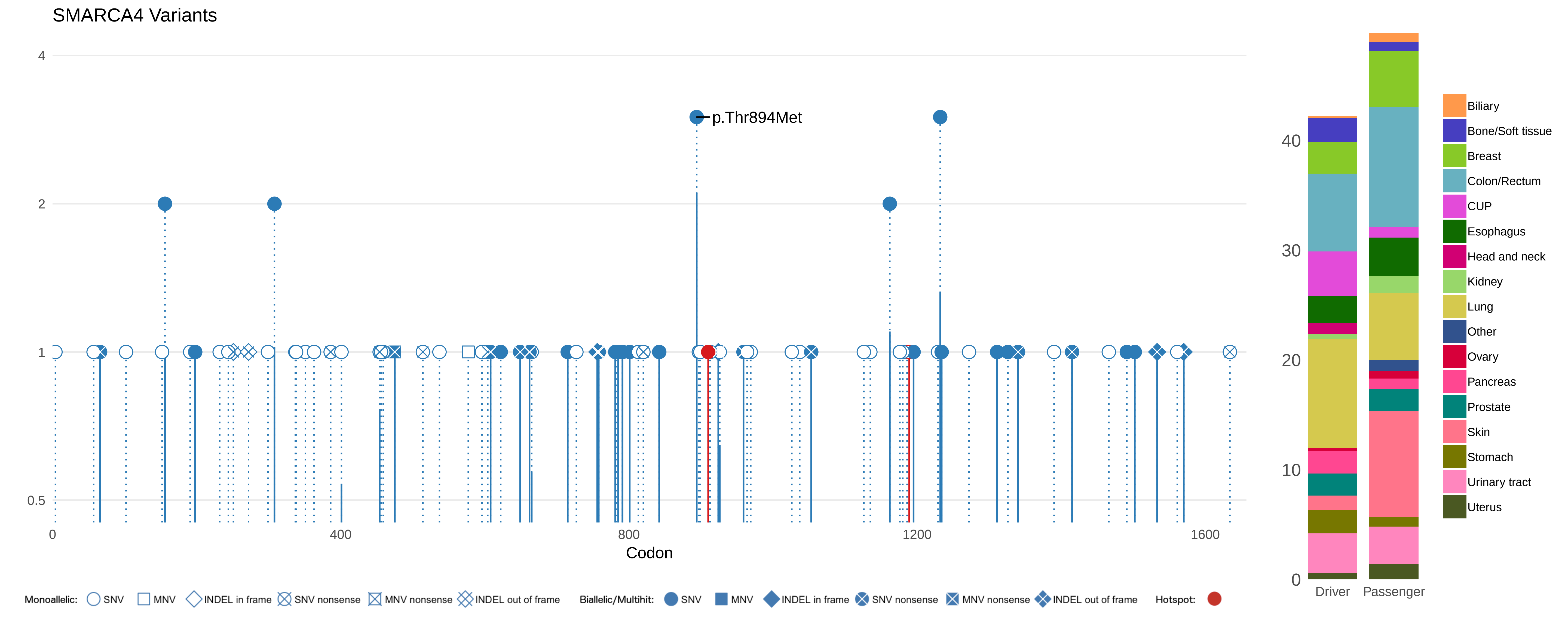

SMARCA4 Variants
p.Thr894Met
4
2
1
0.5
0
400
800
1200
1600
Codon
40
30
20
10
0
Driver
Passenger
Biliary
Bone/Soft tissue
Breast
Colon/Rectum
CUP
Esophagus
Head and neck
Kidney
Lung
Other
Ovary
Pancreas
Prostate
Skin
Stomach
Urinary tract
Uterus
Monoallelic: SNV MNV INDEL in frame SNV nonsense MNV nonsense INDEL out of frame
Biallelic/Multihit: SNV MNV INDEL in frame SNV nonsense MNV nonsense INDEL out of frame
Hotspot:

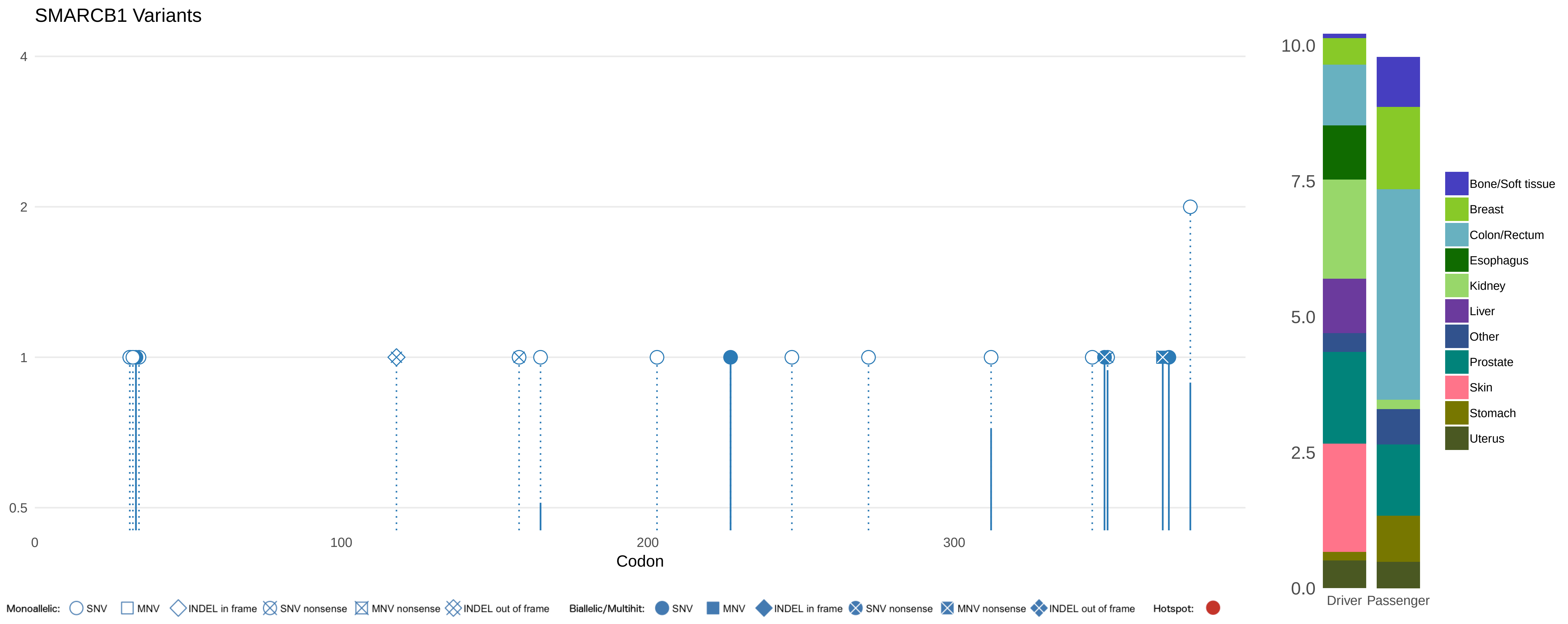
SMARCB1 Variants
4
2
1
0.5
0
100
200
300
Codon
10.0
7.5
5.0
2.5
0.0
Driver
Passenger
Bone/Soft tissue
Breast
Colon/Rectum
Esophagus
Kidney
Liver
Other
Prostate
Skin
Stomach
Uterus
Monoallelic: SNV MNV INDEL in frame SNV nonsense MNV nonsense INDEL out of frame
Biallelic/Multihit: SNV MNV INDEL in frame SNV nonsense MNV nonsense INDEL out of frame
Hotspot:

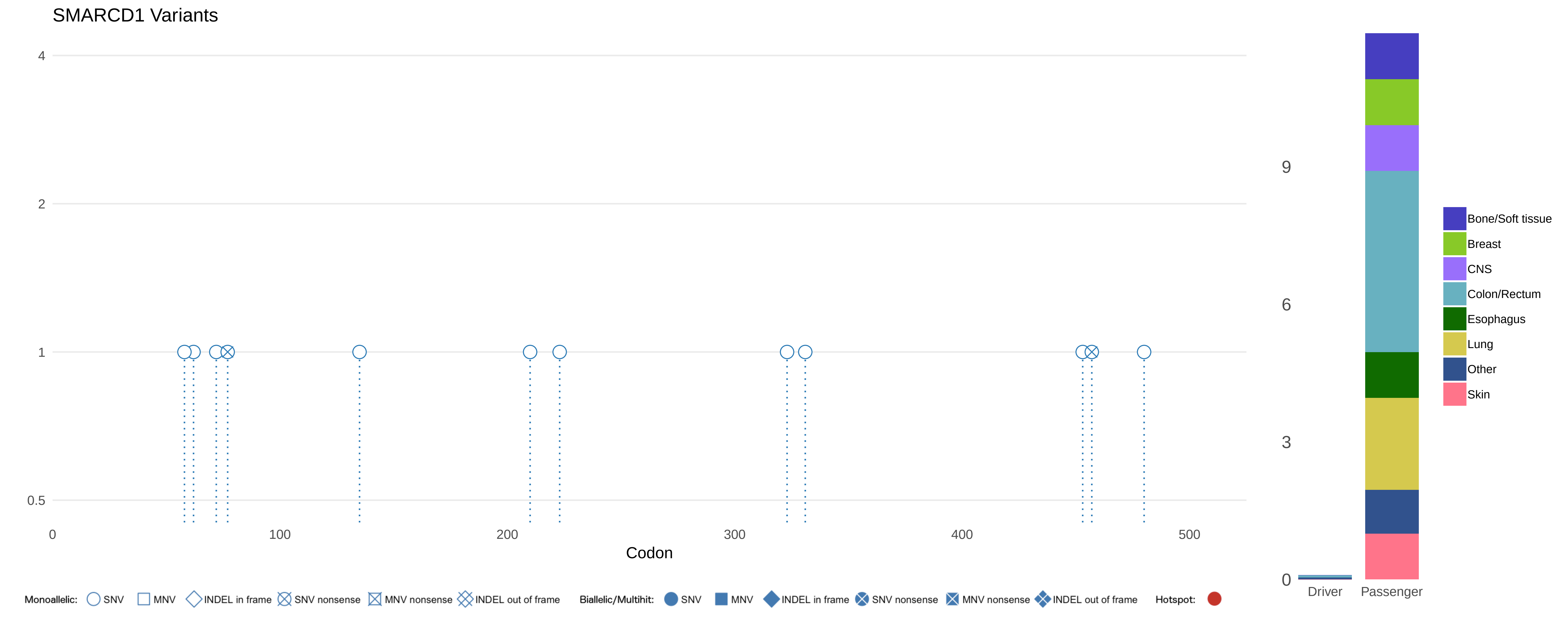
SMARCD1 Variants
4
2
1
0.5
0
100
200
300
400
500
Codon
9
6
3
0
Driver
Passenger
Bone/Soft tissue
Breast
CNS
Colon/Rectum
Esophagus
Lung
Other
Skin
Monoallelic: SNV MNV INDEL in frame SNV nonsense MNV nonsense INDEL out of frame
Biallelic/Multihit: SNV MNV INDEL in frame SNV nonsense MNV nonsense INDEL out of frame
Hotspot:

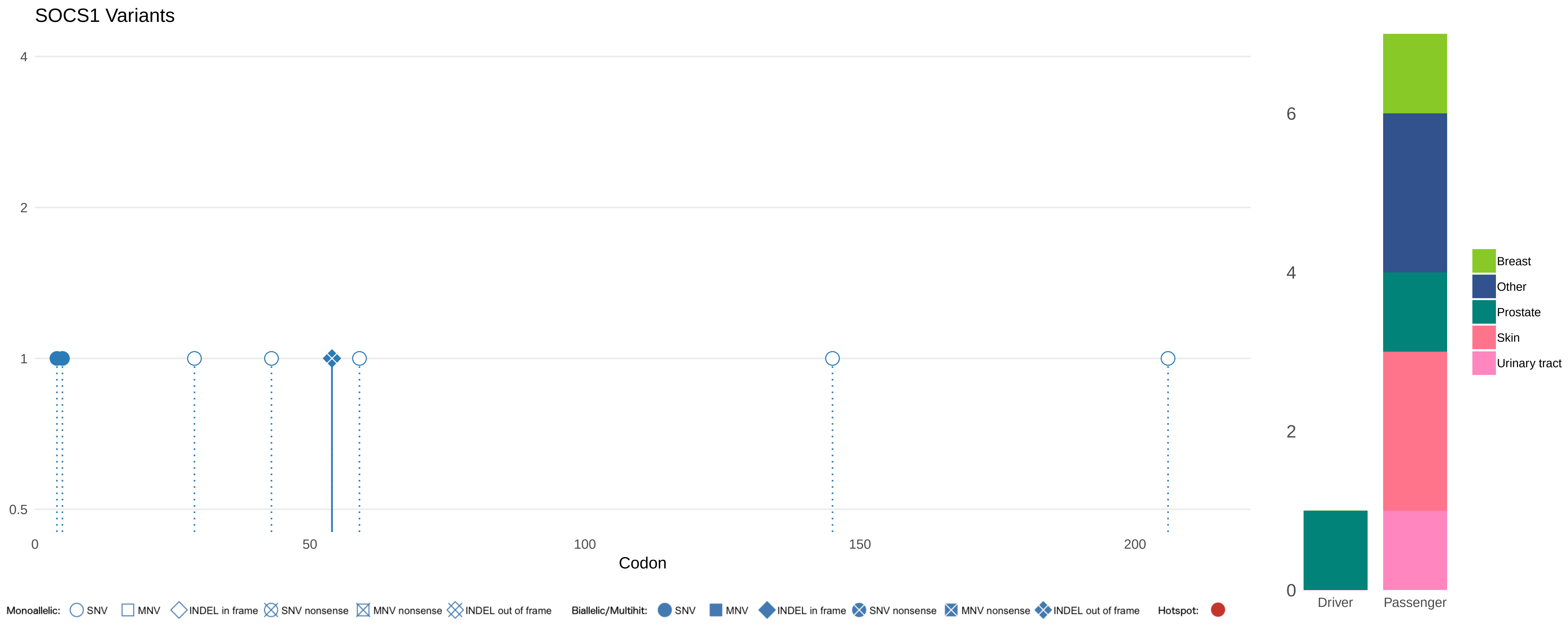

SOCS1 Variants
4
2
1
0.5
0
50
100
150
200
Codon
6
4
2
0
Driver
Passenger
Breast
Other
Prostate
Skin
Urinary tract
Monoallelic: SNV MNV INDEL in frame SNV nonsense MNV nonsense INDEL out of frame
Biallelic/Multihit: SNV MNV INDEL in frame SNV nonsense MNV nonsense INDEL out of frame
Hotspot:

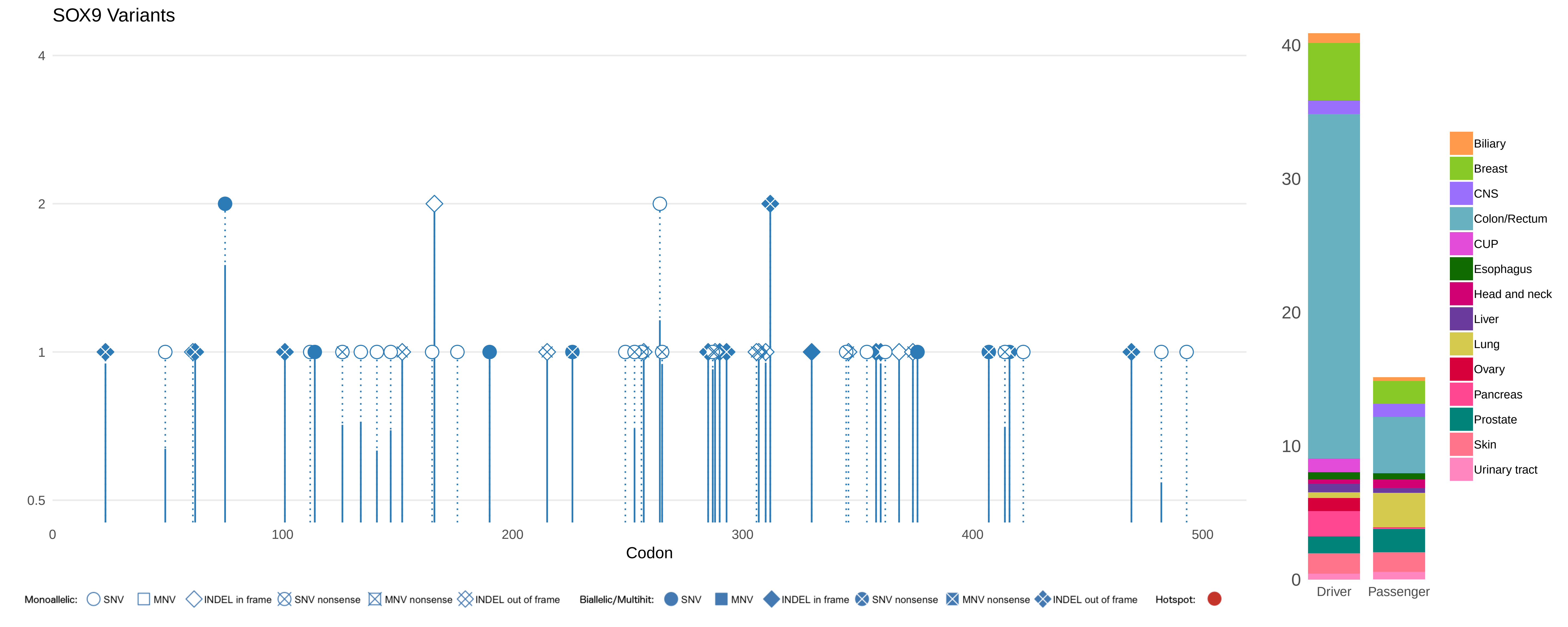
SOX9 Variants
4
2
1
0.5
0
100
200
300
400
500
Codon
40
30
20
10
0
Driver
Passenger
Biliary
Breast
CNS
Colon/Rectum
CUP
Esophagus
Head and neck
Liver
Lung
Ovary
Pancreas
Prostate
Skin
Urinary tract
Monoallelic:
SNV
MNV
INDEL in frame
SNV nonsense
MNV nonsense
INDEL out of frame
Biallelic/Multihit:
SNV
MNV
INDEL in frame
SNV nonsense
MNV nonsense
INDEL out of frame
Hotspot:

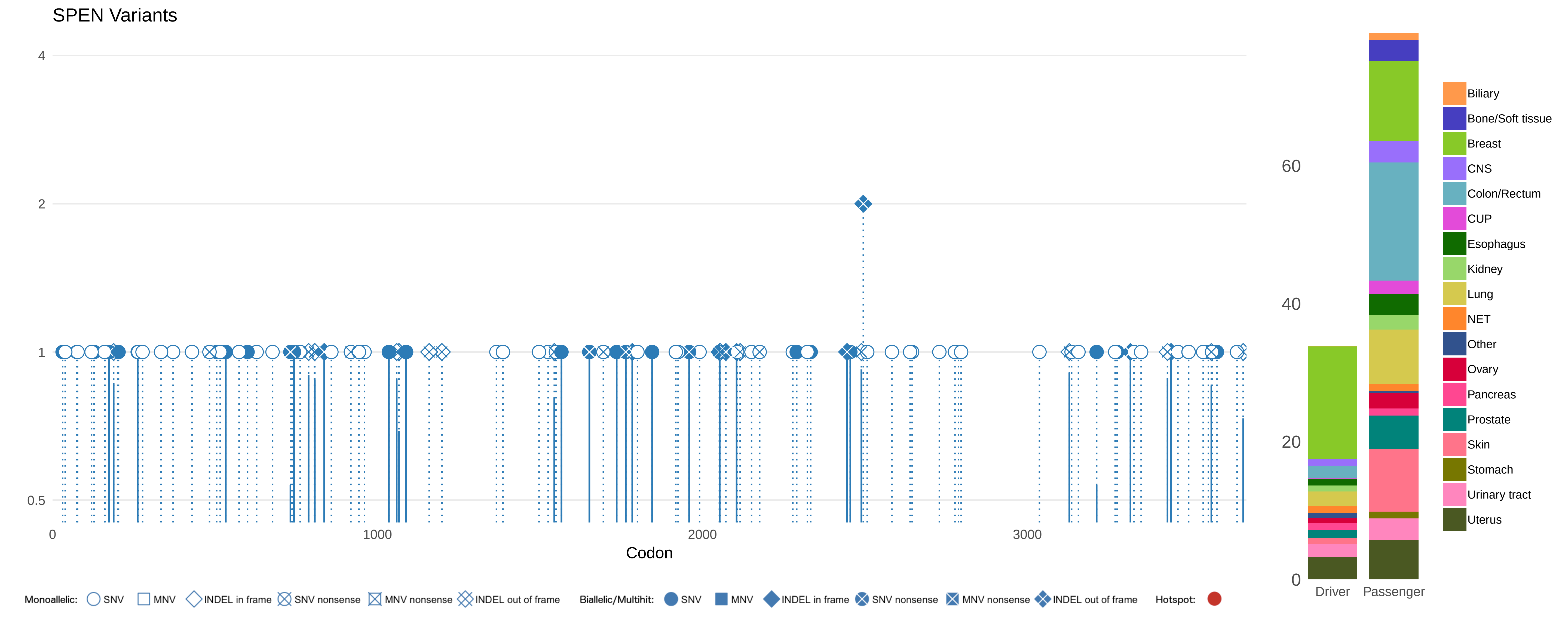

SPEN Variants
4
2
1
0.5
0
1000
2000
3000
Codon
60
40
20
0
Driver
Passenger
Biliary
Bone/Soft tissue
Breast
CNS
Colon/Rectum
CUP
Esophagus
Kidney
Lung
NET
Other
Ovary
Pancreas
Prostate
Skin
Stomach
Urinary tract
Uterus
Monoallelic: SNV MNV INDEL in frame SNV nonsense MNV nonsense INDEL out of frame
Biallelic/Multihit: SNV MNV INDEL in frame SNV nonsense MNV nonsense INDEL out of frame
Hotspot:

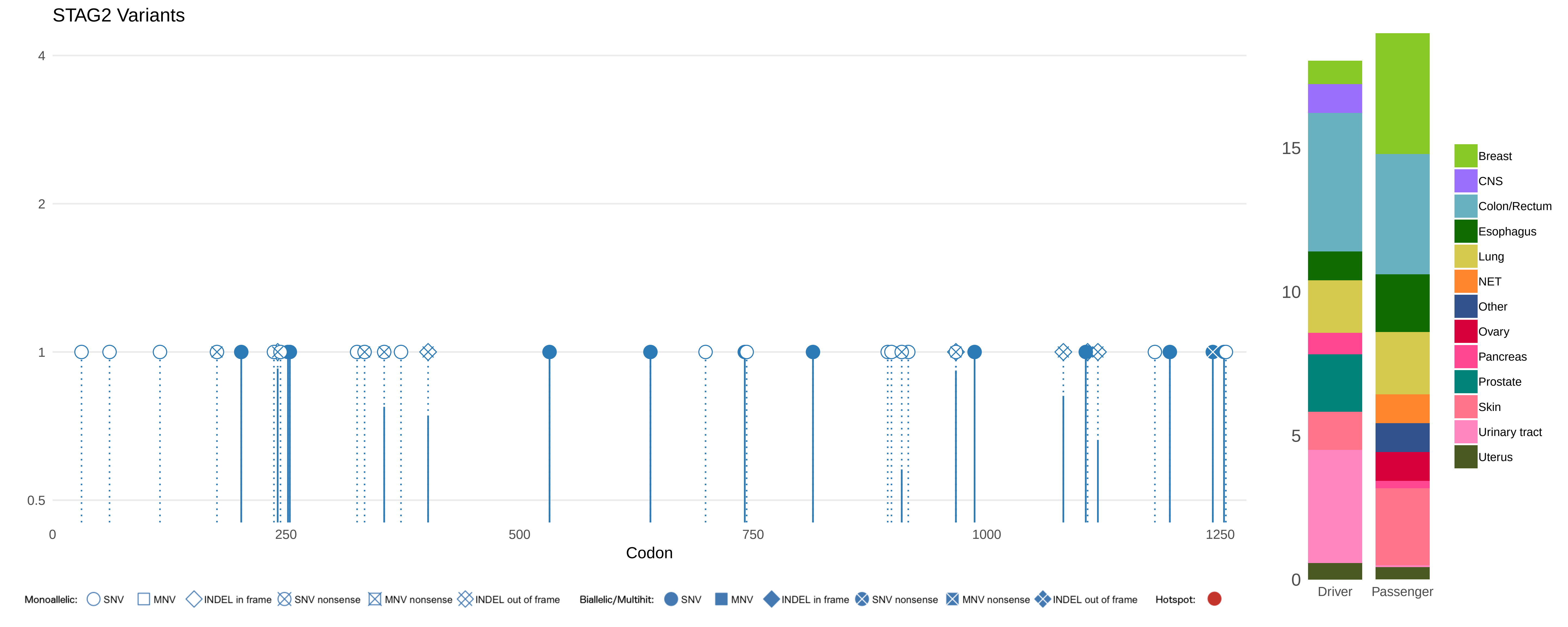

STAG2 Variants
4
2
1
0.5
0
250
500
750
1000
1250
Codon
Monoallelic:
SNV
MNV
INDEL in frame
SNV nonsense
MNV nonsense
INDEL out of frame
Biallelic/Multihit:
SNV
MNV
INDEL in frame
SNV nonsense
MNV nonsense
INDEL out of frame
Hotspot:
15
10
5
0
Driver
Passenger
Breast
CNS
Colon/Rectum
Esophagus
Lung
NET
Other
Ovary
Pancreas
Prostate
Skin
Urinary tract
Uterus

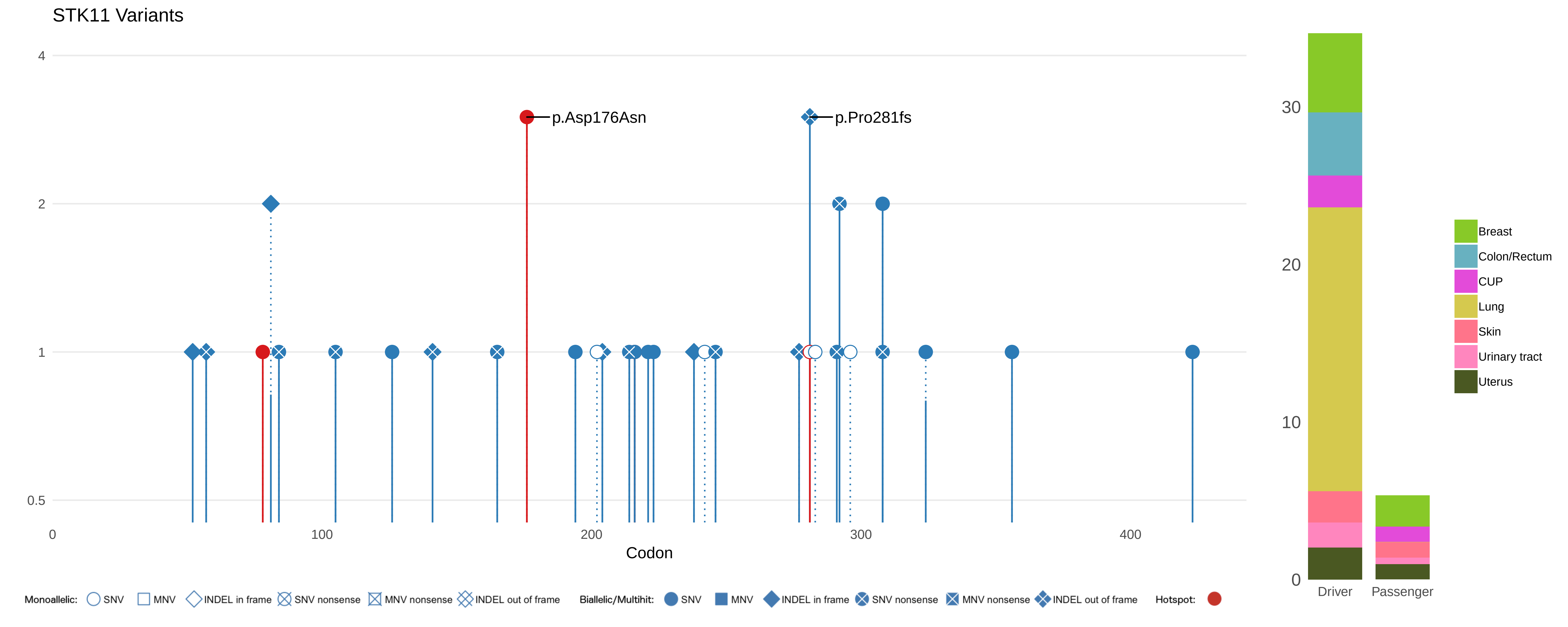
STK11 Variants
p.Asp176Asn
p.Pro281fs
4
2
1
0.5
0
100
200
300
400
Codon
30
20
10
0
Driver
Passenger
Breast
Colon/Rectum
CUP
Lung
Skin
Urinary tract
Uterus
Monoallelic: SNV MNV INDEL in frame SNV nonsense MNV nonsense INDEL out of frame
Biallelic/Multihit: SNV MNV INDEL in frame SNV nonsense MNV nonsense INDEL out of frame
Hotspot:

SUFU Variants

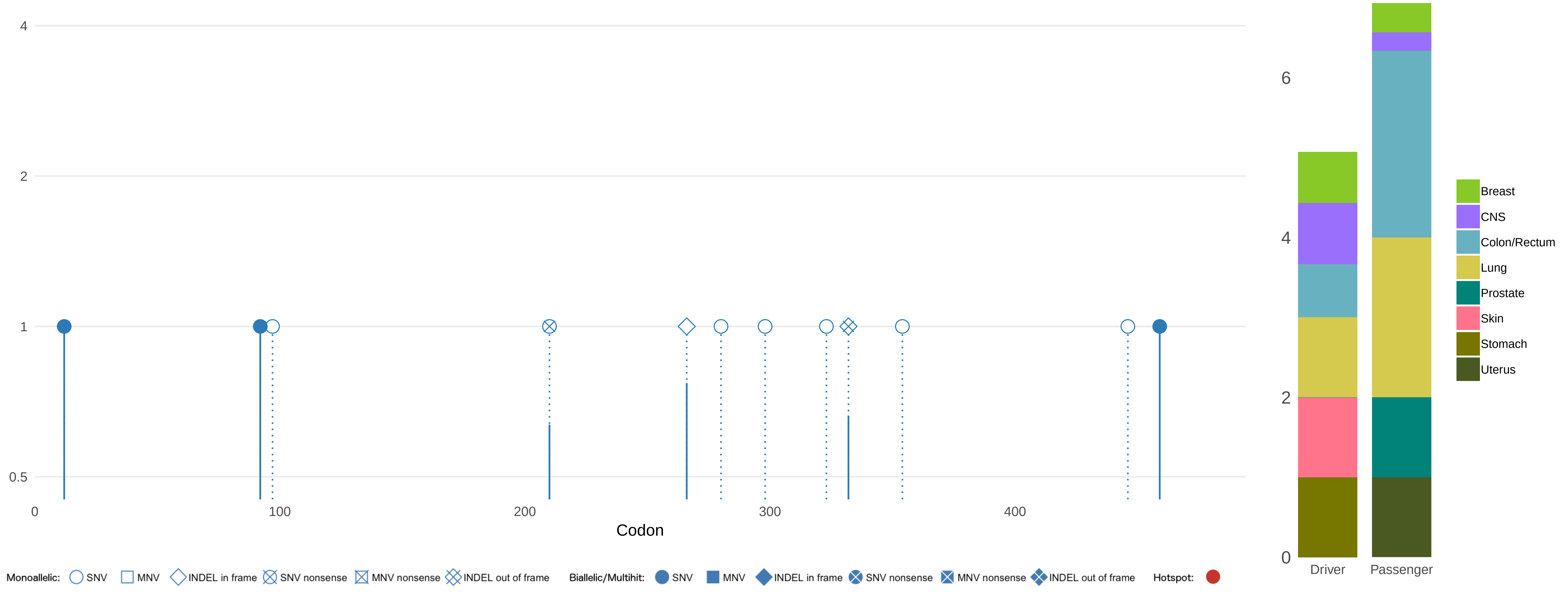

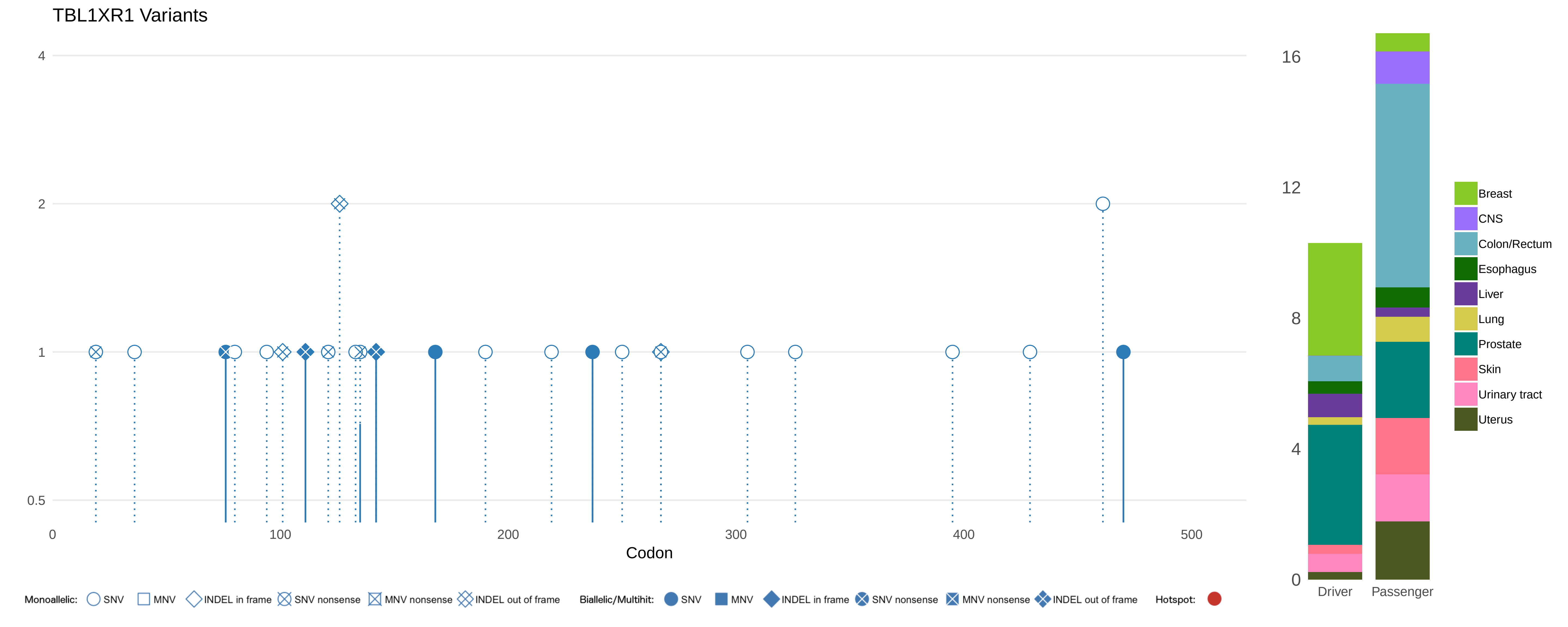
TBL1XR1 Variants
4
2
1
0.5
0
100
200
300
400
500
Codon
16
12
8
4
0
Driver
Passenger
Breast
CNS
Colon/Rectum
Esophagus
Liver
Lung
Prostate
Skin
Urinary tract
Uterus
Monoallelic: SNV MNV INDEL in frame SNV nonsense MNV nonsense INDEL out of frame
Biallelic/Multihit: SNV MNV INDEL in frame SNV nonsense MNV nonsense INDEL out of frame
Hotspot:

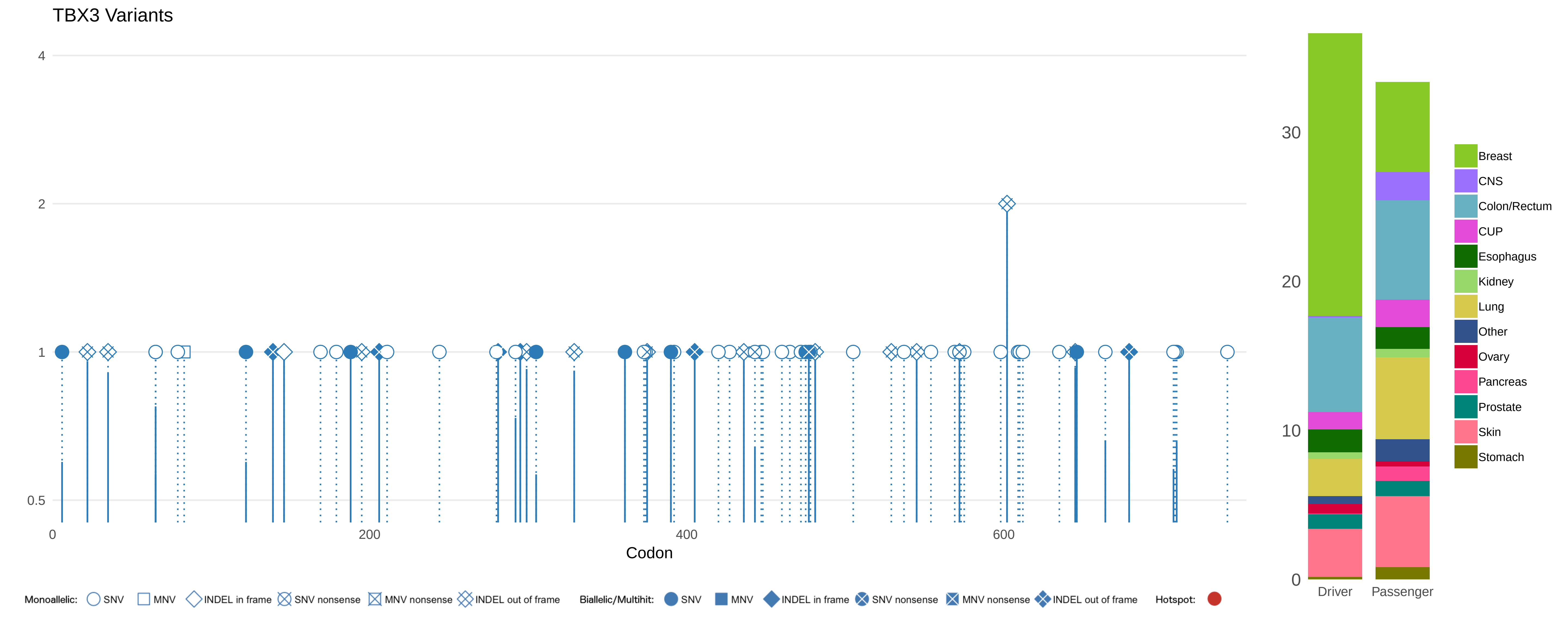
TBX3 Variants
4
2
1
0.5
0
200
400
600
Codon
Monoallelic: SNV MNV INDEL in frame SNV nonsense MNV nonsense INDEL out of frame
Biallelic/Multihit: SNV MNV INDEL in frame SNV nonsense MNV nonsense INDEL out of frame
Hotspot:
30
20
10
0
Driver
Passenger
Breast
CNS
Colon/Rectum
CUP
Esophagus
Kidney
Lung
Other
Ovary
Pancreas
Prostate
Skin
Stomach

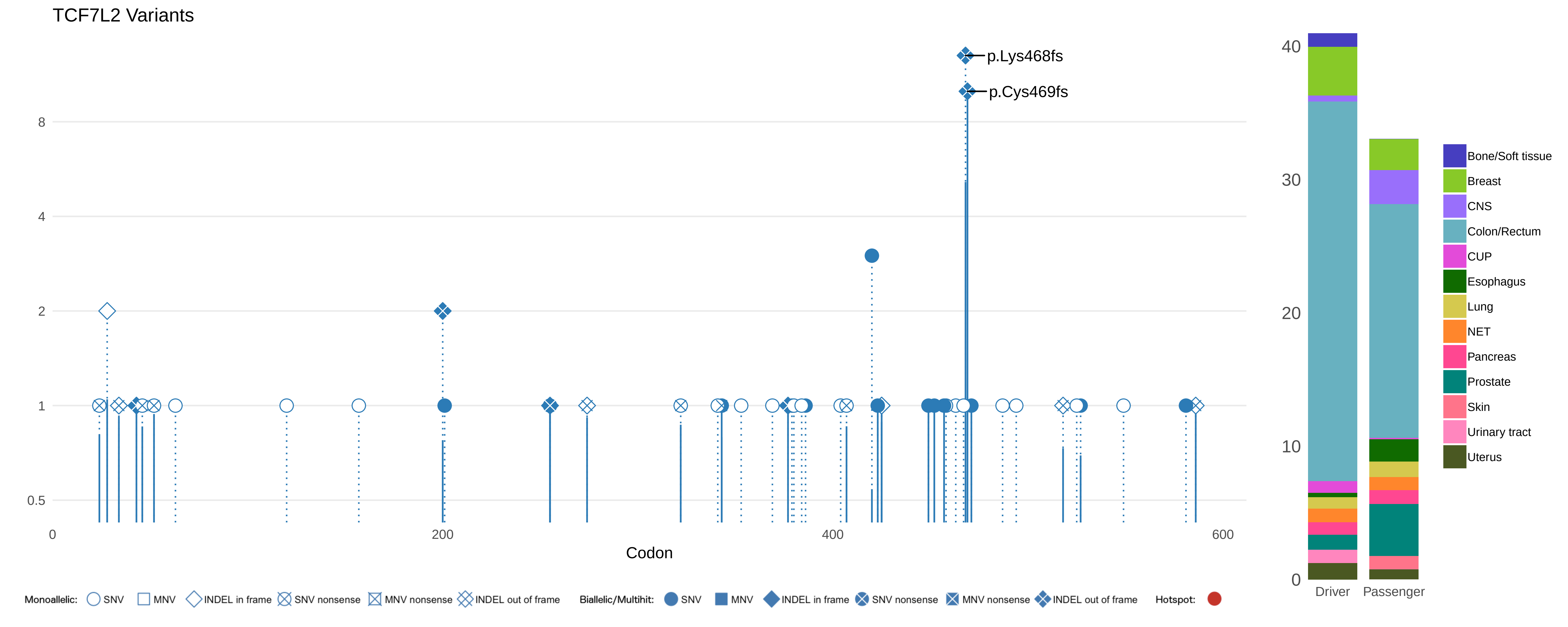

TCF7L2 Variants
p.Lys468fs
p.Cys469fs
8
4
2
1
0.5
0
200
400
600
Codon
40
30
20
10
0
Driver
Passenger
Bone/Soft tissue
Breast
CNS
Colon/Rectum
CUP
Esophagus
Lung
NET
Pancreas
Prostate
Skin
Urinary tract
Uterus
Monoallelic: SNV MNV INDEL in frame SNV nonsense MNV nonsense INDEL out of frame
Biallelic/Multihit: SNV MNV INDEL in frame SNV nonsense MNV nonsense INDEL out of frame
Hotspot:

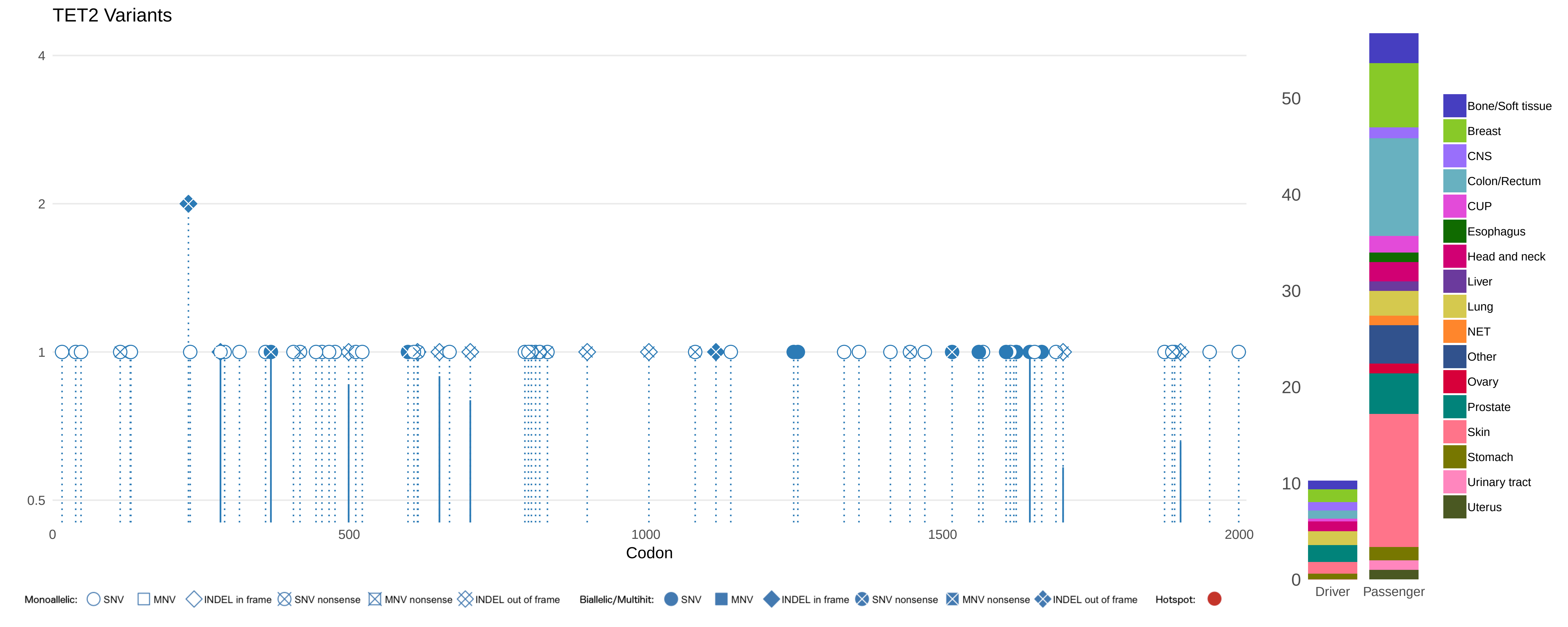
TET2 Variants
4
2
1
0.5
0
500
1000
1500
2000
Codon
50
40
30
20
10
0
Driver
Passenger
Bone/Soft tissue
Breast
CNS
Colon/Rectum
CUP
Esophagus
Head and neck
Liver
Lung
NET
Other
Ovary
Prostate
Skin
Stomach
Urinary tract
Uterus
Monoallelic: SNV MNV INDEL in frame SNV nonsense MNV nonsense INDEL out of frame
Biallelic/Multihit: SNV MNV INDEL in frame SNV nonsense MNV nonsense INDEL out of frame
Hotspot:

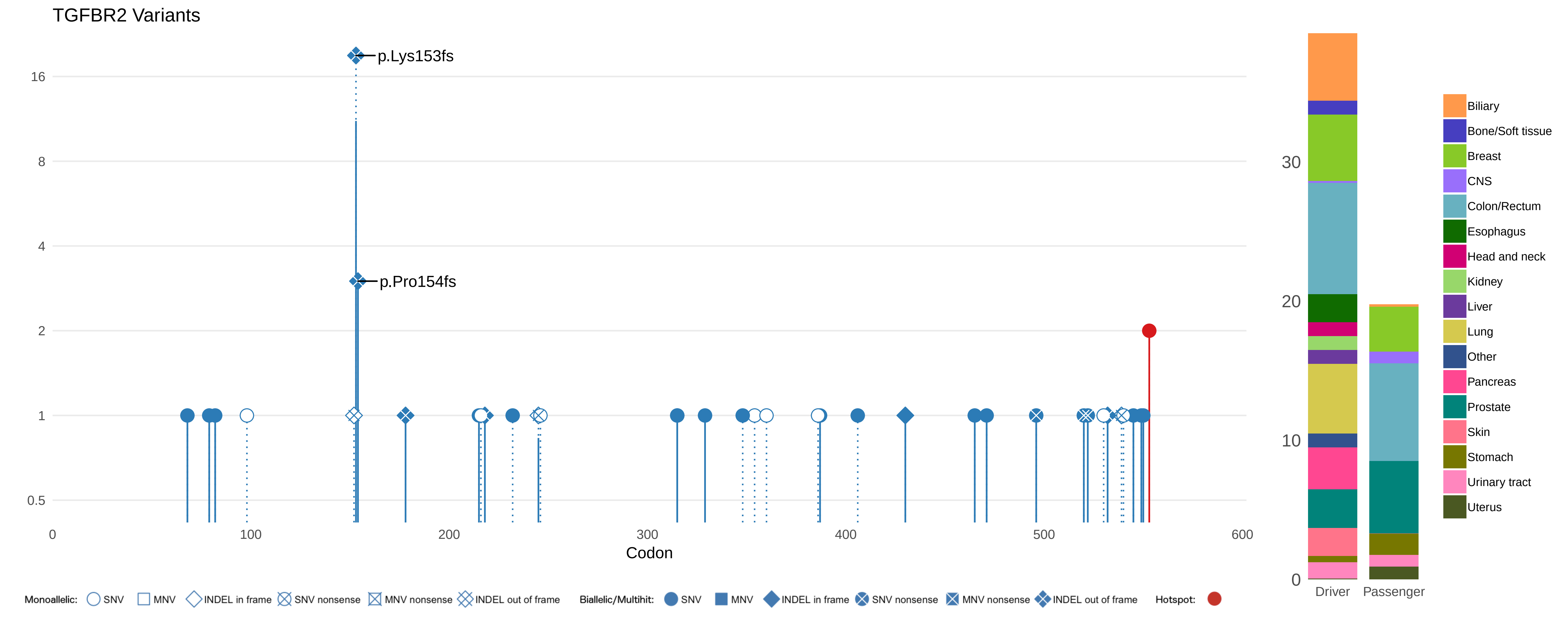

TGFBR2 Variants
p.Lys153fs
p.Pro154fs
16
8
4
2
1
0.5
0
100
200
300
400
500
600
Codon
Monoallelic: SNV MNV INDEL in frame SNV nonsense MNV nonsense INDEL out of frame
Biallelic/Multihit: SNV MNV INDEL in frame SNV nonsense MNV nonsense INDEL out of frame
Hotspot:
30
20
10
0
Driver
Passenger
Biliary
Bone/Soft tissue
Breast
CNS
Colon/Rectum
Esophagus
Head and neck
Kidney
Liver
Lung
Other
Pancreas
Prostate
Skin
Stomach
Urinary tract
Uterus

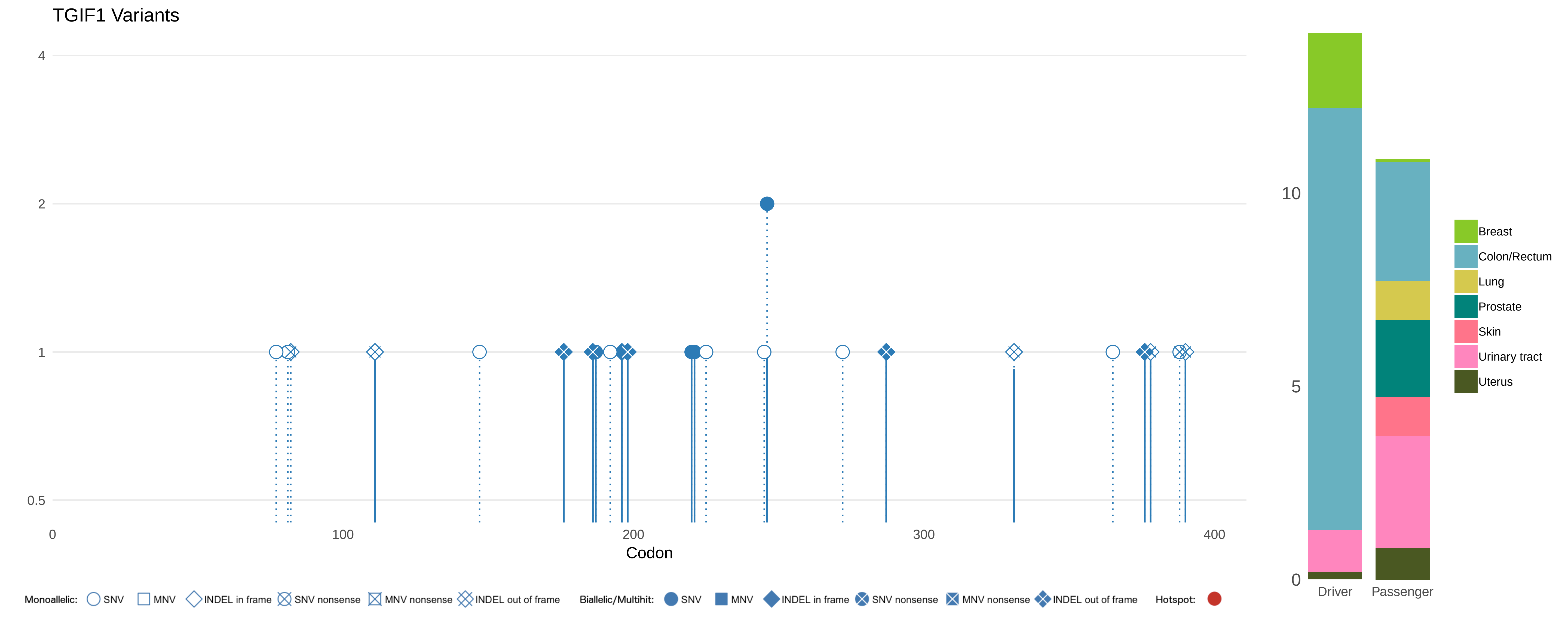
TGIF1 Variants
4
2
1
0.5
0
100
200
300
400
Codon
10
5
0
Driver
Passenger
Breast
Colon/Rectum
Lung
Prostate
Skin
Urinary tract
Uterus
Monoallelic:
SNV
MNV
INDEL in frame
SNV nonsense
MNV nonsense
INDEL out of frame
Biallelic/Multihit:
SNV
MNV
INDEL in frame
SNV nonsense
MNV nonsense
INDEL out of frame
Hotspot:

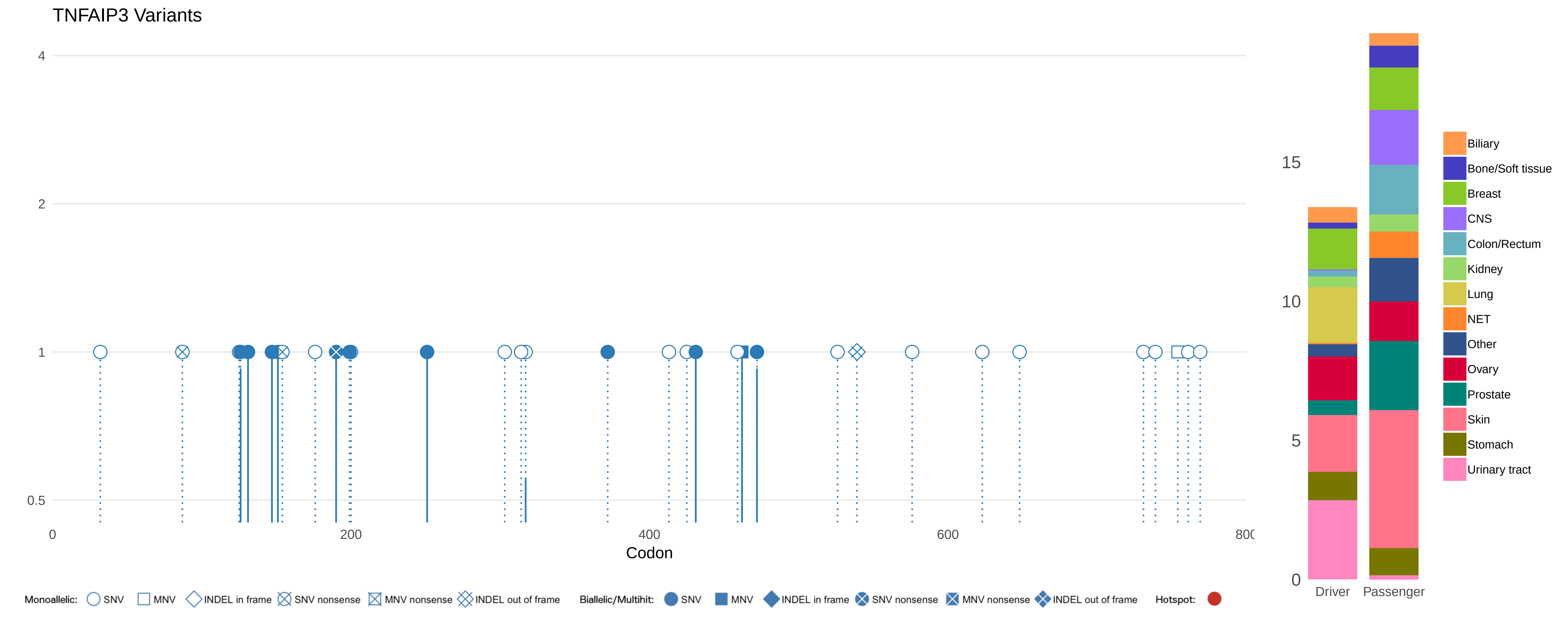

TNFAIP3 Variants
4
2
1
0.5
0
200
400
600
800
Codon
15
10
5
0
Driver
Passenger
Biliary
Bone/Soft tissue
Breast
CNS
Colon/Rectum
Kidney
Lung
NET
Other
Ovary
Prostate
Skin
Stomach
Urinary tract
Monoallelic: SNV MNV INDEL in frame SNV nonsense MNV nonsense INDEL out of frame
Biallelic/Multihit: SNV MNV INDEL in frame SNV nonsense MNV nonsense INDEL out of frame
Hotspot:

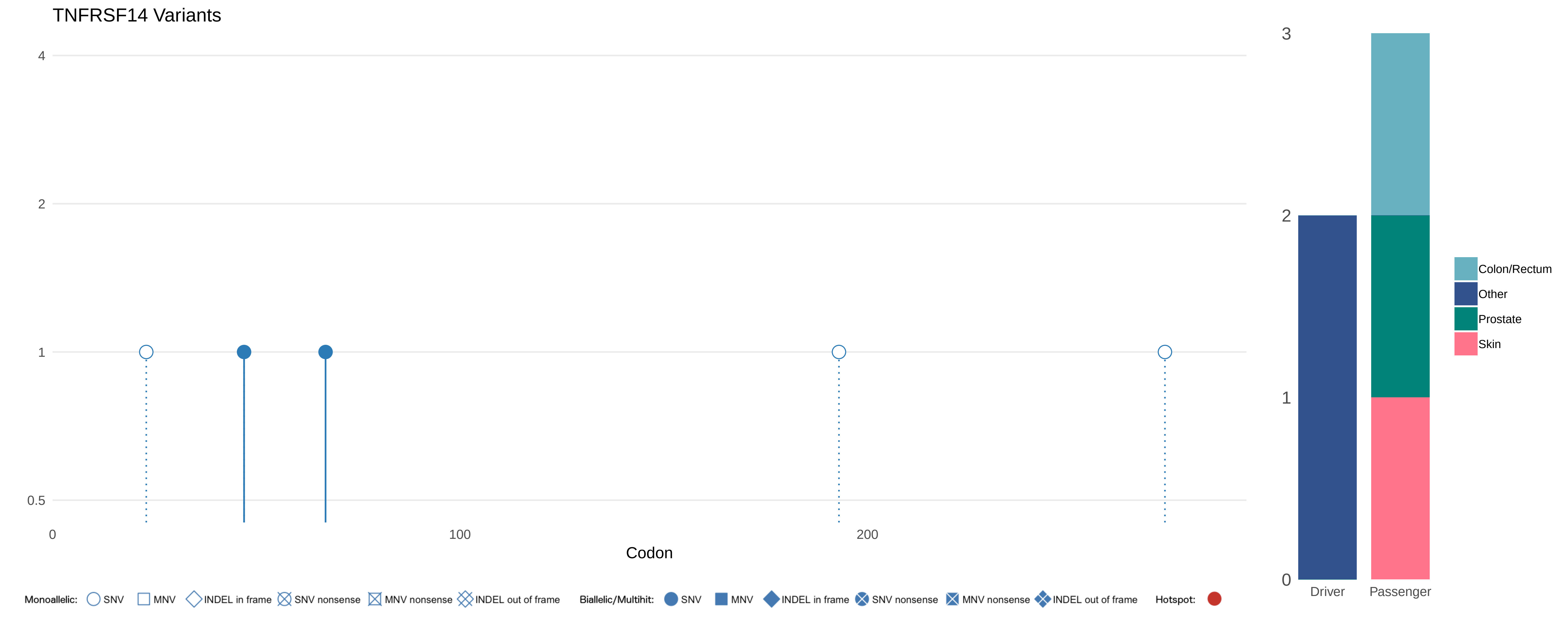
TNFRSF14 Variants
4
2
1
0.5
0
100
200
Codon
Monoallelic: SNV MNV INDEL in frame SNV nonsense MNV nonsense INDEL out of frame
Biallelic/Multihit: SNV MNV INDEL in frame SNV nonsense MNV nonsense INDEL out of frame
Hotspot:
3
2
1
0
Driver
Passenger
Colon/Rectum
Other
Prostate
Skin

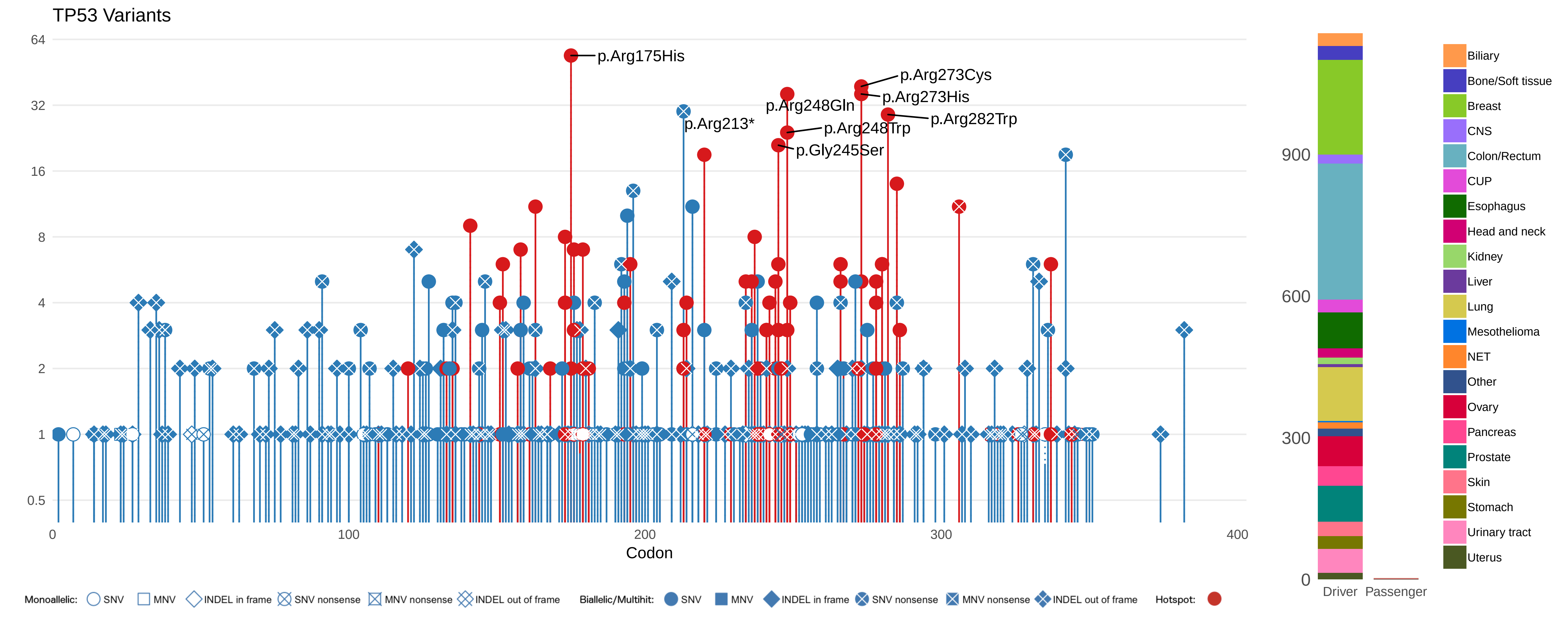

TP53 Variants
64
32
16
8
4
2
1
0.5
0
100
200
300
400
Codon
p.Arg175His
p.Arg273Cys
p.Arg273His
p.Arg248Gln
p.Arg213*
p.Arg282Trp
p.Arg248Trp
p.Gly245Ser
Monoallelic: SNV MNV INDEL in frame SNV nonsense MNV nonsense INDEL out of frame
Biallelic/Multihit: SNV MNV INDEL in frame SNV nonsense MNV nonsense INDEL out of frame
Hotspot:
900
600
300
0
Driver
Passenger
Biliary
Bone/Soft tissue
Breast
CNS
Colon/Rectum
CUP
Esophagus
Head and neck
Kidney
Liver
Lung
Mesothelioma
NET
Other
Ovary
Pancreas
Prostate
Skin
Stomach
Urinary tract
Uterus

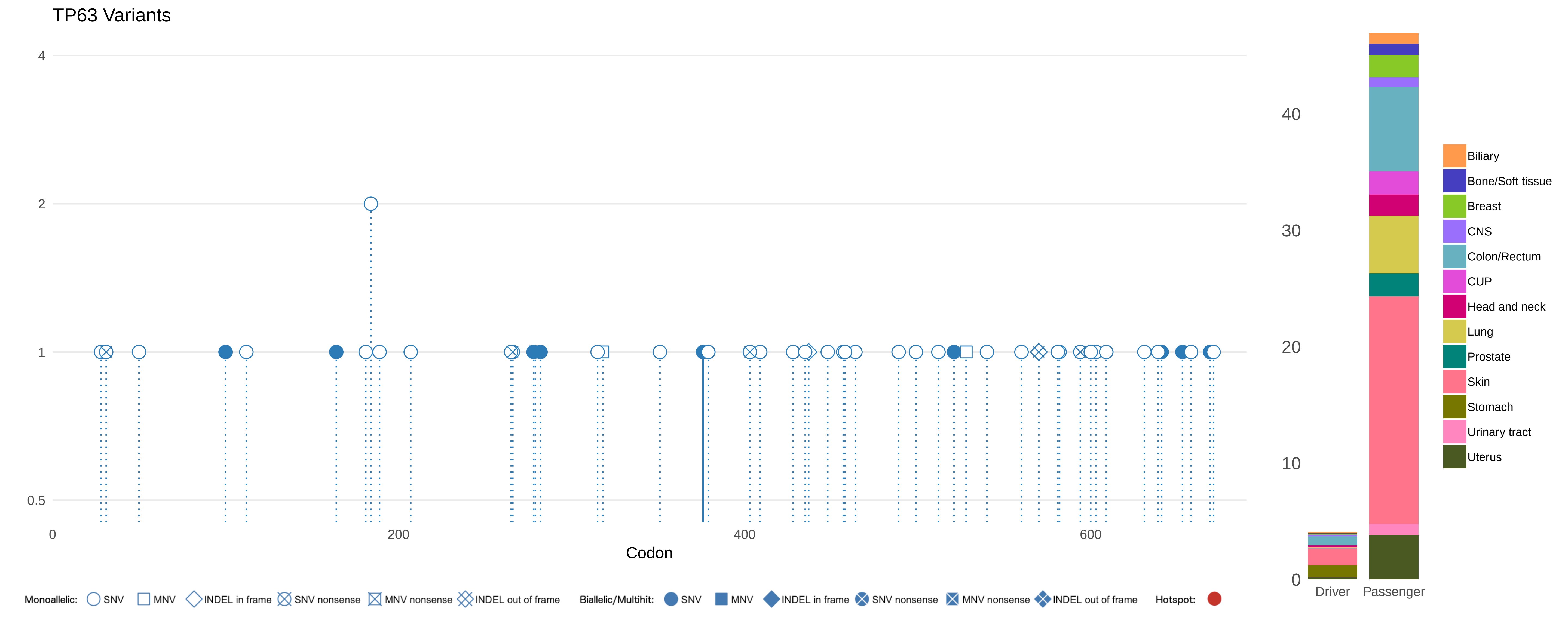
TP63 Variants
4
2
1
0.5
0
200
400
600
Codon
40
30
20
10
0
Driver
Passenger
Biliary
Bone/Soft tissue
Breast
CNS
Colon/Rectum
CUP
Head and neck
Lung
Prostate
Skin
Stomach
Urinary tract
Uterus
Monoallelic: SNV MNV INDEL in frame SNV nonsense MNV nonsense INDEL out of frame
Biallelic/Multihit: SNV MNV INDEL in frame SNV nonsense MNV nonsense INDEL out of frame
Hotspot:

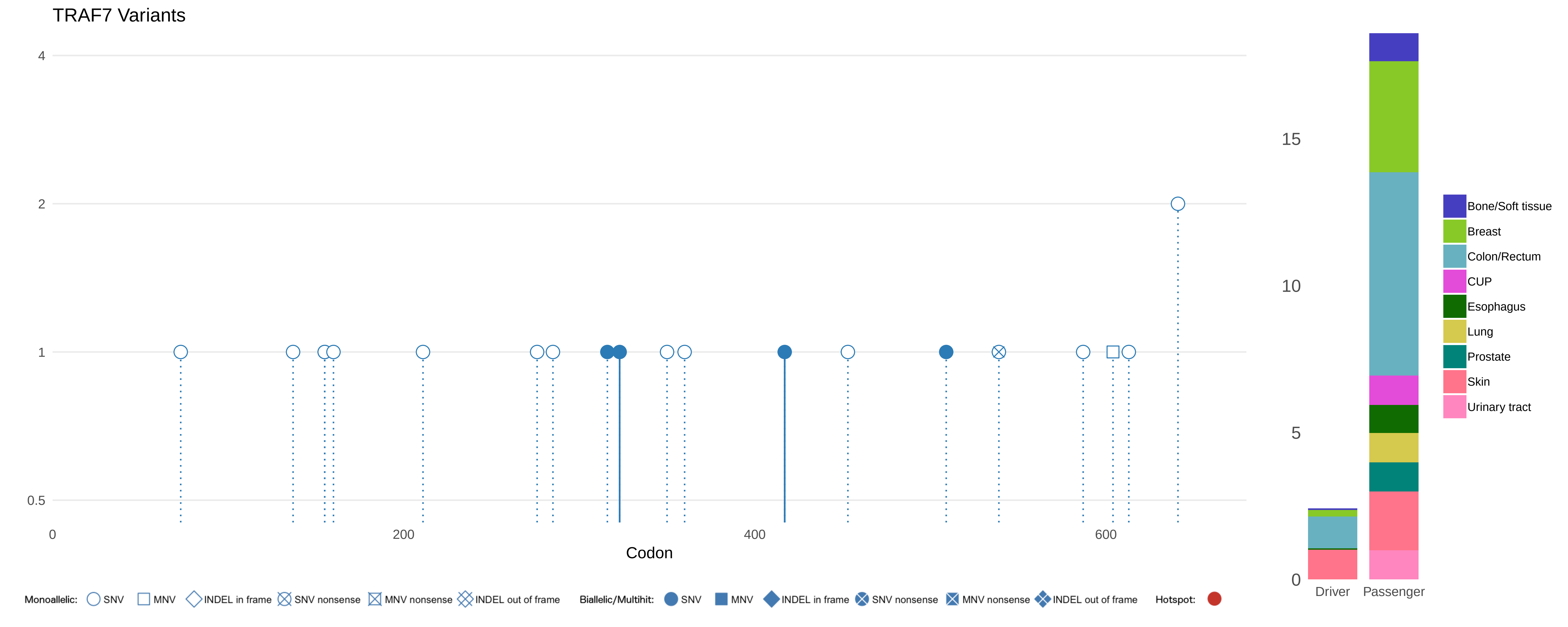
TRAF7 Variants
4
2
1
0.5
0
200
400
600
Codon
15
10
5
0
Driver
Passenger
Bone/Soft tissue
Breast
Colon/Rectum
CUP
Esophagus
Lung
Prostate
Skin
Urinary tract
Monoallelic: SNV MNV INDEL in frame SNV nonsense MNV nonsense INDEL out of frame
Biallelic/Multihit: SNV MNV INDEL in frame SNV nonsense MNV nonsense INDEL out of frame
Hotspot:

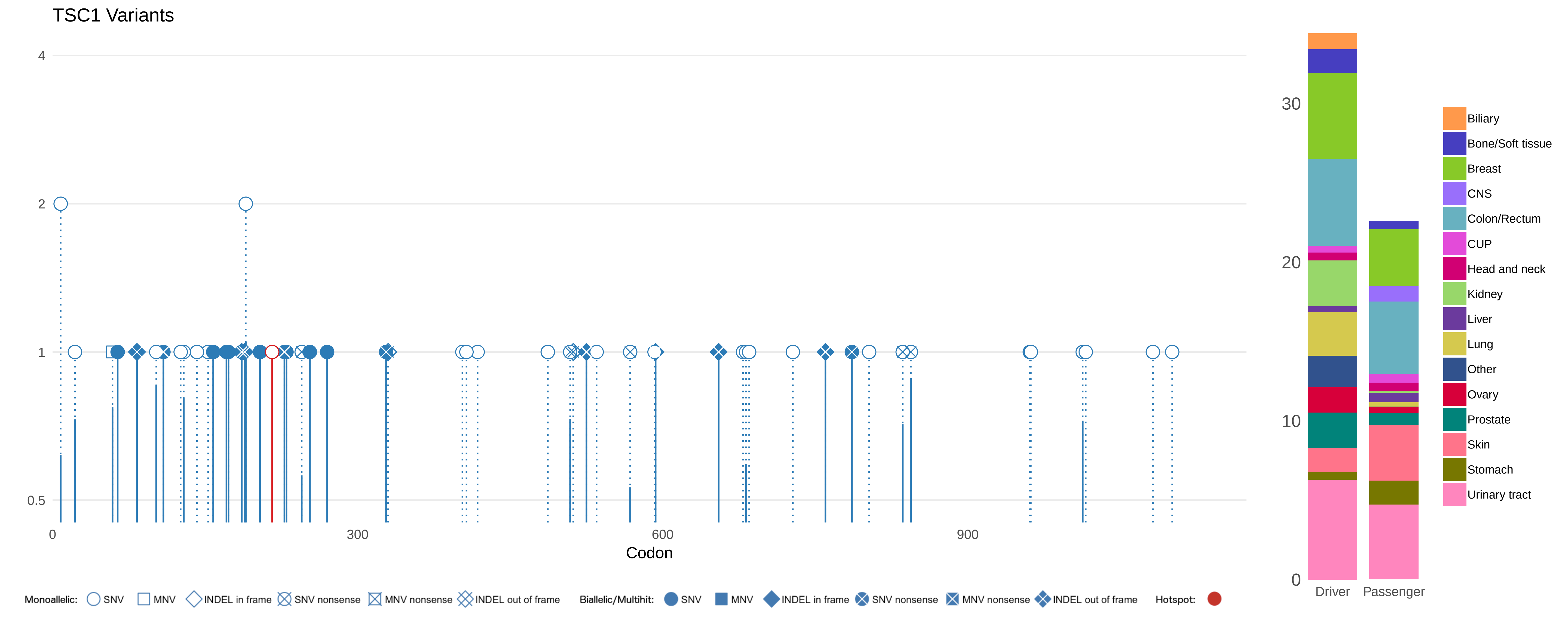
TSC1 Variants
4
2
1
0.5
0
300
600
900
Codon
Monoallelic: SNV MNV INDEL in frame SNV nonsense MNV nonsense INDEL out of frame
Biallelic/Multihit: SNV MNV INDEL in frame SNV nonsense MNV nonsense INDEL out of frame
Hotspot:
30
20
10
0
Driver
Passenger
Biliary
Bone/Soft tissue
Breast
CNS
Colon/Rectum
CUP
Head and neck
Kidney
Liver
Lung
Other
Ovary
Prostate
Skin
Stomach
Urinary tract

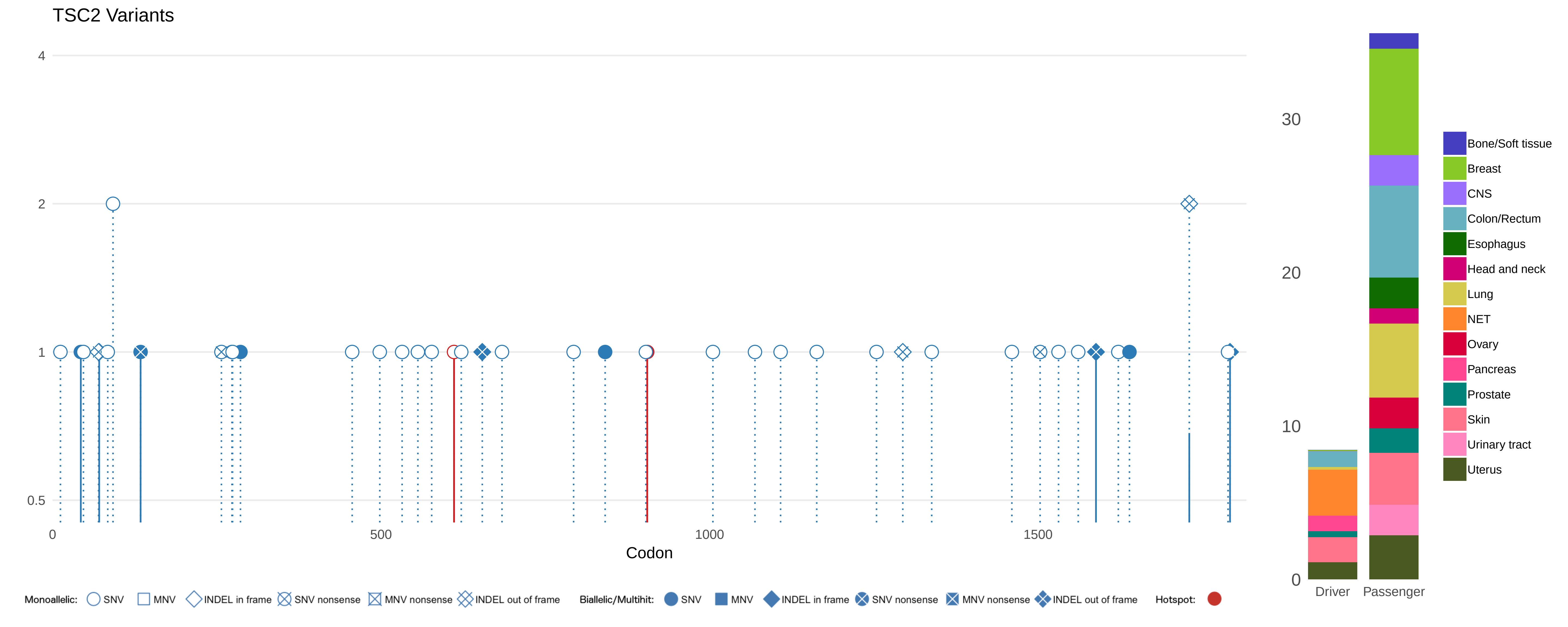

TSC2 Variants
4
2
1
0.5
0
500
1000
1500
Codon
30
20
10
0
Driver
Passenger
Bone/Soft tissue
Breast
CNS
Colon/Rectum
Esophagus
Head and neck
Lung
NET
Ovary
Pancreas
Prostate
Skin
Urinary tract
Uterus
Monoallelic: SNV MNV INDEL in frame SNV nonsense MNV nonsense INDEL out of frame
Biallelic/Multihit: SNV MNV INDEL in frame SNV nonsense MNV nonsense INDEL out of frame
Hotspot:

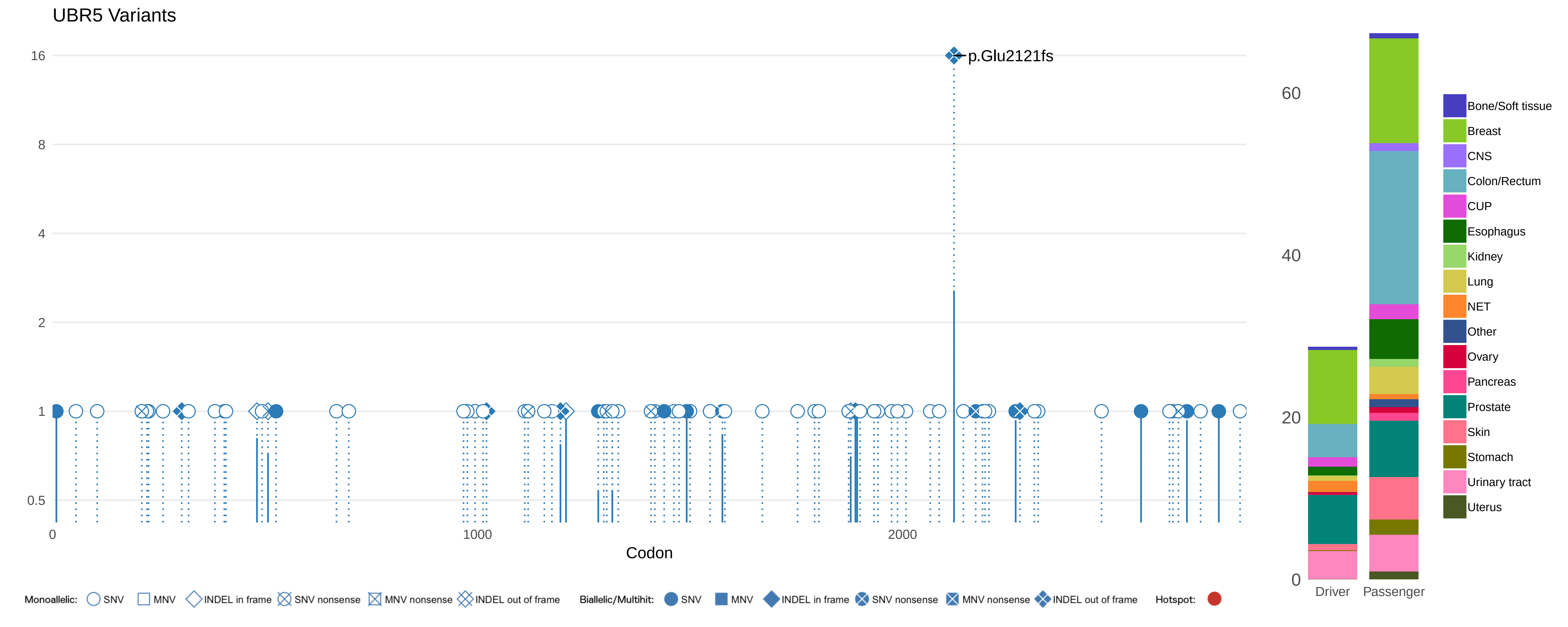
UBR5 Variants
16
8
4
2
1
0.5
p.Glu2121fs
0
1000
2000
Codon
60
40
20
0
Driver
Passenger
Bone/Soft tissue
Breast
CNS
Colon/Rectum
CUP
Esophagus
Kidney
Lung
NET
Other
Ovary
Pancreas
Prostate
Skin
Stomach
Urinary tract
Uterus
Monoallelic: SNV MNV INDEL in frame SNV nonsense MNV nonsense INDEL out of frame
Biallelic/Multihit: SNV MNV INDEL in frame SNV nonsense MNV nonsense INDEL out of frame
Hotspot:

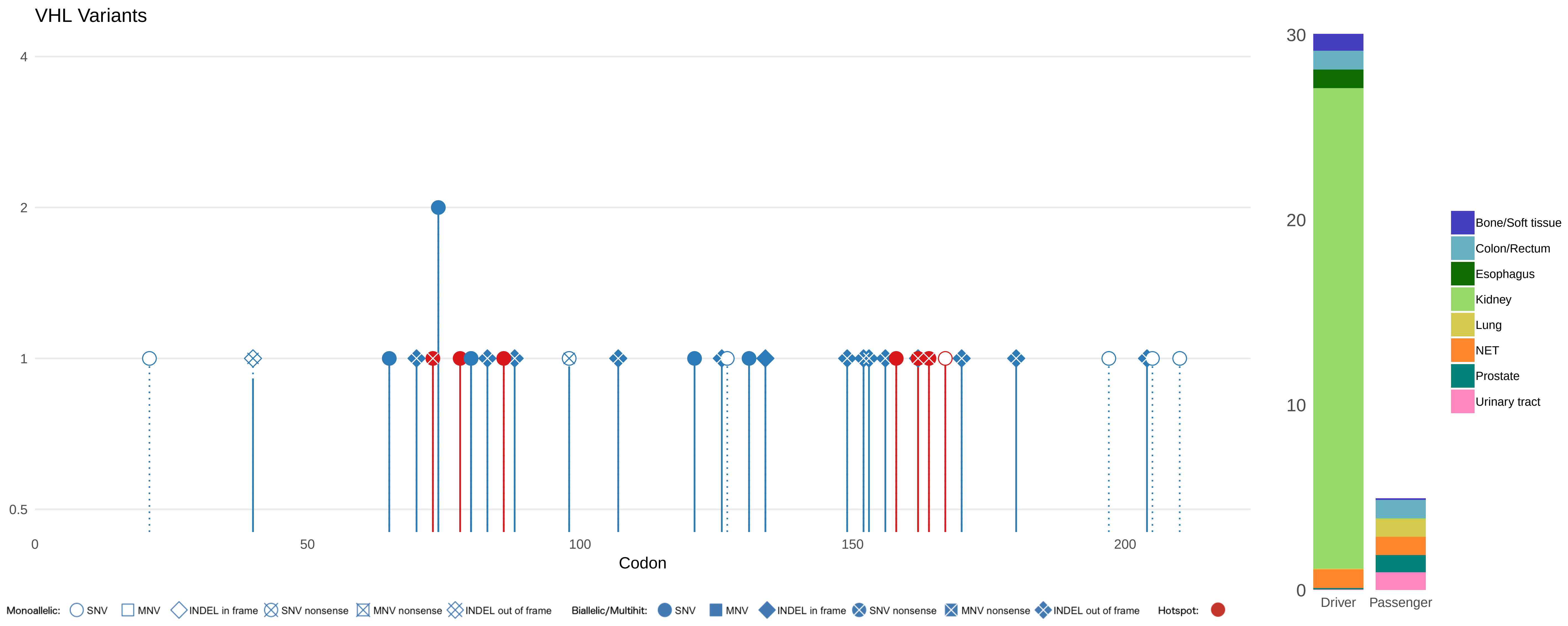
VHL Variants
4
2
1
0.5
0
50
100
150
200
Codon
Monoallelic: SNV MNV INDEL in frame SNV nonsense MNV nonsense INDEL out of frame
Biallelic/Multihit: SNV MNV INDEL in frame SNV nonsense MNV nonsense INDEL out of frame
Hotspot:
30
20
10
0
Driver
Passenger
Bone/Soft tissue
Colon/Rectum
Esophagus
Kidney
Lung
NET
Prostate
Urinary tract

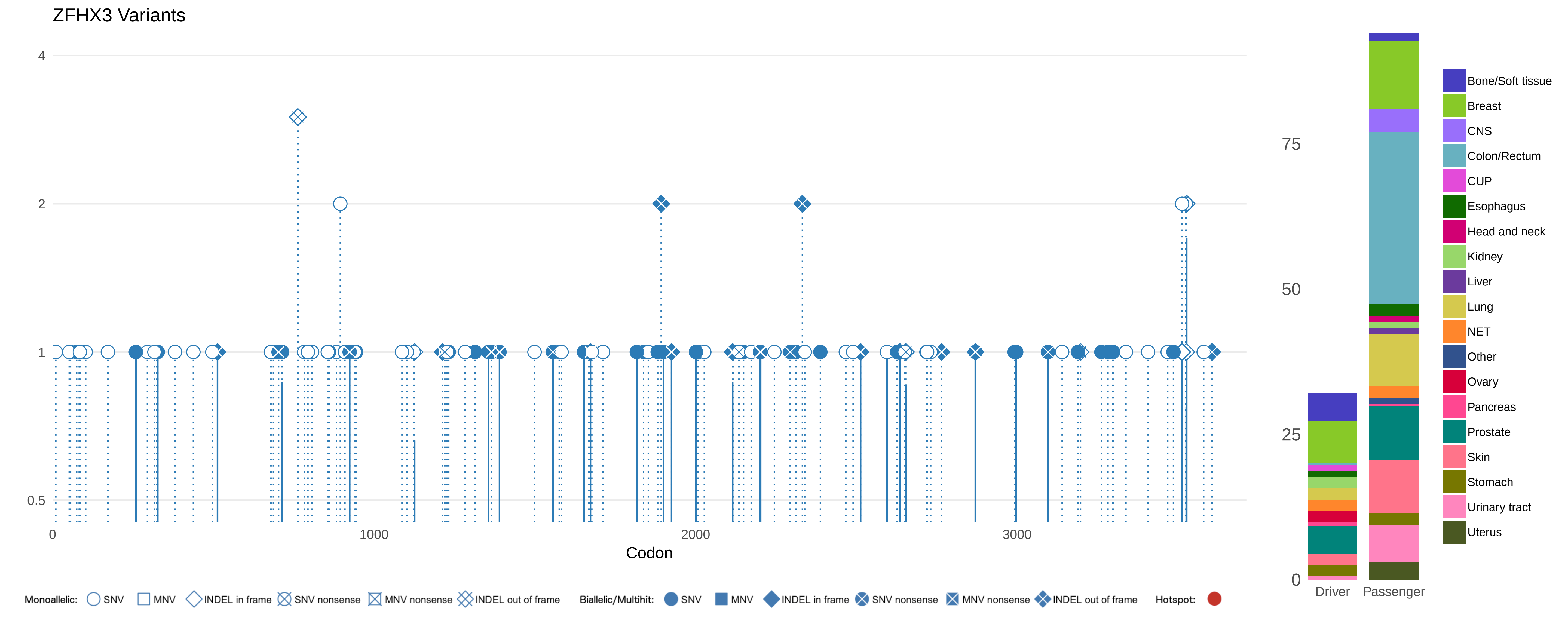
ZFHX3 Variants
4
2
1
0.5
0
1000
2000
3000
Codon
75
50
25
0
Driver
Passenger
Bone/Soft tissue
Breast
CNS
Colon/Rectum
CUP
Esophagus
Head and neck
Kidney
Liver
Lung
NET
Other
Ovary
Pancreas
Prostate
Skin
Stomach
Urinary tract
Uterus
Monoallelic: SNV MNV INDEL in frame SNV nonsense MNV nonsense INDEL out of frame
Biallelic/Multihit: SNV MNV INDEL in frame SNV nonsense MNV nonsense INDEL out of frame
Hotspot:

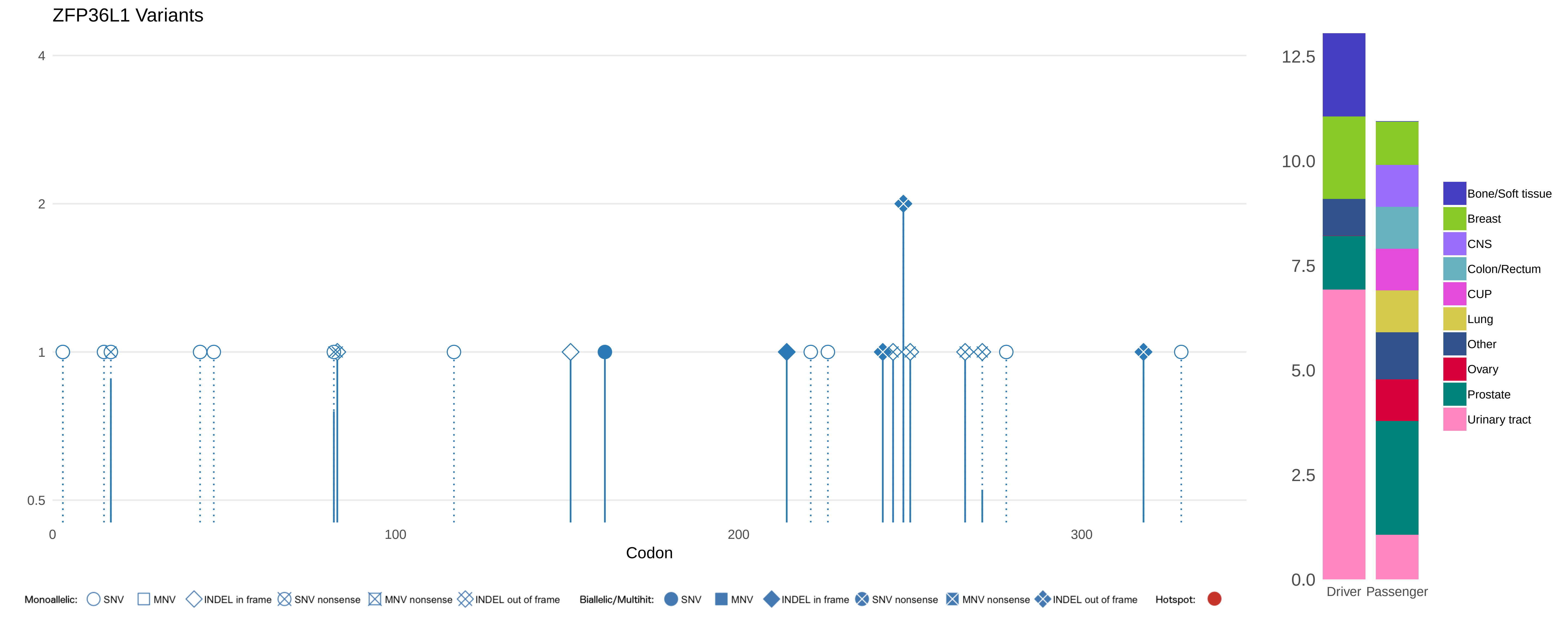

ZFP36L1 Variants
4
2
1
0.5
0
100
200
300
Codon
12.5
10.0
7.5
5.0
2.5
0.0
Driver
Passenger
Bone/Soft tissue
Breast
CNS
Colon/Rectum
CUP
Lung
Other
Ovary
Prostate
Urinary tract
Monoallelic:
SNV
MNV
INDEL in frame
SNV nonsense
MNV nonsense
INDEL out of frame
Biallelic/Multihit:
SNV
MNV
INDEL in frame
SNV nonsense
MNV nonsense
INDEL out of frame
Hotspot:

ZFP36L2 Variants

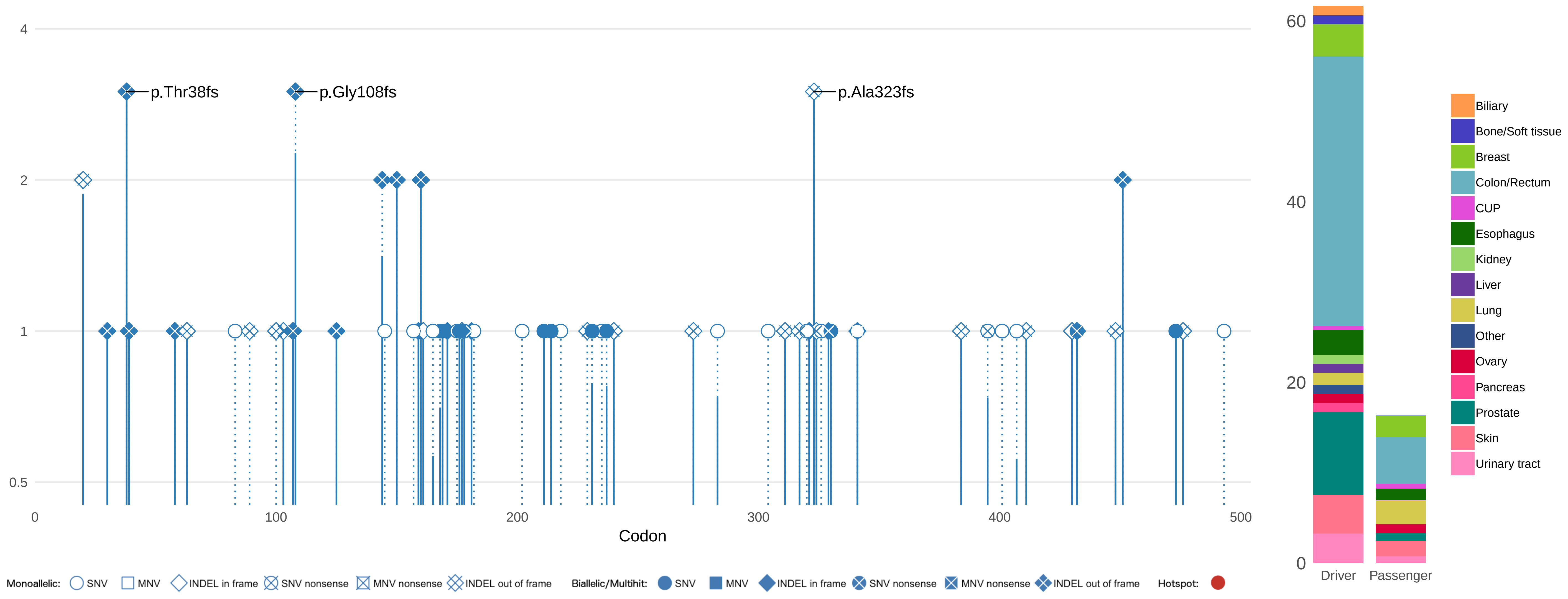

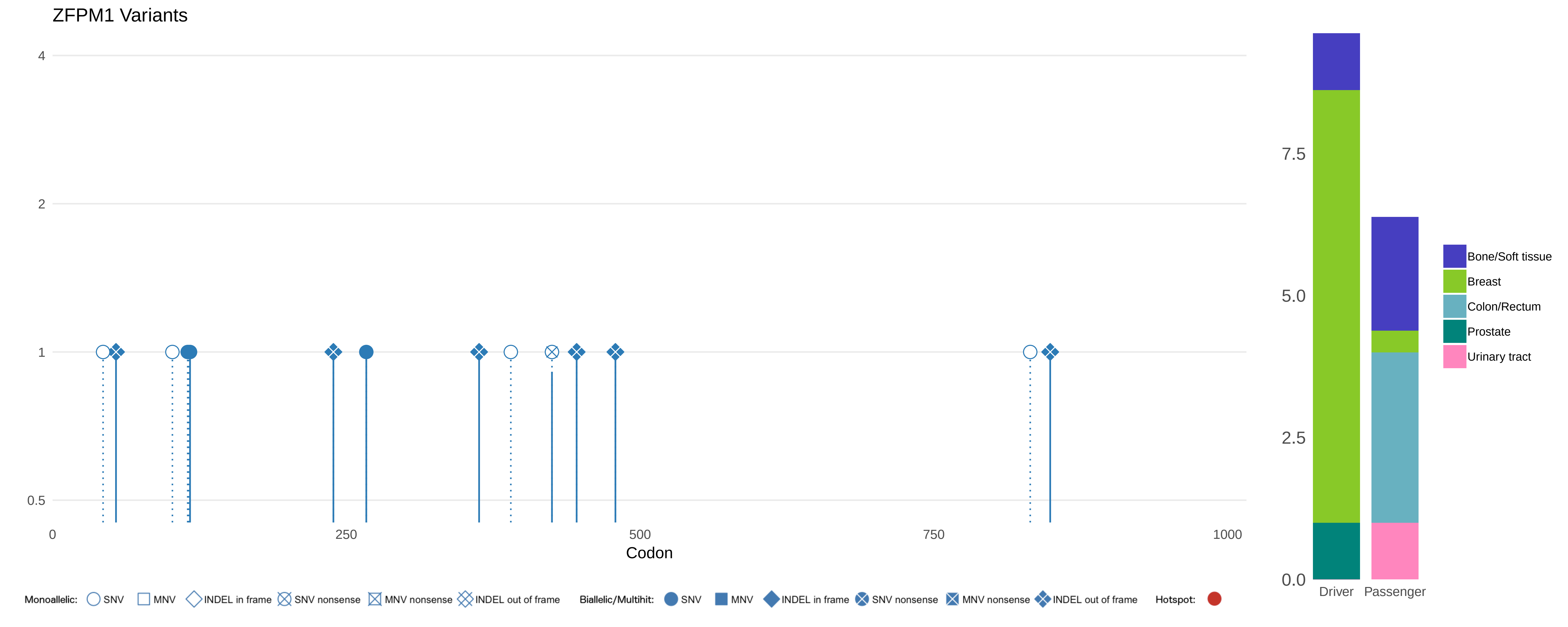
ZFPM1 Variants
4
2
1
0.5
0
250
500
750
1000
Codon
7.5
5.0
2.5
0.0
Driver
Passenger
Bone/Soft tissue
Breast
Colon/Rectum
Prostate
Urinary tract
Monoallelic:
SNV
MNV
INDEL in frame
SNV nonsense
MNV nonsense
INDEL out of frame
Biallelic/Multihit:
SNV
MNV
INDEL in frame
SNV nonsense
MNV nonsense
INDEL out of frame
Hotspot:

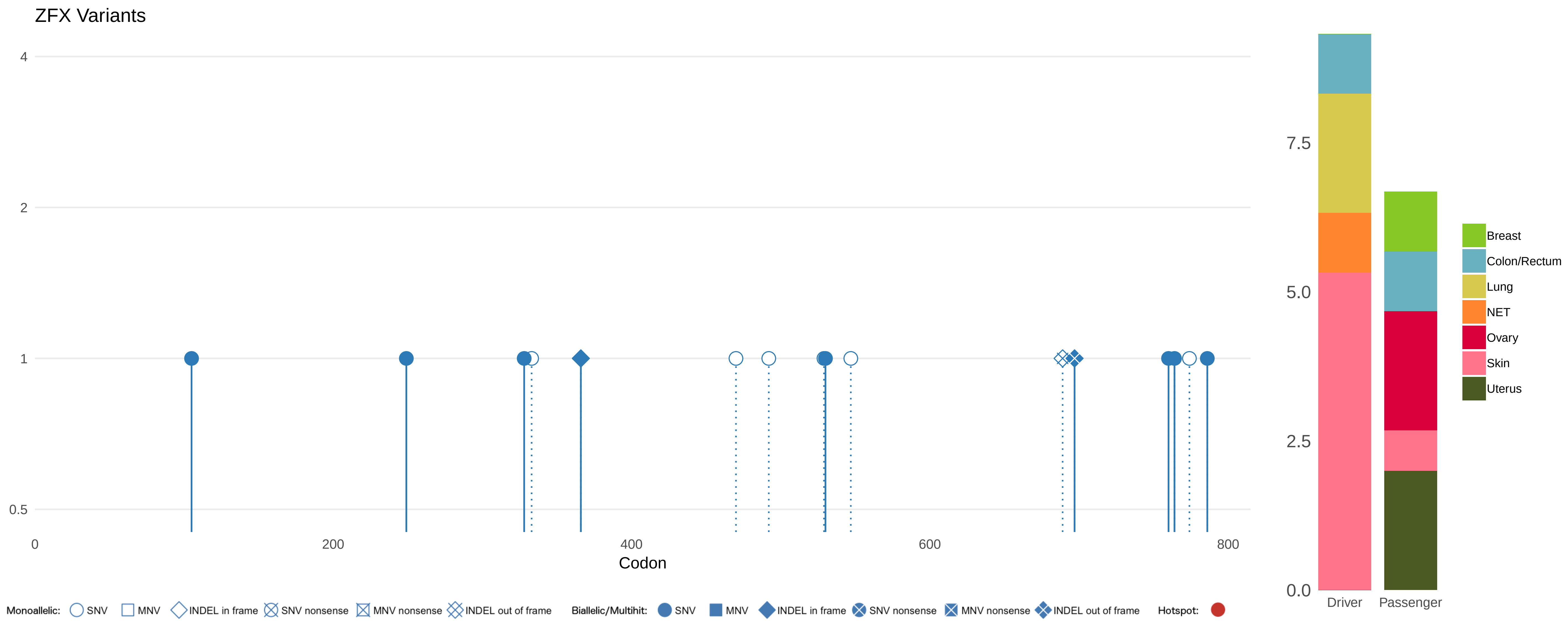

ZFX Variants
4
2
1
0.5
0
200
400
600
800
Codon
7.5
5.0
2.5
0.0
Driver
Passenger
Breast
Colon/Rectum
Lung
NET
Ovary
Skin
Uterus
Monoallelic: SNV MNV INDEL in frame SNV nonsense MNV nonsense INDEL out of frame
Biallelic/Multihit: SNV MNV INDEL in frame SNV nonsense MNV nonsense INDEL out of frame
Hotspot:

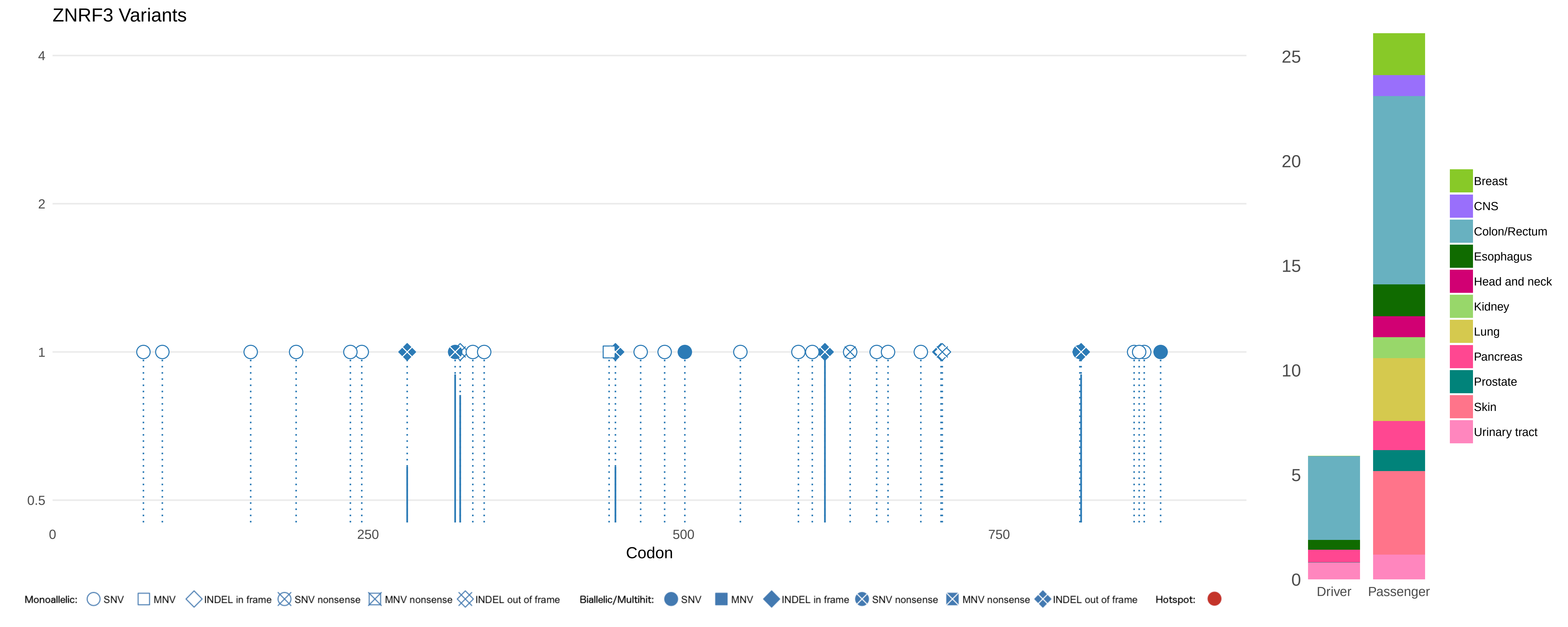
ZNRF3 Variants
4
2
1
0.5
0
250
500
750
Codon
25
20
15
10
5
0
Driver
Passenger
Breast
CNS
Colon/Rectum
Esophagus
Head and neck
Kidney
Lung
Pancreas
Prostate
Skin
Urinary tract
Monoallelic: SNV MNV INDEL in frame SNV nonsense MNV nonsense INDEL out of frame
Biallelic/Multihit: SNV MNV INDEL in frame SNV nonsense MNV nonsense INDEL out of frame
Hotspot:

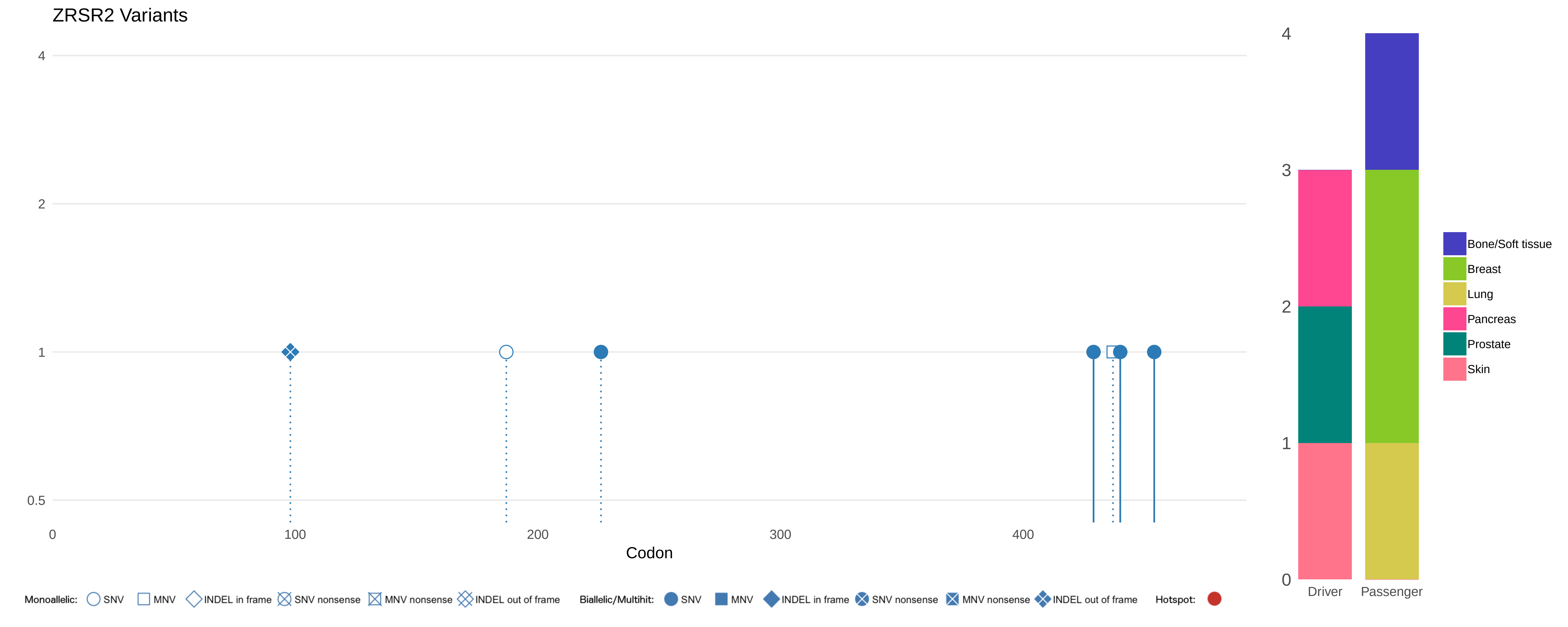
ZRSR2 Variants
4
2
1
0.5
0
100
200
300
400
Codon
Monoallelic: SNV MNV INDEL in frame SNV nonsense MNV nonsense INDEL out of frame
Biallelic/Multihit: SNV MNV INDEL in frame SNV nonsense MNV nonsense INDEL out of frame
Hotspot:
4
3
2
1
0
Driver
Passenger
Bone/Soft tissue
Breast
Lung
Pancreas
Prostate
Skin
