# Supplementary material for: Pan-cancer whole-genome analyses of metastatic solid tumours
Source: Nature. 2019 Oct 23;575(7781):210–6. doi: 10.1038/s41586-019-1689-y (PMC6872491; doi:10.1038/s41586-019-1689-y)
Supplement: Supplementary file 5 — Supplementary Figure 3.Coding mutation profiles by oncogene driver gene. Location and driver classification of all coding mutations (SNVs and indels) in oncogenes (a) and tumour suppressor genes (TSG) (b) in the driver catalogue. The lollipops on the chart show the location (coding sequence coordinates) and count of mutations for all candidate drivers. The height of lollipop represents the total count of each individual variant in the cohort (log scale). The height of the solid line represents the sum of driver likelihoods for that variant, i.e. the proportion that are expected to be drivers. (Partially) dotted lines hence indicate variants for which driver role is uncertain. The right column chart shows the stimated number of drivers (calculated as the sum of driver likelihoods) and passenger variants in each gene by cancer type [file 41586_2019_1689_MOESM5_ESM.pdf]

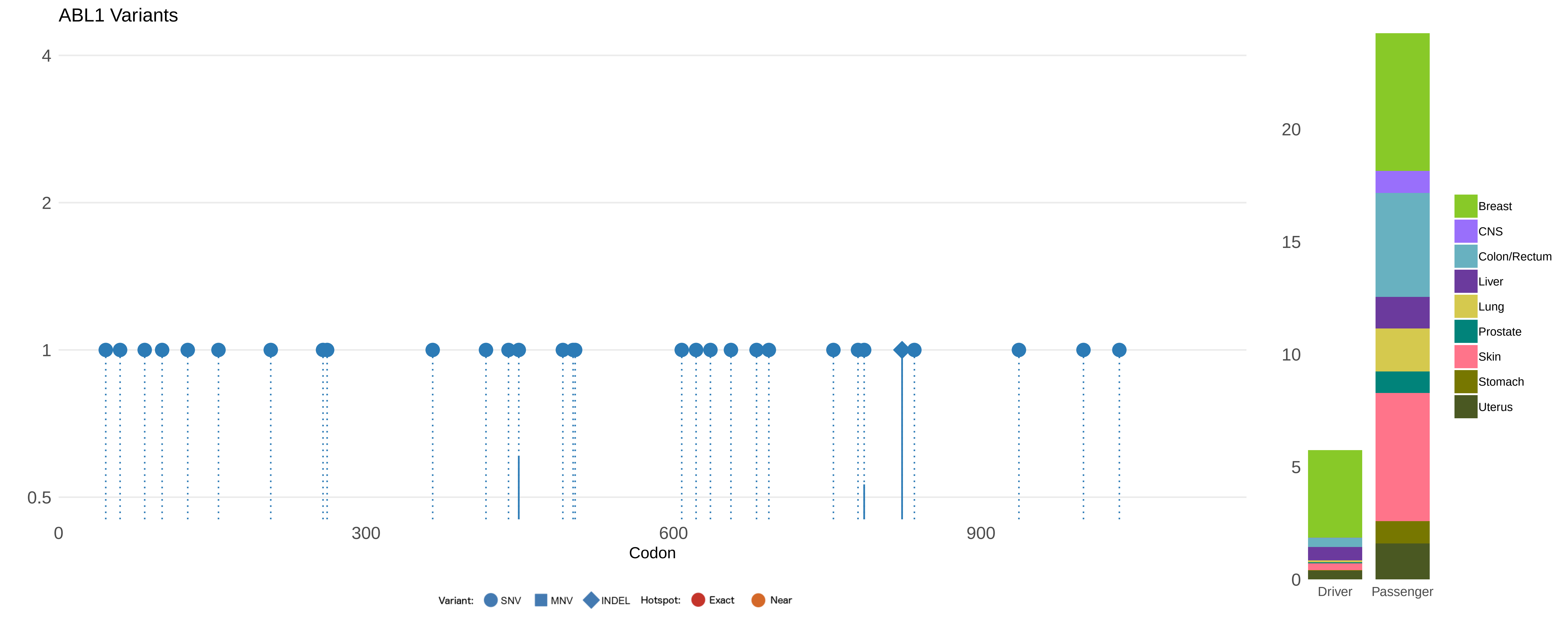

ABL1 Variants
4
2
1
0.5
0
300
600
900
Codon
Variant:
SNV
MNV
INDEL
Hotspot:
Exact
Near
20
15
10
5
0
Driver
Passenger
Breast
CNS
Colon/Rectum
Liver
Lung
Prostate
Skin
Stomach
Uterus

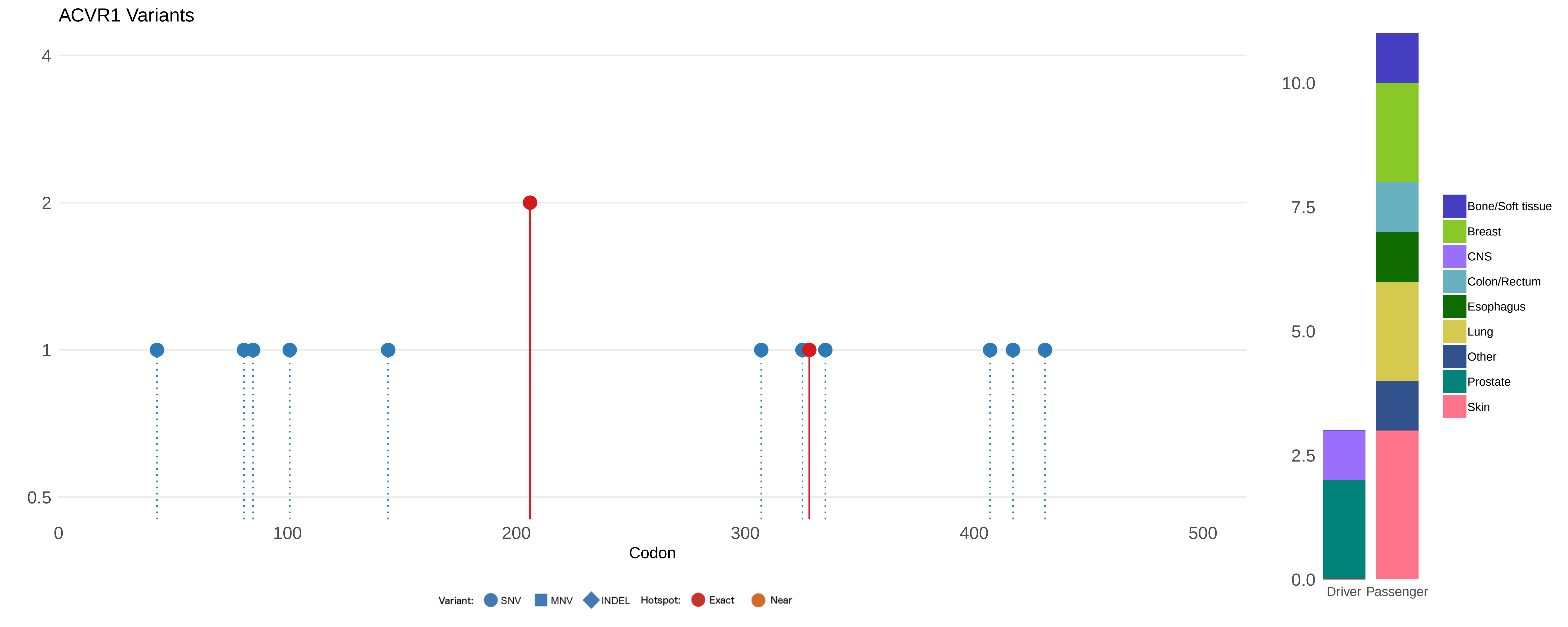

ACVR1 Variants
4
2
1
0.5
0
100
200
300
400
500
Codon
Variant: SNV MNV INDEL Hotspot: Exact Near
10.0
7.5
5.0
2.5
0.0
Driver
Passenger
Bone/Soft tissue
Breast
CNS
Colon/Rectum
Esophagus
Lung
Other
Prostate
Skin

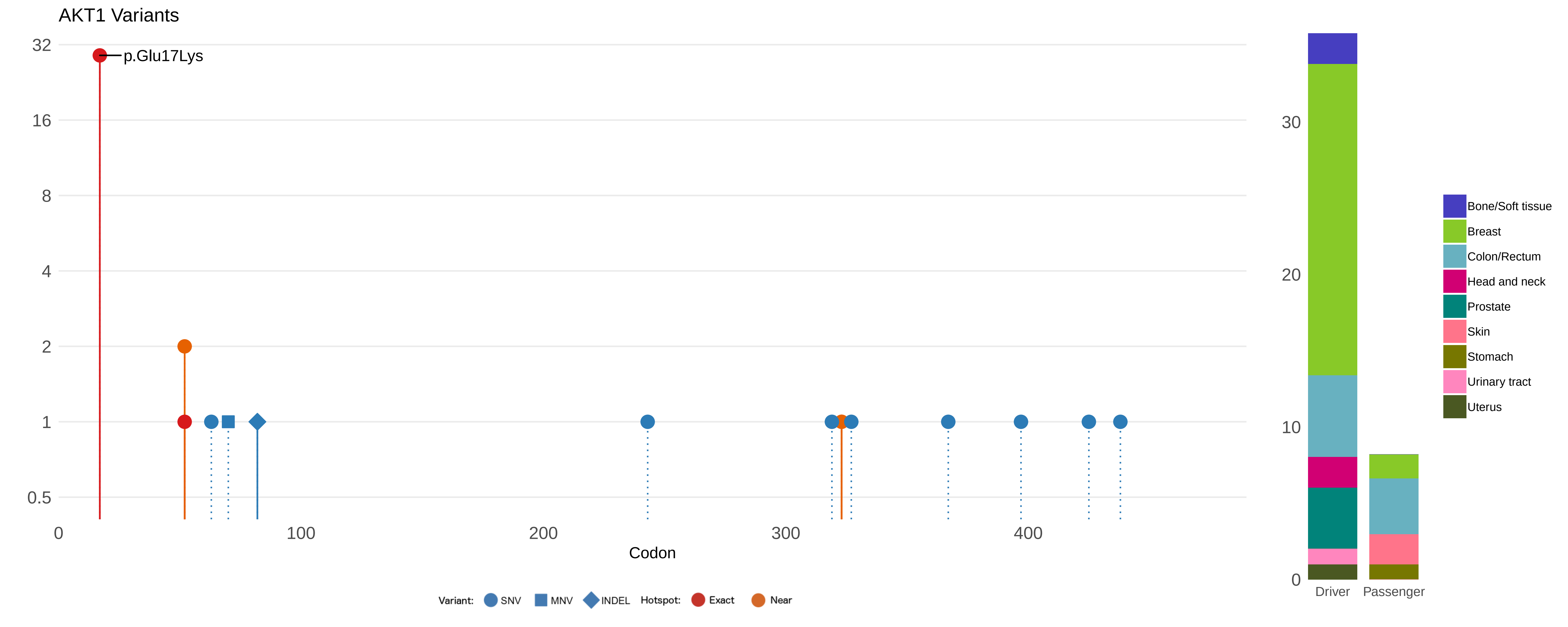

AKT1 Variants
32
16
8
4
2
1
0.5
p.Glu17Lys
0
100
200
300
400
Codon
Variant:
SNV
MNV
INDEL
Hotspot:
Exact
Near
30
20
10
0
Driver
Passenger
Bone/Soft tissue
Breast
Colon/Rectum
Head and neck
Prostate
Skin
Stomach
Urinary tract
Uterus

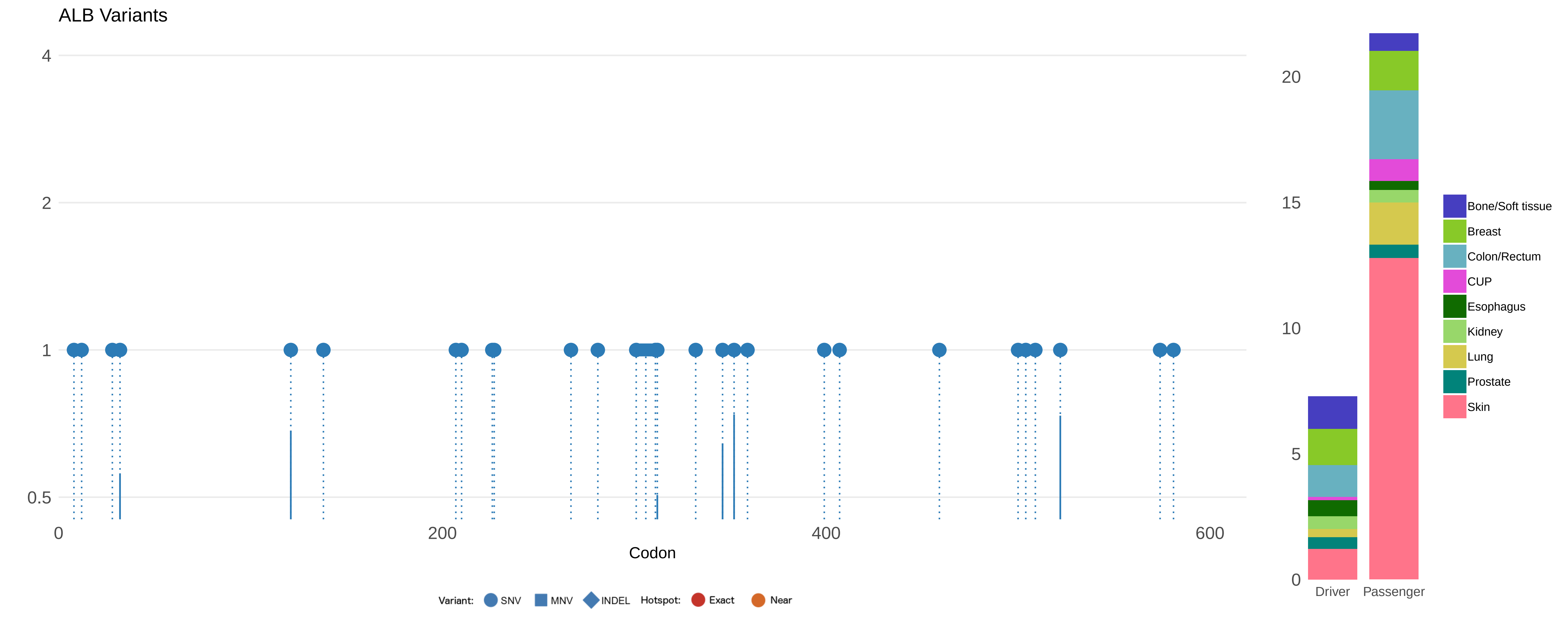

ALB Variants
4
2
1
0.5
0
200
400
600
Codon
Variant:
SNV
MNV
INDEL
Hotspot:
Exact
Near
20
15
10
5
0
Driver
Passenger
Bone/Soft tissue
Breast
Colon/Rectum
CUP
Esophagus
Kidney
Lung
Prostate
Skin

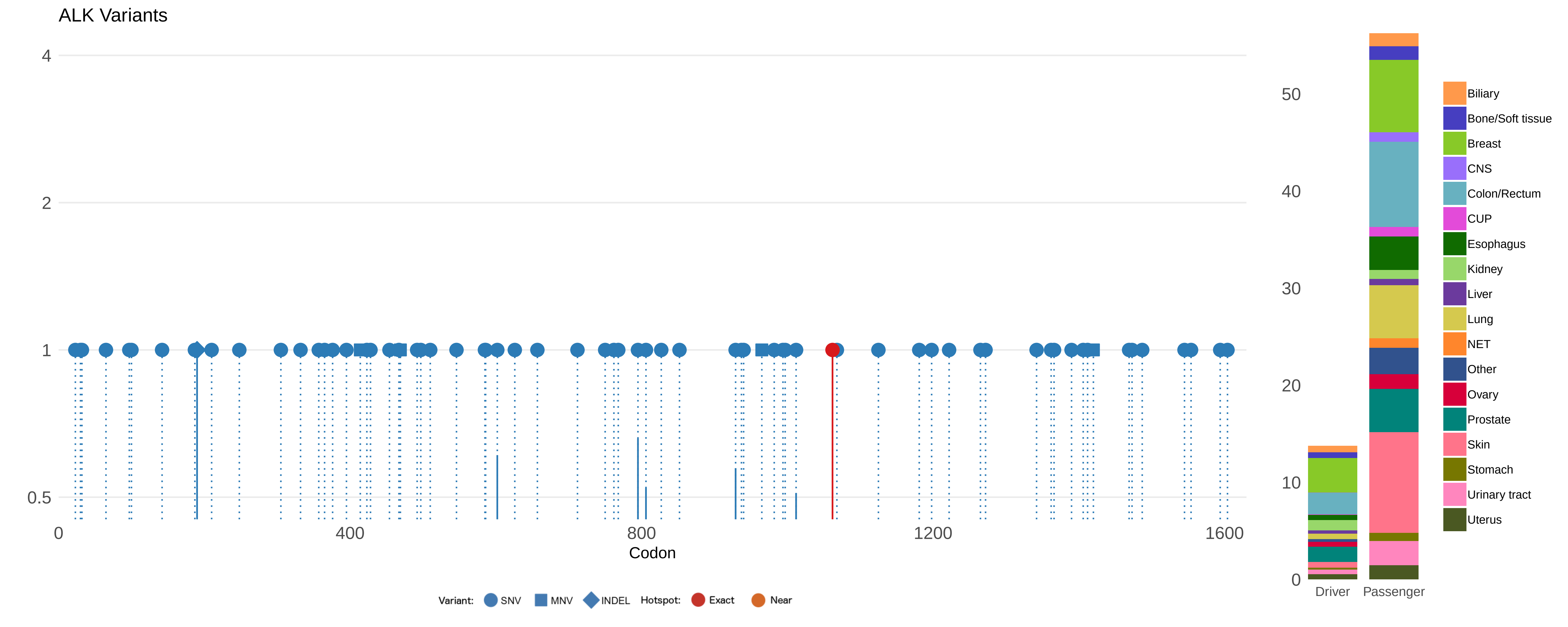

ALK Variants
4
2
1
0.5
0
400
800
1200
1600
Codon
Variant: SNV MNV INDEL Hotspot: Exact Near
50
40
30
20
10
0
Driver
Passenger
Biliary
Bone/Soft tissue
Breast
CNS
Colon/Rectum
CUP
Esophagus
Kidney
Liver
Lung
NET
Other
Ovary
Prostate
Skin
Stomach
Urinary tract
Uterus

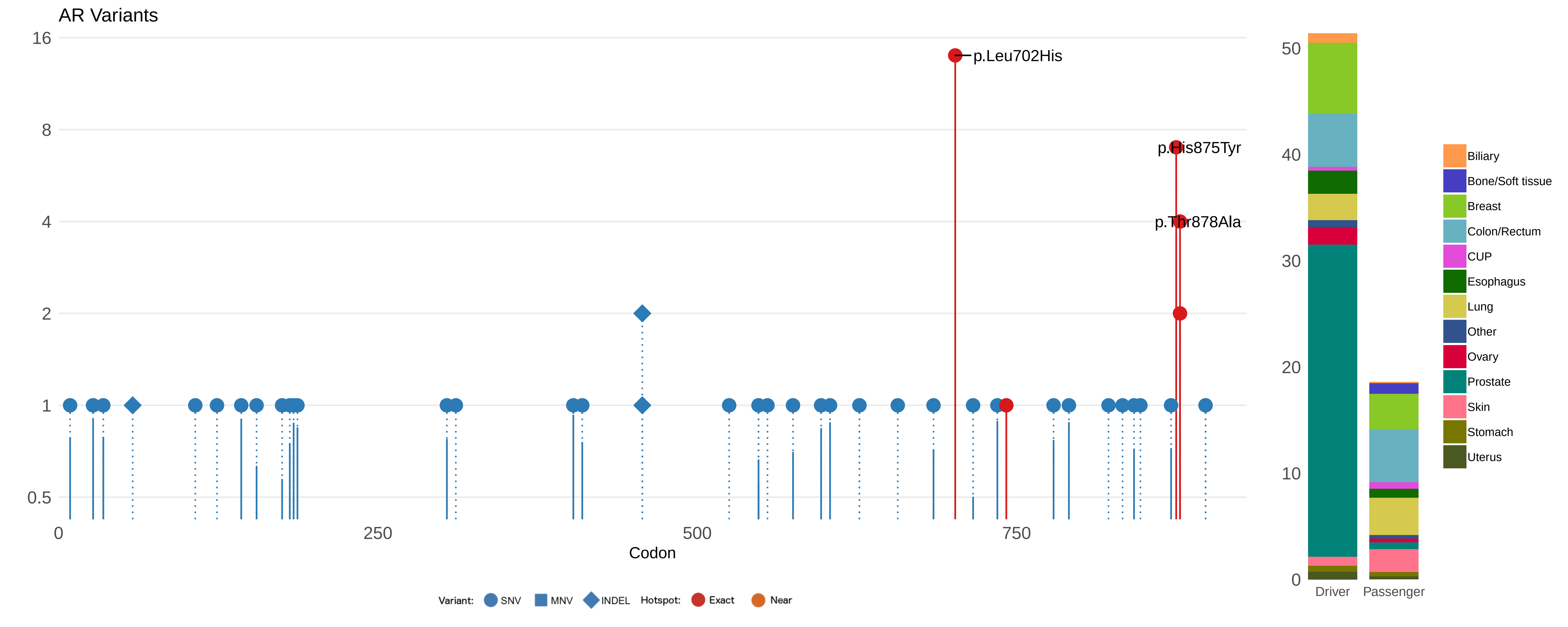
AR Variants
16
8
4
2
1
0.5
0
250
500
750
Codon
p.Leu702His
p.His875Tyr
p.Thr878Ala
Variant: SNV MNV INDEL Hotspot: Exact Near
50
40
30
20
10
0
Driver
Passenger
Biliary
Bone/Soft tissue
Breast
Colon/Rectum
CUP
Esophagus
Lung
Other
Ovary
Prostate
Skin
Stomach
Uterus

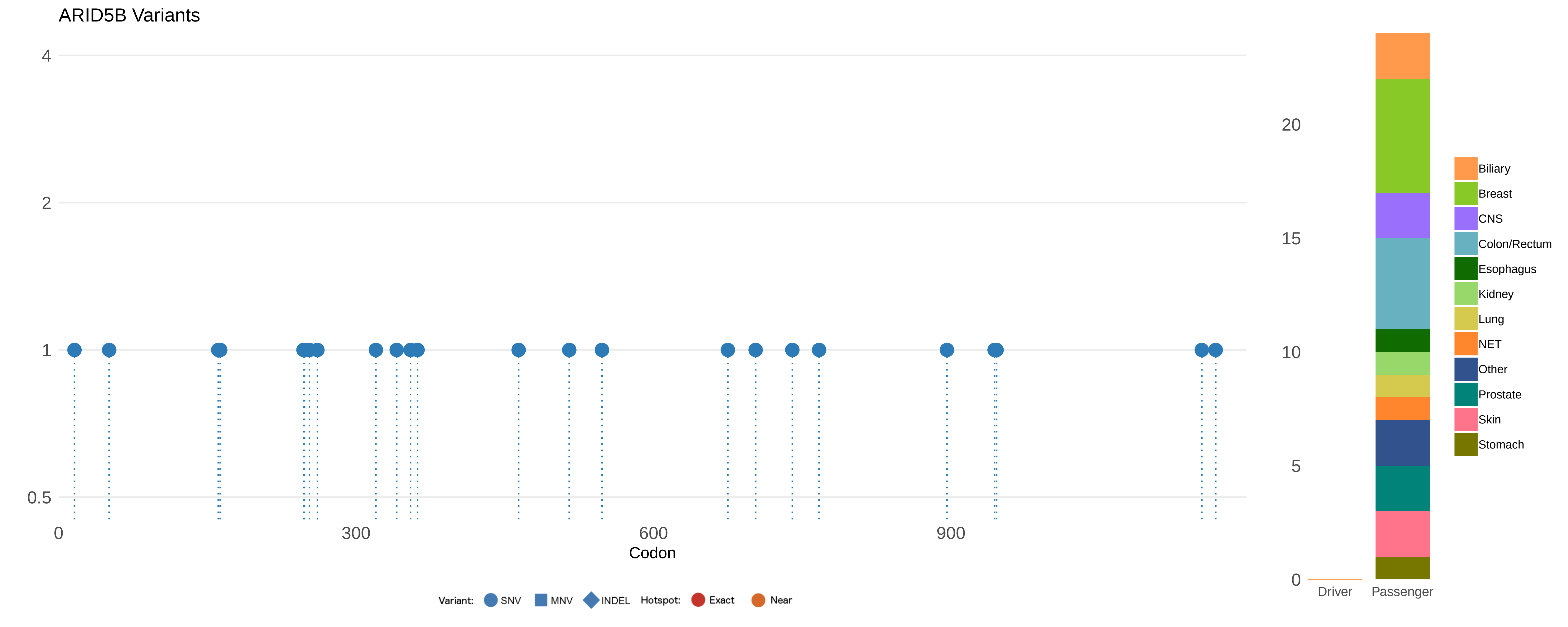

ARID5B Variants
4
2
1
0.5
0
300
600
900
Codon
Variant: SNV MNV INDEL Hotspot: Exact Near
20
15
10
5
0
Driver
Passenger
Biliary
Breast
CNS
Colon/Rectum
Esophagus
Kidney
Lung
NET
Other
Prostate
Skin
Stomach

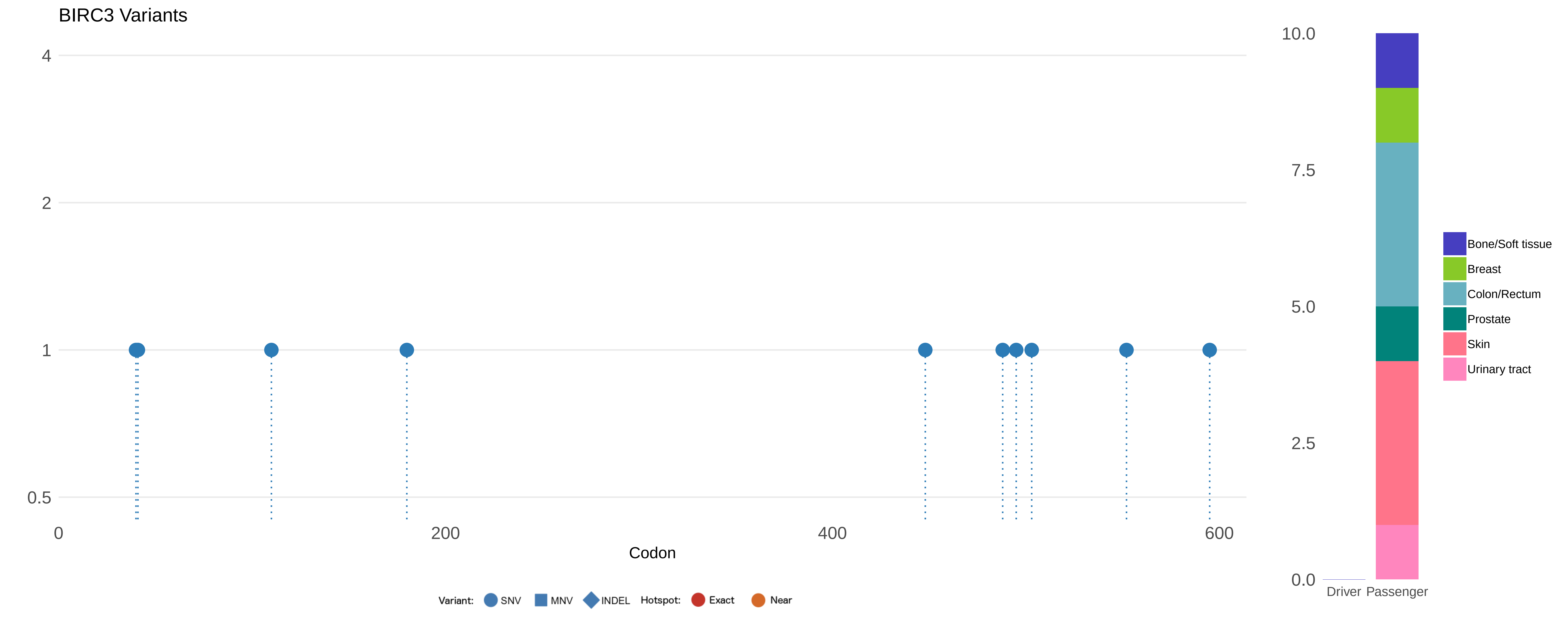

BIRC3 Variants
4
2
1
0.5
0
200
400
600
Codon
Variant: SNV MNV INDEL Hotspot: Exact Near
10.0
7.5
5.0
2.5
0.0
Driver Passenger
Bone/Soft tissue
Breast
Colon/Rectum
Prostate
Skin
Urinary tract

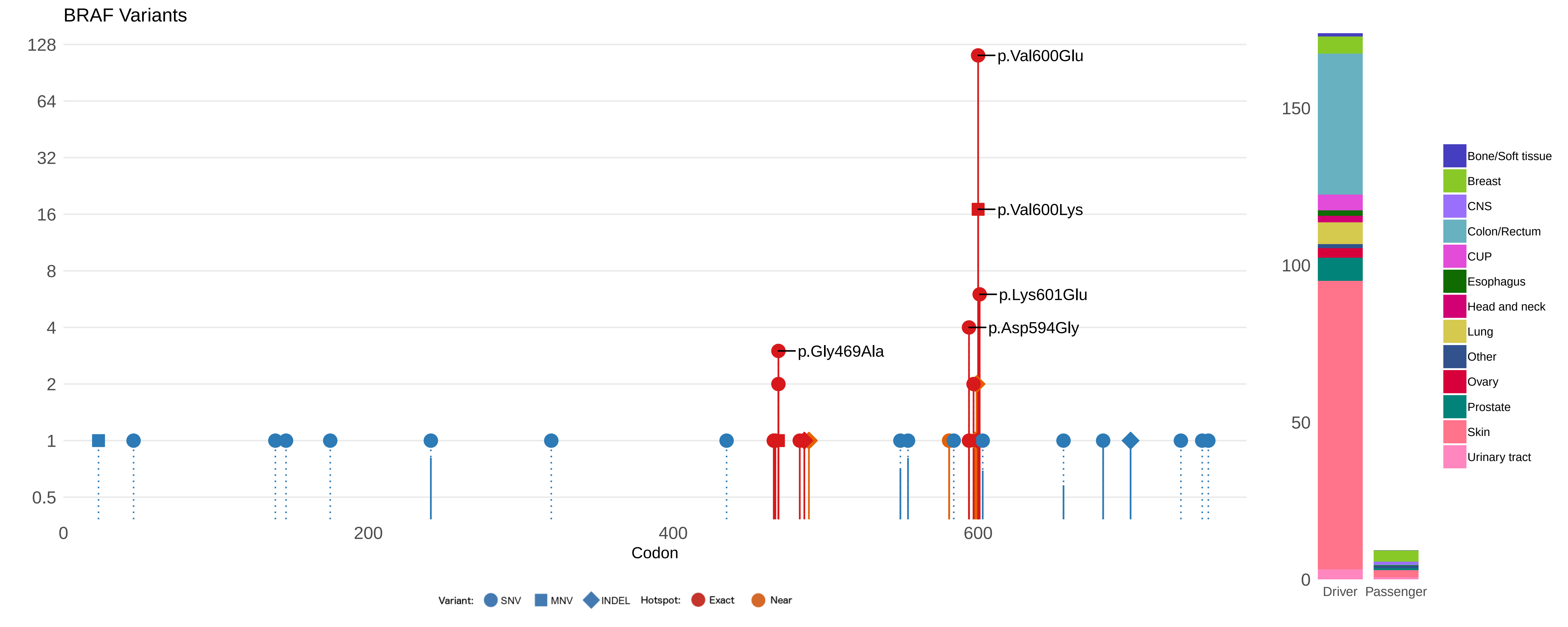

BRAF Variants
128
64
32
16
8
4
2
1
0.5
0
200
400
600
Codon
p.Val600Glu
p.Val600Lys
p.Lys601Glu
p.Asp594Gly
p.Gly469Ala
Variant:
SNV
MNV
INDEL
Hotspot:
Exact
Near
150
100
50
0
Driver
Passenger
Bone/Soft tissue
Breast
CNS
Colon/Rectum
CUP
Esophagus
Head and neck
Lung
Other
Ovary
Prostate
Skin
Urinary tract

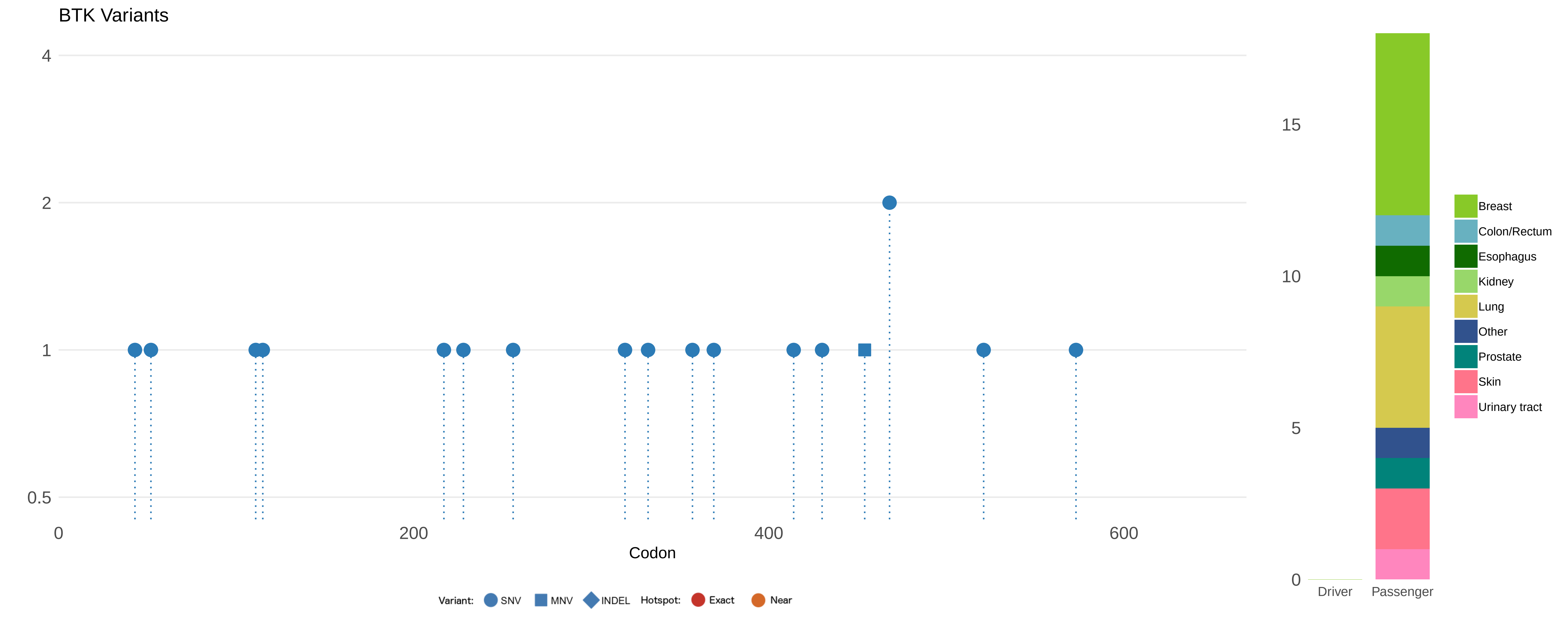

BTK Variants
4
2
1
0.5
0
200
400
600
Codon
Variant:
SNV
MNV
INDEL
Hotspot:
Exact
Near
15
10
5
0
Driver
Passenger
Breast
Colon/Rectum
Esophagus
Kidney
Lung
Other
Prostate
Skin
Urinary tract

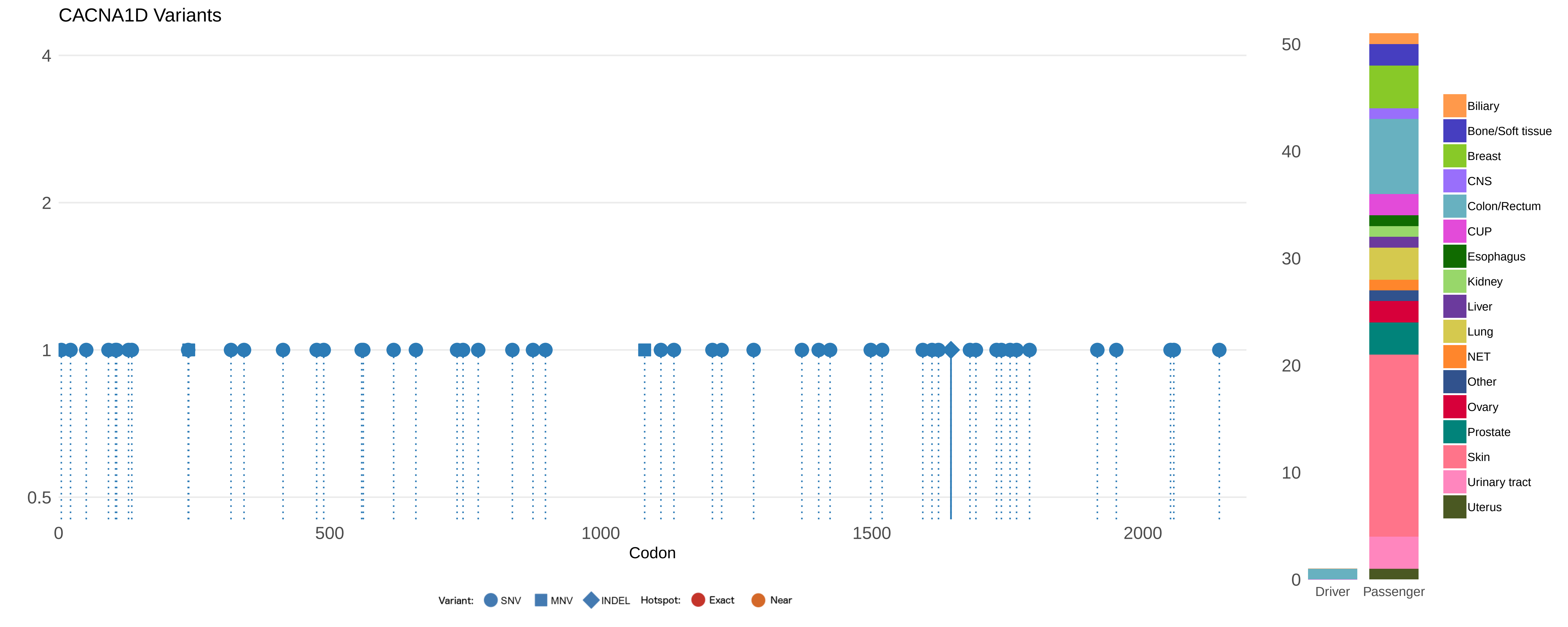

CACNA1D Variants
4
2
1
0.5
0
500
1000
1500
2000
Codon
Variant: SNV MNV INDEL Hotspot: Exact Near
50
40
30
20
10
0
Driver
Passenger
Biliary
Bone/Soft tissue
Breast
CNS
Colon/Rectum
CUP
Esophagus
Kidney
Liver
Lung
NET
Other
Ovary
Prostate
Skin
Urinary tract
Uterus

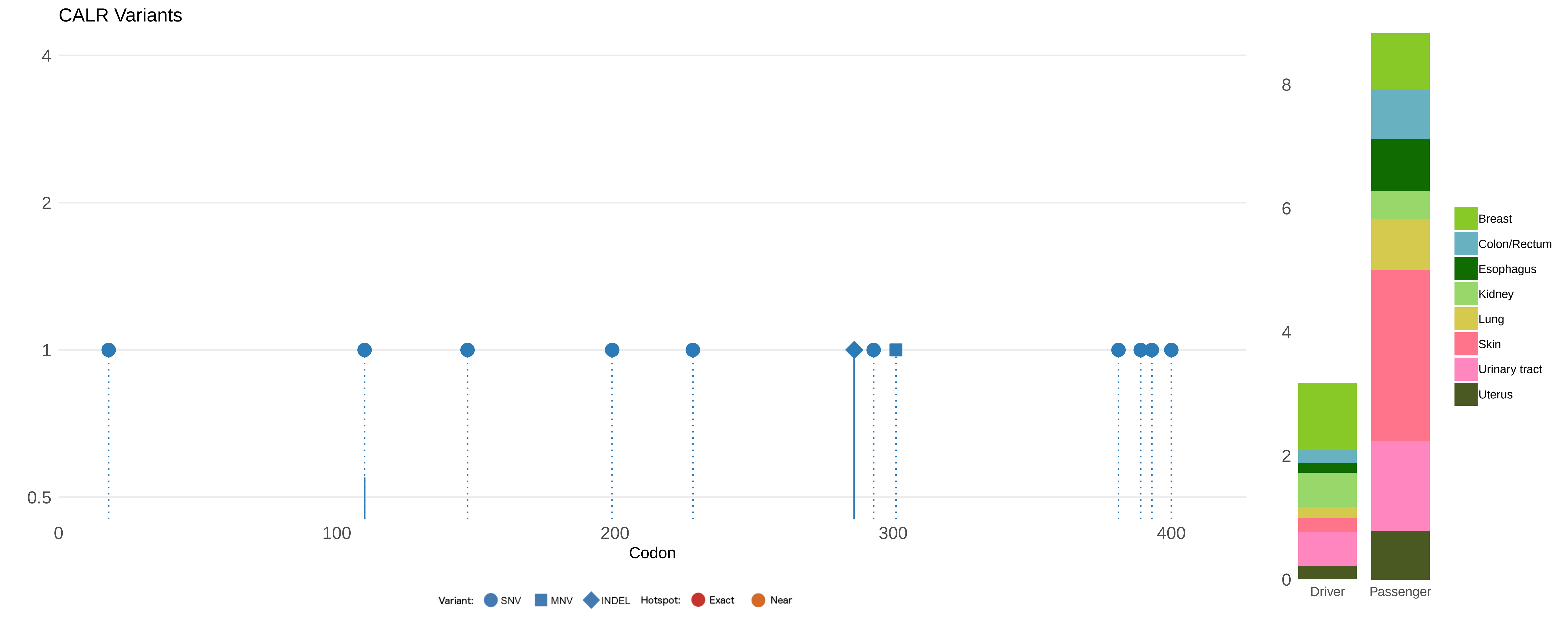
CALR Variants
4
2
1
0.5
0
100
200
300
400
Codon
Variant:
SNV
MNV
INDEL
Hotspot:
Exact
Near
8
6
4
2
0
Driver
Passenger
Breast
Colon/Rectum
Esophagus
Kidney
Lung
Skin
Urinary tract
Uterus

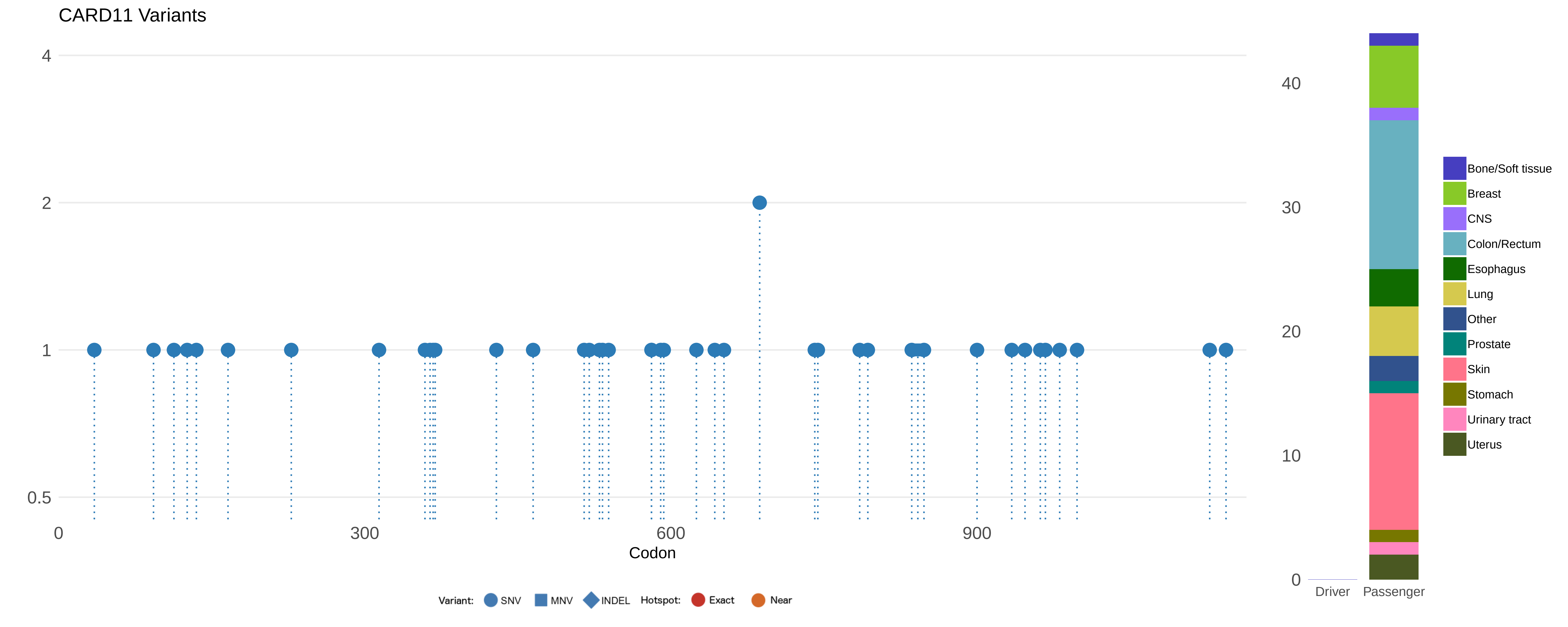

CARD11 Variants
4
2
1
0.5
0
300
600
900
Codon
40
30
20
10
0
Driver
Passenger
Bone/Soft tissue
Breast
CNS
Colon/Rectum
Esophagus
Lung
Other
Prostate
Skin
Stomach
Urinary tract
Uterus
Variant:
SNV
MNV
INDEL
Hotspot:
Exact
Near

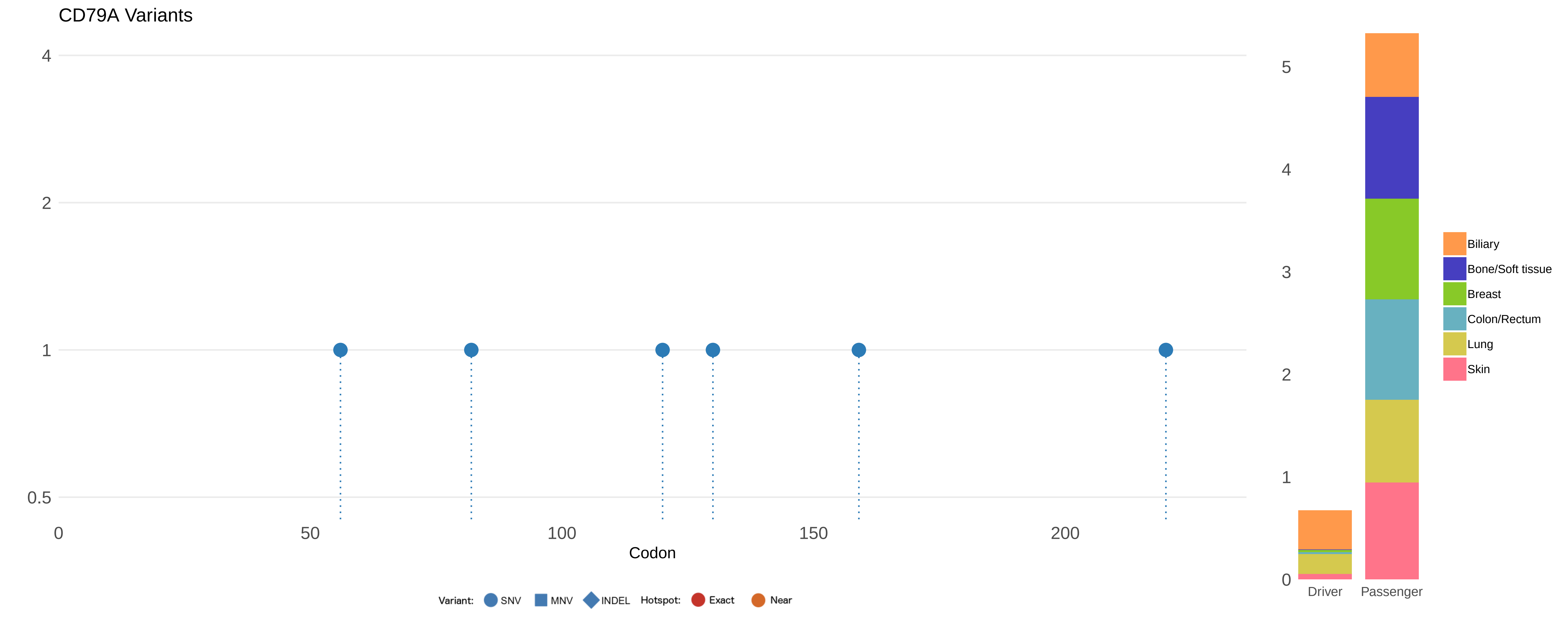

CD79A Variants
4
2
1
0.5
0
50
100
150
200
Codon
Variant: SNV MNV INDEL Hotspot: Exact Near
5
4
3
2
1
0
Driver
Passenger
Biliary
Bone/Soft tissue
Breast
Colon/Rectum
Lung
Skin

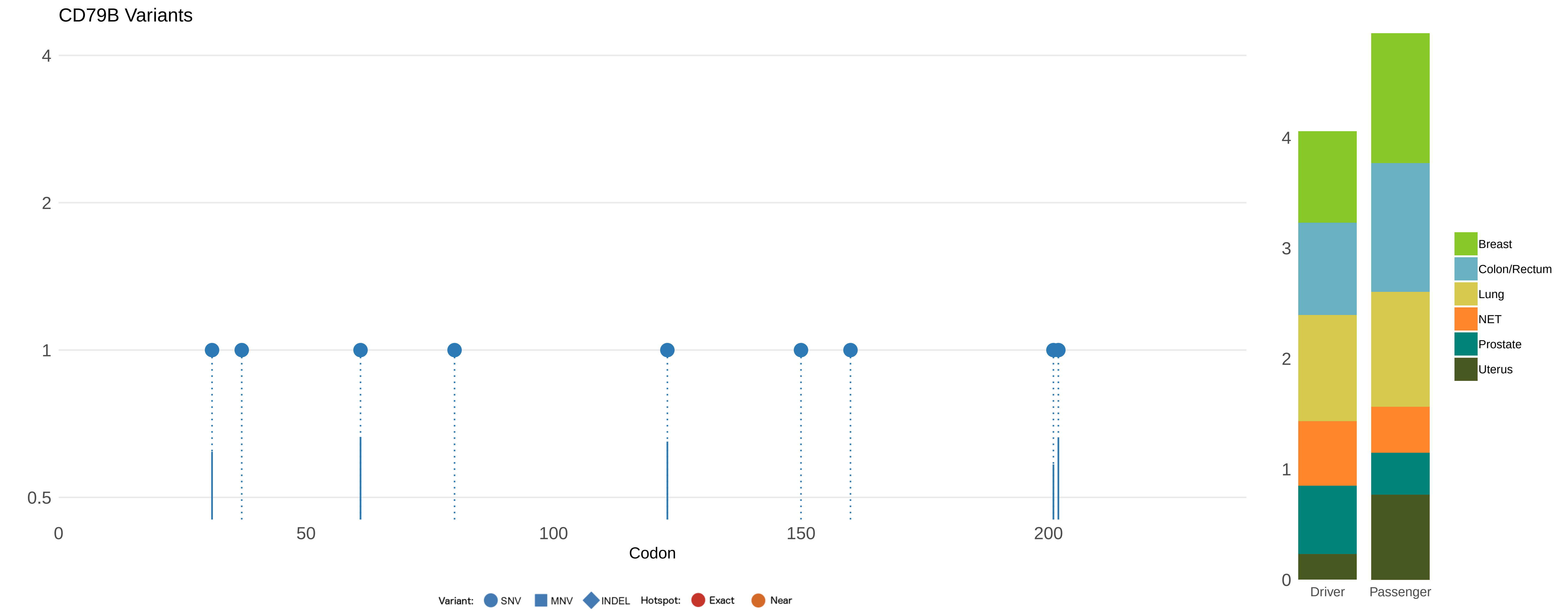

CD79B Variants
4
2
1
0.5
0
50
100
150
200
Codon
Variant:
SNV
MNV
INDEL
Hotspot:
Exact
Near
4
3
2
1
0
Driver
Passenger
Breast
Colon/Rectum
Lung
NET
Prostate
Uterus

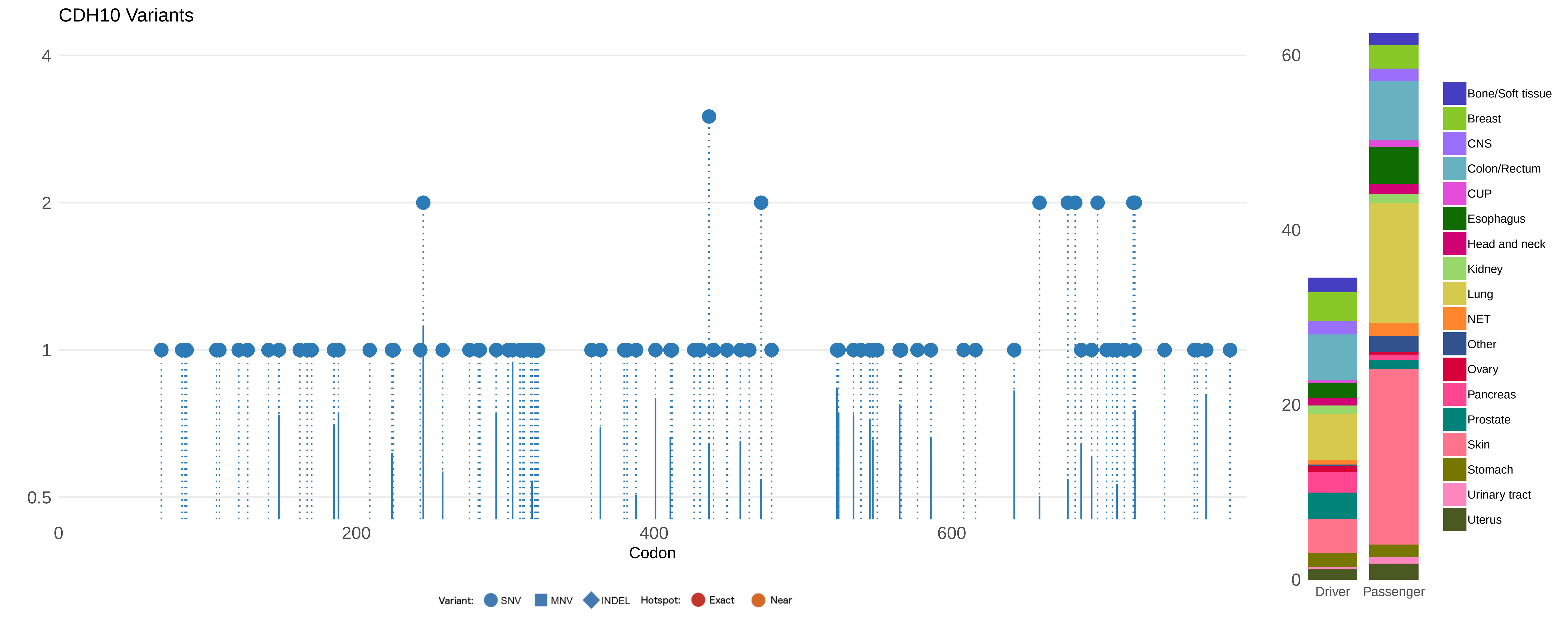

CDH10 Variants
4
2
1
0.5
0
200
400
600
Codon
60
40
20
0
Driver
Passenger
Bone/Soft tissue
Breast
CNS
Colon/Rectum
CUP
Esophagus
Head and neck
Kidney
Lung
NET
Other
Ovary
Pancreas
Prostate
Skin
Stomach
Urinary tract
Uterus
Variant: SNV MNV INDEL Hotspot: Exact Near

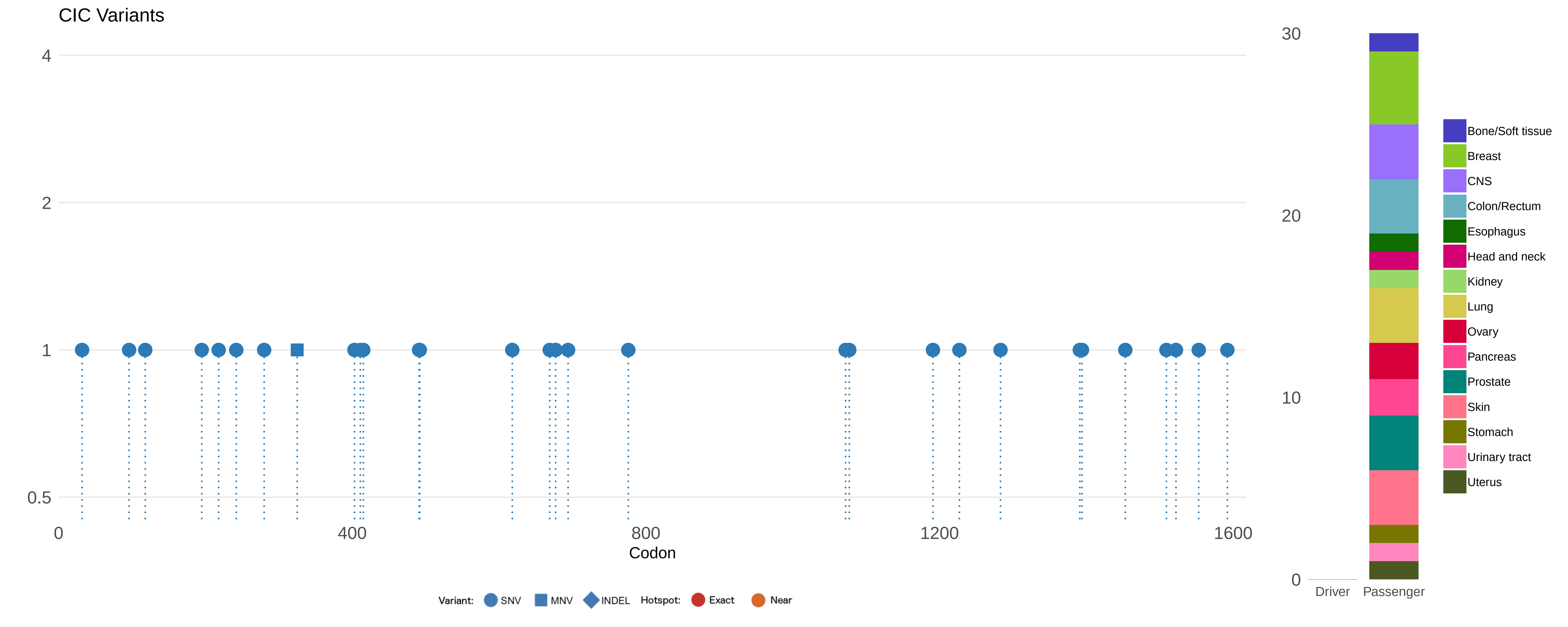

CIC Variants
4
2
1
0.5
0
400
800
1200
1600
Codon
Variant: SNV MNV INDEL Hotspot: Exact Near
30
20
10
0
Driver
Passenger
Bone/Soft tissue
Breast
CNS
Colon/Rectum
Esophagus
Head and neck
Kidney
Lung
Ovary
Pancreas
Prostate
Skin
Stomach
Urinary tract
Uterus

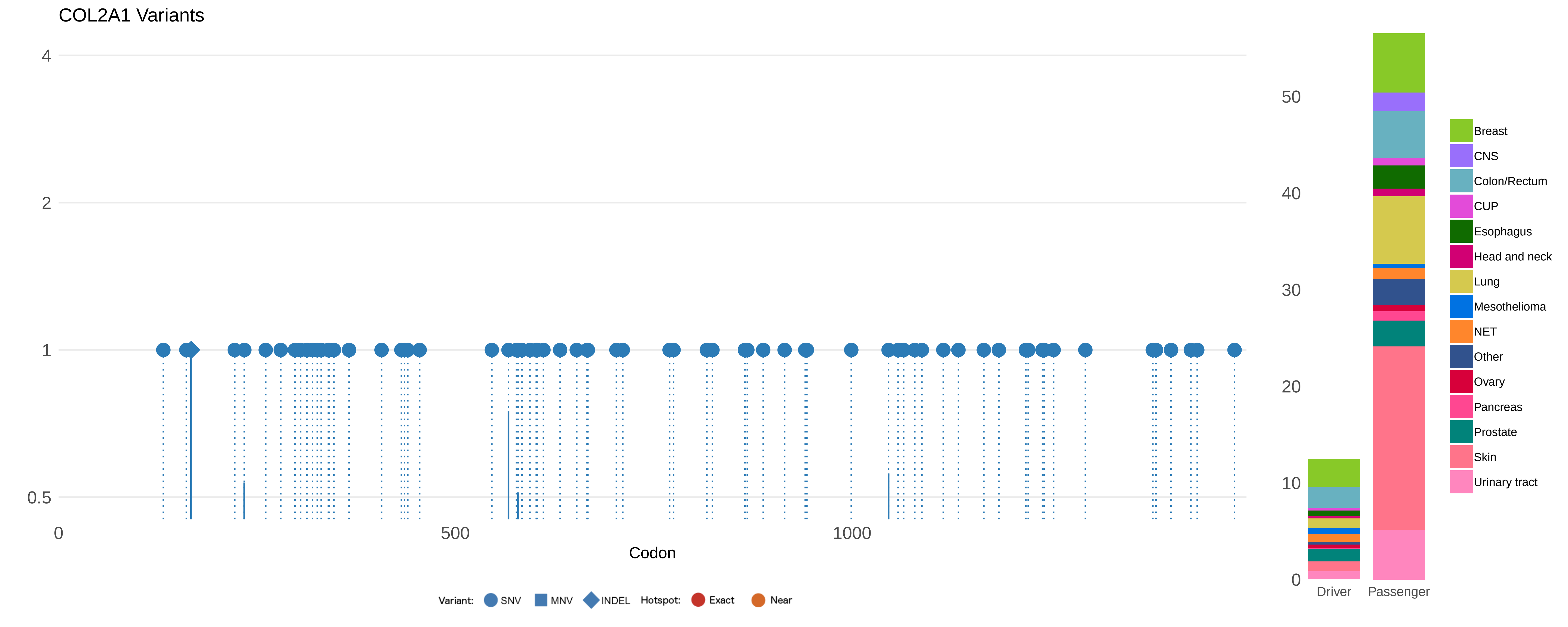

COL2A1 Variants
4
2
1
0.5
0
500
1000
Codon
50
40
30
20
10
0
Driver
Passenger
Breast
CNS
Colon/Rectum
CUP
Esophagus
Head and neck
Lung
Mesothelioma
NET
Other
Ovary
Pancreas
Prostate
Skin
Urinary tract
Variant: SNV MNV INDEL Hotspot: Exact Near

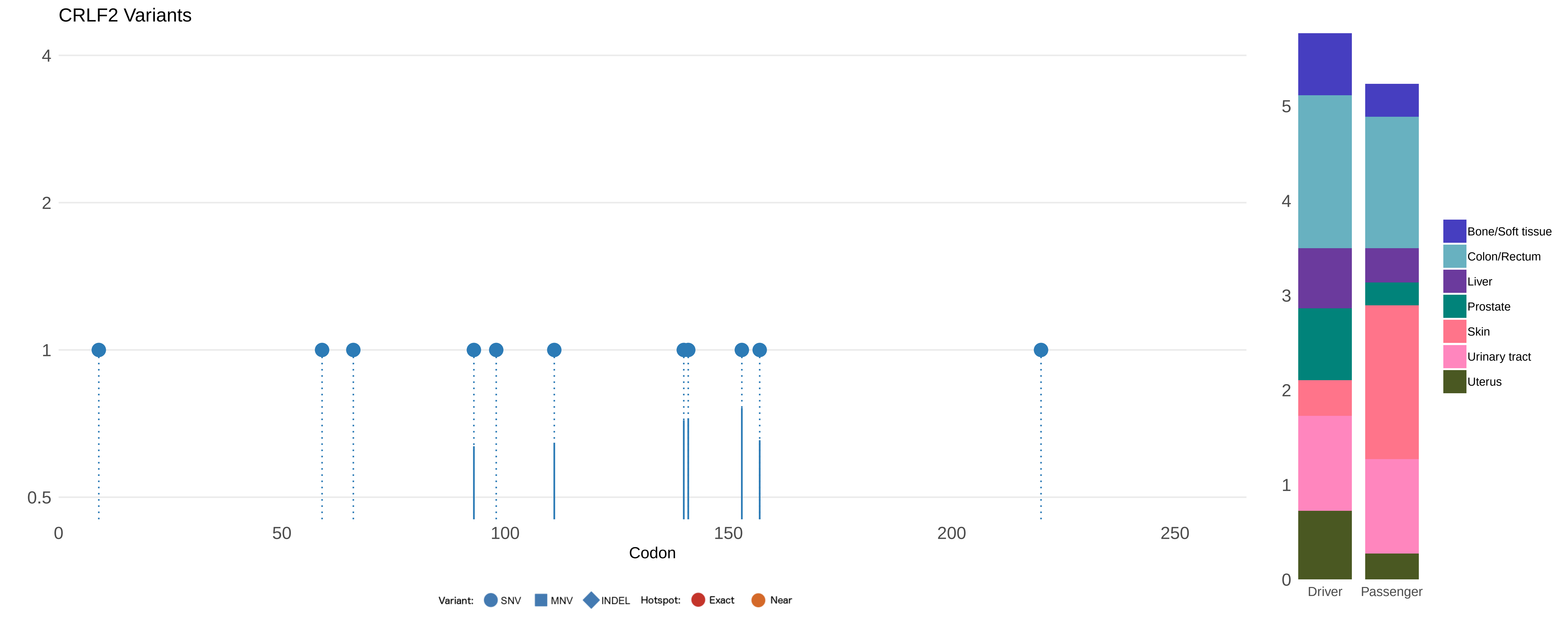

CRLF2 Variants
4
2
1
0.5
0
50
100
150
200
250
Codon
Variant:
SNV
MNV
INDEL
Hotspot:
Exact
Near
5
4
3
2
1
0
Driver
Passenger
Bone/Soft tissue
Colon/Rectum
Liver
Prostate
Skin
Urinary tract
Uterus

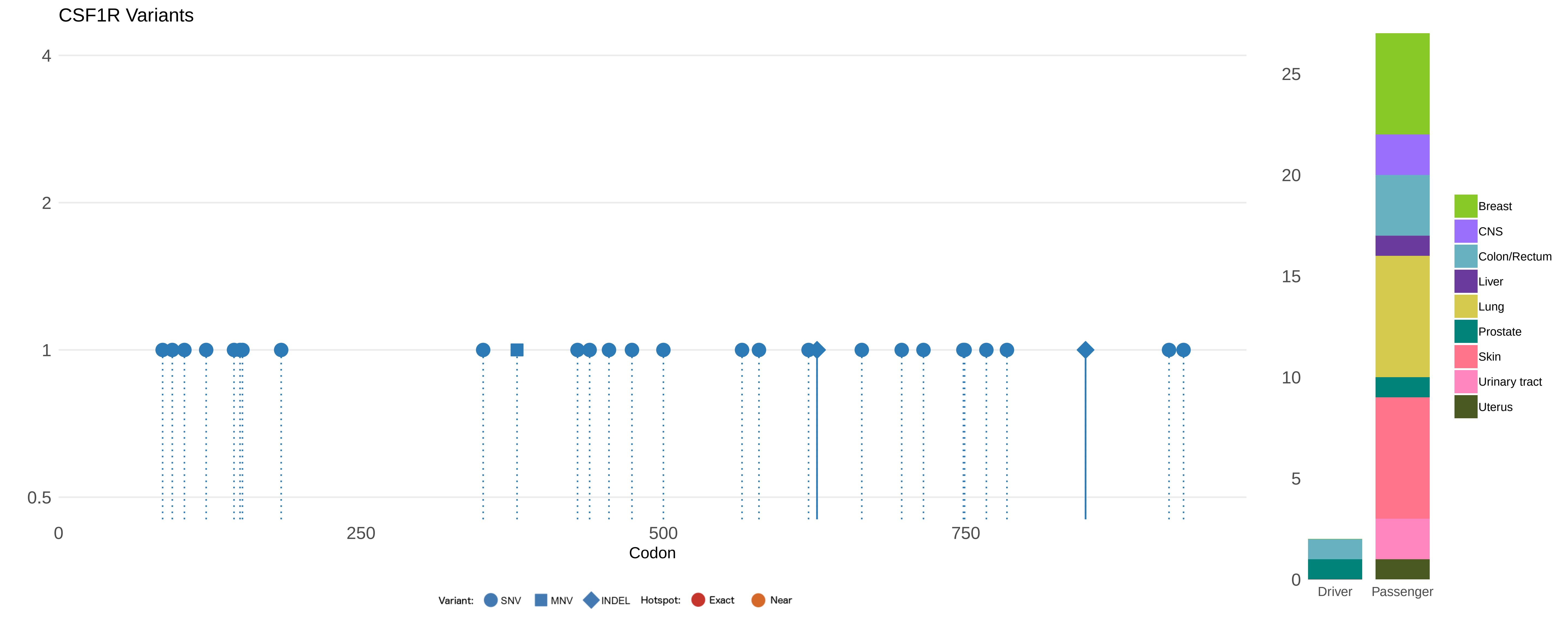

CSF1R Variants
4
2
1
0.5
0
250
500
750
Codon
Variant: SNV MNV INDEL Hotspot: Exact Near
25
20
15
10
5
0
Driver
Passenger
Breast
CNS
Colon/Rectum
Liver
Lung
Prostate
Skin
Urinary tract
Uterus

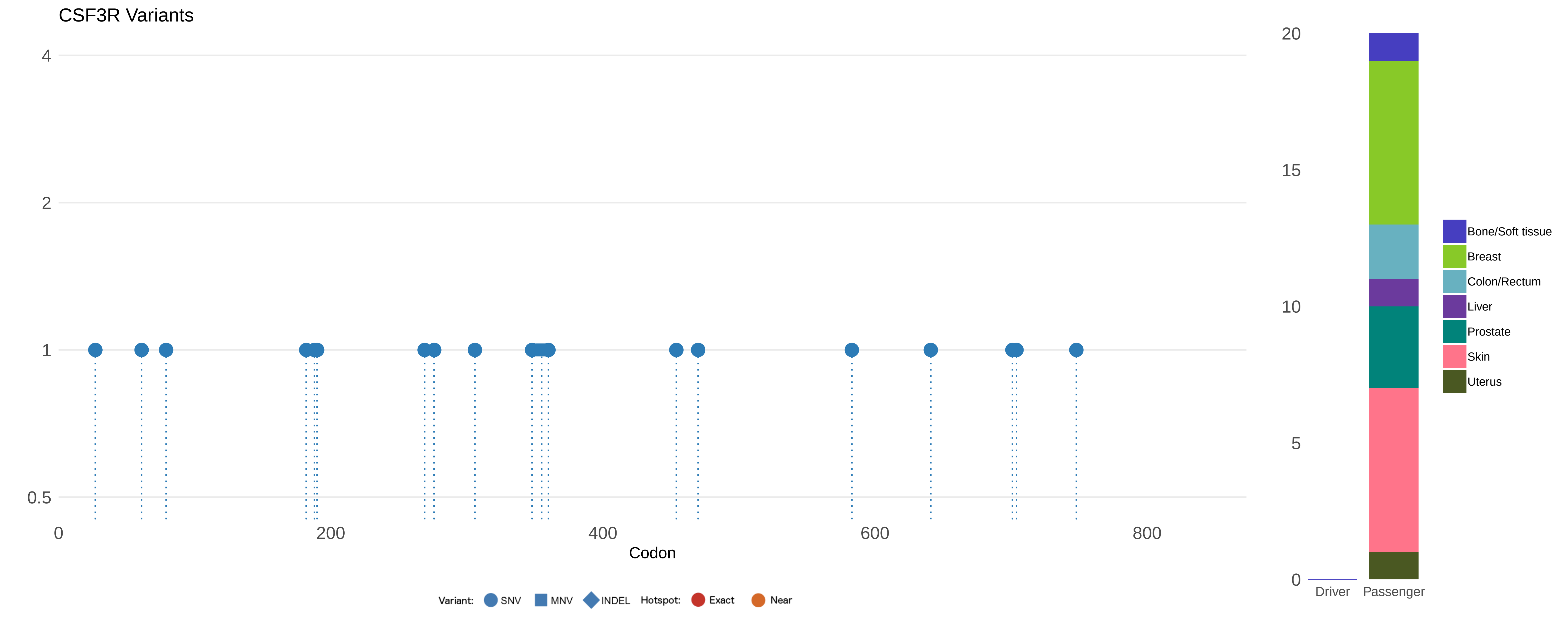
CSF3R Variants
4
2
1
0.5
0
200
400
600
800
Codon
Variant:
SNV
MNV
INDEL
Hotspot:
Exact
Near
20
15
10
5
0
Driver
Passenger
Bone/Soft tissue
Breast
Colon/Rectum
Liver
Prostate
Skin
Uterus

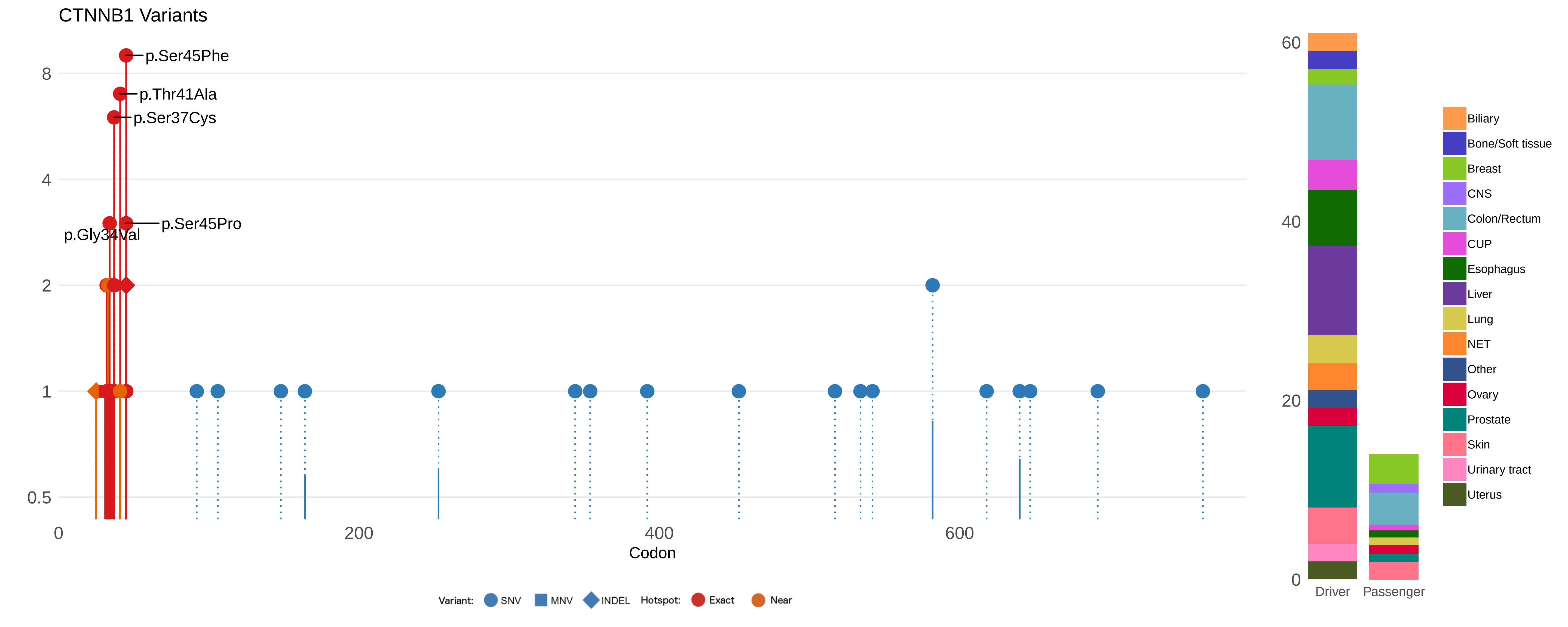

CTNNB1 Variants
p.Ser45Phe
p.Thr41Ala
p.Ser37Cys
p.Ser45Pro
p.Gly34Val
8
4
2
1
0.5
0
200
400
600
Codon
Variant: SNV MNV INDEL Hotspot: Exact Near
60
40
20
0
Driver
Passenger
Biliary
Bone/Soft tissue
Breast
CNS
Colon/Rectum
CUP
Esophagus
Liver
Lung
NET
Other
Ovary
Prostate
Skin
Urinary tract
Uterus

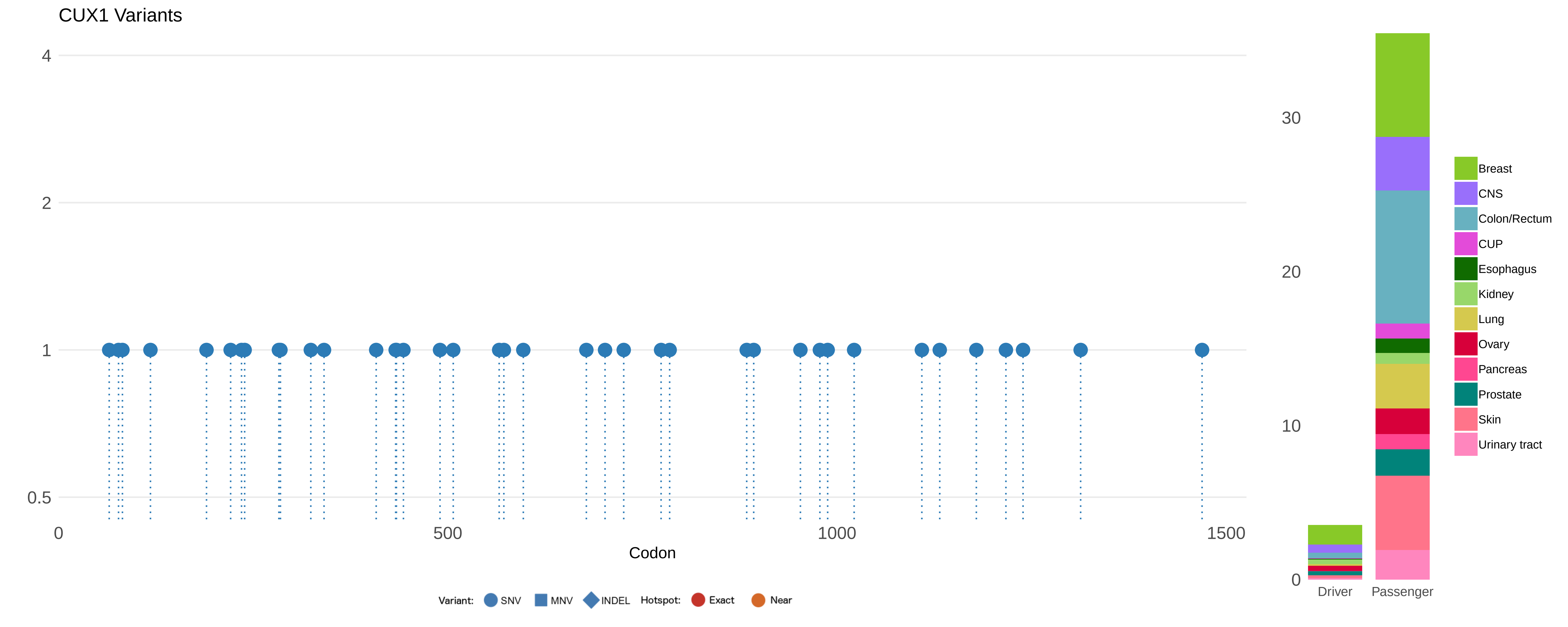

CUX1 Variants
4
2
1
0.5
0
500
1000
1500
Codon
Variant:
SNV
MNV
INDEL
Hotspot:
Exact
Near
30
20
10
0
Driver
Passenger
Breast
CNS
Colon/Rectum
CUP
Esophagus
Kidney
Lung
Ovary
Pancreas
Prostate
Skin
Urinary tract

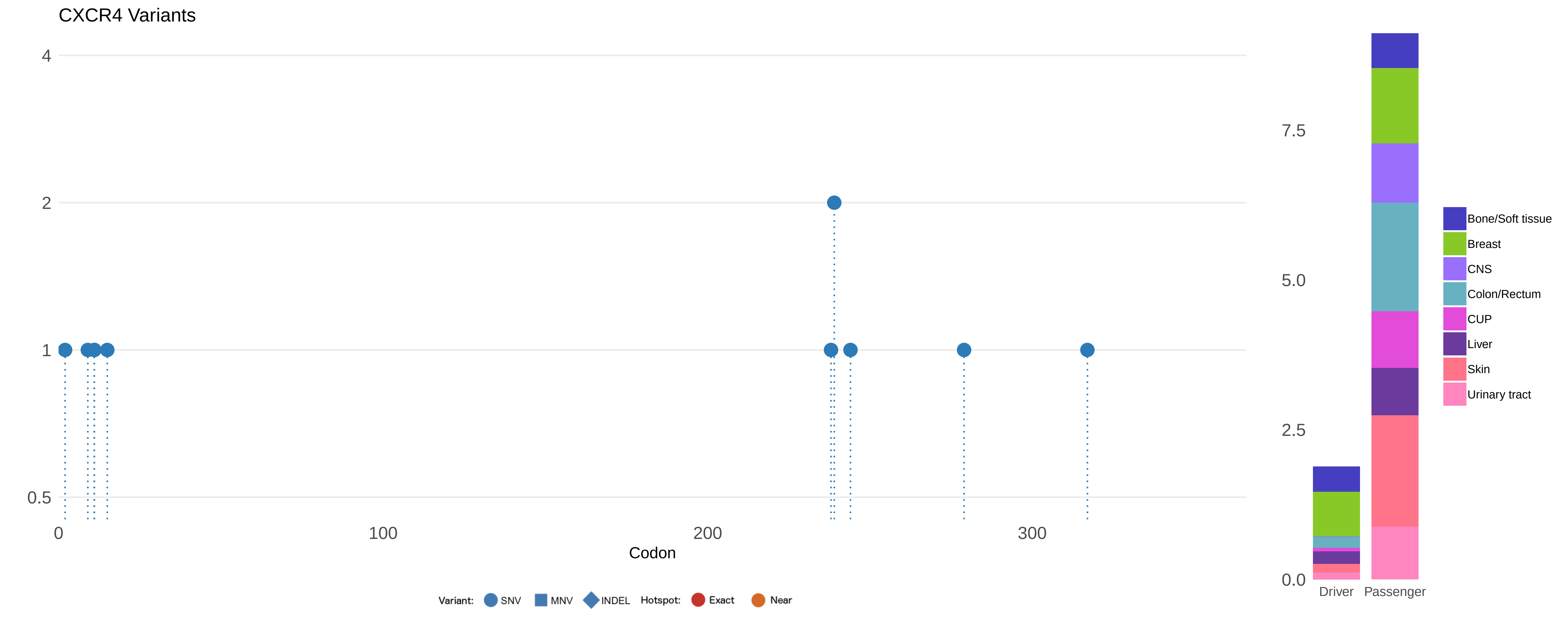

CXCR4 Variants
4
2
1
0.5
0
100
200
300
Codon
Variant:
SNV
MNV
INDEL
Hotspot:
Exact
Near
7.5
5.0
2.5
0.0
Driver
Passenger
Bone/Soft tissue
Breast
CNS
Colon/Rectum
CUP
Liver
Skin
Urinary tract

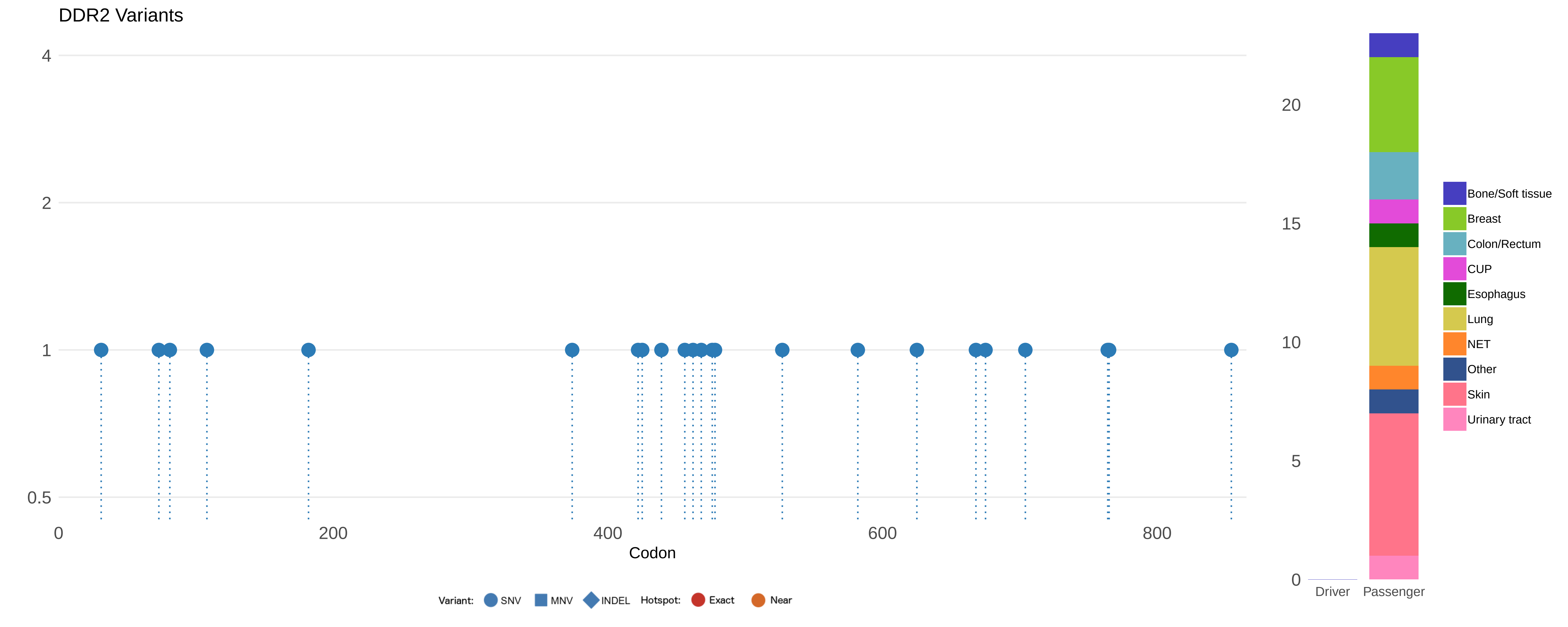
DDR2 Variants
4
2
1
0.5
0
200
400
600
800
Codon
Variant: SNV MNV INDEL Hotspot: Exact Near
20
15
10
5
0
Driver
Passenger
Bone/Soft tissue
Breast
Colon/Rectum
CUP
Esophagus
Lung
NET
Other
Skin
Urinary tract

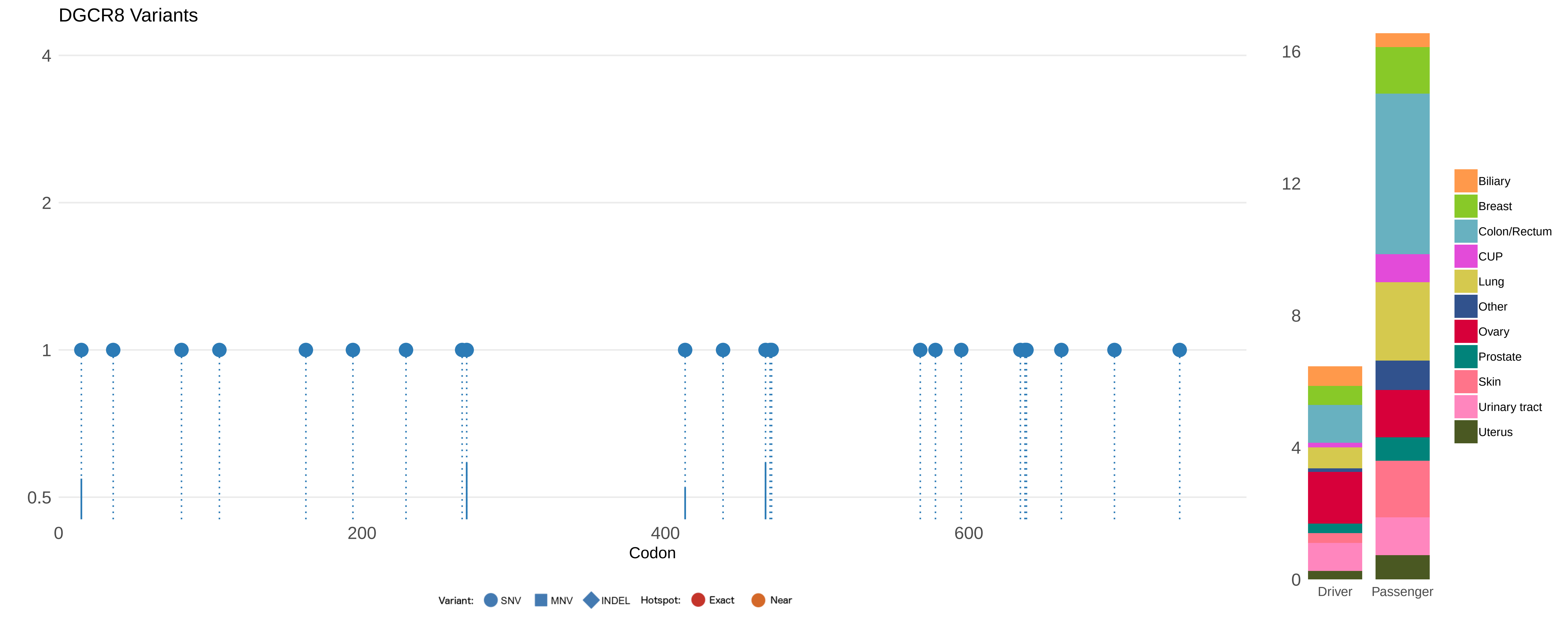

DGCR8 Variants
4
2
1
0.5
0
200
400
600
Codon
Variant:
SNV
MNV
INDEL
Hotspot:
Exact
Near
16
12
8
4
0
Driver
Passenger
Biliary
Breast
Colon/Rectum
CUP
Lung
Other
Ovary
Prostate
Skin
Urinary tract
Uterus

EEF1A1 Variants

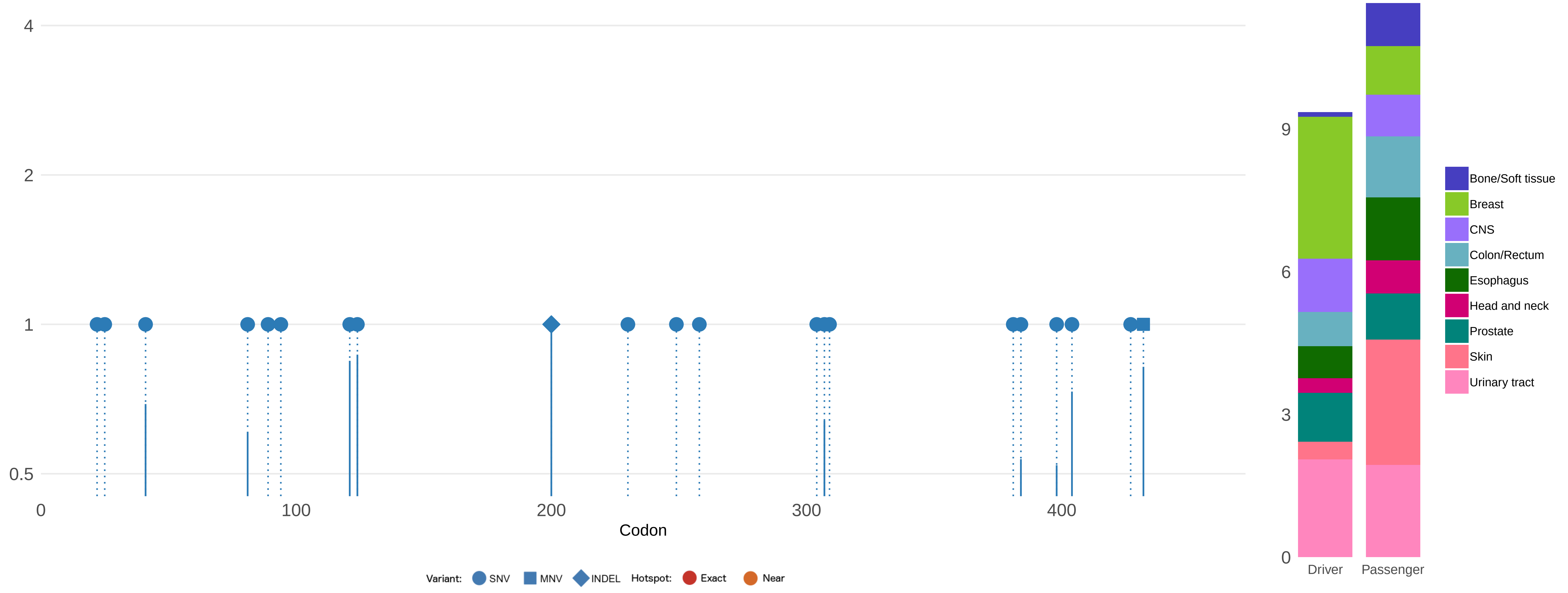

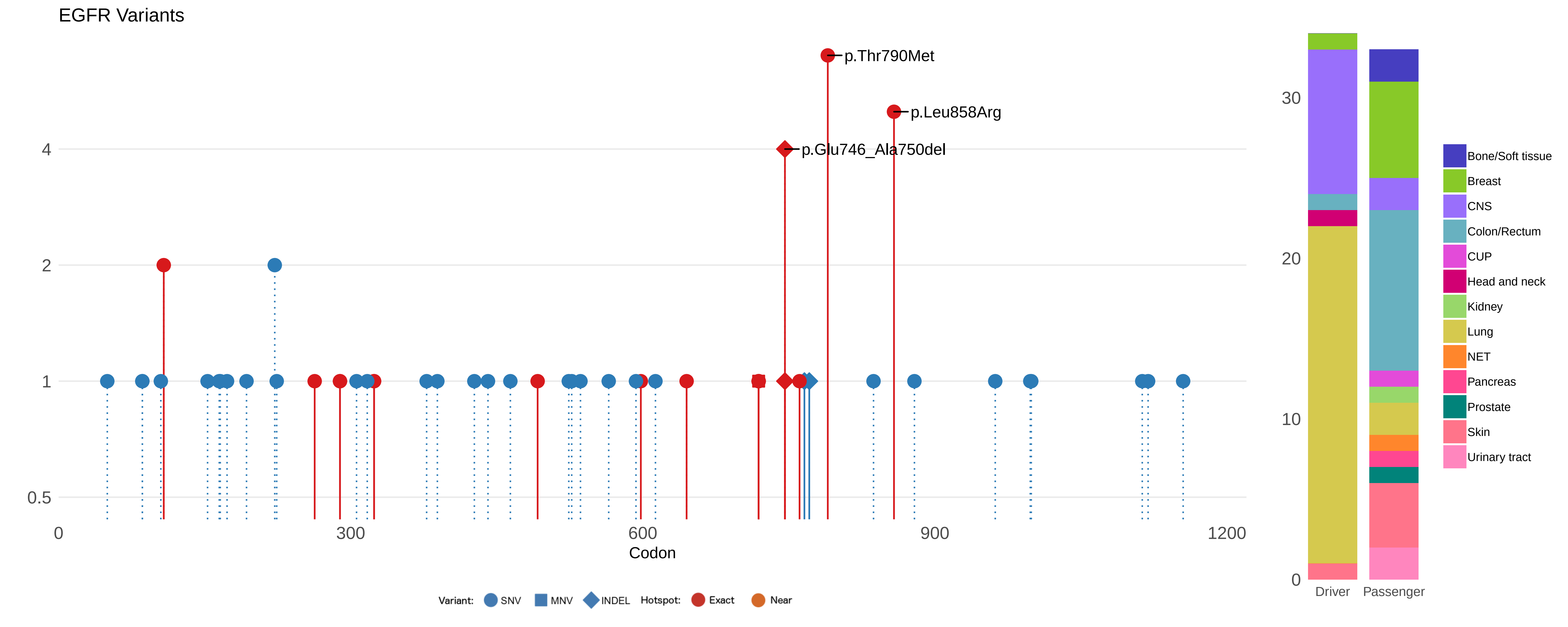
EGFR Variants
p.Thr790Met
p.Leu858Arg
p.Glu746_Ala750del
4
2
1
0.5
0
300
600
900
1200
Codon
Variant: SNV MNV INDEL Hotspot: Exact Near
30
20
10
0
Driver
Passenger
Bone/Soft tissue
Breast
CNS
Colon/Rectum
CUP
Head and neck
Kidney
Lung
NET
Pancreas
Prostate
Skin
Urinary tract

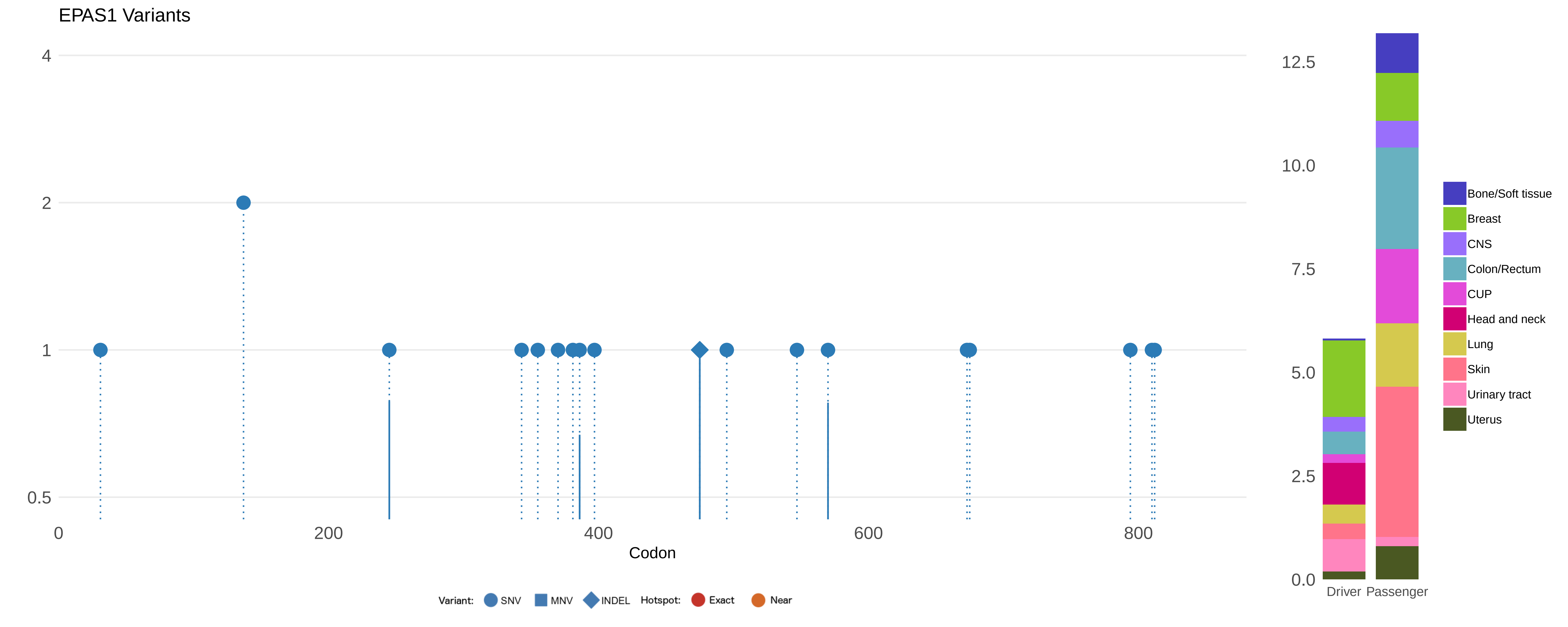

EPAS1 Variants
4
2
1
0.5
0
200
400
600
800
Codon
Variant:
SNV
MNV
INDEL
Hotspot:
Exact
Near
12.5
10.0
7.5
5.0
2.5
0.0
Driver
Passenger
Bone/Soft tissue
Breast
CNS
Colon/Rectum
CUP
Head and neck
Lung
Skin
Urinary tract
Uterus

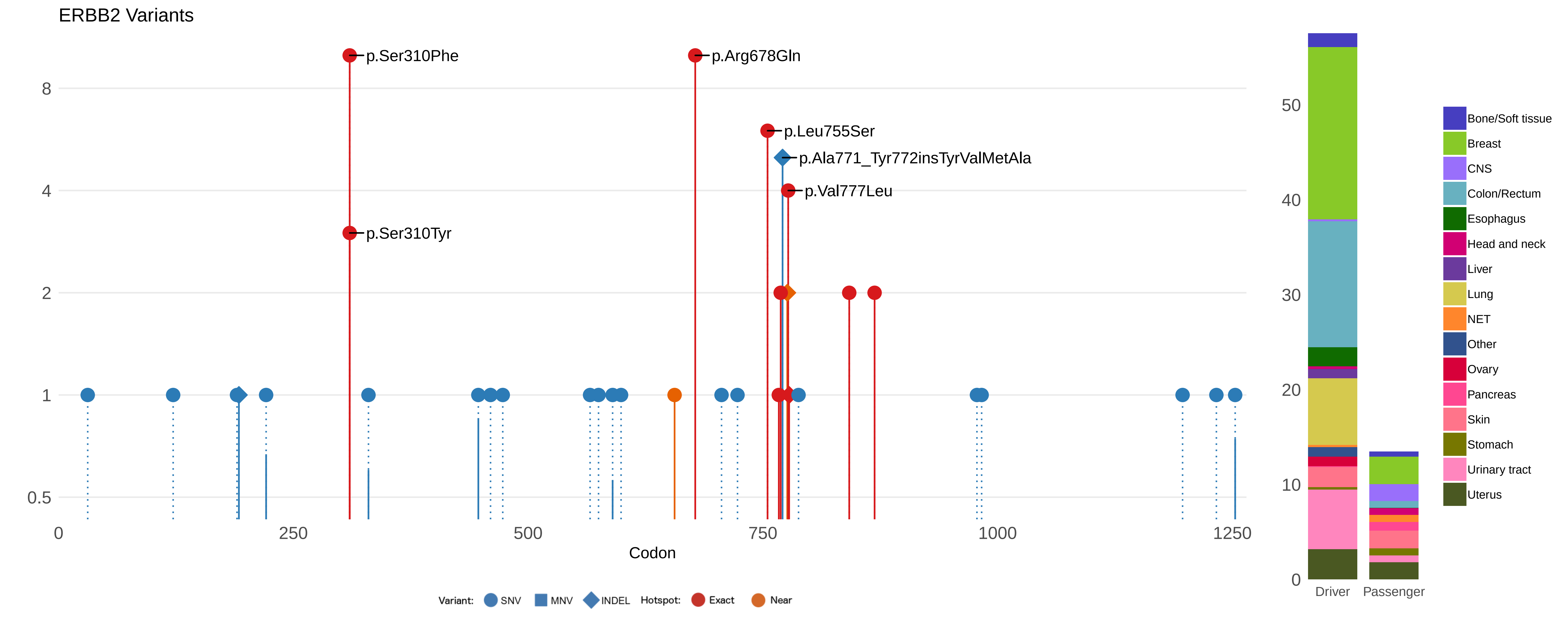

ERBB2 Variants
p.Ser310Phe
p.Arg678Gln
p.Leu755Ser
p.Ala771_Tyr772insTyrValMetAla
p.Val777Leu
p.Ser310Tyr
8
4
2
1
0.5
0
250
500
750
1000
1250
Codon
Variant: SNV MNV INDEL Hotspot: Exact Near
50
40
30
20
10
0
Driver
Passenger
Bone/Soft tissue
Breast
CNS
Colon/Rectum
Esophagus
Head and neck
Liver
Lung
NET
Other
Ovary
Pancreas
Skin
Stomach
Urinary tract
Uterus

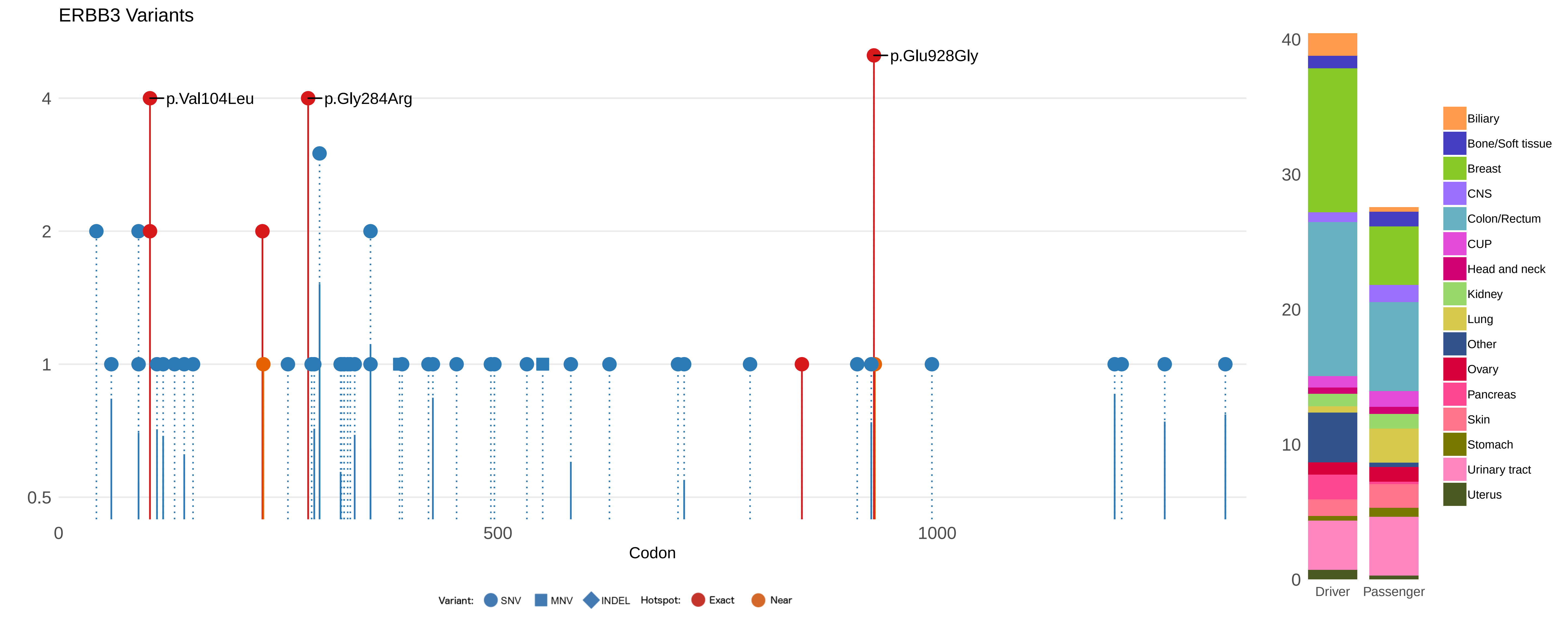

ERBB3 Variants
p.Val104Leu
p.Gly284Arg
p.Glu928Gly
4
2
1
0.5
0
500
1000
Codon
Variant: SNV MNV INDEL Hotspot: Exact Near
40
30
20
10
0
Driver
Passenger
Biliary
Bone/Soft tissue
Breast
CNS
Colon/Rectum
CUP
Head and neck
Kidney
Lung
Other
Ovary
Pancreas
Skin
Stomach
Urinary tract
Uterus

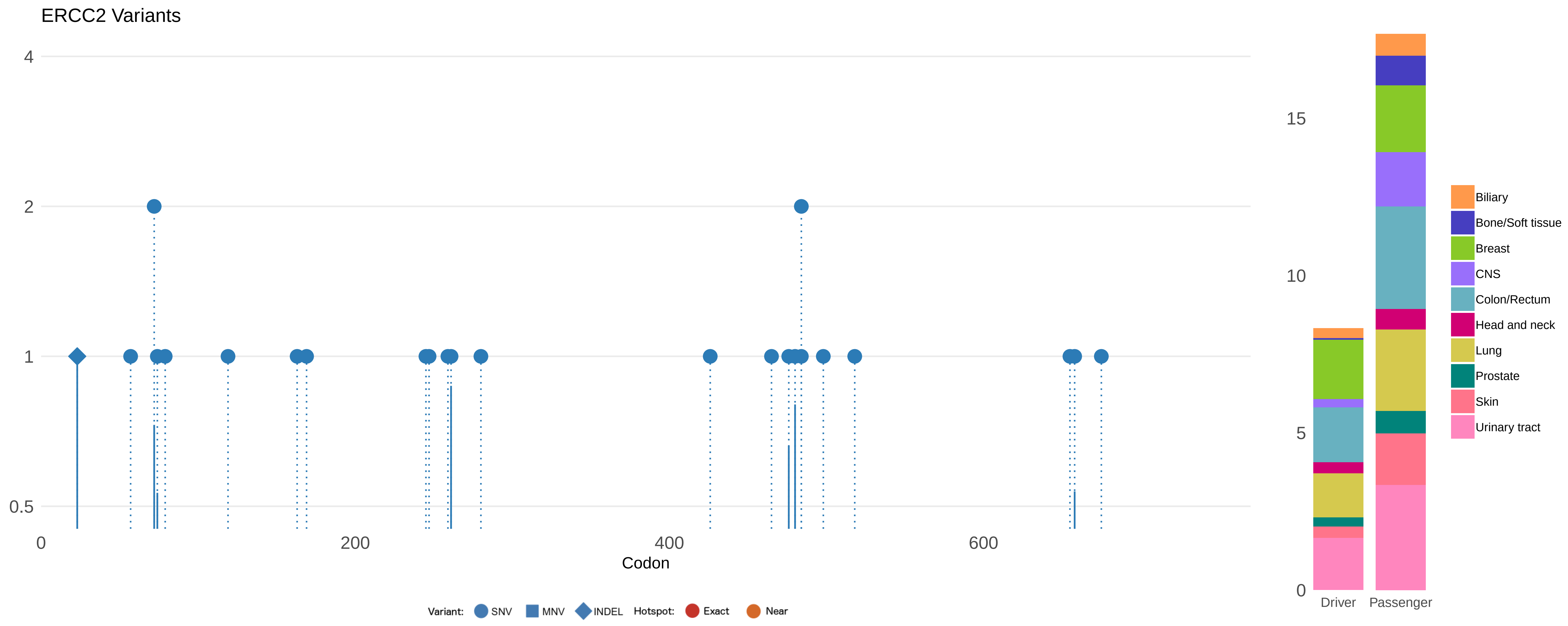

ERCC2 Variants
4
2
1
0.5
0
200
400
600
Codon
Variant: SNV MNV INDEL Hotspot: Exact Near
15
10
5
0
Driver
Passenger
Biliary
Bone/Soft tissue
Breast
CNS
Colon/Rectum
Head and neck
Lung
Prostate
Skin
Urinary tract

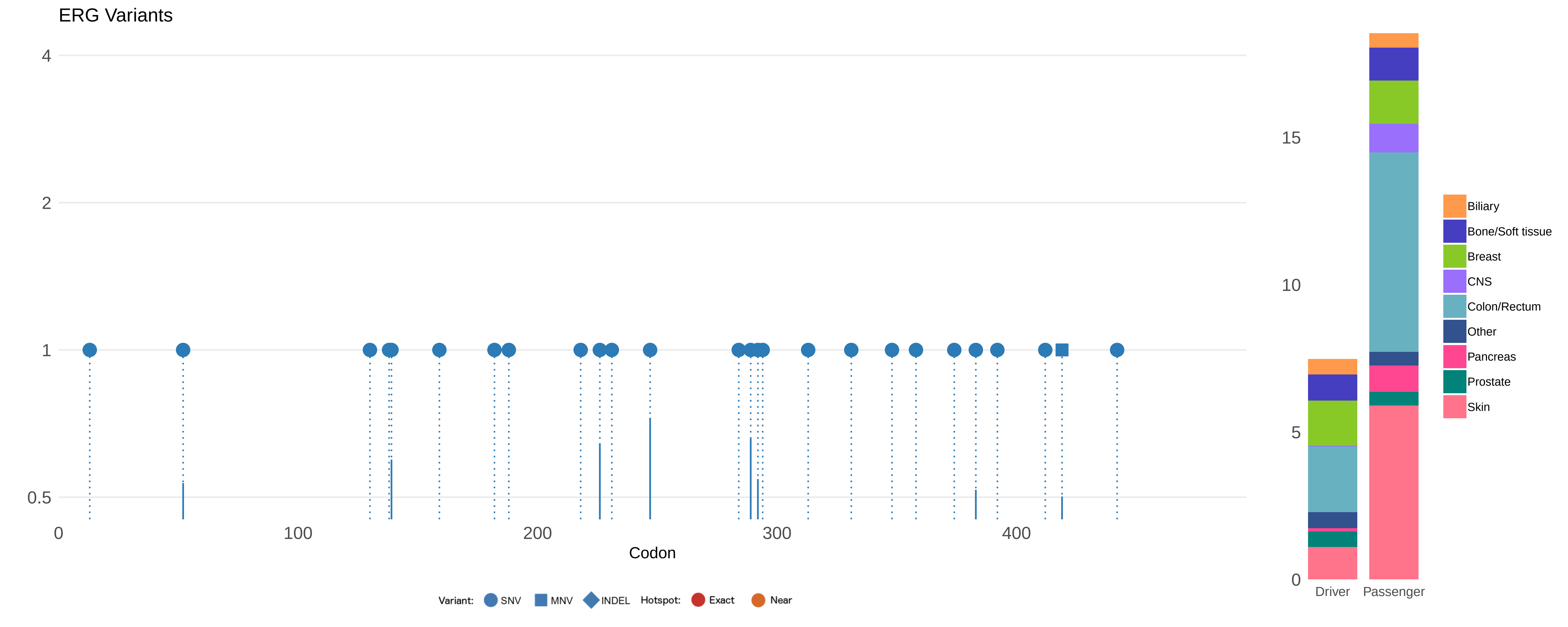

ERG Variants
4
2
1
0.5
0
100
200
300
400
Codon
Variant: SNV MNV INDEL Hotspot: Exact Near
15
10
5
0
Driver
Passenger
Biliary
Bone/Soft tissue
Breast
CNS
Colon/Rectum
Other
Pancreas
Prostate
Skin

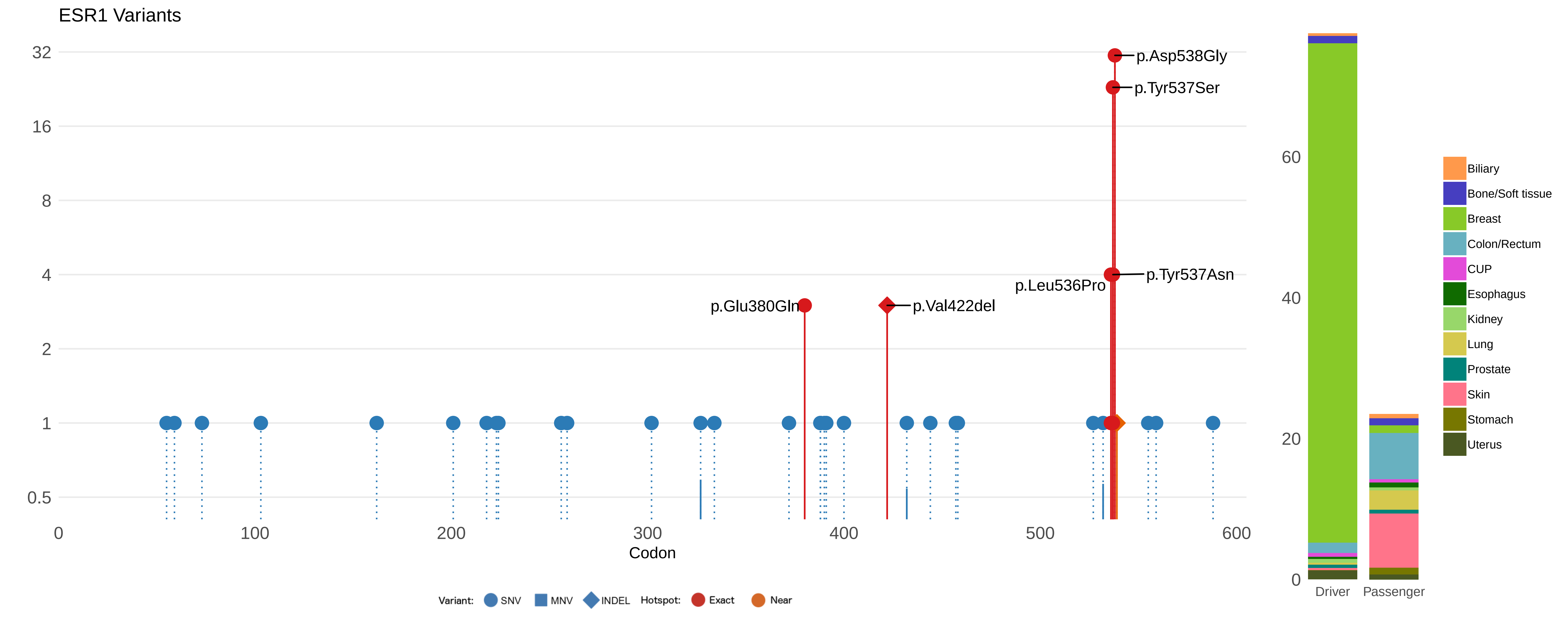

ESR1 Variants
32
16
8
4
2
1
0.5
p.Asp538Gly
p.Tyr537Ser
p.Tyr537Asn
p.Leu536Pro
p.Glu380Gln
p.Val422del
0
100
200
300
400
500
600
Codon
Variant: SNV MNV INDEL Hotspot: Exact Near
60
40
20
0
Driver
Passenger
Biliary
Bone/Soft tissue
Breast
Colon/Rectum
CUP
Esophagus
Kidney
Lung
Prostate
Skin
Stomach
Uterus

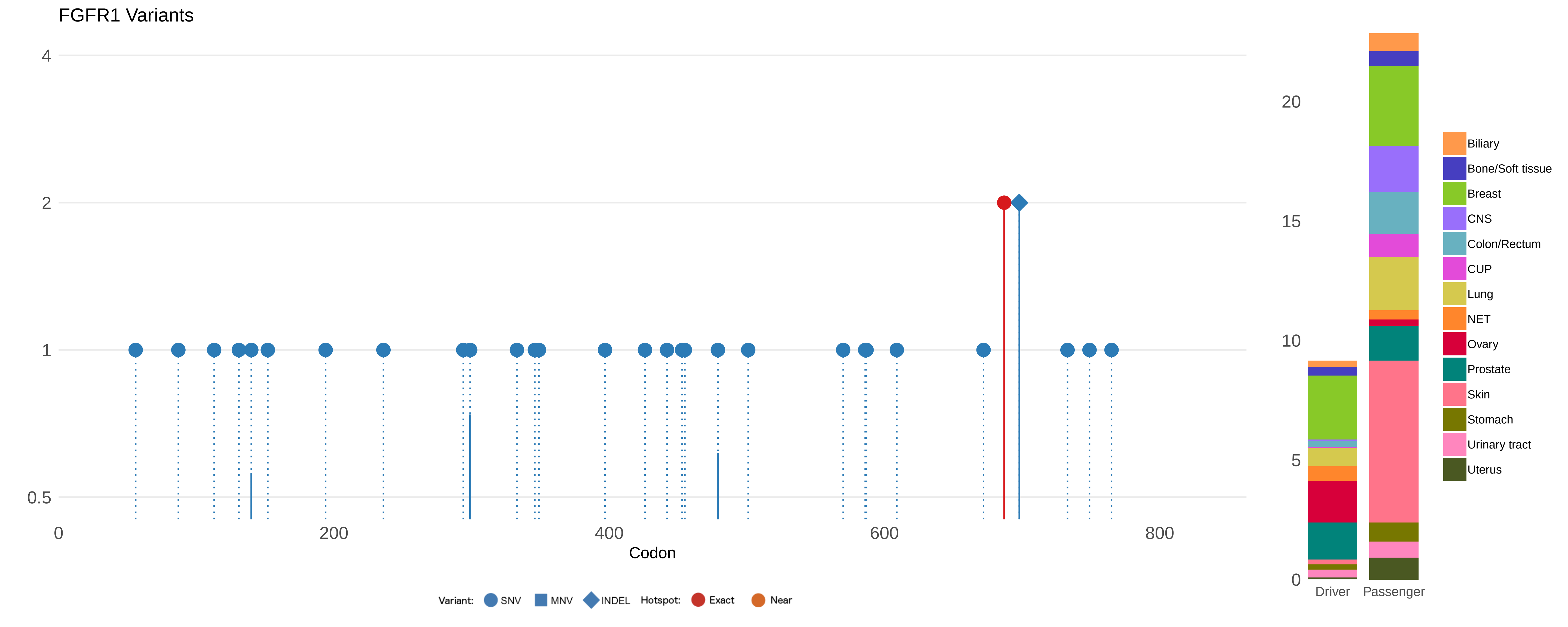
FGFR1 Variants
4
2
1
0.5
0
200
400
600
800
Codon
Variant:
SNV
MNV
INDEL
Hotspot:
Exact
Near
20
15
10
5
0
Driver
Passenger
Biliary
Bone/Soft tissue
Breast
CNS
Colon/Rectum
CUP
Lung
NET
Ovary
Prostate
Skin
Stomach
Urinary tract
Uterus

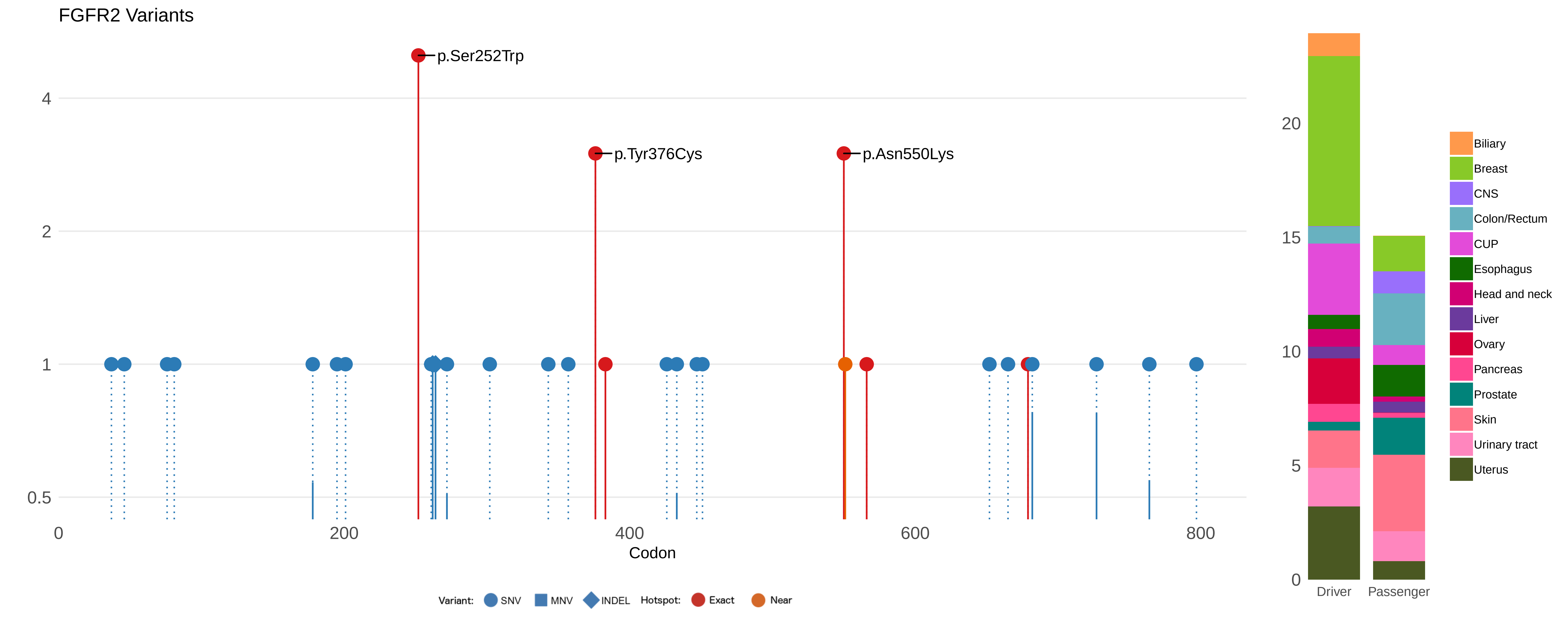

FGFR2 Variants
p.Ser252Trp
p.Tyr376Cys
p.Asn550Lys
4
2
1
0.5
0
200
400
600
800
Codon
Variant:
SNV
MNV
INDEL
Hotspot:
Exact
Near
20
15
10
5
0
Driver
Passenger
Biliary
Breast
CNS
Colon/Rectum
CUP
Esophagus
Head and neck
Liver
Ovary
Pancreas
Prostate
Skin
Urinary tract
Uterus

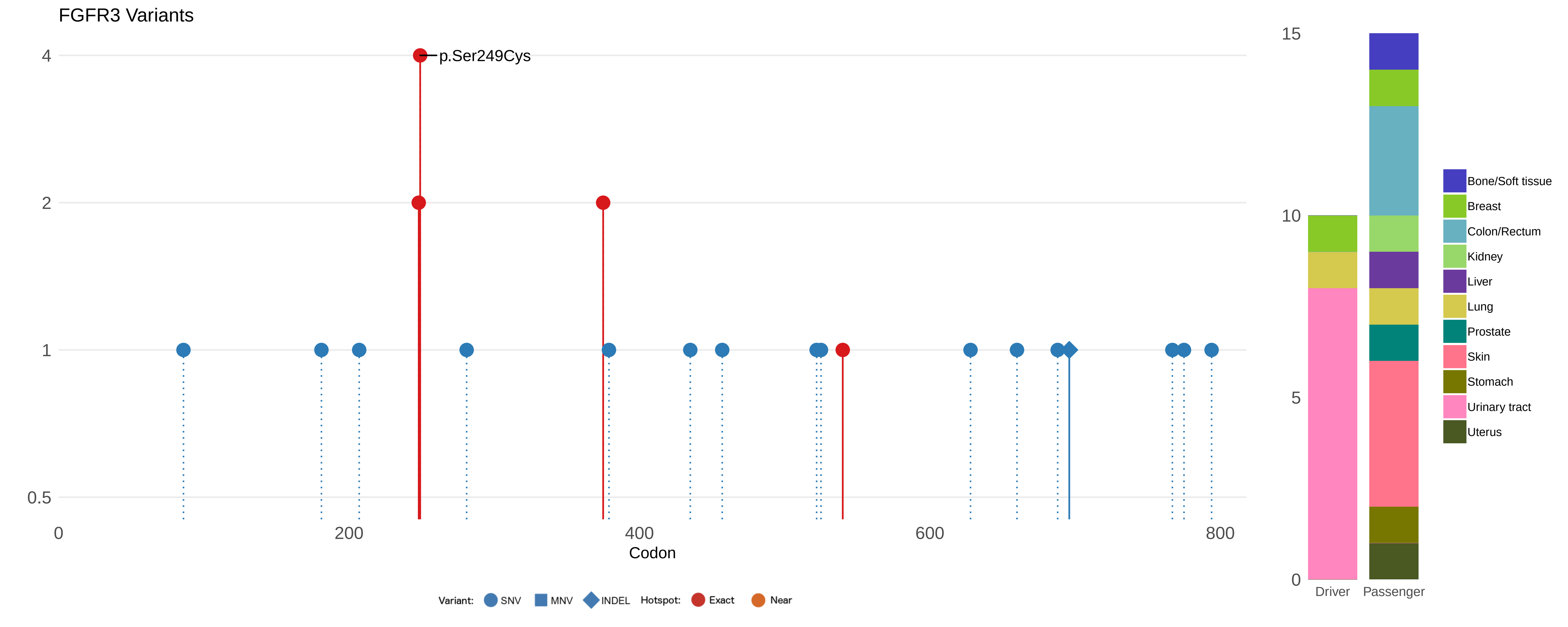

FGFR3 Variants
p.Ser249Cys
4
2
1
0.5
0
200
400
600
800
Codon
Variant:
SNV
MNV
INDEL
Hotspot:
Exact
Near
15
10
5
0
Driver
Passenger
Bone/Soft tissue
Breast
Colon/Rectum
Kidney
Liver
Lung
Prostate
Skin
Stomach
Urinary tract
Uterus

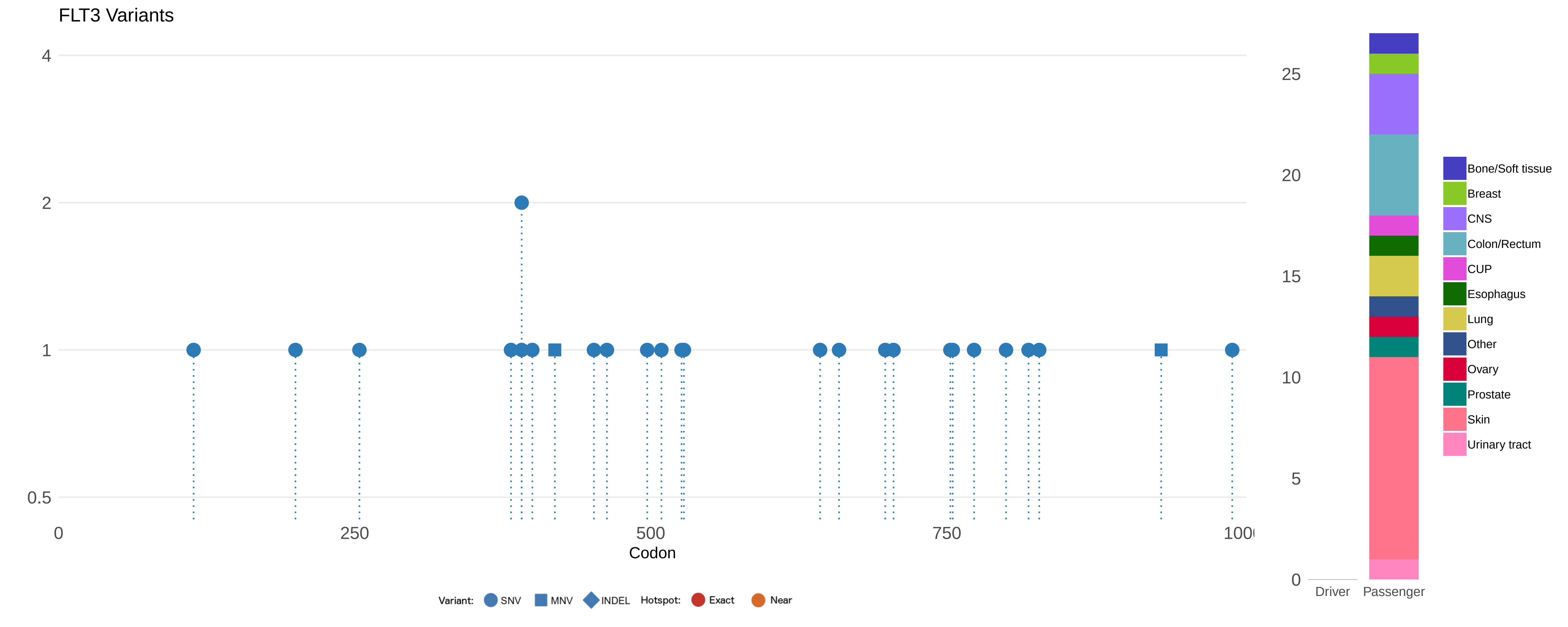

FLT3 Variants
4
2
1
0.5
0
250
500
750
100
Codon
Variant:
SNV
MNV
INDEL
Hotspot:
Exact
Near
25
20
15
10
5
0
Driver
Passenger
Bone/Soft tissue
Breast
CNS
Colon/Rectum
CUP
Esophagus
Lung
Other
Ovary
Prostate
Skin
Urinary tract

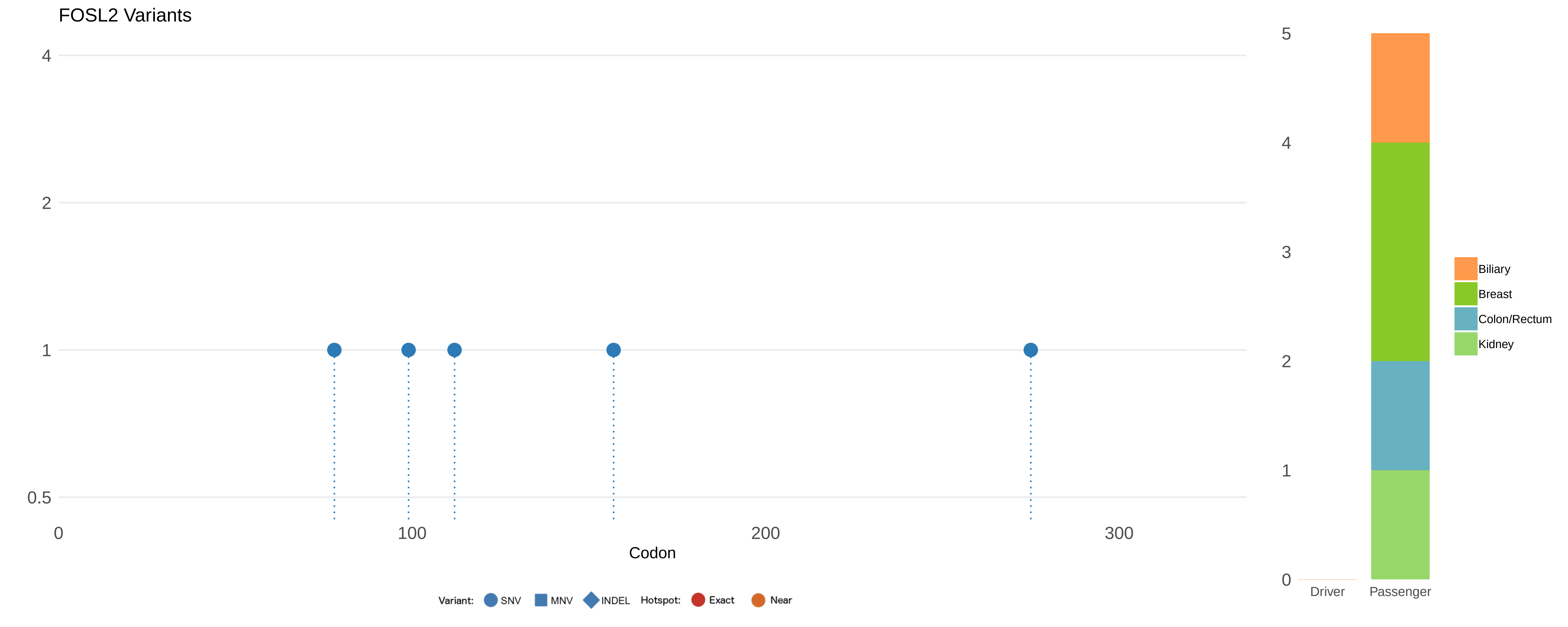
FOSL2 Variants
4
2
1
0.5
0
100
200
300
Codon
Variant: SNV MNV INDEL Hotspot: Exact Near
5
4
3
2
1
0
Driver
Passenger
Biliary
Breast
Colon/Rectum
Kidney

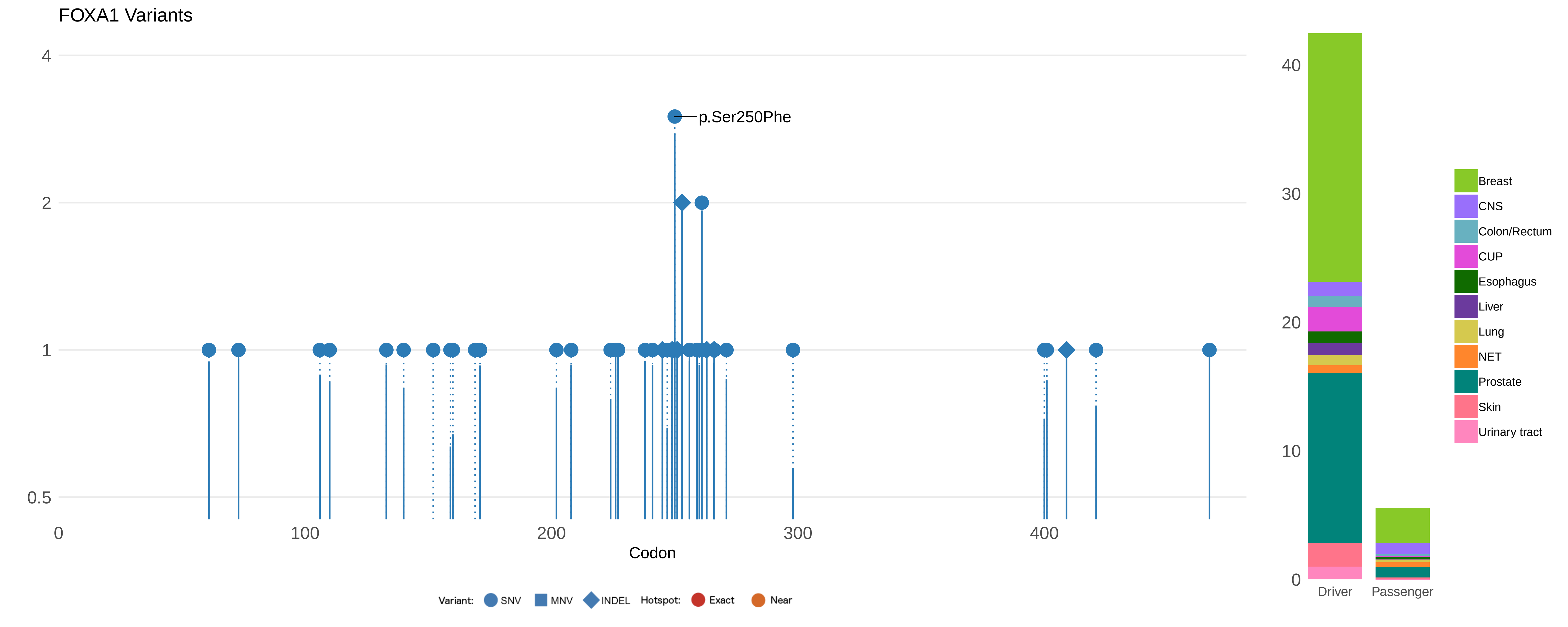

FOXA1 Variants
p.Ser250Phe
4
2
1
0.5
0
100
200
300
400
Codon
Variant: SNV MNV INDEL Hotspot: Exact Near
40
30
20
10
0
Driver
Passenger
Breast
CNS
Colon/Rectum
CUP
Esophagus
Liver
Lung
NET
Prostate
Skin
Urinary tract

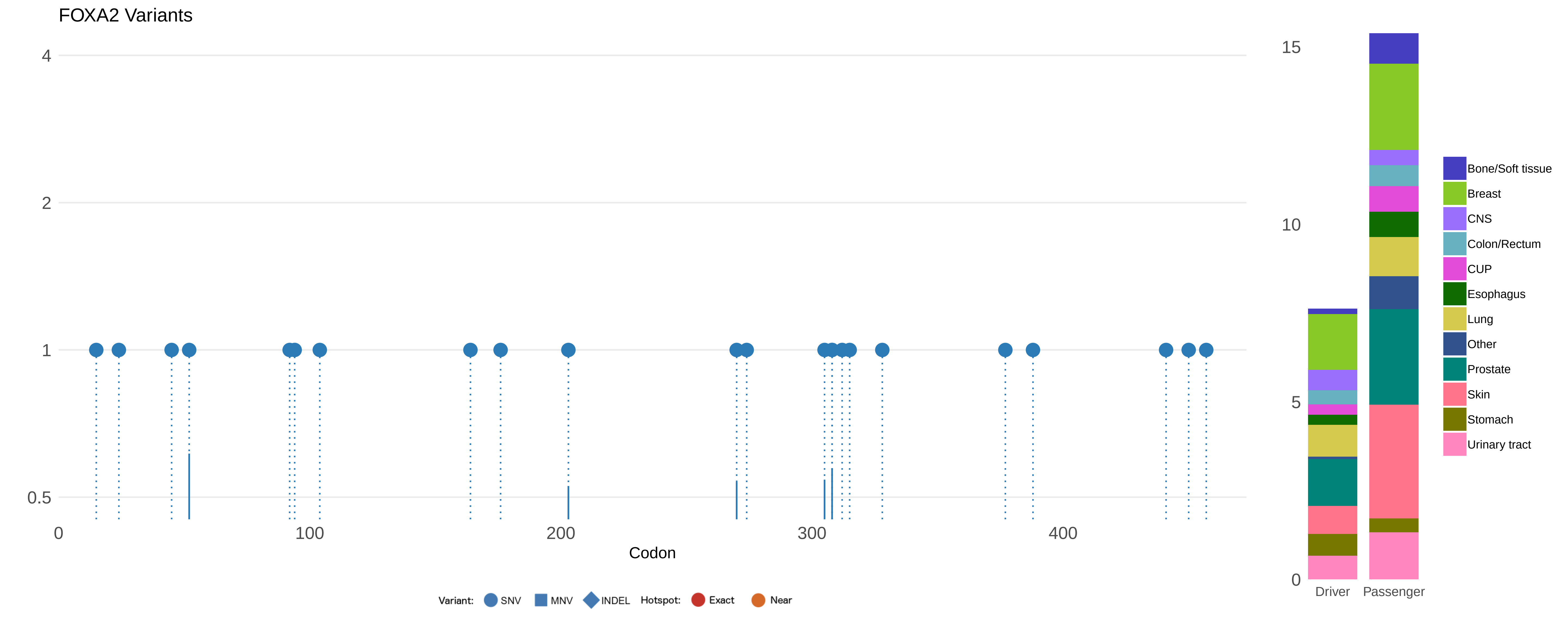

FOXA2 Variants
4
2
1
0.5
0
100
200
300
400
Codon
Variant:
SNV
MNV
INDEL
Hotspot:
Exact
Near
15
10
5
0
Driver
Passenger
Bone/Soft tissue
Breast
CNS
Colon/Rectum
CUP
Esophagus
Lung
Other
Prostate
Skin
Stomach
Urinary tract

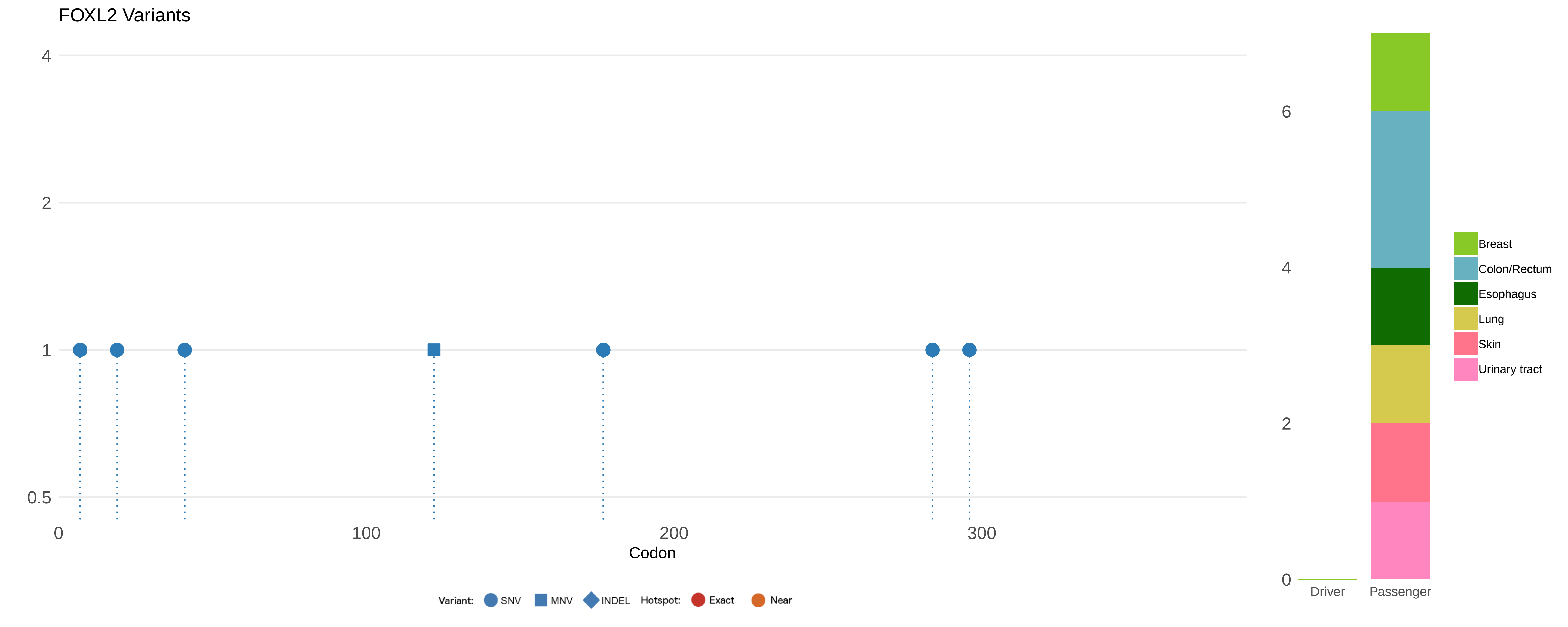

FOXL2 Variants
4
2
1
0.5
0
100
200
300
Codon
Variant: SNV MNV INDEL Hotspot: Exact Near
6
4
2
0
Driver
Passenger
Breast
Colon/Rectum
Esophagus
Lung
Skin
Urinary tract

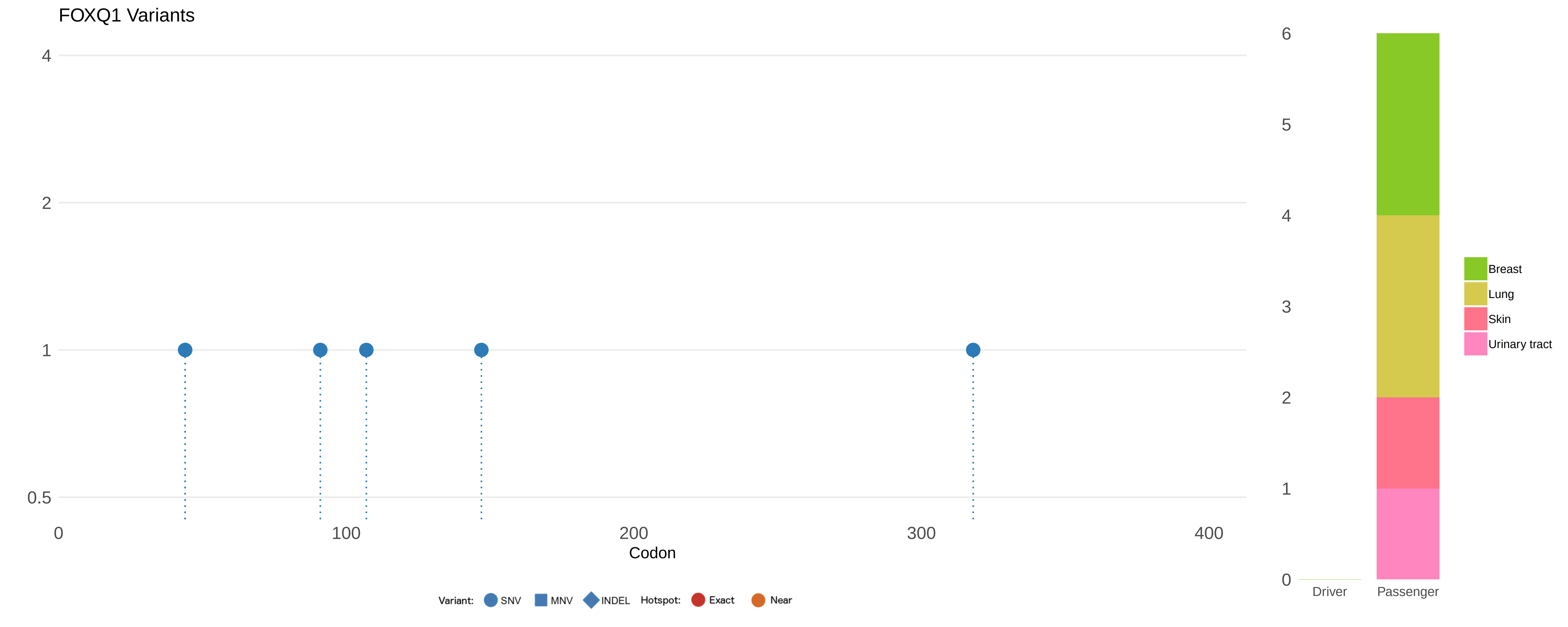

FOXQ1 Variants
4
2
1
0.5
0
100
200
300
400
Codon
Variant:
SNV
MNV
INDEL
Hotspot:
Exact
Near
6
5
4
3
2
1
0
Driver
Passenger
Breast
Lung
Skin
Urinary tract

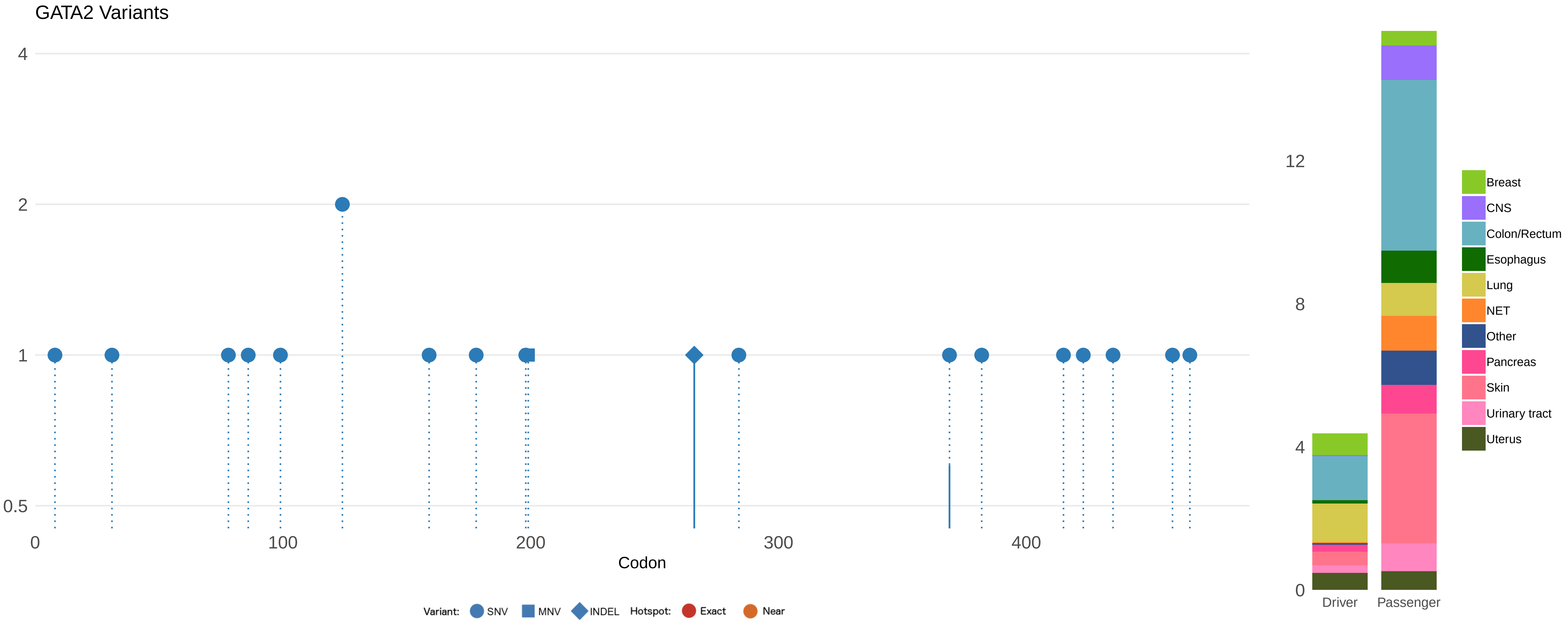

GATA2 Variants
4
2
1
0.5
0
100
200
300
400
Codon
Variant:
SNV
MNV
INDEL
Hotspot:
Exact
Near
12
8
4
0
Driver
Passenger
Breast
CNS
Colon/Rectum
Esophagus
Lung
NET
Other
Pancreas
Skin
Urinary tract
Uterus

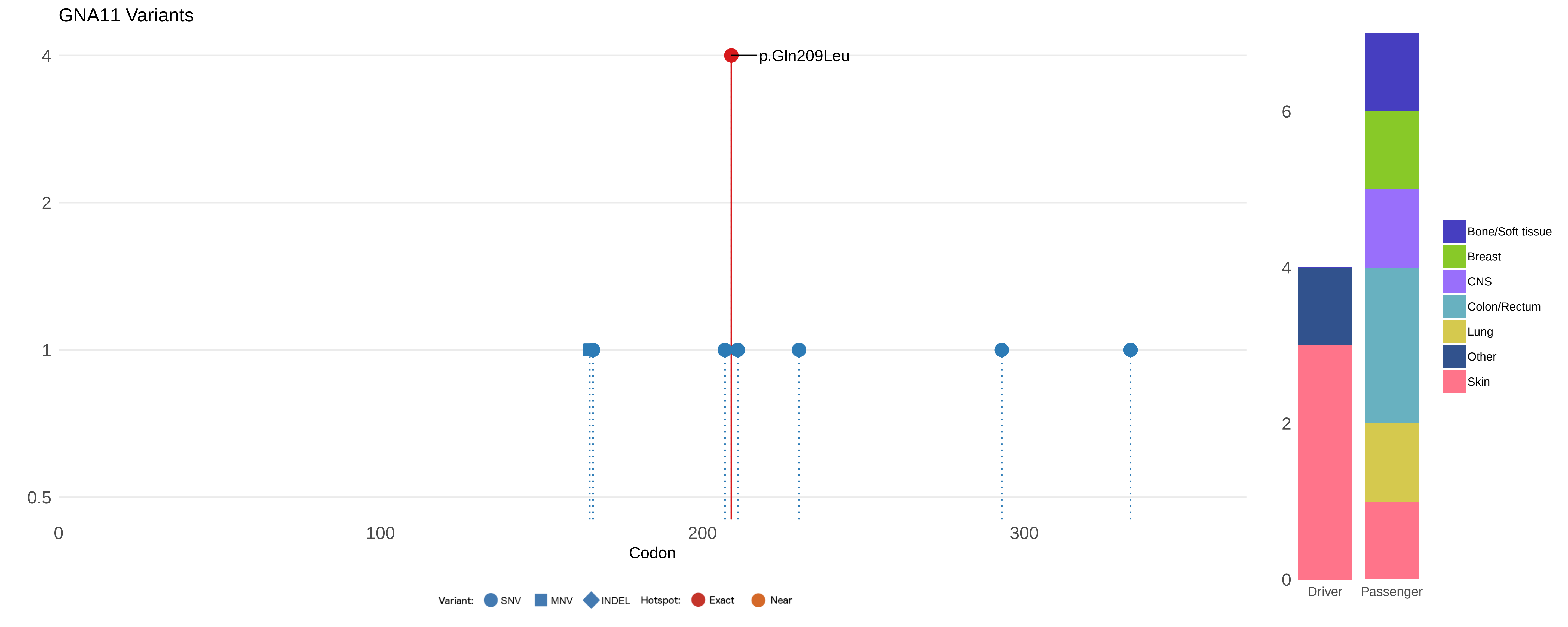
GNA11 Variants
p.Gln209Leu
4
2
1
0.5
0
100
200
300
Codon
Variant:
SNV
MNV
INDEL
Hotspot:
Exact
Near
6
4
2
0
Driver
Passenger
Bone/Soft tissue
Breast
CNS
Colon/Rectum
Lung
Other
Skin

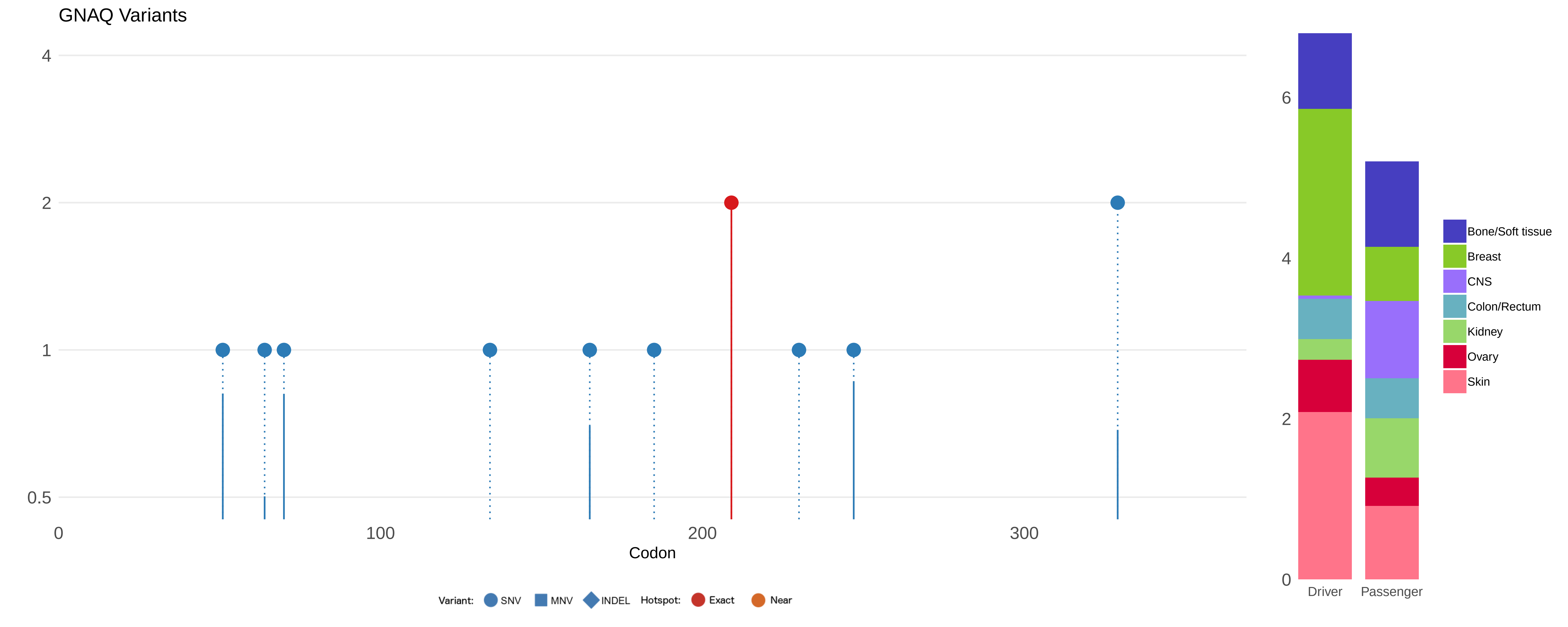

GNAQ Variants
4
2
1
0.5
0
100
200
300
Codon
Variant: SNV MNV INDEL Hotspot: Exact Near
6
4
2
0
Driver
Passenger
Bone/Soft tissue
Breast
CNS
Colon/Rectum
Kidney
Ovary
Skin

GNAS Variants

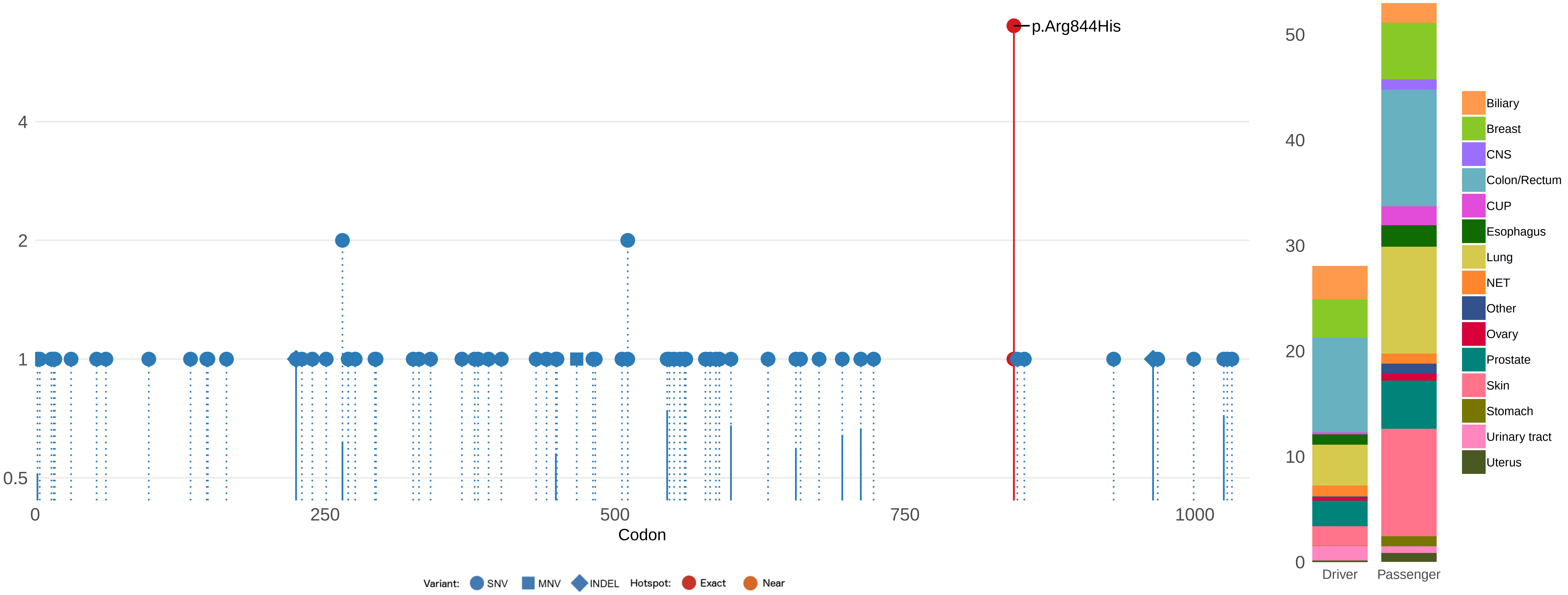

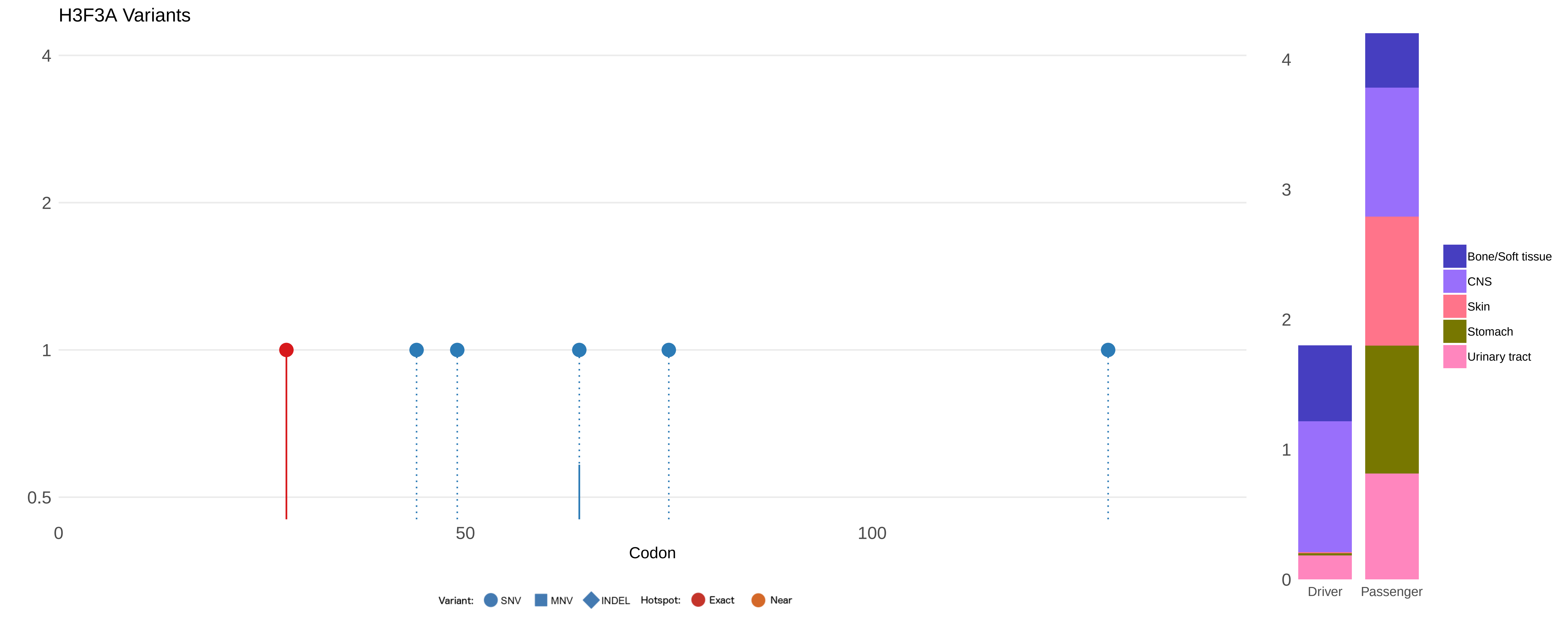
H3F3A Variants
4
2
1
0.5
0
50
100
Codon
Variant: SNV MNV INDEL Hotspot: Exact Near
4
3
2
1
0
Driver
Passenger
Bone/Soft tissue
CNS
Skin
Stomach
Urinary tract

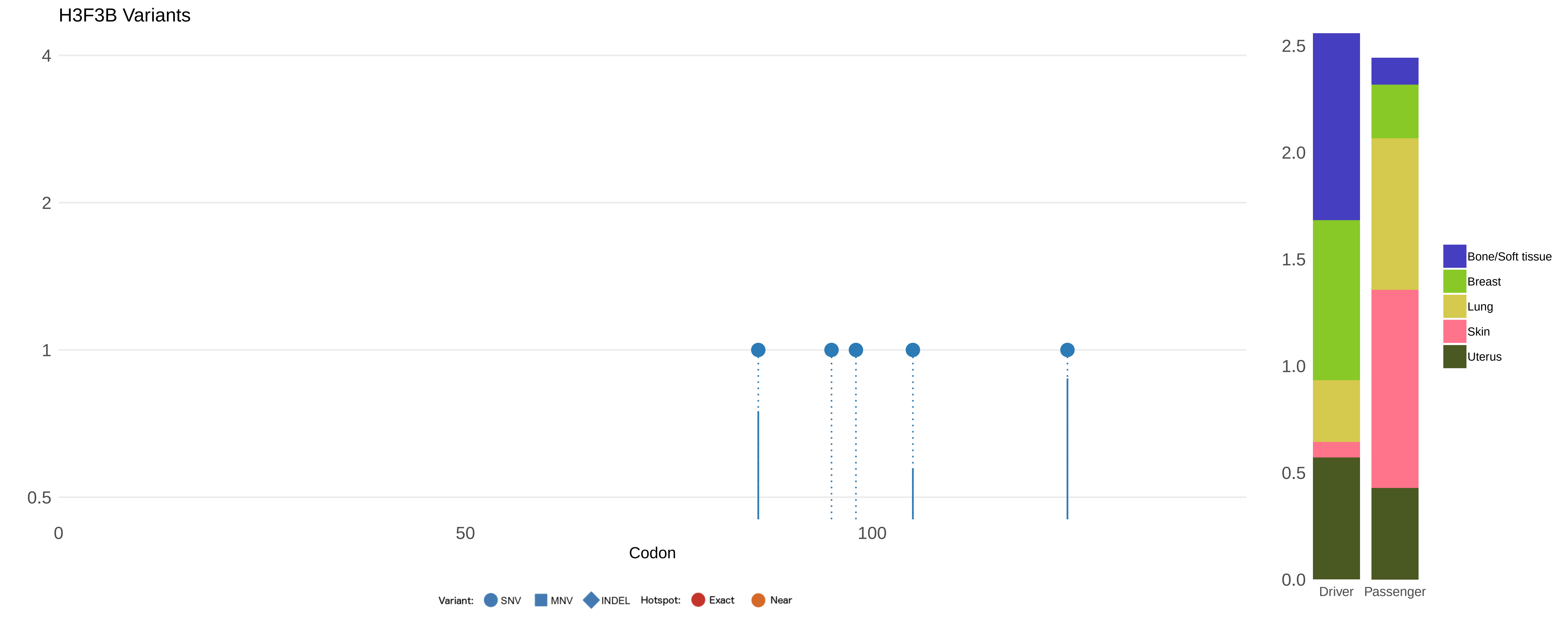
H3F3B Variants
4
2
1
0.5
0
50
100
Codon
Variant:
SNV
MNV
INDEL
Hotspot:
Exact
Near
2.5
2.0
1.5
1.0
0.5
0.0
Driver
Passenger
Bone/Soft tissue
Breast
Lung
Skin
Uterus

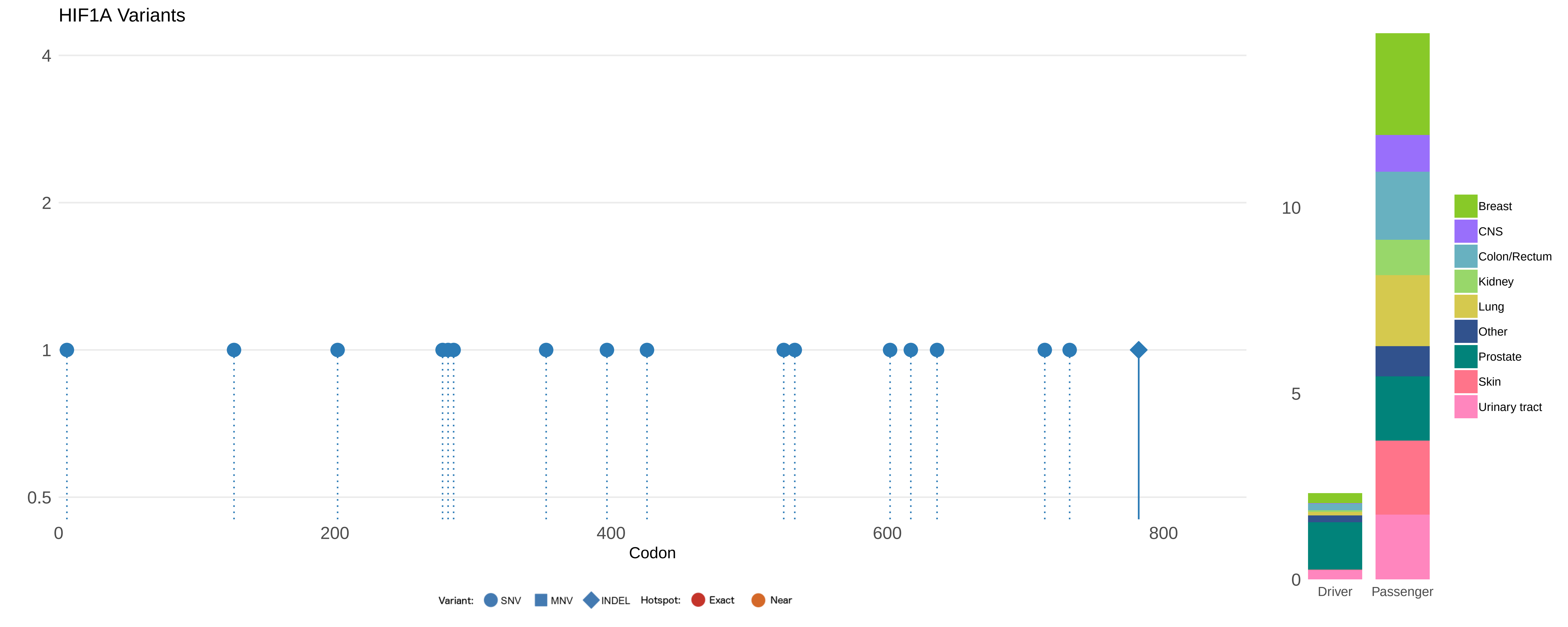
HIF1A Variants
4
2
1
0.5
0
200
400
600
800
Codon
Variant: SNV MNV INDEL Hotspot: Exact Near
10
5
0
Driver
Passenger
Breast
CNS
Colon/Rectum
Kidney
Lung
Other
Prostate
Skin
Urinary tract

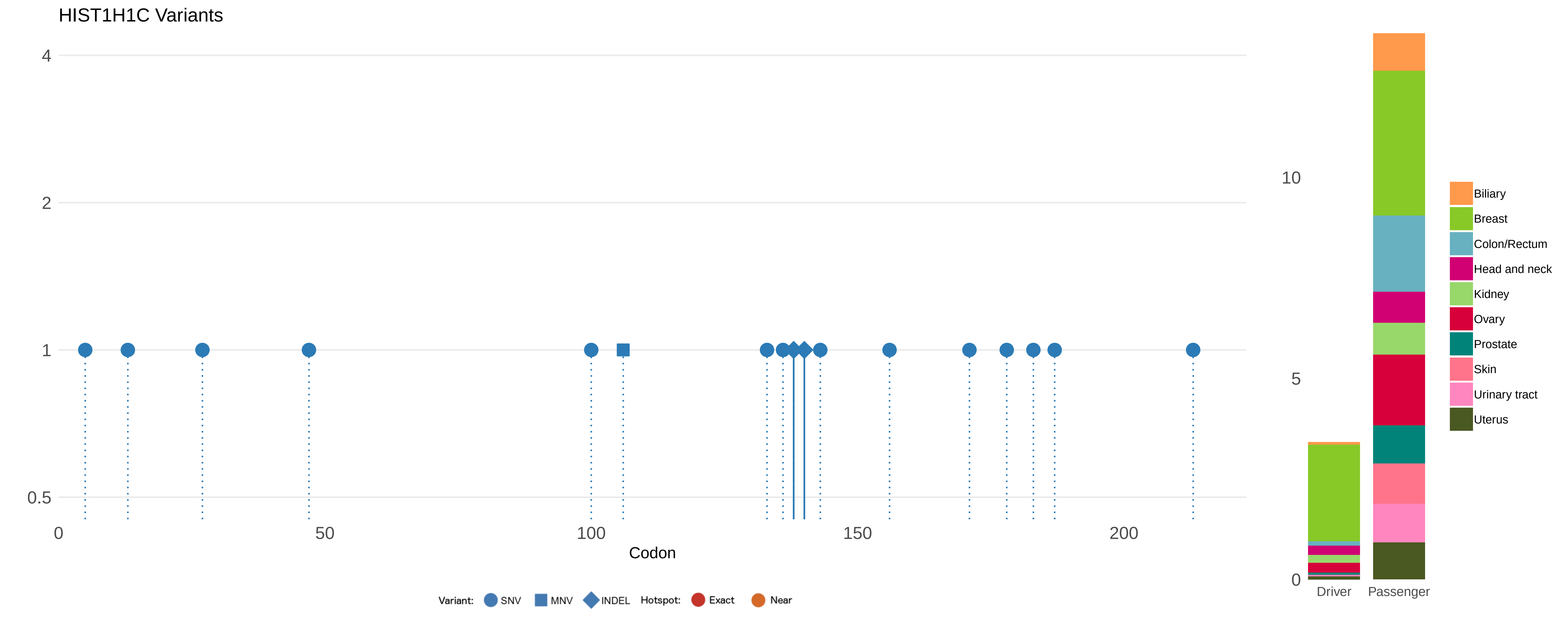

HIST1H1C Variants
4
2
1
0.5
0
50
100
150
200
Codon
Variant: SNV MNV INDEL Hotspot: Exact Near
10
5
0
Driver
Passenger
Biliary
Breast
Colon/Rectum
Head and neck
Kidney
Ovary
Prostate
Skin
Urinary tract
Uterus

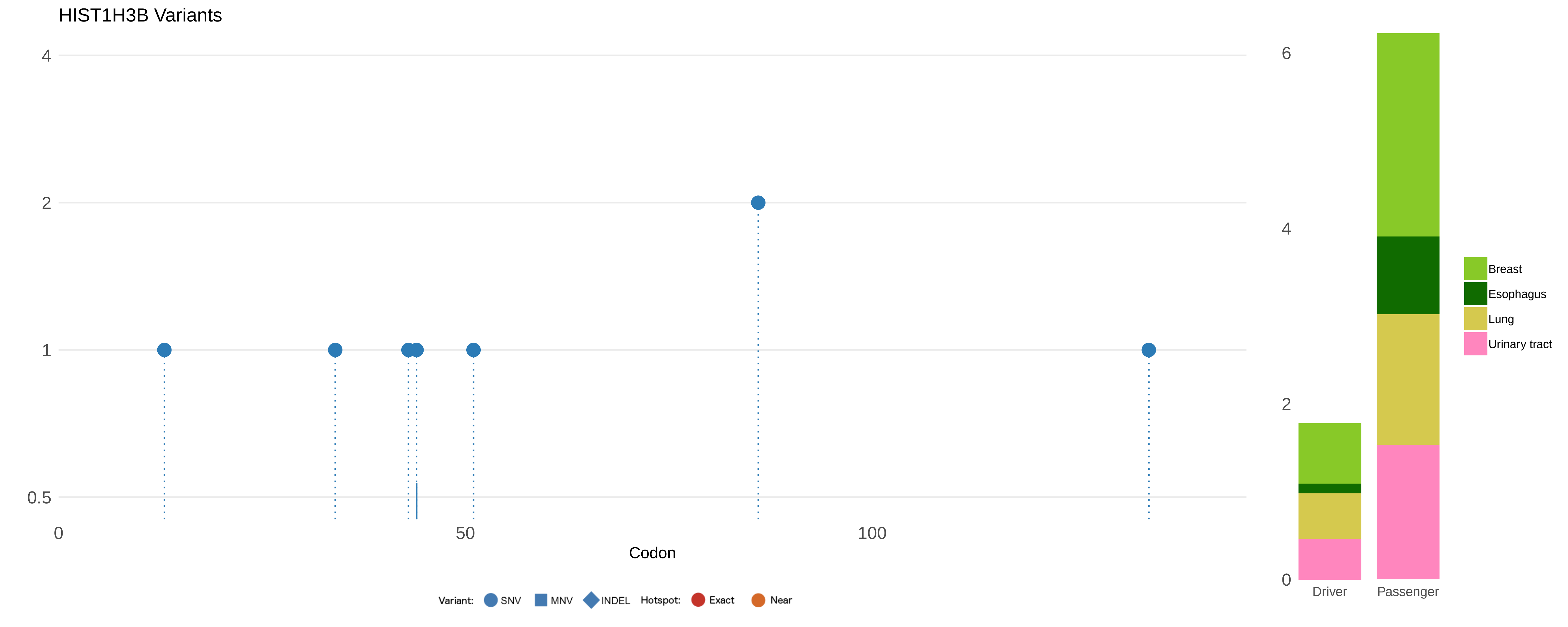

HIST1H3B Variants
4
2
1
0.5
0
50
100
Codon
Variant:
SNV
MNV
INDEL
Hotspot:
Exact
Near
6
4
2
0
Driver
Passenger
Breast
Esophagus
Lung
Urinary tract

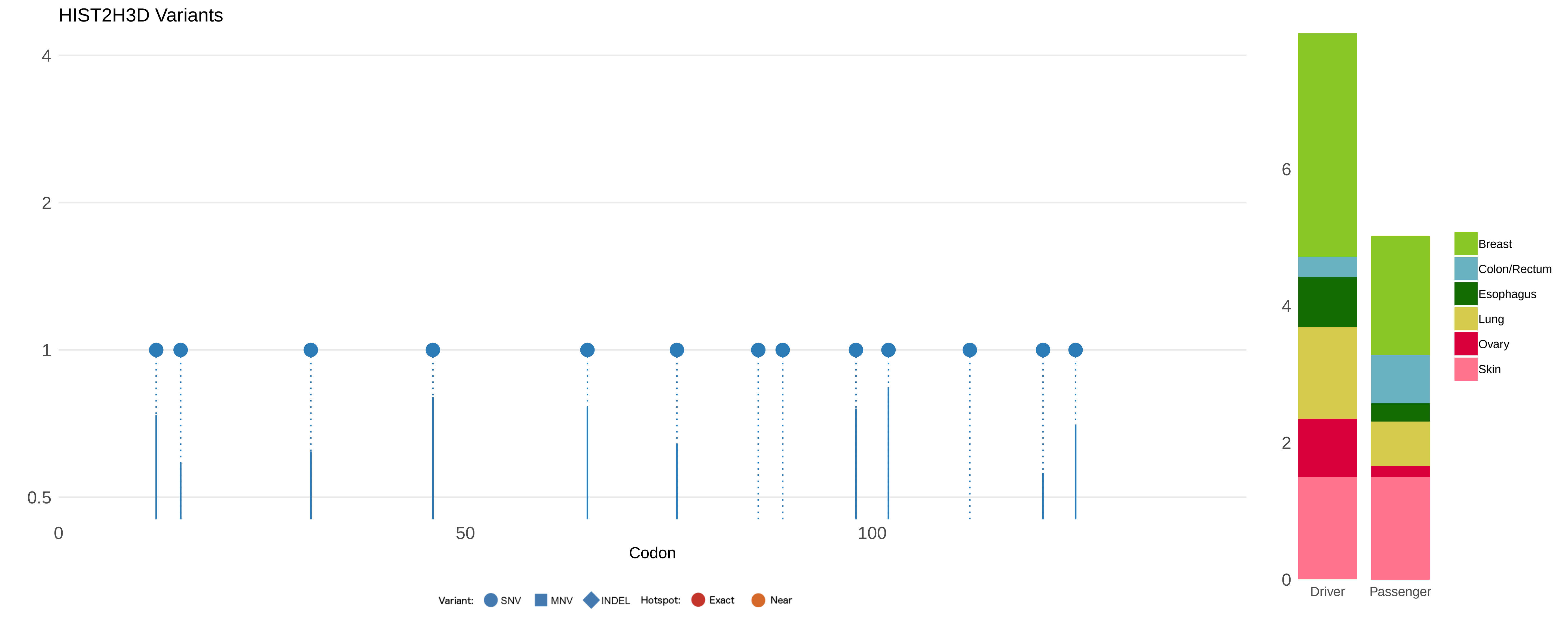

HIST2H3D Variants
4
2
1
0.5
0
50
100
Codon
Variant:
SNV
MNV
INDEL
Hotspot:
Exact
Near
6
4
2
0
Driver
Passenger
Breast
Colon/Rectum
Esophagus
Lung
Ovary
Skin

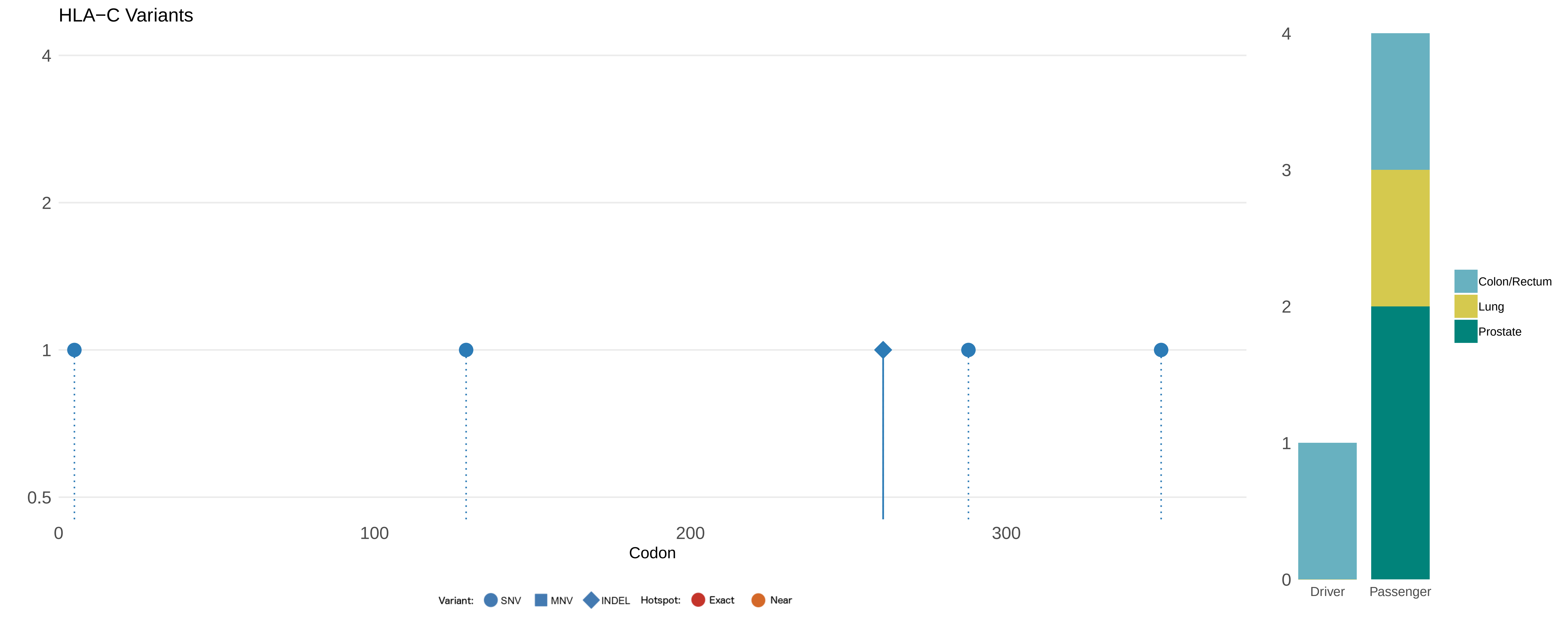

HLA−C Variants
4
2
1
0.5
0
100
200
300
Codon
Variant: SNV MNV INDEL Hotspot: Exact Near
4
3
2
1
0
Driver
Passenger
Colon/Rectum
Lung
Prostate

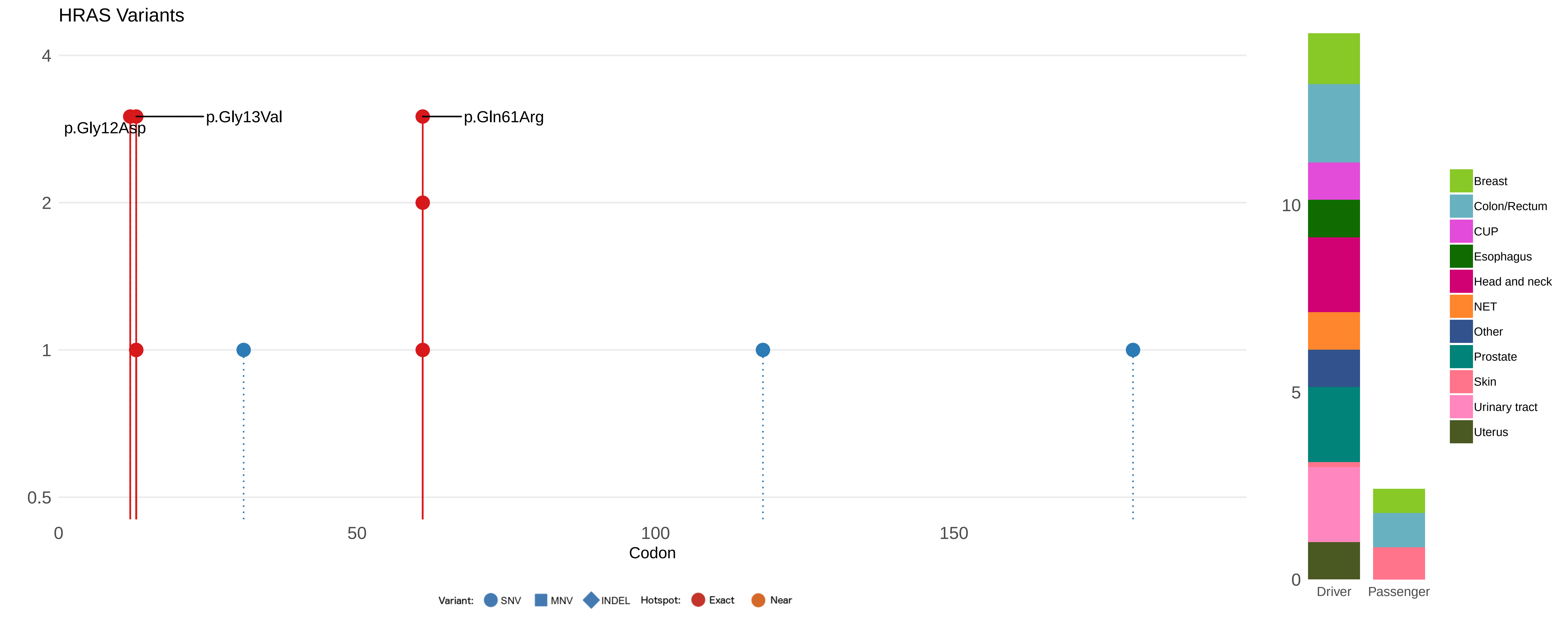

HRAS Variants
p.Gly13Val
p.Gly12Asp
p.Gln61Arg
4
2
1
0.5
0
50
100
150
Codon
Variant: SNV MNV INDEL Hotspot: Exact Near
10
5
0
Driver
Passenger
Breast
Colon/Rectum
CUP
Esophagus
Head and neck
NET
Other
Prostate
Skin
Urinary tract
Uterus

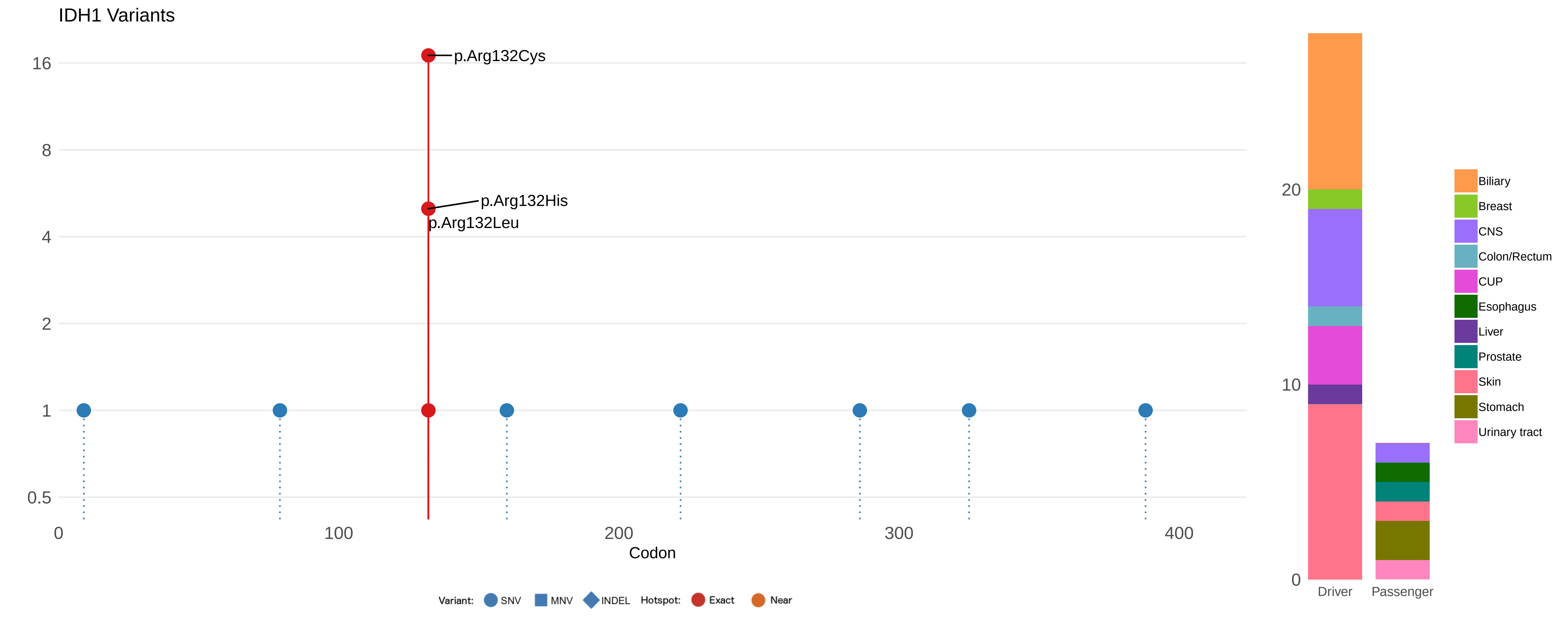

IDH1 Variants
p.Arg132Cys
p.Arg132His
p.Arg132Leu
16
8
4
2
1
0.5
0
100
200
300
400
Codon
Variant: SNV MNV INDEL Hotspot: Exact Near
20
10
0
Driver
Passenger
Biliary
Breast
CNS
Colon/Rectum
CUP
Esophagus
Liver
Prostate
Skin
Stomach
Urinary tract

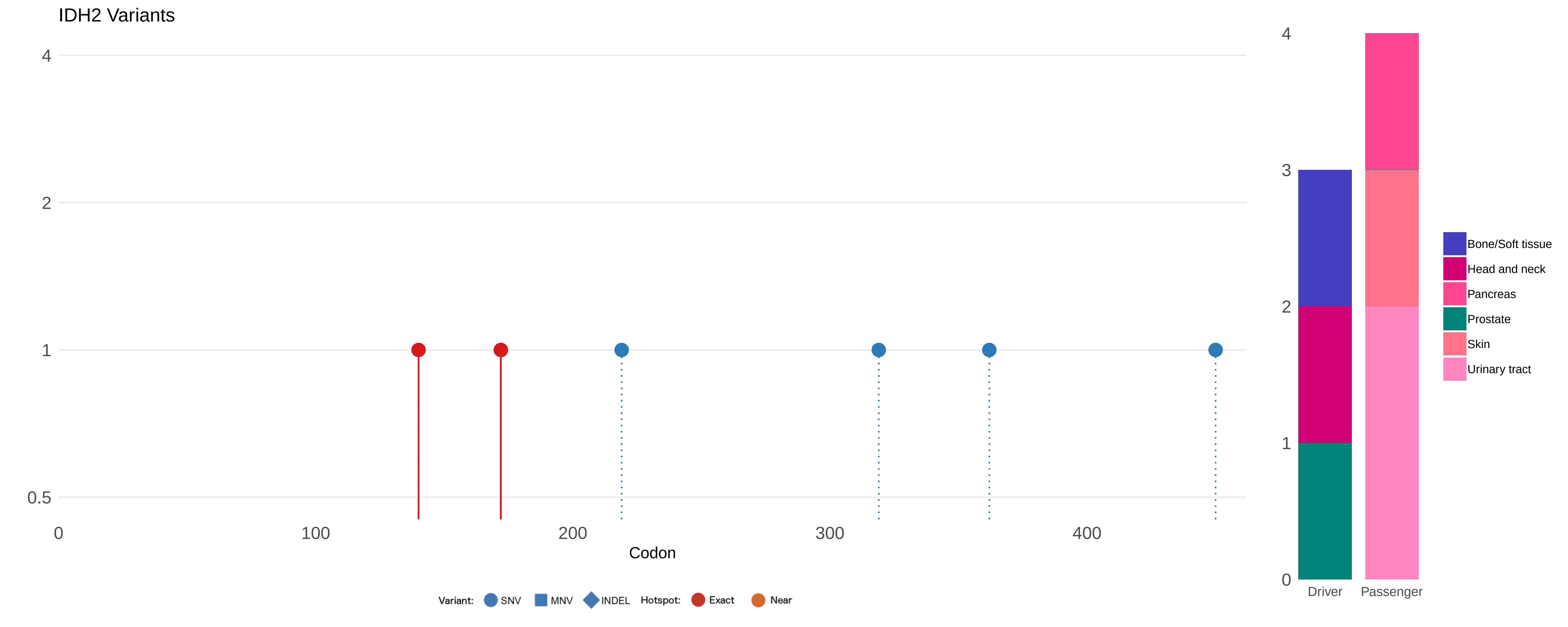

IDH2 Variants
4
2
1
0.5
0
100
200
300
400
Codon
Variant: SNV MNV INDEL Hotspot: Exact Near
4
3
2
1
0
Driver
Passenger
Bone/Soft tissue
Head and neck
Pancreas
Prostate
Skin
Urinary tract

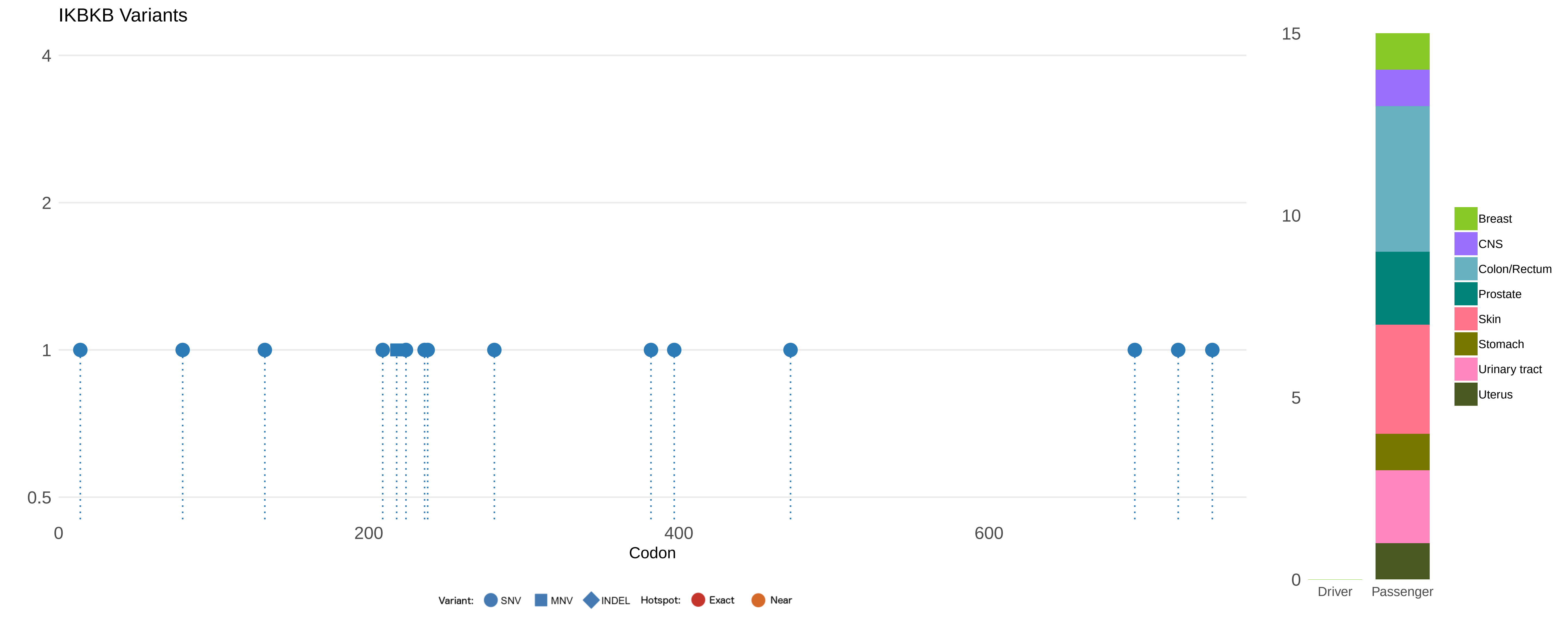

IKBKB Variants
4
2
1
0.5
0
200
400
600
Codon
Variant: SNV MNV INDEL Hotspot: Exact Near
15
10
5
0
Driver
Passenger
Breast
CNS
Colon/Rectum
Prostate
Skin
Stomach
Urinary tract
Uterus

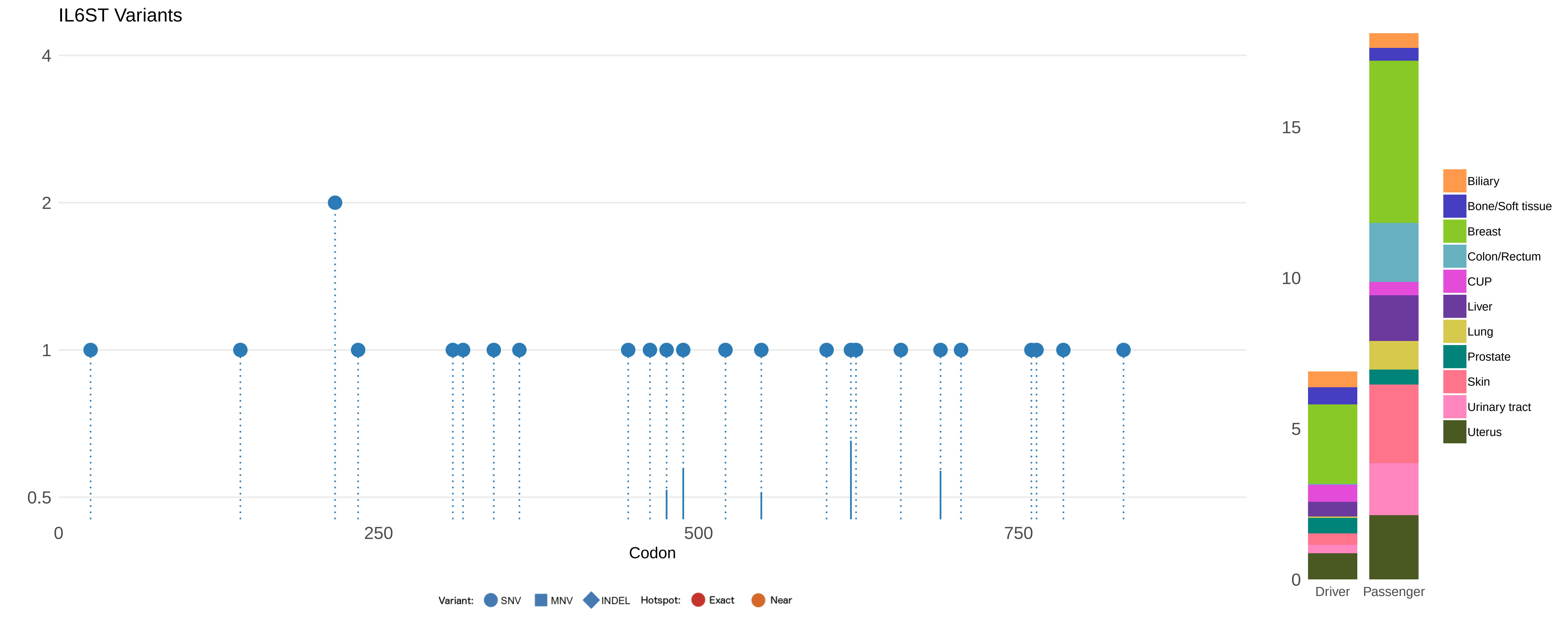

IL6ST Variants
4
2
1
0.5
0
250
500
750
Codon
Variant: SNV MNV INDEL Hotspot: Exact Near
15
10
5
0
Driver
Passenger
Biliary
Bone/Soft tissue
Breast
Colon/Rectum
CUP
Liver
Lung
Prostate
Skin
Urinary tract
Uterus

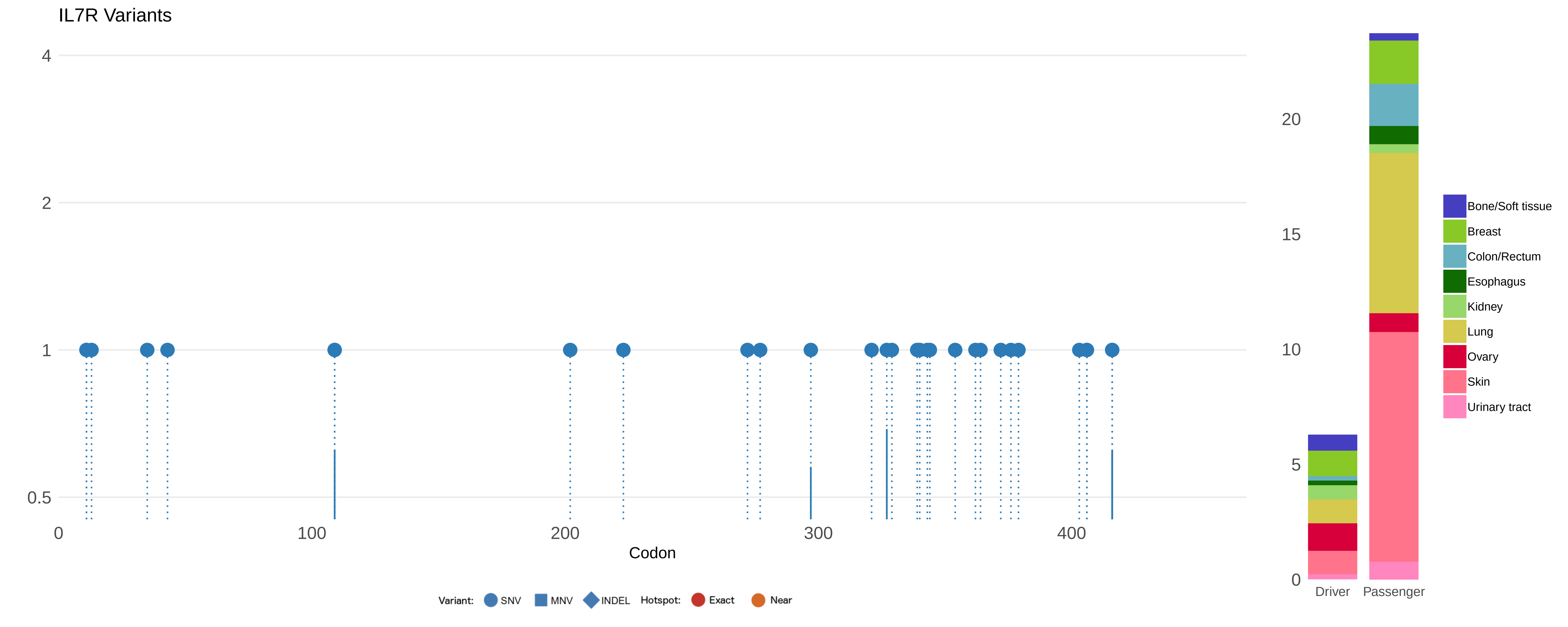
IL7R Variants
4
2
1
0.5
0
100
200
300
400
Codon
Variant: SNV MNV INDEL Hotspot: Exact Near
20
15
10
5
0
Driver
Passenger
Bone/Soft tissue
Breast
Colon/Rectum
Esophagus
Kidney
Lung
Ovary
Skin
Urinary tract

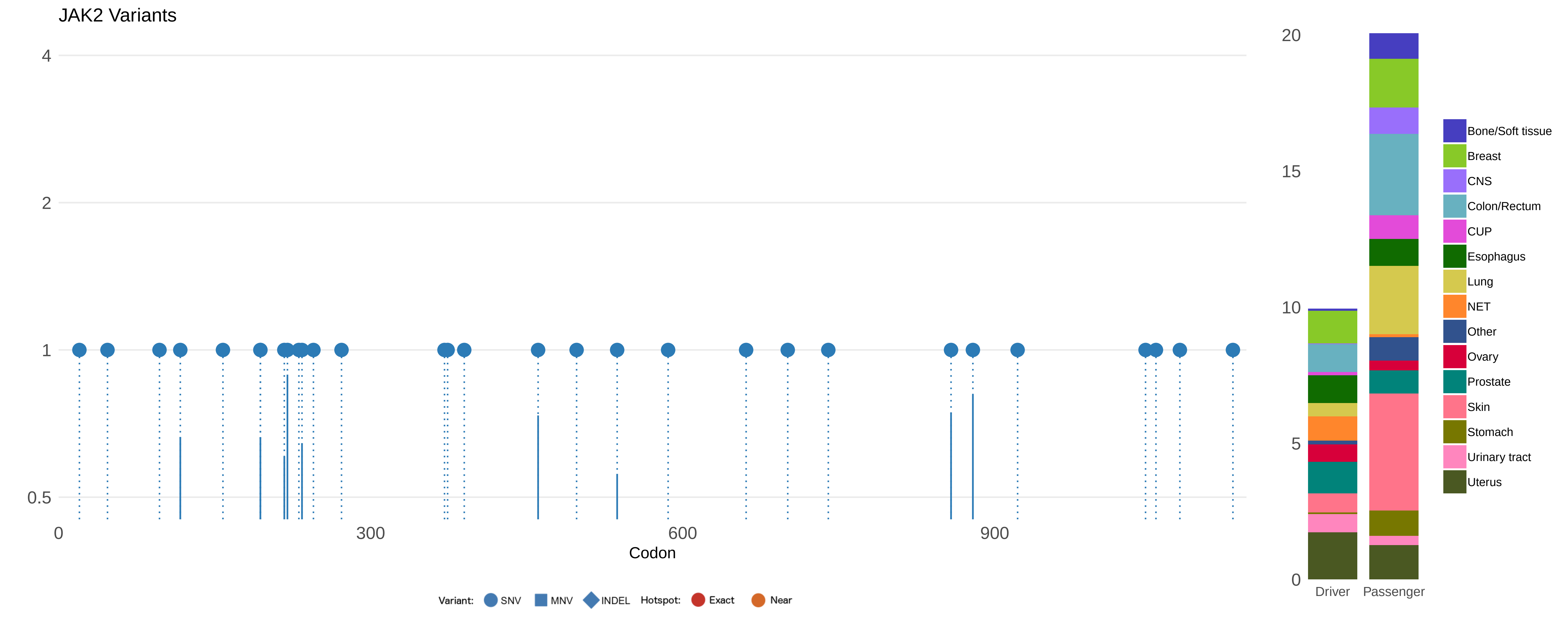
JAK2 Variants
4
2
1
0.5
0
300
600
900
Codon
Variant: SNV MNV INDEL Hotspot: Exact Near
20
15
10
5
0
Driver
Passenger
Bone/Soft tissue
Breast
CNS
Colon/Rectum
CUP
Esophagus
Lung
NET
Other
Ovary
Prostate
Skin
Stomach
Urinary tract
Uterus

JAK3 Variants

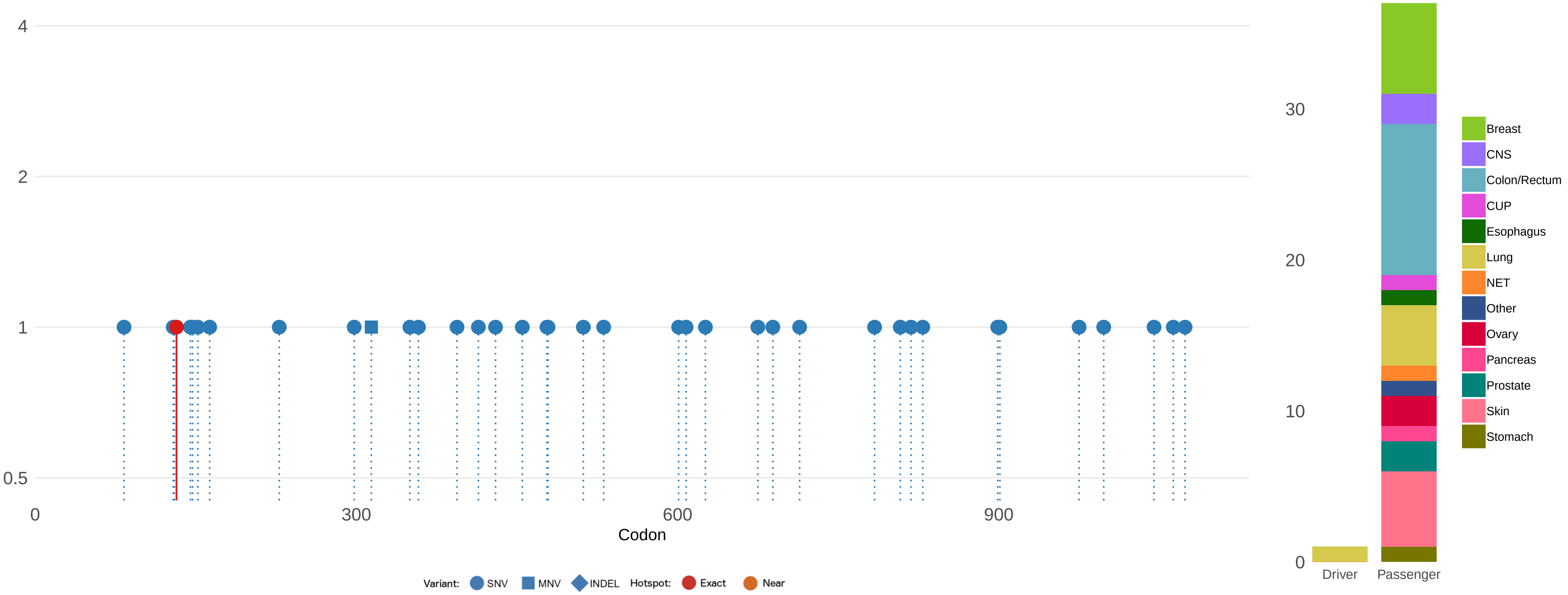

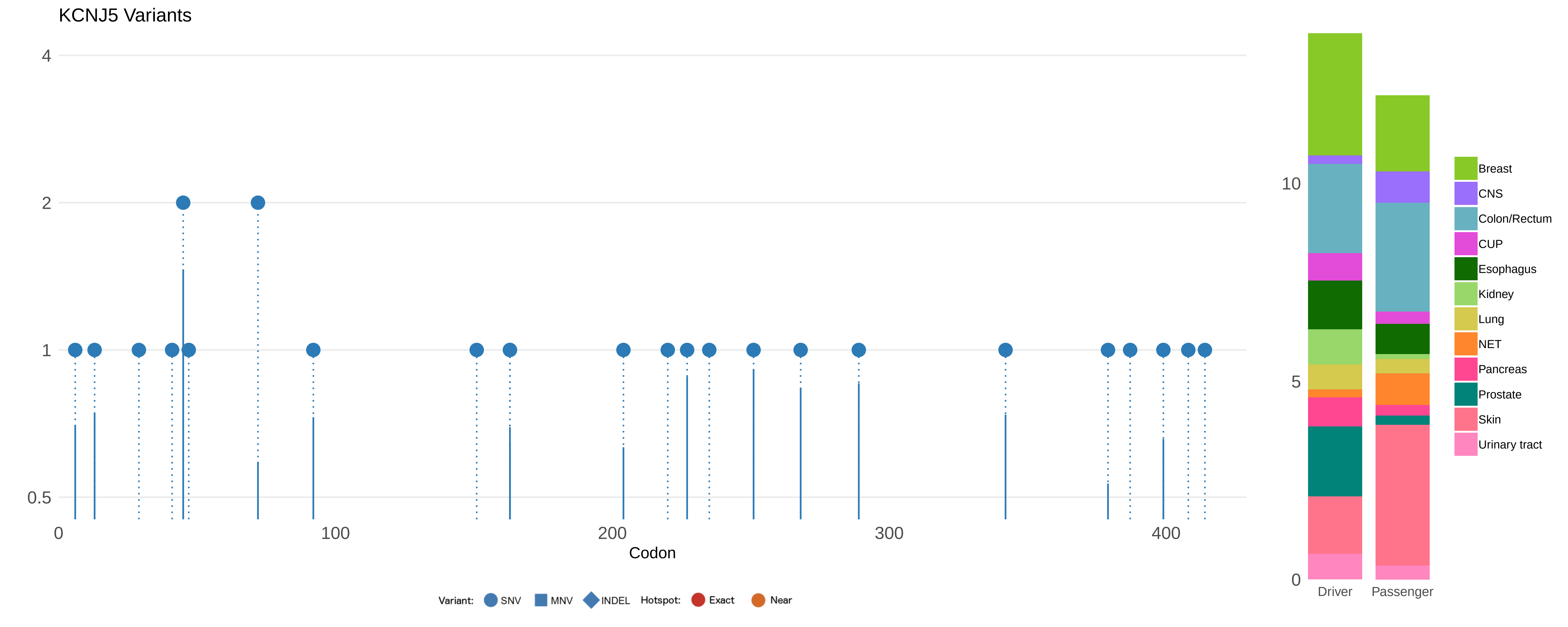
KCNJ5 Variants
4
2
1
0.5
0
100
200
300
400
Codon
Variant: SNV MNV INDEL Hotspot: Exact Near
10
5
0
Driver
Passenger
Breast
CNS
Colon/Rectum
CUP
Esophagus
Kidney
Lung
NET
Pancreas
Prostate
Skin
Urinary tract

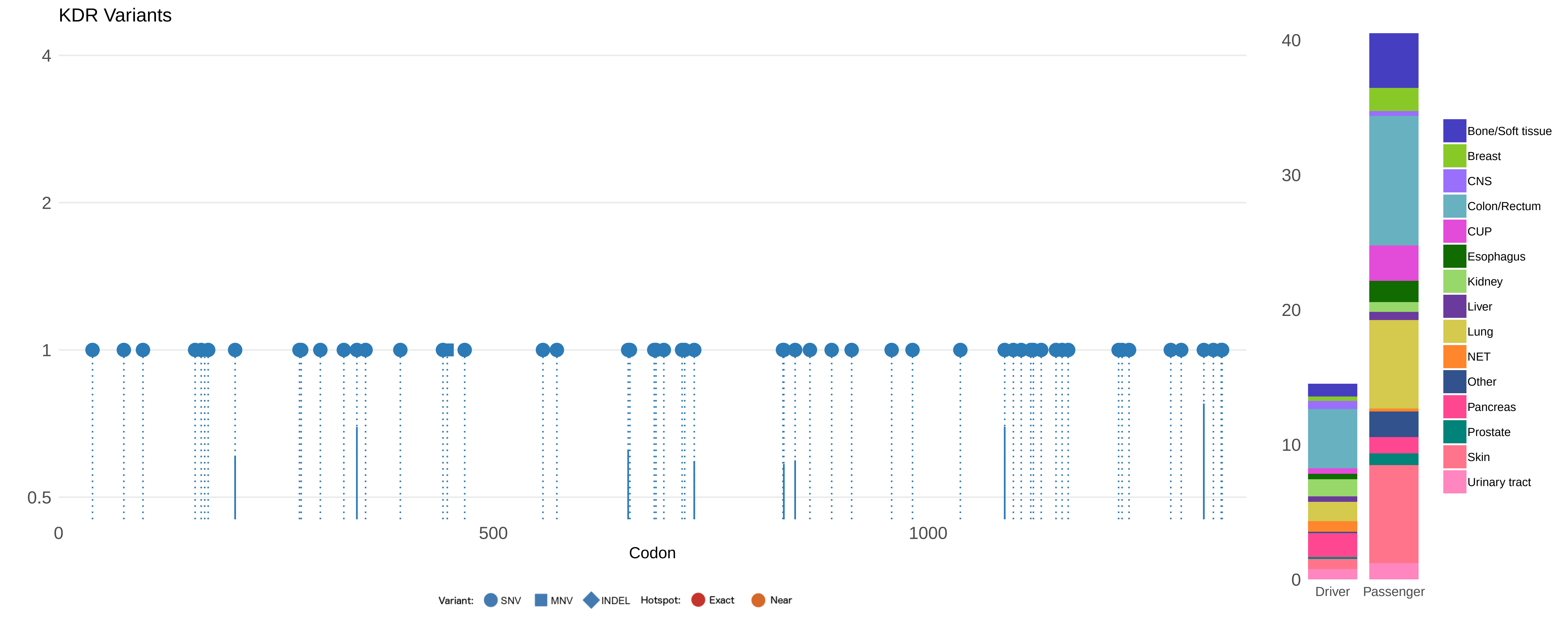

KDR Variants
4
2
1
0.5
0
500
1000
Codon
Variant: SNV MNV INDEL Hotspot: Exact Near
40
30
20
10
0
Driver
Passenger
Bone/Soft tissue
Breast
CNS
Colon/Rectum
CUP
Esophagus
Kidney
Liver
Lung
NET
Other
Pancreas
Prostate
Skin
Urinary tract

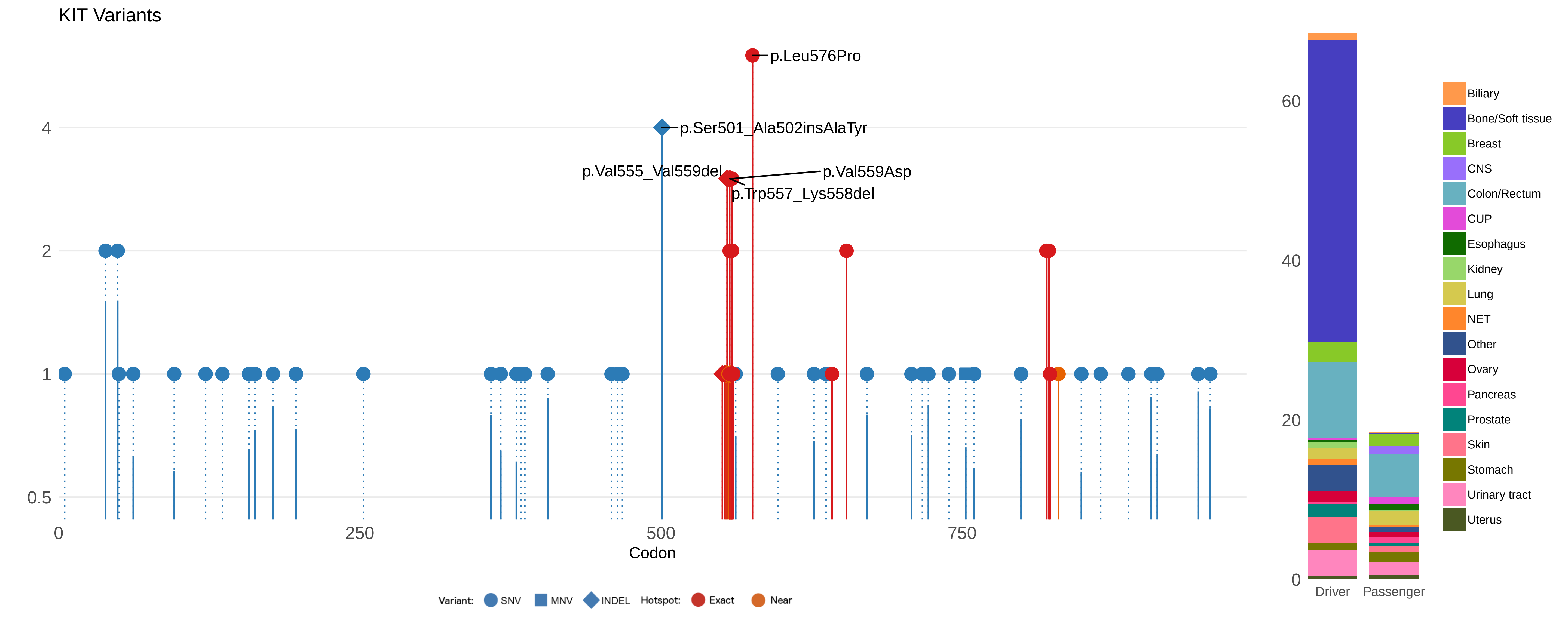
KIT Variants
p.Leu576Pro
p.Ser501_Ala502insAlaTyr
p.Val555_Val559del
p.Val559Asp
p.Trp557_Lys558del
4
2
1
0.5
0
250
500
750
Codon
Variant: SNV MNV INDEL Hotspot: Exact Near
60
40
20
0
Driver
Passenger
Biliary
Bone/Soft tissue
Breast
CNS
Colon/Rectum
CUP
Esophagus
Kidney
Lung
NET
Other
Ovary
Pancreas
Prostate
Skin
Stomach
Urinary tract
Uterus

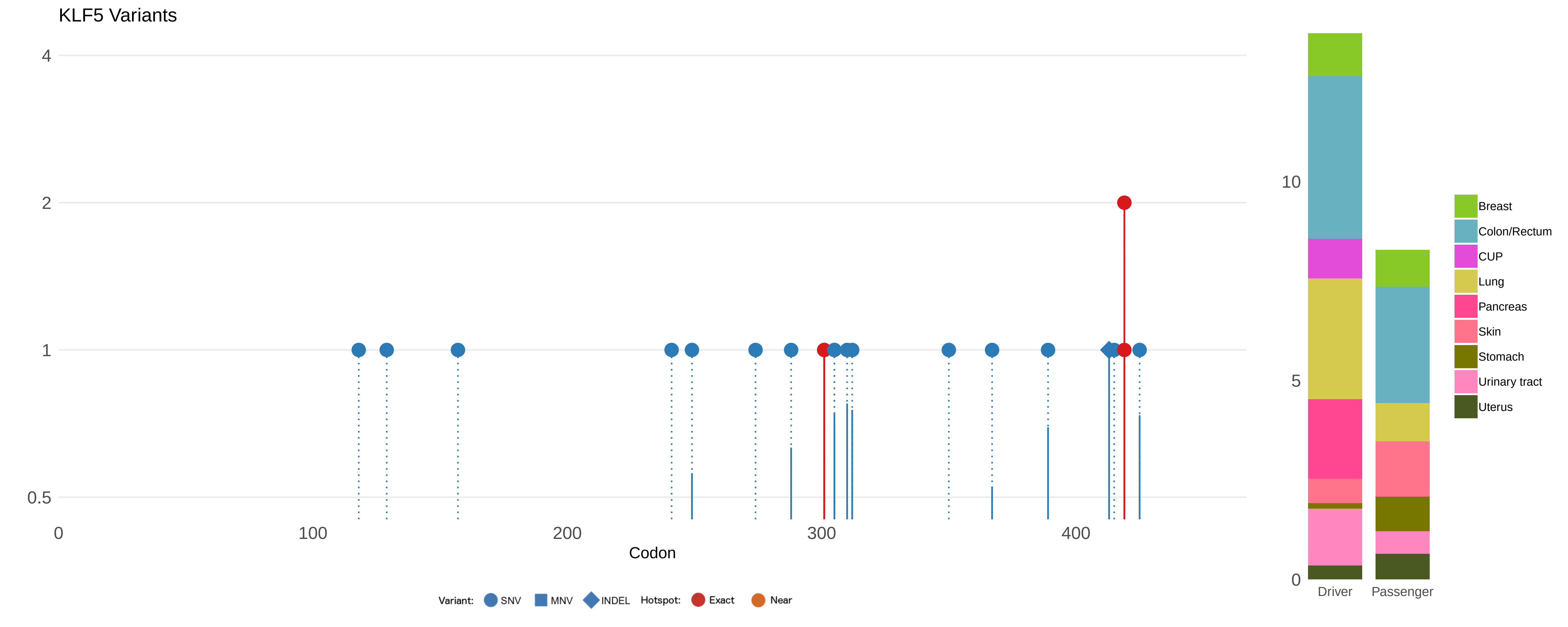
KLF5 Variants
4
2
1
0.5
0
100
200
300
400
Codon
Variant:
SNV
MNV
INDEL
Hotspot:
Exact
Near
10
5
0
Driver
Passenger
Breast
Colon/Rectum
CUP
Lung
Pancreas
Skin
Stomach
Urinary tract
Uterus

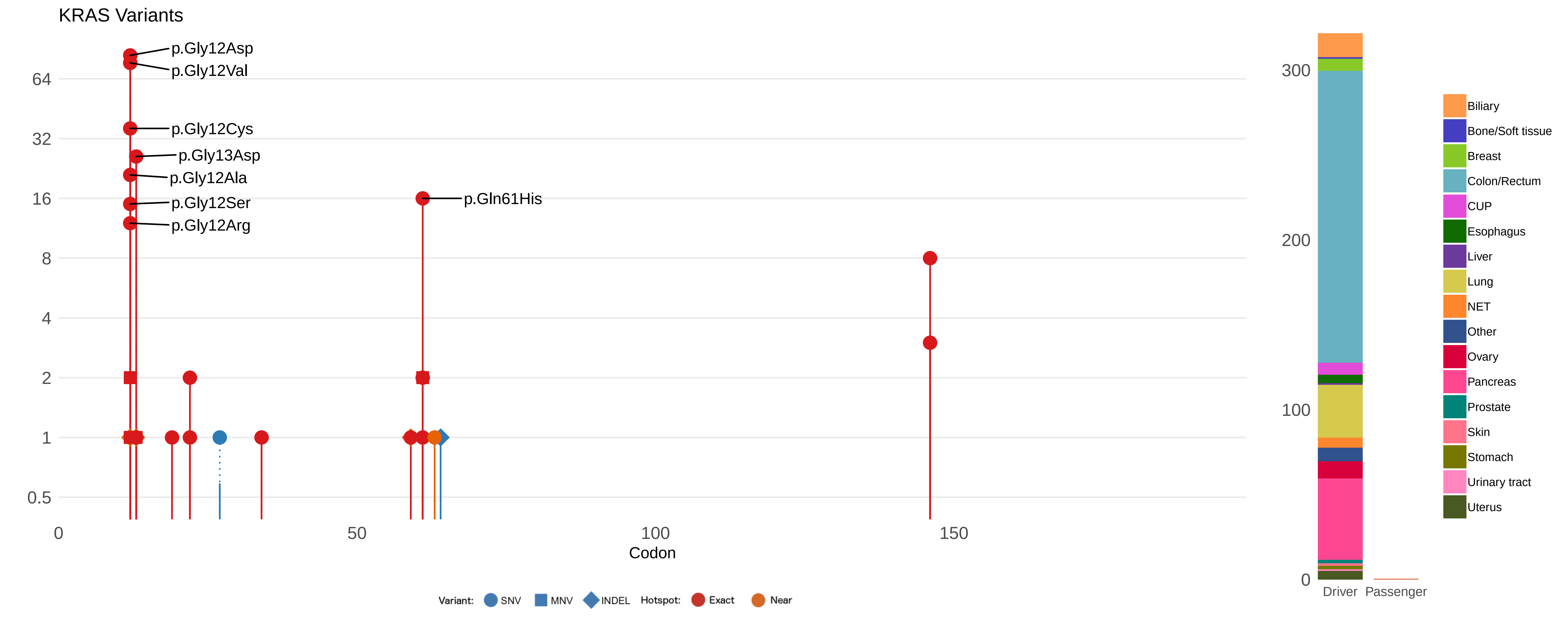

KRAS Variants
p.Gly12Asp
p.Gly12Val
p.Gly12Cys
p.Gly13Asp
p.Gly12Ala
p.Gly12Ser
p.Gly12Arg
p.Gln61His
64
32
16
8
4
2
1
0.5
0
50
100
150
Codon
Variant: SNV MNV INDEL Hotspot: Exact Near
300
200
100
0
Driver
Passenger
Biliary
Bone/Soft tissue
Breast
Colon/Rectum
CUP
Esophagus
Liver
Lung
NET
Other
Ovary
Pancreas
Prostate
Skin
Stomach
Urinary tract
Uterus

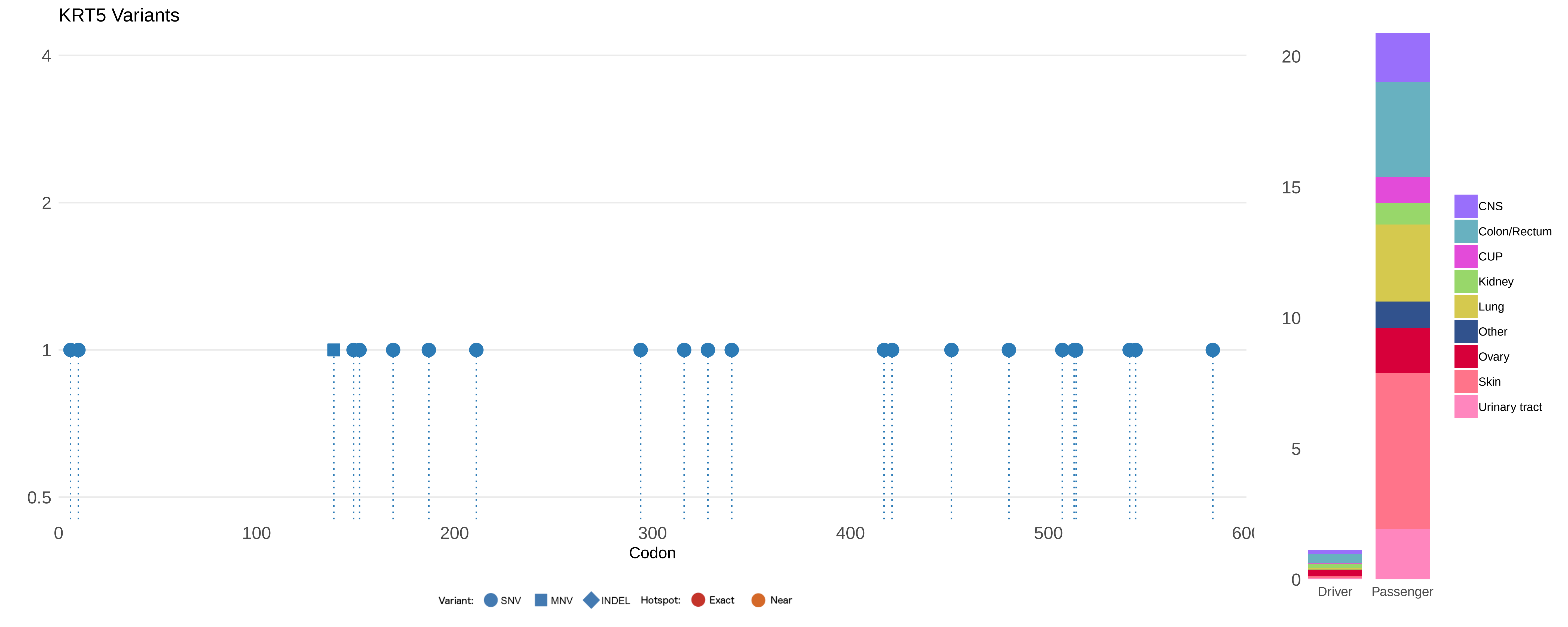

KRT5 Variants
4
2
1
0.5
0
100
200
300
400
500
600
Codon
Variant:
SNV
MNV
INDEL
Hotspot:
Exact
Near
20
15
10
5
0
Driver
Passenger
CNS
Colon/Rectum
CUP
Kidney
Lung
Other
Ovary
Skin
Urinary tract

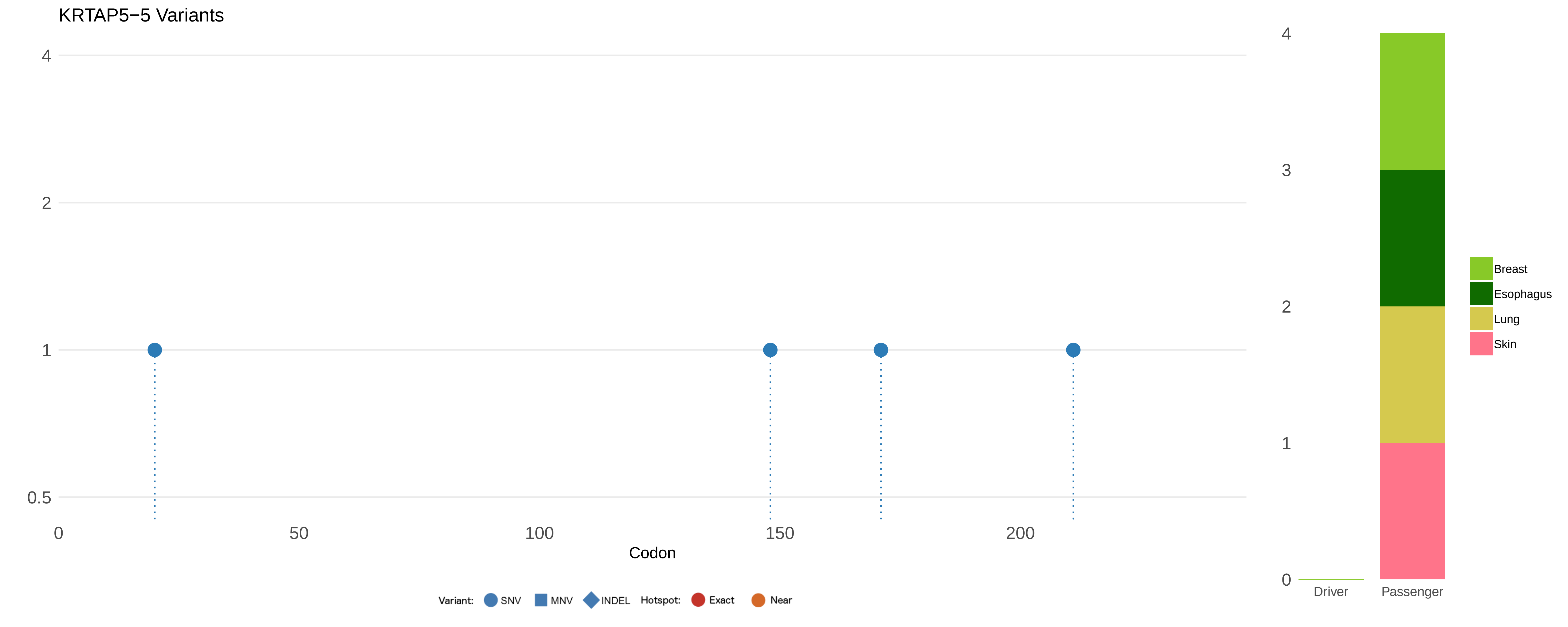
KRTAP5−5 Variants
4
2
1
0.5
0
50
100
150
200
Codon
Variant:
SNV
MNV
INDEL
Hotspot:
Exact
Near
4
3
2
1
0
Driver
Passenger
Breast
Esophagus
Lung
Skin

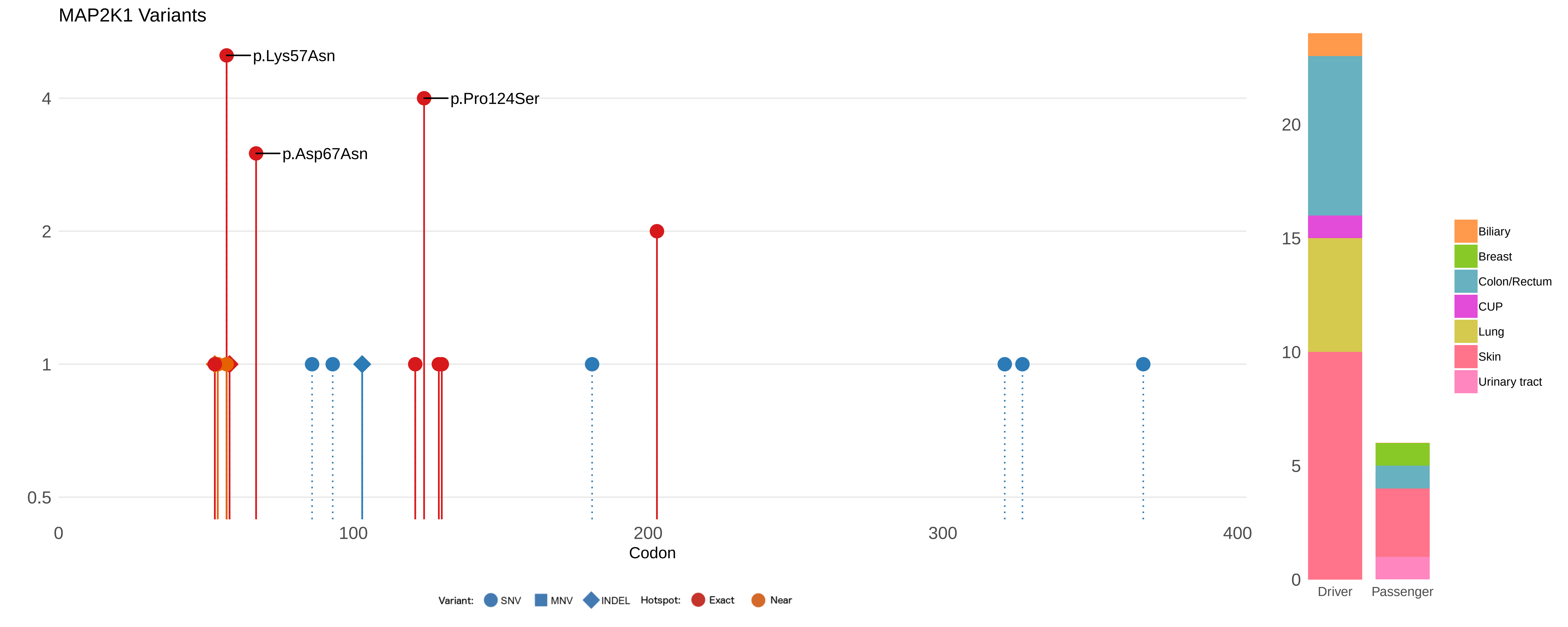
MAP2K1 Variants
p.Lys57Asn
p.Pro124Ser
p.Asp67Asn
4
2
1
0.5
0
100
200
300
400
Codon
20
15
10
5
0
Driver
Passenger
Biliary
Breast
Colon/Rectum
CUP
Lung
Skin
Urinary tract
Variant:
SNV
MNV
INDEL
Hotspot:
Exact
Near

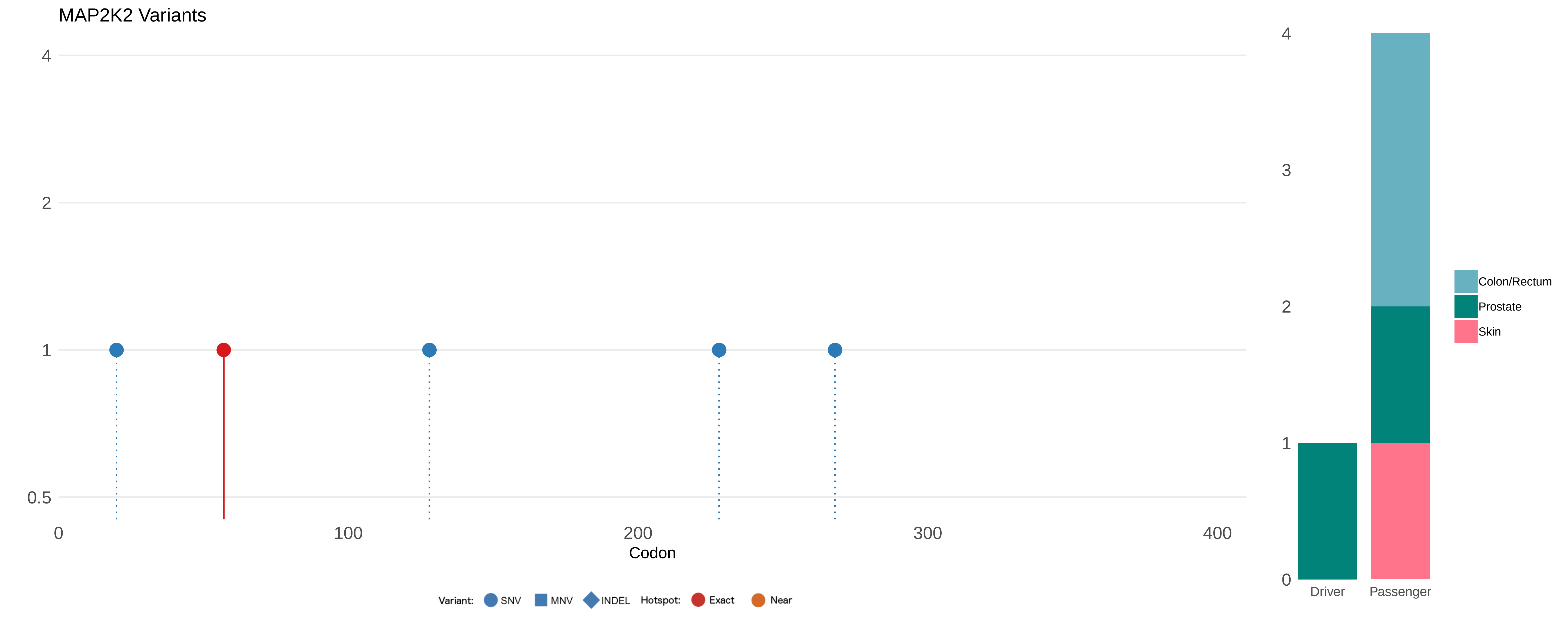

MAP2K2 Variants
4
2
1
0.5
0
100
200
300
400
Codon
Variant:
SNV
MNV
INDEL
Hotspot:
Exact
Near
4
3
2
1
0
Driver
Passenger
Colon/Rectum
Prostate
Skin

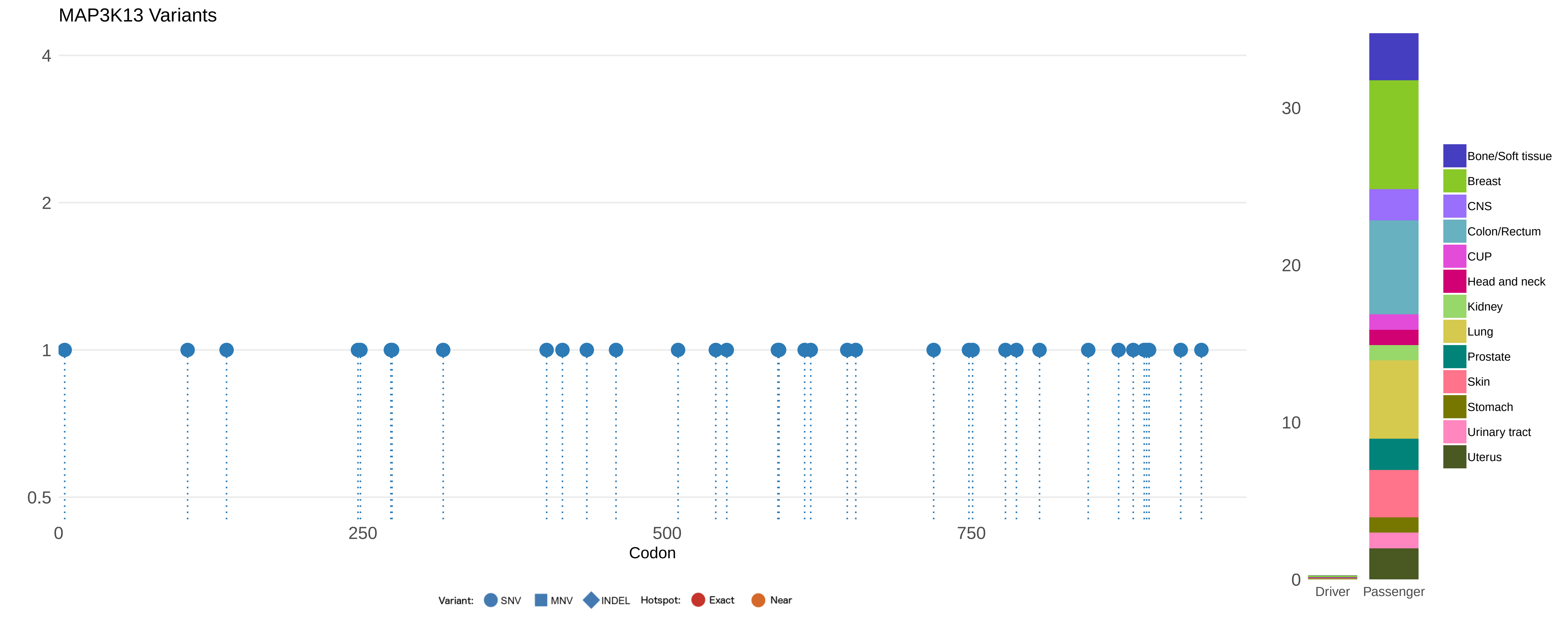
MAP3K13 Variants
4
2
1
0.5
0
250
500
750
Codon
Variant:
SNV
MNV
INDEL
Hotspot:
Exact
Near
30
20
10
0
Driver
Passenger
Bone/Soft tissue
Breast
CNS
Colon/Rectum
CUP
Head and neck
Kidney
Lung
Prostate
Skin
Stomach
Urinary tract
Uterus

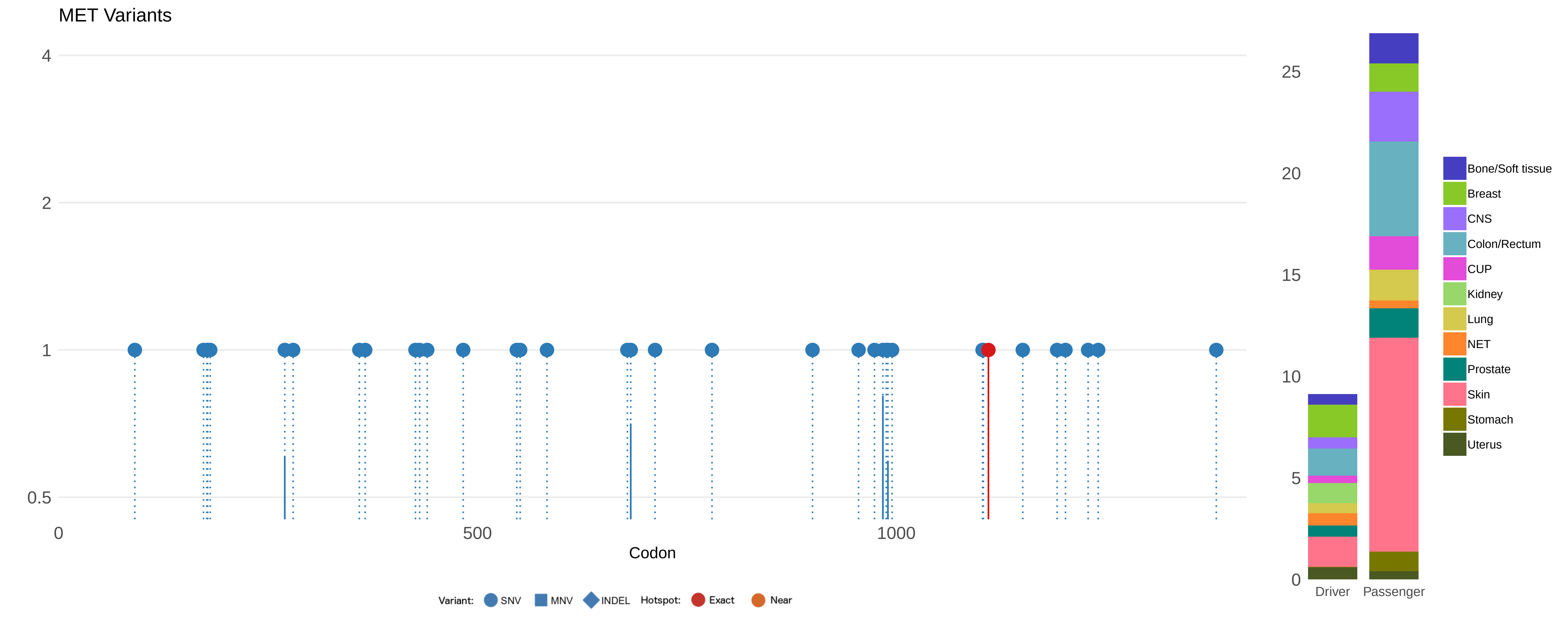

MET Variants
4
2
1
0.5
0
500
1000
Codon
Variant:
SNV
MNV
INDEL
Hotspot:
Exact
Near
25
20
15
10
5
0
Driver
Passenger
Bone/Soft tissue
Breast
CNS
Colon/Rectum
CUP
Kidney
Lung
NET
Prostate
Skin
Stomach
Uterus

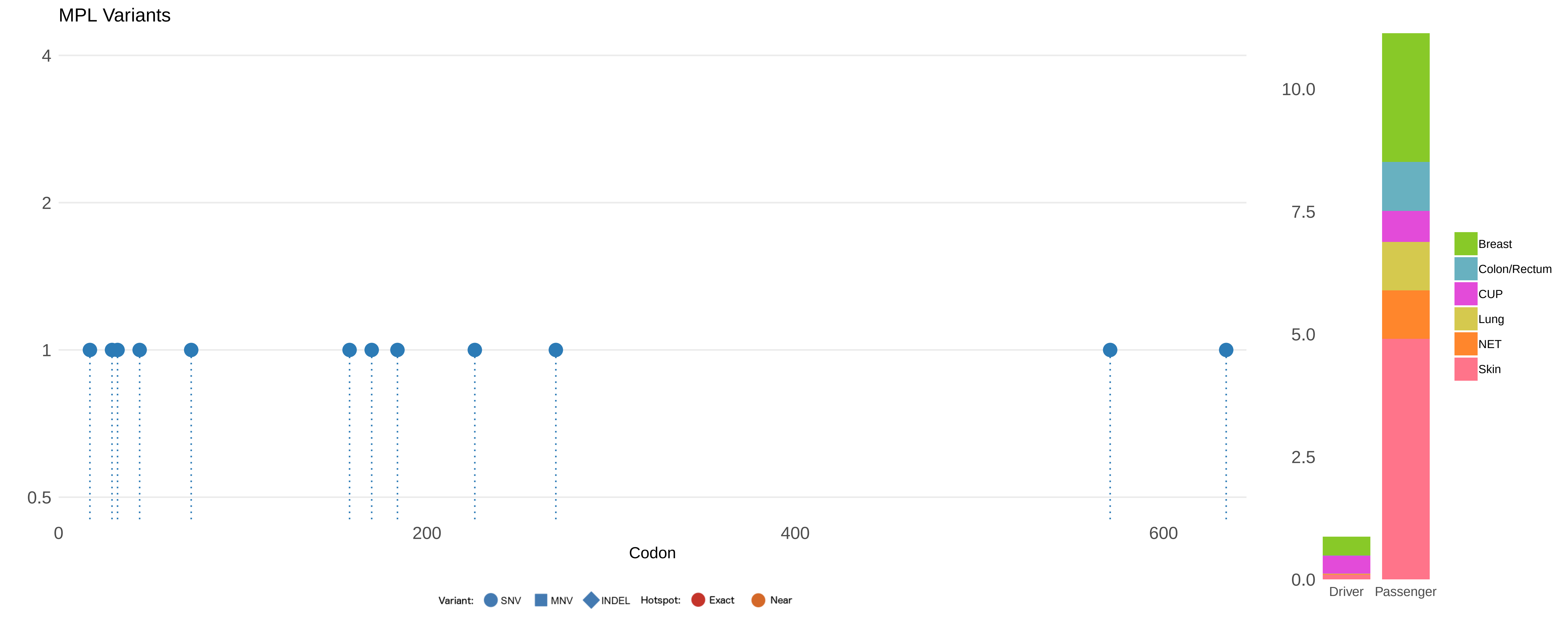

MPL Variants
4
2
1
0.5
0
200
400
600
Codon
Variant: SNV MNV INDEL Hotspot: Exact Near
10.0
7.5
5.0
2.5
0.0
Driver
Passenger
Breast
Colon/Rectum
CUP
Lung
NET
Skin

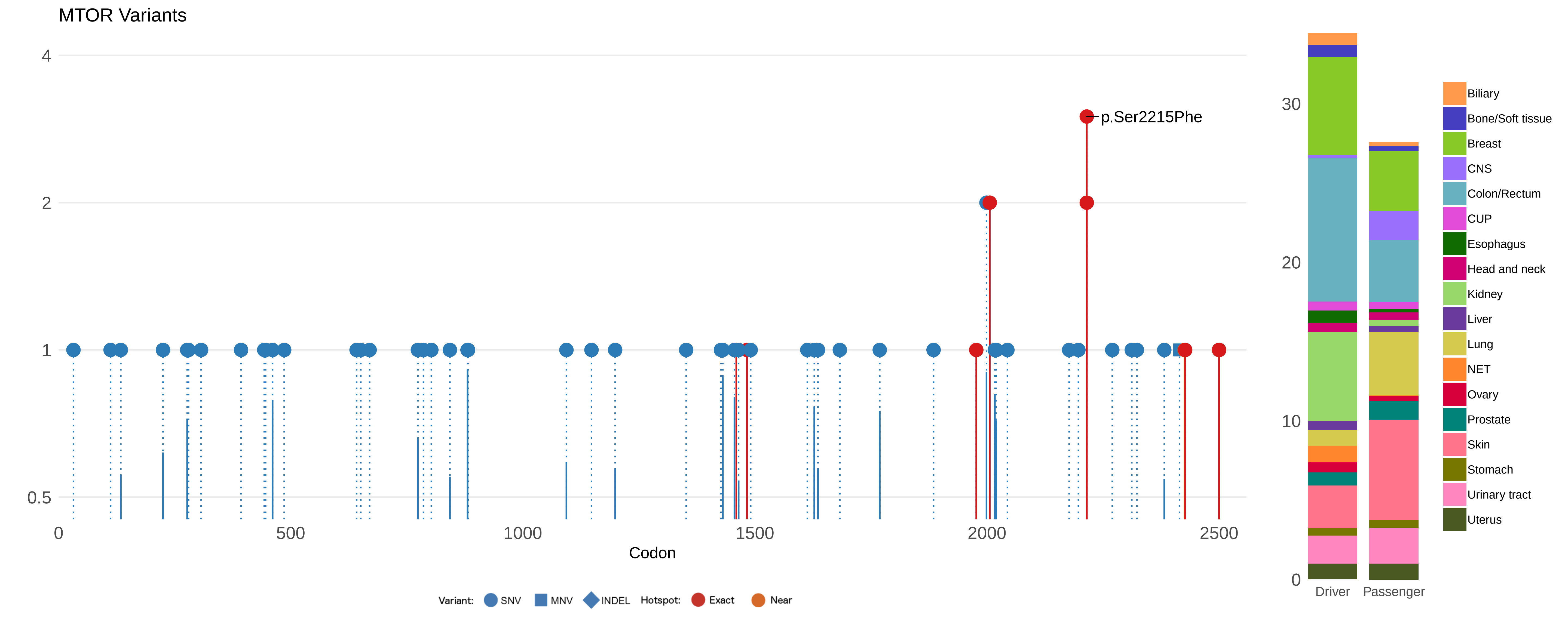

MTOR Variants
4
2
1
0.5
0
500
1000
1500
2000
2500
Codon
p.Ser2215Phe
Variant:
SNV
MNV
INDEL
Hotspot:
Exact
Near
30
20
10
0
Driver
Passenger
Biliary
Bone/Soft tissue
Breast
CNS
Colon/Rectum
CUP
Esophagus
Head and neck
Kidney
Liver
Lung
NET
Ovary
Prostate
Skin
Stomach
Urinary tract
Uterus

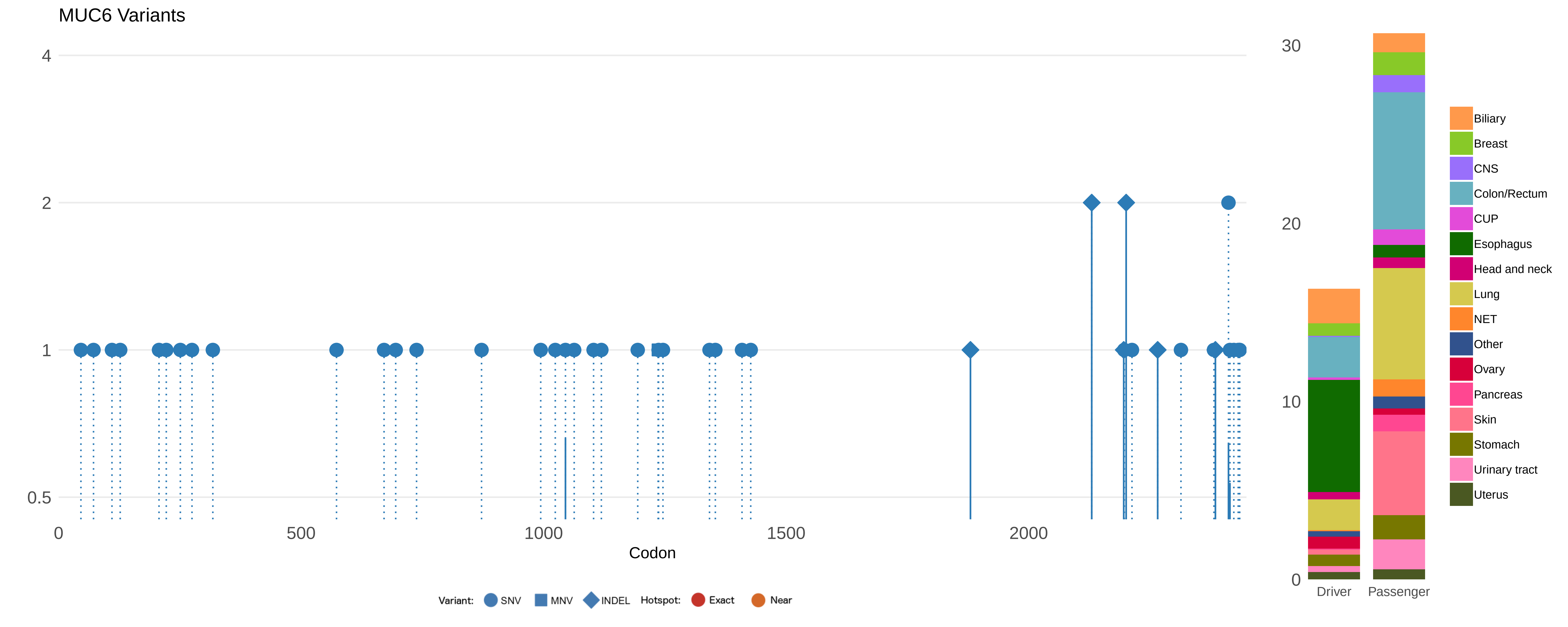
MUC6 Variants
4
2
1
0.5
0
500
1000
1500
2000
Codon
Variant: SNV MNV INDEL Hotspot: Exact Near
30
20
10
0
Driver
Passenger
Biliary
Breast
CNS
Colon/Rectum
CUP
Esophagus
Head and neck
Lung
NET
Other
Ovary
Pancreas
Skin
Stomach
Urinary tract
Uterus

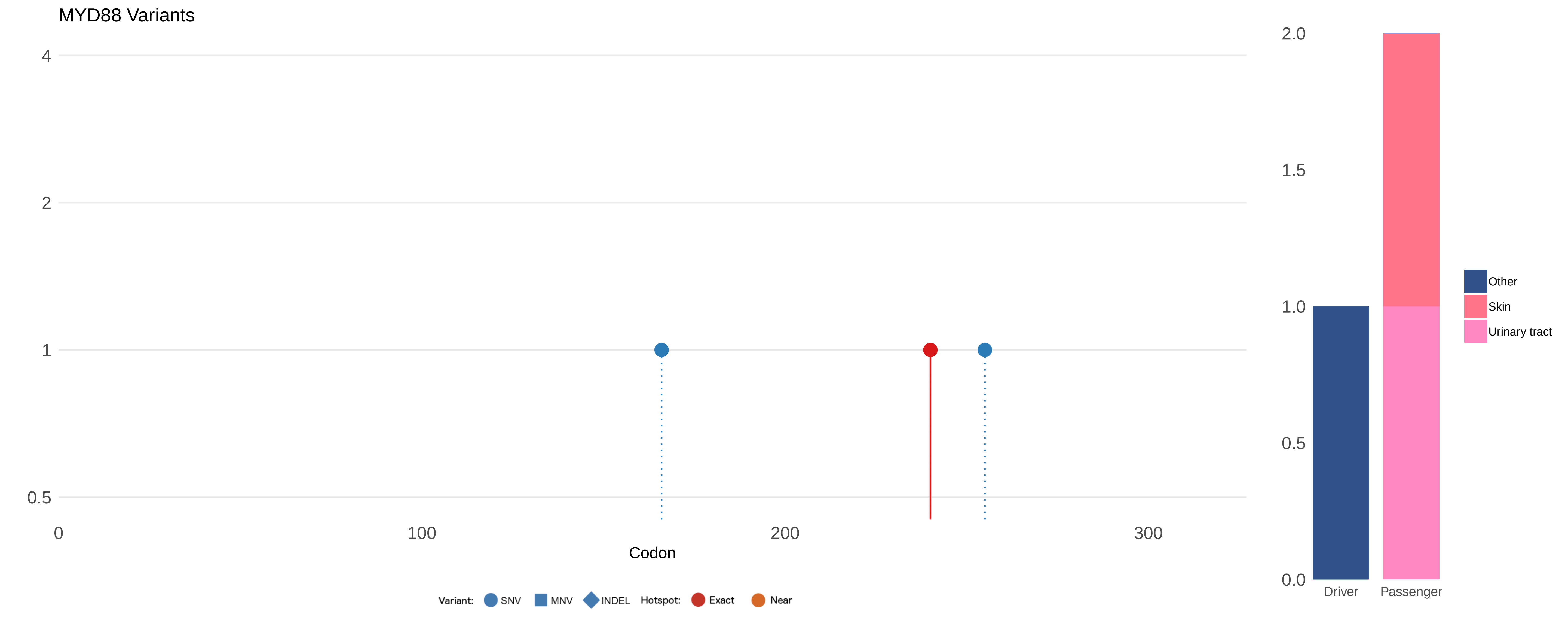
MYD88 Variants
4
2
1
0.5
0
100
200
300
Codon
Variant:
SNV
MNV
INDEL
Hotspot:
Exact
Near
2.0
1.5
1.0
0.5
0.0
Driver
Passenger
Other
Skin
Urinary tract

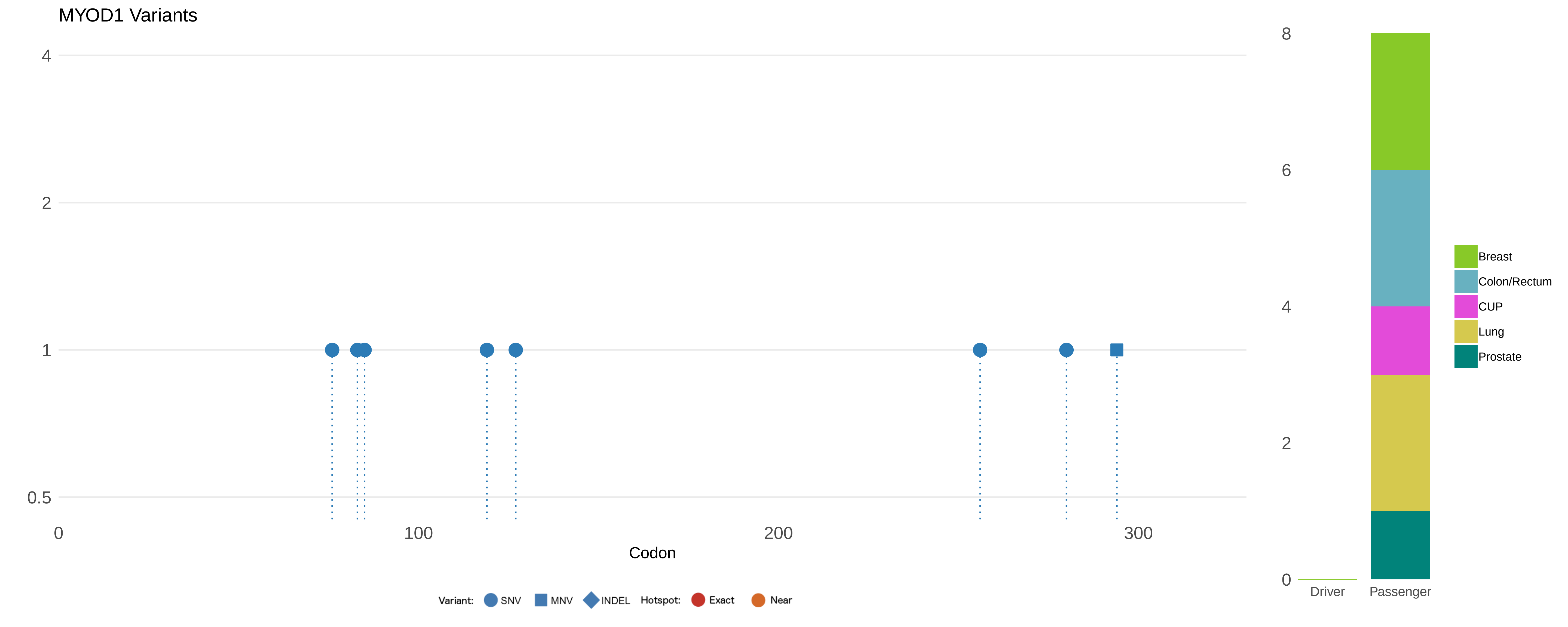
MYOD1 Variants
4
2
1
0.5
0
100
200
300
Codon
Variant:
SNV
MNV
INDEL
Hotspot:
Exact
Near
8
6
4
2
0
Driver
Passenger
Breast
Colon/Rectum
CUP
Lung
Prostate

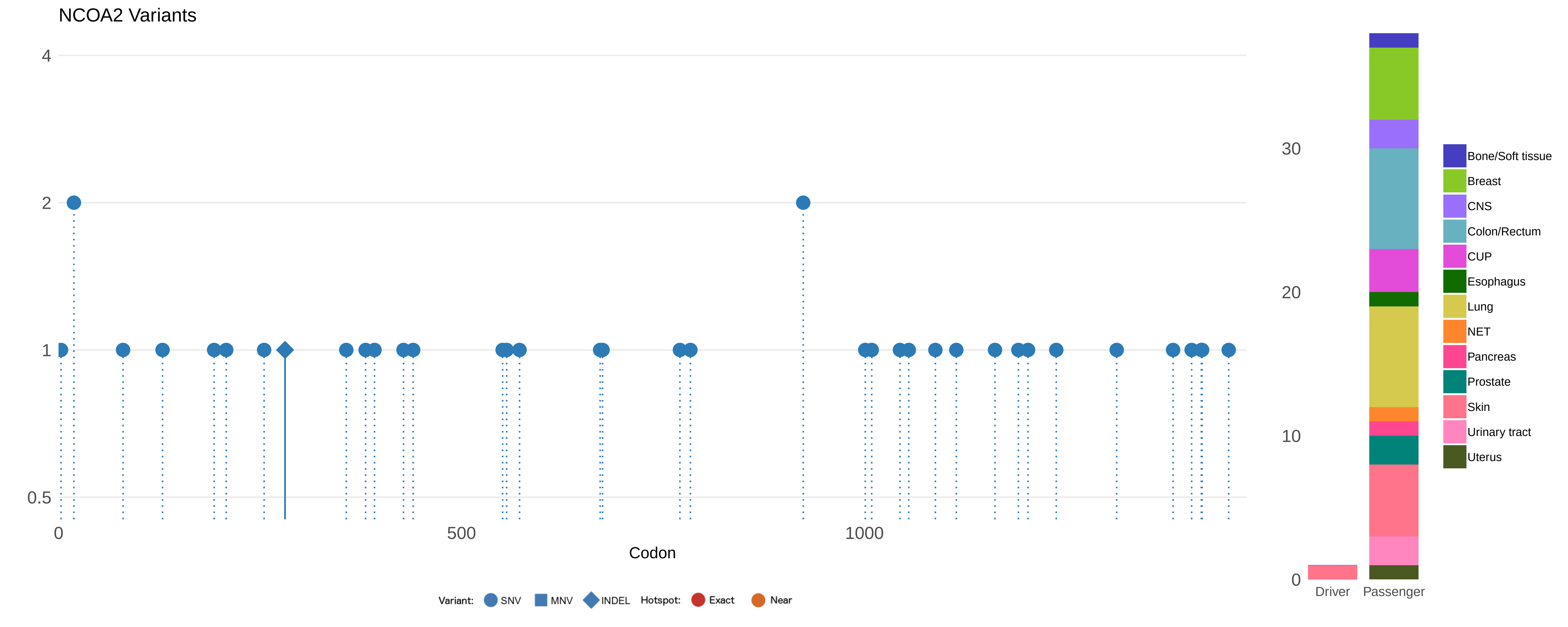
NCOA2 Variants
4
2
1
0.5
0
500
1000
Codon
Variant:
SNV
MNV
INDEL
Hotspot:
Exact
Near
30
20
10
0
Driver
Passenger
Bone/Soft tissue
Breast
CNS
Colon/Rectum
CUP
Esophagus
Lung
NET
Pancreas
Prostate
Skin
Urinary tract
Uterus

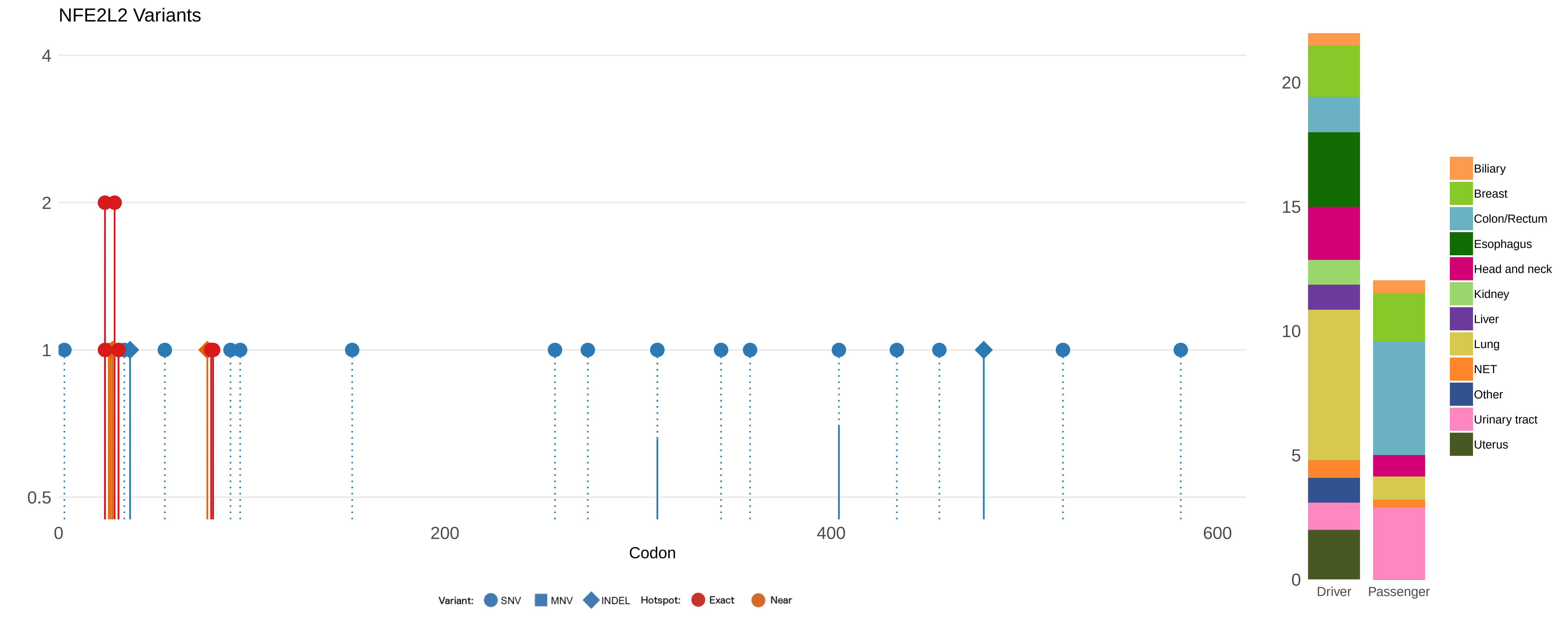
NFE2L2 Variants
4
2
1
0.5
0
200
400
600
Codon
Variant:
SNV
MNV
INDEL
Hotspot:
Exact
Near
20
15
10
5
0
Driver
Passenger
Biliary
Breast
Colon/Rectum
Esophagus
Head and neck
Kidney
Liver
Lung
NET
Other
Urinary tract
Uterus

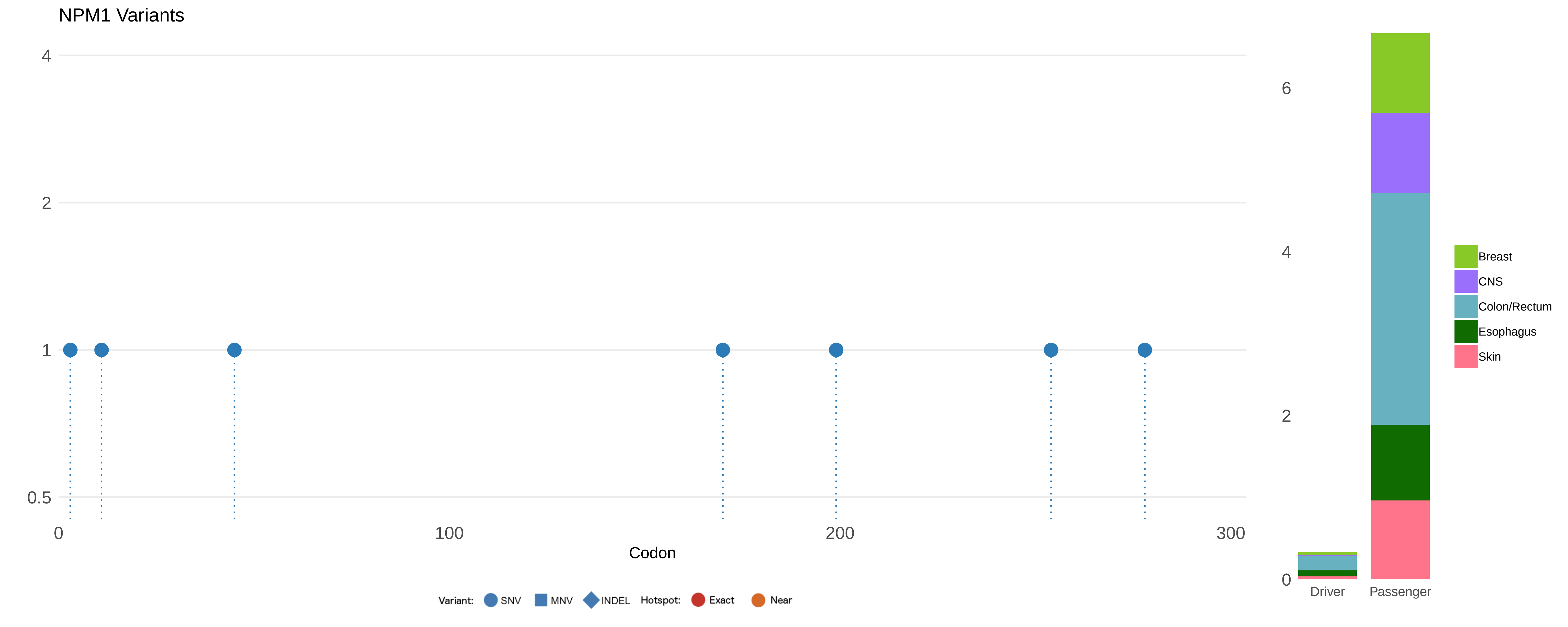

NPM1 Variants
4
2
1
0.5
0
100
200
300
Codon
Variant:
SNV
MNV
INDEL
Hotspot:
Exact
Near
6
4
2
0
Driver
Passenger
Breast
CNS
Colon/Rectum
Esophagus
Skin

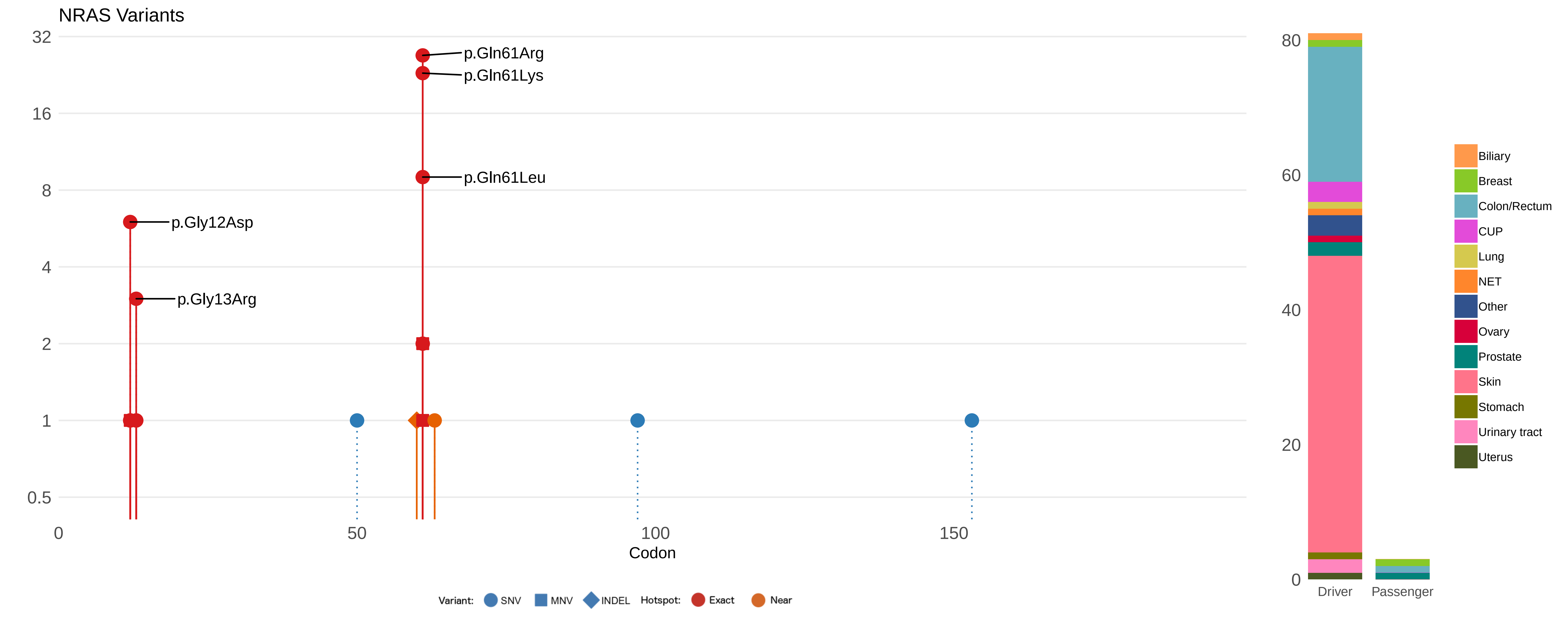

NRAS Variants
32
16
8
4
2
1
0.5
0
50
100
150
Codon
p.Gln61Arg
p.Gln61Lys
p.Gln61Leu
p.Gly12Asp
p.Gly13Arg
Variant: SNV MNV INDEL Hotspot: Exact Near
80
60
40
20
0
Driver
Passenger
Biliary
Breast
Colon/Rectum
CUP
Lung
NET
Other
Ovary
Prostate
Skin
Stomach
Urinary tract
Uterus

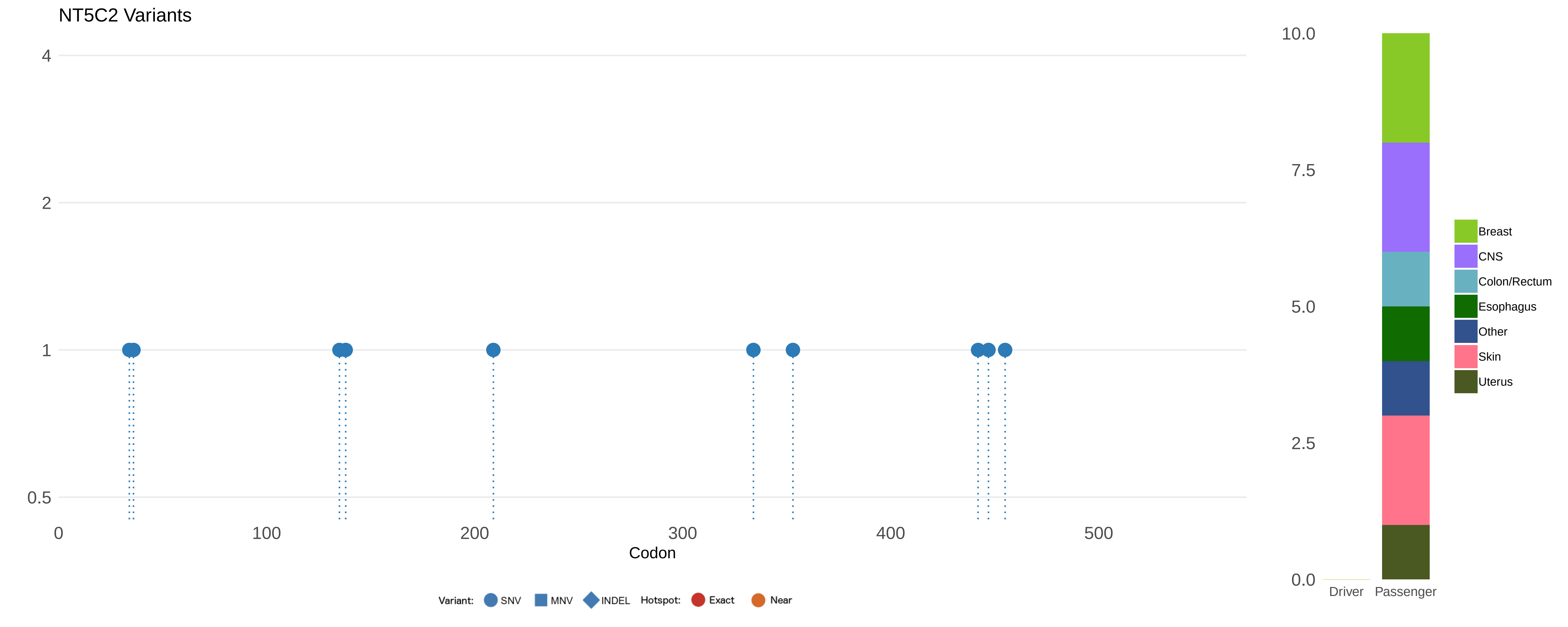

NT5C2 Variants
4
2
1
0.5
0
100
200
300
400
500
Codon
Variant:
SNV
MNV
INDEL
Hotspot:
Exact
Near
10.0
7.5
5.0
2.5
0.0
Driver
Passenger
Breast
CNS
Colon/Rectum
Esophagus
Other
Skin
Uterus

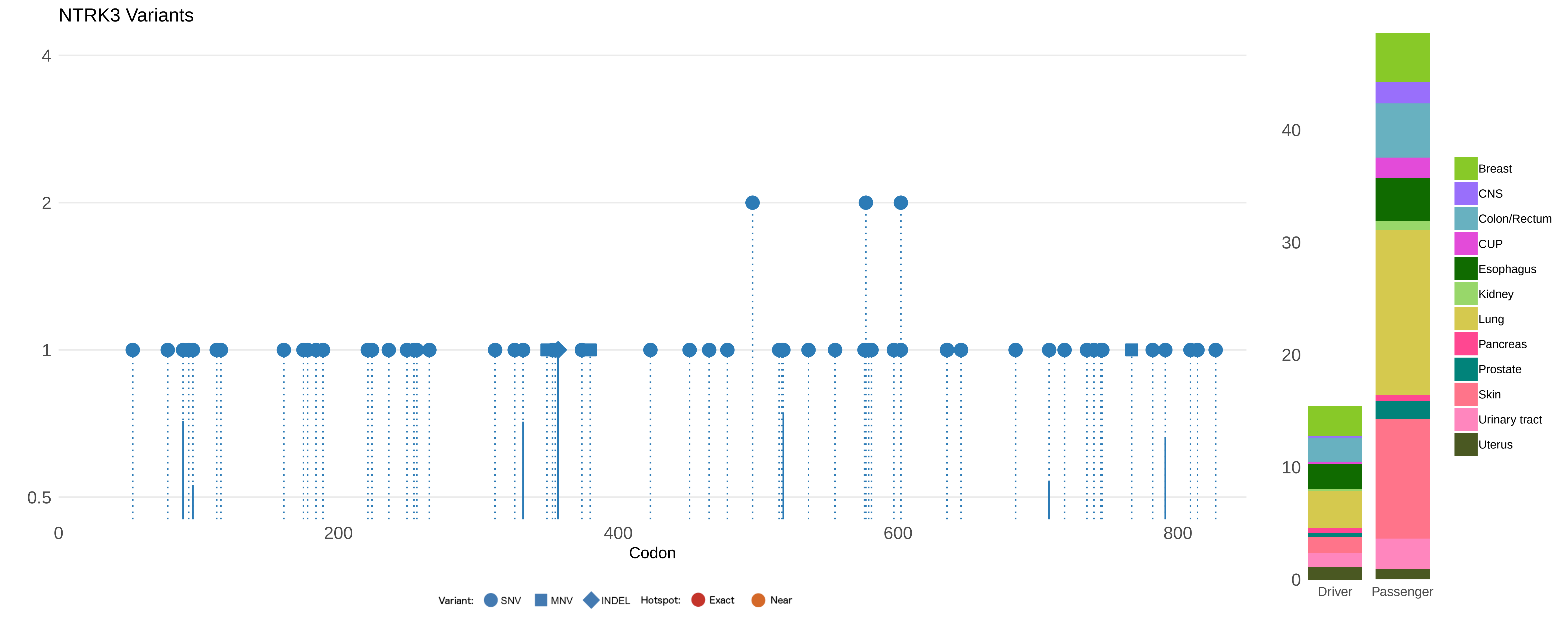
NTRK3 Variants
4
2
1
0.5
0
200
400
600
800
Codon
Variant: SNV MNV INDEL Hotspot: Exact Near
40
30
20
10
0
Driver
Passenger
Breast
CNS
Colon/Rectum
CUP
Esophagus
Kidney
Lung
Pancreas
Prostate
Skin
Urinary tract
Uterus

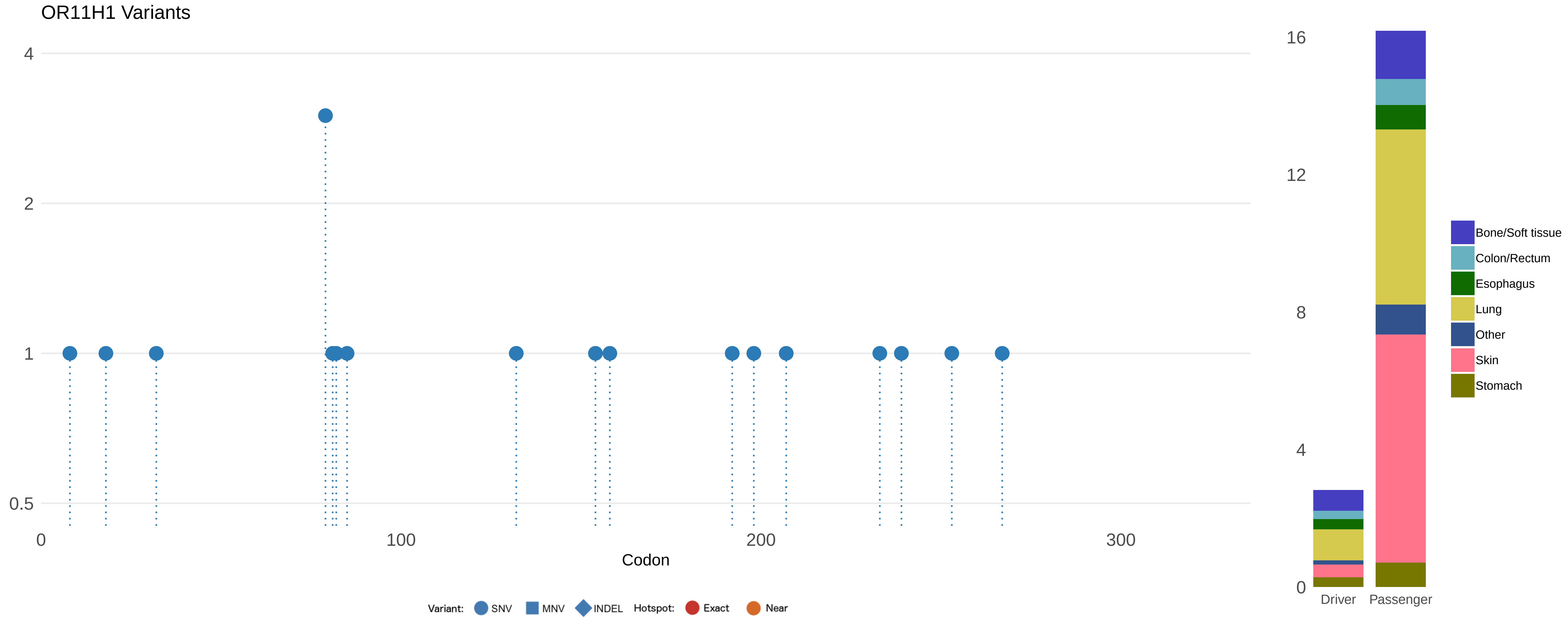

OR11H1 Variants
4
2
1
0.5
0
100
200
300
Codon
Variant:
SNV
MNV
INDEL
Hotspot:
Exact
Near
16
12
8
4
0
Driver
Passenger
Bone/Soft tissue
Colon/Rectum
Esophagus
Lung
Other
Skin
Stomach

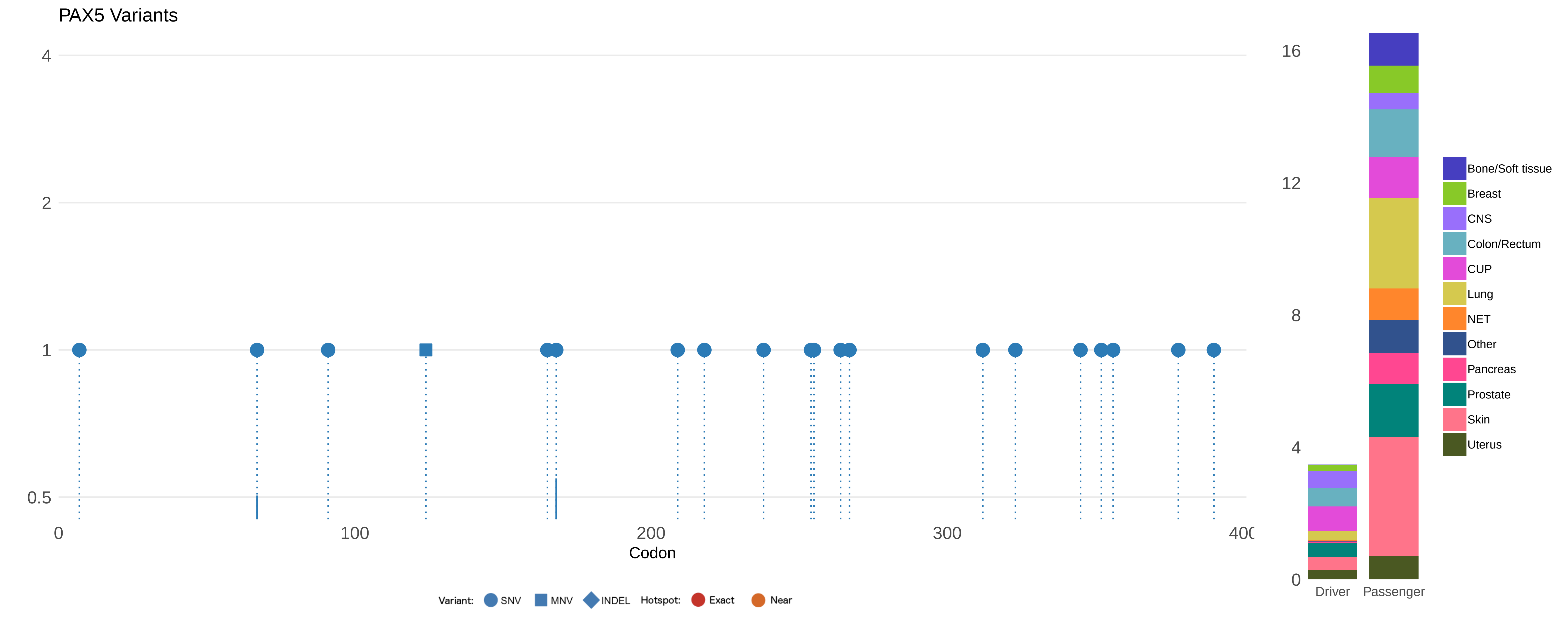

PAX5 Variants
4
2
1
0.5
0
100
200
300
400
Codon
Variant: SNV MNV INDEL Hotspot: Exact Near
16
12
8
4
0
Driver
Passenger
Bone/Soft tissue
Breast
CNS
Colon/Rectum
CUP
Lung
NET
Other
Pancreas
Prostate
Skin
Uterus

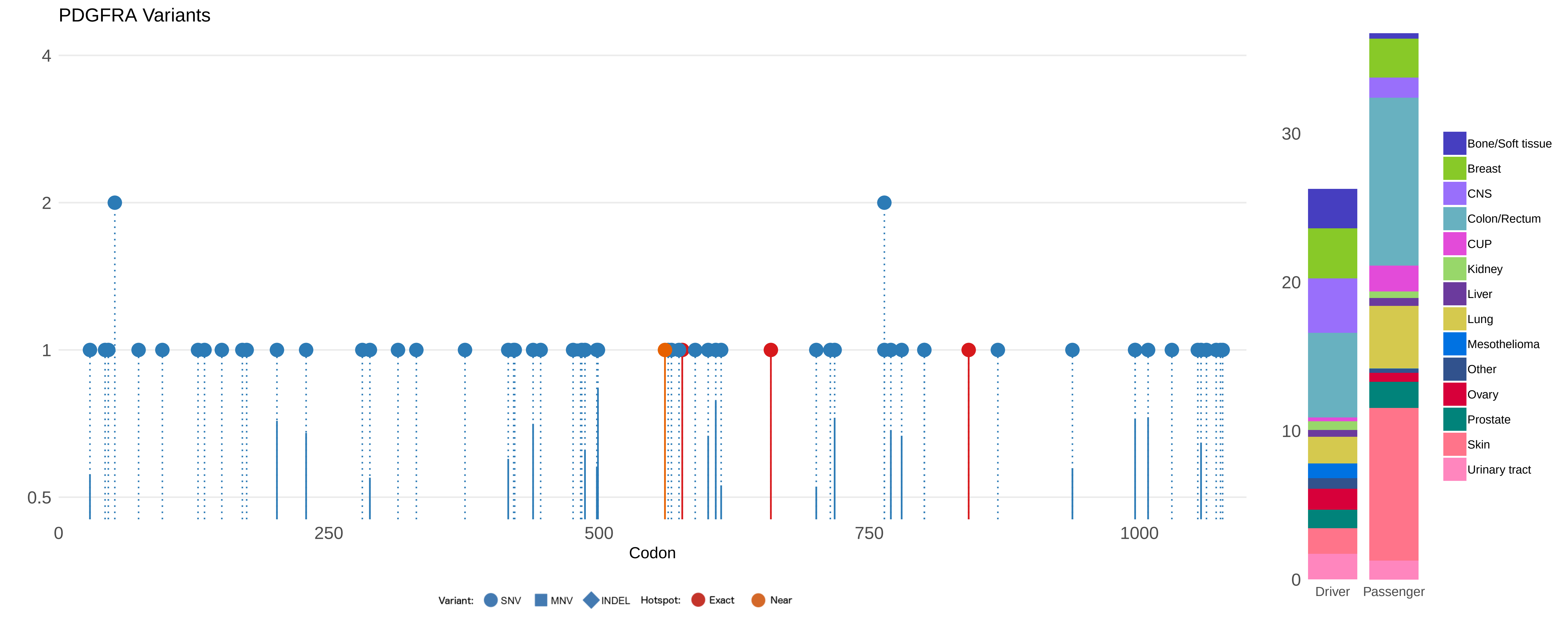

PDGFRA Variants
4
2
1
0.5
0
250
500
750
1000
Codon
Variant:
SNV
MNV
INDEL
Hotspot:
Exact
Near
30
20
10
0
Driver
Passenger
Bone/Soft tissue
Breast
CNS
Colon/Rectum
CUP
Kidney
Liver
Lung
Mesothelioma
Other
Ovary
Prostate
Skin
Urinary tract

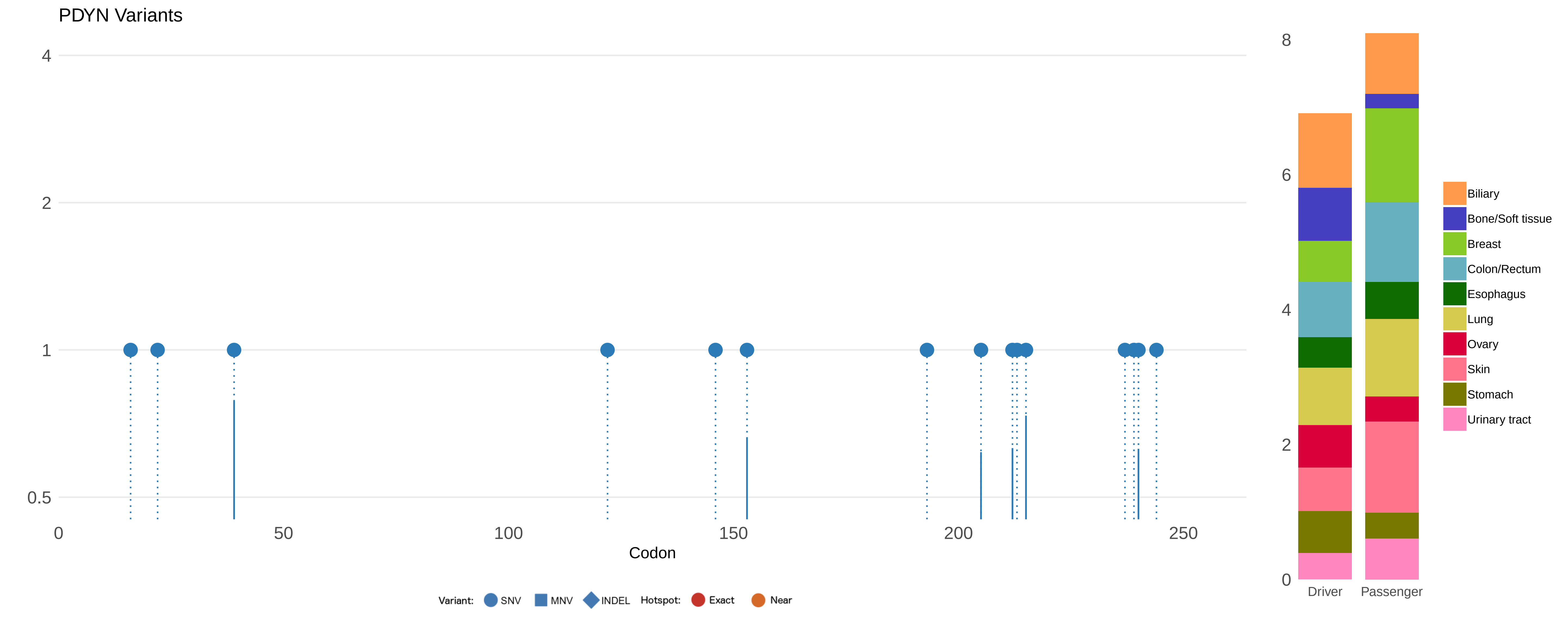
PDYN Variants
4
2
1
0.5
0
50
100
150
200
250
Codon
Variant:
SNV
MNV
INDEL
Hotspot:
Exact
Near
8
6
4
2
0
Driver
Passenger
Biliary
Bone/Soft tissue
Breast
Colon/Rectum
Esophagus
Lung
Ovary
Skin
Stomach
Urinary tract

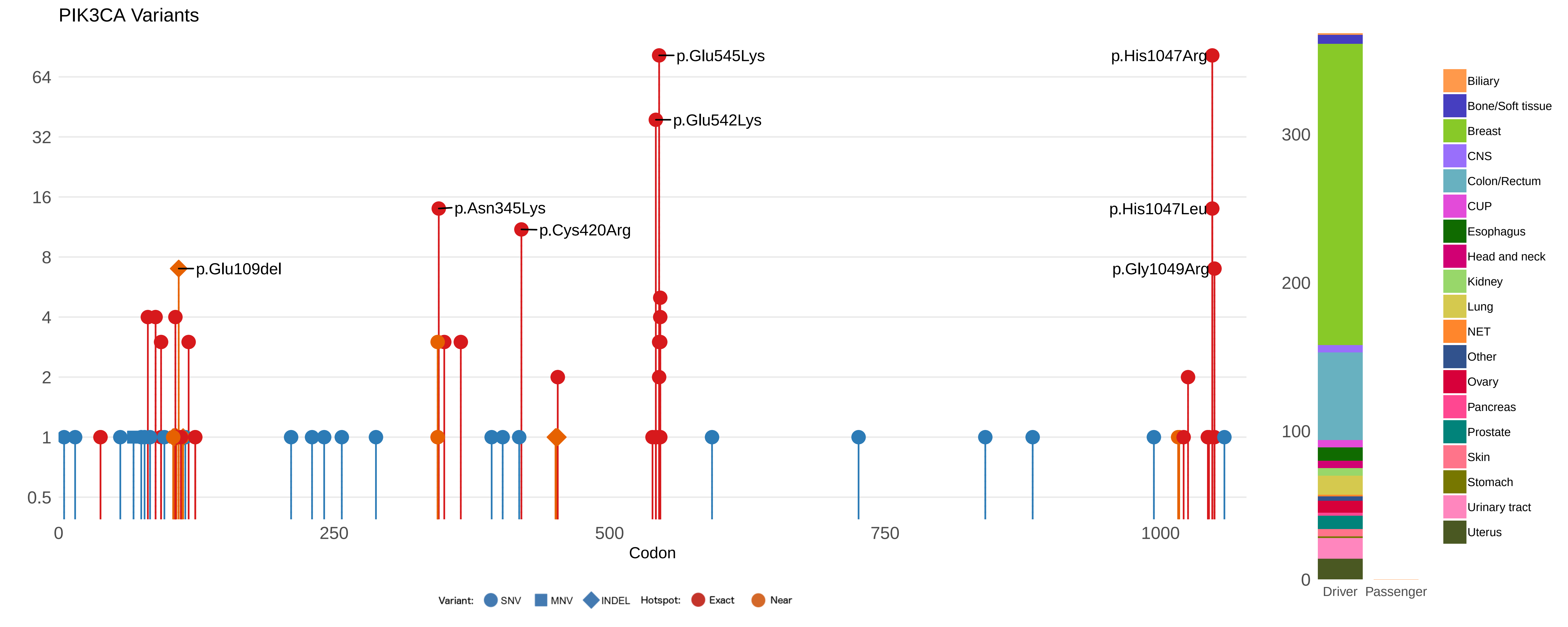

PIK3CA Variants
p.Glu545Lys
p.Glu542Lys
p.Asn345Lys
p.Cys420Arg
p.Glu109del
p.His1047Arg
p.His1047Leu
p.Gly1049Arg
64
32
16
8
4
2
1
0.5
0
250
500
750
1000
Codon
Variant: SNV MNV INDEL Hotspot: Exact Near
300
200
100
0
Driver Passenger
Biliary
Bone/Soft tissue
Breast
CNS
Colon/Rectum
CUP
Esophagus
Head and neck
Kidney
Lung
NET
Other
Ovary
Pancreas
Prostate
Skin
Stomach
Urinary tract
Uterus

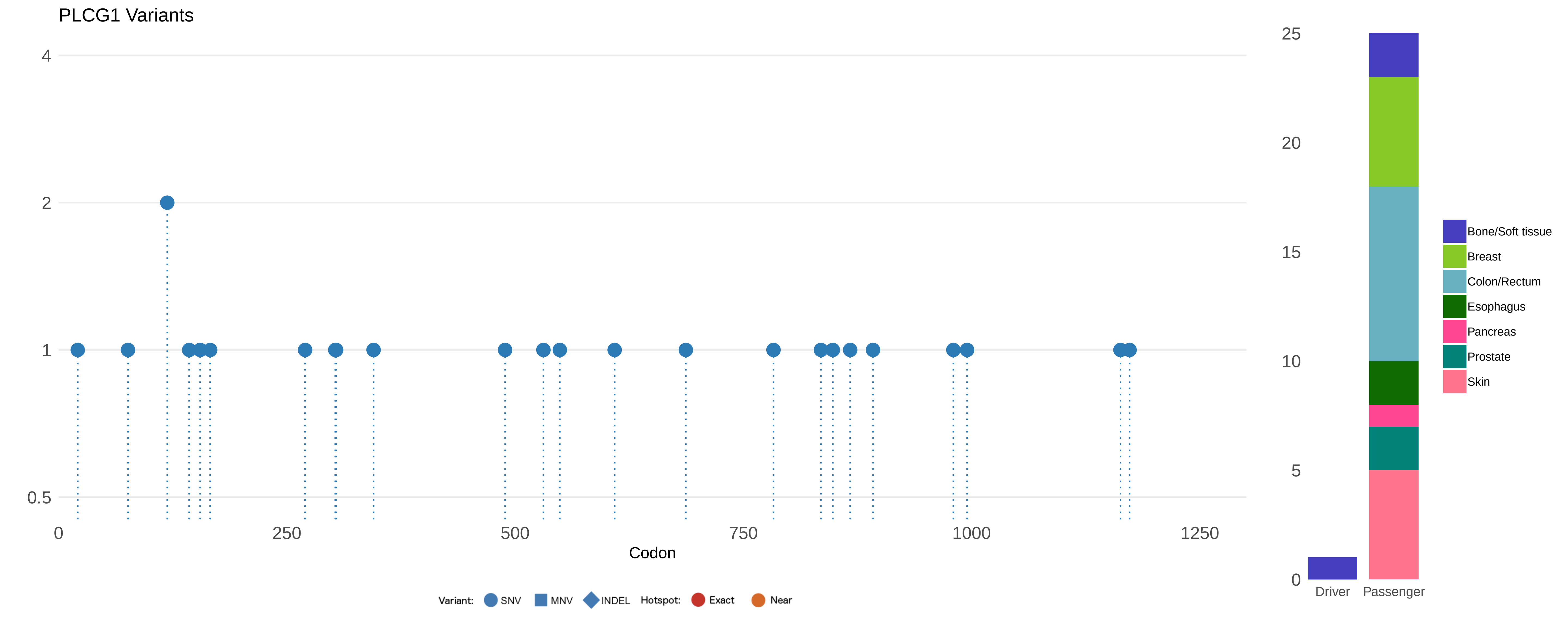
PLCG1 Variants
4
2
1
0.5
0
250
500
750
1000
1250
Codon
Variant: SNV MNV INDEL Hotspot: Exact Near
25
20
15
10
5
0
Driver
Passenger
Bone/Soft tissue
Breast
Colon/Rectum
Esophagus
Pancreas
Prostate
Skin

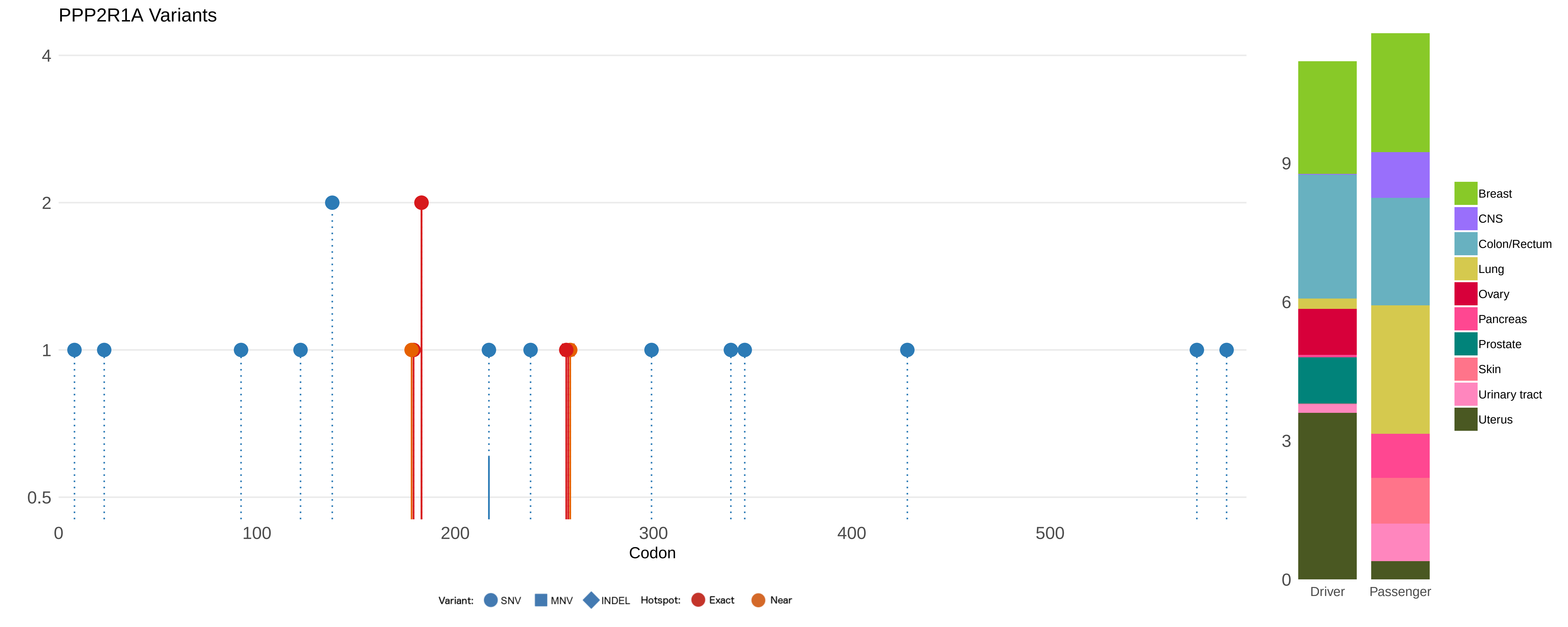

PPP2R1A Variants
4
2
1
0.5
0
100
200
300
400
500
Codon
Variant:
SNV
MNV
INDEL
Hotspot:
Exact
Near
9
6
3
0
Driver
Passenger
Breast
CNS
Colon/Rectum
Lung
Ovary
Pancreas
Prostate
Skin
Urinary tract
Uterus

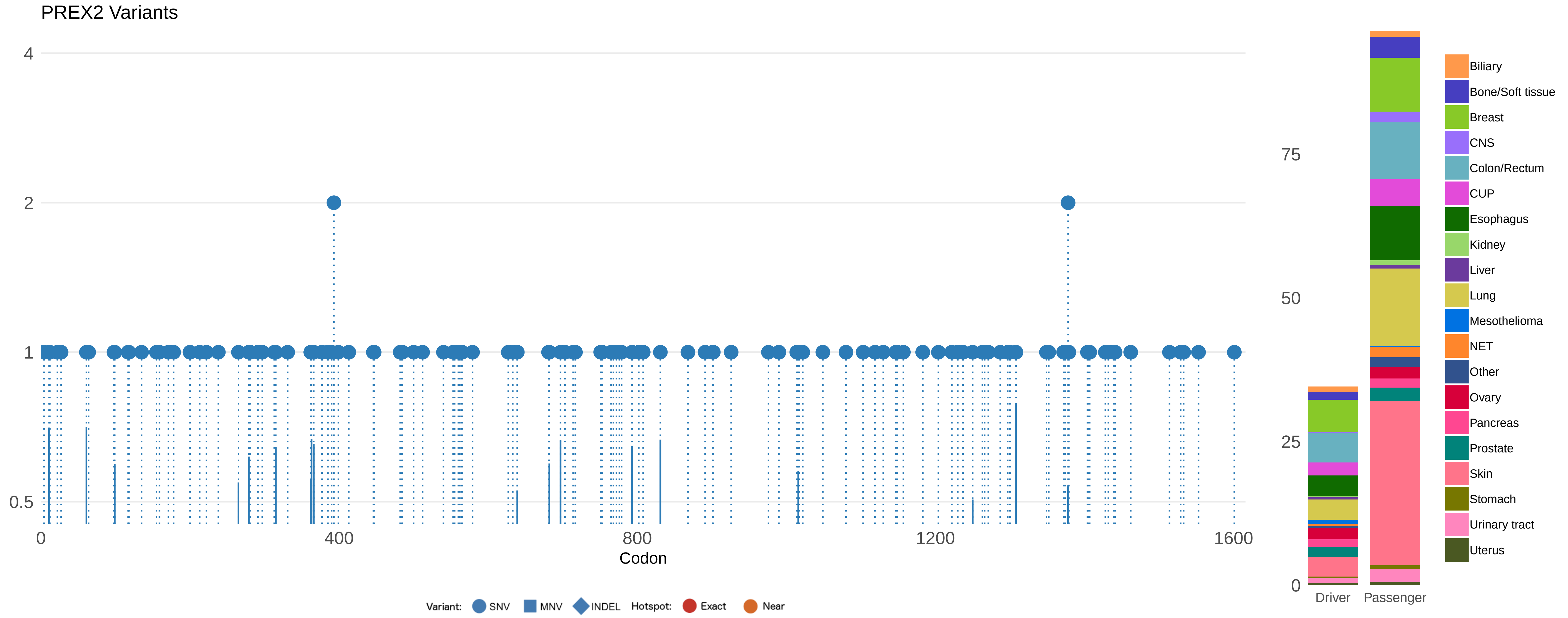

PREX2 Variants
4
2
1
0.5
0
400
800
1200
1600
Codon
Variant:
SNV
MNV
INDEL
Hotspot:
Exact
Near
75
50
25
0
Driver
Passenger
Biliary
Bone/Soft tissue
Breast
CNS
Colon/Rectum
CUP
Esophagus
Kidney
Liver
Lung
Mesothelioma
NET
Other
Ovary
Pancreas
Prostate
Skin
Stomach
Urinary tract
Uterus

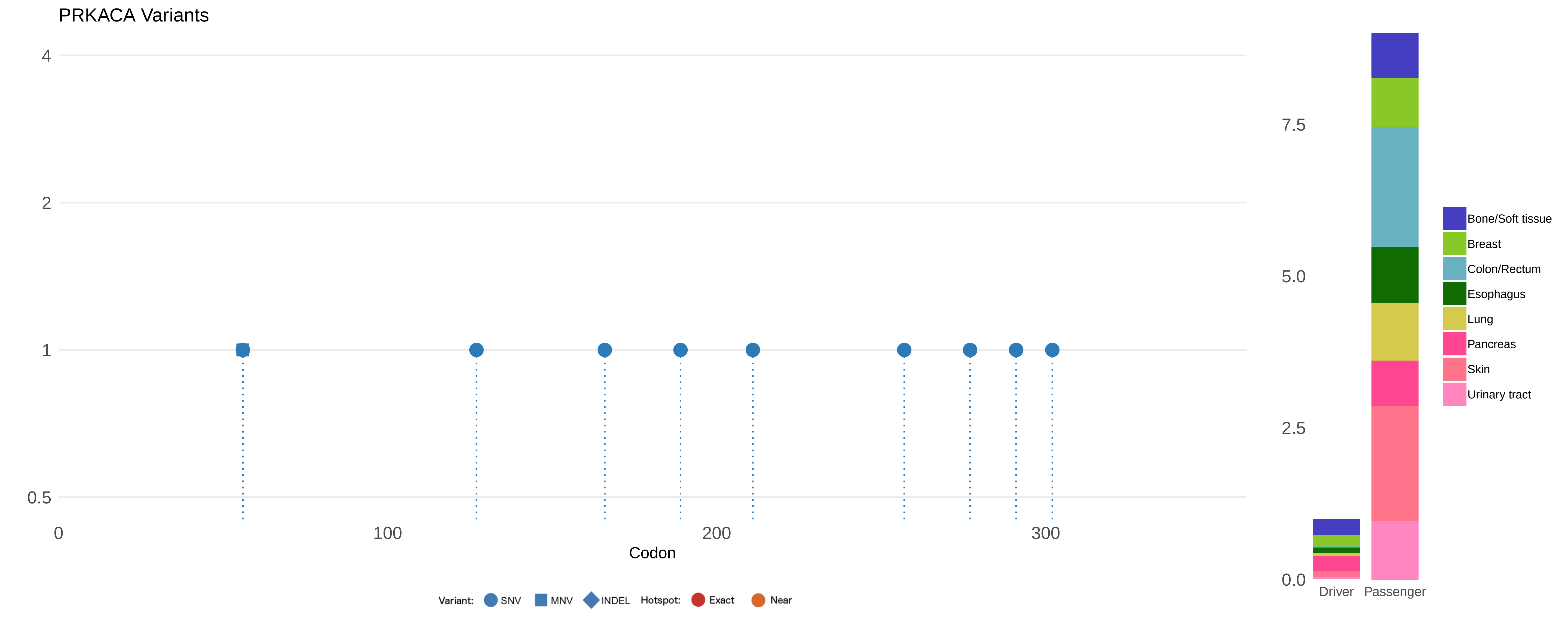

PRKACA Variants
4
2
1
0.5
0
100
200
300
Codon
Variant:
SNV
MNV
INDEL
Hotspot:
Exact
Near
7.5
5.0
2.5
0.0
Driver
Passenger
Bone/Soft tissue
Breast
Colon/Rectum
Esophagus
Lung
Pancreas
Skin
Urinary tract

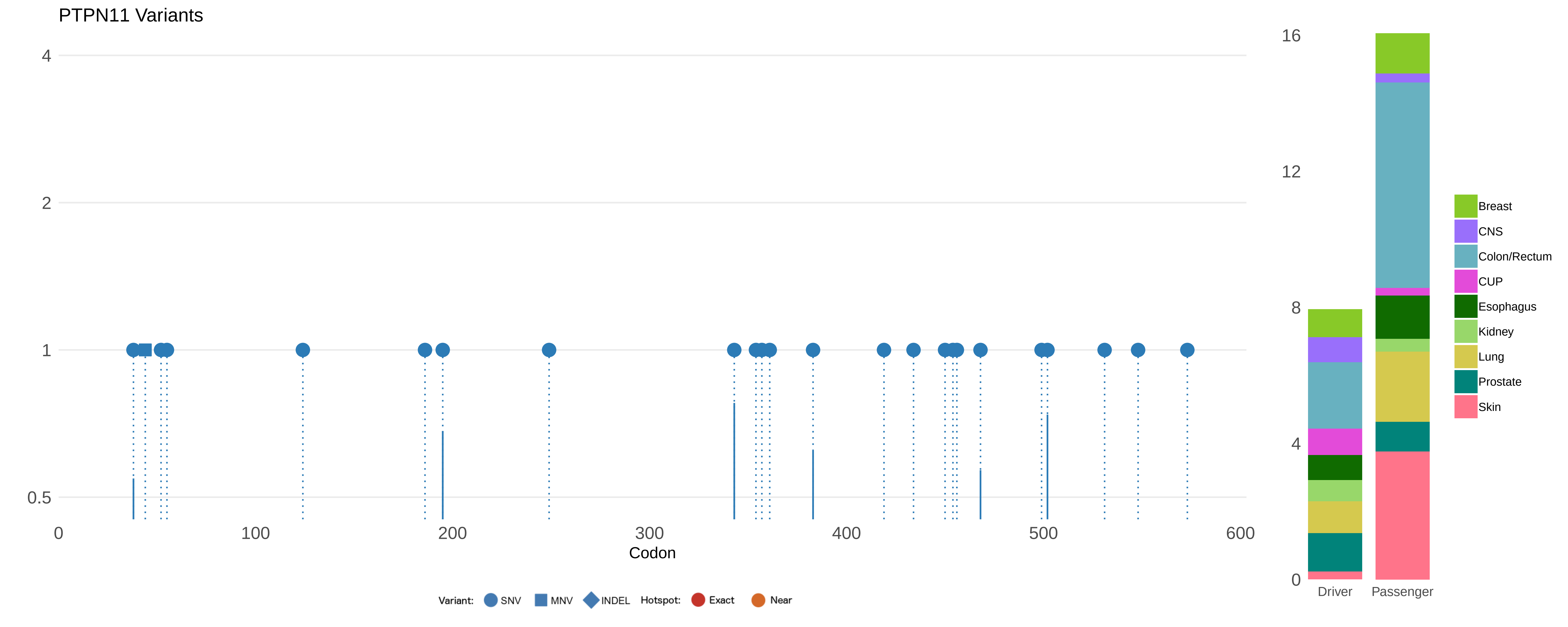
PTPN11 Variants
4
2
1
0.5
0
100
200
300
400
500
600
Codon
16
12
8
4
0
Driver
Passenger
Breast
CNS
Colon/Rectum
CUP
Esophagus
Kidney
Lung
Prostate
Skin
Variant:
SNV
MNV
INDEL
Hotspot:
Exact
Near

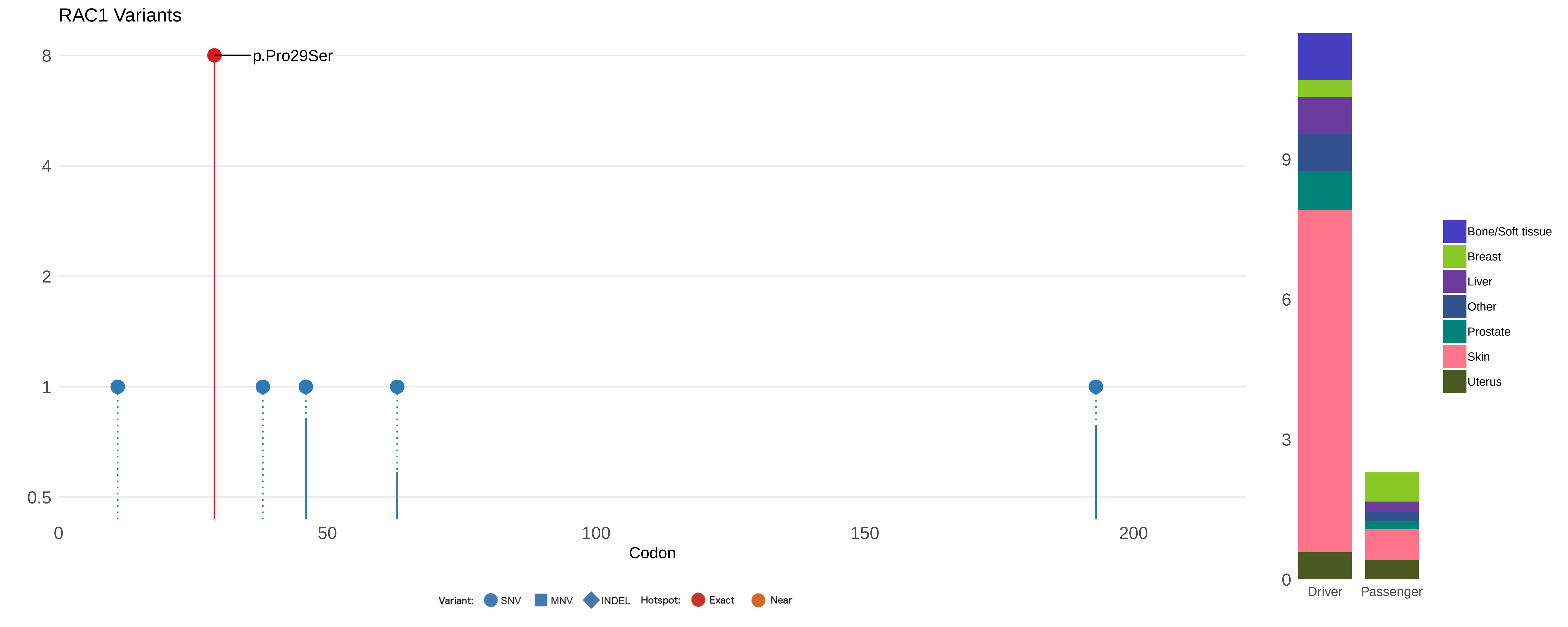

RAC1 Variants
p.Pro29Ser
8
4
2
1
0.5
0
50
100
150
200
Codon
Variant:
SNV
MNV
INDEL
Hotspot:
Exact
Near
9
6
3
0
Driver
Passenger
Bone/Soft tissue
Breast
Liver
Other
Prostate
Skin
Uterus

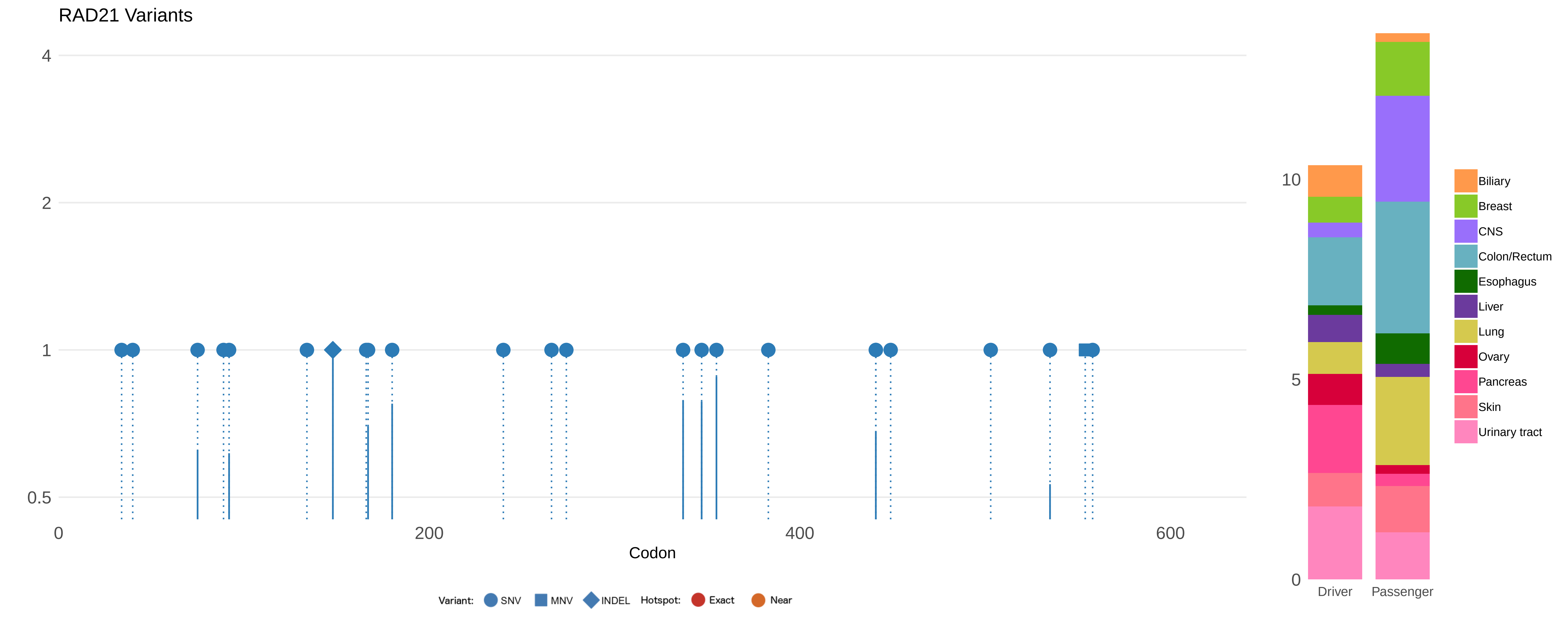

RAD21 Variants
4
2
1
0.5
0
200
400
600
Codon
Variant:
SNV
MNV
INDEL
Hotspot:
Exact
Near
10
5
0
Driver
Passenger
Biliary
Breast
CNS
Colon/Rectum
Esophagus
Liver
Lung
Ovary
Pancreas
Skin
Urinary tract

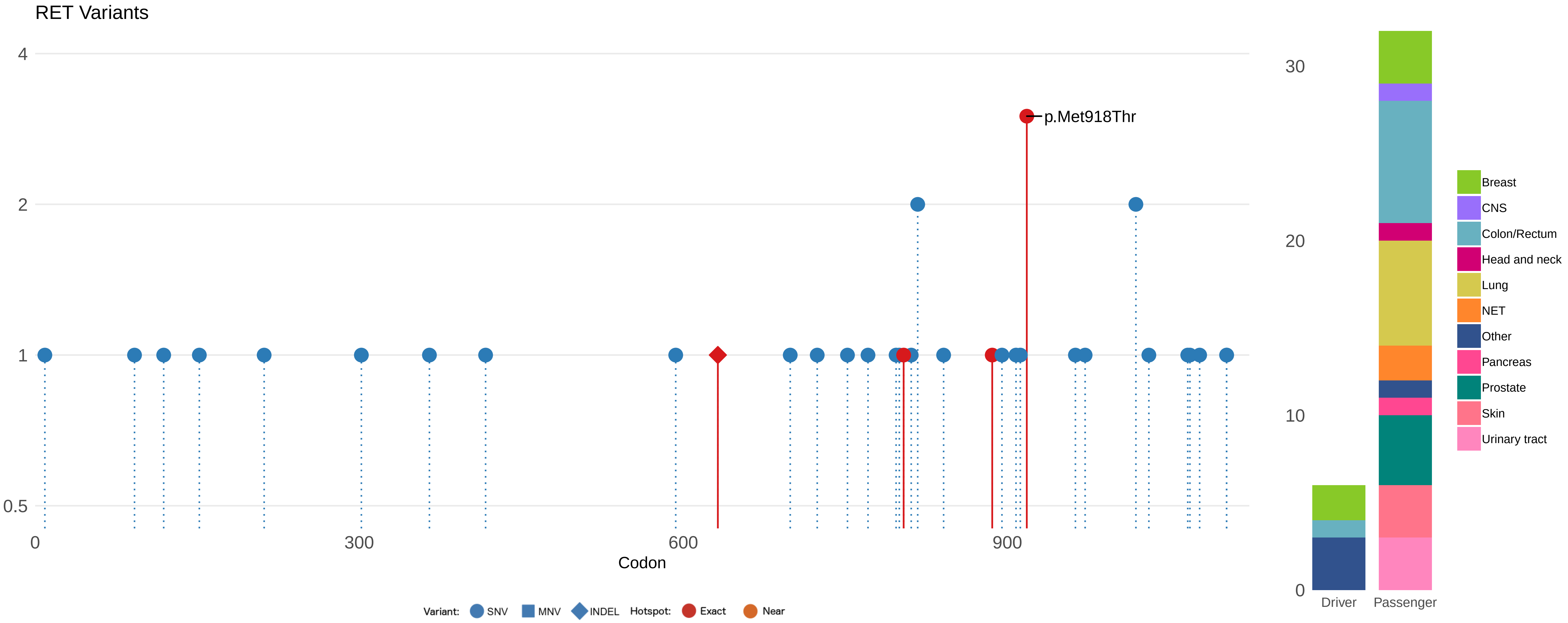

RET Variants
p.Met918Thr
4
2
1
0.5
0
300
600
900
Codon
Variant:
SNV
MNV
INDEL
Hotspot:
Exact
Near
30
20
10
0
Driver
Passenger
Breast
CNS
Colon/Rectum
Head and neck
Lung
NET
Other
Pancreas
Prostate
Skin
Urinary tract

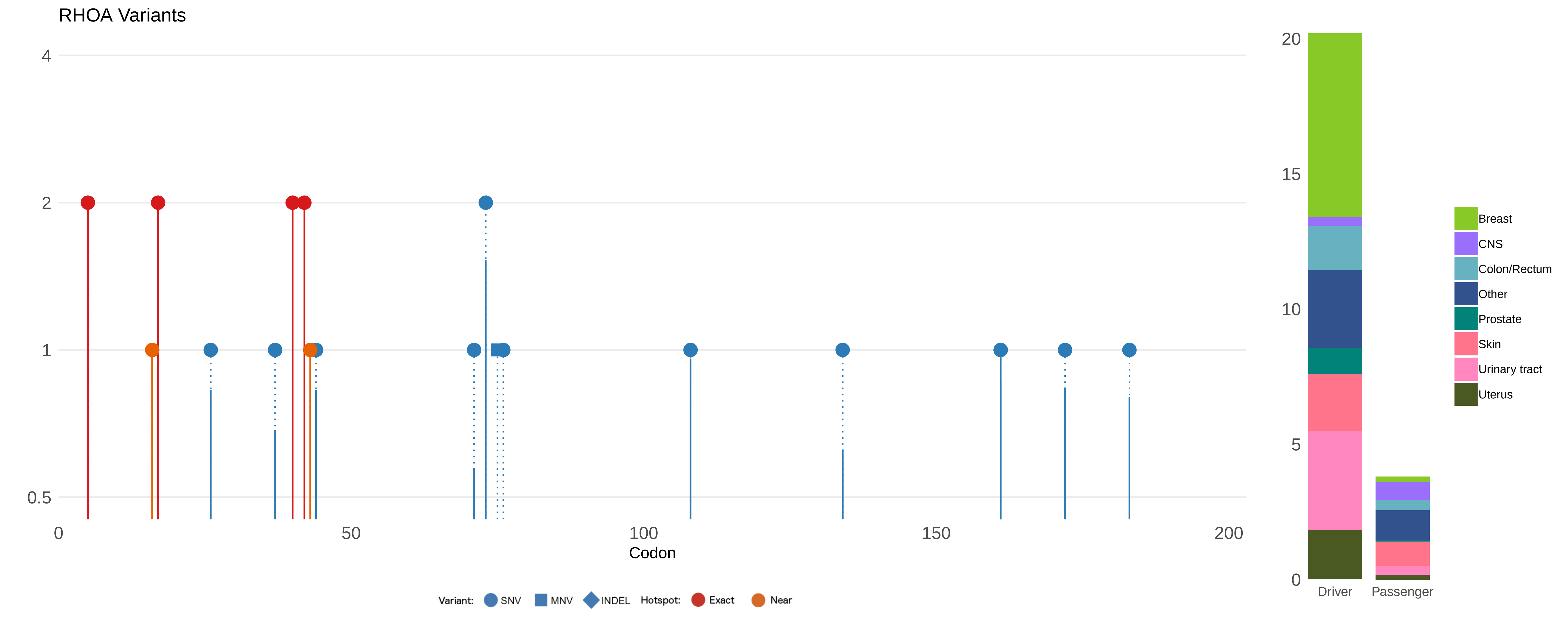
RHOA Variants
4
2
1
0.5
0
50
100
150
200
Codon
Variant:
SNV
MNV
INDEL
Hotspot:
Exact
Near
20
15
10
5
0
Driver
Passenger
Breast
CNS
Colon/Rectum
Other
Prostate
Skin
Urinary tract
Uterus

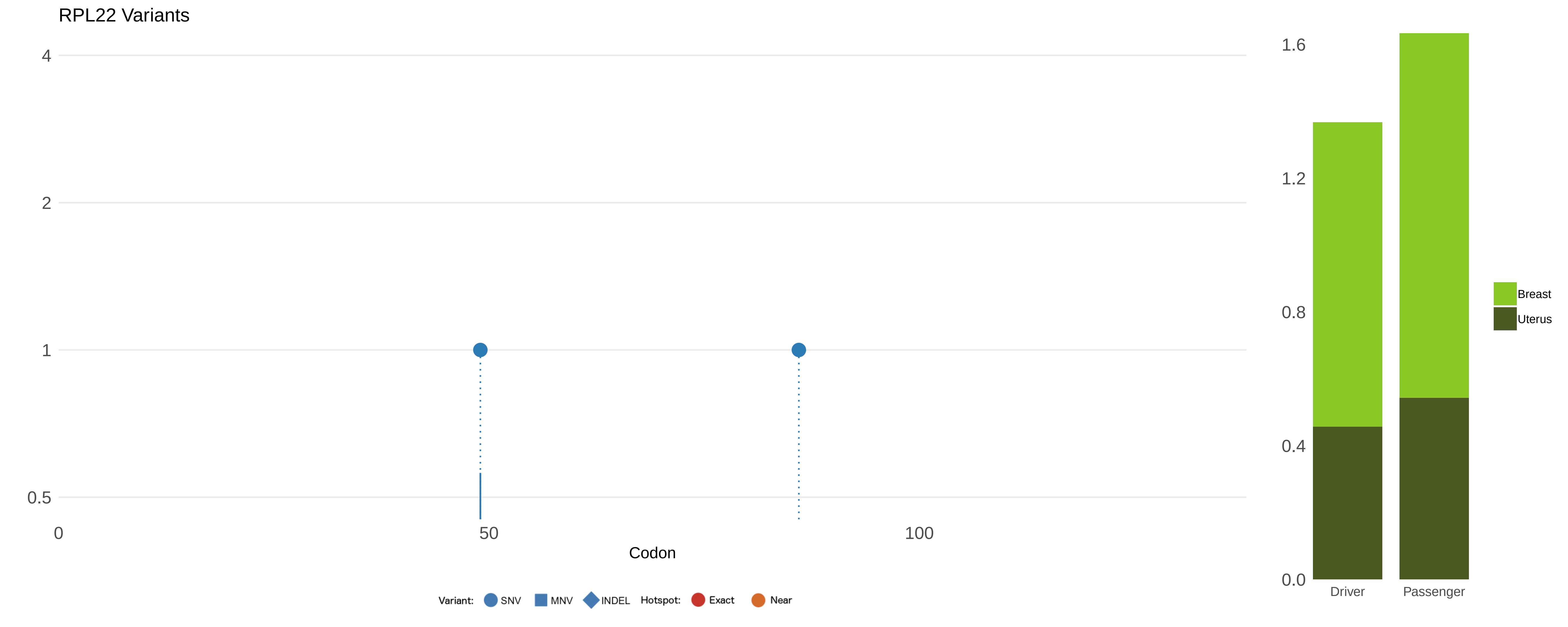
RPL22 Variants
4
2
1
0.5
0
50
100
Codon
Variant:
SNV
MNV
INDEL
Hotspot:
Exact
Near
1.6
1.2
0.8
0.4
0.0
Driver
Passenger
Breast
Uterus

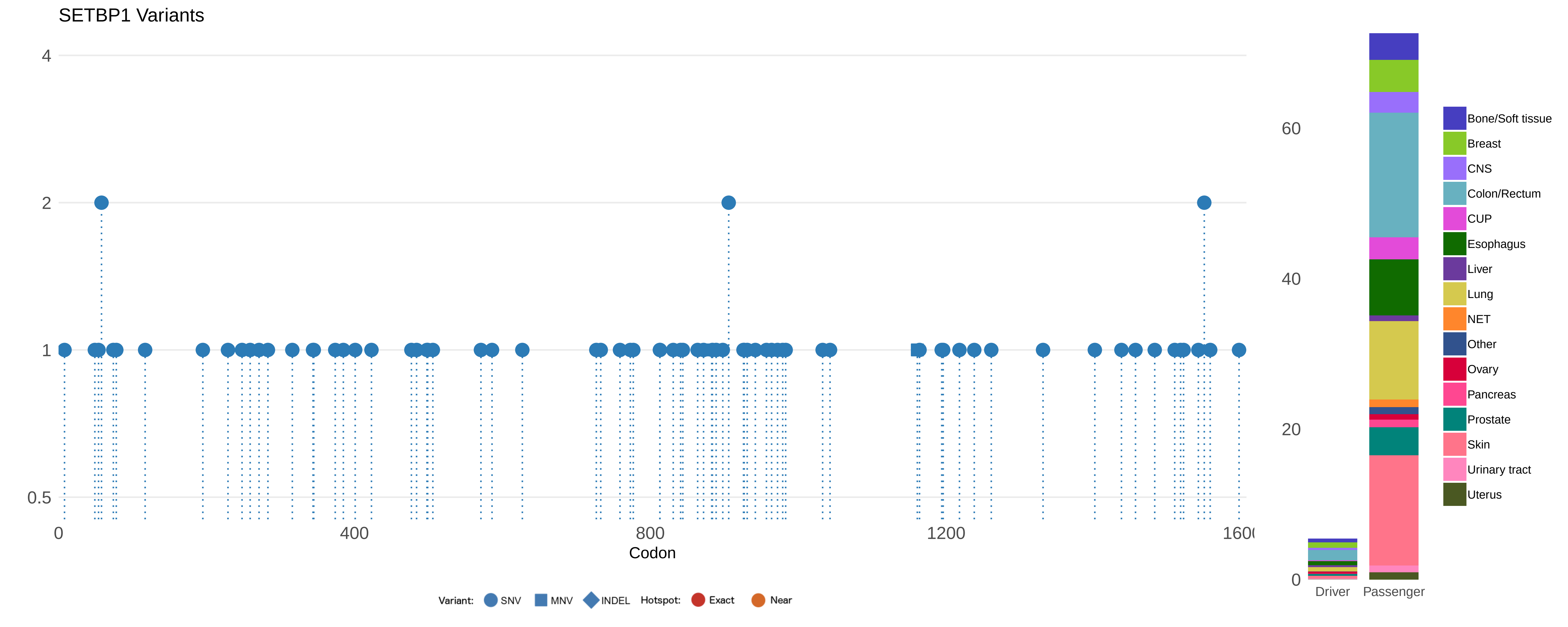

SETBP1 Variants
4
2
1
0.5
0
400
800
1200
1600
Codon
Variant: SNV MNV INDEL Hotspot: Exact Near
60
40
20
0
Driver
Passenger
Bone/Soft tissue
Breast
CNS
Colon/Rectum
CUP
Esophagus
Liver
Lung
NET
Other
Ovary
Pancreas
Prostate
Skin
Urinary tract
Uterus

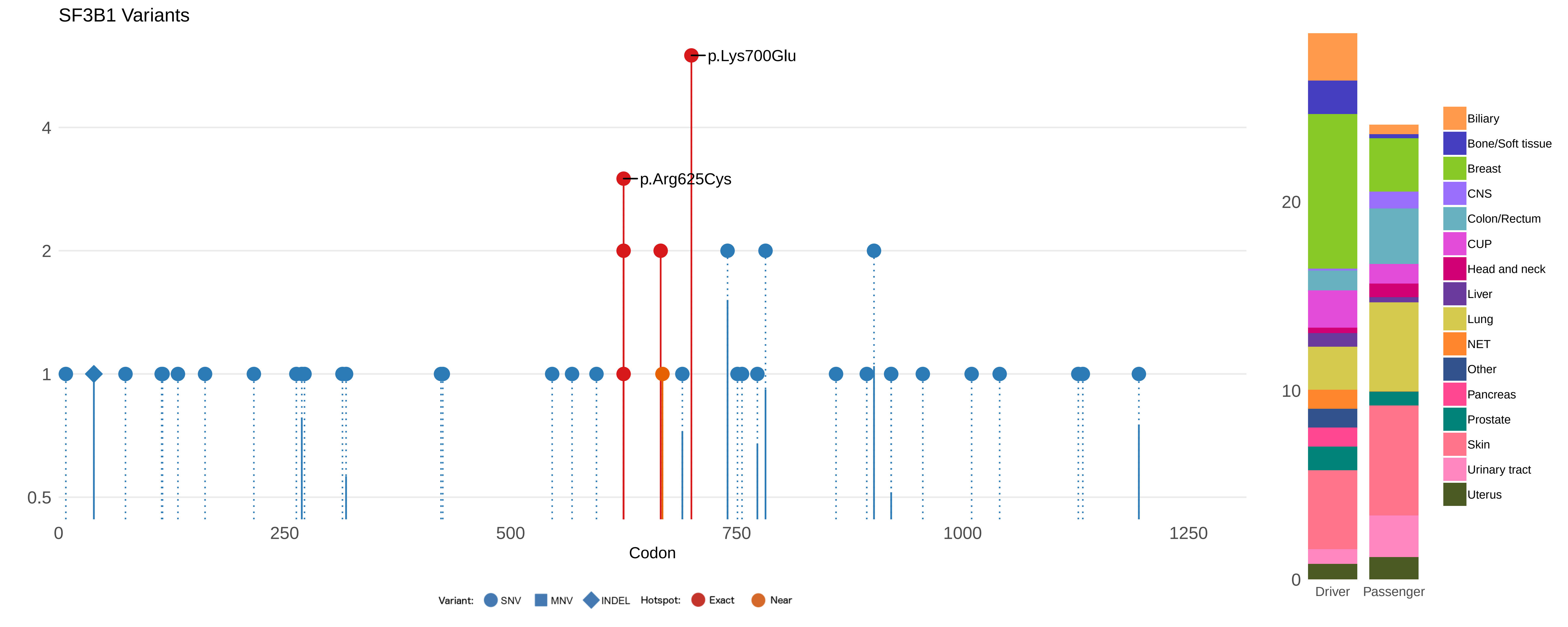
SF3B1 Variants
p.Lys700Glu
p.Arg625Cys
4
2
1
0.5
0
250
500
750
1000
1250
Codon
Variant: SNV MNV INDEL Hotspot: Exact Near
20
10
0
Driver
Passenger
Biliary
Bone/Soft tissue
Breast
CNS
Colon/Rectum
CUP
Head and neck
Liver
Lung
NET
Other
Pancreas
Prostate
Skin
Urinary tract
Uterus

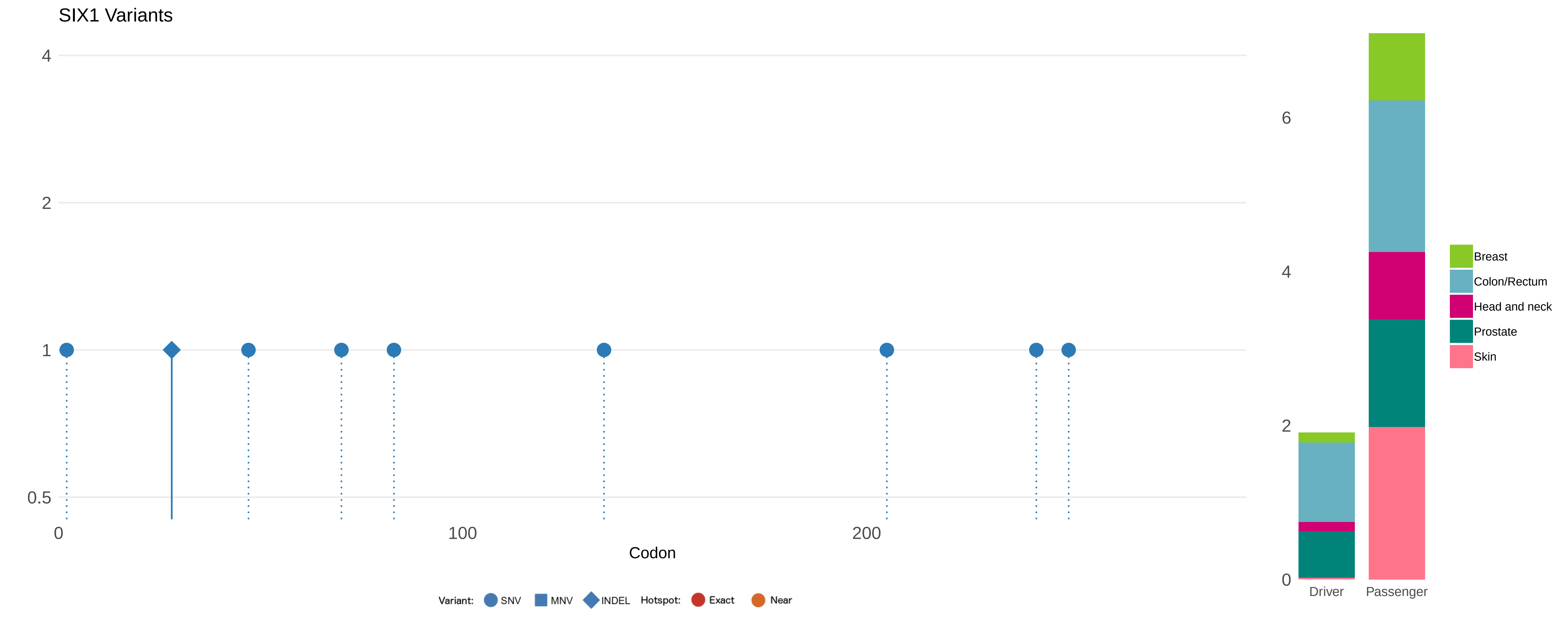
SIX1 Variants
4
2
1
0.5
0
100
200
Codon
Variant: SNV MNV INDEL Hotspot: Exact Near
6
4
2
0
Driver
Passenger
Breast
Colon/Rectum
Head and neck
Prostate
Skin

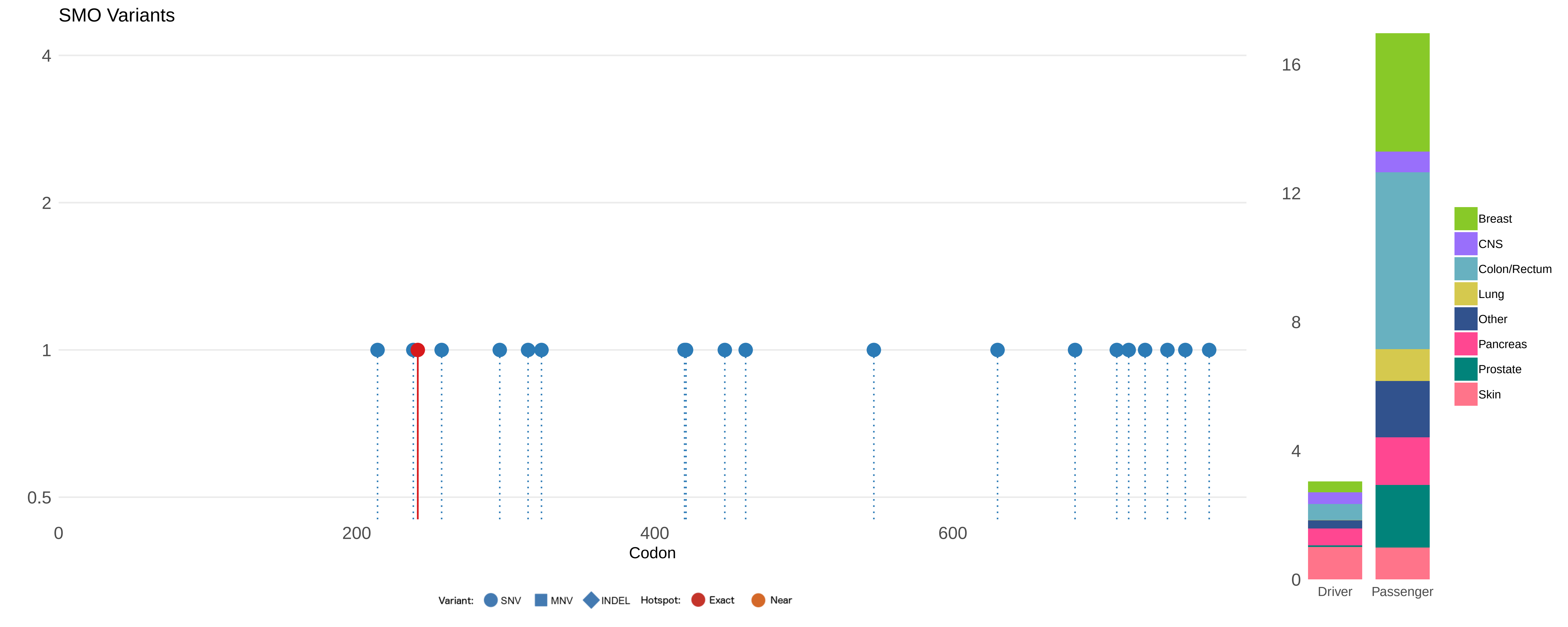
SMO Variants
4
2
1
0.5
0
200
400
600
Codon
Variant:
SNV
MNV
INDEL
Hotspot:
Exact
Near
16
12
8
4
0
Driver
Passenger
Breast
CNS
Colon/Rectum
Lung
Other
Pancreas
Prostate
Skin

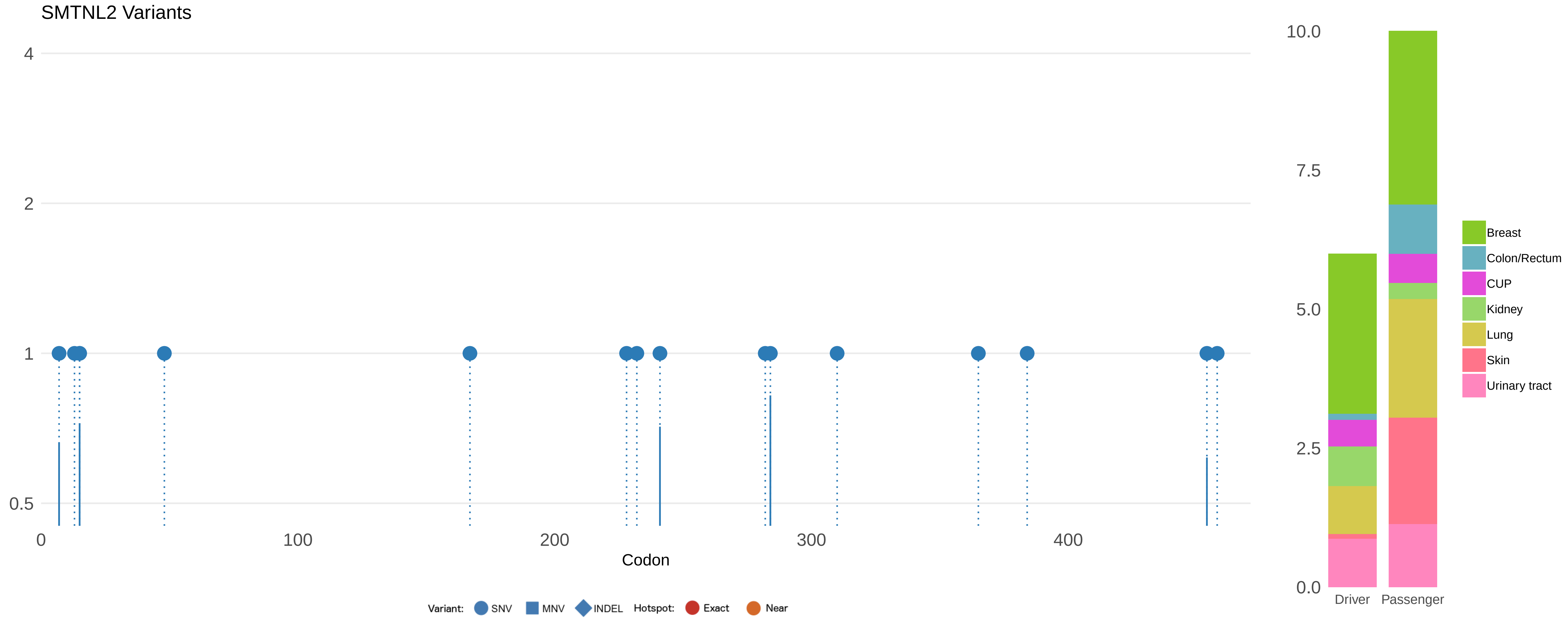

SMTNL2 Variants
4
2
1
0.5
0
100
200
300
400
Codon
Variant:
SNV
MNV
INDEL
Hotspot:
Exact
Near
10.0
7.5
5.0
2.5
0.0
Driver
Passenger
Breast
Colon/Rectum
CUP
Kidney
Lung
Skin
Urinary tract

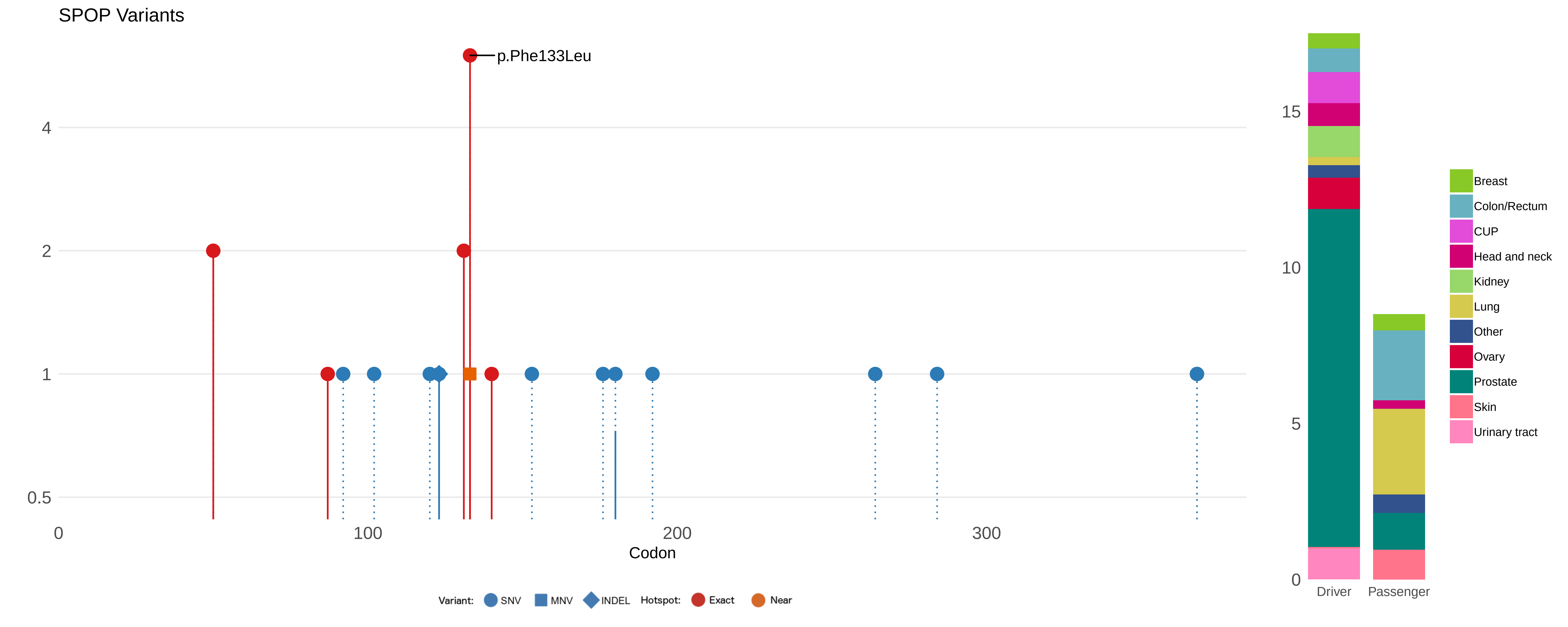

SPOP Variants
p.Phe133Leu
4
2
1
0.5
0
100
200
300
Codon
Variant: SNV MNV INDEL Hotspot: Exact Near
15
10
5
0
Driver
Passenger
Breast
Colon/Rectum
CUP
Head and neck
Kidney
Lung
Other
Ovary
Prostate
Skin
Urinary tract

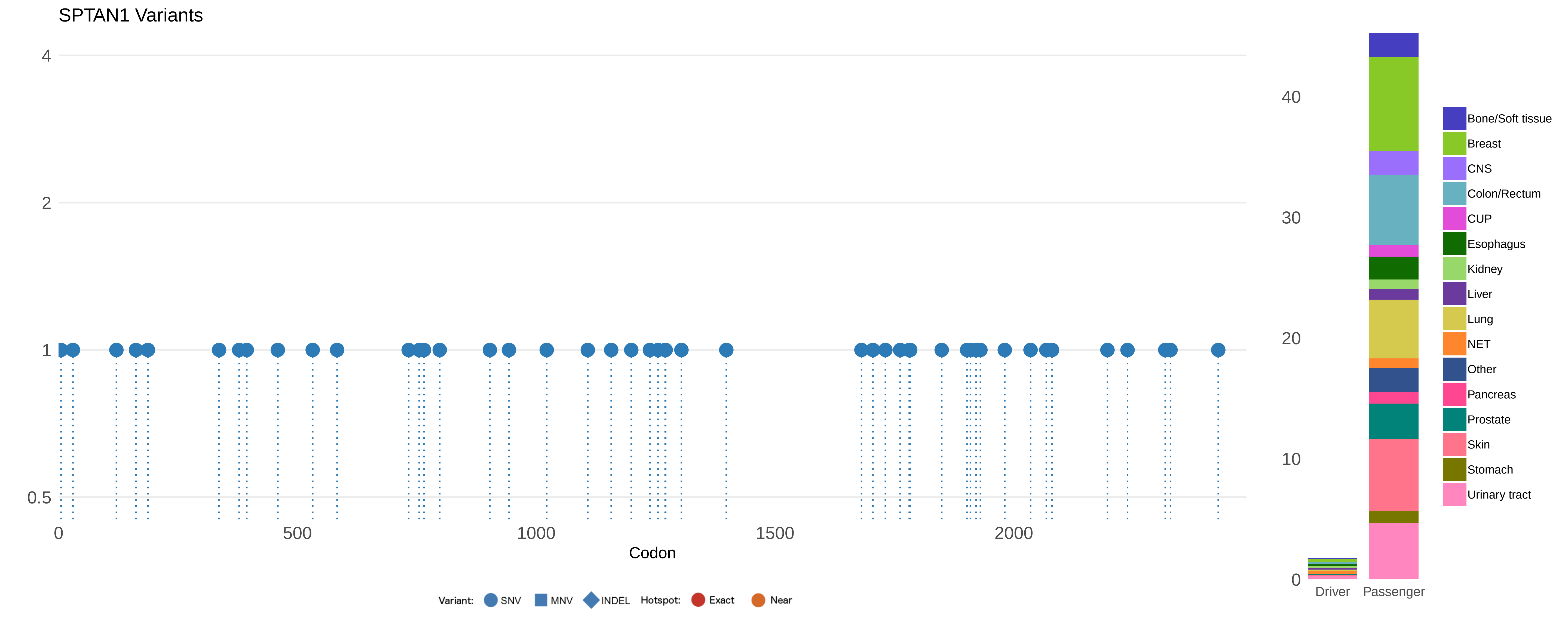

SPTAN1 Variants
4
2
1
0.5
0
500
1000
1500
2000
Codon
Variant: SNV MNV INDEL Hotspot: Exact Near
40
30
20
10
0
Driver
Passenger
Bone/Soft tissue
Breast
CNS
Colon/Rectum
CUP
Esophagus
Kidney
Liver
Lung
NET
Other
Pancreas
Prostate
Skin
Stomach
Urinary tract

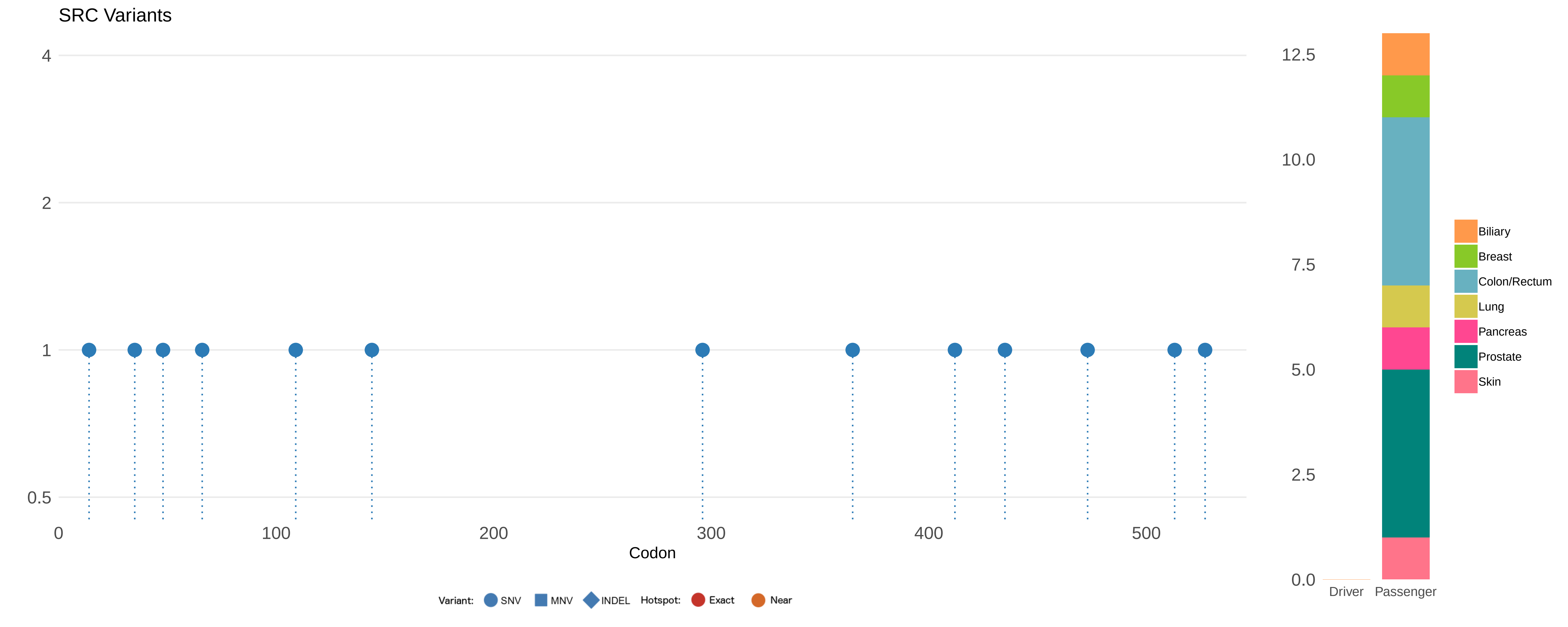

SRC Variants
4
2
1
0.5
0
100
200
300
400
500
Codon
Variant:
SNV
MNV
INDEL
Hotspot:
Exact
Near
12.5
10.0
7.5
5.0
2.5
0.0
Driver
Passenger
Biliary
Breast
Colon/Rectum
Lung
Pancreas
Prostate
Skin

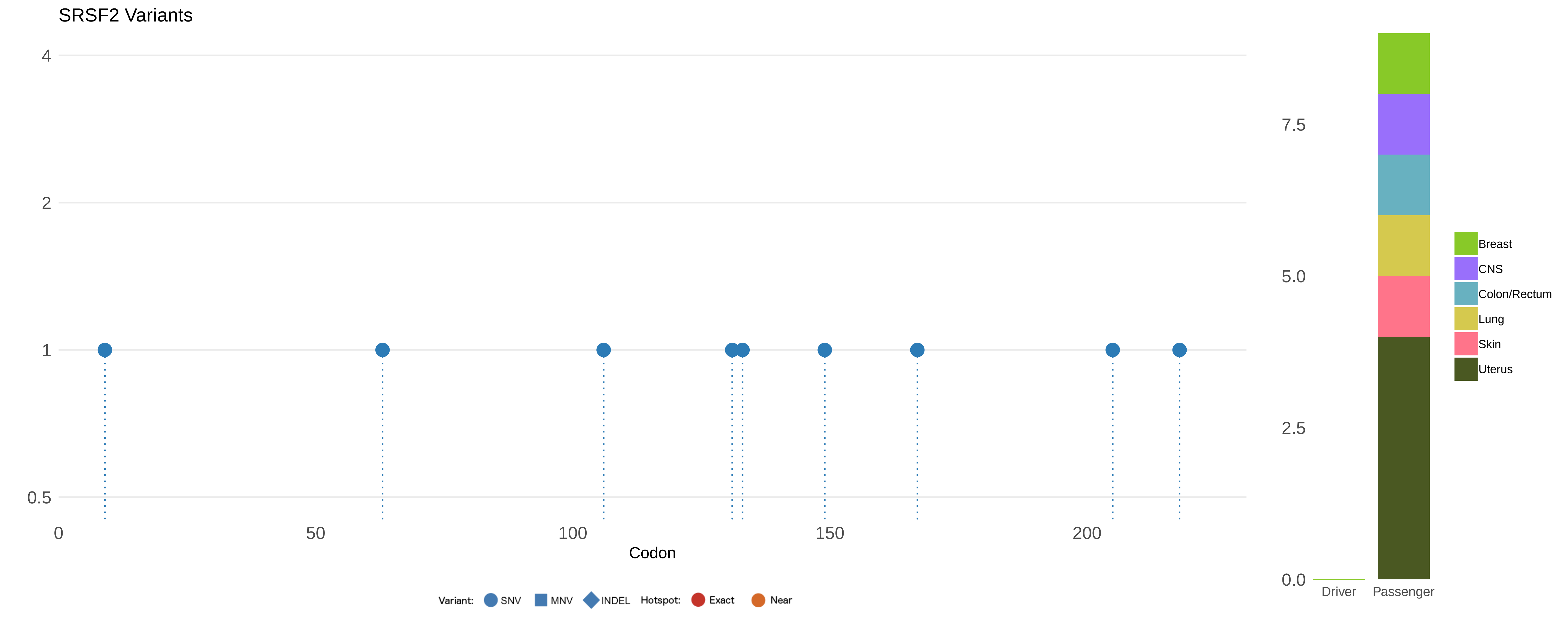

SRSF2 Variants
4
2
1
0.5
0
50
100
150
200
Codon
Variant:
SNV
MNV
INDEL
Hotspot:
Exact
Near
7.5
5.0
2.5
0.0
Driver
Passenger
Breast
CNS
Colon/Rectum
Lung
Skin
Uterus

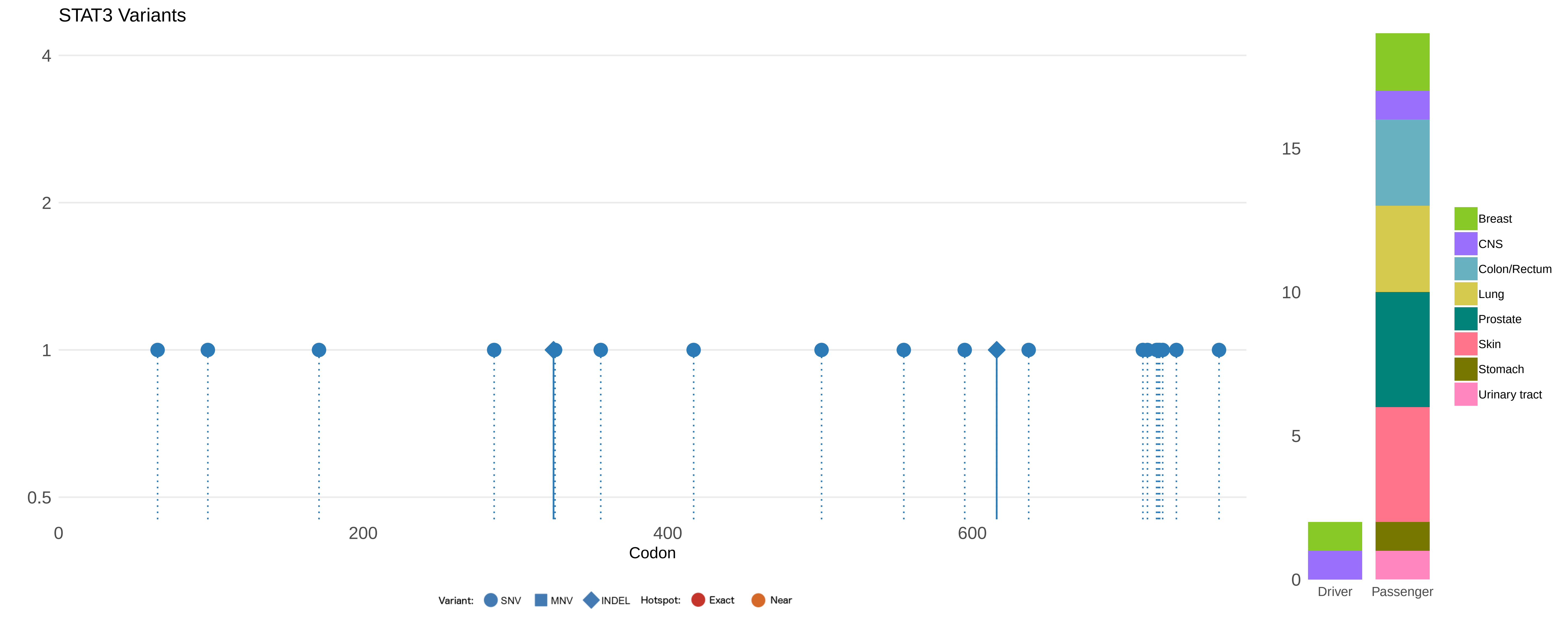

STAT3 Variants
4
2
1
0.5
0
200
400
600
Codon
Variant: SNV MNV INDEL Hotspot: Exact Near
15
10
5
0
Driver
Passenger
Breast
CNS
Colon/Rectum
Lung
Prostate
Skin
Stomach
Urinary tract

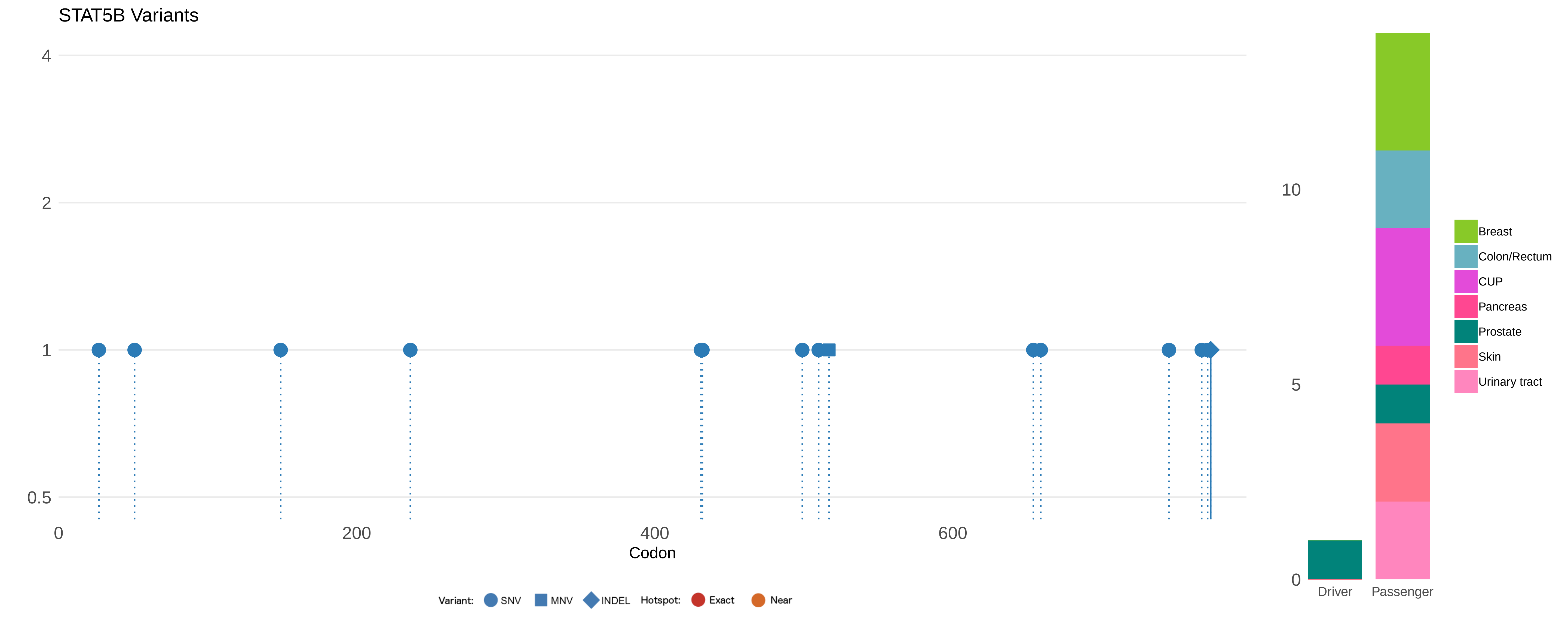

STAT5B Variants
4
2
1
0.5
0
200
400
600
Codon
Variant:
SNV
MNV
INDEL
Hotspot:
Exact
Near
10
5
0
Driver
Passenger
Breast
Colon/Rectum
CUP
Pancreas
Prostate
Skin
Urinary tract

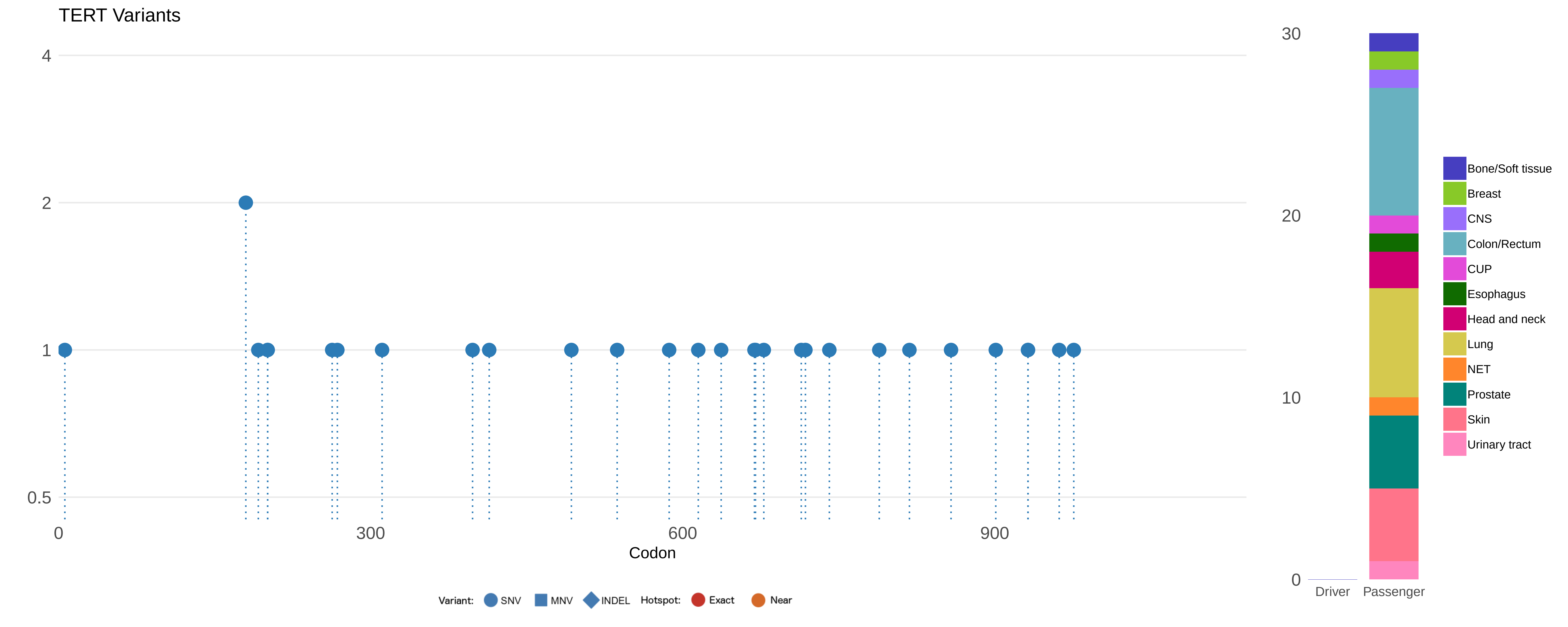

TERT Variants
4
2
1
0.5
0
300
600
900
Codon
Variant: SNV MNV INDEL Hotspot: Exact Near
30
20
10
0
Driver
Passenger
Bone/Soft tissue
Breast
CNS
Colon/Rectum
CUP
Esophagus
Head and neck
Lung
NET
Prostate
Skin
Urinary tract

TOP2A Variants

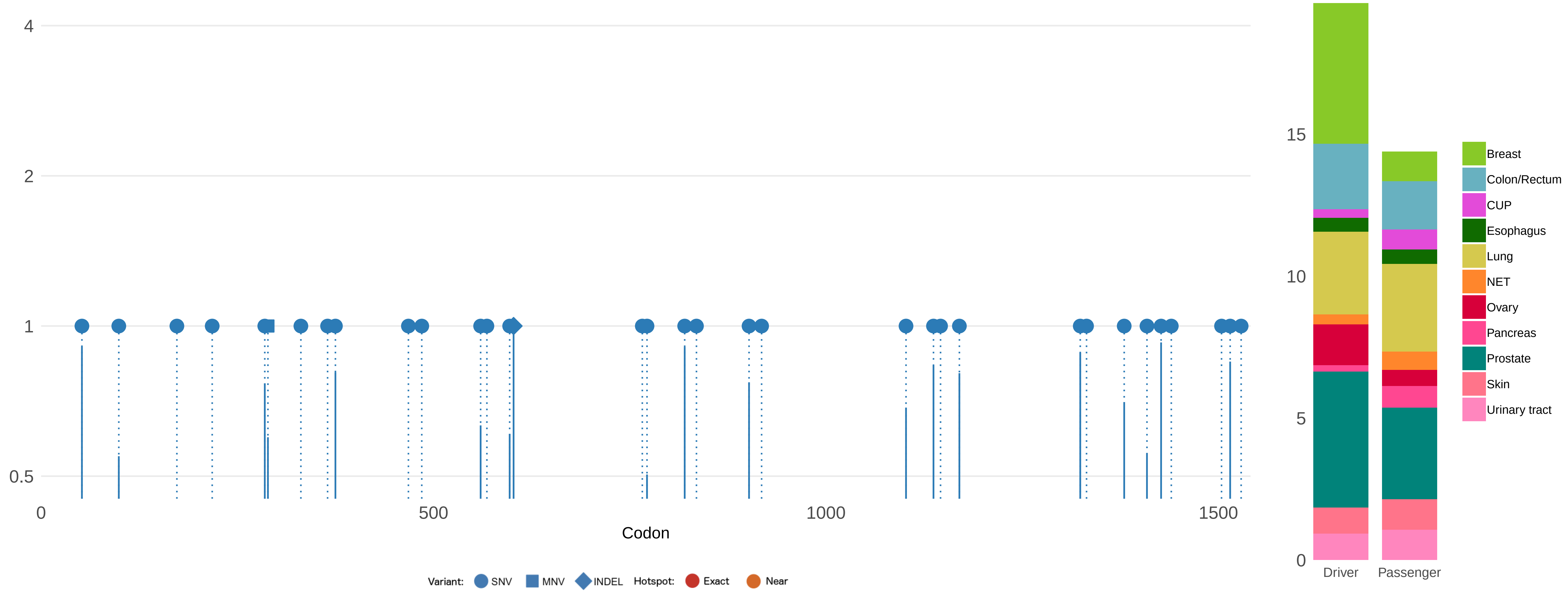

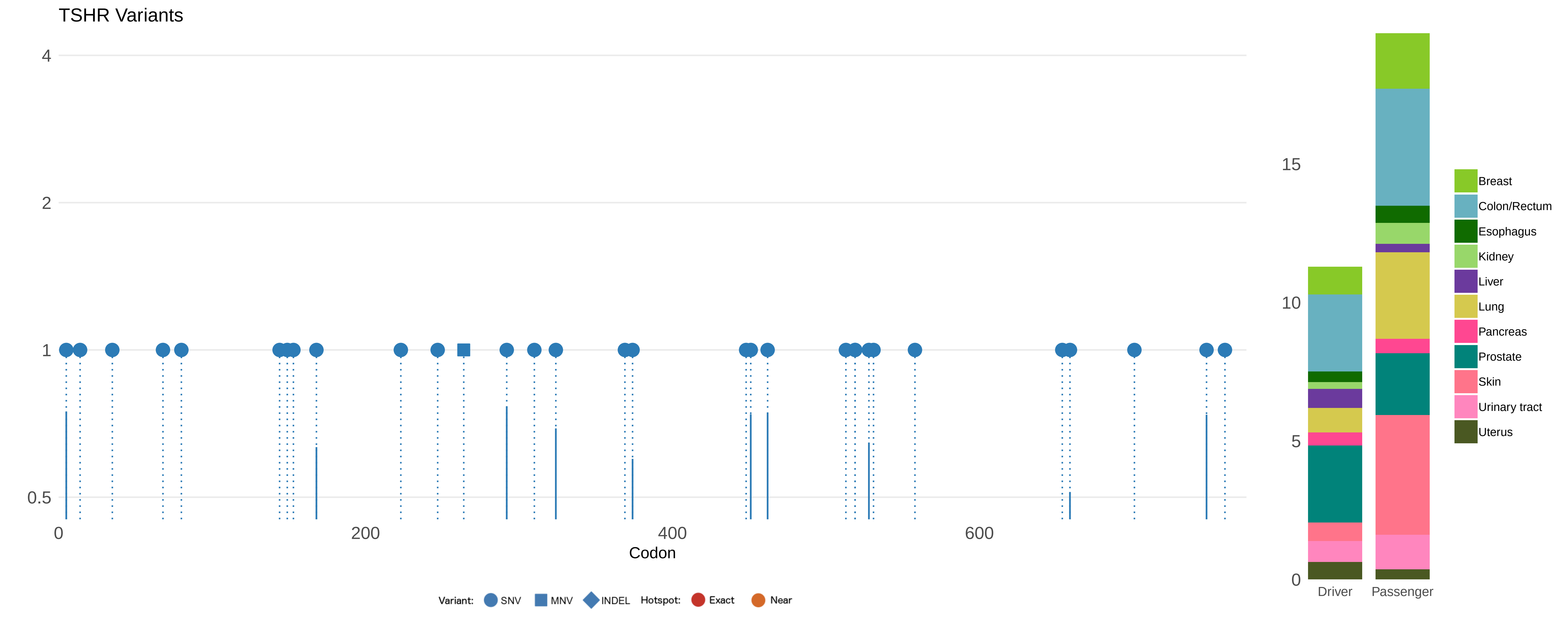
TSHR Variants
4
2
1
0.5
0
200
400
600
Codon
Variant:
SNV
MNV
INDEL
Hotspot:
Exact
Near
15
10
5
0
Driver
Passenger
Breast
Colon/Rectum
Esophagus
Kidney
Liver
Lung
Pancreas
Prostate
Skin
Urinary tract
Uterus

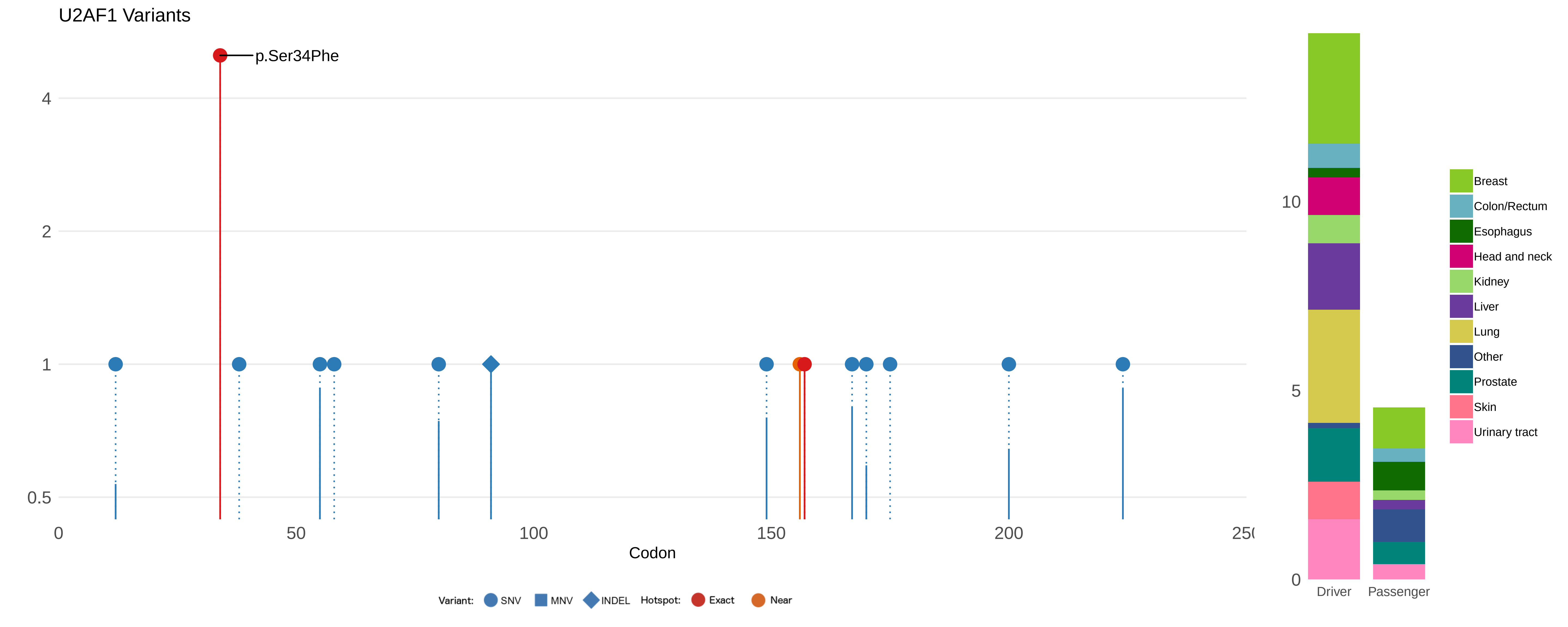
U2AF1 Variants
p.Ser34Phe
4
2
1
0.5
0
50
100
150
200
250
Codon
Variant:
SNV
MNV
INDEL
Hotspot:
Exact
Near
10
5
0
Driver
Passenger
Breast
Colon/Rectum
Esophagus
Head and neck
Kidney
Liver
Lung
Other
Prostate
Skin
Urinary tract

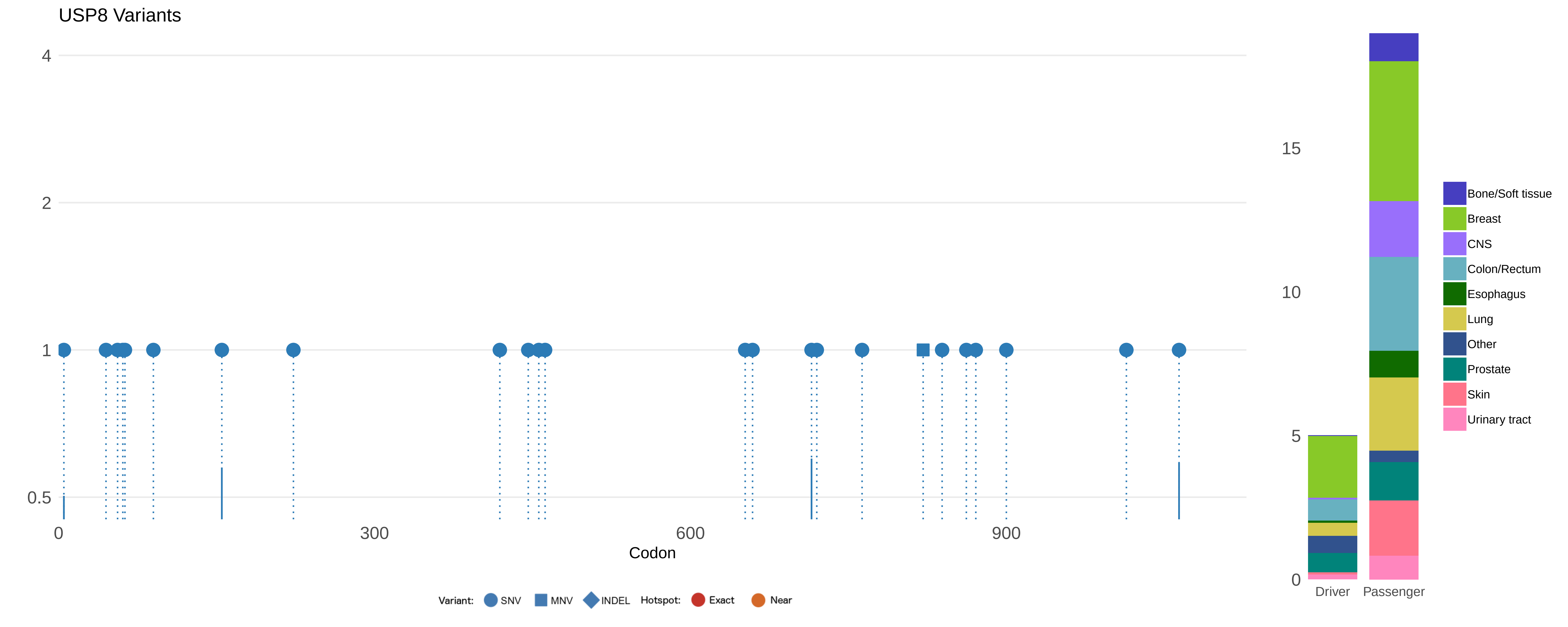

USP8 Variants
4
2
1
0.5
0
300
600
900
Codon
Variant: SNV MNV INDEL Hotspot: Exact Near
15
10
5
0
Driver
Passenger
Bone/Soft tissue
Breast
CNS
Colon/Rectum
Esophagus
Lung
Other
Prostate
Skin
Urinary tract

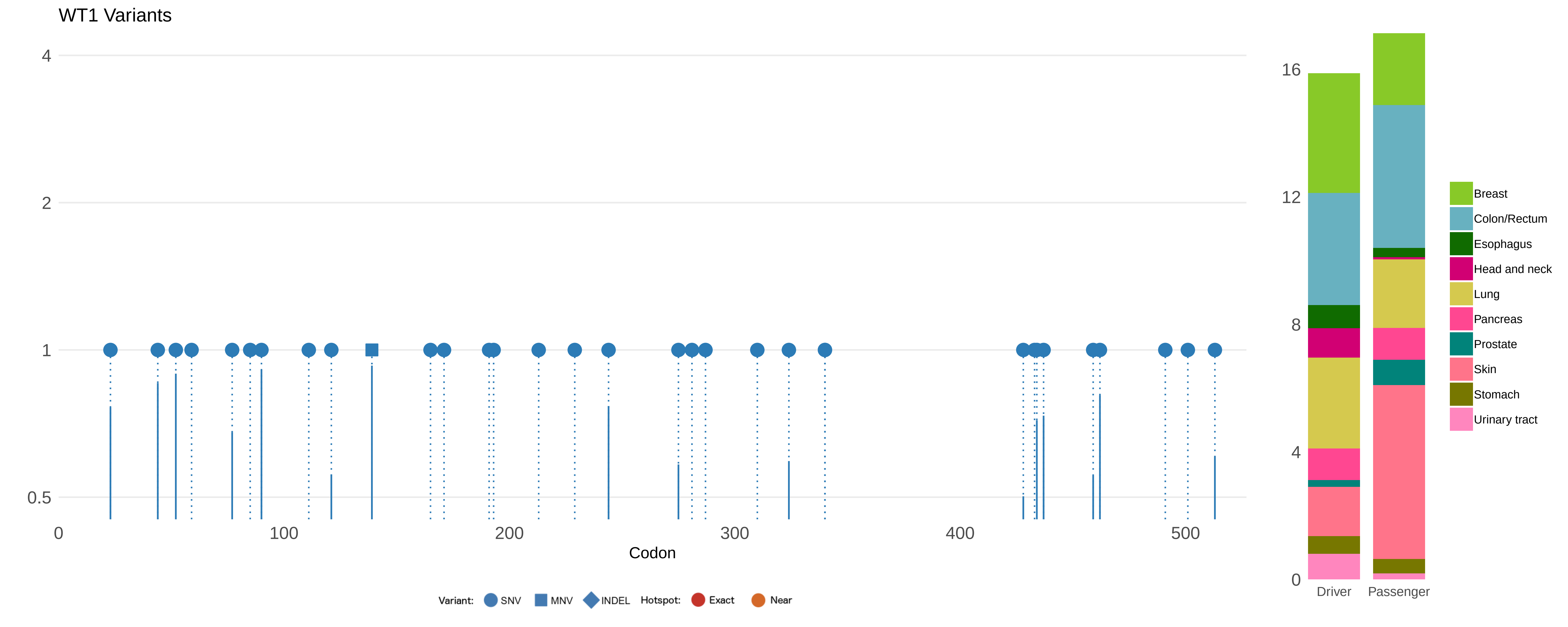
WT1 Variants
4
2
1
0.5
0
100
200
300
400
500
Codon
Variant:
SNV
MNV
INDEL
Hotspot:
Exact
Near
16
12
8
4
0
Driver
Passenger
Breast
Colon/Rectum
Esophagus
Head and neck
Lung
Pancreas
Prostate
Skin
Stomach
Urinary tract

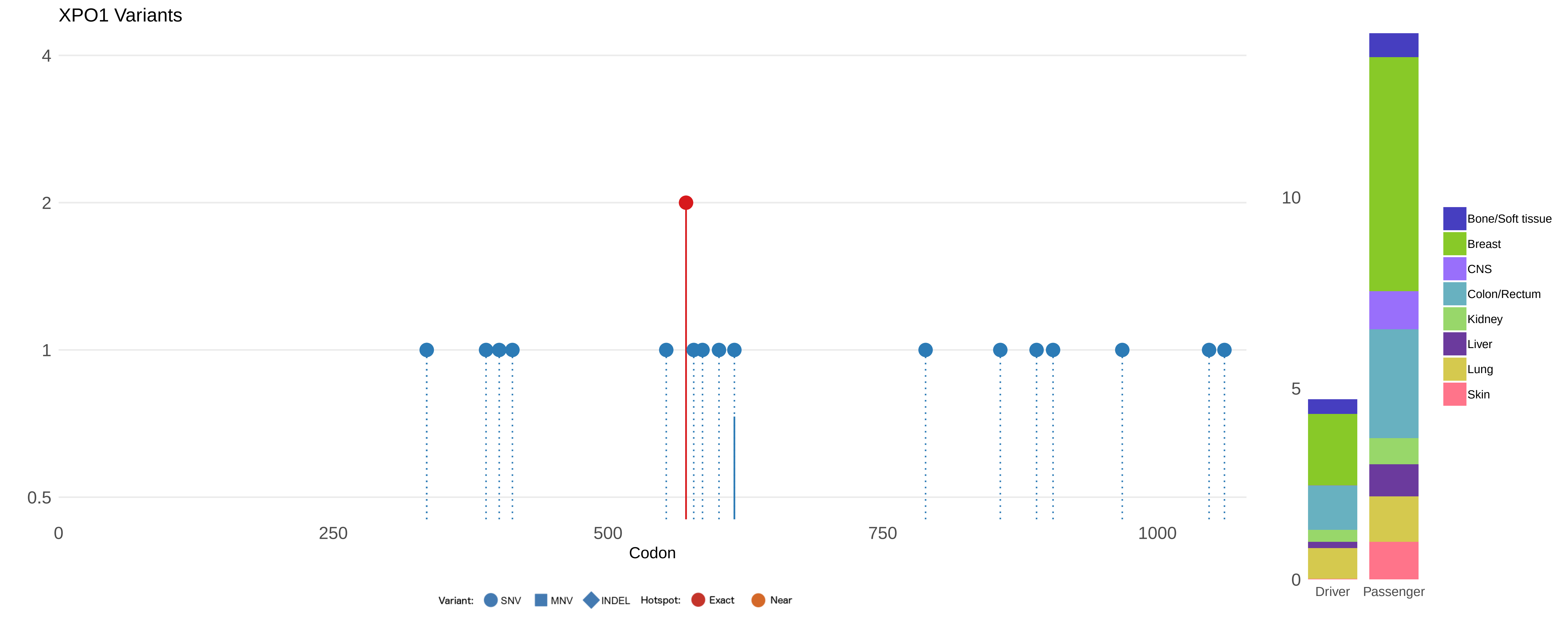
XPO1 Variants
4
2
1
0.5
0
250
500
750
1000
Codon
Variant:
SNV
MNV
INDEL
Hotspot:
Exact
Near
10
5
0
Driver
Passenger
Bone/Soft tissue
Breast
CNS
Colon/Rectum
Kidney
Liver
Lung
Skin

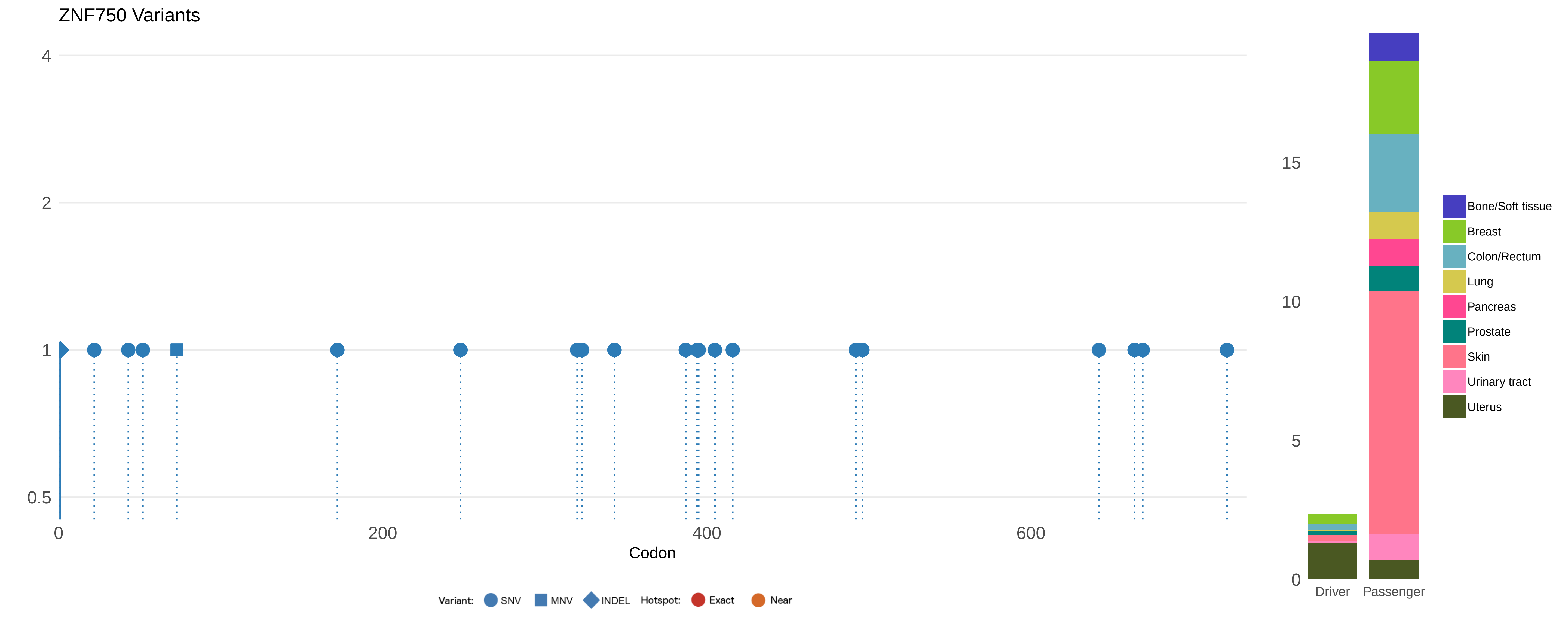
ZNF750 Variants
4
2
1
0.5
0
200
400
600
Codon
Variant: SNV MNV INDEL Hotspot: Exact Near
15
10
5
0
Driver
Passenger
Bone/Soft tissue
Breast
Colon/Rectum
Lung
Pancreas
Prostate
Skin
Urinary tract
Uterus
